# Palladacarboxamide Capping Reagents for Carbon Isotope Labeling and Pharmaceutical Diversification

**DOI:** 10.1002/anie.1188892

**Published:** 2026-05-19

**Authors:** Daniel V. Hoffmann, Anika Schick, Lasse Kjær, Vitus J. Enemærke, Clemens Kaussler, Jens Torp, Pablo Martínez‐Pardo, Charles S. Elmore, Troels Skrydstrup

**Affiliations:** ^1^ Interdisciplinary Nanoscience Center (iNANO) Department of Chemistry Aarhus University, Gustav Wieds Vej 14 Aarhus C Denmark; ^2^ Early Chemical Development, Pharmaceutical Sciences, R&D AstraZeneca, Gothenburg, Pepparedsleden 1 Mölndal Sweden; ^3^ Early Chemical Development, Pharmaceutical Sciences BioPharmaceuticals R&D, AstraZeneca Boston Massachusetts USA

**Keywords:** aryl carboxamides, carbonylation, carbon isotope labeling, oxidative addition complexes, palladium

## Abstract

Radiolabeling of bioactive molecules is essential for elucidating their pharmacokinetic and pharmacodynamic properties. Aryl carboxamides represent common constituents in a wide number of pharmaceuticals. Herein, we present a direct approach for the ^14^C‐labeling of this functional group facilitated by organometallic carboxamide capping reagents, based on palladium. Near stoichiometric amounts of ^14^CO are readily generated from a ^14^CO surrogate and exploited to assemble a range of palladium oxidative addition complexes. When subjected to Suzuki cross‐coupling conditions with boronic acids or esters, structurally diverse high‐value aryl carboxamides can be accessed directly. The protocol's robustness is demonstrated by late‐stage modification of amine‐displaying small drug molecules with ^14^C‐incorporation. Since the organometallic carboxamide complexes are air stable, this protocol provides a simple and direct means for carbon isotope labeling, which can easily be adapted to drug development programs.

## Graphical abstract

Herein, we present a method for carbon isotope labeling of arylamides via the coupling of air‐stable palladium carboxamide complexes and boronic ester/acids. A wide variety of functionalized boronic esters and acids have been coupled with simple palladium carboxamides complexes. Further development led to late‐stage derivatization of small amine‐displaying drug molecules and the direct ^14^C‐isotope labeling of these derivatives.

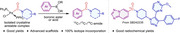

## 1 Introduction

**SCHEME 1:**
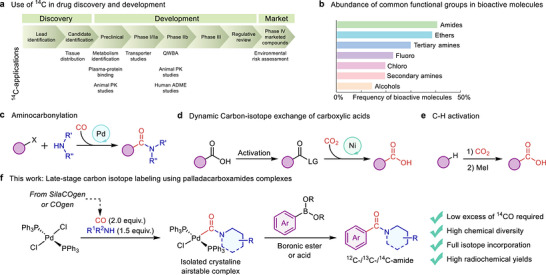
State of the art in isotope labeling of amides. (a) ^14^C in drug development [4]. (b) Abundance of common functional groups in bioactive molecules [5, 6]. (c) Aminocarbonylation catalyzed by transition metals. (d) Dynamic carbon‐isotope exchange is presented here as a two‐step procedure. (e) C─H activation utilizing CO_2_ as a C1 building block. (f) This work: Late‐stage labeling via oxidative addition complexes.

The introduction of radiolabels into strategic positions of biologically active molecules is a crucial step in drug development programs. Such studies provide essential data about their pharmacokinetic and pharmacodynamic properties, including drug metabolism and pharmacokinetics (DMPK), absorption, distribution, metabolism, and excretion (ADME), as well as their environmental fate. The use of ^14^C‐labeled drug candidate analogs is critical for these studies, highlighting the importance of isotopically labeled compounds in pharmaceutical research (Scheme 1a) [1, 2, 3, 4]. Due to the high metabolic stability of the amide functional group and its high frequency of use in bioactive molecules (Scheme 1b), it is a valuable target for carbon‐isotope labeling [5, 6]. Few last‐stage methods exist for the complete isotopic incorporation of the carbonyl carbon. Available late‐stage radiolabeling methods, for example, aminocarbonylation, require the synthesis of a halide‐containing precursor for the labeling step and often necessitate the use of excess CO or amines (Scheme 1c) [7, 8, 9, 10]. This is a significant drawback, as ^14^C is a precious and expensive isotope, and because the generation of hazardous radioactive waste should be minimized. Another approach involves direct carbon‐isotope exchange (CIE) methods, often relying on an extrusion‐insertion equilibrium mechanism of a functional group containing the isotope label. These methods suffer from isotope dilution, necessitating an excess of ^14^CO or ^14^CO_2_ to achieve >50% isotope incorporation [11, 12, 13, 14, 15, 16, 17]. Examples are the work performed by the groups of Baran and Audisio (Scheme 1d) [11, 14]. Similarly, C─H activation of aryl‐ and benzyl‐containing substrates, as exemplified by the groups of Shigeno and Perry, forms the corresponding carboxylic acid from CO_2_‐releasing molecules (Scheme 1e) [18, 19]. No method has been disclosed for the direct CIE of aryl carboxamides. Previous work performed in our group has attempted to tackle these issues by employing a two‐chamber COware system with ex situ generated CO [20, 21, 22]. A wide variety of bench‐stable Pd(II) oxidative addition complexes (OACs) have previously been reported, either by oxidative addition of a Pd(0) species or the exchange of one X‐type ligand for another in a Pd(II) species [23, 24, 25, 26]. Some more advanced, bench‐stable Pd(II) OACs have been prepared by the Buchwald group. These were applied in stoichiometric amounts for late‐stage pharmaceutical diversification [27]. In 2015, the same team demonstrated the worth of Pd(II) OACs for selective cysteine conjugation on peptides [28]. Inspired by previous work employing palladacarboxylates as capping reagents for labeling aryl carboxylates [29], we envisioned an analogous method for the specific isotope incorporation into aryl carboxamides. To make the method broadly applicable, we set out to design a reaction bypassing the need for glovebox or Schlenk equipment while utilizing only bench‐stable reagents. We developed a method employing a near stoichiometric amount of CO, released from either of the two CO surrogates COgen or SilaCOgen, and other readily accessible starting materials. Furthermore, we showcase how these organometallic capping reagents tolerate a wide range of functional groups on either side of the amide group. Finally, diluted ^14^C‐labeling experiments have been performed on active pharmaceutical ingredients, exemplifying how the method can be directly applied to future drug development programs.

## 2 Results and Discussion

### 2.1 Initial Findings

**SCHEME 2:**
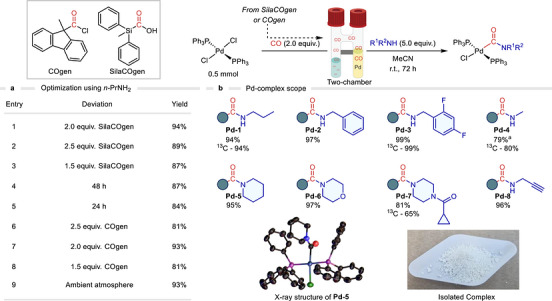
Synthesis of oxidative addition complexes. Yields were determined by purification and corrected by ^31^P‐qNMR and are reported as the average of two experiments. (a) Optimization of the synthesis of **Pd‐1** at a 0.5 mmol reaction scale. (b) Scope of simple palladacarboxamides. X‐ray structure of **Pd‐5** with ellipsoids drawn at the 50% probability level. Hydrogen atoms and co‐crystallized solvent molecules are omitted for clarity. Color code: C (black), N (blue), O (red), Cl (green), P (purple), Pd (turquoise) [34]. ^a^ 2.5 h instead of 72 h.

A method for synthesizing *n*‐propyl palladacarboxamides was pioneered by Ugozolli [25] and later by Yamamoto [30]. Inspired by this work, we set out to develop a method applying near‐stoichiometric amounts of CO generated from SilaCOgen or COgen [31, 32] in a two‐chamber COware reactor (Scheme 2) [33]. Gratifyingly, we found that subjecting PdCl_2_(PPh_3_)_2_ to five equivalents of *n*‐propylamine under an atmosphere of only 2.0 equivalents of ex situ generated CO from SilaCOgen at room temperature for 72 h afforded the crystalline complex **Pd‐1** in 94% yield. Purification was achieved by a simple filtration of the reaction mixture and washing with cold diethyl ether and pentane (Scheme 2a, entry 1). The reaction demonstrated resilience as reducing reaction times and altering the CO equivalents only slightly diminished the yield (84%–89%, entries 2–5). In the interest of radiolabeling, COgen was employed for CO release, as previous attempts in our team to synthesize ^14^C‐SilaCOgen were unsuccessful. Exchanging SilaCOgen to COgen [33] also afforded **Pd‐1** in an excellent yield of 93% (entry 7), and similarly, altering the CO equivalents only slightly diminished the yield (81%, entries 6 and 8). Finally, the reaction demonstrated versatility by affording the product in an excellent yield under ambient atmosphere (93%, entry 9), making this chemistry accessible without the need for glovebox or Schlenk line techniques. A detailed description of the optimization studies is available in Section S2. The benzyl‐containing palladacarboxamide **Pd‐2** was synthesized in an excellent yield of 97%.

**SCHEME 3:**
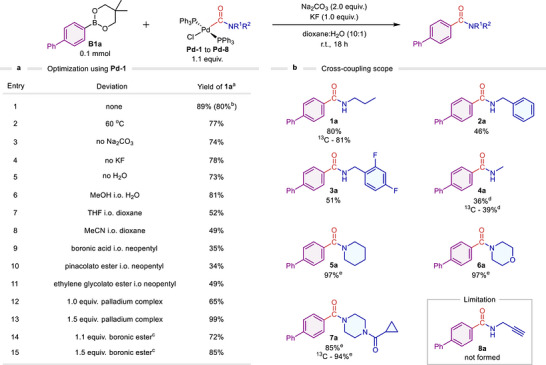
Conditions for the Suzuki cross‐coupling reaction. (a) Optimization of coupling between **Pd‐1** and 4‐biphenyl neopentyl boronic ester. ^a^Yields calculated based on ^1^H‐qNMR with 1,3,5‐trimethoxybenzene as an internal standard. ^b^Isolated yield. ^c^Yields based on **Pd‐1** as the limiting reagent. (b) Scope of coupling products between palladium carboxamide complex (**Pd‐1** to **Pd‐8**) and **B1a**. Yields are reported as the average of two experiments. Where relevant ^12^C and ^13^C yields are reported individually. ^d^40°C used instead of room temperature. ^e^60°C instead of room temperature.

Next, we set out to evaluate the reactivity with different amines of pharmaceutical relevance. Fluorinated *N*‐benzylamides, exemplified by dolutegravir [35] and cabotegravir [36], as well as *N*‐methylamides, are structural entities found in 13 of the top 200 small molecule drugs by sales in 2024 [37]. 2,4‐Fluorinated benzylamide **Pd‐3** was prepared in an excellent yield of 99%. Synthesizing the methyl palladacarboxamide (**Pd‐4**) proved difficult but was achieved by employing methylamine (9 w/w% in acetonitrile), leading to the corresponding palladium complex **Pd‐4** in just 2.5 h in an 80% yield. Extending the reaction time diminishes the yield for this specific palladium complex. To our delight, secondary amines were also compatible with the reaction conditions. Thus, employing the cyclic secondary amines such as piperidine, morpholine, and cyclopropyl carbonyl piperazine (substructure of the anti‐cancer active pharmaceutical ingredient, olaparib) yielded the complexes **Pd‐5**, **Pd‐6**, and **Pd‐7** in high yields of 65%–95%. Finally, propargylamine was employed to form **Pd‐8** in a 96% yield. Some limitations of the methodology are found in Section S4.3, which include ethylenediamines, PEG chains, and amine salts. The use of aniline and *p*‐toluidine failed to yield the products in any reasonable amounts, likely due to the lower basicity of these nucleophiles. For further information, see Section S4.3. With these palladium complexes in hand, we set out to study the reaction conditions and limitations of complex **Pd‐1** in Suzuki coupling reactions [38, 39, 40]. Previously, our group developed conditions for synthesizing methyl carboxylates exploiting the corresponding methyl palladacarboxylates with neopentyl boronic esters [29]. These conditions were slightly modified and adapted to the synthesis of the aryl carboxamides. Optimized conditions include the use of 1.1 equivalents of complex **Pd‐1** relative to the neopentyl boronic ester **B1a**, using 2.0 equivalents Na_2_CO_3_ and 1.0 equivalent KF in a dioxane:H_2_O (10:1) solvent mixture at room temperature for 18 h (Scheme 3a, entry 1). Deviation of the reaction conditions, including higher temperature (entry 2), exclusion of base (entries 3–4) and water (entry 5), as well as examining different organic solvents, including MeOH, THF, or MeCN (entries 6–8), all led to lower yields (49%–81%). Other boron reagents, including boronic acid, pinacolato boronic ester, and ethylene glycolato boronic ester, were investigated as substrates. Here, the neopentyl boronic esters performed the best for this transformation (entries 9–11), which could be attributed to two main factors: the parasitic side‐reaction forming the corresponding [1,1'‐biphenyl]‐4‐ol is suppressed, and higher conversion of this type of boronic ester. The parasitic [1,1'‐biphenyl]‐4‐ol possibly originates from the hydrolysis of the palladium intermediate, after the transmetalation step. The formation of the phenol from the boronic ester has previously been reported by Lei and coworkers from the hydroxypalladium (II) aryl complex [41, 42]. A similar intermediate could be formed after the transmetalation of the palladacarboxamide and the aryl boronic ester, although the precise mechanism for this transformation is speculative.

Finally, we explored the effect of the stoichiometry between the palladium complex **Pd‐1** and the boronic ester **B1a**, as both coupling partners are of high value, especially for the incorporation of the ^14^C radiolabel. Decreasing the **Pd‐1** complex loading from 1.1 equivalents to 1.0 equivalents relative to the biphenyl boronic ester **B1a** resulted in a drop in yield to 65% (entry 12). Increasing the loading of the palladium complex to 1.5 equivalents relative to the boronic ester gratifyingly resulted in the quantitative conversion of the boronic ester to the amide (entry 13). Similarly, increasing the stoichiometry of the boronic ester with respect to the palladacarboxamide increases the yield, although not as significantly (entries 14 and 15). This underlines the variability of the reaction with respect to the most valuable component in the reaction.

### 2.2 Scoping Coupling Reaction

With acceptable reaction conditions for the Suzuki coupling in hand, we successfully expanded the coupling reaction of the palladium complexes **Pd‐1** to **Pd‐8** with 4‐biphenyl boronic ester **B1a** for a diverse scope (Scheme 3b). *n*‐Propylamide **1a** was isolated in an excellent yield of 80%. *N*‐Benzylamide **2a** and 1,4‐difluorobenzylamide **3a** were formed in moderate yields of 46% and 51%, respectively. *N*‐Methylamide **4a** initially presented a challenge with incomplete conversion of the boronic ester and was isolated in a 25% yield. Increasing the temperature to 40°C significantly increased the yield to 36%–39%, while also increasing the conversion to the parasitic [1,1'‐biphenyl]‐4‐ol. The secondary palladacarboxamides **Pd‐5** to **Pd‐7**, which demonstrated no conversion at room temperature, delivered excellent results when the reaction temperature was increased to 60°C. Both the piperidine benzamide **5a** and the morpholine benzamide **6a** could be isolated in 97% yield, while the cyclopropyl carbonyl piperazine **7a** was isolated in an excellent yield of 90%. Finally, the propargyl palladium complex **Pd‐8** led to full conversion to the undesired [1,1'‐biphenyl]‐4‐ol even when carrying out the coupling reaction at varying temperatures (20°C, 30°C, 40°C, or 60°C). The inability to isolate the desired coupling product could be the result of Pd(0), generated from the reductive elimination step, undergoing an oxidative addition into the activated carbon‐nitrogen bond of the *N*‐acyl propargyl amine.

**SCHEME 4:**
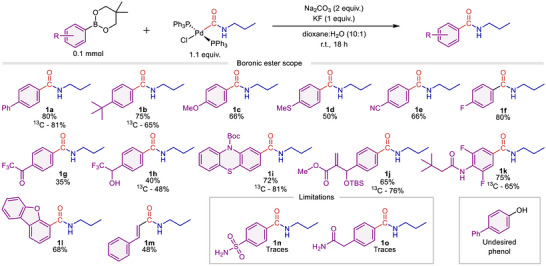
Functional group tolerance of the aryl boronic ester coupling partner. Yields are reported as the average of two experiments. Where relevant ^12^C and ^13^C yields are reported individually.

Having demonstrated various functional groups on the palladacarboxamide to be acceptable for the cross‐coupling reaction conditions (Scheme 3b), we next explored the scope of the boronic ester coupling partner (Scheme 4). Different electronic properties were tolerated as exemplified by the substitution of various electron‐donating groups (**1a–d**) and electron‐withdrawing groups (**1e‐g**) in the *para* position of the aryl boronic ester. The products containing *p*‐phenyl **1a** and *p‐tert‐*butyl **1b** were both formed in good yields of 80%–81% and 65%–75%, respectively, whereas *p*‐methoxy‐ and *p*‐thiomethylphenyl boronic esters produced **1c** and **1d** in slightly lower yields of 66% and 50%. The *N*‐propylamides *p*‐nitrilephenyl **1e** and *p*‐fluorophenyl **1f** were obtained in good yields of 66% and 80%, while *p*‐trifluoroacetylphenyl **1g** was formed in an acceptable yield of 35%. Free alcohols can be tolerated to some degree, as illustrated with **1h** being formed in a modest yield of 40%–48%. More elaborate motifs, such as a *N*‐Boc‐protected phenothiazine **1i**, a terminal alkene containing **1j**, and a secondary amide containing 4‐(3,3‐dimethylbutanam‐ido)‐3,5‐difluoro‐*N*‐propylbenzamide **1k**, were formed in good yields of 65%–81%. The tolerance of substituents in the *ortho*‐position is exemplified by *N*‐propyldibenzo‐[*b,d*]furan‐4‐carboxamide **1l** in a good yield of 68%. A clear trend in yield was not observed with regard to the functional groups. Finally, the boronic ester is not limited to aryl groups; neopentyl styryl boronic ester could be converted into the corresponding cinnamamide **1m** in a yield of 48%. On the other hand, an example with a primary amide and a sulfonamide afforded only trace amounts of the respective products **1n** and **1o**. When the method was applied to 2‐cyclopentyl pinacol boronic ester and 2‐cyclopentyl boronic acid with either **Pd‐1** or **Pd‐5**, at 60°C, no conversion of the boron species was observed, and both the boronic ester and acid were recovered.

### 2.3 Late‐Stage Functionalization of API's

**SCHEME 5:**
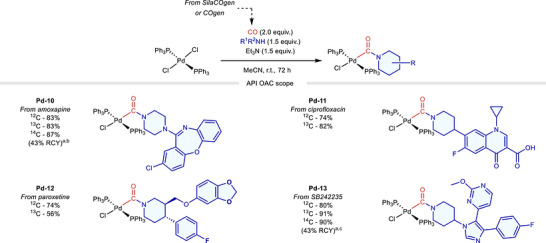
Scope of secondary amine‐displaying drug molecules for palladacarboxamide complex formation. Yields were determined by weight after purification and corrected by ^31^P‐qNMR. ^a^All ^14^C labeling experiments were conducted with a 91.5:8.5 mixture of ^12^COgen:^14^COgen as the CO source to reduce radioactive waste. ^b^83.6 MBq isolated, 8.7% ^14^C incorporation. ^c^83.0 MBq isolated, 8.7% ^14^C incorporation.

Finally, palladacarboxamides were synthesized using small drug molecules containing an amine, enabling both pharmaceutical diversification and carbon isotope labeling of these drug analogs. The general reaction conditions were modified to accommodate more complex amines. In contrast to the scope where simple amine building blocks were investigated, we sought to apply near‐stoichiometric amounts of the more valuable complex amine‐displaying small drug molecules. This could be realized by the addition of triethylamine to avoid using the more valuable amine‐containing small drug molecules as the base required for the deprotonation step. The stoichiometry of the carbon monoxide precursor was kept low to maximize the radiochemical yield (RCY). Validating the method for pharmaceutically relevant compounds, containing secondary amines, several approved and investigational bioactive molecules were chosen, namely amoxapine **10** [43], ciprofloxacin **11** [44], paroxetine **12** [45], and SB242235 **13** [46]. **10**, **12**, and **13** led to complete conversion to **Pd**‐**10**, **Pd**‐**12**, and **Pd**‐**13** in good to excellent yields (Scheme 5). In contrast, **Pd‐11** was completely soluble in the reaction medium. Thus, evaporation of the solvent was necessary before purification. The synthesis of **Pd‐11** led to incomplete conversion of PdCl_2_(PPh_3_)_2_ but still allowed the isolation of the product in high yields of 74%–82%. Further, secondary amine small drug molecules were applied with low conversions (see Section S4.3). Notably, the geometry of the isolated complexes is depicted as mainly being the *trans*‐isomer, as supported by the crystal structure of **Pd‐5**. With these results in hand, we set out to synthesize the palladacomplexes ^14^ **C‐Pd**‐**10** and ^14^ **C‐Pd**‐**13**. Since the release of CO from COgen is sensitive to air, we developed a method using a three‐way T‐bore connected to a nitrogen line and a vacuum pump to mimic a Schlenk line in the laboratory handling radioactive material. Using a dilution of 8.5:91.5 ^14^COgen:^12^COgen as the CO precursor to reduce radiochemical waste, the product was afforded in 87% and 90% yield for ^14^ **C‐Pd‐10** and ^14^ **C‐Pd‐13**, respectively. The radiochemical yield [47] was determined by triplicate scintillation counting to be 43% for both ^14^ **C‐Pd‐10** and ^14^ **C‐Pd**‐**13**, compared to the theoretical maximum of 50% radiochemical yield for two equivalents of ^14^CO used.

**SCHEME 6:**
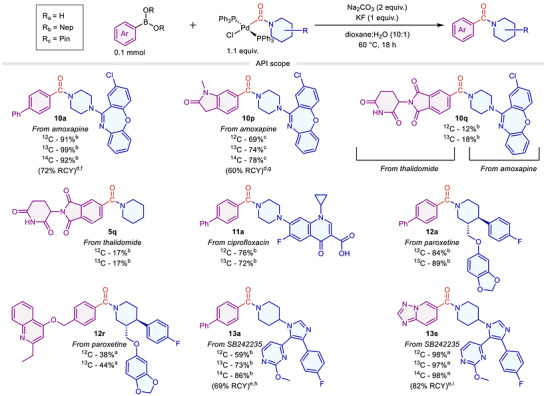
Coupling of different boronic esters/acids to the Pd‐[^12/13/14^CO‐API] complexes. ^a^Employing boronic acid. ^b^Employing boronic neopentyl ester. ^c^Employing boronic pinacol ester. ^d^8.7% dilution of ^12^C:^14^C‐complex used. ^e^8.7% dilution of ^12^C:^14^C‐complex used. ^f^15.3 MBq isolated, RCP >99%. ^g^13.5 MBq isolated, RCP 99%. ^h^15.9 MBq isolated, RCP >99%. ^i^17.8 MBq isolated, RCP >99%.

The reactivity of the palladacarboxamide drug complexes was examined in the coupling step (Scheme 6). First, the model boronic ester **B1a** was employed to form analogs of the corresponding small drug molecules. To our satisfaction, the coupling with the palladium complexes **Pd‐10**, **Pd‐11**, **Pd‐12**, and **Pd‐13** led to high yields of 91%–95% of the derivatives of amoxapine **10a**, 72%–76% of the ciprofloxacin **11a**, 84%–89% of paroxetine **12a**, and 59%–73% of SB242235 **13a**. To highlight the usefulness of this chemistry to more advanced building blocks, the 4‐biphenylboronic ester **B1a** was exchanged for more structurally complex boronic ester and boronic acid building blocks. This afforded the amoxapine **10p** in 69%–74% yields, the paroxetine **12r** in 38%–44% yields, and the SB242235 **13s** in 97%–98% yields. Finally, we coupled **Pd‐5** and **Pd‐10** with a thalidomide [48] neopentyl boronic ester derivative to form **5q** and **10q** in 17% and 12%–18% yield, respectively. With these promising results, we demonstrate that the method can be adapted to coupling reactions without the need for a glovebox when employing the amoxapine‐derived ^14^ **C‐Pd‐10** and the SB242235‐derived ^14^ **C‐Pd‐13**. With the organometallic complex ^14^ **C‐Pd‐10**, we successfully prepared the radiolabeled compound ^14^ **C‐10a** in a 92% yield and 72% radiochemical yield, while the synthesis of ^14^ **C‐**10p afforded the product in a 78% yield and 60% RCY. Switching to the SB242235‐derived complex ^14^ **C‐Pd‐13**, the labeled product ^14^ **C‐13a** was formed in an excellent 86% yield and 69% RCY, whereas ^14^ **C‐13s** was isolated in an excellent 98% yield and 82% RCY, compared to the theoretical maximum radiochemical yield of 91%. These results showcase the excellent efficiency of this last‐stage radiolabeling method. **10a**, **13a**, and **13s** were all found to have a radiochemical purity (RCP) greater than 99%, while **10p** was found to have a RCP of 99%.

## 3 Conclusion

In conclusion, we have developed bench‐stable palladacarboxamide capping reagents from simple, commercially available starting materials utilizing a two‐chamber reactor. Starting from primary or secondary amines, PdCl_2_(PPh_3_)_2_ and ex situ generated CO, these palladacarboxamides are readily formed. We have demonstrated the applicability of the reaction outside an argon‐filled glovebox, both under ambient atmosphere when employing the carbon monoxide‐releasing molecule SilaCOgen, and under a nitrogen atmosphere using Schlenk conditions with the alternative ^14^CO‐releasing reagent, ^14^COgen. The method was expanded to the synthesis of pharmaceutically relevant aryl carboxamides by incorporating small bioactive amine molecules into oxidative addition complexes and pharmaceutically relevant scaffolds. Furthermore, we developed a method applicable for a radiochemical laboratory and performed illustrative reactions with the use of diluted ^14^COgen, providing high to excellent yields of the desired aryl carboxamides displaying high radioisotope incorporation and radiochemical yields. We believe this chemistry will open the door for new opportunities for the rapid labeling of important bioactive and pharmaceutically relevant molecules.

## Author Contributions

**Daniel V. Hoffmann**: conceptualization, investigation, writing – original draft, methodology, writing – review and editing. **Anika Schick**: investigation, methodology, writing – review and editing. **Lasse Kjær**: investigation, methodology. **Vitus J. Enemærke**: investigation, methodology, writing – review and editing. **Clemens Kaussler**: investigation, writing – review and editing, methodology. **Jens Torp**: investigation, methodology. **Pablo Martínez–pardo**: investigation, writing – review and editing, methodology, project administration, supervision. **Charles S. Elmore**: conceptualization, investigation, writing – review and editing, project administration, supervision. **Troels Skrydstrup**: conceptualization, investigation, writing – review and editing, methodology, project administration, supervision.

## Conflicts of Interest

T.S. is co‐owner of SyTracks A/S, which commercializes the two‐chamber system (COware) and SilaCOgen.

A.S., P.M., and C.E. are AstraZeneca employees and may hold shares and/or stock options in the company.

## Supporting information



The authors have cited additional references within the Supporting Information section [25, 29, 49, 50, 51, 52, 53, 54, 55, 56, 57].
**Supporting File**: anie72717‐sup‐0001‐SuppMat.pdf.

## Data Availability

The data that support the findings of this study are available from the corresponding author upon reasonable request.
