# Supplementary material for: Palladacarboxamide Capping Reagents for Carbon Isotope Labeling and Pharmaceutical Diversification
Source: Angew Chem Int Ed Engl. 2026 May 19;65(28):e1188892. doi: 10.1002/anie.1188892 (PMC13340519; doi:10.1002/anie.1188892)

Palladacarboxamide Capping Reagents for Carbon Isotope Labelling and Pharmaceutical Diversification

Daniel V. Hoffmann,[a] Anika Schick,[a,b] Lasse Kjær,[a] Vitus J. Enemærke,[a] Clemens Kaussler,[a] Jens Torp,[a] Pablo Martínez-Pardo[b], Charles S. Elmore[c], and Troels Skrydstrup*[a]

[a]Interdisciplinary Nanoscience Center (iNANO), Department of Chemistry, Aarhus University, Gustav Wieds Vej 14, 8000 Aarhus C, Denmark

[b]Early Chemical Development, Pharmaceutical Sciences, R&D, AstraZeneca, Gothenburg, Pepparedsleden 1, 43183 Mölndal, Sweden

[c]Early Chemical Development, Pharmaceutical Sciences, R&D, AstraZeneca, Boston, MA, 35 Gatehouse Drive Waltham, MA 02451

Table of contents

1 General methods 3
2 Optimisation table 5
3 General procedures 6
4 Experimental section 9
4.1 Starting materials 9
4.2 Palladacarboxamide complexes 13
4.3 Additional Pd-complexes 21
4.4 Cross-coupling scope 23
4.5 Boronic ester scope 26
4.6 API scope 32
5 ^{14}C-analysis 43
6 Crystallographic appendix 53
6.1 Data collection 53
6.2 Structure solution and refinement 53
7 References 55
8 NMR spectra of compounds 56
8.1 Additional spectra 177

1 General methods

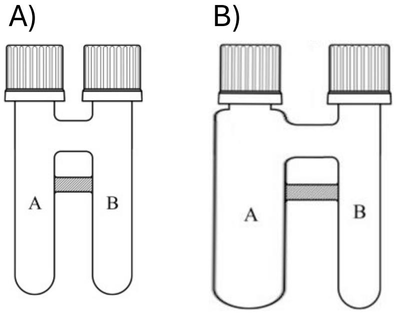

Figure S1: A) COware for 0.1 mmol scale reactions. B) COware for 0.5 mmol scale reactions.

All reactions employing CO-release molecules (CORMs) were performed in COware reactors with 2x10 mL chambers for a 0.10 mmol test-scale (Figure S1A) and 20+10 mL chambers for a 0.5 mmol scale (Figure S1B). The COware reactors were sealed with single-use silicone/PTFE seals, stabilised with PTFE discs and screwcaps. All commercially available chemicals were used without additional purification, except $(PPh_3)_2PdCl_2$ from BLDpharm, which was purified by washing with -30 °C Et_2O and -30 °C pentane. A glovebox kept under an argon atmosphere was used for some experiments. All anhydrous reactions were performed in flame or oven-dried glassware under an argon atmosphere unless otherwise specified. Some solvents were purified using MB-SPS-800 and used directly or degassed by purging with argon and stored over molecular sieves (3Å). All ^{14}C-experiments were performed with anhydrous solvent purchased from Sigma Aldrich and degassed with nitrogen for 5 minutes before use. Duplicated experiments are reported as their average yields. Analytical thin-layer chromatography (TLC) was performed on silica-coated aluminium plates (Merck Kieselgel 60 F_{254}), which were visualised under UV-radiation (254 nm) and by staining with $KMnO_4$ afterwards. flash column chromatography was performed on interchim puriflash XS 520Plus automatic column machines unless otherwise specified. Manual flash column chromatography was performed using silica gel 60 (230-400 mesh). Purification by HPLC was performed on a Waters 2545 Quaternary Gradient Module equipped with a Waters 2489 UV/Vis detector with an XBridge Prep C-18 (30×250 mm column, 10 μm OBD) using either MeOH or MeCN as the apolar solvent and 0.2v/v% NH_4OH in H_2O as the polar solvent.

NMR analyses were acquired using a Bruker Avance (III) or Avance Neo spectrometers with a frequency of 400, 500, or 600 MHz for ^{1}H-NMR, 101 MHz ^{13}C-NMR, 376 MHz for ^{19}F-NMR, and 162 MHz for ^{31}P-NMR, respectively. Chemical shifts are reported in parts per million (ppm) relative to the residual solvent signal ($CDCl_3$: 7.26 ppm for ^{1}H-NMR and 77.16 ppm for ^{13}C-NMR). The following abbreviations are used to indicate the multiplicity in NMR spectra: m, multiplet; s, singlet; d, doublet; dd, doublet of doublets; t, triplet; q, quartet; p, pentet; sext, sextet; td, triplet of doublets; dt, doublet of triplets; qd, quartet of doublets. HR-MS was recorded on a Bruker Maxis Impact spectrometer (LC TOF, ESI). LC-MS and LC-UV was measured on a Waters Aquity UPLC system. Detection was accomplished with a QDA detector running ESI +/- scanning 100-800 da at 5 Hz and a PDA detector running 220-350 nm with 1.2 nm resolution measuring 20 points/s. The gradient used was 0.2 min 10 % MeCN, 3.5 min 10-99 % MeCN, 0.3 min 99 % MeCN using one of the two eluent and column combinations shown below. 1. pH 10 method: MeCN-NH4/NH4CO3:water employing a BEH C18 column (2.1 x 50 mm with 1.7 μm particles) S3 2. pH 4: MeCN-formic acid:water employing a HSS C18 column (2.1 x 50 mm with 1.8 μm particles). This was used to determine the isotope incorporation of ^{14}C. Radiochemical purity was determined on a Waters Acquity UPLC equipped with a Waters Xbridge C18 (3.5 μm, 4.6×100 mm) column with in-line radioactivity detection using a Lab logic Beta Ram 5 and liquid scintillation counting using a Hidex 300 SL with Ultima Gold cocktail. The specific activities of the products were

determined by LC-MS using the Excel sheet ”IsoPat[2]” with an algorithm developed by Kroutil and co-workers to deconvolute the MS signals.[1]

2 Optimisation table

Supplementary Table 1: All recorded data for the optimisation of **Pd-1**.

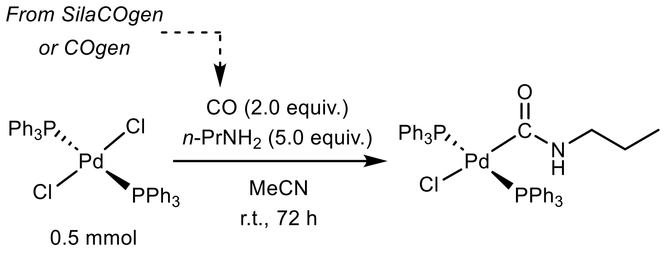

a Optimisation using n-$PrNH_2$

Entry	Deviation	Yield
1	2.0 equiv. SilaCOgen	94%
2	2.5 equiv. SilaCOgen	89%
3	1.5 equiv. SilaCOgen	87%
4	48 h	87%
5	24 h	84%
6	48 h + 2.5 equiv. SilaCOgen	88%
7	24 h + 2.5 equiv. SilaCOgen	79%
8	2.5 equiv. COgen	81%
9	2.0 equiv. COgen	93%
10	1.5 equiv. COgen	81%
11	48 h + 2.5 equiv. COgen	84%
12	24 h + 2.5 equiv. COgen	83%
13	Ambient atmosphere	93%
14	Ambient atmosphere + 2.5 equiv. SilaCOgen	87%

3 General procedures

General procedure A.1 for the formation of palladacarboxamide complexes

The procedure was adapted from a literature procedure.[2] In a glovebox, $PdCl_2(PPh_3)_2$ (351 mg, 0.50 mmol, 1.0 equiv.) was suspended in MeCN (13 mL) in chamber A of a two-chamber reactor. To this, the amine (2.5 mmol, 5.0 equiv.) was added, and the chamber was closed.

For SilaCOgen experiments (example for 2.0 equivalents): Chamber B, KF (58.1 mg, 1.0 mmol, 2.0 equiv.) and SilaCOgen (242 mg, 1.0 mmol, 2.0 equiv.) were added, followed by DMF (1mL), and the chamber was immediately closed. The reaction mixture was stirred at room temperature for 72 hours. The solvent was removed by filtration, and the obtained powder was washed with -30 °C Et_2O (3x4.5 mL) and -30 °C pentane (3x4.5 mL) before residual solvent was removed *in vacuo*.

For COgen experiments (example for 2.0 equivalents): Chamber B was charged with COgen (243 mg, 1.0 mmol, 2.0 equiv.), $PdCl_2$(cod) (14.3 mg, 0.050 mmol, 10 mol%) and HP(*t*-Bu)$_3$BF$_4$ (14.5 mg, 0.050 mmol, 10 mol%). The solids were dissolved in DMF (1 mL), followed by the addition of *N*-methyl-dicyclohexylamine (425 µL, 2.0 mmol, 4.0 equiv.), and the chamber was immediately closed. The reaction mixture was stirred at room temperature for 72 hours. The solvent was removed by filtration, and the obtained powder was washed with -30 °C Et_2O (3x4.5 mL) and -30 °C pentane (3x4.5 mL) before residual solvent was removed *in vacuo*.

General procedure A.2 for the formation of ^{13}C-palladacarboxamide complexes

The procedure was adapted from a literature procedure.[2] In a glovebox, $PdCl_2(PPh_3)_2$ (351 mg, 0.5 mmol, 1.0 equiv.) was suspended in MeCN (13 mL) in chamber A of a two-chamber reactor. To this, the amine (2.5 mmol, 5.0 equiv.) was added, and the chamber was closed.

For SilaCOgen experiments (example for 2 equivalents): Chamber B, KF (58.1 mg, 1.0 mmol, 2.0 equiv.) and Sila^{13}COgen (243 mg, 1 mmol, 2.0 equiv.) were added, followed by DMF (1mL), and the chamber was immediately closed. The reaction mixture was stirred at room temperature for 72 hours. The solvent was removed by filtration, and the obtained powder was washed with -30 °C Et_2O (3x4.5 mL) and -30 °C pentane (3x4.5 mL) before residual solvent was removed *in vacuo*.

For COgen experiments (example for 2 equivalents): Chamber B was charged with ^{13}COgen (243 mg, 1.0 mmol, 2.0 equiv.), $PdCl_2$(cod) (14.3 mg, 0.050 mmol, 10 mol%) and HP(*t*-Bu)$_3$BF$_4$ (14.5 mg, 0.050 mmol, 10 mol%). The solids were dissolved in DMF (1 mL), followed by the addition of *N*-methyl-dicyclohexylamine (425 µL, 2.0 mmol, 4.0 equiv.), and the chamber was immediately closed. The reaction mixture was stirred at room temperature for 72 hours. The solvent was removed by filtration, and the obtained powder was washed with -30 °C Et_2O (3x4.5 mL) and -30 °C pentane (3x4.5 mL) before residual solvent was removed *in vacuo*.

General procedure B.1 for the formation of palladacarboxamide complexes using APIs

The procedure was adapted from a literature procedure.[2] In a glovebox, $PdCl_2(PPh_3)_2$ (351 mg, 0.5 mmol, 1.0 equiv.) and the amine API (0.75 mmol, 1.5 equiv.) were suspended in

MeCN (13 mL) in chamber A of a two-chamber reactor. To this, triethylamine (105 µL, 0.75 mmol, 1.5 equiv.) was added, and the chamber was closed. To chamber B, KF (58.1 mg, 1.0 mmol, 2.0 equiv.) and SilaCOgen (242 mg, 1.0 mmol, 2.0 equiv.) were added, followed by DMF (1mL), and the chamber was immediately closed. The reaction mixture was stirred at room temperature for 72 hours. The solvent was removed by filtration, and the obtained powder was washed with -30 °C Et_2O (3 x 4.5 mL) and -30 °C pentane (3 x 4.5 mL) before residual solvent was removed *in vacuo*.

General procedure B.2 for the formation of ^{13}C-palladacarboxamide complexes using APIs

The procedure was adapted from a literature procedure.[2] In a glovebox, $PdCl_2(PPh_3)_2$ (351 mg, 0.50 mmol, 1.0 equiv.) and the amine API (0.75 mmol, 1.5 equiv.) were suspended in MeCN (13 mL) in chamber A of a two-chamber reactor. To this, triethylamine (105 µL, 0.75 mmol, 1.5 equiv.) was added, and the chamber was closed. To chamber B, KF (58.1 mg, 1.0 mmol, 2.0 equiv.) and ^{13}C-SilaCOgen (242 mg, 1.0 mmol, 2.0 equiv.) were added, followed by DMF (1mL), and the chamber was immediately closed. The reaction mixture was stirred at room temperature for 72 hours. The solvent was removed by filtration, and the obtained powder was washed with -30 °C Et_2O (3 x 4.5 mL) and -30 °C pentane (3 x 4.5 mL) before residual solvent was removed *in vacuo*.

General procedure C for the formation of ^{14}C-palladacarboxamide complexes

9-Methyl-9H-fluorene-9-^{12}C-carboxylic acid (201 mg, 0.90 mmol, 1.8 equiv.) was dissolved in toluene (8 mL), and a suspension of 9-methyl-9H-fluorene-9-^{14}C-carboxylic acid (194 MBq, 88.2 MBq/mL, 0.090 mmol, 0.18 equiv.) in toluene (2.2 mL) was added. Oxalyl dichloride (0.558 mL, 6.4 mmol, 11.8 equiv.) was added, followed by a catalytic amount of DMF. The reaction was stirred for 1 hour, until gas evolution stopped. Then the solvent and residual oxalyl dichloride were removed *in vacuo*, and the residue was dissolved in DMF (2.0 mL).

In a two-chamber reactor, $PdCl_2(PPh_3)_2$ (351 mg, 0.50 mmol, 1.0 equiv.) and the secondary amine API (0.75 mmol, 1.5 equiv.) were added to chamber A, and the chamber was sealed with a pierceable silicone/PTFE septum. To chamber B, $PdCl_2$(cod) (14.3 mg, 0.050 mmol, 0.025 equiv.), HP(*t*-Bu)$_3BF_4$ (14.5 mg, 0.050 mmol, 0.025 equiv.) were added, and the chamber was sealed with a pierceable septum. Then, the two-chamber reactor was connected to a Schlenk line via a syringe, and the atmosphere was evacuated and backfilled with nitrogen 3 times. Finally, the two-chamber reactor was evacuated and removed from the Schlenk line. To chamber A, a mixture of Et_3N (0.105 mL, 0.75 mmol, 1.5 equiv.) and MeCN (13 mL) were added. To chamber B, the COgen mixture in DMF was added (194 MBq, 194 MBq/mmol, 0.99 mmol, 1.98 equiv.), followed by *N*-methyl-dicyclohexylamine (0.428 mL, 2.00 mmol, 4.0 equiv.). Care was taken to pierce the silicone/PTFE disc in a new place to not compromise the integrity of the seal. The reaction was stirred for 72 hours. After completion, the reaction mixture of chamber A was transferred to a 15 mL Falcon tube. The tube was centrifuged, and the solvent was removed by syringe. Next, Et_2O (13 mL) was added to the solid, and the mixture was shaken to form a new suspension. The tube was centrifuged again, and the solvent was removed by syringe. Then the powder was dried under vacuum overnight. The specific activities of the products were determined by LC-MS using

Isopat to deconvolute the MS signals.[1] While the MS specific activity measurements for the Pd-complexes have been reported in this manuscript, they are likely inaccurate due to the numerous naturally occurring Pd isotopes (Pd-102 (1.0%), Pd-104 (22.3%), Pd-105 (27.3%), Pd-106 (27.3%), Pd-108 (26.5%), Pd-110 (11.7%)) and the low ^{14}C content in the molecule.[3] The specific activity has also been calculated by the gravimetric method and then converted to MBq/mmol.

General procedure D.1 for the coupling of boronic ester with palladacarboxamides

The protocol was inspired by a previously published protocol[4]. In a glovebox, arylboronic neopentyl glycol ester (0.10 mmol, 1.0 equiv.), palladacarboxamide complex (0.11 mmol, 1.10 equiv.), Na_2CO_3 (21.2 mg, 0.20 mmol, 2.0 equiv.) and KF (5.8 mg, 0.10 mmol, 1.0 equiv.) were dissolved in a mixture of 1,4-dioxane (1.5 mL) and H_2O (0.15 mL) in a 4 mL vial. The reaction was stirred at room temperature in a heat block for 18 hours. The black crude mixture was diluted with EtOAc (20 mL) and washed with water (20 mL), sat. aq. $NaHCO_3$ (20 mL) and brine (20 mL). The organic phase was dried over $MgSO_4$, and the solvent was removed *in vacuo*. Purification by flash column chromatography on silica is specified for each entry.

General procedure D.2 for the coupling of boronic esters/acids with secondary palladacarboxamides

The boronic ester/acid (0.10 mmol, 1.0 equiv.), Na_2CO_3 (0.20 mmol, 21.2 mg, 2.0 equiv.), Pd-API (0.11 mmol, 1.10 equiv.) and KF (0.10 mmol, 5.8 mg, 1.0 equiv.) were added to a 4 mL vial, followed by a mixture of 1,4-dioxane (1.5 mL) and H_2O (0.15 mL). The reaction was stirred at 60 °C in a heat block for 18 hours. The black crude mixture was directly purified, and flash column chromatography conditions are specified for each entry.

General procedure E for the coupling of boronic ester/acid with ^{14}C-palladacarboxamides drugs

The boronic ester/acid (0.10 mmol, 1.0 equiv.), Na_2CO_3 (0.20 mmol, 21.2 mg, 2.0 equiv.), **Pd-API** (0.11 mmol, 1.10 equiv.) and KF (0.10 mmol, 5.8 mg, 1.0 equiv.) were added to a 4 mL vial, followed by degassed water (0.150 mL) and degassed 1,4-dioxane (1.5 mL), and nitrogen was bubbled through the liquid phase for 60 seconds. The vial was carefully closed and sealed with parafilm. The reaction was stirred at 60 °C for 18 hours and afterwards purified by HPLC. The specific activities of the products that were determined by LC-MS used Isopat to deconvolute the MS signals.[1] While the MS specific activity measurements for the Cl-containing molecules have been reported in this manuscript, they are likely inaccurate due to the naturally occurring Cl isotopes (Cl-35 (75.8%), Cl-37 (24.2%) and the low ^{14}C content in the molecule.[3] The specific activity has also been calculated by the gravimetric method and then converted to MBq/mmol.

4 Experimental section

4.1 Starting materials

_N_-Boc-2-chloro-phenothiazine (Cl1i)

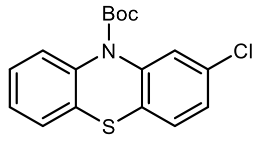

Following an adapted literature procedure[5], in a round-bottomed flask, 2-chloro-phenothiazine (2337 mg, 10 mmol, 1.0 equiv.) was mixed with 4-DMAP (490 mg, 4 mmol, 0.4 equiv.) in dried MeCN (50 mL), and Boc_2O (21.8 g, 100 mmol, 10 equiv.) was added before flushing the round-bottomed flask with argon. The reaction was stirred for 4 h at 40°C. The reaction was quenched by the addition of sat. aq. $NaHCO_3$ (50 mL), after which H_2O (50 mL) and EtOAc (50 mL) were added, followed by extraction of the aqueous phase with EtOAc (3x50 mL). The combined organic layers were dried over anhydrous $MgSO_4$, filtered, and concentrated under reduced pressure. Purification performed by flash column chromatography (0:100 to 5:95 EtOAc in heptane), afforded the product as a white solid (3070 mg, 9.2 mmol, 92%).

^{1}H-NMR (400 MHz, $CDCl_3$) δ 7.55 (d, *J* = 2.2 Hz, 1H), 7.50 (dd, *J* = 8.2, 1.0 Hz, 1H), 7.33 (dd, *J* = 7.7, 1.2 Hz, 1H), 7.29 (td, *J* = 7.6, 1.2 Hz, 1H), 7.24 (s, 1H), 7.18 (dd, *J* = 7.6, 1.0 Hz, 1H), 7.14 (dd, *J* = 8.2, 2.2 Hz, 1H), 1.50 (s, 9H). **^{13}C-NMR** (101 MHz, $CDCl_3$) δ 152.2, 139.8, 138.4, 132.4, 131.7, 130.8, 128.1, 127.6, 127.5, 127.3, 126.9, 126.4, 126.4, 82.7, 28.3. **HR-MS** (ESI+) calc. for $C_{17}H_{16}ClNO_2SNa^+$ 356.0482, found: 356.0490.

Methyl 2-((4-bromophenyl)((_tert_-butyldimethylsilyl)oxy)methyl)acrylate (Br1j)

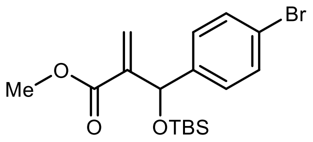

Following an adapted literature protocol[5], 4-bromobenzaldehyde (1852 mg, 10 mmol, 1.0 equiv.) and DABCO® (565 mg, 5.0 mmol, 0.5 equiv.) were added to a round-bottomed flask and dissolved in methyl acrylate (1.8 mL, 20 mmol, 2 equiv.). The reaction vessel was purged with argon, sealed with a septum, equipped with an argon balloon, and left stirring overnight at room temperature After the reaction time had elapsed, the methyl acrylate was removed under reduced pressure, and the crude was dried onto celite. Purification using flash column chromatography (0:1 to 1:3 EtOAc in heptane) afforded a white crystalline solid. The solid was dissolved in DMF (10 mL) in a round-bottomed flask, and TBSCl (3.77 g, 25 mmol, 2.5 equiv.) and imidazole (1702 mg, 25 mmol, 2.5 equiv.) were added. The reaction vessel was purged with argon, sealed with a rubber septum, and equipped with an argon balloon, and left stirring overnight. After the reaction time had elapsed, the crude was transferred into a separatory funnel, diluted with H_2O (20 mL) and DCM (20 mL). The aqueous phase was extracted with DCM (3x20 mL). The combined organic phases were dried over anhydrous $MgSO_4$, followed by filtration, concentration under reduced pressure, and dried onto celite. Purification using flash column chromatography (0:100 to 4:96 EtOAc in heptane) afforded the product as a clear oil (1949.7 mg, 4.88 mmol, 49 % over two steps).

^{1}H-NMR (400 MHz, $CDCl_3$) δ 7.41 (d, *J* = 8.4 Hz, 2H), 7.24 (d, *J* = 8.4 Hz, 2H), 6.25 (t, *J* = 1.3 Hz, 1H), 6.08 (t, *J* = 1.3 Hz, 1H), 5.54 (s, 1H), 3.67 (s, 3H), 0.86 (s, 9H), 0.04 (s, 3H), -0.11 (s, 3H). **^{13}C-NMR** (101 MHz, $CDCl_3$) δ 166.3, 143.6, 142.0, 131.3, 124.2, 121.4, 72.2, 51.9, 25.9, 18.3, -4.8, -4.9. **HR-MS** (ESI+) Calc. for $C_{17}H_{25}BrO_3SiNa^+$ 407.0649, found: 407.0637.

_N_-(4-Bromo-2,6-difluorophenyl)-3,3-dimethylbutanamide (Br1k)

Adapted from a literature protocol[6], 4-bromo-2,6-difluoroaniline (787 mg, 3.8 mmol, 1.0 equiv.) and 3,3-dimethylbutyryl chloride (1.0 mL, 7.2 mmol, 1.9 equiv.) were dissolved in DCM (20 mL), and Et_3N (0.70 mL, 5.0 mmol, 1.3 equiv.) was added. The mixture was stirred at room temperature overnight. The reaction mixture was concentrated *in vacuo,* and the product was isolated by flash column chromatography (5:95 to 50:50 EtOAc:heptane) as a white crystalline solid (961 mg, 3.1 mmol, 83%).

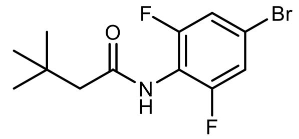

^{1}H-NMR (400 MHz, $CDCl_3$) δ 7.12 (dd, *J* = 6.7, 3.0 Hz, 2H), 6.74 (d, *J* = 21.4 Hz, 1H), 2.27 (s, 2H), 1.10 (d, *J* = 1.4 Hz, 9H). **^{13}C-NMR** (101 MHz, $CDCl_3$) δ 170.1, 157.8 (dd, *J* = 254.4, 6.1 Hz), 119.6, 115.8 (d, *J* = 27.1 Hz), 113.6, 50.4, 31.3, 29.8. **^{19}F-NMR** (376 MHz, $CDCl_3$) δ -115.40 (d, *J* = 6.8 Hz). **HR-MS** (ESI+) Calc. for $C_{12}H_{15}BrF_2NO^+$ 306.0300, found: 306.0307.

*tert-B*utyl 2-(5,5-dimethyl-1,3,2-dioxaborinan-2-yl)-10*H*-phenothiazine-10-carboxylate (B1i)

In a glovebox, *N*-Boc-2-chloro-phenothiazine (1.67 g, 5.0 mmol, 1.0 equiv.), B_2nep_2 (1.30 g, 5.8 mmol, 1.15 equiv.), XPhos Pd G4 (86 mg, 0.10 mmol, 2 mol%), XPhos (85.3 mg, 0.16 mmol, 4.0 mol%), and KOAc (1.47 g, 15.0 mmol, 3.0 equiv.) were added to a COtube® and dissolved in dioxane (12.5 mL). The COtube® was sealed and stirred overnight at 110 °C. After the reaction time had elapsed, the COtube® was cooled to room temperature, and the solution was transferred into a separatory funnel. H_2O (100 mL) and EtOAc (100 mL) were added, and the aqueous phase was extracted further with EtOAc (2x100 mL) followed by a wash of the combined organic phases with brine (75 mL). The organic phase was dried over $MgSO_4$ and concentrated under reduced pressure. Purification by flash column chromatography (0:100 to 25:75 EtOAc in heptane) to afford a white solid (1463 mg, 3.31 mmol, 66%).

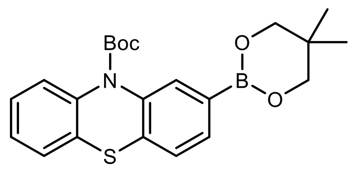

^{1}H-NMR (400 MHz, $CDCl_3$) δ 7.94 (s, 1H), 7.56 (dd, *J* = 7.8, 0.9 Hz, 1H), 7.50 (d, *J* = 7.8 Hz, 1H), 7.32 (d, *J* = 7.7 Hz, 2H), 7.25 (td, *J* = 7.7, 1.0 Hz, 1H), 7.1 (td, *J* = 7.7, 1.0 Hz, 1H), 3.76 (s, 4H), 1.48 (s, 9H), 1.01 (s, 6H). **^{13}C-NMR** (101 MHz, $CDCl_3$) δ 152.6, 138.9, 138.1, 134.9, 132.6, 132.0, 131.3, 127.5, 127.3, 126.8, 126.7, 126.1, 82.0, 72.5, 32.0, 28.3, 22.0. **HR-MS** (ESI+) calc. for $C_{22}HH_{26}NO_4BSNa^+$ 434.1568, found: 434.1570.

Methyl 2-(((*tert*-butyldimethylsilyl)oxy)(4-(5,5-dimethyl-1,3,2-dioxaborinan-2-yl)phenyl)-methyl)acrylate (B1j)

In a glovebox, Methyl 2-((4-bromophenyl)((tert-butyldimethylsilyl)oxy)methyl)acrylate (770 mg, 2.0 mmol, 1.0 equiv.), B_2nep_2 (520 mg, 2.3 mmol, 1.15 equiv.), XPhos Pd G4 (34.4 mg, 40 µmol, 2.0 mol%), XPhos (38.1 mg, 80 µmol, 4 mol%), and KOAc (589 mg, 6.0 mmol, 3.0 equiv.) were added to a COtube® and were dissolved in dioxane (5 mL). The COtube® was sealed and stirred overnight at 110 °C. After the reaction time had elapsed, the COtube® was cooled to room temperature and the solution was transferred into a separatory funnel. H_2O (40 mL) and EtOAc (40 mL) were added, and the aqueous phase was extracted further with EtOAc (2x40 mL) followed by a wash of the combined organic phases with brine (30 mL). The organic phase was dried over $MgSO_4$ and concentrated under reduced pressure. Purification using flash column chromatography (10:90 to

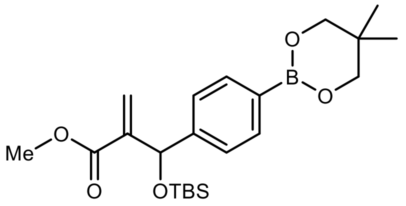

15:85 EtOAc in heptane) to afforded the product as a yellow, very viscous oil (579 mg, 1.34 mmol, 67%).

^{1}H-NMR (400 MHz, $CDCl_3$) δ 7.71 (d, *J* = 8.0 Hz, 2H), 7.33 (d, *J* = 8.0 Hz, 2H), 6.23 (t, *J* = 1.4 Hz, 1H), 6.04 (t, *J* = 1.4 Hz, 1H), 5.60 (s, 1H), 3.75 (s, 4H), 3.65 (s, 3H), 1.01 (s, 6H), 0.86 (s, 9H), 0.04 (s, 3H), -0.14 (s, 3H). **^{13}C-NMR** (101 MHz, $CDCl_3$) δ 166.5, 145.2, 144.0, 133.7, 126.5, 124.1, 72.9, 72.4, 51.8, 32.0, 25.9, 22.1, 18.3, -4.7, -4.9. **HR-MS** (ESI+) calc. for $C_{17}H_{27}O_5SiBNa^+$ 373.1613 Found: 373.1624.

3,5-difluoro-4-(3,3-dimethylbutanamido)phenylboronic acid neopentyl glycol ester (B1k)

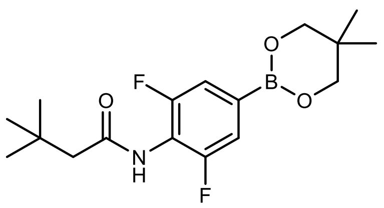

Adapted from a literature protocol[4], in a glovebox, **Br1k** (307 mg, 1.0 mmol, 1.0 equiv.), KOAc (295 mg, 3.0 mmol, 3.0 equiv.), XPhos Pd G4 (25.8 mg, 0.030 mmol, 3.0 mol%), $B_2(OH)_4$ (180 mg, 2.0 mmol, 2.0 equiv.), XPhos (28.5 mg, 0.060 mmol, 6.0 mol%) were dissolved in EtOH (5 mL). The mixture was stirred at 80 °C for 4 hours. The reaction was cooled to room temperature, and the solvent was removed *in vacuo*. The residue was dissolved in EtOAc (25 mL) and washed with saturated brine (25 mL). The organic layer was dried over $MgSO_4$ and concentrated *in vacuo*. The crude was dissolved in CH_2Cl_2 (5 mL), and neopentyl glycol (207 mg, 2.0 mmol, 2 equiv.) was added, and the vessel was flushed with argon. The mixture was stirred at room temperature overnight. The reaction mixture was diluted with EtOAc, filtered through a pad of celite, and purified with flash column chromatography (10:90 to 50:50 EtOAc in heptane) yielded the product as an off-white solid (230 mg, 0.68 mmol, 68%) containing a small, un-known impurity visible in the ^{19}F-NMR spectrum.

^{1}H-NMR (400 MHz, $CDCl_3$) δ 7.30 (d, *J* = 8.3 Hz, 2H), 6.94 (s, 1H), 3.74 (s, 4H), 2.26 (s, 2H), 1.09 (s, 9H), 1.00 (s, 6H). **^{13}C-NMR** (101 MHz, $CDCl_3$) δ 170.1, 157.3 (dd, *J* = 251.3 Hz, *J* = 4.2 Hz), 116.4, 115.9, 111.7, 72.5, 50.4, 32.0, 31.2, 29.8, 21.9. **^{19}F-NMR** (376 MHz, $CDCl_3$) δ -119.08 (d, *J* = 8.9 Hz). **HR-MS** (ESI+) Calc. for $C_{12}H_{17}BF_2NO_3^+$ 272.1264, found: 272.1268.

5-(5,5-dimethyl-1,3,2-dioxaborinan-2-yl)-2-(2,6-dioxopiperidin-3-yl)isoindoline-1,3-dione (B1q)

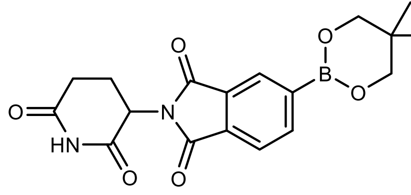

Adapted from a patent protocol, in a glovebox,[7] 5-bromo-2-(2,6-dioxopiperidin-3-yl)isoindoline-1,3-dione (2528 mg, 7.5 mmol, 1.0 equiv.), KOAc (2.21 g, 22.5 mmol, 3.0 equiv.) and Pd(dppf)Cl_2 (409 mg, 0.56 mmol, 7.5 mol%), B_2nep_2 (3.39 g, 15 mmol, 2.0 equiv.) were dissolved in 1,4-dioxane (45 mL). The mixture was stirred at 90 °C for 16 hours. The reaction was cooled to room temperature and, water (20 mL) was added to the reaction mixture, extracted with ethyl acetate (3×40 mL), the combined organic phase was washed with 20 mL of brine solution, and concentrated under reduced pressure, and purified with flash column chromatography (20:80 to 50:50 EtOAc in heptane) yielded the product as a white solid (2180 mg, 5.9 mmol, 79%).

^{1}H-NMR (400 MHz, $CDCl_3$) δ 8.31 (s, 1H), 8.18 (d, *J* = 7.3 Hz, 1H), 8.04 (s, 1H), 7.84 (d, *J* = 7.4 Hz, 1H), 4.99 (dd, *J* = 12.4, 5.2 Hz, 1H), 3.80 (d, *J* = 1.8 Hz, 5H), 2.83 (m, 4H), 2.18

(m, 1H), 1.04 (s, 6H). **^{13}C-NMR** (101 MHz, $CDCl_3$) δ 207.2, 171.0, 168.0, 167.7, 167.7, 140.2, 133.4, 130.8, 129.2, 122.8, 72.6, 49.3, 32.1, 31.5, 31.1, 22.8, 22.0 **HR-MS** (ESI+) Calc. for $C_{12}H_{17}BF_2NO_3^+$ 303.0783, found: 303.0783

4.2 Palladacarboxamide complexes

trans-Chloro(propylaminecarbonyl)bis(triphenylphosphine) palladium(II) (Pd-1)

n-Propylamine (148 mg, 2.5 mmol, 5.0 equiv.) was subjected to general procedure A.1, yielding the product as a pale-yellow powder (354 mg, 0.47 mmol, 94%).

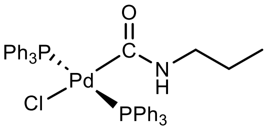

^{1}H-NMR (400 MHz, $CDCl_3$) δ 7.76 (m, 12H), 7.40 (m, 18H), 4.64 (t, *J* = 4.9 Hz, 1H), 1.95 (q, *J* = 7.3 Hz, 2H), 0.58 (sext, *J* = 7.4 Hz, 2H), 0.40 (t, *J* = 7.3 Hz, 3H). **^{13}C-NMR** (101 MHz, $CDCl_3$) δ 181.0, 135.2 (t, *J* = 6.2 Hz), 134.9 (t, *J* = 6.5 Hz), 132.3, 132.2, 132.0, 131.8, 130.7, 130.3, 128.7, 128.6, 128.3 (t, *J* = 5.1 Hz), 128.2, 128.2, 43.7, 21.9, 11.2. **^{31}P-NMR** (162 MHz, $CDCl_3$) δ 19.52. **HR-MS** (ESI+) Calc. for $C_{40}H_{38}NOP_2Pd^+$ 716.1463, found: 716.1463.

trans-Chloro(propylamine-^{13}C-carbonyl)bis(triphenylphosphine) palladium(II) (^{13}C-Pd-1)

n-Propylamine (148 mg, 2.5 mmol, 5.0 equiv.) was subjected to general procedure A.2, yielding the product as a pale-yellow powder (309 mg, 0.41 mmol, 82%).

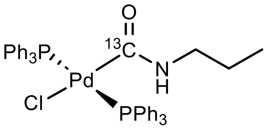

^{1}H-NMR (400 MHz, $CDCl_3$) δ 7.76 (m, 12H), 7.40 (m, 18H), 4.63 (t, *J* = 4.9 Hz, 1H), 1.93 (m, 2H), 0.58 (sext, *J* = 7.4 Hz, 2H), 0.40 (t, *J* = 7.3 Hz, 3H). **^{13}C-NMR** (101 MHz, $CDCl_3$) δ 180.9 (t, *J* = 5.7 Hz), 134.9 (t, *J* = 6.5 Hz), 132.0, 130.3, 128.3 (t, *J* = 5.1 Hz), 43.6, 21.9, 11.2. **^{31}P-NMR** (162 MHz, $CDCl_3$) δ 19.51. **HR-MS** (ESI+) Calc. for $C_{21}{}^{13}CH_{38}NOPPd^+$ 455.0586, found: 455.0587.

trans-Chloro(benzylamidecarbonyl)bis(triphenylphosphine) palladium(II) (Pd-2)

Benzylamine (268 mg, 2.5 mmol, 5.0 equiv.) was subjected to general procedure A.1, yielding the product as an off-white powder (388 mg, 0.485 mmol, 97%).

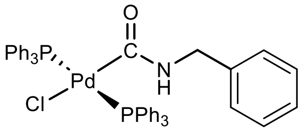

^{1}H-NMR (400 MHz, $CDCl_3$) δ 7.72 (m, 12H), 7.40 (m, 18H), 7.16 (t, *J* = 7.4 Hz, 1H), 7.09 (t, *J* = 7.3 Hz, 2H), 6.42 (d, *J* = 7.0 Hz, 2H), 4.81 (t, *J* = 5.5 Hz, 1H), 3.17 (d, *J* = 4.4 Hz, 2H). **^{13}C-NMR** (101 MHz, $CDCl_3$) δ 181.4, 137.9, 135.2 (t, *J* = 6.4 Hz), 134.9 (t, *J* = 6.4 Hz), 131.9 (t, *J* = 22.2 Hz), 130.7, 130.4, 128.4 (m), 128.2 (t, *J* = 5.6 Hz), 127.3, 46.4. **^{31}P-NMR** (162 MHz, $CDCl_3$) δ 19.84. **HR-MS** (ESI+) Calc. for $C_{26}H_{23}NOPPd^+$ 502.0552, found: 502.0556.

trans-Chloro(2,4-difluorobenzylaminecarbonyl)bis(triphenylphosphine) palladium(II) (Pd-3)

2,4-Difluorobenzylamine (358 mg, 2.5 mmol, 5.0 equiv.) was subjected to general procedure A.1, yielding the product as a white powder (414 mg, 0.495 mmol, 99%).

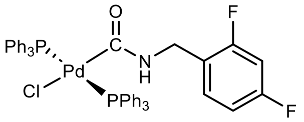

^{1}H-NMR (400 MHz, $CDCl_3$) δ 7.72 (m, 12H), 7.40(m, 18H), 6.60 (m, 1H), 6.51 (t, *J* = 8.3 Hz, 1H), 6.34 (q, *J* = 8.4 Hz, 1H), 4.96 (t, *J* = 5.5 Hz, 1H), 3.23 (d, *J* = 5.3 Hz, 2H).**^{13}C-NMR** (101 MHz, $CDCl_3$) δ 181.9, 134.7 (t, *J* = 6.5 Hz), 131.7 (t, *J* = 22.4 Hz), 130.7, 130.4, 128.4 (t, *J* = 5.1 Hz), 111.1 (dd, *J* = 21.1, 3.6 Hz), 103.6 (t, *J* = 25.5 Hz), 38.6 (d, *J* = 2.9 Hz). Due to the low intensity and splitting of the quaternary C-F carbons, they are not observed. **^{31}P-**

NMR (162 MHz, $CDCl_3$) δ 19.69. **^{19}F-NMR** (376 MHz, $CDCl_3$) δ -111.40 (p, *J* = 7.6 Hz), -114.58 (q, *J* = 8.6 Hz). **HR-MS** (ESI+) Calc. for $C_{26}H_{21}F_2NOPPd^+$ 538.0364, found: 538.0371.

***trans*-Chloro(2,4-difluorobenzylamine-^{13}C-carbonyl)bis(triphenylphosphine) palladium(II) (^{13}C-Pd-3)**

2,4-Difluorobenzylamine (358 mg, 2.5 mmol, 5.0 equiv.) was subjected to general procedure A.2, yielding the product as a pale-yellow powder (415 mg, 0.495 mmol, 99%).

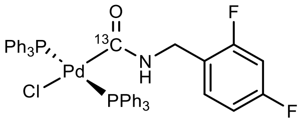

^{1}H-NMR (400 MHz, $CDCl_3$) δ 7.70 (m, 12H), 7.40 (m, 18H), 6.60 (m, 1H), 6.51 (td, *J* = 8.1, 2.3 Hz, 1H), 6.34 (q, *J* = 8.5 Hz, 1H), 4.96 (q, *J* = 5.2 Hz, 1H), 3.23 (t, *J* = 3.9 Hz, 2H). **^{13}C-NMR** (101 MHz, $CDCl_3$) δ 181.9 (t, *J* = 5.5 Hz), 134.7 (t, *J* = 6.5 Hz), 131.7 (t, *J* = 22.4 Hz), 130.4, 128.4 (t, *J* = 5.2 Hz), 111.1 (dd, *J* = 20.7, 3.5 Hz), 103.6, 38.6. **^{31}P-NMR** (162 MHz, $CDCl_3$) δ 19.70. Due to the low intensity and splitting of the quaternary C-F carbons they are not observed. **^{19}F-NMR** (376 MHz, $CDCl_3$) δ -111.39 (p, *J* = 7.9 Hz), -114.58 (q, *J* = 8.6 Hz). **HR-MS** (ESI+) Calc. for $C_{25}{}^{13}CH_{21}F_2NOPPd^+$ 539.0397, found: 539.0404.

***trans*-Chloro(melthylaminecarbonyl)bis(triphenylphosphine) palladium(II) (Pd-4)**

Methylamine (9v/v% in MeCN, 0.959 ml, 2.5 mmol, 5.0 equiv.) was subjected to general procedure A.1, with the exception that the reaction time was reduced to 2 hours, yielding the product as an off-white powder (287 mg, 0.396 mmol, 79%).

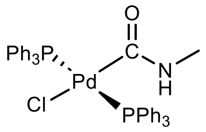

^{1}H-NMR (400 MHz, $CDCl_3$) δ 7.77 (m, 12H), 7.40 (m, 18H), 4.67 (d, *J* = 5.3 Hz, 1H), 1.58 (d, *J* = 4.8 Hz, 3H). **^{13}C-NMR** (101 MHz, $CDCl_3$) δ 182.0, 134.9 (t, *J* = 6.5 Hz), 131.9 (t, *J* = 22.2 Hz), 130.4, 128.3 (t, *J* = 5.1 Hz), 27.5. **^{31}P-NMR** (162 MHz, $CDCl_3$) δ 19.57. **HR-MS** (ESI+) Calc. for $C_{20}H_{34}NOP_2Pd^+$ 688.1145, found: 688.1158.

***trans*-Chloro(melthylamine-^{13}C-carbonyl)bis(triphenylphosphine) palladium(II) (^{13}C-Pd-4)**

Methylamine (9v/v% in MeCN, 0.959 ml, 2.5 mmol, 5.0 equiv.) was subjected to general procedure A.2, with the exception that the reaction time was reduced to 2 hours, yielding the product as an off-white powder (290 mg, 0.400 mmol, 80%).

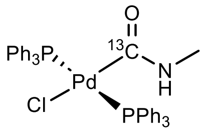

^{1}H-NMR (400 MHz, $CDCl_3$) δ 7.76 (m, 12H), 7.39 (m, 18H), 4.67 (s, 1H), 1.58 (s, 3H). **^{13}C-NMR** (101 MHz, $CDCl_3$) δ 183.0 (m), 134.9 (d, *J* = 7.2 Hz), 131.2, 130.4, 128.3. **^{31}P-NMR** (162 MHz, $CDCl_3$) δ 19.56. **HR-MS** (ESI+) Calc. for $C_{19}{}^{13}CH_{34}NOP_2Pd^+$ 689.1178, found: 689.1213.

***trans*-Chloro(piperidinecarbonyl)bis(triphenylphosphine) palladium(II) (Pd-5)**

Piperidine (213 mg, 2.5 mmol, 5.0 equiv.) was subjected to general procedure A.1, yielding the product as a white powder (370 mg, 0.475 mmol, 95%).

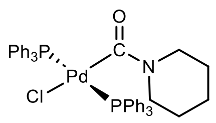

^{1}H-NMR (400 MHz, $CDCl_3$) δ 7.72 (m, 12H), 7.38 (m, 18H), 3.65 (t, *J* = 5.5 Hz, 1H), 2.34 (t, *J* = 5.7 Hz, 1H), 1.04 (q, *J* = 5.9 Hz, 1H), 0.93 (p, *J* = 5.9 Hz, 1H), 0.51 (p, *J* = 5.8 Hz, 1H). **^{13}C-NMR** (101 MHz, $CDCl_3$) δ 181.3 (d, *J* = 9.0 Hz), 135.0 (t, *J* = 6.5 Hz), 131.8 (t, *J* = 22.0

Hz), 130.3, 128.3 (t, *J* = 5.1 Hz), 47.5, 44.0, 25.3, 24.7, 24.4. **^{31}P-NMR** (162 MHz, $CDCl_3$) δ 20.14. **HR-MS** (ESI+) Calc. for $C_{42}H_{40}NOP_2Pd^+$ 742.1614, found: 742.1678.

***trans*-Chloro(piperidine-^{13}C-carbonyl)bis(triphenylphosphine) palladium(II) (^{13}C-Pd-5)**

Piperidine (213 mg, 2.5 mmol, 5.0 equiv.) was subjected to general procedure A.2, yielding the product as a white powder (383 mg, 0.492 mmol, 98%).

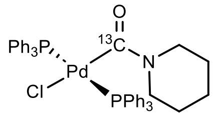

^{1}H-NMR (400 MHz, $CDCl_3$) δ 7.74 (q, *J* = 5.7 Hz, 12H), 7.40 (m, 19H), 3.65 (p, *J* = 3.7 Hz, 2H), 2.30 (m, 2H), 1.04 (q, *J* = 6.0 Hz, 2H), 0.93 (q, *J* = 5.7 Hz, 2H), 0.51 (p, *J* = 5.7 Hz, 2H). **^{13}C-NMR** (101 MHz, $CDCl_3$) δ 181.3 (t, *J* = 9.0 Hz), 135.0 (t, *J* = 6.5 Hz), 131.8 (t, *J* = 22.0 Hz), 130.3, 128.3 (t, *J* = 5.1 Hz), 44.3 (d, *J* = 75.5 Hz), 24.5 (d, *J* = 36.8 Hz), 22.7.**^{31}P-NMR** (162 MHz, $CDCl_3$) δ 20.14. **HR-MS** (ESI+) Calc. for $C_{42}H_{40}NOP_2Pd^+$ 743.1648, found: 743.1693.

***trans*-Chloro(morpholinecarbonyl)bis(triphenylphosphine) palladium(II) (Pd-6)**

Morpholine (218 mg, 2.5 mmol, 5.0 equiv.) was subjected to general procedure A.1, yielding the product as a white powder (380 mg, 0.487 mmol, 97%).

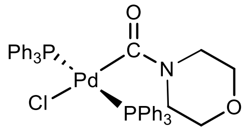

^{1}H-NMR (400 MHz, $CDCl_3$) δ 7.73 (m, 12H), 7.41 (m, 18H), 3.64 (t, *J* = 4.8 Hz, 2H), 2.95 (t, *J* = 4.8 Hz, 2H), 2.60 (t, *J* = 4.9 Hz, 2H), 2.41 (t, *J* = 4.9 Hz, 2H). **^{13}C-NMR** (101 MHz, $CDCl_3$) δ 183.3 (t, *J* = 8.1 Hz), 135.0 (t, *J* = 6.5 Hz), 131.5 (t, *J* = 22.4 Hz), 130.6, 128.5 (t, *J* = 5.1 Hz), 77.4. **^{31}P-NMR** (162 MHz, $CDCl_3$) δ 20.32. **HR-MS** (ESI+) Calc. for $C_{41}H_{39}NO_2P_2Pd^+$ 744.1407, found: 744.1429.

***trans*-Chloro(cyclopropyl(4l^2-piperazin-1-yl)methanonecarbonyl)bis(triphenylphosphine) palladium(II) (Pd-7)**

Cyclopropyl-1-piperazinylmethanone (386 mg, 2.5 mmol, 5.0 equiv.) was subjected to general procedure A.1, yielding the product as a white powder (331 mg, 0.403 mmol, 81%).

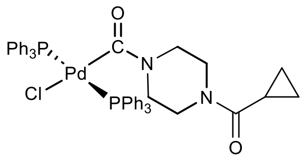

^{1}H-NMR (400 MHz, $CDCl_3$) δ 7.70 (m, 12H), 7.40 (m, 18H), 3.70 (m, 2H), 2.95 (t, *J*=5.1, 2H), 2.57 (m, 2H), 2.42 (m, 2H), 1.45 (m, 1H), 0.85 (m, 2H), 0.66 (m, 2H). **^{13}C-NMR** (101 MHz, $CDCl_3$) δ 135.0 (t, *J*=6.5), 131.4 (t, *J*=22.7), 130.6 (d, *J*=7.2), 128.5 (t, *J*=5.1), 46.0, 44.38, 42.8 (m), 41.6, 11.0, 7.5. **^{31}P{^{1}H}-NMR** (162 MHz, $CDCl_3$) δ 20.27 (d, *J*=12.7). **HR-MS** (ESI+) calc. for $C_{44}{}^{12}CH_{43}N_2O_2P_2Pd^+$ 811.1829, found: 811.1837.

***trans*-Chloro(cyclopropyl(4l^2-piperazin-1-yl)methanone-^{13}C-carbonyl)bis(triphenylphosphine) palladium(II) (^{13}C-Pd-7)**

Cyclopropyl-1-piperazinylmethanone (386 mg, 2.5 mmol, 5.0 equiv.) was subjected to general procedure A.2, yielding the product as a white powder (266 mg, 0.324 mmol, 65%).

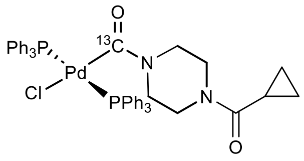

^{1}H-NMR (400 MHz, $CDCl_3$) δ 7.72 (m, 12H), 7.40 (m, 18H), 3.70 (m, 2H), 2.94 (m, 2H), 2.58 (m, 2H), 2.40 (m, 2H), 1.45 (m, 1H), 0.84 (m, 2H), 0.66 (m, 2H). **^{13}C-NMR** (101 MHz, $CDCl_3$) δ 184.2 (t, *J*=7.8), 183.3 (t, *J*=7.3), 135.0 (t, *J*=6.4), 131.4 (t, *J*=22.5), 130.6 (m), 128.5 (t, *J*=5.1), 46.0, 44.4, 42.9 (d, *J*=11.9), 41.6, 11.0, 7.4 (m). **^{31}P{^{1}H}-**

NMR (162 MHz, $CDCl_3$) δ 20.26 (d, *J*=7.8). **HR-MS** (ESI+) calc. for $C_{44}{}^{13}CH_{43}N_2O_2P_2Pd^+$ 812.1863, found: 812.1878.

trans-Chloro(prop-2-yn-1-ylaminecarbonyl)bis(triphenylphosphine) palladium(II) (Pd-8)

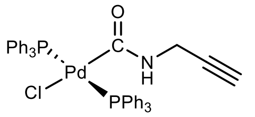

Freshly distilled propargylamine (0.159 mL, 2.5 mmol, 5.0 equiv.) was subjected to general procedure A.1, yielding the product as a white powder (410 mg, 0.500 mmol, >99%).

^{1}H-NMR (400 MHz, $CDCl_3$) δ 7.76 (m, 12H), 7.40 (m, 18H), 4.83 (t, *J* = 4.2 Hz, 1H), 2.68 (dd, *J* = 4.4, 2.7 Hz, 2H), 1.98 (t, *J* = 2.6 Hz, 1H). **^{13}C-NMR** (101 MHz, $CDCl_3$) δ 182.2, 135.2, 135.2, 135.1, 134.9, 134.8, 134.8, 131.8, 131.6, 131.4, 130.7, 130.5, 128.4, 128.4, 128.3, 128.3, 128.2, 128.1, 116.5, 80.0, 72.0, 30.3, 2.0. **^{31}P-NMR** (162 MHz, $CDCl_3$) δ 19.3. **HR-MS** (ESI+) calc. for $C_{40}H_{34}NOP_2Pd^+$ 712.1145, found: 712.1196.

trans-Chloro(2-chloro-11-(piperazin-1-yl)dibenzo[*b,f*][1,4]oxazepine)carbonyl)bis(triphenylphosphine) palladium(II) (Pd-10)

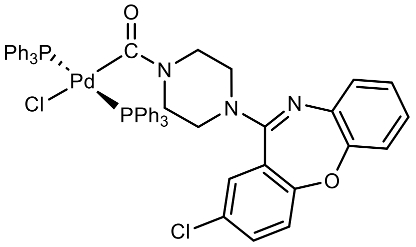

Amoxapine (235 mg, 0.75 mmol, 1.5 equiv.) was subjected to general procedure B.1, yielding the product as a white powder (419 mg, 0.415 mmol, 83%).

^{1}H-NMR (400 MHz, $CDCl_3$) δ 7.72 (m, 12H), 7.40 (m, 18H), 7.33 (m, 1H), 7.14 (d, *J*=8.7, 1H), 7.06 (m, 3H), 6.97 (m, 1H), 6.92 (m, 1H), 3.27 – 4.33 (m, 2H), 3.00 (m, 2H), 2.08 – 2.73 (m, 4H). **^{13}C-NMR** (101 MHz, $CDCl_3$) δ 184.2 (t, *J*=7.6), 159.2, 158.3, 151.8, 140.1, 135.04(t, *J*=6.5), 132.7, 131.5 (t, *J*=22.4), 130.6, 130.3, 128.9, 128.5 (t, *J*=5.2), 127.1, 125.9, 125.0, 124.7, 122.8, 120.2, 45.6, 42.7, 41.0 (d, *J*=10.2), 39.4. **^{31}P-NMR** (162 MHz, $CDCl_3$) δ 20.24. **HR-MS** (ESI+) calc. for $C_{53}{}^{12}CH_{45}N_3O_2P_2Pd+$ 970.1705, found: 970.1720.

trans-Chloro(2-chloro-11-(piperazin-1-yl)dibenzo[*b,f*][1,4]oxazepine)-^{13}C-carbonyl)bis(triphenylphosphine) palladium(II) (^{13}C-Pd-10)

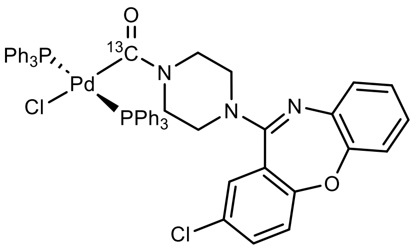

Amoxapine (235 mg, 0.75 mmol, 1.5 equiv.) was subjected to general procedure B.2, yielding the product as a white powder (418 mg, 0.415 mmol, 83%).

^{1}H-NMR (400 MHz, $CDCl_3$) δ 7.77 (m, 12H), 7.40 (m, 18H), 7.34 (d, *J*=2.5, 1H), 7.14 (d, *J*=8.7, 1H), 7.07 (m, 3H), 6.98 (m, 1H), 6.92 (d, *J*=2.6, 1H), 3.23 – 4.39 (m, 2H), 2.93 (m, 2H), 2.23 – 2.69 (m, 4H). **^{13}C-NMR** (101 MHz, $CDCl_3$) δ 184.2 (t, *J*=7.6), 159.2, 158.3, 151.8, 140.1, 135.0 (t, *J*=6.5), 132.7, 131.5 (t, *J*=22.4), 130.6, 130.3, 128.9, 128.5 (t, *J*=5.2), 127.1, 125.9, 125.0, 124.7, 122.8, 120.2, 45.6, 42.7, 41.0 (d, *J*=10.2), 39.4. **^{31}P-NMR** (162 MHz, $CDCl_3$) δ 20.23 (d, *J*=7.8). **HR-MS** (ESI+) calc. for $C_{53}{}^{13}CH_{45}N_3O_2P_2Pd$ 971.1738, found: 971.1760.

trans-Chloro(2-chloro-11-(piperazin-1-yl)dibenzo[_b,f_][1,4]oxazepine)-^{14}C-carbonyl)bis-(triphenylphosphine) palladium(II) (^{14}C-Pd-10)

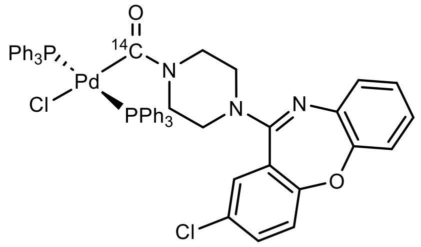

Amoxapine (235 mg, 0.75 mmol, 1.5 equiv.) was subjected to general procedure C, yielding the product as a white powder (83.6 MBq, SA: 194 MBq/mmol or 599 MBq/mmol by MS, 87%, 43% RCY).

^{1}H-NMR (400 MHz, $CDCl_3$) δ 7.79 (m, 12H), 7.44 (m, 18H), 7.38 (dd, *J* = 8.7, 2.6 Hz, 1H), 7.16 (d, *J* = 8.6 Hz, 1H), 7.09 (m, 3H), 7.00 (m, 1H), 6.95 (d, *J* = 2.6 Hz, 1H), 3.03 – 2.36 (m, 8H). **^{13}C-NMR** (101 MHz, $CDCl_3$) δ 184.2, 159.2, 158.3, 151.8, 140.1, 135.0 (t, *J* = 6.4 Hz), 131.5 (t, *J* = 22.3 Hz), 130.6, 130.3, 128.9, 128.5 (t, *J* = 5.1 Hz), 127.1, 125.9, 125.0, 124.7, 122.8, 120.2, 116.5, 45.9, 42.7. **^{31}P-NMR** (162 MHz, $CDCl_3$) δ 20.23 (d, *J*=7.8). **LR-LCMS** (ESI+) calc. for $C_{53}{}^{14}CH_{45}N_3O_2P_2Pd$ 972.1737, found: 971.2.

trans-Chloro(1-cyclopropyl-6-fluoro-4-oxo-7-(piperidin-4-yl)-1,4-dihydroquinoline-3-carboxylic acid)carbonyl)bis(triphenylphosphine) palladium(II) (Pd-11)

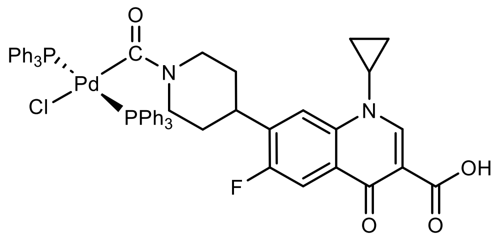

Ciprofloxacin (248 mg, 0.75 mmol, 1.5 equiv.) was subjected to general procedure B.1, yielding the product as a white powder (505 mg, 0.369 mmol, 74%).

^{1}H-NMR (400 MHz, $CDCl_3$) δ 14.89 (br s, 1H), 8.75 (m, 1H), 7.95 (d, *J* = 12.9 Hz, 1H), 7.74 (m, 17H), 7.41 (m, 26H), 6.94 (d, *J*=6.9, 1H), 3.91 (s, 1H), 2.88 – 3.77 (m, 3H), 2.64 (s, 3H), 2.21 (s, 2H), 1.36 (m, 2H), 1.16 (s, 2H). **^{13}C-NMR** (101 MHz, $CDCl_3$) *Spectrum attached, but not reported due to low intensity.* **^{31}P-NMR** (162 MHz, $CDCl_3$) δ 20.37. **^{19}F-NMR** (376 MHz, $CDCl_3$) δ -120.80, -120.86. **HR-MS** (ESI+) calc. for $C_{53}{}^{12}CH_{47}FN_3O_4P_2Pd+$ 988.2055, found: 988.2097.

trans-Chloro(1-cyclopropyl-6-fluoro-4-oxo-7-(piperidin-4-yl)-1,4-dihydroquinoline-3-carboxylic acid)-^{13}C-carbonyl)bis(triphenylphosphine) palladium(II) (^{13}C-Pd-11)

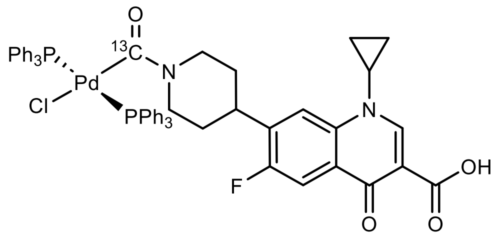

Ciprofloxacin (248 mg, 0.75 mmol, 1.5 equiv.) was subjected to general procedure B.2, yielding the product as a pale yellow powder (559 mg, 0.300 mmol, 82%).

^{1}H-NMR (400 MHz, $CDCl_3$) δ 14.97 (br s, 1H), 8.72 (m, 1H), 8.00 (m, 1H), 7.72 (m, 12H), 7.41 (m, 18H), 6.94 (d, *J*=6.8, 1H), 3.91 (s, 1H), 2.98 – 3.77 (m, 4H), 2.64 (s, 2H), 2.21 (s, 2H), 1.35 (d, *J*=6.9, 2H), 1.17 (s, 2H). **^{13}C-NMR** (101 MHz, $CDCl_3$) *Spectrum attached, but not reported due to low intensity.* **^{31}P-NMR** (162 MHz, $CDCl_3$) δ 20.38 (d, *J*=7.2). **^{19}F-NMR** (376 MHz, $CDCl_3$) δ -120.76 (m), -120.88 (m). **HR-MS** (ESI+) calc. for $C_{53}{}^{13}CH_{47}FN_3O_4P_2Pd+$ 989.2089, found: 989.2123.

***trans*-Chloro((3S,4R)-3-((benzo[*d*][1,3]dioxol-5-yloxy)methyl)-4-(4-fluorophenyl)piperidine)carbonyl)bis(triphenylphosphine) palladium(II) (Pd-12)**

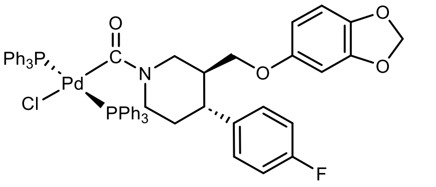

Paroxetine-Acetic acid salt (247 mg, 0.635 mmol, 1.27 equiv.) was subjected to general procedure B.1. In contrast to the other palladium complexes, this palladium complex dissolved in acetonitrile, leading to a changed work-up procedure. After the reaction, the solvent was removed *in vacuo*. Et_2O and pentane were added, the suspension was mixed, and the solvent was removed *in vacuo* again. This step was repeated one additional time, after which a solid formed upon complete dryness. The solid was washed four times with Et_2O, and four times with pentane using centrifugation, yielded the product as a white powder (380 mg, 0.371 mmol, 74%). Analysis by ^{19}F-NMR shows the presence of the starting material.

^{1}H-NMR (400 MHz, $CDCl_3$) *Spectrum attached but not reported due to a mixture of starting material and product.* **^{13}C-NMR** (101 MHz, $CDCl_3$) *Spectrum attached but not reported due to a mixture of starting material and product.* **^{31}P-NMR** (162 MHz, $CDCl_3$) δ 20.8 (d, *J*=20.2), 20.1 (d, *J*=17.6). **^{19}F-NMR** (376 MHz, $CDCl_3$) δ -116.12, -116.27. **HR-MS** (ESI+) calc. for $C_{55}{}^{12}CH_{49}FNO_4P_2Pd+$ 986.2150, found: 986.2196.

***trans*-Chloro((3S,4R)-3-((benzo[*d*][1,3]dioxol-5-yloxy)methyl)-4-(4-fluorophenyl)piperidine)-^{13}C-carbonyl)bis(triphenylphosphine) palladium(II) (^{13}C-Pd-12)**

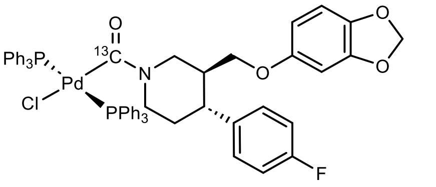

Paroxetine-Acetic acid salt (247 mg, 0.635 mmol, 1.27 equiv.) was subjected to general procedure B.2. In contrast to the other palladium complexes, this palladium complex dissolved in acetonitrile, leading to a changed work-up procedure. After the reaction, the solvent was removed *in vacuo*. Et_2O and pentane were added, the suspension was mixed, and the solvent was removed *in vacuo* again. This step was repeated one additional time, after which a solid formed upon complete dryness. The solid was washed four times with Et_2O, and four times with pentane using centrifugation, yielding the product as a white powder (312.5 mg, 0.305 mmol, 61% corrected). Analysis by ^{19}F-NMR shows the presence of the starting material.

^{1}H-NMR (400 MHz, $CDCl_3$) *Spectrum attached but not reported due to a mixture of starting material and product.* **^{13}C-NMR** (101 MHz, $CDCl_3$) δ 182.8 (t, *J*=8.7), 182.4 (t, *J*=8.6), *Spectrum attached, but only the ^{13}C-enriched signals reported due to a mixture of starting material and product.* **^{31}P-NMR** (162 MHz, $CDCl_3$) δ 20.81 (dd, *J*=19.8, 8.7), 20.12 (dd, *J*=17.7, 8.7). **^{19}F-NMR** (376 MHz, $CDCl_3$) δ -116.13, -116.27. **HR-MS** (ESI+) calc. for $C_{55}{}^{13}CH_{49}FNO_4P_2Pd+$ 987.2184, found: 987.2226.

trans-Chloro(4-(4-(4-fluorophenyl)-1-(piperidin-4-yl)-1H-imidazol-5-yl)-2-methoxy-pyrimidine)carbonyl)bis(triphenylphosphine) palladium(II) (Pd-13)

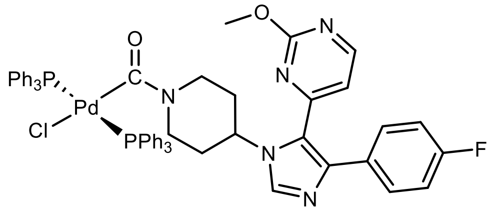

SB-242235 (265 mg, 0.75 mmol, 1.5 equiv.) was subjected to general procedure B.1, yielding the product as a white powder (419 mg, 0.400 mmol, 80%).

^{1}H-NMR (400 MHz, $CDCl_3$) δ 8.31 (d, *J*=5.1, 1H), 7.72 (m, 12H), 7.41 (m, 20H), 7.19 (s, 1H), 7.00 (t, *J*=8.6, 2H), 6.71 (d, *J*=5.1, 1H), 5.40 (d, *J*=12.9, 1H), 4.37 (t, *J*=12.3, 1H), 3.96 (s, 3H), 3.80 (d, *J*=13.5, 1H), 2.12 (t, *J*=12.6, 1H), 1.82 (d, *J*=11.8, 1H), 1.47 (d, *J*=11.2, 1H), 1.30 (m, 1H), 0.75 (qd, *J*=12.3, 4.3, 1H), 0.50 (qd, *J*=11.9, 3.6, 1H). **^{13}C-NMR** (101 MHz, $CDCl_3$) δ 183.1, 165.6, 159.8, 159.4, 143.4, 136.6, 135.1 (m), 131.5 (m), 130.6 (d, *J*=5.8), 130.4 (d, *J*=8.3), 128.6 (dd, *J*=6.5, 3.7), 128.4 (dd, *J*=6.6, 3.6), 123.7, 115.8, 115.6, 55.0, 54.1, 45.7, 41.8, 33.2, 32.0. The doublet peak corresponding to the C-F carbon is not observed. **^{31}P-NMR** (162 MHz, $CDCl_3$) δ 20.31 (d, *J*=17.8). **^{19}F-NMR** (376 MHz, $CDCl_3$) δ -113.74. **HR-MS** (ESI+) calc. for $C_{55}{}^{12}CH_{49}FN_5O_2P_2Pd^+$ 1010.2375, found: 1010.2395.

trans-Chloro(4-(4-(4-fluorophenyl)-1-(piperidin-4-yl)-1H-imidazol-5-yl)-2-methoxy-pyrimidine)-^{13}C-carbonyl)bis(triphenylphosphine) palladium(II) (^{13}C-Pd-13)

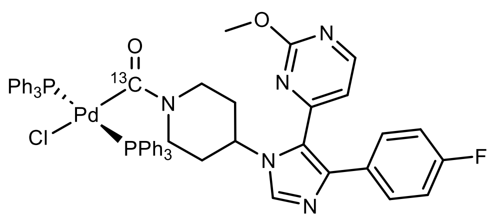

SB-242235 (265 mg, 0.75 mmol, 1.5 equiv.) was subjected to general procedure B.2, yielding the product as a white powder (475 mg, 0.454 mmol, 91%).

^{1}H-NMR (400 MHz, $CDCl_3$) δ 8.31 (d, *J*=5.2, 1H), 7.72 (m, 12H), 7.40 (m, 20H), 7.19 (s, 1H), 7.00 (t, *J*=8.7, 2H), 6.71 (d, *J*=5.2, 1H), 5.40 (d, *J*=13.1, 1H), 4.37 (m, 1H), 3.96 (s, 3H), 3.80 (d, *J*=13.5, 1H), 2.12 (t, *J*=12.6, 1H), 1.82 (d, *J*=12.1, 1H), 1.47 (d, *J*=12.2, 1H), 1.31 (t, *J*=12.9, 1H), 0.75 (qd, *J*=12.2, 4.1, 1H), 0.50 (qd, *J*=12.0, 3.9, 1H). **^{13}C-NMR** (101 MHz, $CDCl_3$) δ 183.1 (t, *J*=8.1), 165.6, 162.8 (d, *J*=247.8 Hz) 159.8, 159.4, 143.4, 136.6, 135.1 (m), 131.5 (m), 130.6 (d, *J*=6.0), 130.4 (d, *J*=8.1), 128.6 (dd, *J*=6.7, 3.5), 128.4 (dd, *J*=6.6, 3.7), 123.7, 115.8, 115.6, 55.0, 54.1, 45.8, 41.8, 33.2, 32.0. **^{31}P-NMR** (162 MHz, $CDCl_3$) δ 20.31 (dd, *J*=17.8, 8.0). **^{19}F-NMR** (376 MHz, $CDCl_3$) δ -113.74. **HR-MS** (ESI+) calc. for $C_{55}{}^{13}CH_{49}FN_5O_2P_2Pd^+$ 1011.2408, found: 1011.2434.

trans-Chloro(4-(4-(4-fluorophenyl)-1-(piperidin-4-yl)-1H-imidazol-5-yl)-2-methoxypyrimidine)-^{14}C-carbonyl)bis(triphenylphosphine) palladium(II) (^{14}C-Pd-13)

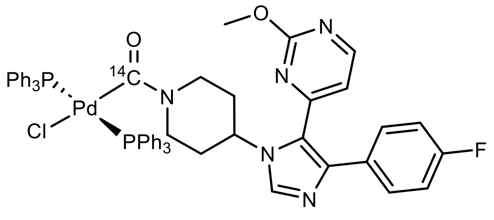

SB-242235 (265 mg, 0.75 mmol, 1.5 equiv.) was subjected to general procedure C, yielding the product (86% purity by ^{31}P-qNMR) as a white powder (83.0 MBq, SA: 190 MBq/mmol or 346 MBq/mmol by MS, 90%, 43% RCY).

^{1}H-NMR (400 MHz, $CDCl_3$) δ 8.31 (d, *J* = 5.1 Hz, 1H), 7.74 (m, 13H), 7.39 (m, 19H), 7.19 (s, 1H), 7.01 (m, 2H), 6.70 (d, *J* = 5.2 Hz, 1H), 5.40 (d, *J* = 12.9 Hz, 1H), 4.37 (ddd, *J* = 12.1, 8.6, 3.6 Hz, 1H), 3.96 (s, 3H), 3.80 (d, *J* = 13.8 Hz, 1H), 2.12 (m, 1H), 2.00 (s, 3H), 1.82 (d, *J* = 12.0 Hz, 1H), 1.32 (t, *J* = 12.8 Hz, 1H), 0.76 (qd, *J* = 12.2, 4.3 Hz, 1H), 0.51 (qd, *J* = 12.2, 4.5 Hz, 1H). **^{13}C-NMR** (101 MHz, $CDCl_3$) δ 183.1, 165.6, 159.8, 159.4, 143.4, 136.6,

135.2, 135.0, 131.5, 130.6 (d, *J*=6.0), 130.4 (d, *J*=8.1), 128.6 (dd, *J*=6.7, 3.5), 128.4 (dd, *J*=6.6, 3.7), 123.7, 115.8, 115.6, 55.0, 54.1, 45.8, 41.8, 33.2, 32.0. The doublet peak corresponding to the C-F carbon is not observed. **^{31}P-NMR** (162 MHz, $CDCl_3$) δ 20.31 (dd, *J*=17.8, 8.0). (Some peaks have very low intensity) **^{19}F-NMR** (376 MHz, $CDCl_3$) δ -113.74. **LR-LCMS** (ESI+) calc. for $C_{37}{}^{14}CH_{34}FN_5O_2PPd^+$ 750.1496, found: 750.2.

4.3 Additional Pd-complexes

Below are examples of Pd-complexes not included in the manuscript, due to either no or low conversion.

***trans*-Chloro(2-morpholinoethan-1-amine-carbonyl)bis(triphenylphosphine) palladium(II) (Pd-9)**

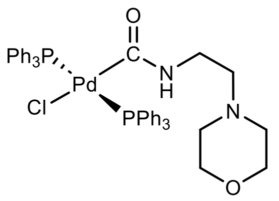

2-Morpholinoethan-1-amine (97.5 mg, 0.75 mmol, 1.5 equiv.) was subjected to general procedure A.1, a yellow powder was obtained, the desired product was not isolated, but observed by PNMR. The purity was estimated to 50%, leading to a yield of 42%. The ^{1}H-NMR, ^{13}C-NMR, and ^{31}P-NMR are attached.

^{1}H-NMR (400 MHz, $CDCl_3$) Spectrum attached, the compound rapidly degrades in solvents. **^{13}C-NMR** (101 MHz, $CDCl_3$) Spectrum attached, the compound rapidly degrades in solvents. **^{31}P-NMR** (162 MHz, $CDCl_3$) δ 19.56. **HR-MS** (ESI+) calc. for $C_{25}H_{28}N_2O_2PPd^+$ 526.0951, found: 526.0961.

***trans*-Chloro(3,6,9,12-tetraoxapentadec-14-yn-1-amine-carbonyl)bis(triphenylphosphine) palladium(II) (Pd-14)**

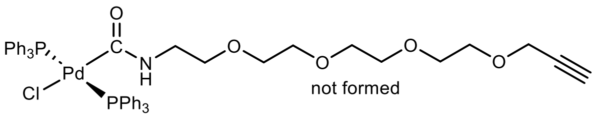

3,6,9,12-Tetraoxapentadec-14-yn-1-amine (34.7 mg, 0.15 mmol, 1.5 equiv.) was subjected to general procedure B.1 at a 0.10 mmol scale, a yellow powder was obtained, and the desired product was not observed by PNMR.

***trans*-Chloro((4-Ethynylphenyl)methanamine-carbonyl)bis(triphenylphosphine) palladium(II) (Pd-15)**

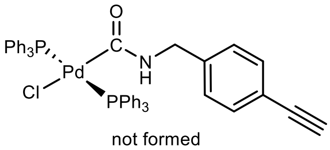

(4-Ethynylphenyl)methanamine (34.7 mg, 0.15 mmol, 1.5 equiv.) was subjected to general procedure B.1, at a 0.10 mmol scale, only the starting material was recovered.

***trans*-Chloro(1-ethynylpiperazine-carbonyl)bis(triphenylphosphine) palladium(II) (Pd-16)**

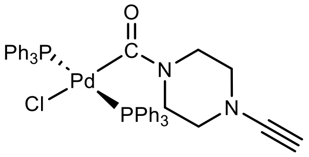

1-ethynylpiperazine (16.5 mg, 0.15 mmol, 1.5 equiv.) was subjected to general procedure B.1, at a 0.10 mmol scale, full conversion was observed, and the product was isolated as a white powder (35.8 mg, 91%). The ^{1}H-NMR and ^{31}P-NMR are attached.

***trans*-Chloro(8-fluoro-5-(4-((methylamino)methyl)phenyl)-2,3,4,6-tetrahydro-1*H*-azepino[5,4,3-*cd*]indol-1-one -carbonyl)bis(triphenylphosphine) palladium(II) (Pd-17)**

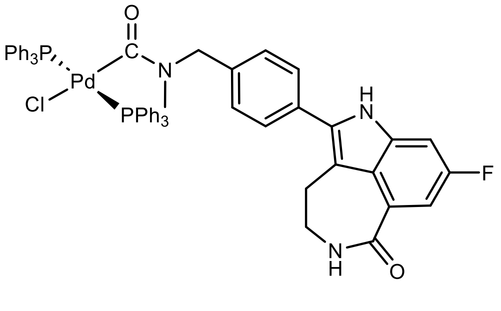

8-fluoro-5-(4-((methylamino)methyl)phenyl)-2,3,4,6-tetrahydro-1*H*-azepino[5,4,3-*cd*]indol-1-one (48.5 mg, 0.15 mmol, 1.5 equiv.) was subjected to general procedure B.1, at a 0.10 mmol scale, the desired product was observed with a conversion to the desired product based on ^{31}P-qNMR to be 12%. The ^{1}H-NMR and ^{31}P-NMR are attached.

trans-Chloro(1-ethynylpiperazine-carbonyl)bis(triphenylphosphine) palladium(II) (Pd-18)

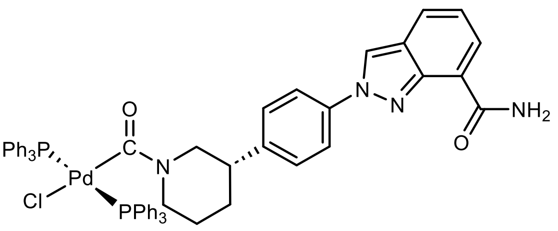

Niraparib-*para*-methylbenzenesulfonate salt (73.9 mg, 0.15 mmol, 1.5 equiv.) was subjected to general procedure B.1, at a 0.10 mmol scale, the desired product was observed with a conversion to the desired product based on ^{31}P-qNMR to be 9%. The ^{1}H-NMR and ^{31}P-NMR are attached.

trans-Chloro(4-aniline-carbonyl)bis(triphenylphosphine) palladium(II) (Pd-19)

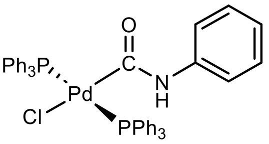

Initial conditions:

Aniline (37.3 mg, 0.40 mmol, 4.0 equiv.) and sodium hydride (60 wt% dispersion in mineral oil, 4.8 mg, 0.12 mmol, 1.2 equiv.) were subjected to general procedure B.1, at a 0.10 mmol scale. A yellow solid was obtained, and the product was identified by ^{31}P-NMR, and q-^{31}P-NMR indicated a >5% yield.

Example $PdI_2(PPh_3)_2$ conditions:

Aniline (37.3 mg, 0.40 mmol, 4.0 equiv.) and potassium hydride (30 wt% dispersion in mineral oil, 4.8 mg, 0.12 mmol, 1.2 equiv.) were subjected to a modified general procedure B.1 with $PdI_2(PPh_3)_2$ (89.7 mg, 0.10 mmol, 1.0 equiv) as the palladium species, at a 0.10 mmol scale. A yellow solid was obtained, and the product was identified by ^{31}P-NMR, and q-^{31}P-NMR indicated a >5% yield.

Example preformed PhNHK conditions:

Aniline (37.3 mg, 0.40 mmol, 4.0 equiv.) and potassium hydride (30 wt% dispersion in mineral oil, 4.8 mg, 0.12 mmol, 1.2 equiv.) were mixed in a vial inside a glovebox. The mixture was subjected to general procedure B.1, with dropwise addition of the PhNHK mixture over an hour, at a 0.10 mmol scale. A yellow solid was obtained, and the product was identified by ^{31}P-NMR, and q-^{31}P-NMR indicated a >5% yield

Best conditions:

Aniline (37.3 mg, 0.40 mmol, 4.0 equiv.) and potassium hydride (30 wt% dispersion in mineral oil, 9.6 mg, 0.24 mmol, 2.4 equiv.) were subjected to general procedure B.1, at a 0.10 mmol scale. A yellow solid was obtained (31.7 mg), and the product was identified by ^{31}P-NMR, and q-^{31}P-NMR indicated a purity of 18%. The spectra can be found in section 8.1.

trans-Chloro(4-(_para_-toluidine)-carbonyl)bis(triphenylphosphine) palladium(II) (Pd-20)

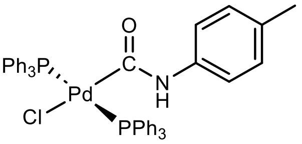

Example conditions:

para-Toluidine (42.9 mg, 0.40 mmol, 4.0 equiv.) and sodium hydride (60% dispersion in mineral oil, 4.8 mg, 0.12 mmol, 1.2 equiv.) were subjected to general procedure B.1, at a 0.10 mmol scale. A yellow solid was obtained, and the product was identified by ^{31}P-NMR, and q-^{31}P-NMR indicated a >5% yield. The spectra can be found in section 8.1.

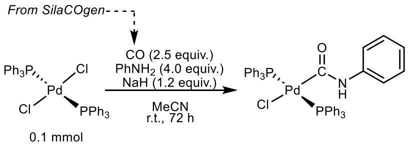

a

Entry	Deviation	NMR yield
1	Initial conditions	<5%
2	24h	<5%
3	2.0 equiv NaH	0%
4	Preformed PhNHK	0%
5	2.4 equiv KH	7%
6	$PdI_2(PPh_3)_2$, KH	<5%
7	$PdI_2(PPh_3)_2$, K_3PO_4	0%
8	$PdI_2(PPh_3)_2$, K_2CO_3	0%
9	$PdI_2(PPh_3)_2$, Et_3N	0%
10	Et_3N	<5%
11	DBU	0%

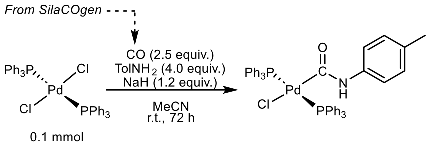

a

Entry	Deviation	NMR yield
1	Initial conditions	0%
2	DBU	0%
3	Et_3N	<5%
4	K_3PO_4	<5%
5	K_2CO_3	0%
6	*t*-BuOK	0%
7	KH	<5%

4.4 Cross-coupling scope

***N*-Propyl-[1,1'-biphenyl]-4-carboxamide (1a)**

4-Biphenylboronic ester (26.6 mg, 0.10 mmol, 1.0 equiv.) and **Pd-1** were subjected to general procedure D.1. Purification by flash column chromatography (30:70 EtOAc:heptane), yielded the product as a yellow solid (19.2 mg, 0.080 mmol, 80%) in duplicated experiments.

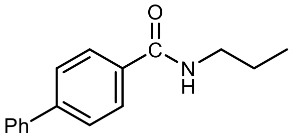

^{1}H-NMR (400 MHz, $CDCl_3$) δ 7.81 (m, 2H), 7.68 (m, 2H), 7.60 (m, 2H), 7.46 (td, *J* = 7.1, 1.3 Hz, 2H), 7.39 (m, 1H), 6.27 (s, 1H), 3.97 – 3.08 (m, 2H), 1.66 (h, *J* = 7.4 Hz, 2H), 1.00 (t, *J* = 7.4 Hz, 3H). **^{13}C-NMR** (101 MHz, $CDCl_3$) δ 167.3, 144.1, 140.1, 133.5, 128.9, 128.0, 127.4, 127.2, 127.2, 41.8, 23.0, 11.5. **HR-MS** (ESI+) Calc. for $C_{20}H_{18}NO^+$ 240.1383, found 240.1380.

***N*-Propyl-[1,1'-biphenyl]-4-^{13}C-carboxamide (^{13}C-1a)**

4-Biphenylboronic ester (26.6 mg, 0.10 mmol, 1.0 equiv.) and **Pd-1** were subjected to general procedure D.1. Purification by flash column chromatography (30:70 EtOAc:heptane), yielded the product as a yellow solid (19.4 mg, 0.081 mmol, 81%).

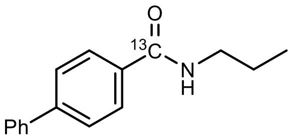

^{1}H-NMR (400 MHz, $CDCl_3$) δ 7.84 (dd, *J* = 8.2, 3.7 Hz, 2H), 7.65 (d, *J* = 7.9 Hz, 2H), 7.61 (d, *J* = 7.0 Hz, 2H), 7.46 (t, *J* = 7.5 Hz, 2H), 7.38 (t, *J* = 7.4 Hz, 1H), 6.25 (s, 1H), 3.45 (tq, *J* = 6.2, 3.1 Hz, 2H), 1.66 (h, *J* = 7.3 Hz, 2H), 1.00 (t, *J* = 7.4 Hz, 3H). **^{13}C-NMR** (101 MHz, $CDCl_3$) δ 167.4, 144.2, 140.2, 133.6, 129.0, 128.1, 127.5, 127.3, 127.3, 41.9, 23.1, 11.6. **HR-MS** (ESI+) Calc. for $C_{19}{}^{13}CH_{18}NO^+$ 241. 1416, found: 241.1419.

***N*-Benzyl-[1,1'-biphenyl]-4-carboxamide (2a)**

The relevant boronic ester 4-biphenylboronic ester (26.6 mg, 0.10 mmol, 1.0 equiv.) and **Pd-2** were subjected to general procedure D.1. Purification by flash column chromatography (15:85

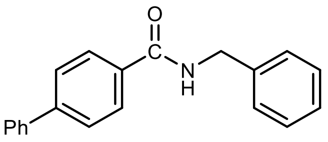

EtOAc:heptane), yielded the product as a yellow solid (13.2 mg, 0.046 mmol, 46%) in duplicated experiments.

^{1}H-NMR (400 MHz, $CDCl_3$) δ 7.87 (d, *J* = 8.4 Hz, 2H), 7.65 (d, *J* = 8.4 Hz, 2H), 7.61 (d, *J* = 7.0 Hz, 2H), 7.46 (t, *J* = 7.5 Hz, 2H), 7.35 (m, 6H), 6.53 (s, 1H), 4.68 (d, *J* = 5.7 Hz, 2H). **^{13}C-NMR** (101 MHz, $CDCl_3$) δ 167.2, 144.5, 140.1, 138.3, 133.1, 129.1, 129.0, 128.1, 128.1, 127.8, 127.6, 127.4, 127.3, 44.3. **HR-MS** (ESI+) Calc. for $C_{20}H_{18}NO^+$ 288.1383 Found: 288.1384.

***N*-(2,4-Difluorobenzyl)-[1,1'-biphenyl]-4-carboxamide (3a)**

4-Biphenylboronic ester (26.6 mg, 0.10 mmol, 1.0 equiv.) and **Pd-3** were subjected to general procedure D.1. Purification by flash column chromatography (15:85 EtOAc:heptane), yielded the product as a yellow solid (18.6 mg, 0.058 mmol, 58%) in duplicated experiments.

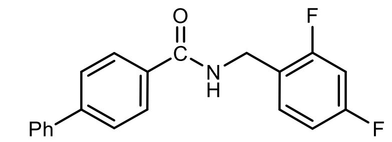

^{1}H-NMR (400 MHz, $CDCl_3$) δ 7.85 (d, *J* = 8.3 Hz, 2H), 7.65 (d, *J* = 8.4 Hz, 2H), 7.60 (d, *J* = 7.0 Hz, 2H), 7.43 (m, 4H), 6.85 (m, 2H), 6.62 (s, 1H), 4.67 (d, *J* = 5.9 Hz, 2H). **^{13}C-NMR** (101 MHz, $CDCl_3$) δ 167.3, 162.6 (dd, *J* = 248.4 Hz, *J* = 12.1 Hz),161.2 (dd, *J* = 248.9 Hz, *J* = 11.8 Hz), 144.7, 140.1, 132.8, 131.5 (dd, *J* = 9.7, 5.9 Hz), 129.1, 128.2, 127.6, 127.4, 127.3, 121.4 (dd, *J* = 15.0, 3.9 Hz), 111.6 (dd, *J* = 21.2, 3.7 Hz), 104.1 (t, *J* = 25.4 Hz), 37.7 (d, *J* = 3.3 Hz). **^{19}F-NMR** (376 MHz, $CDCl_3$) δ -110.63 (p, *J* = 7.7 Hz), -114.80 (q, *J* = 8.6 Hz). **HR-MS** (ESI+) Calc. for $C_{20}H_{16}F_2NO^+$ 324.1194, found: 324.1194.

***N*-Methyl-[1,1'-biphenyl]-4-carboxamide (4a)**

4-Biphenylboronic ester (26.6 mg, 0.10 mmol, 1.0 equiv.) and **Pd-4** were subjected to general procedure D.2 except for a temperature change to 40 °C. Purification by flash column chromatography (95:5:0 to 80:18:2 EtOAc:heptane:MeOH), yielded the product as a yellow solid (7.6 mg, 0.036 mmol, 36%).

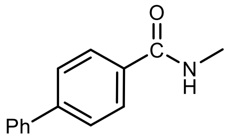

^{1}H-NMR (400 MHz, $CDCl_3$) δ 7.84 (d, *J* = 8.4 Hz, 2H), 7.64 (d, *J* = 8.3 Hz, 2H), 7.60 (dt, *J* = 6.2, 1.4 Hz, 2H), 7.46 (td, *J* = 7.3, 1.4 Hz, 2H), 7.38 (dd, *J* = 7.3, 1.4 Hz, 1H), 6.28 (s, 1H), 3.04 (d, *J* = 4.8 Hz, 3H). **^{13}C-NMR** (101 MHz, $CDCl_3$) δ 168.1, 144.3, 140.2, 133.4, 129.0, 128.7 (d, *J* = 12.1 Hz), 128.1, 127.5, 127.3 (d, *J* = 3.0 Hz), 27.0. **HR-MS** (ESI+) Calc. for $C_{14}H_{14}NO^+$ 212.1070, found: 212.1068.

***N*-Methyl-[1,1'-biphenyl]-4-^{13}C-carboxamide (^{13}C-4a)**

4-Biphenylboronic ester (26.6 mg, 0.10 mmol, 1.0 equiv.) and **Pd-4** were subjected to general procedure D.2 except for a temperature change to 40 °C. Purification by flash column chromatography (95:5:0 to 80:18:2 EtOAc:heptane:MeOH), yielded the product as a yellow solid (8.3 mg, 0.039 mmol, 39%).

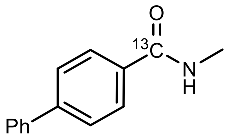

^{1}H-NMR (400 MHz, $CDCl_3$) δ 7.84 (dd, *J* = 8.3, 3.6 Hz, 2H), 7.66 (d, *J* = 8.0 Hz, 2H), 7.61 (m, 2H), 7.46 (dd, *J* = 8.4, 6.7 Hz, 1H), 7.38 (m, 1H), 6.28 (s, 1H), 3.05 (dd, *J* = 4.9, 3.4 Hz, 3H). **^{13}C-NMR** (101 MHz, $CDCl_3$) δ 68.1, 144.3, 140.2, 133.4 (d, *J* = 65.0 Hz), 129.0, 128.1,

127.5 (d, *J* = 2.3 Hz), 127.4 (d, *J* = 4.2 Hz), 127.3, 27.0. **HR-MS** (ESI+) Calc. for $C_{13}{}^{13}CH_{14}NO^+$ 213.1103, found: 213.1103.

[1,1'-Biphenyl]-4-yl(piperidin-1-yl)methanone (5a)

4-Biphenylboronic ester (26.6 mg, 0.10 mmol, 1.0 equiv.) and **Pd-5** were subjected to general procedure D.2. Purification by flash column chromatography (75:25 EtOAc:heptane), yielded the product as a white solid (26 mg, 0.097 mmol, 97%) in duplicated experiments.

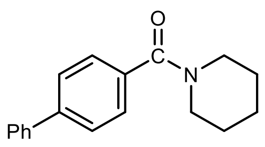

^{1}H-NMR (400 MHz, $CDCl_3$) δ 7.60 (t, *J* = 8.4 Hz, 4H), 7.46 (dd, *J* = 10.8, 7.6 Hz, 4H), 7.37 (t, *J* = 7.4 Hz, 1H), 3.90 – 3.28 (m, 4H), 1.80 – 1.48 (m, 6H). **^{13}C-NMR** (101 MHz, $CDCl_3$) δ 170.2, 142.4, 140.5, 135.4, 129.0, 127.8, 127.5, 127.2 (d, *J* = 1.3 Hz), 51.4, 31.0, 24.7. **HR-MS** (ESI+) Calc. for $C_{18}H_{20}NO^+$ 266.1539, found: 266.1552.

[1,1'-Biphenyl]-4-yl(morpholine-1-yl)methanone (6a)

4-Biphenylboronic ester (26.6 mg, 0.10 mmol, 1.0 equiv.) and **Pd-6** were subjected to general procedure D.2. Purification by flash column chromatography (75:25 EtOAc:heptane), yielded the product as a white solid (26 mg, 0.097 mmol, 97%) in duplicated experiments.

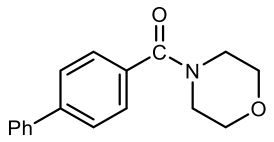

^{1}H-NMR (400 MHz, $CDCl_3$) δ 7.63 (d, *J* = 7.8 Hz, 2H), 7.59 (d, *J* = 7.6 Hz, 2H), 7.47 (dd, *J* = 16.2, 7.9 Hz, 4H), 7.38 (t, *J* = 7.3 Hz, 1H), 4.18 – 3.26 (m, 8H). **^{13}C-NMR** (101 MHz, $CDCl_3$) δ 170.4, 142.9, 140.2, 134.1, 129.0, 128.0, 127.8, 127.4, 127.3, 67.0, 31.0. **HR-MS** (ESI+) Calc. for $C_{17}H_{17}NO_2{}^+$ 268.1332, found: 268.1341.

(4-([1,1'-Biphenyl]-4-carbonyl)piperazin-1-yl)(cyclopropyl)methanone (7a)

4-Biphenylboronic ester (26.6 mg, 0.10 mmol, 1.0 equiv.) and **Pd-7** were subjected to general procedure D.2. Purification by flash column chromatography (0:50:50 to 4:48:48 MeOH:EtOAc:heptane) as a white solid (30.2 mg, 0.085 mmol, 85%).

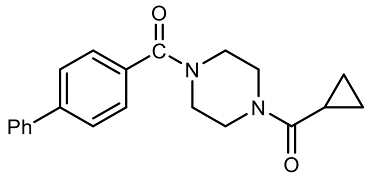

^{1}H-NMR (400 MHz, $CDCl_3$) δ 7.65 (m, 2H), 7.60 (m, 2H), 7.48 (m, 4H), 7.39 (m, 1H), 3.36 – 4.00 (m, 8H), 1.75 (br s, 1H), 1.02 (m, 2H), 0.81 (m, 2H). **^{13}C-NMR** (101 MHz, $CDCl_3$) δ 172.5, 170.7, 143.2, 140.2, 132.2 (d, *J*=9.8), 129.1, 128.0, 127.9, 127.5, 127.3, 11.2, 7.9. **HR-MS** (ESI+) Calc. for $C_{21}H_{23}N_2O_2{}^+$ 335.1754 found: 335.1765.

(4-([1,1'-Biphenyl]-4-carbonyl)piperazin-1-yl)(cyclopropyl)-^{13}C-methanone (^{13}C-7a)

4-Biphenylboronic ester (26.6 mg, 0.10 mmol, 1.0 equiv.) and **^{13}C-Pd-7** were subjected to general procedure D.2. Purification by flash column chromatography (0:50:50 to 4:48:48 MeOH:EtOAc:heptane) as a white solid (33.4 mg, 0.094 mmol, 94%).

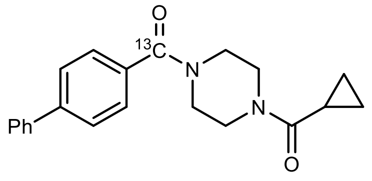

^{1}H-NMR (400 MHz, $CDCl_3$) δ 7.65 (m, 2H), 7.60 (m, 2H), 7.48 (m, 4H), 7.39 (m, 1H), 3.36 – 4.00 (m, 8H), 1.75 (br s, 1H), 1.02 (m, 2H), 0.81 (m, 2H). **^{13}C-NMR** (101 MHz, $CDCl_3$) δ 172.5, 170.7, 143.2, 140.2, 132.3, 129.1, 128.1, 127.9 (d, *J*=2.2), 127.5 (d, *J*=4.3), 127.3, 11.2, 7.9. **HR-MS** (ESI+) Calc. for $C_{20}{}^{13}CH_{23}N_2O_2{}^+$ 336.1788, found: 336.1791.

N-Propargyl-[1,1'-biphenyl]-4-carboxamide (8a)

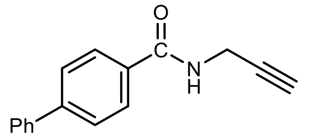

4-Biphenylboronic ester (26.6 mg, 0.10 mmol, 1.0 equiv.) and **Pd-8** were subjected to general procedure D.1. No product was detected by ^{1}H-NMR.

4.5 Boronic ester scope

4-(*tert-B*utyl)-*N*-propylbenzamide (1b)

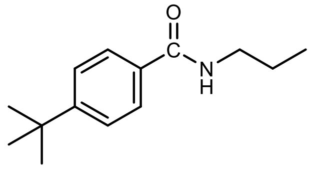

4-(*tert-B*utyl)phenylboronic ester (24.6 mg, 0.10 mmol, 1.0 equiv.) and **Pd-1** were subjected to general procedure D.1. Purification by flash column chromatography (15:85 to 25:75 EtOAc:heptane), yielded the product as a yellow oil (16.5 mg, 0.075 mmol, 75%).

^{1}H-NMR (400 MHz, $CDCl_3$) δ 7.70 (d, *J* = 8.4 Hz, 2H), 7.42 (d, *J* = 8.4 Hz, 2H), 6.28 (br s, 1H), 3.40 (q, *J* = 6.5 Hz, 2H), 1.62 (sext, *J* = 7.4 Hz, 2H), 1.32 (s, 9H), 0.96 (t, *J* = 7.4 Hz, 3H). **^{13}C-NMR** (101 MHz, $CDCl_3$) δ 167.6, 154.8, 132.1, 126.8, 125.5, 41.8, 35.0, 31.3, 23.1, 11.6. **HR-MS** (ESI+) Calc. for $C_{14}H_{22}NO^+$ 220.1696, found: 220.1697.

4-(*tert-B*utyl)-*N*-propylbenz-^{13}C-amide (^{13}C-1b)

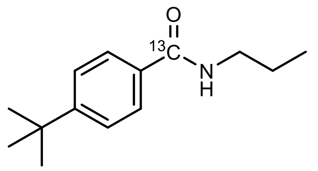

4-(*tert-B*utyl)phenylboronic ester (24.6 mg, 0.10 mmol, 1.0 equiv.) and **^{13}C-Pd-1** were subjected to general procedure D.1. Purification by flash column chromatography (15:85 EtOAc:heptane) yielded the product as a brown oil (14.3 mg, 0.065 mmol, 65%).

^{1}H-NMR (400 MHz, $CDCl_3$) δ 7.70 (dd, *J* = 8.4, 3.6 Hz, 2H), 7.43 (d, *J* = 8.2 Hz, 2H), 6.18 (br s, 1H), 3.41 (qd, *J* = 6.3, 3.1 Hz, 2H), 1.63 (sext, *J* = 7.3 Hz, 2H), 1.32 (s, 9H), 0.97 (t, *J* = 7.4 Hz, 3H). **^{13}C-NMR** (101 MHz, $CDCl_3$) δ 167.2, 162.1, 128.7, 127.3, 113.8, 55.5, 41.8, 23.1, 11.6. **HR-MS** (ESI+) Calc. for $C_{13}{}^{13}CH_{22}NO+$ 221.1729, found: 221.1736.

4-Methoxy-*N*-propylbenzamide (1c)

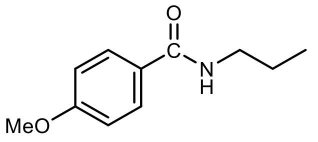

4-Methoxyphenylboronic ester (22.0 mg, 0.10 mmol, 1.0 equiv.) and **Pd-1** were subjected to general procedure D.1. Purification by flash column chromatography (10:90 to 45:55 EtOAc:heptane) yielded the product as a pale white solid (12.8 mg, 0.066 mmol, 66%) in duplicated experiments.

^{1}H-NMR (400 MHz, $CDCl_3$) δ 7.73 (d, *J* = 8.8 Hz, 2H), 6.91 (d, *J* = 8.8 Hz, 2H), 6.13 (br s, 1H), 3.84 (s, 3H), 3.60 – 3.12 (m, 2H), 1.62 (h, *J* = 7.4 Hz, 2H), 0.97 (t, *J* = 7.4 Hz, 3H). **^{13}C-NMR** (101 MHz, $CDCl_3$) δ 167.6, 154.9, 132.4, 131.8, 126.8 (d, *J* = 2.4 Hz), 125.6 (d, *J* = 4.1 Hz), 41.8, 35.0, 31.3, 23.1 (d, *J* = 1.4 Hz), 11.6. **HR-MS** (ESI+) Calc. for $C_{11}H_{16}NO_2^+$ 194.1176, found: 194.1176.

4-(Methylthio)-*N*-propylbenzamide (1d)

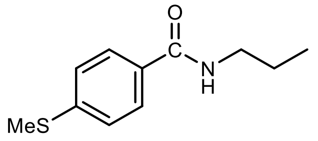

4-(Methylthio)phenylboronic ester (23.6 mg, 0.10 mmol, 1.0 equiv.) and **Pd-1** were subjected to general procedure D.1. Purification by flash column chromatography (20:80 to 35:65 EtOAc:heptane) yielded the product as a pale yellow solid (10.5 mg, 0.050 mmol, 50%) in duplicated experiments.

^{1}**H-NMR** (400 MHz, $CDCl_3$) δ 7.67 (d, *J* = 8.5 Hz, 2H), 7.24 (d, *J* = 8.5 Hz, 2H), 6.18 (br s, 1H), 3.40 (q, *J* = 7.2 Hz, 2H), 2.50 (s, 3H), 1.63 (sext, *J* = 7.4 Hz, 2H), 0.97 (t, *J* = 7.4 Hz, 3H). ^{13}**C-NMR** (101 MHz, $CDCl_3$) δ 167.1, 143.2, 131.1, 127.4, 125.6, 41.9, 23.1, 15.2, 11.6. **HR-MS** (ESI+) Calc. for $C_{11}H_{16}NOS^+$ 210.0947, found: 210.0944.

4-Cyano-*N*-propylbenzamide (1e)

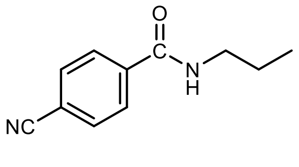

4-Cyanophenylboronic ester (21.5 mg, 0.10 mmol, 1.0 equiv.) and **Pd-1** were subjected to general procedure D.1. Purification by flash column chromatography (20:80 to 25:75 EtOAc:heptane) yielded the product as an orange sticky solid (12.4 mg, 0.066 mmol, 66%) in duplicated experiments.

^{1}**H-NMR** (400 MHz, $CDCl_3$) δ 7.86 (d, *J* = 8.5 Hz, 2H), 7.72 (d, *J* = 8.4 Hz, 2H), 6.32 (br s, 1H), 3.42 (q, *J* = 7.1 Hz, 2H), 1.64 (sext, *J* = 7.4 Hz, 2H), 0.98 (t, *J* = 7.4 Hz, 3H). ^{13}**C-NMR** (101 MHz, $CDCl_3$) δ 165.9, 138.9, 132.5, 127.7, 118.2, 115.0, 42.1, 22.9, 11.5. **HR-MS** (ESI+) Calc. for $C_{11}H_{13}N_2O^+$ 189.1022, found: 189.1021.

4-Fluoro-*N*-propylbenzamide (1f)

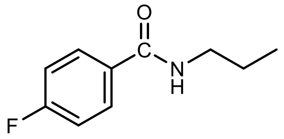

4-Fluorophenylboronic ester (20.8 mg, 0.10 mmol, 1.0 equiv.) and **Pd-1** were subjected to general procedure D.1. Purification by flash column chromatography (8:92 to 25:75 EtOAc:heptane) as a yellow solid (15.0 mg, 0.083 mmol, 83%).

^{1}**H-NMR** (400 MHz, $CDCl_3$) δ 7.77 (dd, *J* = 8.5, 5.2 Hz, 2H), 7.09 (t, *J* = 8.4 Hz, 2H), 6.21 (br s, 1H), 3.40 (q, *J* = 6.7 Hz, 2H), 1.63 (h, *J* = 7.4 Hz, 2H), 0.97 (t, *J* = 7.4 Hz, 3H). ^{13}**C-NMR** (101 MHz, $CDCl_3$) δ 166.7, 164.7 (d, *J* = 251.6 Hz), 131.1 (d, *J* = 3.1 Hz), 129.3 (d, *J* = 8.8 Hz), 115.7 (d, *J* = 21.9 Hz), 42.0, 23.0, 11.6. ^{19}**F-NMR** (376 MHz, $CDCl_3$) δ -108.60 (tt, *J* = 8.2, 5.3 Hz). **HR-MS** (ESI+) Calc. for $C_{10}H_{13}FNO^+$ 182. 0976, found: 182.0976

***N*-Propyl-4-(2,2,2-trifluoroacetyl)benzamide (1g)**

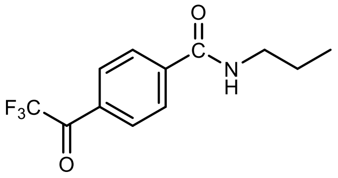

4-(2,2,2-Trifluoroethan-1-one)phenylboronic ester (28.6 mg, 0.10 mmol, 1.0 equiv.) and **Pd-1** were subjected to general procedure D.1. Purification by flash column chromatography (8:92 to 25:75 EtOAc:heptane) as a yellow oil (9.1 mg, 0.035 mmol, 35%).

^{1}**H-NMR** (400 MHz, $CDCl_3$) δ 8.13 (d, *J* = 8.7 Hz, 2H), 7.91 (d, *J* = 8.6 Hz, 2H), 6.27 (br s, 1H), 3.45 (q, *J* = 6.8 Hz, 2H), 1.67 (h, *J* = 7.4 Hz, 3H), 1.00 (t, *J* = 7.4 Hz, 4H). ^{13}**C-NMR** (101 MHz, $CDCl_3$) δ 180.1 (d, *J* = 35.6 Hz), 166.1, 141.0, 131.9, 130.5 (q, *J* = 2.2 Hz), 127.7, 116.6 (d, *J* = 292.0 Hz),115.2, 42.2, 23.0, 11.6. ^{19}**F-NMR** (376 MHz, $CDCl_3$) δ -71.62. **HR-MS** (ESI+) Calc. for $C_{12}H_{13}F_3NO_2^+$ 260.0893, found: 260.0900.

***N*-Propyl-4-(2,2,2-trifluoro-1-hydroxyethyl)benzamide (1h)**

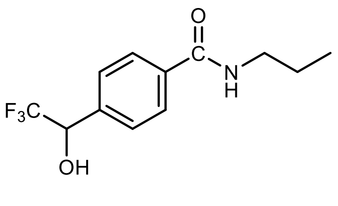

4-(2,2,2-Trifluoro-1-hydroxyethyl)phenyl-boronic ester (28.8 mg, 0.10 mmol, 1.0 equiv.) and **Pd-1** were subjected to general procedure D.1. Purification by flash column chromatography (30:70 to 50:50 EtOAc:heptane) as a yellow oil (10.4 mg, 0.04 mmol, 40%).

^{1}**H-NMR** (400 MHz, $CDCl_3$) δ 7.65 (d, *J* = 8.3 Hz, 2H), 7.46 (d, *J* = 8.0 Hz, 2H), 6.18 (br s, 1H), 5.04 (m, 1H), 3.95 (s, 1H), 3.41 (q, *J* = 6.8 Hz, 2H), 1.63 (sext,

J = 7.7 Hz, 2H), 0.98 (t, J = 7.4 Hz, 3H). **^{13}C-NMR** (101 MHz, $CDCl_3$) δ 167.7, 137.9, 135.7, 127.9, 127.0, 124.2 (d, J = 282.1 Hz), 72.3 (q, J = 32.0 Hz), 42.0, 23.0, 11.5. **^{19}F-NMR** (376 MHz, $CDCl_3$) δ -78.06 (d, J = 6.8 Hz). **HR-MS** (ESI+) Calc. for $C_{12}H_{15}F_3NO_2^+$ 262.1049, found: 262.1046.

N-Propyl-4-(2,2,2-trifluoro-1-hydroxyethyl)benz-^{13}C-amide (^{13}C-1h)

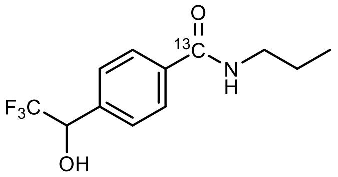

4-(2,2,2-Trifluoro-1-hydroxyethyl)phenyl-boronic ester (28.8 mg, 0.10 mmol, 1.0 equiv.) ester and **^{13}C-Pd-1** were subjected to general procedure D.1. Purification by flash column chromatography (30:70 to 50:50 EtOAc:heptane) as a yellow solid (12.6 mg, 0.048 mmol, 48%).

^{1}H-NMR (400 MHz, $CDCl_3$) δ 7.60 (dd, J = 8.2, 3.6 Hz, 2H), 7.43 (d, J = 8.0 Hz, 2H), 6.25 (br s, 1H), 5.01 (qd, J = 6.8, 4.7 Hz, 1H), 4.41 (d, J = 4.8 Hz, 1H), 3.39 (qd, J = 7.0, 3.4 Hz, 2H), 1.62 (sext, J = 7.3 Hz, 2H), 0.97 (t, J = 7.4 Hz, 3H). **^{13}C-NMR** (101 MHz, $CDCl_3$) δ 167.8, 138.0, 135.6 (d, J = 64.3 Hz), 127.9 (d, J = 4.1 Hz), 127.0 (d, J = 2.5 Hz), 124.3 (d, J = 282.2 Hz), 72.3 (q, J = 31.8 Hz), 42.0, 23.0 (d, J = 1.4 Hz), 11.5. **^{19}F-NMR** (376 MHz, $CDCl_3$) δ -78.10 (d, J = 6.7 Hz). **HR-MS** (ESI+) Calc. for $C_{11}{}^{13}CH_{15}F_3NO_2^+$ 263.1083, found: 263.1074.

***tert-B*utyl 2-(propylcarbamoyl)-10*H*-phenothiazine-10-carboxylate (1i)**

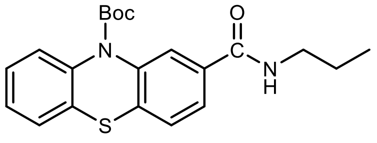

t-Butyl 10*H*-phenothiazine-10-carboxylate boronic ester (44.2 mg, 0.10 mmol, 1.0 equiv.) ester and **Pd-1** were subjected to general procedure D.1. Purification by flash column chromatography (0:100 to 25:75 EtOAc:heptane) as an off-white solid (28 mg, 0.072 mmol, 72%).

^{1}H-NMR (400 MHz, $CDCl_3$) δ 7.94 (d, J = 1.6 Hz, 1H), 7.54 (dd, J = 8.1, 1.6 Hz, 1H), 7.50 (d, J = 8.1 Hz, 1H), 7.34 (m, 2H), 7.28 (td, J = 7.5, 1.1 Hz, 1H), 7.16 (td, J = 7.7, 1.1 Hz, 1H), 6.25 (t, J = 5.5 Hz, 1H), 3.39 (q, J = 6.5 Hz, 2H), 1.62 (sext, J = 7.3 Hz, 2H), 1.49 (s, 9H), 0.97 (t, J = 7.3 Hz, 3H). **^{13}C-NMR** (101 MHz, $CDCl_3$) δ 166.6, 152.4, 138.8, 138.3, 136.1, 133.5, 131.3, 127.6, 127.5, 127.3, 127.0, 126.4, 125.8, 124.5, 82.7, 41.9, 28.3, 23.0, 11.6. **HR-MS** (ESI+) calc. for $C_{21}H_{24}N_2O_3SNa^+$ 407.1400, found: 407.1405.

***tert-B*utyl 2-(propyl-^{13}C-carbamoyl)-10H-phenothiazine-10-carboxylate (^{13}C-1i)**

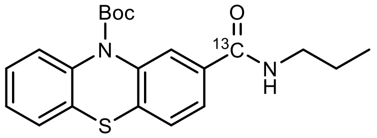

t-Butyl 10*H*-phenothiazine-10-carboxylate boronic ester (44.2 mg, 0.10 mmol, 1.0 equiv.) ester and **^{13}C-Pd-1** were subjected to general procedure D.1. Purification by flash column chromatography (0:100 to 25:75 EtOAc:heptane) as an off-white solid (32 mg, 0.081 mmol, 81%).

^{1}H-NMR (400 MHz, $CDCl_3$) δ 7.94 (dd, J = 4.0, 1.7 Hz, 1H), 7.54 (ddd, J = 8.1, 3.5, 1.7 Hz, 1H), 7.49 (dd, J = 8.1, 0.8 Hz, 1H), 7.34 (m, 2H), 7.27 (dt, J = 7.6, 1.3 Hz, 1H), 7.16 (td, J = 7.7, 1.3 Hz, 1H), 6.26 (d, J = 4.0 Hz, 1H), 3.38 (m, 2H), 1.61 (sext, J = 7.4 Hz, 2H), 1.49 (s, 9H), 0.96 (t, J = 7.4 Hz, 3H). **^{13}C-NMR** (101 MHz, $CDCl_3$) δ 166.5, 152.4, 138.8 (d, J = 5.3 Hz), 138.3, 136.1, 133.5 (d, J = 65.2 Hz), 131.3, 127.6, 127.4 (d, J = 4.6 Hz), 127.3, 127.0, 126.4, 125.8 (d, J = 2.7 Hz), 124.5 (d, J = 2.2 Hz), 82.7, 41.9, 28.3, 23.0 (d, J = 1.4 Hz), 11.6. **HR-MS** (ESI+) calc. for $C_{20}{}^{13}CH_{24}N_2O_3SNa^+$ 408.1433, found: 408.1432.

Methyl 2-(((*tert*-butyldimethylsilyl)oxy)(4-(propylcarbamoyl)phenyl)methyl)acrylate (1j)

Methyl 2-(((*tert*-butyldimethylsilyl)-oxy)(4-phenyl)methyl)-acrylate boronic ester (45 mg, 0.10 mmol, 1.0 equiv.) ester and **Pd-1** were subjected to general procedure D.1. Purification by flash column chromatography (0:100 to 30:70 EtOAc:heptane) as an off-white solid (25.3 mg, 0.065 mmol, 65%).

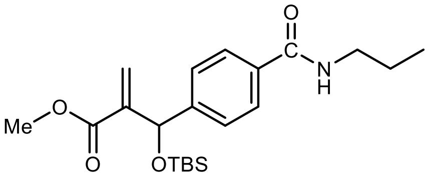

^{1}H-NMR (400 MHz, $CDCl_3$) δ 7.68 (d, *J* = 8.2 Hz, 2H), 7.42 (d, *J* = 8.2 Hz, 2H), 6.26 (t, *J* = 1.3 Hz, 1H), 6.14 (s, 1H), 6.09 (t, *J* = 1.3 Hz, 1H), 5.61 (br s, 1H), 3.66 (s, 3H), 3.40 (q, *J* = 6.8 Hz, 2H), 1.62 (sext, *J* = 7.4 Hz, 2H), 0.97 (t, *J* = 7.4 Hz, 3H), 0.86 (s, 9H), 0.05 (s, 3H), -0.11 (s, 3H). **^{13}C-NMR** (101 MHz, $CDCl_3$) δ 167.6, 166.3, 146.3, 143.5, 134.1, 127.3, 126.8, 124.4, 72.4, 51.9, 41.9, 25.8, 23.1, 18.3, 11.6, -4.8, -4.9. **HR-MS** (ESI+) calc. for $C_{21}H_{34}NO_4Si^+$ 392.2252, found: 392.2259.

Methyl 2-(((*tert*-butyldimethylsilyl)oxy)(4-(propyl-^{13}C-carbamoyl)phenyl)methyl)-acrylate (^{13}C-1j)

Methyl 2-(((*tert*-butyldimethylsilyl)-oxy)(4-phenyl)methyl)-acrylate boronic ester (45 mg, 0.10 mmol, 1.0 equiv.) ester and **Pd-1** were subjected to general procedure D.1. Purification by flash column chromatography (0:100 to 30:70 EtOAc:heptane) as an off-white solid (32.4 mg, 0.076 mmol, 76%).

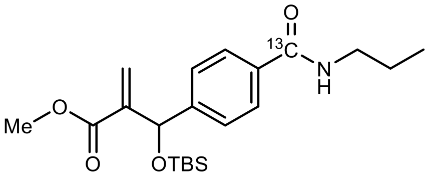

^{1}H-NMR (400 MHz, $CDCl_3$) δ 7.68 (dd, *J* = 8.2, 3.6 Hz, 2H), 7.42 (d, *J* = 8.2 Hz, 2H), 6.26 (t, *J* = 1.4 Hz, 1H), 6.12 (m, 1H), 6.10 (m, 1H), 5.62 (s, 1H), 3.66 (s, 3H), 3.40 (m, 2H), 1.62 (sext, *J* = 7.4 Hz, 2H), 0.97 (t, *J* = 7.4 Hz, 3H), 0.86 (s, 9H), 0.05 (s, 3H), -0.11 (s, 3H). **^{13}C-NMR** (101 MHz, $CDCl_3$) δ 167.6, 166.3, 146.3, 143.5, 134.1 (d, *J* = 64.6 Hz), 127.3 (d, *J* = 4.3 Hz), 126.8 (d, *J* = 2.3 Hz), 124.4, 72.4, 51.9, 41.9, 25.8, 23.1 (d, *J* = 1.4 Hz), 18.3, 11.6, -4.8, -4.9. **HR-MS** (ESI+) calc. for $C_{20}{}^{13}CH_{34}NO_4Si^+$ 393.2285, found: 393.2291

4-(3,3-Dimethylbutanamido)-3,5-difluoro-*N*-propylbenzamide (1k)

4-(3,3-Dimethylbutanamido)-3,5-difluorophenylboronic ester (33.9 mg, 0.10 mmol, 1.0 equiv.) and **Pd-1** were subjected to general procedure D.1. Purification by flash column chromatography (15:85 to 30:70 EtOAc:heptane) yielded the product as a pale yellow solid (23.3 mg, 0.075 mmol, 75%).

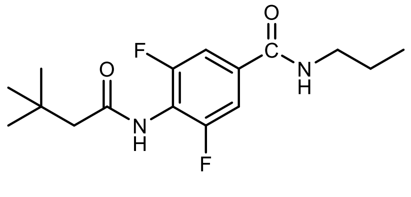

^{1}H-NMR (400 MHz, $CDCl_3$) δ 7.27 (d, *J* = 8.3 Hz, 2H), 7.11 (s, 1H), 6.50 (m, 1H), 3.38 (q, *J* = 6.8 Hz, 2H), 2.33 (s, 2H), 1.64 (q, *J* = 7.1 Hz, 2H), 1.13 (s, 9H), 0.98 (t, *J* = 7.4 Hz, 3H). **^{113}C-NMR** (101 MHz, $CDCl_3$) δ 170.4, 164.1, 157.4 (dd, *J* = 252.4 Hz, *J* = 6.0 Hz),135.2, 134.7, 110.6 (d, *J* = 24.3 Hz), 50.4, 42.2, 31.4, 29.9, 22.8, 11.5. **^{19}F-NMR** (376 MHz, $CDCl_3$) δ -115.52 (d, *J* = 7.9 Hz). **HR-MS** (ESI+) Calc. for $C_{15}{}^{13}CH_{23}F_2N_2O_2^+$ 313.1722, found: 313.1732.

4-(3,3-Dimethylbutanamido)-3,5-difluoro-*N*-propylbenz-^{13}C-amide (^{13}C-1k)

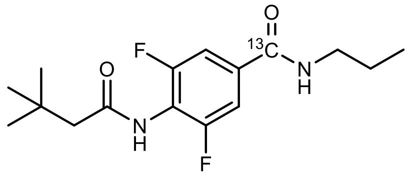

4-(3,3-Dimethylbutanamido)-3,5-difluorophenylboronic ester (33.9 mg, 0.10 mmol, 1.0 equiv.) and **^{13}C-Pd-1** were subjected to general procedure D.1. Purification by flash column chromatography (15:85 to 30:70 EtOAc:heptane) yielded the product as a pale yellow solid (20.1 mg, 0.065 mmol, 65%).

^{1}H-NMR (400 MHz, $CDCl_3$) δ 7.34 (s, 2H), 7.17 (dd, *J* = 8.0, 4.1 Hz, 1H), 6.81 (q, *J* = 5.2 Hz, 1H), 3.35 (qd, *J* = 6.1, 3.4 Hz, 2H), 2.31 (s, 2H), 1.61 (sext, *J* = 7.4 Hz, 2H), 1.12 (s, 9H), 0.95 (t, *J* = 7.4 Hz, 3H). **^{13}C-NMR** (101 MHz, $CDCl_3$) δ 170.5, 165.1 (t, *J* = 2.5 Hz), 157.4 (dt, *J* = 251.9, 6.6 Hz), 135.0, 134.3 (t, *J* = 8.1 Hz), 116.6 (t, *J* = 16.6 Hz), 110.6 (d, *J* = 23.4 Hz), 50.3, 42.1, 31.3, 29.9, 22.8, 11.5. **^{19}F-NMR** (376 MHz, $CDCl_3$) δ -115.58 (dd, *J* = 8.1, 2.6 Hz). **HR-MS** (ESI+) Calc. for $C_{15}{}^{13}CH_{23}F_2N_2O_2^+$ 314.1756, found: 314.1752.

N-Propyldibenzo[*b,d*]furan-4-carboxamide (1l)

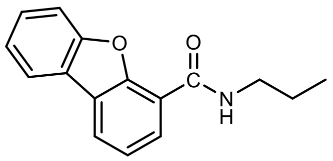

Dibenzo[*b,d*]furan-4-boronic ester (28.0 mg, 0.10 mmol, 1.0 equiv.) and **Pd-1** were subjected to general procedure D.1. Purification by flash column chromatography (8:92 to 20:80 EtOAc:heptane), yielded the product as a yellow solid (17.2 mg, 0.068 mmol, 68%) in duplicated experiments.

^{1}H-NMR (400 MHz, $CDCl_3$) δ 8.27 (dd, *J* = 7.7, 1.4 Hz, 1H), 8.06 (dd, *J* = 7.6, 1.4 Hz, 1H), 7.97 (d, *J* = 7.0 Hz, 1H), 7.69 (m, 1H), 7.63 (d, *J* = 8.0 Hz, 1H), 7.41 (m, 3H), 3.59 (m, 2H), 1.77 (h, *J* = 7.2 Hz, 2H), 1.08 (t, *J* = 7.4 Hz, 3H). **^{13}C-NMR** (101 MHz, $CDCl_3$) δ 163.8, 155.6, 153.1, 129.1, 127.8, 124.9, 124.0, 123.8, 123.6, 123.4, 121.0, 118.3, 111.7, 41.8, 23.1, 11.7. **HR-MS** (ESI+) Calc. for $C_{16}H_{16}NO_2^+$ 254.1176, found: 254.1173.

N-Propylcinnamamide (1m)

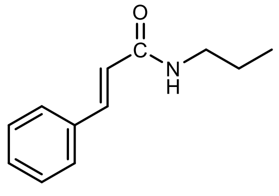

(E)-styryl boronic ester (21.6 mg, 0.10 mmol, 1.0 equiv.) and **Pd-1** were subjected to general procedure D.1. Purification by flash column chromatography (20:80 to 30:70 EtOAc:heptane) as a white solid (9.1 mg, 0.048 mmol, 48%) in duplicated experiments.

^{1}H-NMR (400 MHz, $CDCl_3$) δ 7.62 (d, *J* = 15.5 Hz, 1H), 7.49 (m, 2H), 7.35 (m, 3H), 6.40 (d, *J* = 15.6 Hz, 1H), 5.72 (br s, 1H), 3.36 (q, *J* = 6.8 Hz, 2H), 1.60 (sext, *J* = 7.4 Hz, 2H), 0.97 (t, *J* = 7.4 Hz, 3H). **^{13}C-NMR** (101 MHz, $CDCl_3$) δ 166.0, 141.0, 135.0, 129.7, 128.9, 127.9, 120.9, 41.6, 23.1, 11.6. **HR-MS** (ESI+) Calc. for $C_{12}H_{16}NO^+$ 190.1226, found: 190.1225.

N-Propyl-4-sulfamoylbenzamide (1n)

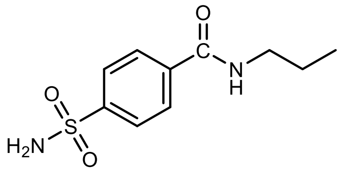

4-phenylsulfonamide boronic ester (26.9 mg, 0.10 mmol, 1.0 equiv.) and **Pd-1** were subjected to general procedure D.1. Product was detected by ^{1}H-NMR in the crude.

4-(2-Amino-2-oxoethyl)-*N*-propylbenzamide (1o)

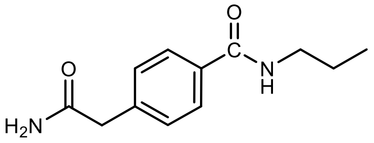

4-phenylacetamide boronic ester (24.7 mg, 0.10 mmol, 1.0 equiv.) and **Pd-1** were subjected to general procedure D.1. Product was detected in traces by ^{1}H-NMR in the crude.

***N*-Propylcyclopentanecarboxamide (1t)**

Room temperature method

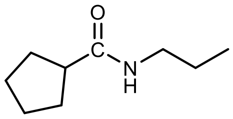

cyclopentyl boronic ester (19.6 mg, 0.10 mmol, 1.0 equiv.) and **Pd-1** were subjected to general procedure D.1. Product was not detected by ^{1}H-NMR in the crude. Starting material was isolated.

cyclopentyl boronic acid (19.6 mg, 0.10 mmol, 1.0 equiv.) and **Pd-1** were subjected to general procedure D.1. Product was not detected by ^{1}H-NMR in the crude. Starting material was isolated.

Cyclopentyl(piperidin-1-yl)methanone (1u)

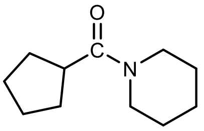

Cyclopentyl boronic ester (19.6 mg, 0.10 mmol, 1.0 equiv.) and **Pd-5** were subjected to general procedure D.2. Product was not detected by ^{1}H-NMR in the crude. Starting material was isolated.

Cyclopentyl boronic acid (11.4 mg, 0.10 mmol, 1.0 equiv.) and **Pd-5** were subjected to general procedure D.2. Product was not detected by ^{1}H-NMR in the crude. Starting material was isolated, some had hydrolyzed to the corresponding boronic acid.

4.6 API scope

[1,1'-Biphenyl]-4-yl(4-(2-chlorodibenzo[*b,f*][1,4]oxazepin-11-yl)piperazin-1-yl)-methanone (^{12}C-10a)

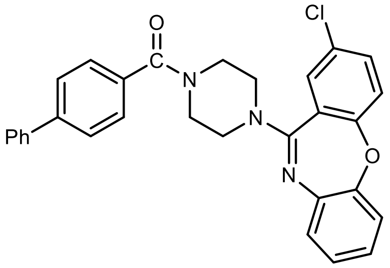

4-Biphenyl-neopentyl boronic ester (26.6 mg, 0.10 mmol, 1.0 equiv.) and Pd-amoxapine (**Pd-10**) (111 mg, 0.11 mmol, 1.10 equiv.) were subjected to general procedure D.2. Purification by flash column chromatography (20:80 to 30:70 EtOAc:heptane), yielded the product as a white solid (45.8 mg, 0.091 mmol, 91%) in duplicated results.

^{1}H-NMR (400 MHz, $CDCl_3$) δ 7.62 (m, 4H), 7.52 (m, 2H), 7.47 (m, 2H), 7.40 (m, 2H), 7.34 (d, *J* = 2.6 Hz, 1H), 7.20 (d, *J* = 8.6 Hz, 1H), 7.11 (m, 3H), 7.02 (td, *J* = 7.5, 1.9 Hz, 1H), 3.91 (m, 2H), 3.78 – 3.42 (m, 6H). **^{13}C-NMR** (101 MHz, $CDCl_3$) δ 170.6, 159.5, 158.9, 151.9, 143.0, 140.3, 139.9, 134.3, 133.0, 130.6, 129.0, 129.0, 128.0, 127.8, 127.4, 127.3, 127.3, 126.0, 125.2, 124.9, 123.0, 120.3, 47.8. **HR-MS** (ESI+) calc. for $C_{30}H_{25}ClN_3O_2^+$ 494.1630, found: 494.1671.

[1,1'-Biphenyl]-4-yl(4-(2-chlorodibenzo[*b,f*][1,4]oxazepin-11-yl)piperazin-1-yl)-^{13}C-methanone (^{13}C-10a)

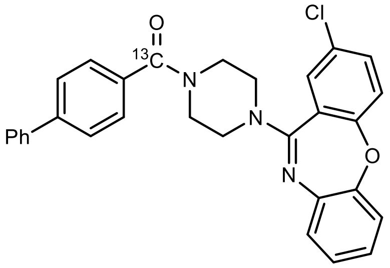

4-Biphenyl-neopentyl boronic ester (26.6 mg, 0.10 mmol, 1.0 equiv.) and ^{13}C-Pd-amoxapine (**^{13}C-Pd-10**) (111 mg, 0.11 mmol, 1.10 equiv.) were subjected to general procedure. Purification by flash column chromatography (15:85 to 30:70 EtOAc:heptane), yielded the product as a white-slight yellow solid (47.2 mg, 0.95 mmol, 95%) in duplicated results.

^{1}H-NMR (400 MHz, $CDCl_3$) δ 7.62 (m, 4H), 7.52 (m, 2H), 7.47 (m, 2H), 7.40 (m, 2H), 7.34 (d, *J* = 2.6 Hz, 1H), 7.20 (d, *J* = 8.6 Hz, 1H), 7.12 (m, 3H), 7.02 (td, *J* = 7.5, 1.9 Hz, 1H), 4.08 – 3.78 (m, 2H), 3.77 – 3.40 (m, 6H). **^{13}C-NMR** (101 MHz, $CDCl_3$) δ 170.6, 159.5, 158.9, 151.9, 143.0, 140.3, 139.9, 134.3 (d, *J* = 66.9 Hz), 133.0, 130.6, 129.1, 129.0, 128.0, 127.8 (d, *J* = 2.2 Hz), 127.4 (d, *J* = 4.3 Hz), 127.3, 127.3, 126.0, 125.2, 124.9, 123.0, 120.3, 47.8. **HR-MS** (ESI+) calc. for $C_{29}{}^{13}CH_{25}ClN_3O_2^+$ 495.1663, found: 495.1666.

[1,1'-Biphenyl]-4-yl(4-(2-chlorodibenzo[*b,f*][1,4]oxazepin-11-yl)piperazin-1-yl)-^{14}C-methanone (^{14}C-10a)

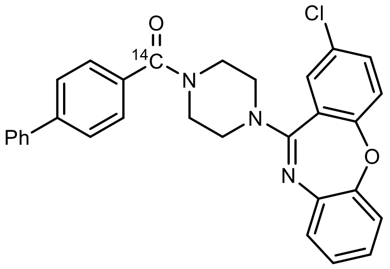

4-Biphenyl-neopentyl boronic ester (26.6 mg, 0.10 mmol, 1.0 equiv.) and ^{14}C-Pd-amoxapine (**^{14}C-Pd-10**) (21.3 MBq, 111 mg, 0.11 mmol, 1.10 equiv.) were subjected to general procedure E. Purification by preparative HPLC (48:52 to 58:42 MeOH:NH_3 2 v/v% in H_2O for 13 min, wavelength of 265nm, 20 mL/min). yielded the product as a brown solid in (46.1 mg, 15.3 MBq, SA: 164 MBq/mmol or 406 MBq/mmol by MS, 92%, 72% RCY, >99% RCP).

^{1}H-NMR (400 MHz, $CDCl_3$) δ 7.64 (m, 2H), 7.60 (m, 2H), 7.52 (m, 2H), 7.46 (dd, *J* = 8.4, 6.9 Hz, 2H), 7.39 (m, 2H), 7.34 (d, *J* = 2.5 Hz, 1H), 7.20 (d, *J* = 8.7 Hz, 1H), 7.16 (dd, *J* = 7.8,

1.8 Hz, 1H), 7.11 (m, 2H), 7.02 (td, *J* = 7.5, 1.7 Hz, 1H), 3.75 (m, 8H). **^{13}C-NMR** (101 MHz, $CDCl_3$) δ 170.5, 159.5, 158.8, 151.9, 143.0, 140.3, 139.9, 134.3, 132.9, 130.6, 129.0, 129.0, 128.0, 127.8, 127.4, 127.3, 127.2, 126.0, 125.1, 124.9, 123.0, 120.3, 47.8. **LR-LCMS** (ESI+) calc. for $C_{29}{}^{14}CH_{25}ClN_3O_2^+$ 496.1663, found: 496.2.

6-(4-(2-Chlorodibenzo[*b,f*][1,4]oxazepin-11-yl)piperazine-1-carbonyl)-1-methyl-indolin-2-one (^{12}C-10p)

1-Methyl-6-(4,4,5,5-tetramethyl-1,3,2-dioxaborolan-2-yl)indolin-2-one (27.3 mg, 0.10 mmol, 1.0 equiv.) and Pd-Amoxapine (**Pd-10**) (111 mg, 0.11 mmol, 1.10 equiv.) were subjected to general procedure D.2. Purification by flash column chromatography (0:40:60 to 4:36:60 MeOH:EtOAc:heptane), yielded the product as a white solid (37.0 mg, 0.069 mmol, 69%).

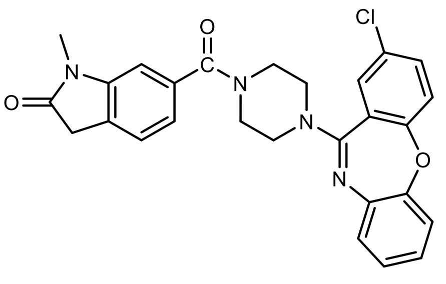

^{1}H-NMR (400 MHz, $CDCl_3$) δ 7.34 (dd, *J* = 8.6, 2.6 Hz, 1H), 7.26 (d, *J* = 2.6 Hz, 1H), 7.19 (t, *J* = 3.8 Hz, 1H), 7.13 (d, *J* = 8.6 Hz, 1H), 7.01 (m, 5H), 6.85 (d, *J* = 1.4 Hz, 1H), 3.98 – 3.37 (m, 10H), 3.15 (s, 3H). **^{13}C-NMR** (101 MHz, $CDCl_3$) δ 174.9, 170.3, 159.5, 158.9, 151.9, 145.9, 139.8, 135.6, 133.0, 130.6, 129.0, 127.2, 126.5, 126.0, 125.3, 124.9, 124.3, 123.0, 121.0, 120.3, 107.2, 35.7, 26.5. **HR-MS** (ESI+) calc. for $C_{27}H_{24}ClN_4O_3^+$ 487.1531, found: 487.1536.

6-(4-(2-Chlorodibenzo[*b,f*][1,4]oxazepin-11-yl)piperazine-1-^{13}C-carbonyl)-1-methyl-indolin-2-one (^{13}C-10p)

1-Methyl-6-(4,4,5,5-tetramethyl-1,3,2-dioxaborolan-2-yl)-indolin-2-one (27.3 mg, 0.10 mmol, 1.0 equiv.) and Pd-Amoxapine (**^{13}C-Pd-10**) (111 mg, 0.11 mmol, 1.10 equiv.) were subjected to general procedure D.2. Purification by flash column chromatography (0:40:60 to 4:36:60 MeOH:EtOAc:heptane), yielded the product as a white solid (39.6 mg, 0.074 mmol, 74%).

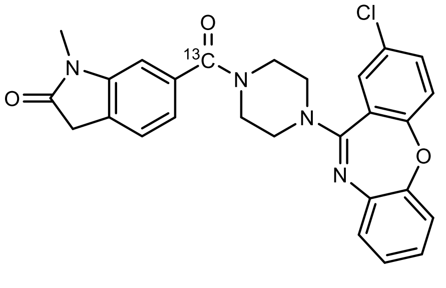

^{1}H-NMR (400 MHz, $CDCl_3$) δ 7.33 (dd, *J* = 8.6, 2.6 Hz, 1H), 7.25 (s, 1H), 7.20 (m, 1H), 7.13 (d, *J* = 8.6 Hz, 1H), 7.00 (m, 5H), 6.84 (m, 1H), 3.82 – 3.48 (m, 10H), 3.15 (s, 3H). **^{13}C-NMR** (101 MHz, $CDCl_3$) δ 174.9, 170.3, 159.5, 158.8, 151.8, 145.9 (d, *J* = 5.4 Hz), 139.8, 135.5 (d, *J* = 66.5 Hz), 133.0, 130.6, 128.9, 127.2, 126.5, 126.0, 125.2, 124.8, 124.2 (d, *J* = 4.8 Hz), 123.0, 121.0 (d, *J* = 2.1 Hz), 120.3, 107.2 (d, *J* = 2.6 Hz), 35.7, 26.4. **HR-MS** (ESI+) calc. for $C_{27}{}^{13}CH_{24}ClN_4O_3^+$ 488.1565, found: 488.1573

6-(4-(2-Chlorodibenzo[*b,f*][1,4]oxazepin-11-yl)piperazine-1-^{14}C-carbonyl)-1-methyl-indolin-2-one (^{14}C-10p)

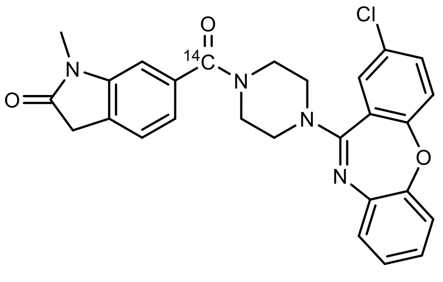

1-Methyl-6-(4,4,5,5-tetramethyl-1,3,2-dioxaborolan-2-yl)-indolin-2-one (28.7 mg, 0.105 mmol, 1.0 equiv.) and Pd-Amoxapine (**^{14}C-Pd-10**) (22.5 MBq, 117 mg, 1.16 mmol, 1.10 equiv.) were subjected to general procedure E. Purification by preparative HPLC (48:52 to 58:42 MeCN:NH_3 2 v/v% in H_2O for 17 min, wavelength of 265nm, 20 mL/min). yielded the product as a brown solid in (41.8 mg, 13.5 MBq, SA: 157 MBq/mmol or 32 MBq/mmol by MS, 78%, 60% RCY, >99% RCP in basic conditions and 97% in acidic conditions).

^{1}H-NMR (400 MHz, $CDCl_3$) δ 7.42 (dd, *J* = 8.7, 2.6 Hz, 1H), 7.34 (d, *J* = 2.6 Hz, 1H), 7.28 (d, *J* = 7.5 Hz, 1H), 7.22 (d, *J* = 8.7 Hz, 1H), 7.15 (dd, *J* = 7.8, 1.8 Hz, 1H), 7.12 (ddd, *J* = 7.6, 4.1, 1.6 Hz, 2H), 7.09 (dd, *J* = 7.5, 1.5 Hz, 1H), 7.04 (td, *J* = 7.5, 1.9 Hz, 1H), 6.94 (d, *J* = 1.4 Hz, 1H), 4.03 – 3.39 (m, 10H), 3.24 (s, 3H). **^{13}C-NMR** (101 MHz, $CDCl_3$) δ 174.9, 170.3, 159.5, 158.8, 151.9, 145.9, 139.8, 133.0, 130.6, 128.9, 127.2, 126.5, 126.0, 125.2, 124.9, 124.2, 123.0, 121.0, 120.3, 35.7, 26.4. **LR-LCMS** (ESI+) calc. for $C_{27}{}^{14}CH_{24}ClN_4O_3^+$ 489.1564, found: 489.1

5-(4-(2-Chlorodibenzo[b,f][1,4]oxazepin-11-yl)piperazine-1-carbonyl)-2-(2,6-dioxo-piperidin-3-yl)isoindoline-1,3-dione (^{12}C-10q)

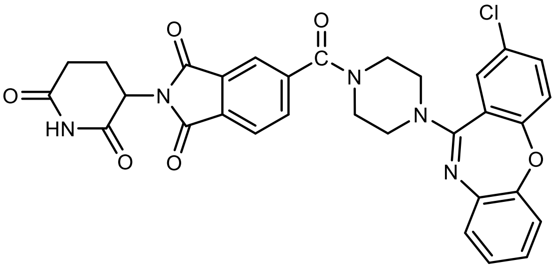

5-(5,5-dimethyl-1,3,2-dioxaborinan-2-yl)-2-(2,6-di-oxopiperidin-3-yl)isoindoline-1,3-dione (37.0 mg, 0.10 mmol, 1.0 equiv.) and Pd-amoxapine (**Pd-10**) (111 mg, 0.11 mmol, 1.10 equiv.) were subjected to general procedure D.2. Purification by flash column chromatography (50:50 to 100:0 EtOAc:heptane), yielded the product as a yellow solid (7.6 mg, 0.013 mmol, 12%).

^{1}H-NMR (400 MHz, $CDCl_3$) δ 8.14 (br s, 1H), 7.95 (m, 2H), 7.83 (dd, *J* = 7.6, 1.4 Hz, 1H), 7.42 (dd, *J* = 8.6, 2.6 Hz, 1H), 7.33 (d, *J* = 2.5 Hz, 1H), 7.21 (d, *J* = 8.6 Hz, 1H), 7.11 (m, 3H), 7.03 (td, *J* = 7.4, 1.9 Hz, 1H), 5.01 (dd, *J* = 12.4, 5.3 Hz, 1H), 3.93 (br s, 2H), 3.68 – 3.49 (m, 6H), 2.98 – 2.69 (m, 3H), 2.18 (m, 1H). **^{13}C-NMR** (101 MHz, $CDCl_3$) δ 170.8, 168.1, 167.8, 166.5 (d, *J*=7.0), 159.6, 158.7, 151.9, 142.0, 139.7, 133.5, 133.1, 132.6, 132.2, 130.7, 128.9, 127.3, 126.1, 125.4, 124.8, 124.5, 123.1, 122.5, 120.4, 71.9, 49.7, 47.7, 31.5, 22.7. **HR-MS** (ESI+) calc. for $C_{31}CH_{25}ClN_5O_6^+$ 598.1488, found: 598.1523.

5-(4-(2-Chlorodibenzo[b,f][1,4]oxazepin-11-yl)piperazine-1-^{13}C-carbonyl)-2-(2,6-di-oxo-piperidin-3-yl)isoindoline-1,3-dione (^{13}C-10q)

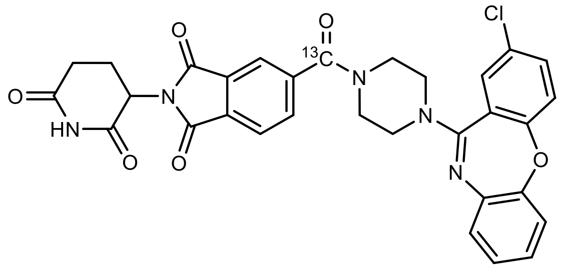

5-(5,5-dimethyl-1,3,2-dioxaborinan-2-yl)-2-(2,6-di-oxopiperidin-3-yl)isoindoline-1,3-dione (37.0 mg, 0.10 mmol, 1.0 equiv.) and Pd-amoxapine (**^{13}C-Pd-10**) (111 mg, 0.11 mmol, 1.10 equiv.) were subjected to general procedure D.2. Purification by flash column chromatography (50:50 to 100:0 EtOAc:heptane), yielded the product as a yellow solid (11.4 mg, 0.018 mmol, 18%).

^{1}H-NMR (400 MHz, $CDCl_3$) δ 8.25 (m, 1H), 7.95 (m, 2H), 7.83 (ddd, *J* = 7.6, 3.8, 1.4 Hz, 1H), 7.42 (dd, *J* = 8.7, 2.6 Hz, 1H), 7.32 (d, *J* = 2.6 Hz, 1H), 7.20 (d, *J* = 8.6 Hz, 1H), 7.12 (m, 3H), 7.03 (td, *J* = 7.4, 2.0 Hz, 1H), 5.00 (dd, *J* = 12.3, 5.3 Hz, 1H), 3.93 (br s , 2H), 3.67 – 3.48 (m, 6H), 2.97 – 2.69 (m, 3H), 2.17 (m, 1H). **^{13}C-NMR** (101 MHz, $CDCl_3$) δ 170.8, 168.1, 167.8, 166.5 (d, *J*=7.1), 159.5, 158.7, 151.9, 142.0 (d, *J*=66.1), 139.7, 133.5 (d), 133.1, 132.6, 132.3, 130.7, 128.9, 127.3, 126.0, 125.4, 124.8, 124.5 (d, *J*=4.4), 123.1, 122.5, 120.4, 71.6, 49.7, 47.6 (m), 31.5, 22.7. **HR-MS** (ESI+) calc. for $C_{30}{}^{13}CH_{25}ClN_5O_6^+$ 599.1521, found: 599.1561.

2-(2,6-Dioxopiperidin-3-yl)-5-(piperidine-1-carbonyl)isoindoline-1,3-dione (^{12}C-5q)

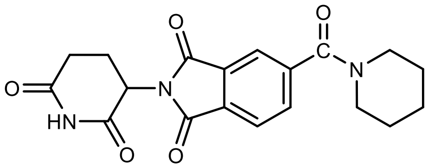

5-(5,5-dimethyl-1,3,2-dioxaborinan-2-yl)-2-(2,6-dioxopiper-idin-3-yl)isoindoline-1,3-dione (37.0 mg, 0.10 mmol, 1.0 equiv.) and **Pd-5** were subjected to general procedure D.2. Purification by flash column chromatography (50:50 to 100:0 EtOAc:heptane), yielded the product as a yellow solid (6.4 mg, 0.017 mmol, 17%).

^{1}H-NMR (400 MHz, $CDCl_3$) δ 8.11 (s, 1H), 7.93 (d, *J* = 7.6 Hz, 1H), 7.87 (s, 1H), 7.78 (d, *J* = 7.6 Hz, 1H), 5.00 (dd, *J* = 12.4, 5.2 Hz, 1H), 3.74 (s, 2H), 3.30 (s, 2H), 2.93 (d, *J* = 17.1 Hz, 1H), 2.89 – 2.65 (m, 2H), 2.36 – 2.02 (m, 1H), 1.67 (d, *J* = 31.7 Hz, 8H). **^{13}C-NMR** (101 MHz, $CDCl_3$) δ 170.8, 167.9, 167.8, 166.7, 166.7, 143.1, 133.2, 132.3 (d, *J* = 9.7 Hz), 128.7 (d, *J* = 12.3 Hz), 124.4, 122.2, 49.7, 31.5, 24.5, 22.7. **HR-MS** (ESI+) calc. for C19H20N3O5+ 370.1397, found: 370.1397.

2-(2,6-Dioxopiperidin-3-yl)-5-(piperidine-1-^{13}C-carbonyl)isoindoline-1,3-dione (^{13}C-5q)

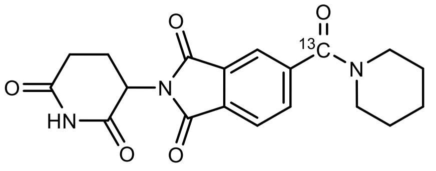

5-(5,5-dimethyl-1,3,2-dioxaborinan-2-yl)-2-(2,6-dioxopiper-idin-3-yl)isoindoline-1,3-dione (37.0 mg, 0.10 mmol, 1.0 equiv.) and **^{13}C-Pd-5** were subjected to general procedure D.2. Purification by flash column chromatography (50:50 to 100:0 EtOAc:heptane), yielded the product as a yellow solid (6.2 mg, 0.017 mmol, 17%).

^{1}H-NMR (400 MHz, $CDCl_3$) δ 8.14 (s, 1H), 7.93 (d, *J* = 7.6 Hz, 1H), 7.87 (d, *J* = 3.6 Hz, 1H), 7.78 (ddd, *J* = 7.6, 3.8, 1.4 Hz, 1H), 5.00 (dd, *J* = 12.3, 5.2 Hz, 1H), 3.74 (s, 2H), 3.30 (s, 2H), 2.93 (d, *J* = 17.5 Hz, 1H), 2.89 – 2.68 (m, 2H), 2.18 (ddd, *J* = 10.6, 5.0, 2.8 Hz, 1H), 1.79 – 1.49 (m, 4H). **^{13}C-NMR** (101 MHz, $CDCl_3$) δ 170.8, 167.9, 167.8, 166.7 (d, *J* =

2.9 Hz), 166.1, 143.1 (d, *J* = 65.9 Hz), 133.2 (d, *J* = 2.3 Hz), 128.7 (d, *J* = 12.1 Hz), 124.4 (d, *J* = 4.3 Hz), 122.2 (d, *J* = 2.3 Hz), 49.7, 31.5, 24.5, 22.7. **HR-MS** (ESI+) calc. for $C_{18}{}^{13}CH_{20}N_3O_5^+$ 371.1431, found: 371.1431

7-(4-([1,1'-Biphenyl]-4-carbonyl)piperazin-1-yl)-1-cyclopropyl-6-fluoro-4-oxo-1,4-dihydro-quinoline-3-carboxylic acid (^{12}C-11a)

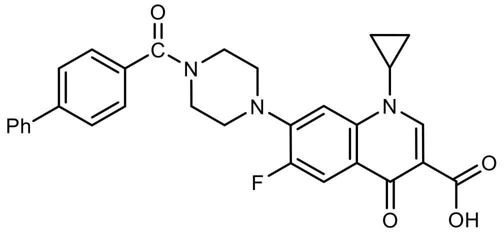

4-Biphenyl-neopentyl boronic ester (26.6 mg, 0.10 mmol, 1.0 equiv.) and Pd-ciprofloxacin (**Pd-11**) (112 mg, 0.11 mmol, 1.10 equiv.) were subjected to general procedure D.2. Purification by flash column chromatography (0:100 to 95:5 MeOH:EtOAc), yielded the product as a white solid (39 mg, 0.076 mmol, 76%).

^{1}H-NMR (400 MHz, $CDCl_3$) δ 14.92 (br s, 1H), 8.77 (s, 1H), 8.04 (d, *J* = 12.7 Hz, 1H), 7.67 (d, *J* = 7.6 Hz, 3H), 7.60 (d, *J* = 7.0 Hz, 1H), 7.55 (d, *J* = 7.5 Hz, 1H), 7.47 (d, *J* = 7.5 Hz, 2H), 7.40 (t, *J* = 6.9 Hz, 2H), 3.90 (d, *J* = 95.5 Hz, 4H), 3.54 (br s, 1H), 3.37 (s, 3H), 1.40 (d, *J* = 6.9 Hz, 2H), 1.21 (m, 4H). **^{13}C-NMR** (101 MHz, $CDCl_3$) δ 177.2, 170.6, 167.0, 155.0, 152.5, 147.7, 145.6 (d, *J* = 10.3 Hz), 143.3, 140.2, 139.1, 134.9 (d, *J* = 9.5 Hz), 133.9, 128.5 (dd, *J* = 29.1, 12.0 Hz), 128.2 (d, *J* = 183.0 Hz), 128.1, 127.7 (d, *J* = 48.6 Hz), 120.5 (d, *J* = 7.8 Hz), 112.8 (d, *J* = 23.5 Hz), 108.3, 105.4 (d, *J* = 3.0 Hz), 35.5, 29.8, 8.4. **^{19}F-NMR** (376 MHz, $CDCl_3$) δ -121.12 (dd, *J* = 12.9, 6.9 Hz). **HR-MS** (ESI+) calc. for $C_{30}H_{27}FN_3O_4^+$ 512.1980, found: 512.1980.

7-(4-([1,1'-Biphenyl]-4-^{13}C-carbonyl)piperazin-1-yl)-1-cyclopropyl-6-fluoro-4-oxo-1,4-dihydroquinoline-3-carboxylic acid (^{13}C-11a)

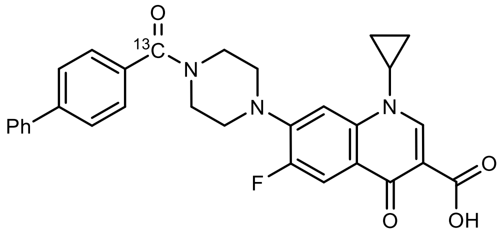

4-Biphenyl-neopentyl boronic ester (26.6 mg, 0.10 mmol, 1.0 equiv.) and Pd-ciprofloxacin (**Pd-11**) (112 mg, 0.11 mmol, 1.10 equiv.) were subjected to general procedure D.2. Purification by flash column chromatography (0:100 to 95:5 MeOH:EtOAc), yielded the product as a white solid (37 mg, 0.072 mmol, 72%).

^{1}H-NMR (400 MHz, $CDCl_3$) δ 14.84 (br s, 1H), 8.71 (s, 1H), 7.96 (d, *J* = 12.7 Hz, 1H), 7.65 (d, *J* = 7.8 Hz, 2H), 7.59 (d, *J* = 7.5 Hz, 2H), 7.54 (t, *J* = 4.1 Hz, 2H), 7.46 (t, *J* = 7.5 Hz, 2H), 7.38 (t, *J* = 7.8 Hz, 2H), 3.91 (m, 3H), 3.55 (s, 1H), 3.37 (br s, 3H), 1.39 (d, *J* = 6.6 Hz, 2H), 1.24 (s, 3H), 1.19 (s, 1H). **^{13}C-NMR** (101 MHz, $CDCl_3$) δ 170.6, 167.1, 147.8, 140.2, 128.6, 128.2 (d, *J* = 183.0 Hz), 128.1, 128.0, 127.5 (d, *J* = 4.5 Hz), 34.7 (d, *J* = 157.8 Hz), 29.8, 8.5. Some Sp^2 carbons missing due to splitting. **^{19}F-NMR** (376 MHz, $CDCl_3$) δ -121.14 (dd, *J* = 12.6, 7.0 Hz). **HR-MS** (ESI+) calc. for $C_{29}{}^{13}CH_{27}FN_3O_4^+$ 513.2014, found: 513.2019.

[1,1'-Biphenyl]-4-yl((3*S*,4*R*)-3-((benzo[*d*][1,3]dioxol-5-yloxy)methyl)-4-(4-fluoro-phenyl)-piperidin-1-yl)methanone (^{12}C-12a)

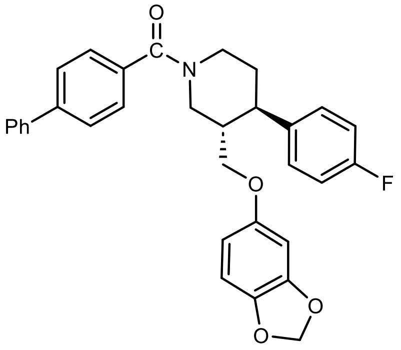

4-Biphenyl-neopentyl boronic ester (26.6 mg, 0.10 mmol, 1.0 equiv.) and Pd-paroxetine (**Pd-12**) (112 mg, 0.11 mmol, 1.10 equiv.) were subjected to general procedure D.2. Purification by flash column chromatography (20:80 to 30:70 EtOAc:heptane), yielded the product as a yellow solid (42.6 mg, 0.084 mmol, 84%).

^{1}H-NMR (400 MHz, $CDCl_3$) δ 7.62 (m, 4H), 7.54 (d, *J*=8.0, 2H), 7.47 (t, *J*=7.5, 2H), 7.38 (t, *J*=7.3, 1H), 7.18 (dd, *J*=8.5, 5.5, 2H), 7.00 (t, *J*=8.4, 2H), 6.62 (m, 1H), 5.94 – 6.46 (m, 2H), 5.86 (m, 2H), 4.83 – 5.11 (m, 1H), 3.93 – 4.30 (m, 1H), 3.32 – 3.77 (m, 2H), 3.06 – 3.29 (m, 1H), 2.71 – 3.05 (m, 2H), 2.14 (m, 1H), 1.63 – 2.02 (m, 2H). **^{13}C-NMR** (101 MHz, $CDCl_3$) δ 170.4, 161.8 (d, *J* = 245.0 Hz),148.2, 142.7, 141.8, 140.3, 138.5, 128.9, 128.8 (d, *J*=7.8), 127.8, 127.7, 127.2 (d, *J*=2.9), 115.8, 115.6, 107.9, 105.5, 101.2, 97.9. **^{19}F-NMR** (376 MHz, DMSO) δ -116.39 (tt, *J*=9.0, 5.5). **HR-MS** (ESI+) calc. for $C_{32}H_{29}FNO_4^+$ 510.2075, found: 510.2097.

[1,1'-Biphenyl]-4-yl((3*S*,4*R*)-3-((benzo[*d*][1,3]dioxol-5-yloxy)methyl)-4-(4-fluoro-phenyl)-piperidin-1-yl)-^{13}C-methanone (^{13}C-12a)

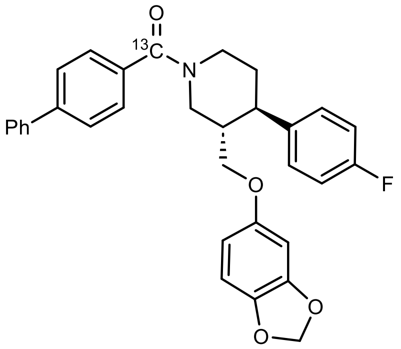

4-Biphenyl-neopentyl boronic ester (26.6 mg, 0.10 mmol, 1.0 equiv.) and Pd-paroxetine (**^{13}C-Pd-12**) (112 mg, 0.11 mmol, 1.10 equiv.) were subjected to general procedure D.2. Purification by flash column chromatography (20:80 to 30:70 EtOAc:heptane), yielded the product as a white-faint-brown solid (45.2 mg, 0.089 mmol, 89%).

^{1}H-NMR (400 MHz, $CDCl_3$) δ 7.63 (dd, *J*=13.5, 7.5, 4H), 7.54 (dd, *J*=8.3, 3.7, 2H), 7.47 (t, *J*=7.5, 2H), 7.38 (t, *J*=7.3, 1H), 7.18 (m, 2H), 7.00 (t, *J*=8.5, 2H), 6.62 (m, 1H), 5.95 – 6.45 (m, 2H), 5.87 (s, 2H), 4.84 – 5.15 (m, 1H), 3.94 – 4.29 (m, 1H), 3.32 – 3.79 (m, 2H), 3.05 – 3.30 (m, 1H), 2.69 – 3.04 (m, 2H), 2.13 (m, 1H), 1.66 – 2.02 (m, 2H). **^{13}C-NMR** (101 MHz, $CDCl_3$) δ 170.5, 161.8(d, *J* = 245.2 Hz), 148.3, 142.8, 141.9, 140.4, 138.7, 129.0, 128.9 (d, *J*=8.0), 127.9, 127.8, 127.3 (d, *J*=4.8), 115.9, 115.7, 108.0, 105.7, 101.3, 98.1, 68.5, 44.3, 43.0, 42.3, 32.0. **^{19}F-NMR** (376 MHz, $CDCl_3$) δ -115.75 (m). **HR-MS** (ESI+) calc. for $C_{31}{}^{13}CH_{29}FNO_4^+$ 511.2109, found: 511.2131.

((*3R,4S*)-3-((Benzo[d][1,3]dioxol-5-yloxy)methyl)-4-(4-fluorophenyl)piperidin-1-yl)(4-(((2-ethylquinolin-4-yl)oxy)methyl)phenyl)methanone (^{12}C-12r)

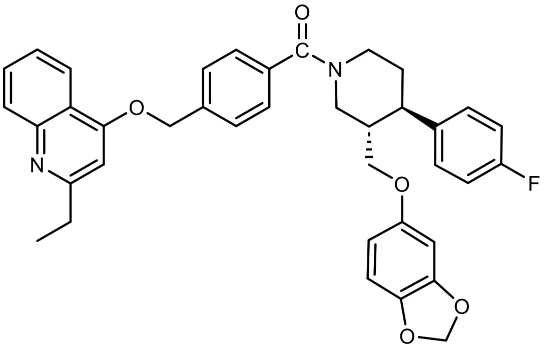

(4-(((2-Ethylquinolin-4-yl)oxy)methyl)phenyl)-boronic acid (30.7 mg, 0.10 mmol, 1.0 equiv.) and Pd-paroxetine (**Pd-12**) (112 mg, 0.11 mmol, 1.10 equiv.) were subjected to general procedure D.2. Purification by flash column chromatography (20:80 to 30:70 EtOAc:heptane), yielded the product as a white solid (23.3 mg, 0.038 mmol, 38%).

^{1}H-NMR (400 MHz, $CDCl_3$) δ 8.21 (d, *J*=7.8, 1H), 7.99 (d, *J*=8.2, 1H), 7.68 (m, 2H), 7.57 (m, 4H), 7.47 (m, 2H), 7.17 (td, *J*=5.4, 2.4, 2H), 7.00 (t, *J*=8.9, 2H), 6.73 (s, 1H), 6.52 – 6.69 (m, 1H), 5.94 – 6.45 (m, 2H), 5.87 (m, 2H), 5.33 (s, 2H), 4.83 – 5.11 (m, 1H), 3.83 – 4.27 (m, 1H), 3.33 – 3.76 (m, 2H), 3.04 – 3.28 (m, 1H), 2.95 (q, *J*=7.7, 2H), 2.82 (m, 2H), 2.03 – 2.22 (m, 1H), 1.76 – 2.03 (m, 2H), 1.38 (t, *J*=7.6, 3H). **^{13}C-NMR** (101 MHz, $CDCl_3$) *Spectrum attached, but not reported due to low intensity.* **^{19}F-NMR** (376 MHz, $CDCl_3$) δ -115.68 (d, *J*=55.2). **HR-MS** (ESI+) calc. for $C_{37}{}^{12}CH_{36}FN_2O_5^+$ 619.2603, found: 619.2647.

((*3R,4S*)-3-((Benzo[d][1,3]dioxol-5-yloxy)methyl)-4-(4-fluorophenyl)piperidin-1-yl)(4-(((2-ethylquinolin-4-yl)oxy)methyl)phenyl)-^{13}C-methanone (^{13}C-12r)

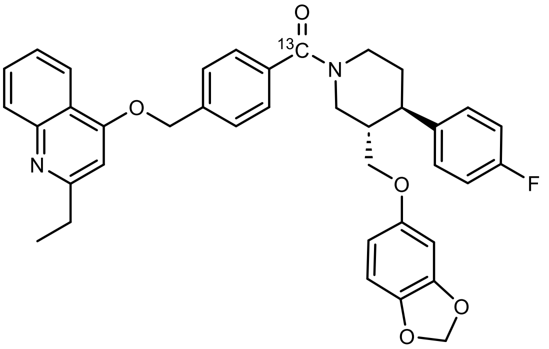

(4-(((2-Ethylquinolin-4-yl)oxy)methyl)phenyl)-boronic acid (30.7 mg, 0.10 mmol, 1.0 equiv.) and Pd-paroxetine (**^{13}C-Pd-12**) (112 mg, 0.11 mmol, 1.10 equiv.) were subjected to general procedure D.2. Purification by flash column chromatography (20:80 to 30:70 EtOAc:heptane), yielded the product as a white solid (47.2 mg, 0.044 mmol, 44%).

^{1}H-NMR (400 MHz, $CDCl_3$) δ 8.21 (d, *J*=8.3, 1H), 7.99 (d, *J*=8.4, 1H), 7.64 (m, 2H), 7.52 (m, 4H), 7.43 (m, 2H), 7.16 (m, 2H), 7.00 (t, *J*=8.4, 2H), 6.73 (s, 1H), 6.61 (m, 1H), 5.94 – 6.43 (m, 2H), 5.86 (m, 2H), 5.33 (s, 2H), 4.82 – 5.10 (m, 1H), 3.85 – 4.22 (m, 1H), 3.33 – 3.77 (m, 2H), 3.04 – 3.26 (m, 1H), 2.95 (q, *J*=7.7, 2H), 2.68 – 2.92 (m, 2H), 2.04 – 2.21 (m, 1H), 1.93 (m, 2H), 1.38 (t, *J*=7.6, 3H). **^{13}C-NMR** (101 MHz, $CDCl_3$) δ 170.1, 165.3, 161.4, 149.1, 148.3, 141.9, 138.6, 137.8, 135.0 (d, *J*=10.7), 133.2, 132.3, 130.0, 127.6 (d, *J*=12.3), 125.1, 121.8, 120.2, 115.8 (d, *J*=21.3), 108.0, 105.7, 101.3, 100.5, 98.1, 69.7, 33.1, 14.3. The large coupling constant of about 250 Hz is not observed for the C-F ipso carbons. **^{19}F-NMR** (376 MHz, $CDCl_3$) δ -115.68 (d, *J*=52.4). **HR-MS** (ESI+) calc. for $C_{37}{}^{13}CH_{36}FN_2O_5^+$ 620.2636, found: 620.2675.

[1,1'-Biphenyl]-4-yl(4-(4-(4-fluorophenyl)-5-(2-methoxypyrimidin-4-yl)-1H-imidazol-1-yl)piperidin-1-yl)methanone (^{12}C-13a)

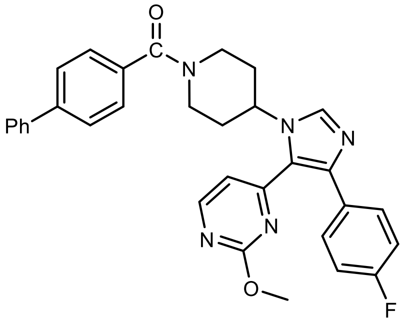

4-Biphenyl-neopentyl boronic ester (26.6 mg, 0.10 mmol, 1.0 equiv.) and Pd-SB242235 (**Pd-13**) (115 mg, 0.11 mmol, 1.10 equiv.) were subjected to general procedure D.2. Purification by flash column chromatography (EtOAc:heptane 1:1 to 0:1), yielded the product as a white solid (31.8 mg, 0.059 mmol, 59%).

^{1}H-NMR (400 MHz, $CDCl_3$) δ 8.36 (d, *J*=5.1, 1H), 7.82 (s, 1H), 7.65 (d, *J*=8.0, 2H), 7.60 (d, *J*=8.8, 2H), 7.52 (d, *J*=8.0, 2H), 7.46 (t, *J*=7.5, 2H), 7.39 (m, 3H), 7.03 (t, *J*=8.7, 2H), 6.79 (d, *J*=5.1, 1H), 5.05 (tt, *J*=12.0, 3.3, 1H), 4.06 (s, 3H), 2.77 – 3.27 (m, 2H), 2.16 – 2.40 (m, 2H), 1.80 – 2.04 (m, 2H), 1.46 – 1.77 (m, 2H). **^{13}C-NMR** (101 MHz, $CDCl_3$) δ 170.6, 165.8, 162.8 (d, *J* = 246.1 Hz), 160.0, 159.5, 143.7, 143.1, 140.3, 136.4, 134.3, 130.4 (d, *J*=8.1), 129.1, 128.0, 127.8, 127.5, 127.3, 124.2, 115.9 (d, *J*=5.4), 115.6, 55.1, 54.3, 29.8. **^{19}F-NMR** (376 MHz, $CDCl_3$) δ -113.70. **HR-MS** (ESI+) calc. for $C_{32}CH_{29}FN_5O_2^+$ 534.2300, found: 534.2340.

[1,1'-Biphenyl]-4-yl(4-(4-(4-fluorophenyl)-5-(2-methoxypyrimidin-4-yl)-1H-imidazol-1-yl)piperidin-1-yl)- ^{13}C-methanone (^{13}C-13a)

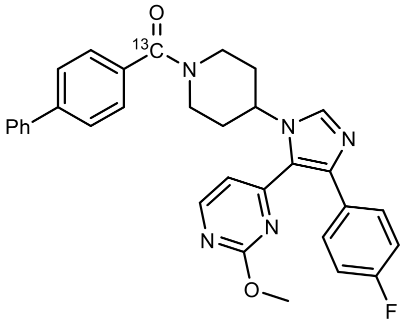

4-Biphenyl-neopentyl boronic ester (26.6 mg, 0.10 mmol, 1.0 equiv.) and Pd-SB242235 (**^{13}C-Pd-13**) (115 mg, 0.11 mmol, 1.10 equiv.) were subjected to general procedure D.2. Purification by flash column chromatography (50:50 to 0:100 EtOAc:heptane), yielded the product as a white-slight-yellow solid (40.1 mg, 0.073 mmol, 73%).

^{1}H-NMR (400 MHz, $CDCl_3$) δ 8.36 (d, *J*=5.1, 1H), 7.82 (s, 1H), 7.65 (d, *J*=7.7, 2H), 7.60 (dd, *J*=7.1, 1.5, 2H), 7.53 (m, 2H), 7.46 (m, 2H), 7.40 (m, 3H), 7.03 (m, 2H), 6.79 (d, *J*=5.1, 1H), 5.05 (tt, *J*=11.8, 3.5, 1H), 4.06 (s, 3H), 2.71 – 3.33 (m, 2H), 2.15 – 2.39 (m, 2H), 1.81 – 2.04 (m, 2H), 1.61 (m, 2H). **^{13}C-NMR** (101 MHz, $CDCl_3$) δ 170.6, 165.8, 162.8 (d, *J* = 247.8 Hz),160.0, 159.5, 143.6, 143.1, 140.3, 136.4, 134.6, 130.5 (d, *J*=8.1), 129.1, 128.1, 127.7 (d, *J*=2.2), 127.5 (d, *J*=4.4), 127.3, 124.2, 115.9 (d, *J*=5.1), 115.7, 55.1, 54.3, 33.8. **^{19}F-NMR** (376 MHz, $CDCl_3$) δ -113.68 (d, *J*=3.4). **HR-MS** (ESI+) calc. for $C_{31}{}^{13}CH_{29}FN_5O_2^+$ 535.2333, found: 535.2369.

[1,1'-Biphenyl]-4-yl(4-(4-(4-fluorophenyl)-5-(2-methoxypyrimidin-4-yl)-1*H*-imidazol-1-yl)piperidin-1-yl)-^{14}C-methanone (^{14}C-13a)

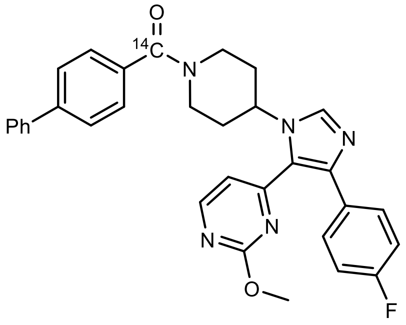

4-Biphenyl-neopentyl boronic ester (29.3 mg, 0.11 mmol, 1.0 equiv.) and Pd-SB242235 (**^{14}C-Pd-13**) (22.9 MBq, 127 mg, 0.121 mmol, 1.10 equiv.) were subjected to general procedure E. The title compound was prepared by preparative HPLC (60:40 to 75:25 MeCN:NH_3 2 v/v% in H_2O for 12 min, wavelength of 265nm, 20 mL/min). yielded the product as a brown solid in (54.6 mg, 15.9 MBq, SA: 156 or 163 MBq/mmol by MS, 86%, 69% RCY, >99% RCP).

^{1}H-NMR (400 MHz, $CDCl_3$) δ 8.37 (d, *J* = 5.1 Hz, 1H), 7.85 (s, 1H), 7.66 (d, *J* = 8.0 Hz, 2H), 7.60 (d, *J* = 7.3 Hz, 2H), 7.54 (m, 2H), 7.46 (m, 4H), 7.42 – 7.36 (m, 1H), 7.15 – 6.91 (m, 2H), 6.80 (d, *J* = 5.1 Hz, 1H), 4.07 (s, 4H), 3.33 – 2.68 (m, 2H), 2.29 (s, 2H). **^{13}C-NMR** (101 MHz, $CDCl_3$) δ 170.5, 165.7, 162.7 (d, *J* = 247.4 Hz), 159.9, 159.4, 143.5, 143.0, 140.2, 136.4, 130.4 (d, *J* = 8.0 Hz), 130.2 (d, *J* = 3.4 Hz), 129.0, 128.6 (d, *J* = 12.3 Hz), 128.0, 127.7, 127.4, 127.2, 115.8 (d, *J* = 10.2 Hz), 115.6, 55.0, 54.2. **LR-LCMS** (ESI+) calc. for $C_{31}{}^{14}CCH_{29}FN_5O_2^+$ 536.2332, found: 536.2.

[1,2,4]Triazolo[1,5-a]pyridin-6-yl(4-(4-(4-fluorophenyl)-5-(2-methoxypyrimidin-4-yl)-1*H*-imidazol-1-yl)piperidin-1-yl)-methanone (^{12}C-13s)

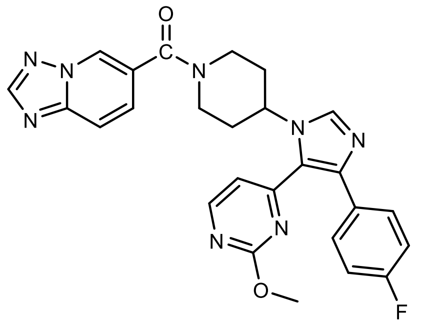

6-[1,2,4]Triazolo[1,5-a]pyridine boronic acid (16.3 mg, 0.10 mmol, 1.0 equiv.) and Pd-SB242235 (**Pd-13**) (115 mg, 0.11 mmol, 1.10 equiv.) were subjected to general procedure D.2. Purification by flash column chromatography (50:50 to 0:100 EtOAc:heptane), yielded the product as a brown solid (48.9 mg, 0.098 mmol, 98%).

^{1}H-NMR (400 MHz, $CDCl_3$) δ 8.80 (s, 1H), 8.39 (s, 1H), 8.32 (d, *J* = 5.2 Hz, 1H), 7.80 (d, *J* = 8.9 Hz, 2H), 7.59 (dd, *J* = 9.1, 1.7 Hz, 1H), 7.38 (dd, *J* = 8.6, 5.5 Hz, 2H), 6.99 (t, *J* = 8.8 Hz, 2H), 6.76 (d, *J* = 5.1 Hz, 1H), 5.04 (tt, *J* = 12.0, 3.8 Hz, 1H), 4.65 – 4.29 (m, 2H), 4.02 (s, 3H), 3.09 (s, 2H), 2.30 (d, *J* = 12.5 Hz, 2H), 1.99 (dt, *J* = 14.3, 6.3 Hz, 2H). **^{13}C-NMR** (101 MHz, $CDCl_3$) δ 166.3, 165.7, 162.7 (d, *J* = 247.7 Hz), 159.9, 159.3, 155.2, 150.6, 143.7, 136.3, 130.4 (d, *J* = 8.1 Hz), 130.2 (d, *J* = 3.3 Hz), 128.8, 128.5, 124.1, 122.6, 116.9, 115.8, 115.6, 55.0, 53.9. **^{19}F-NMR** (376 MHz, $CDCl_3$) δ -113.59 (td, *J* = 8.8, 4.6 Hz). **HR-MS** (ESI+) calc. for $C_{26}H_{24}FN_8O_2^+$ 499.2001, found: 499.2015

[1,2,4]Triazolo[1,5-a]pyridin-6-yl(4-(4-(4-fluorophenyl)-5-(2-methoxypyrimidin-4-yl)-1*H*-imidazol-1-yl)piperidin-1-yl)-^{13}C-methanone (^{13}C-13s)

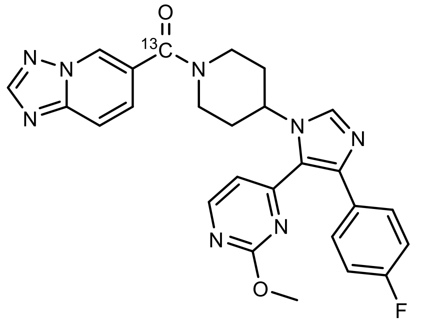

6-[1,2,4]Triazolo[1,5-a]pyridine boronic acid (16.3 mg, 0.10 mmol, 1.0 equiv.) and Pd-SB242235 (**^{13}C-Pd-13**) (115 mg, 0.11 mmol, 1.10 equiv.) were subjected to general procedure D.2. Purification by flash column chromatography (50:50 to 0:100 EtOAc:heptane), yielded the product as a brown solid (48.9 mg, 0.098 mmol, 98%).

^{1}H-NMR (400 MHz, $CDCl_3$) δ 8.80 (dt, *J* = 3.1, 1.3 Hz, 1H), 8.40 (s, 1H), 8.33 (d, *J* = 5.1 Hz, 1H), 7.80 (d, *J* = 9.9 Hz, 2H), 7.59 (ddd, *J* = 9.1, 3.2, 1.7 Hz, 1H), 7.39 (dd, *J* = 8.8, 5.4 Hz, 2H), 7.00 (t, *J* = 8.7 Hz, 2H), 6.76 (d, *J* = 5.2 Hz, 1H), 5.04 (ddt, *J* = 12.2, 7.2, 3.7 Hz, 1H), 4.02 (s, 3H), 3.09 (s, 2H), 2.30 (d, *J* = 12.6 Hz, 2H), 1.96 (m, 1H). **^{13}C-NMR** (101 MHz, $CDCl_3$) δ 166.3, 165.7, 162.7 (d, *J* = 247.6 Hz), 159.9, 159.3, 155.2, 150.6, 143.7, 136.3, 130.4 (d, *J* = 8.1 Hz), 130.2 (d, *J* = 3.3 Hz), 128.8, 128.5 (d, *J* = 4.4 Hz), 124.1, 122.6 (d, *J* = 69.5 Hz), 116.9 (d, *J* = 4.3 Hz), 115.8, 115.6, 55.0, 53.9. **^{19}F-NMR** (376 MHz, $CDCl_3$) δ -113.56 (m). **HR-MS** (ESI+) calc. for $C_{25}{}^{13}CH_{24}FN_8O_2^+$ 500.2034, found: 500.2048

[1,2,4]Triazolo[1,5-a]pyridin-6-yl(4-(4-(4-fluorophenyl)-5-(2-methoxypyrimidin-4-yl)-1*H*-imidazol-1-yl)piperidin-1-yl)-^{14}C-methanone (^{14}C-13s)

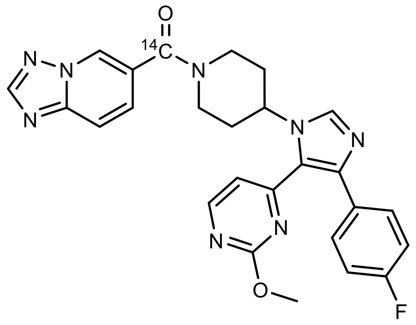

1-Methyl-6-(4,4,5,5-tetramethyl-1,3,2-dioxaborolan-2-yl)indolin-2-one (17.0 mg, 0.104 mmol, 1.0 equiv.) and Pd-SB242235 (**^{14}C-Pd-13**) (21.7 MBq, 120 mg, 0.114 mmol, 1.10 equiv.) were subjected to general procedure E, at a 0.104 mmol scale. The title compound was prepared by preparative HPLC (23:77 to 34:66 MeCN:NH_3 2 v/v% in H_2O for 18 min, wavelength of 265nm, 20 mL/min). yielded the product as a brown solid in (52.0 mg, 17.8 MBq, SA: 171 MBq/mmol or 220 MBq/mmol by MS, 98%, 82% RCY, >99% RCP).

^{1}H-NMR (400 MHz, $CDCl_3$) δ 8.81 (dd, *J* = 1.7, 0.9 Hz, 1H), 8.40 (s, 1H), 8.33 (dd, *J* = 5.1, 0.8 Hz, 1H), 7.84 (s, 1H), 7.81 (m, 1H), 7.59 (dd, *J* = 9.2, 1.7 Hz, 1H), 7.39 (m, 2H), 6.99 (t, *J* = 8.5 Hz, 2H), 6.75 (dd, *J* = 5.2, 0.8 Hz, 1H), 5.04 (tt, *J* = 12.1, 3.8 Hz, 1H), 4.02 (d, *J* = 0.9 Hz, 3H), 3.09 (s, 2H), 2.30 (d, *J* = 12.5 Hz, 2H), 2.04 (s, 1H), 1.96 (m, 2H). **^{13}C-NMR** (101 MHz, $CDCl_3$) δ 166.3, 165.7, 162.7 (d, *J* = 247.6 Hz), 159.8, 159.3, 155.2, 143.5, 136.4, 130.4 (d, *J* = 8.1 Hz), 130.0 (d, *J* = 3.3 Hz), 129.4, 128.8, 128.5, 127.1, 124.2, 122.6, 116.9, 115.8 (d, *J* = 1.8 Hz), 115.6, 55.0, 54.0, 33.6. **LR-LCMS** (ESI+) calc. for $C_{25}{}^{14}CH_{24}FN_8O_2^+$ 501.2034, found: 501.2

5 ^{14}C-analysis

trans-Chloro(2-chloro-11-(piperazin-1-yl)dibenzo[*b,f*][1,4]oxazepine)-^{14}C-carbonyl)bis(triphenylphosphine) palladium(II) (^{14}C-Pd-10)

ISOPAT:

IsoPat²

EN26327-21B

Expected derivatives: 8

Calculate

Labelled atoms: 1

Atom%: 53.8

	Unlabeled compound Abundance	Analyte Abundance
M1+0	1.08E+06	1.18E+06
M1+1	2.51E+06	1.78E+06
M1+2	3.82E+06	4.48E+06
M1+3	2.90E+06	2.82E+06
M1+4	3.24E+06	4.69E+06
M1+5	1.50E+06	2.15E+06
M1+6	2.81E+06	3.15E+06
M1+7	1170660.00	1106198
M1+8	682455.00	891033
M1+9	257703.00	377498
M1+10	0.00	115035
M1+11	0	0
M1+12	0	0
M1+13	0	0
M1+14	0	0
M1+15	0	0
M1+16	0	0
M1+17	0	0
M1+18	0	0
M1+19	0	0
Sum:		22733723

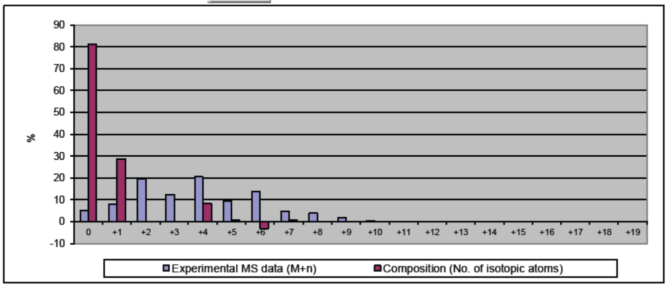

Results	Relative amounts [%]
unlabeled	81.1
1-label	28.7
2-label	0.0
3-label	0.0
4-label	8.3
5-label	0.9
6-label	-3.1
…	0.8
…	

SA	0.599	KBq/nmol
SA	0.016	Ci/mmol
	0.599	MBq/umol
	0.599	GBq/mmol
	0.599	TBq/mol

LCMS:

7:(Time: 1.70) Combine (368:390-(331:342+418:430)) 1:MS ES+ 1.9e+006

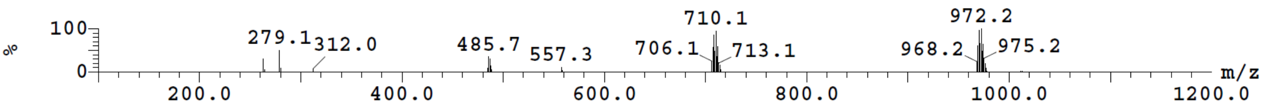

trans-Chloro(4-(4-(4-fluorophenyl)-1-(piperidin-4-yl)-1H-imidazol-5-yl)-2-methoxy-pyrimidine)-^{14}C-carbonyl)bis(triphenylphosphine) palladium(II) (^{14}C-Pd-13)

ISOPAT:

IsoPat²

EN26327-30

Expected derivatives: 8

Calculate

Labelled atoms: 1

Atom%: 22.4

	Unlabeled compound Abundance	Analyte Abundance
M1+0	2.56E+04	7.68E+05
M1+1	1.12E+05	2.10E+06
M1+2	1.35E+05	2.24E+06
M1+3	7.43E+04	1.31E+06
M1+4	6.73E+04	1.96E+06
M1+5	3.97E+04	7.03E+05
M1+6	5.06E+04	1.31E+06
M1+7	25479.00	338089
M1+8	0.00	97762
M1+9	0.00	0
M1+10	0.00	0
M1+11	0	0
M1+12	0	0
M1+13	0	0
M1+14	0	0
M1+15	0	0
M1+16	0	0
M1+17	0	0
M1+18	0	0
M1+19	0	0
Sum:		10818705

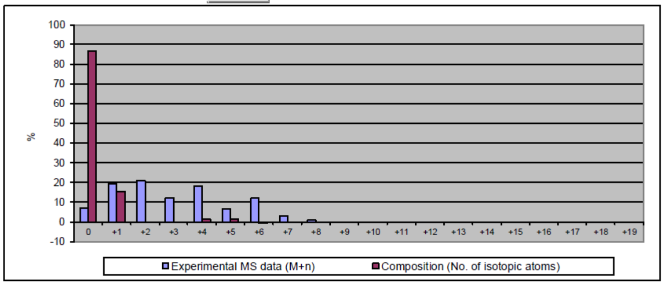

Results	Relative amounts [%]
unlabeled	86.5
1-label	15.4
2-label	0.0
3-label	0.0
4-label	1.2
5-label	1.5
6-label	-0.6
…	-0.3
…	

SA	0.346	KBq/nmol
SA	0.009	Ci/mmol
	0.346	MBq/umol
	0.346	GBq/mmol
	0.346	TBq/mol

LCMS:

5:(Time: 1.55) Combine (334:356-(308:319+383:394)) 1:MS ES+ 1.6e+006

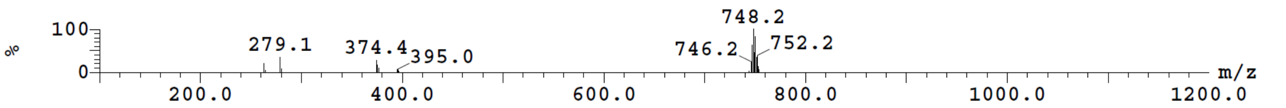

[1,1'-Biphenyl]-4-yl(4-(2-chlorodibenzo[*b,f*][1,4]oxazepin-11-yl)piperazin-1-yl)-^{14}C-methanone (^{14}C-10a)

pH10:

Chromatogram: ^{14}C

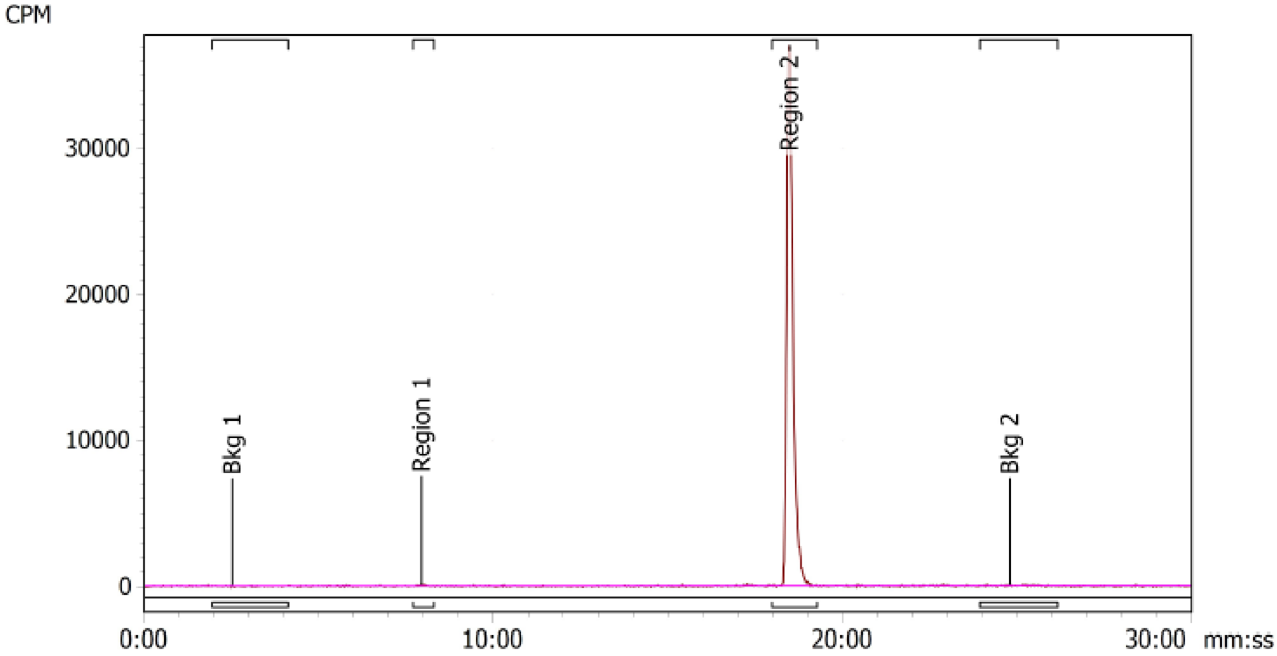

Regions: ^{14}C Detector: ß-RAM

Name	Start (mm:ss)	End (mm:ss)	Retention (mm:ss)	Area (CPM)	%ROI (%)	%Total (%)
Bkg 1	1:57	4:08	2:32			
Region 1	7:42	8:18	7:57	980	0,23	0,23
Region 2	17:59	19:17	18:29	429333	99,77	102,78
Bkg 2	23:56	26:10	24:48			
2 Peaks				430313	100,00	103,02

Total Area: 417715 CPM
Average Background: 60 CPM

Sample Details: SN1147076331; pH10
1 kBq/uL

Injection Volume: 10 µL

pH3:

Chromatogram: ^{14}C

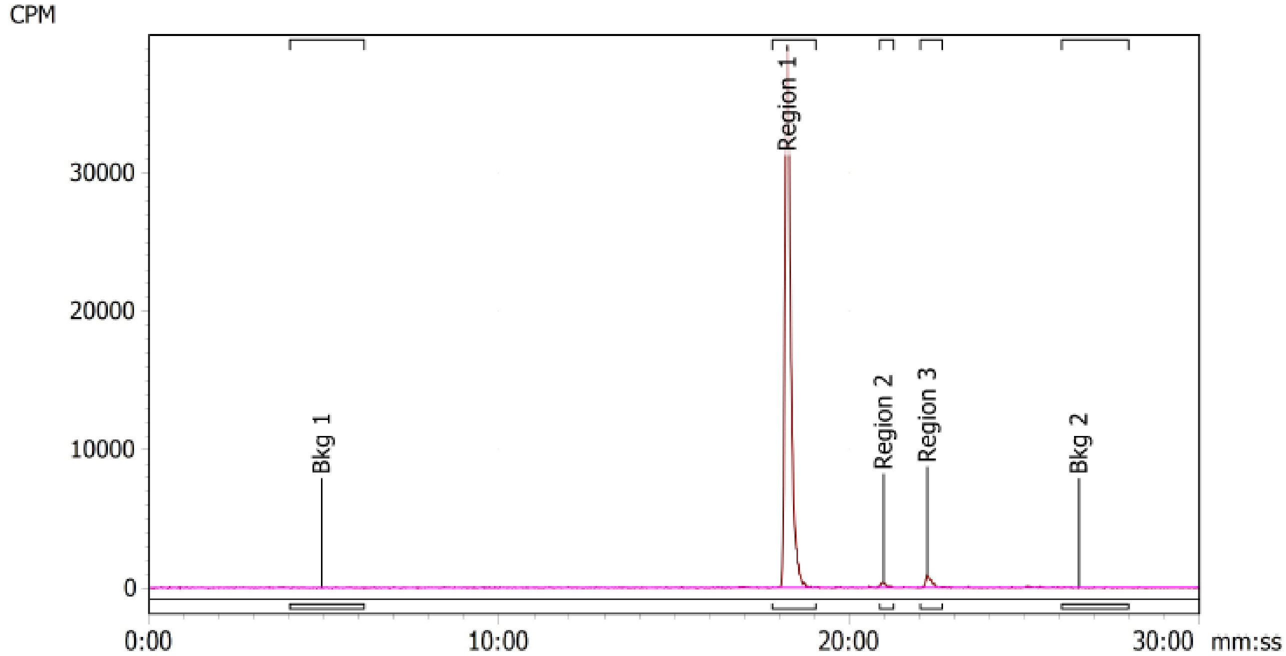

Regions: ^{14}C Detector: β-RAM

Name	Start (mm:ss)	End (mm:ss)	Retention (mm:ss)	Area (CPM)	%ROI (%)	%Total (%)
Bkg 1	4:01	6:09	4:56			
Region 1	17:48	19:03	18:14	423033	97,00	95,85
Region 2	20:52	21:16	20:59	4018	0,92	0,91
Region 3	22:02	22:39	22:14	9045	2,07	2,05
Bkg 2	26:04	27:59	26:34			
3 Peaks				436096	100,00	98,81

Total Area: 441346 CPM
Average Background: 48 CPM

Sample Details: SN1147076331; pH3
1 kBq/uL

Injection Volume: 10 μL

ISOPAT:

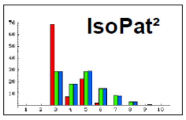

EN26327-34

Expected derivatives: 8

Calculate

Labelled atoms: 1
Atom%: 1.9

	Unlabeled compound Abundance	Analyte Abundance
M1+0	1.60E+07	3.27E+07
M1+1	4.91E+06	9.85E+06
M1+2	5.88E+06	1.26E+07
M1+3	1.96E+06	3.77E+06
M1+4	2.28E+05	1.12E+06
M1+5	0.00E+00	2.78E+05
M1+6	0.00E+00	0.00E+00
M1+7	0.00	0
M1+8	0.00	0
M1+9	0.00	0
M1+10	0.00	0
M1+11	0	0
M1+12	0	0
M1+13	0	0
M1+14	0	0
M1+15	0	0
M1+16	0	0
M1+17	0	0
M1+18	0	0
M1+19	0	0

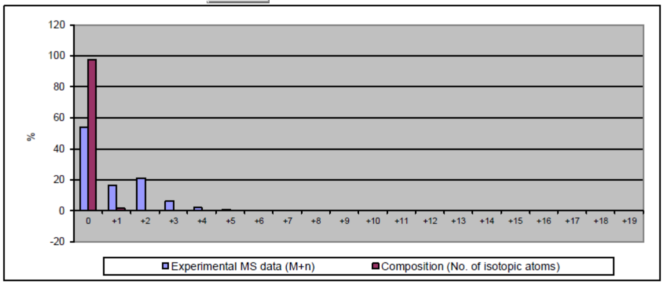

Results

Relative amounts [%]	
unlabeled	97.6
1-label	1.4
2-label	0.0
3-label	0.0
4-label	0.2
5-label	-0.1
6-label	0.0
…	0.0
…	

SA	0.032	KBq/nmol
SA	9E-04	Ci/mmol
	0.032	MBq/umol
	0.032	GBq/mmol
	0.032	TBq/mol

LCMS:

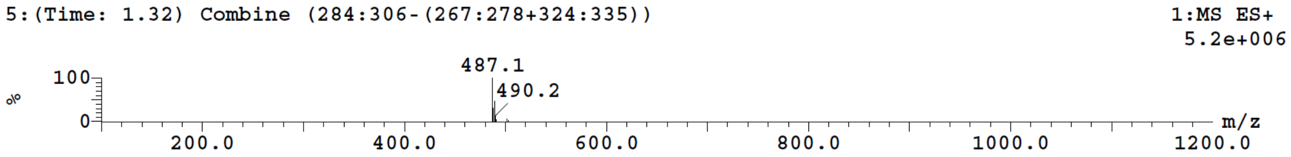

6-(4-(2-Chlorodibenzo[*b,f*][1,4]oxazepin-11-yl)piperazine-1-^{14}C-carbonyl)-1-methylindolin-2-one (^{14}C-10p)

pH10:

Chromatogram: ^{14}C

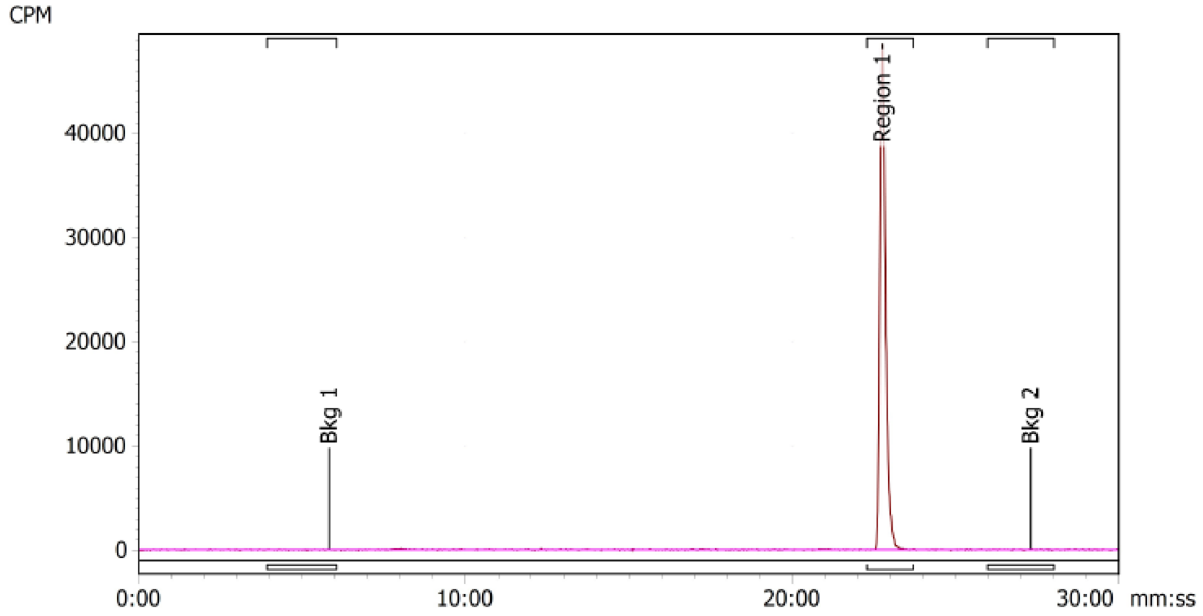

Regions: ^{14}C Detector: ß-RAM

Name	Start (mm:ss)	End (mm:ss)	Retention (mm:ss)	Area (CPM)	%ROI (%)	%Total (%)
Bkg 1	3:55	6:03	5:49			
Region 1	22:17	23:42	22:46	538068	100,00	98,51
Bkg 2	25:59	28:01	27:18			
1 Peak				538068	100,00	98,51

Total Area: 546221 CPM
Average Background: 48 CPM

Sample Details: SN1147076076; pH10
1.2 kBq/uL

Injection Volume:9 µL

pH3:

Chromatogram: ^{14}C

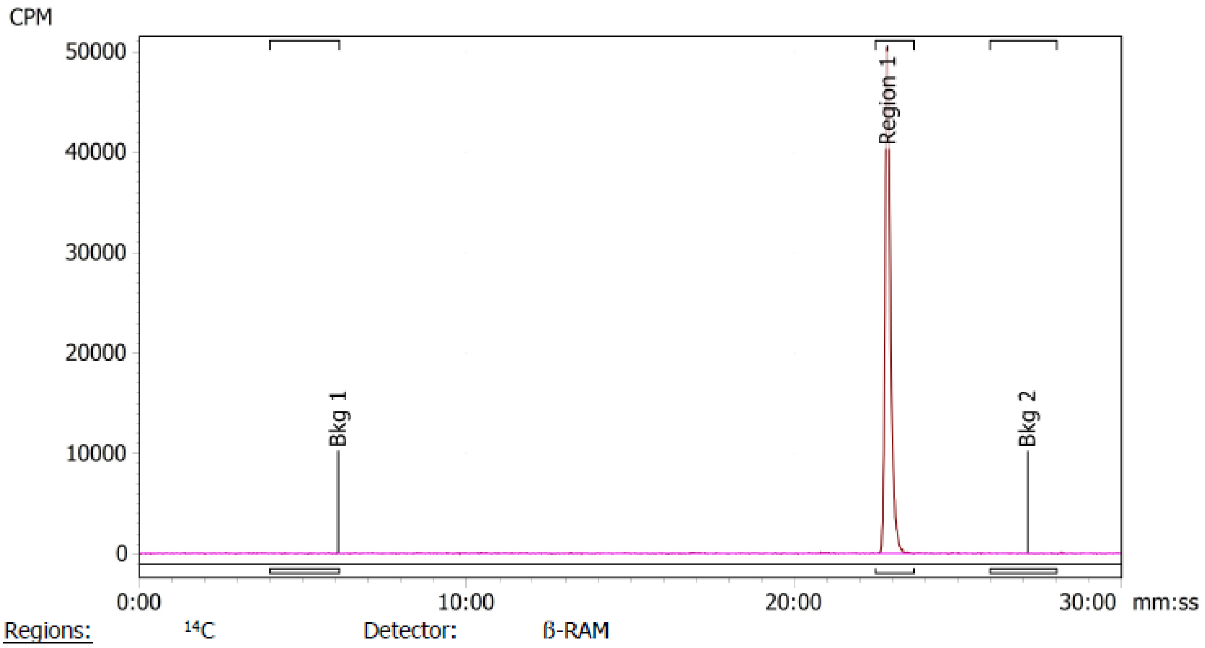

Regions: ^{14}C Detector: ß-RAM

Name	Start (mm:ss)	End (mm:ss)	Retention (mm:ss)	Area (CPM)	%ROI (%)	%Total (%)
Bkg 1	3:59	6:06	6:04			
Region 1	22:29	23:39	22:51	551462	100,00	98,51
Bkg 2	25:59	28:01	27:08			
1 Peak				551462	100,00	98,51

Total Area: 559809 CPM
Average Background: 55 CPM

Sample Details: SN1147076076; pH3
1.2 kBq/uL

Injection Volume:9 µL

ISOPAT:

IsoPat²

EN26327-27

Expected derivatives: 8

Calculate

Labelled atoms: 1
Atom%: 17.2

	Unlabeled compound Abundance	Analyte Abundance
M1+0	1.22E+07	2.21E+07
M1+1	3.07E+06	7.75E+06
M1+2	4.25E+06	1.28E+07
M1+3	1.26E+06	3.30E+06
M1+4	0.00E+00	1.08E+06
M1+5	0.00E+00	0.00E+00
M1+6	0.00E+00	0.00E+00
M1+7	0.00	0
M1+8	0.00	0
M1+9	0.00	0
M1+10	0.00	0
M1+11	0	0
M1+12	0	0
M1+13	0	0
M1+14	0	0
M1+15	0	0
M1+16	0	0
M1+17	0	0
M1+18	0	0
M1+19	0	0

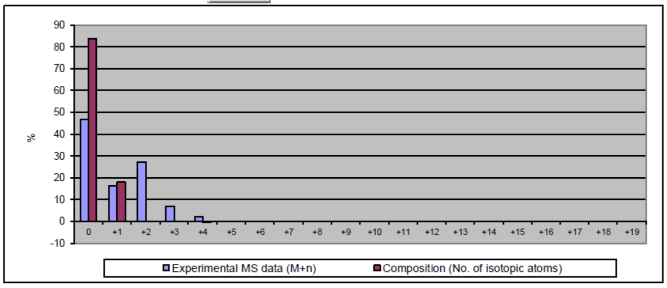

Results

Relative amounts [%]

unlabeled	83.8
1-label	18.1
2-label	0.0
3-label	0.0
4-label	-0.3
5-label	0.1
6-label	0.0
...	0.0
...	

SA	0.406	KBq/nmol
SA	0.011	Ci/mmol
	0.406	MBq/umol
	0.406	GBq/mmol
	0.406	TBq/mol

LCMS:

Peak ID	Time	Mass Found
5	1.61	Not Found

5:(Time: 1.63) Combine (352:374-(331:342+408:419)) 1:MS ES+ 4.5e+006

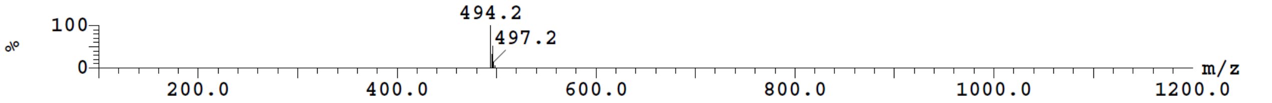

[1,1'-Biphenyl]-4-yl(4-(4-(4-fluorophenyl)-5-(2-methoxypyrimidin-4-yl)-1*H*-imidazol-1-yl)piperidin-1-yl)-^{14}C-methanone (^{14}C-13a)

pH10:

Chromatogram: ^{14}C

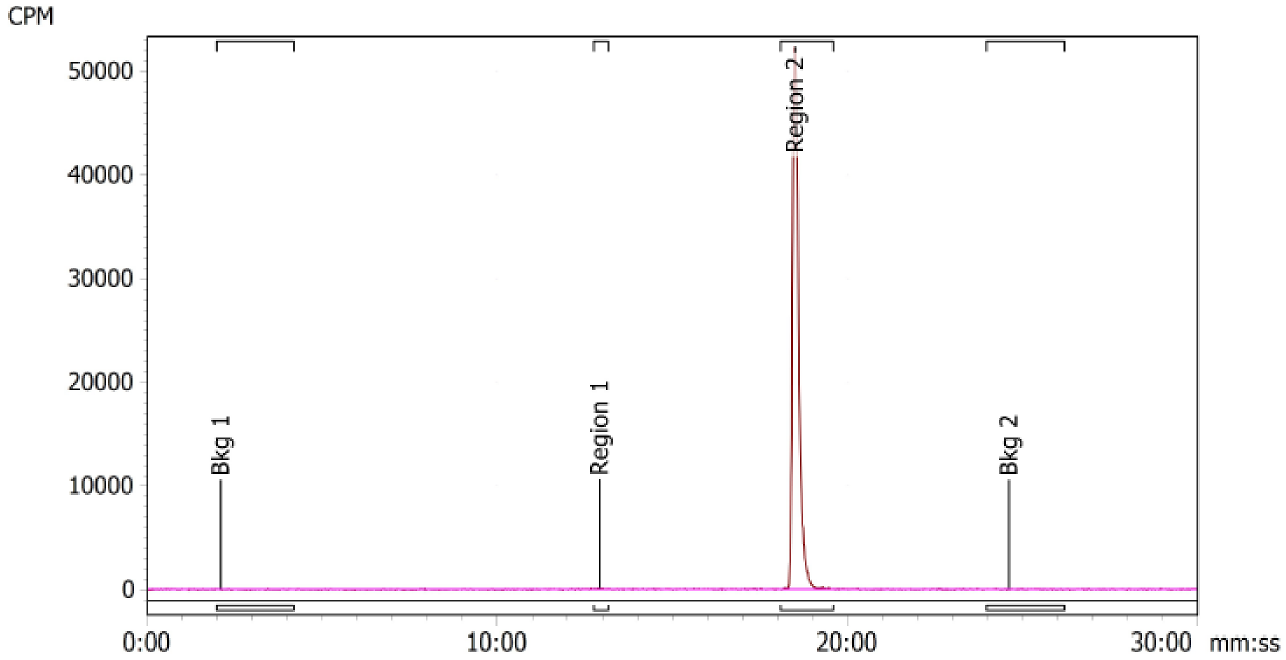

Regions: ^{14}C Detector: ß-RAM

Name	Start (mm:ss)	End (mm:ss)	Retention (mm:ss)	Area (CPM)	%ROI (%)	%Total (%)
Bkg 1	1:59	4:11	2:06			
Region 1	12:45	13:10	12:55	1328	0,22	0,22
Region 2	18:05	19:36	18:30	598079	99,78	99,37
Bkg 2	23:58	26:12	24:37			
2 Peaks				599407	100,00	99,60

Total Area: 601844 CPM
Average Background: 51 CPM

Sample Details: SN1147076359; pH10
1 kBq/uL

Injection Volume: 10 µL

pH3:

Chromatogram: ^{14}C

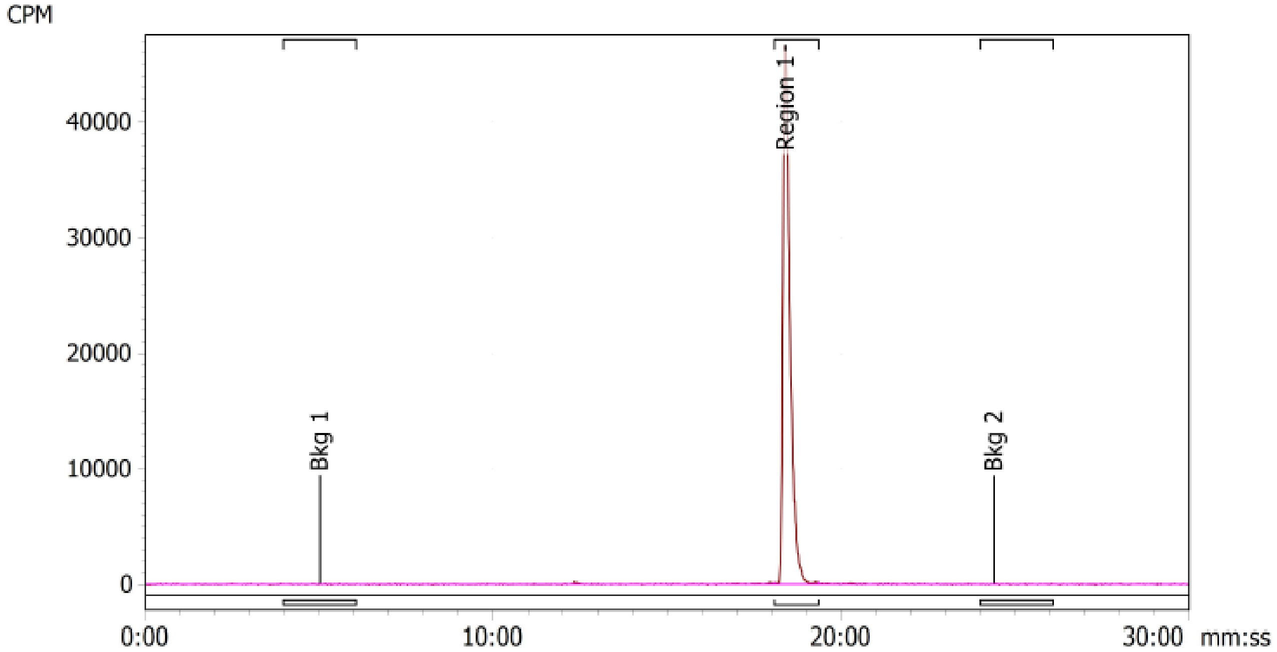

Regions: ^{14}C Detector: ß-RAM

Name	Start (mm:ss)	End (mm:ss)	Retention (mm:ss)	Area (CPM)	%ROI (%)	%Total (%)
Bkg 1	3:58	6:04	5:01			
Region 1	18:05	19:21	18:24	600200	100,00	98,91
Bkg 2	24:00	26:06	24:24			
1 Peak				600200	100,00	98,91

Total Area: 606825 CPM
Average Background: 46 CPM

Sample Details: SN1147076359; pH3
1 kBq/uL

Injection Volume: 10 µL

ISOPAT:

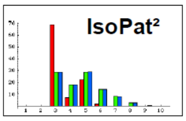

EN26327-35

Expected derivatives: 8

Calculate

Labelled atoms: 1
Atom%: 7.1

	Unlabeled compound Abundance	Analyte Abundance
M1+0	1.43E+07	2.04E+07
M1+1	3.16E+06	6.20E+06
M1+2	8.10E+05	2.74E+06
M1+3	5.22E+04	5.74E+05
M1+4	0.00E+00	0.00E+00
M1+5	0.00E+00	0.00E+00
M1+6	0.00E+00	0.00E+00
M1+7	0.00	0
M1+8	0.00	0
M1+9	0.00	0
M1+10	0.00	0
M1+11	0	0
M1+12	0	0
M1+13	0	0
M1+14	0	0
M1+15	0	0
M1+16	0	0
M1+17	0	0
M1+18	0	0
M1+19	0	0

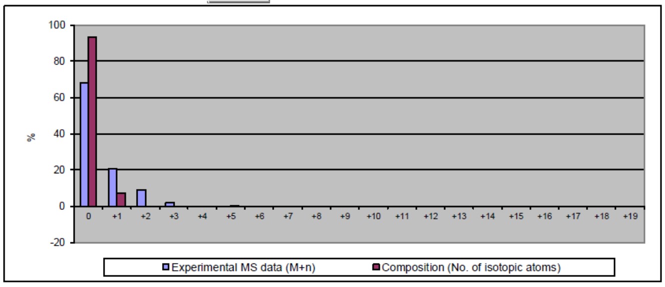

Results

Relative amounts [%]	
unlabeled	93.2
1-label	7.1
2-label	0.0
3-label	0.0
4-label	0.0
5-label	0.0
6-label	0.0
...	0.0
...	

SA 0.163 KBq/nmol
SA 0.004 Ci/mmol

0.163 MBq/umol
0.163 GBq/mmol
0.163 TBq/mol

LCMS:

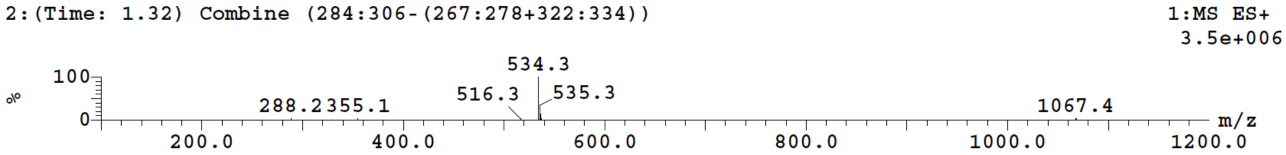

[1,2,4]Triazolo[1,5-a]pyridin-6-yl(4-(4-(4-fluorophenyl)-5-(2-methoxypyrimidin-4-yl)-1*H*-imidazol-1-yl)piperidin-1-yl)-^{14}C-methanone (^{14}C-13s)

pH10:

Chromatogram: ^{3}H

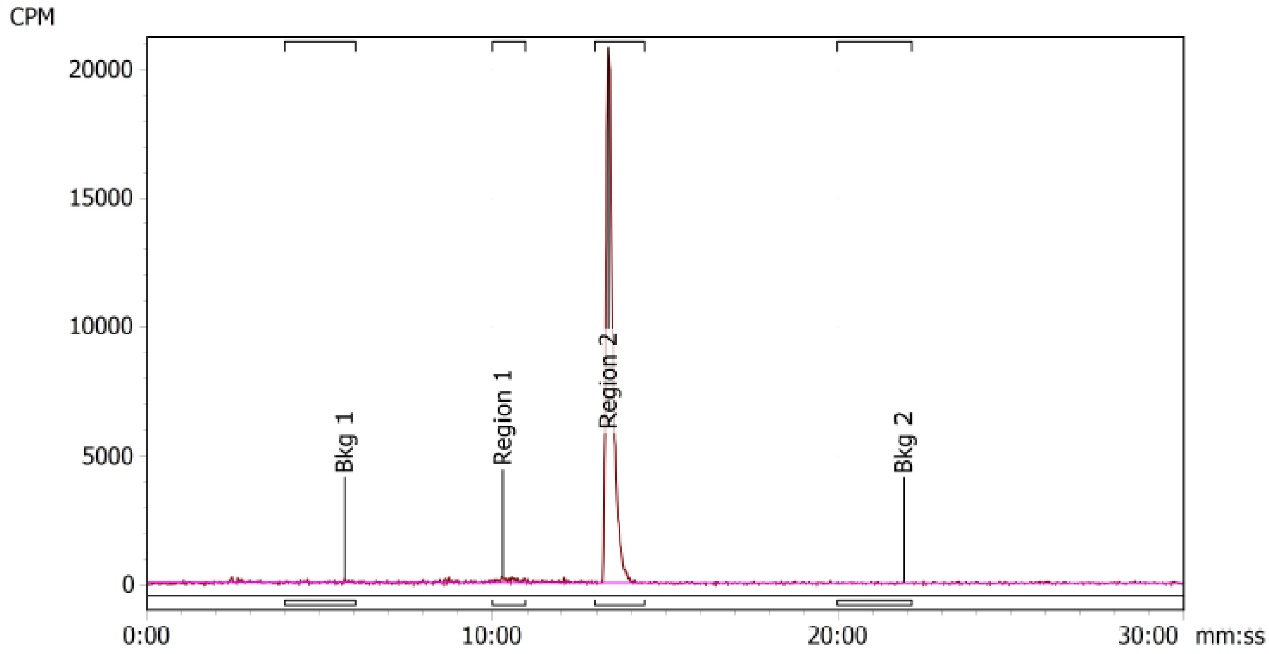

Regions: ^{3}H Detector: ß-RAM

Name	Start (mm:ss)	End (mm:ss)	Retention (mm:ss)	Area (CPM)	%ROI (%)	%Total (%)
Bkg 1	3:59	6:02	5:43			
Region 1	10:00	10:57	10:18	3876	1,40	1,41
Region 2	12:58	14:24	13:21	272578	98,60	98,82
Bkg 2	19:58	22:08	21:54			
2 Peaks				276454	100,00	100,23

Total Area: 275824 CPM
Average Background: 70 CPM

pH3:

Chromatogram: ^3H

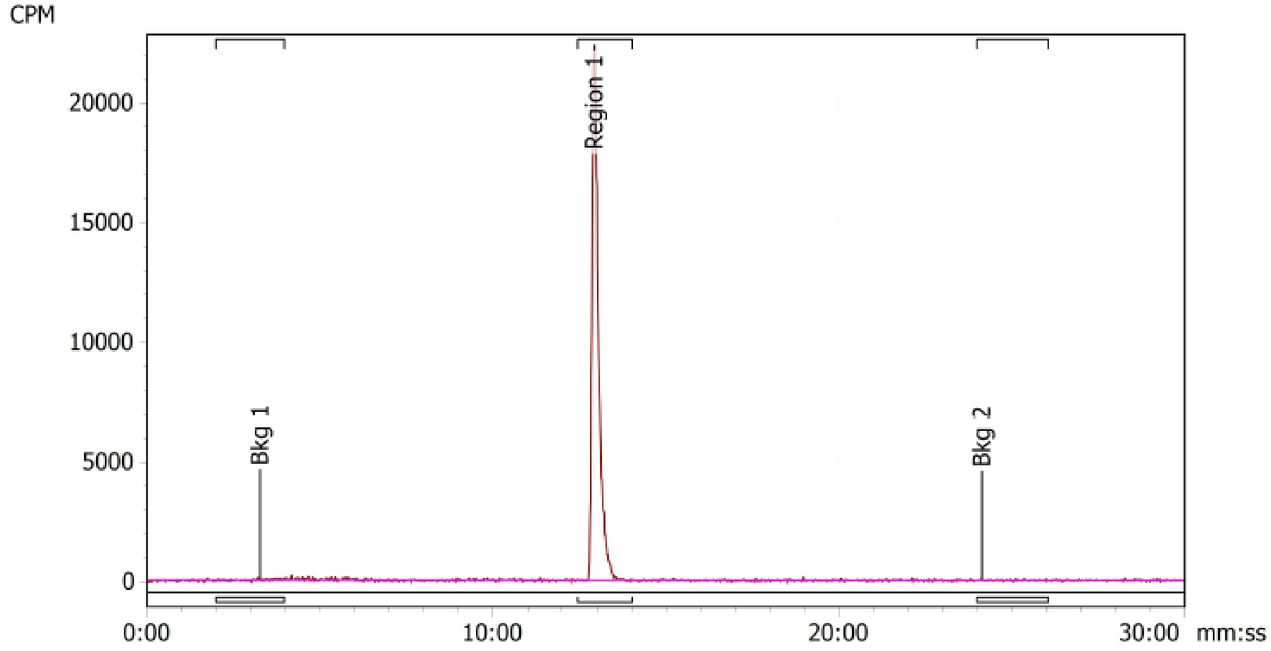

Regions: ^3H Detector: ß-RAM

Name	Start (mm:ss)	End (mm:ss)	Retention (mm:ss)	Area (CPM)	%ROI (%)	%Total (%)
Bkg 1	1:59	3:58	3:16			
Region 1	12:27	14:01	12:56	274933	100,00	101,47
Bkg 2	23:59	26:03	24:08			
1 Peak				274933	100,00	101,47

Total Area: 270961 CPM
Average Background: 63 CPM

Sample Details: SN1147076600; pH3
0.98 kBq/uL

Injection Volume: 10 µL

ISOPAT:

IsoPat²

EN26327-36

Expected derivatives: 8

Labelled atoms: 1
Atom%: 9.6

	Unlabeled compound Abundance	Analyte Abundance
M1+0	2.29E+07	3.03E+07
M1+1	4.34E+06	8.87E+06
M1+2	1.04E+06	4.62E+06
M1+3	1.00E+05	1.05E+06
M1+4	0.00E+00	0.00E+00
M1+5	0.00E+00	0.00E+00
M1+6	0.00E+00	0.00E+00
M1+7	0.00	0
M1+8	0.00	0
M1+9	0.00	0
M1+10	0.00	0
M1+11	0	0
M1+12	0	0
M1+13	0	0
M1+14	0	0
M1+15	0	0
M1+16	0	0
M1+17	0	0
M1+18	0	0
M1+19	0	0

Calculate

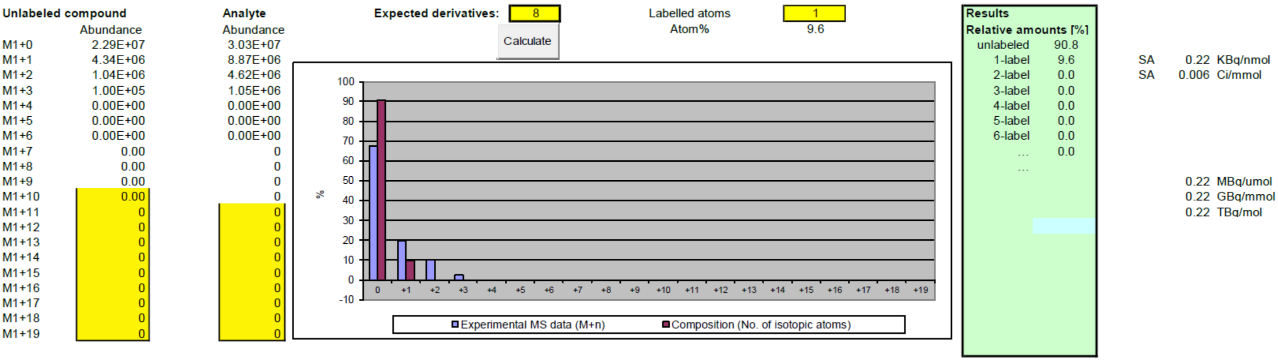

Results

Relative amounts [%]	
unlabeled	90.8
1-label	9.6
2-label	0.0
3-label	0.0
4-label	0.0
5-label	0.0
6-label	0.0
...	0.0
...	

SA 0.22 KBq/nmol
SA 0.006 Ci/mmol

0.22 MBq/umol
0.22 GBq/mmol
0.22 TBq/mol

LCMS:

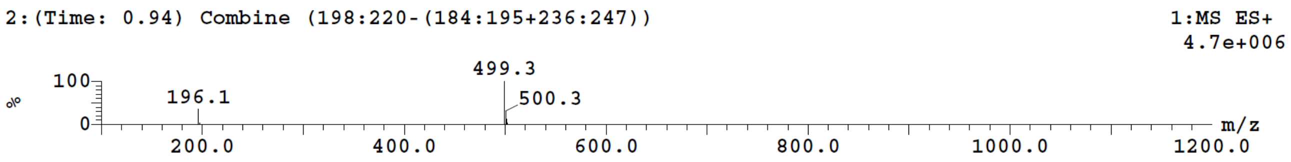

2:(Time: 0.94) Combine (198:220-(184:195+236:247))
1:MS ES+
4.7e+006
100
%
0
196.1
499.3
500.3
200.0
400.0
600.0
800.0
1000.0
1200.0
m/z

6 Crystallographic appendix

6.1 Data collection

Data were collected on an XtaLAB Synergy-S single-crystal X-ray diffractometer. The instrument features a Hybrid Pixel Array detector (HyPix-Arc 100), a PhotonJet microfocus sealed tube source (Mo Kα radiation), and is operated with CrysAlisPro. The measurements were performed at ambient temperature on a single crystal coated with Paratone N.

6.2 Structure solution and refinement

Absorption correction was performed using the SCALE3 ABSPACK multi-scan method. The space group assignment was based upon systematic absences, E statistics, and successful refinement of the structures. The structures were solved by intrinsic phasing using SHELXT[8] and were refined against all data using SHELXL[9] in conjunction with Olex2 v1.5.[10-11] Hydrogen atoms were calculated in ideal positions by placing them in initial, calculated positions and refining them using a riding model with methylene and aromatic C—H distances of 0.97 and 0.93 Å, and $U_{iso}(H) = 1.2\ U_{eq}(C)$. All non-hydrogen atoms were refined with anisotropic displacement parameters. Full-matrix least-squares refinements were carried out by minimising $\Sigma w(F^2_o - F^2_c)^2$ with the SHELXL weighting scheme.[9] Neutral atom scattering factors for all atoms and anomalous dispersion corrections for the non-hydrogen atoms were taken from International Tables for Crystallography.[10-11] Images of the crystal structure were generated with Olex2.[10-11]

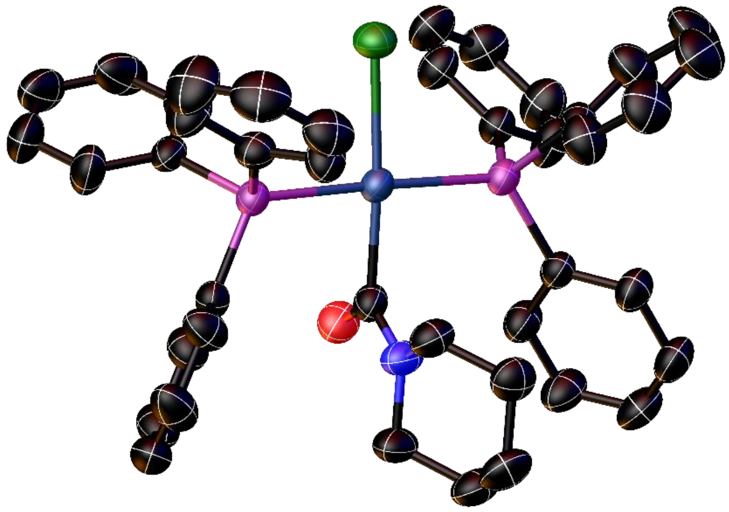

Supplementary Figure 1 Molecular structure of **Pd-5** with thermal ellipsoids displayed at the 50% probability level. Hydrogen atoms and co-crystallised water are omitted for clarity. Color code: Pd (blue), P (pink), C (black), N (blue), O (red).

Supplementary Table 2 Crystal data, data collection, and structure refinement for **Pd-5** (CCDC: 2534115)

**Molecular Formula**		$C_{42}H_{40}ClNOP_2Pd$ x ⅓ H_2O
**Formula Weight**	[g mol^{-1}]	778.61 (excluding co-crystallised water)
**Crystal Dimensions**	[mm^3]	0.21 × 0.15 × 0.045
**Crystal Habit**		clear colourless plate
**Crystal System**		monoclinic
**Space Group**		*Cc*
**Unit Cell Dimensions**	[Å / °]	a = 12.3977(2) α = 90<br>b = 27.6435(5) β = 100.564(2)<br>c = 11.0830(2) γ = 90
**Volume**	[Å^3]	3733.94(12)
**Z**		4
**Density (calculated)**	[g cm^{-3}]	1.396
**Absorption Coefficient μ**	[mm^{-1}]	0.689
**F(000)**	[e^{-1}]	1613
**Temperature**	[K]	299.0(2)
**Radiation Source**		Mo Kα
**Wavelength**	[Å]	0.71073
**Number of Frames**		1438
**Exposure Time**	[h]	3.46
**Θ Range**	[°]	1.826 – 32.636
**Index Ranges**		-18 ≤ h ≤ 18, -40 ≤ k ≤ 40, -16 ≤ l ≤ 16
**Reflections Collected**		30840
**Independent Reflections**		10297 [*R*(int) = 0.0349]
**Coverage to Θ**	[% / °]	99.9 / 25.242
**Max. and min. Transmission**		1.0000, 0.55838
**Data / Restraints / Parameters**		10297/2/445
**Goodness-of-fit on F^2**		1.088
**Δ/σ_{max}**		0.000
**Final *R* indices**	8403 data; $I>2\sigma(I)$<br>all data	R_1 = 0.0516, wR_2 = 0.1270<br>R_1 = 0.0646, wR_2 = 0.1360
**Largest Diff. Peak/Hole**	[eÅ^{-3}]	0.627 / -0.612
**R.M.S. Deviation from Mean**	[eÅ^{-3}]	0.061

7 References

[1] C. C. Gruber, G. Oberdorfer, C. V. Voss, J. M. Kremsner, C. O. Kappe, W. Kroutil, *J. Org. Chem.* 2007, **72**, 5778–5783.

[2] M. Aresta, P. Giannoccaro, I. Tommasi, A. Dibenedetto, A. M. M. Lanfredi, F. Ugozzoli, *Organometallics* 2000, **19**, 3879–3889.

[3] C. S. Elmore, D. J. Schenk, R. Arent, L. Kingston, *J. Labelled Comp. Radiopharm.* 2014, **57**, 645–651.

[4] S. J. Ton, A. K. Ravn, D. V. Hoffmann, C. S. Day, L. Kingston, C. S. Elmore, T. Skrydstrup, *JACS Au* 2023, **3**, 756–761.

[5] A. Latorre, J. A. Sáez, S. Rodríguez, F. V. González, *Tetrahedron* 2014, **70**, 97–102.

[6] A. G. Sams, G. K. Mikkelsen, M. Larsen, M. Langgård, M. E. Howells, T. J. Schrøder, L. T. Brennum, L. Torup, E. B. Jørgensen, C. Bundgaard, M. Kreilgård, B. Bang-Andersen, *J. Med. Chem.* 2011, **54**, 751–764.

[7] C. Zhang, Y. T. Liao, X. G. Chen, J. X. Xu, Y. Yu, P. M. Tang, Q. Gao, J. B. Zhao,Y. P. Li, X. F. Cheng, G. Z. Zhu, F. Ye, Y. Li, J. Ni, P. K. Yan, (Xizang Haisco Pharmaceutical CO., LTD.) Heterocyclic derivative, and composition and pharmaceutical use thereof, WO2023016518, (2023).

[8] G. M. Sheldrick, *Acta Crystallogr., A, Found. Crystallogr.* 2015, **71**, 3–8 .

[9] G. M. Sheldrick, *Acta Crystallogr., Sect. C: Struct. Chem.* 2015, **71**, 3–8.

[10] O. V. Dolomanov, L. J. Bourhis, R. J. Gildea, J. A. K. Howard, H. Puschmann, *Journal of Applied Crystallography* 2009, **42**, 339–341.

[11] A. J. C. Wilson (Ed.), *International Tables for Crystallography Volume C: Mathematical, Physical and Chemical Tables*, Kluwer Academic Publishers, Dordrecht/Boston/London, 1992.

8 NMR spectra of compounds

N-Boc-2-chloro-phenothiazine (Cl1i)

^{1}H-NMR

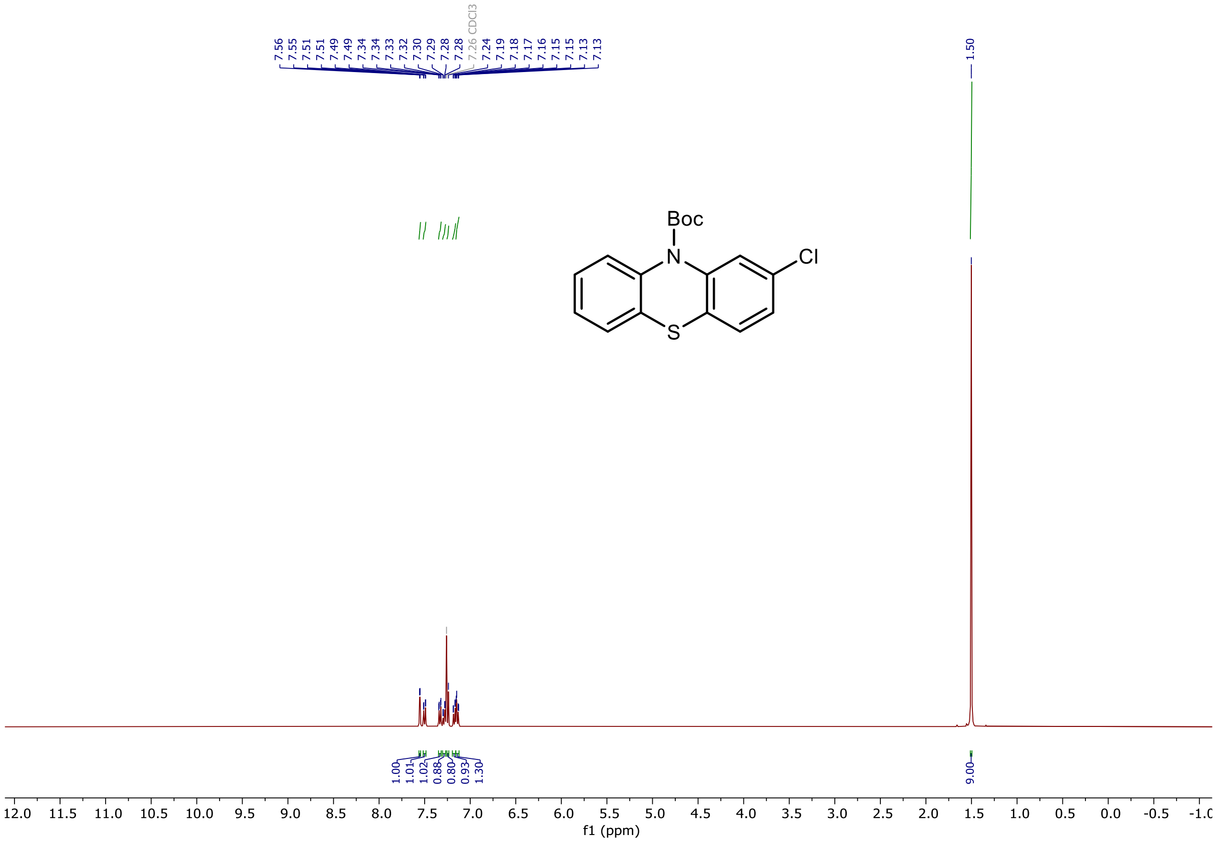

^{13}C-NMR

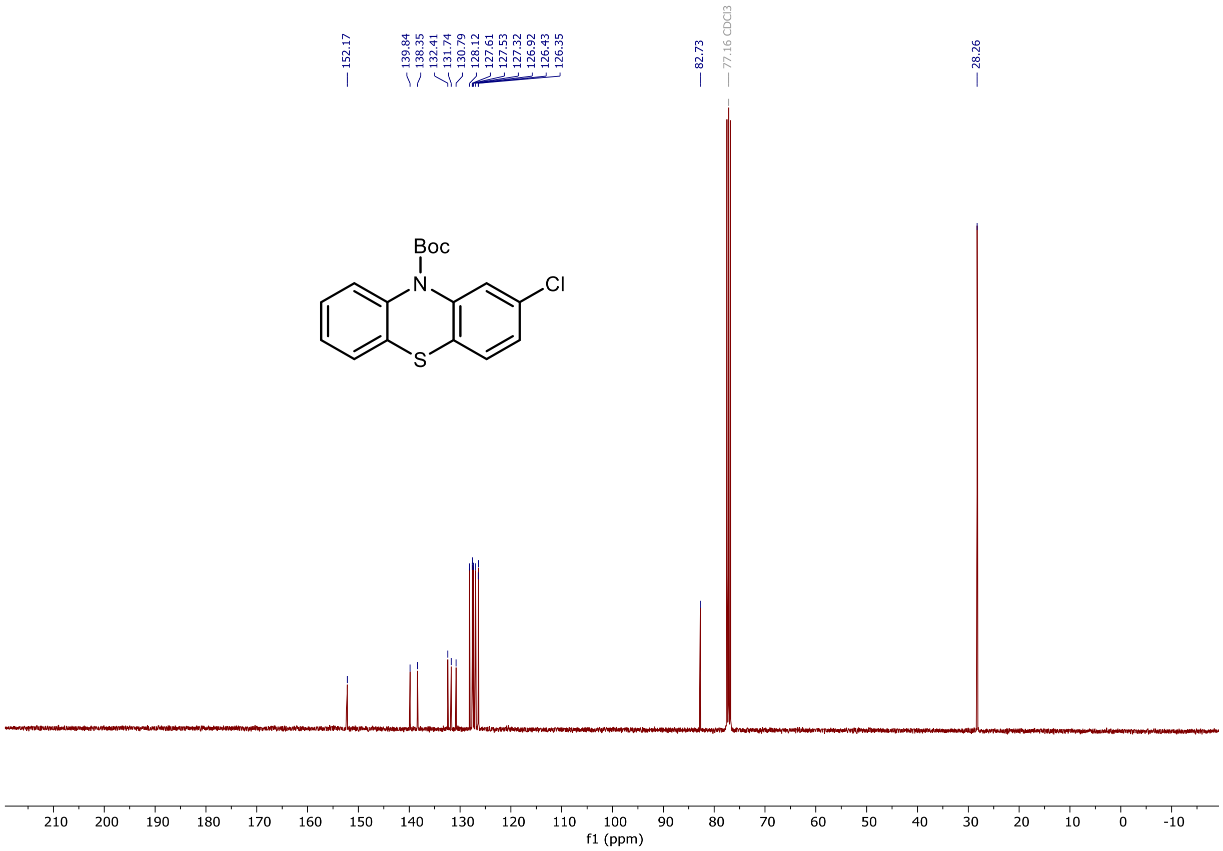

Methyl 2-((4-bromophenyl)((*tert*-butyldimethylsilyl)oxy)methyl)acrylate (Br1j)

^{1}H-NMR

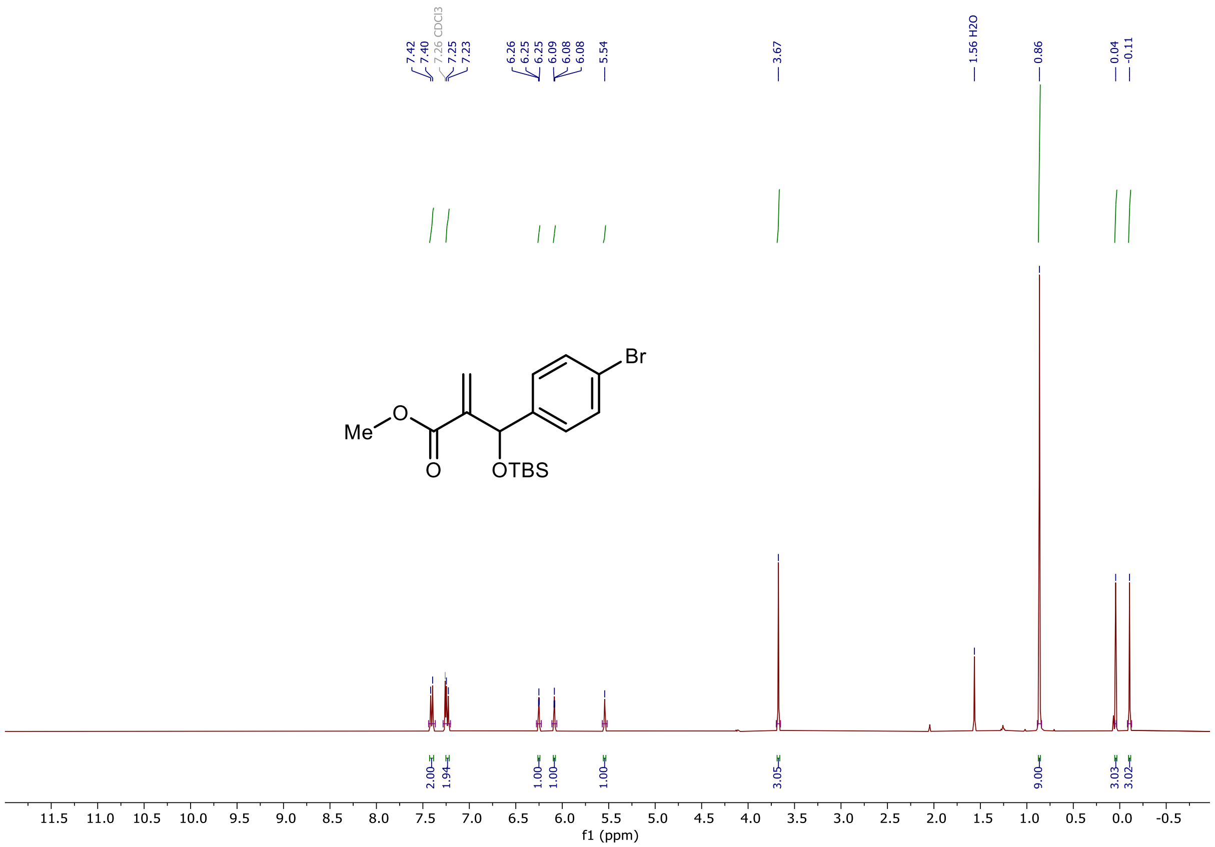

^{13}C-NMR

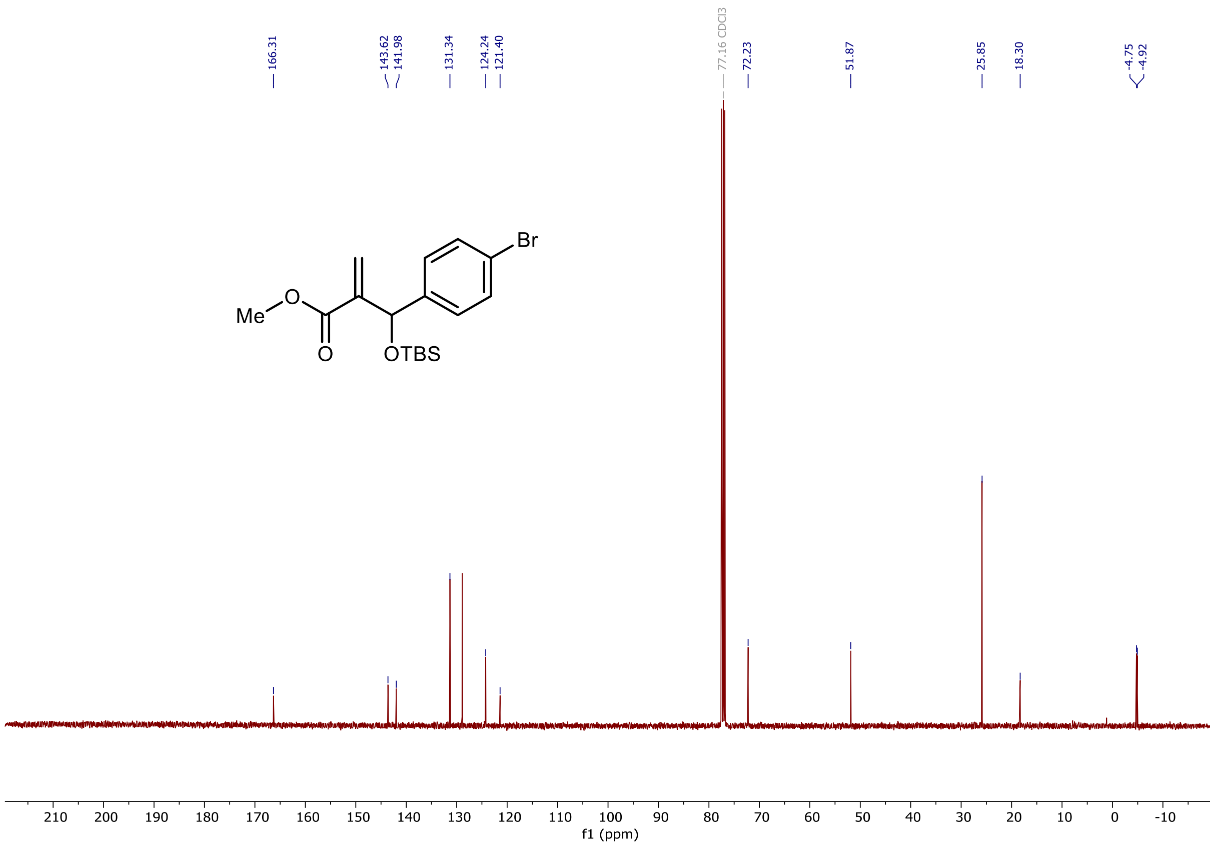

***N*-(4-Bromo-2,6-difluorophenyl)-3,3-dimethylbutanamide (Br1k)**

^{1}H-NMR

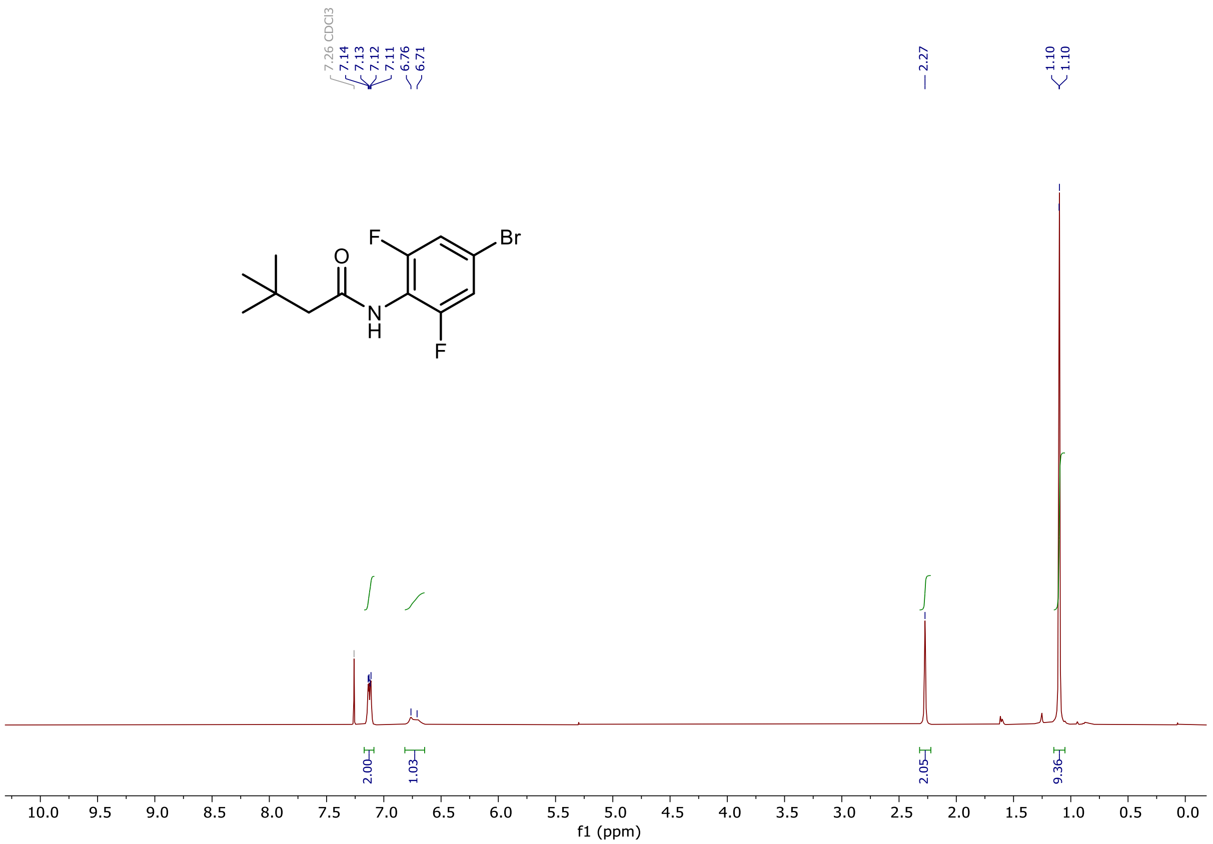

^{13}C-NMR

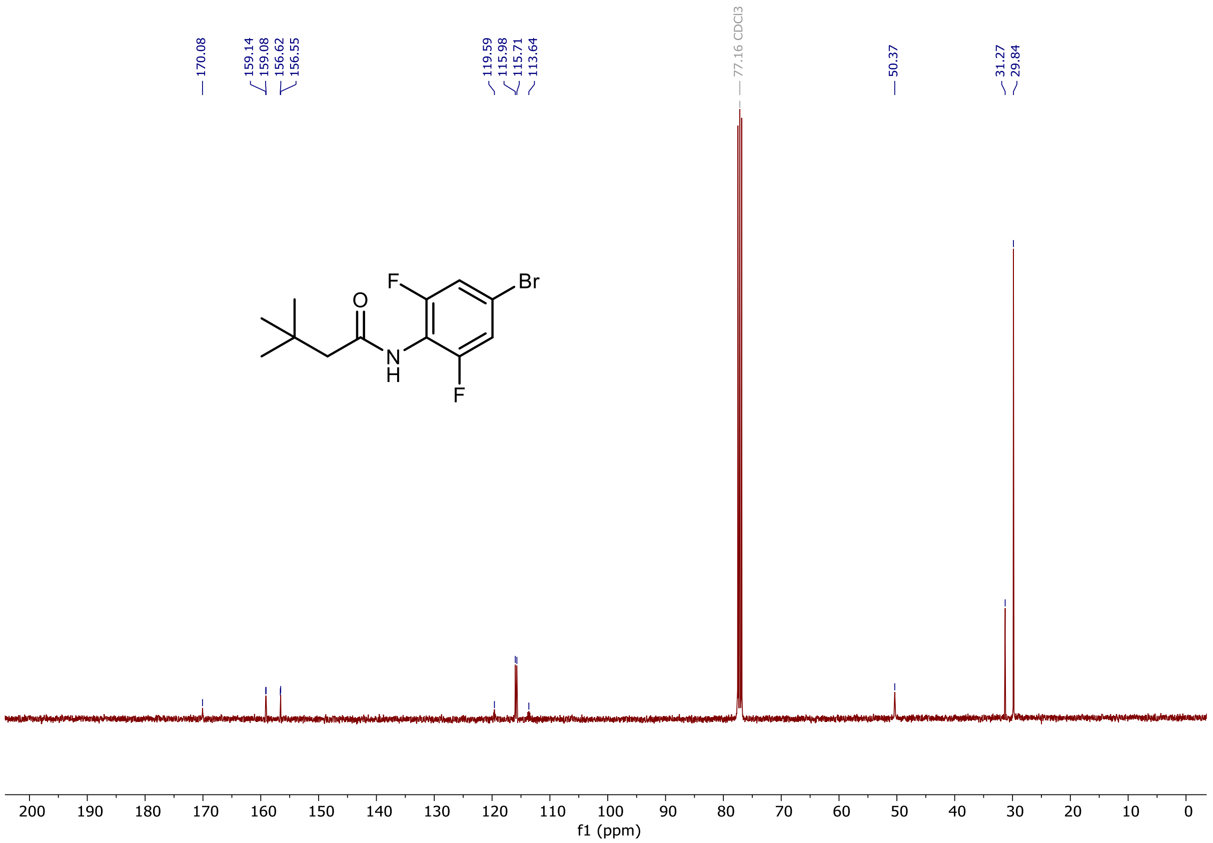

^{19}F-NMR

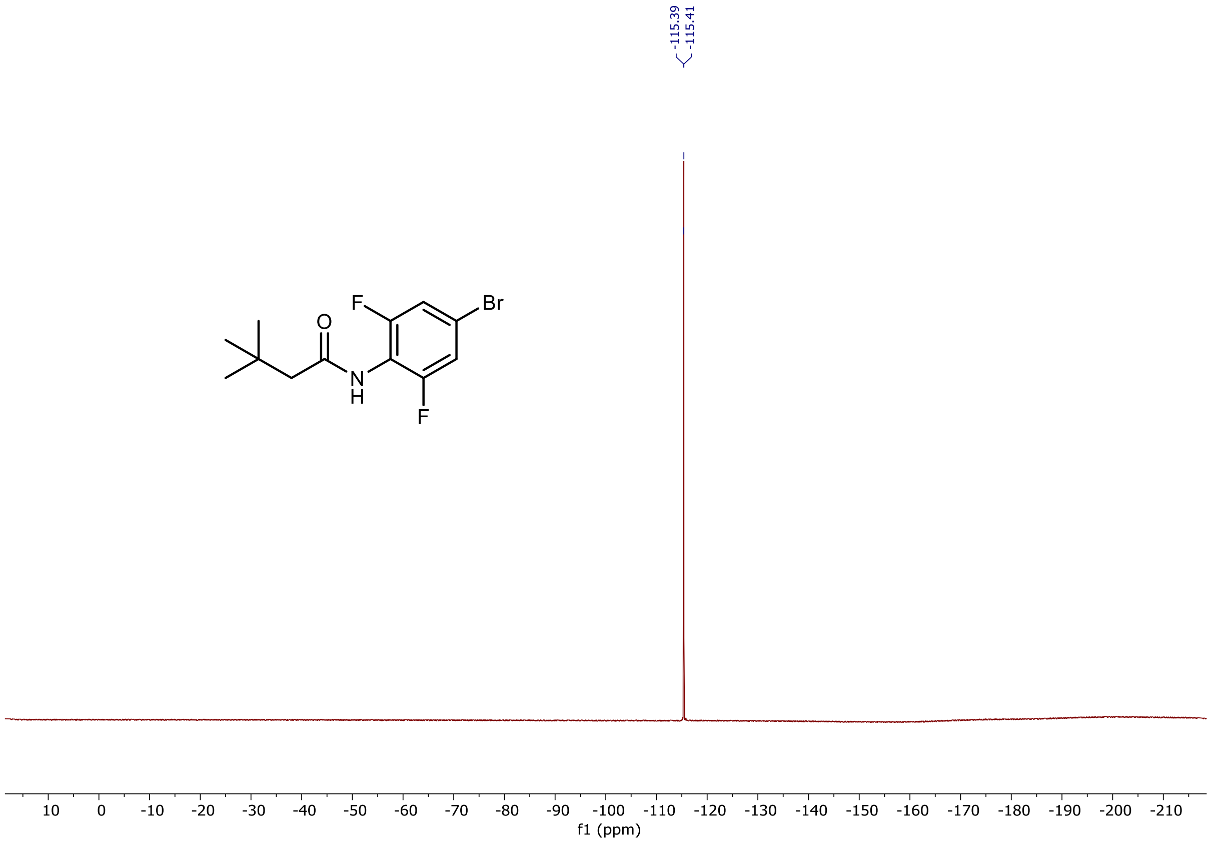
-115.39
-115.41
F
Br
O
N
H
F
10
0
-10
-20
-30
-40
-50
-60
-70
-80
-90
-100
-110
-120
-130
-140
-150
-160
-170
-180
-190
-200
-210
f1 (ppm)

***tert-B*utyl 2-(5,5-dimethyl-1,3,2-dioxaborinan-2-yl)-10*H*-phenothiazine-10-carboxylate (B1i)**

^{1}H-NMR

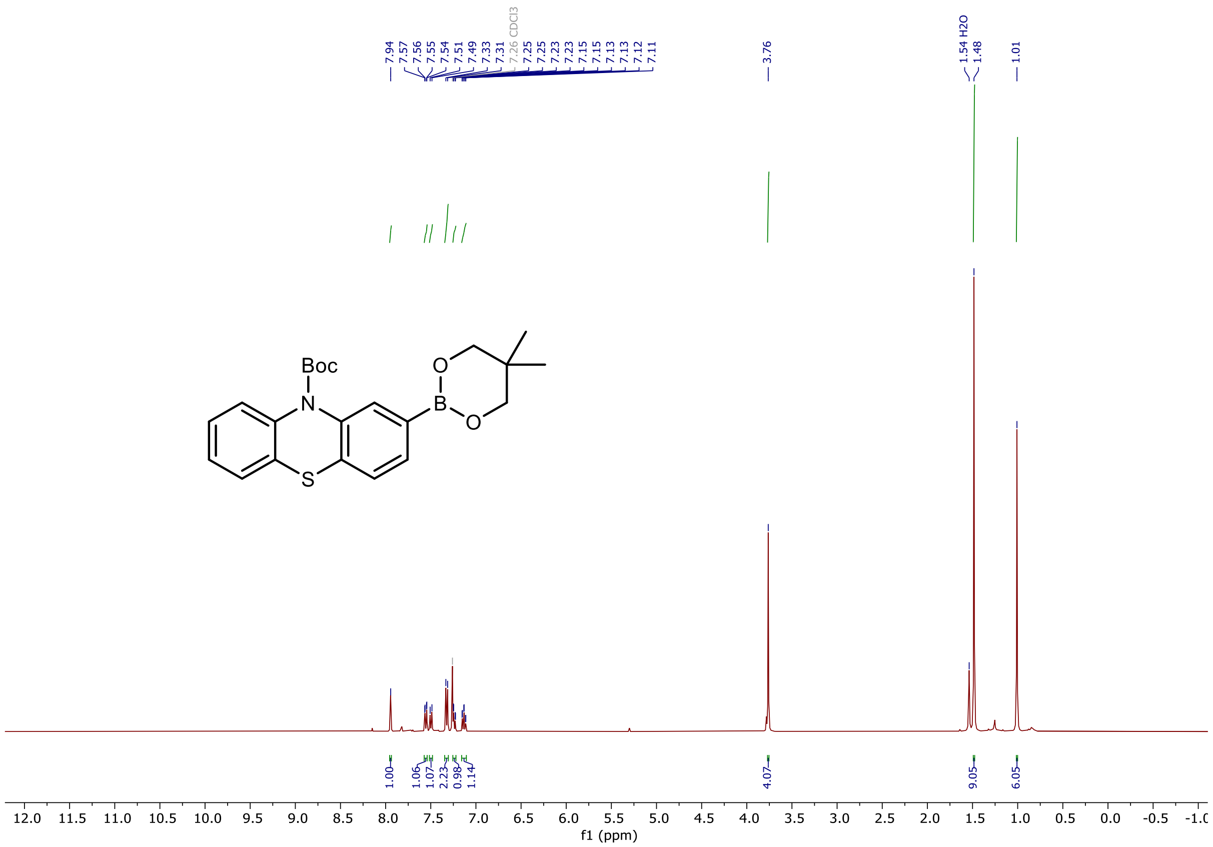

^{13}C-NMR

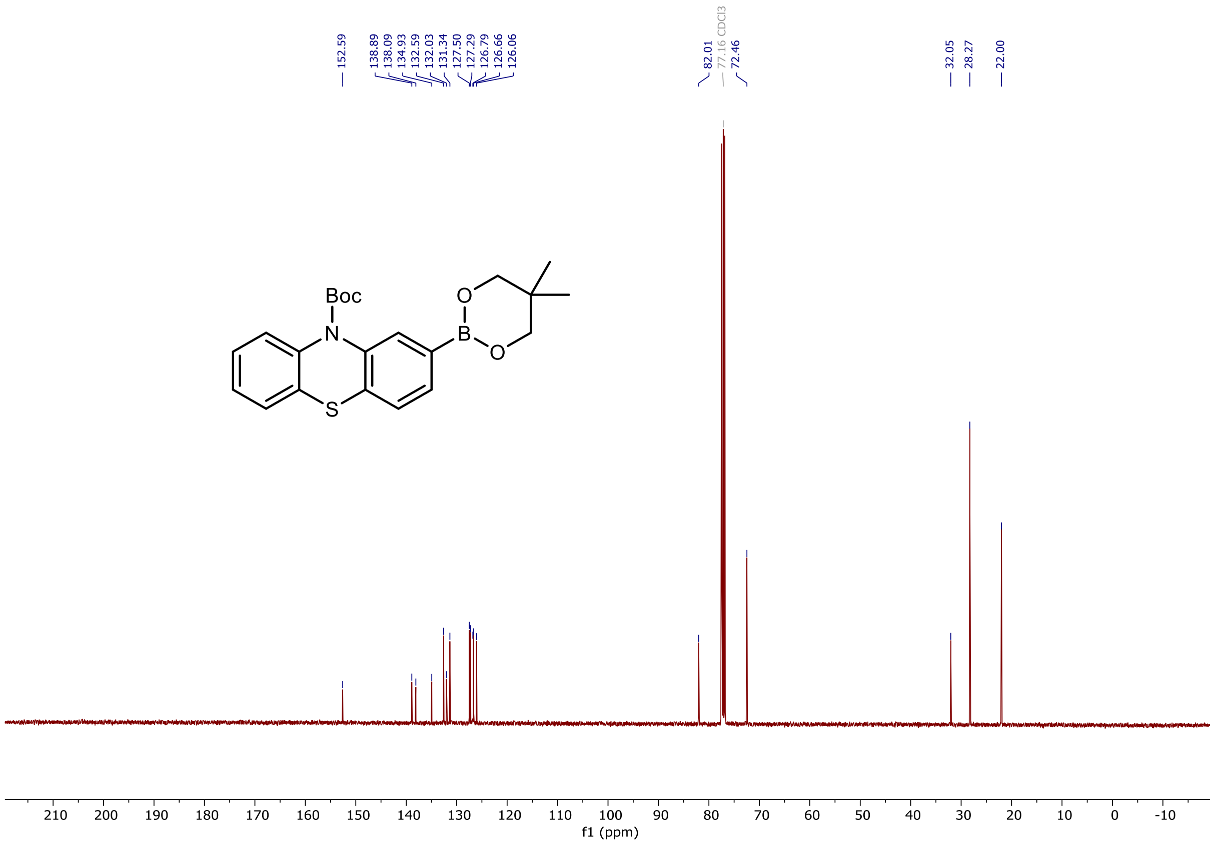

Methyl 2-(((*tert*-butyldimethylsilyl)oxy)(4-(5,5-dimethyl-1,3,2-dioxaborinan-2-yl)phenyl)methyl)acrylate (B1j)

^{1}H-NMR

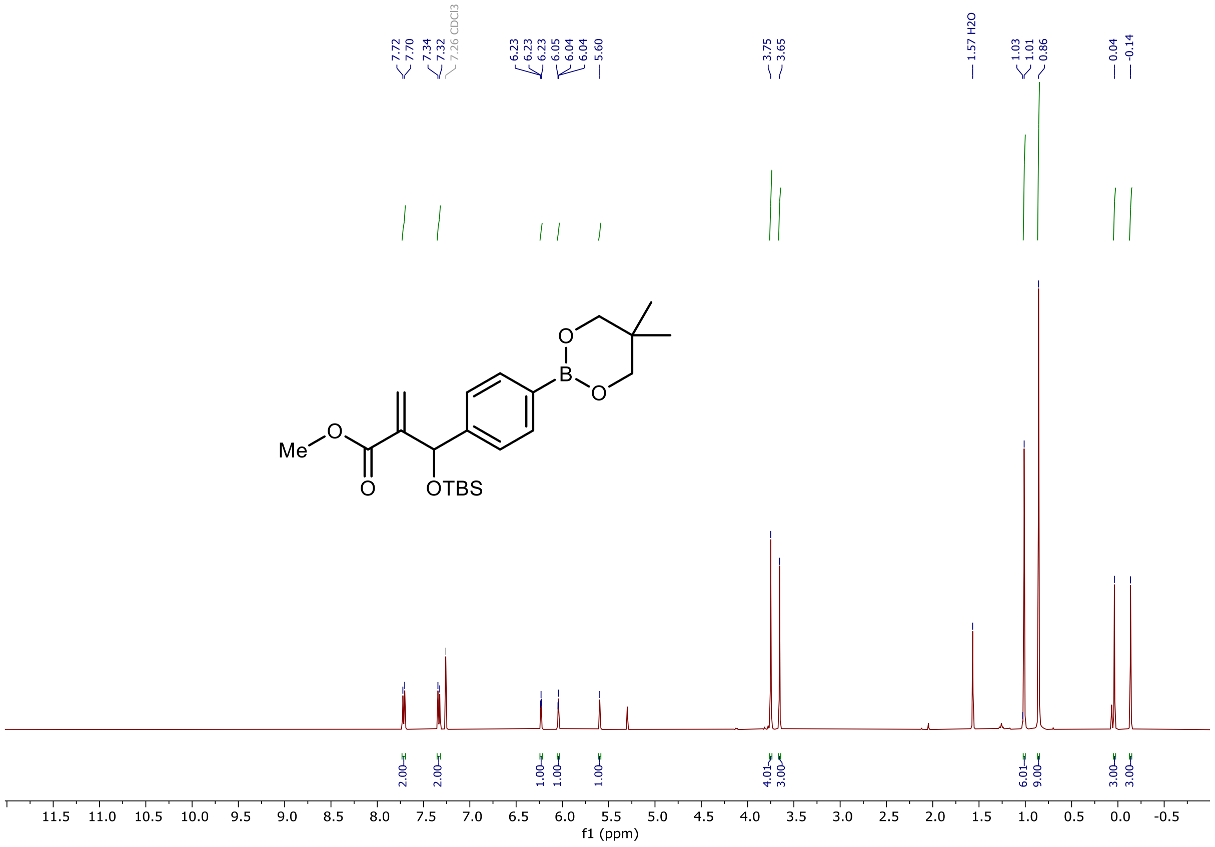

^{13}C-NMR

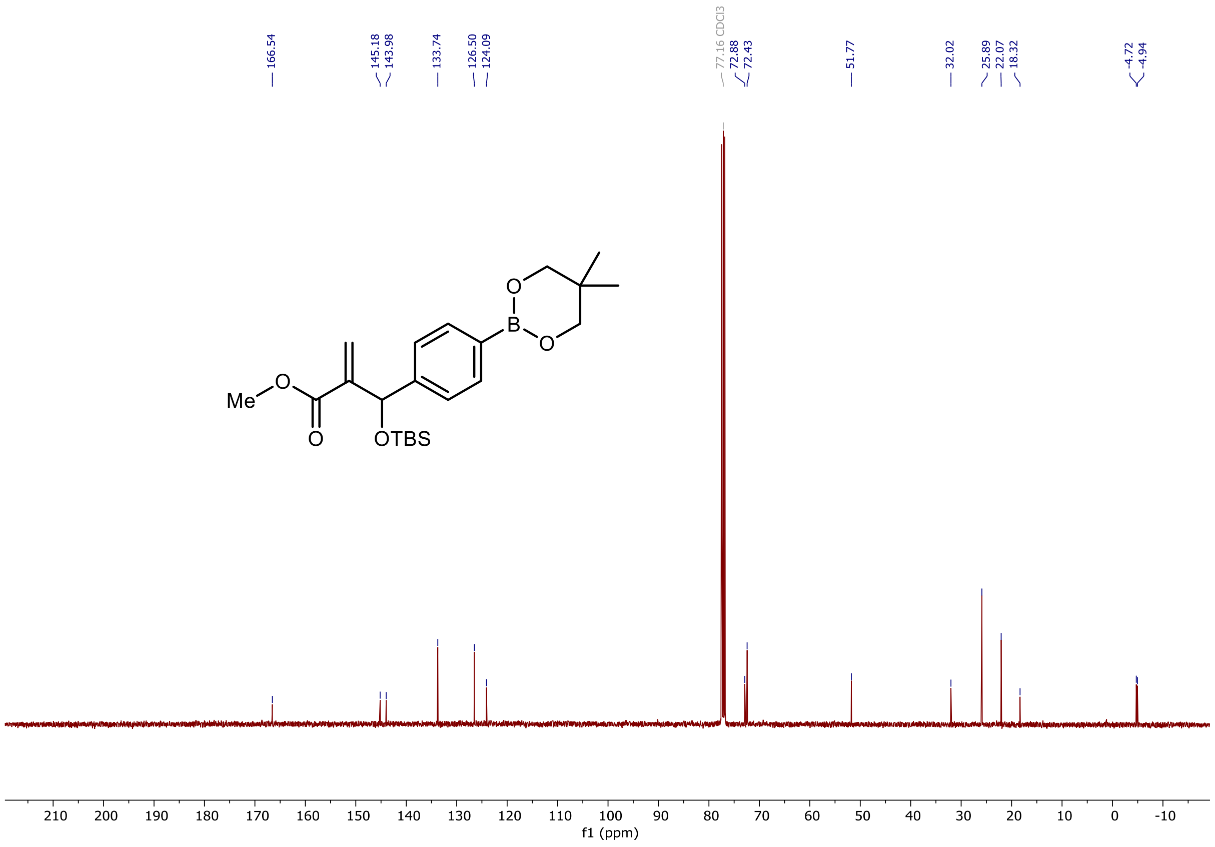

3,5-difluoro-4-(3,3-dimethylbutanamido)phenylboronic acid neopentyl glycol ester (B1k)

^{1}H-NMR

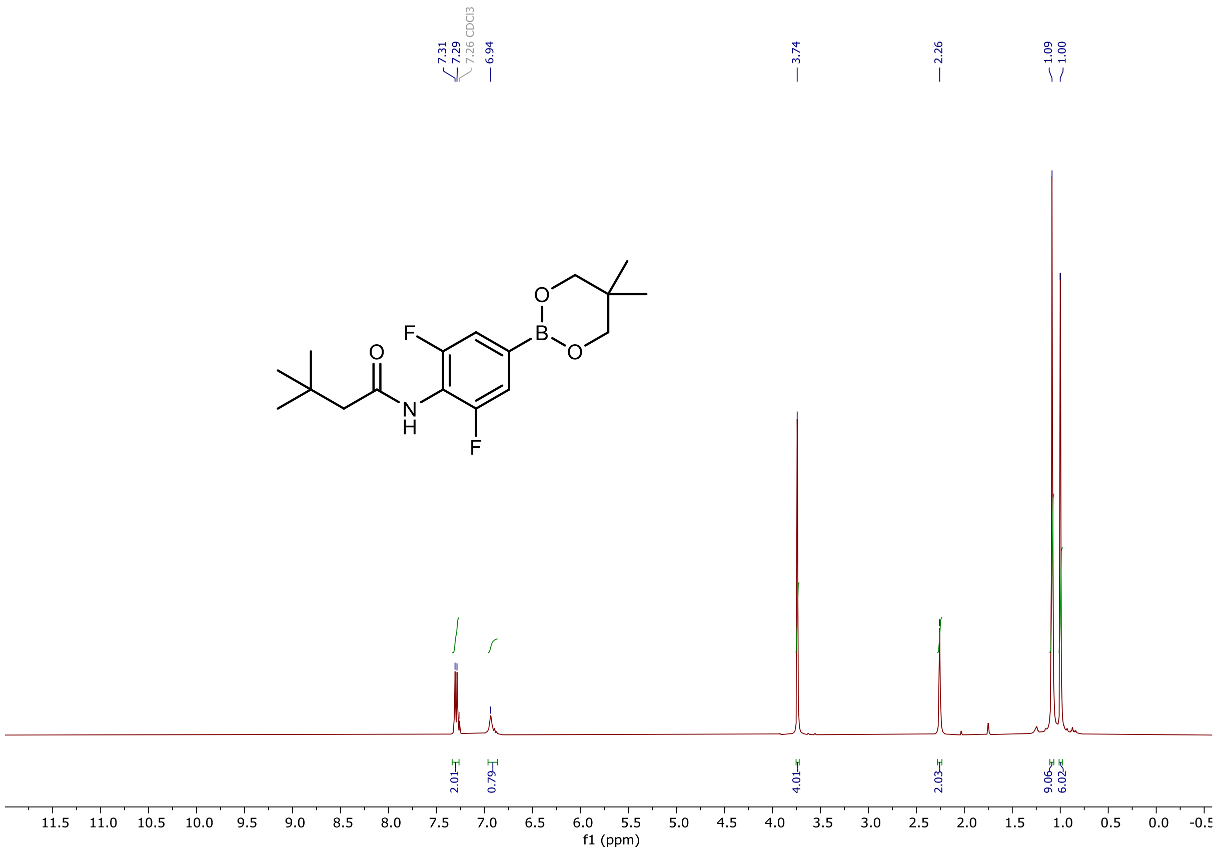

^{13}C-NMR

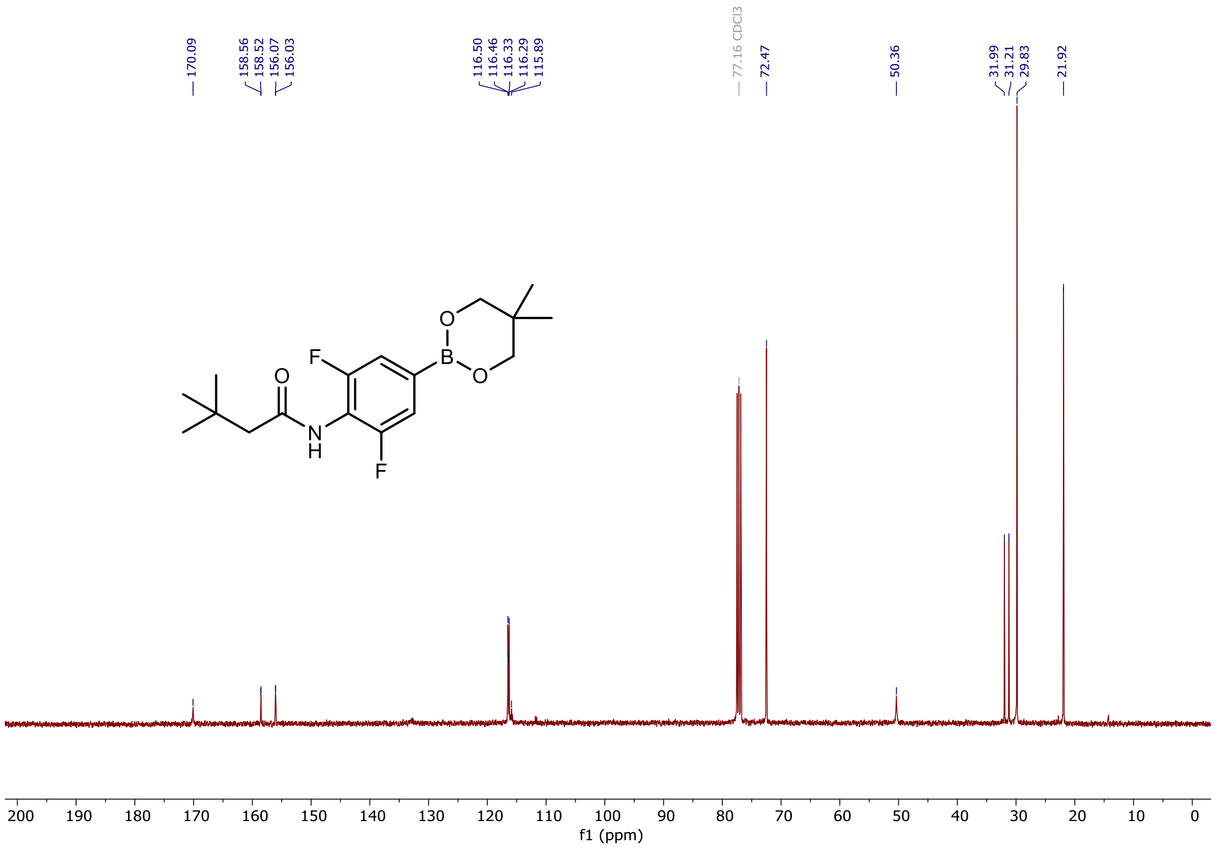

^{19}F-NMR

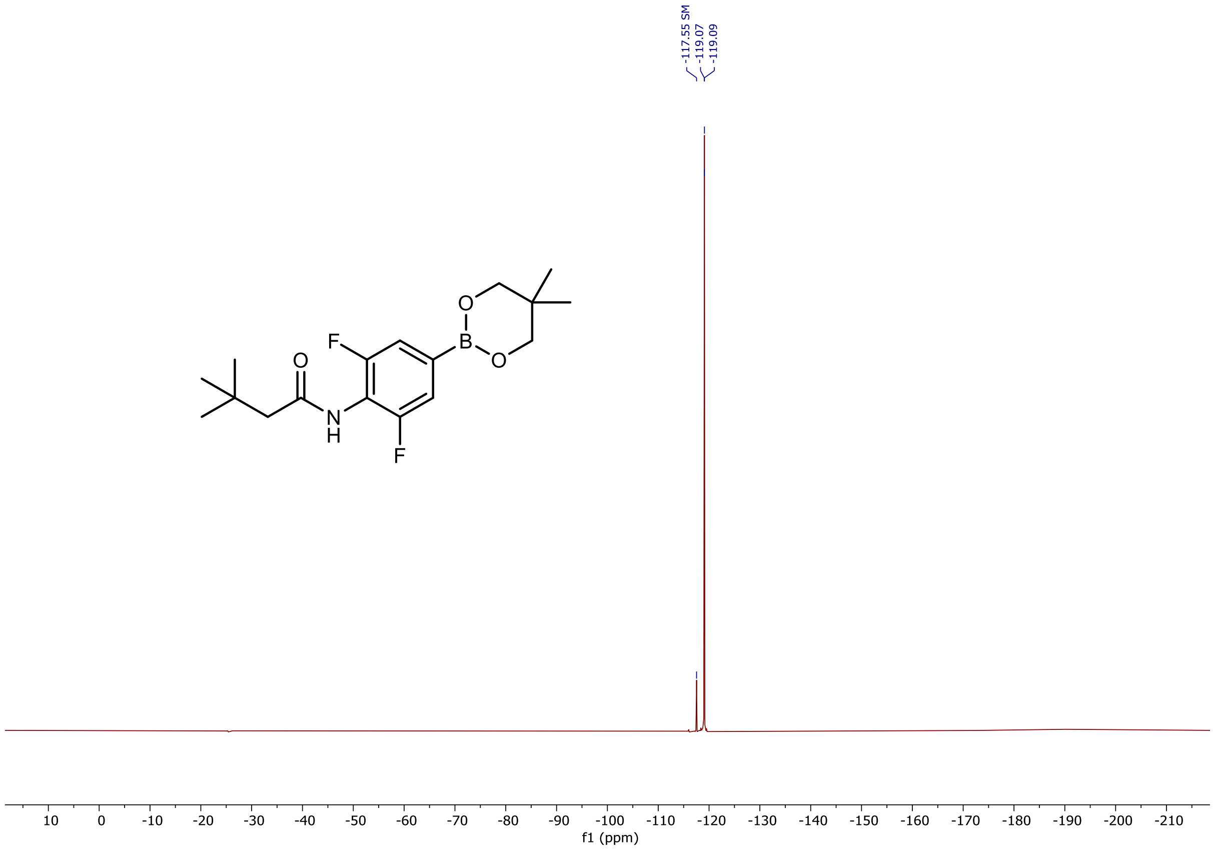

-117.55 SM
-119.07
-119.09
F
F
O
N
H
B
O
O
10
0
-10
-20
-30
-40
-50
-60
-70
-80
-90
-100
-110
-120
-130
-140
-150
-160
-170
-180
-190
-200
-210
f1 (ppm)

5-(5,5-dimethyl-1,3,2-dioxaborinan-2-yl)-2-(2,6-dioxopiperidin-3-yl)isoindoline-1,3-dione (B1q)

^{1}H-NMR

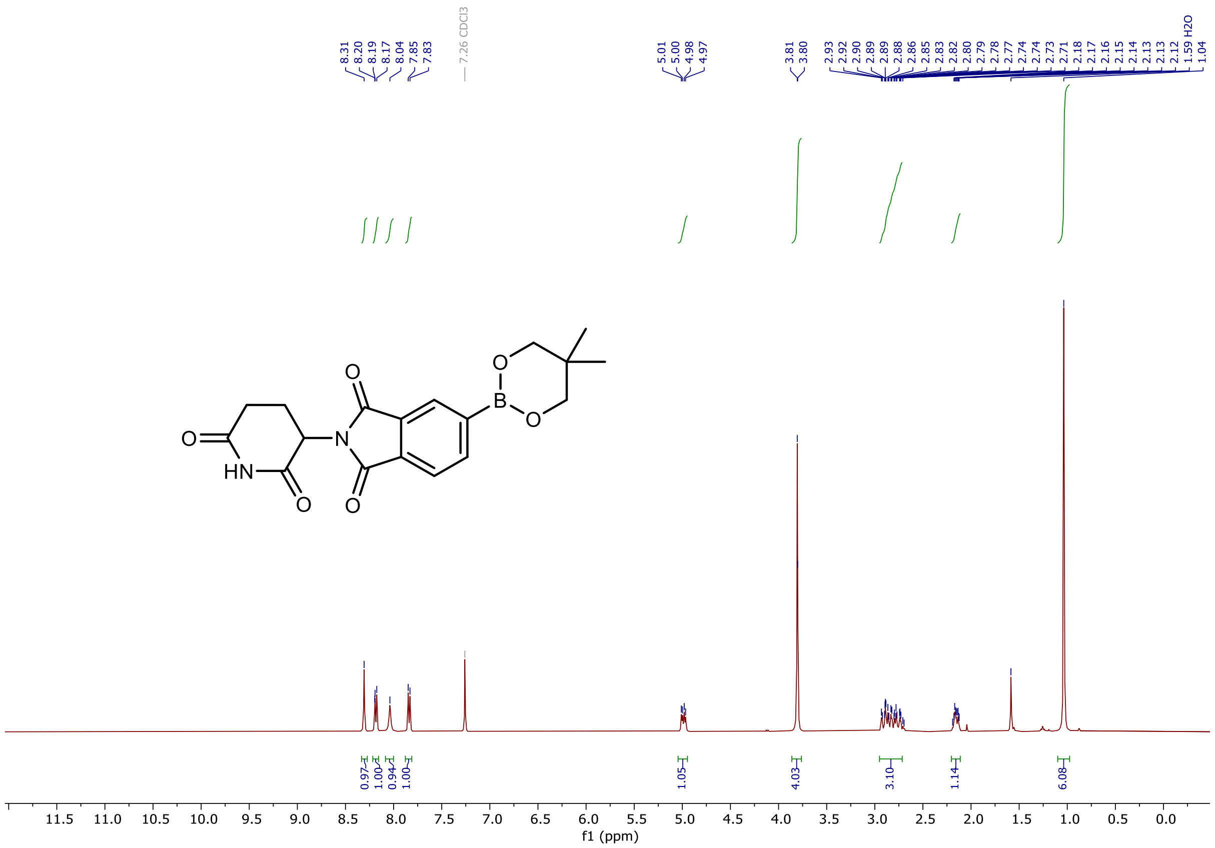

^{13}C-NMR

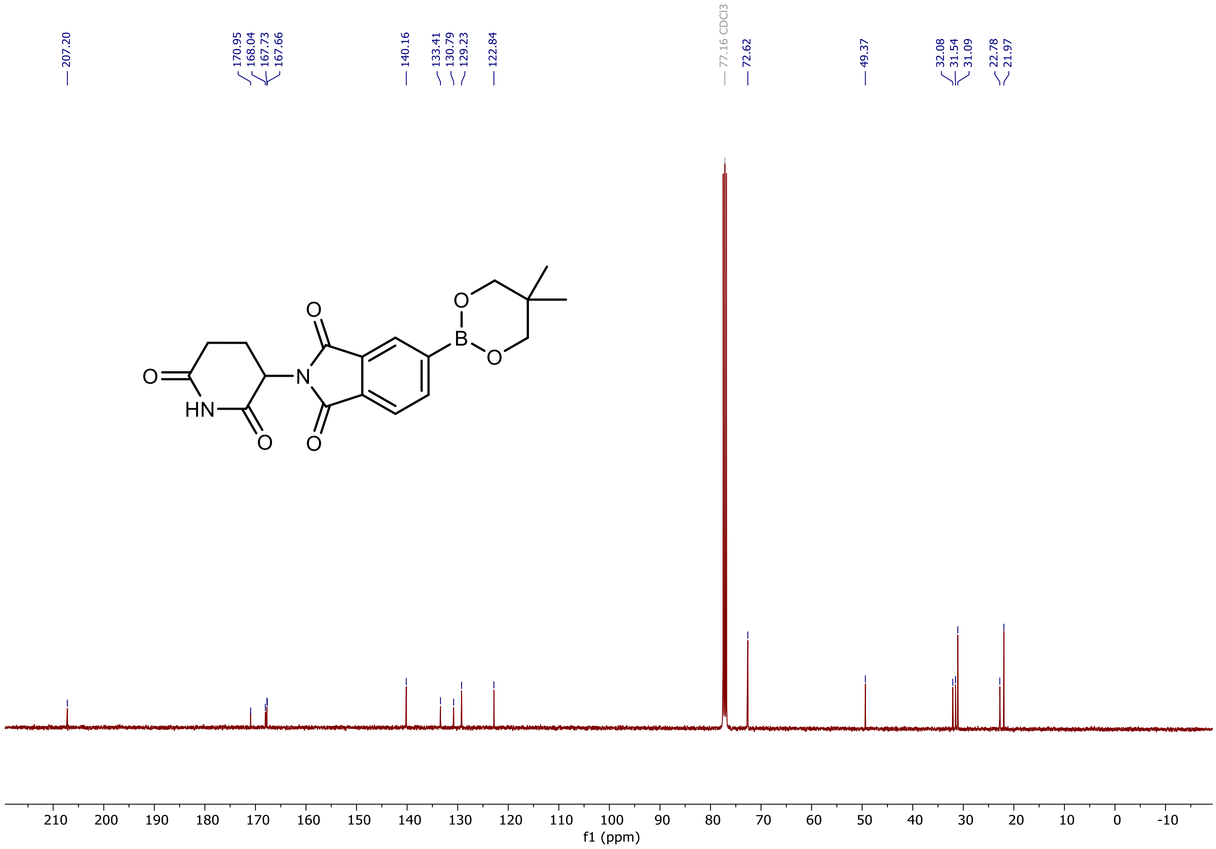

trans-Chloro(propylaminecarbonyl)bis(triphenylphosphine) palladium(II) (Pd-1)

^{1}H-NMR

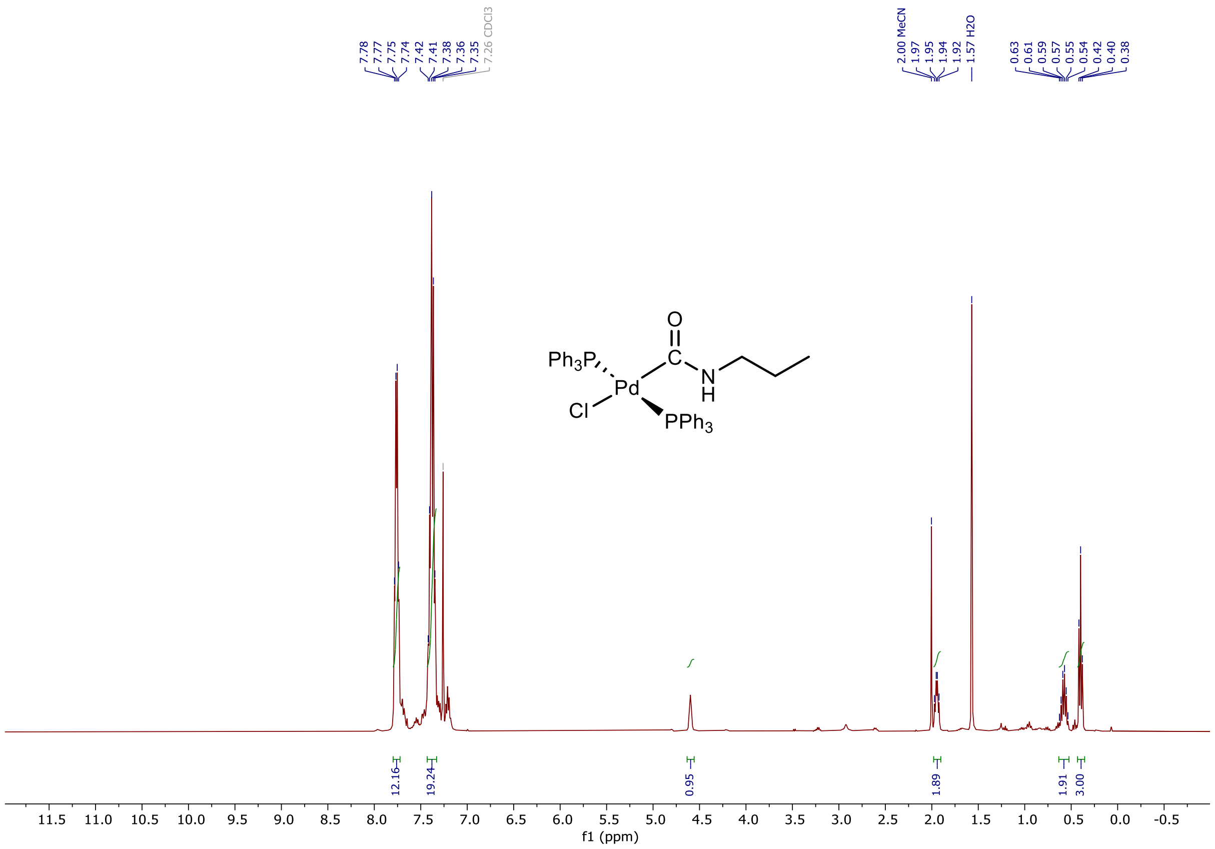

^{13}C-NMR

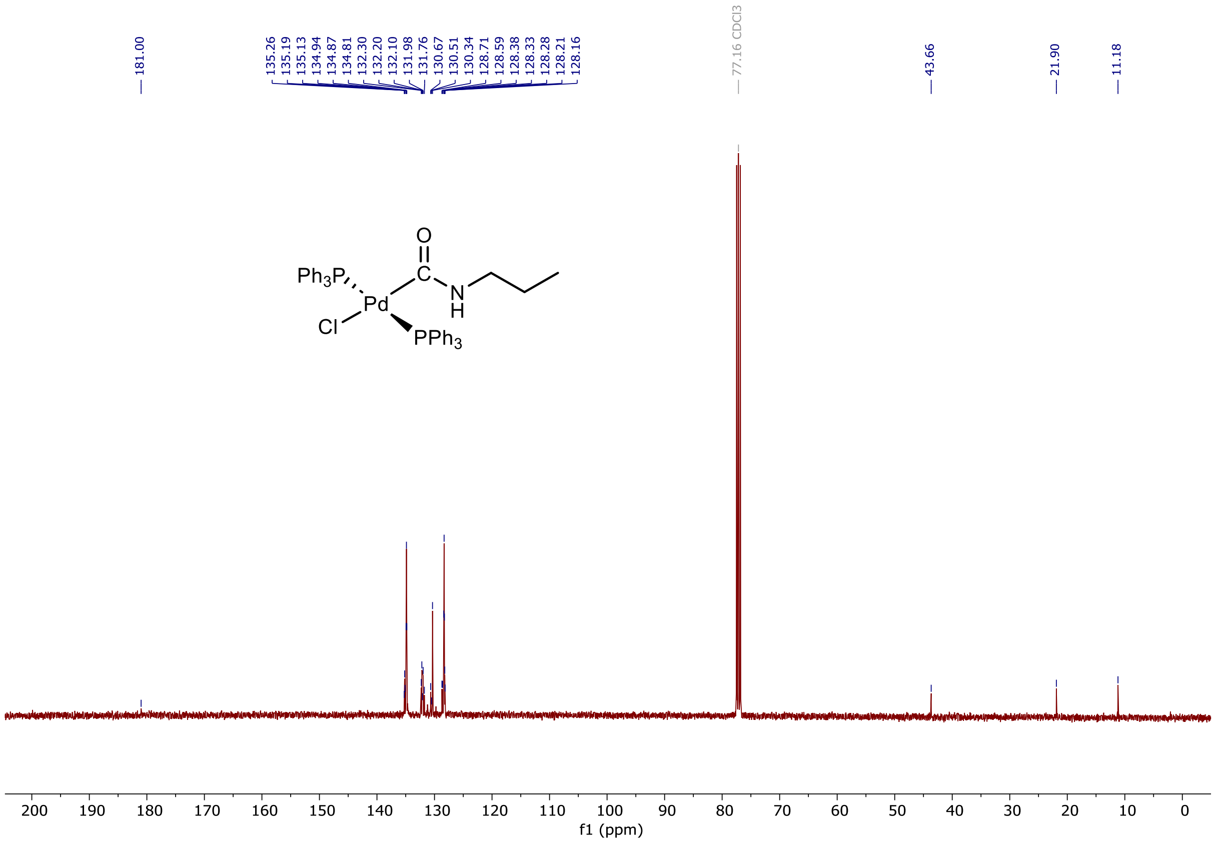

^{31}P-NMR

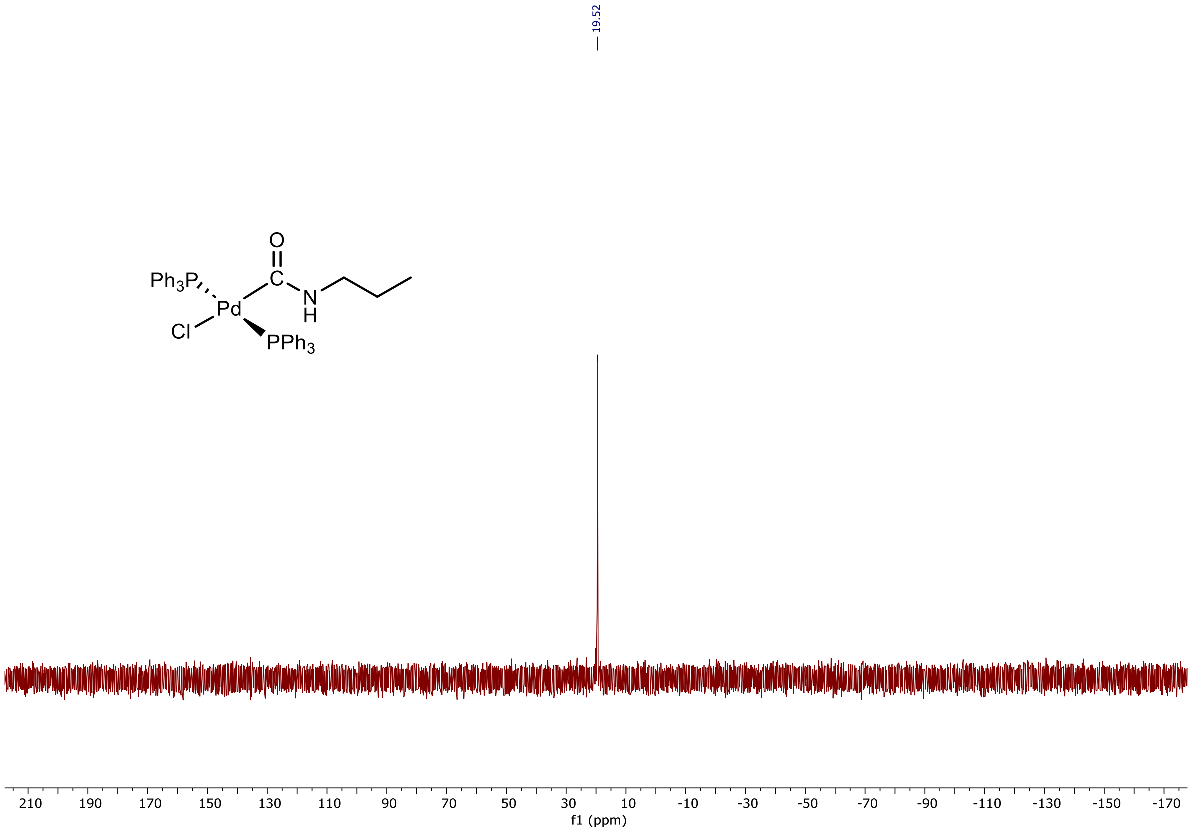
Ph3P
Pd
Cl
PPh3
O
C
N
H
19.52
210
190
170
150
130
110
90
70
50
30
10
-10
-30
-50
-70
-90
-110
-130
-150
-170
f1 (ppm)

trans-Chloro(propylamine-^{13}C-carbonyl)bis(triphenylphosphine) palladium(II) (^{13}C-Pd-1)

^{1}H-NMR

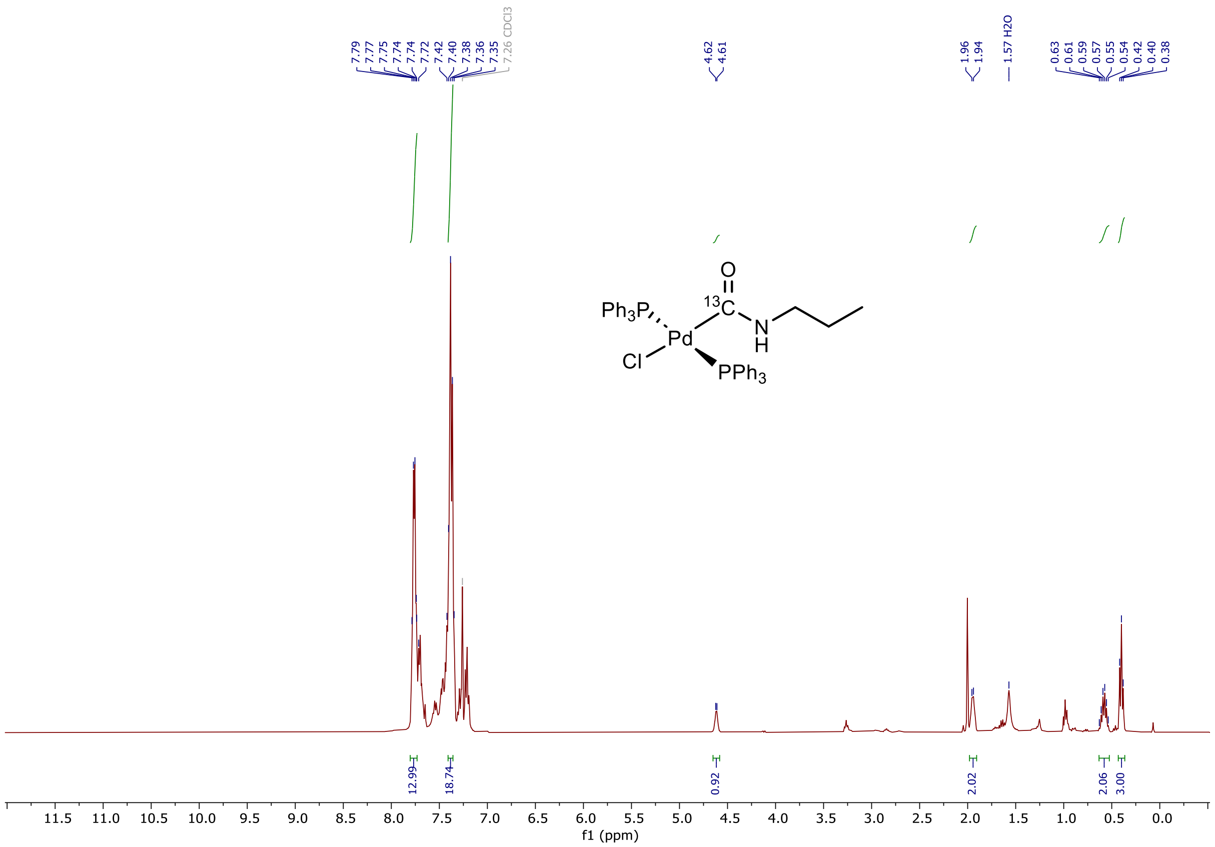

^{13}C-NMR

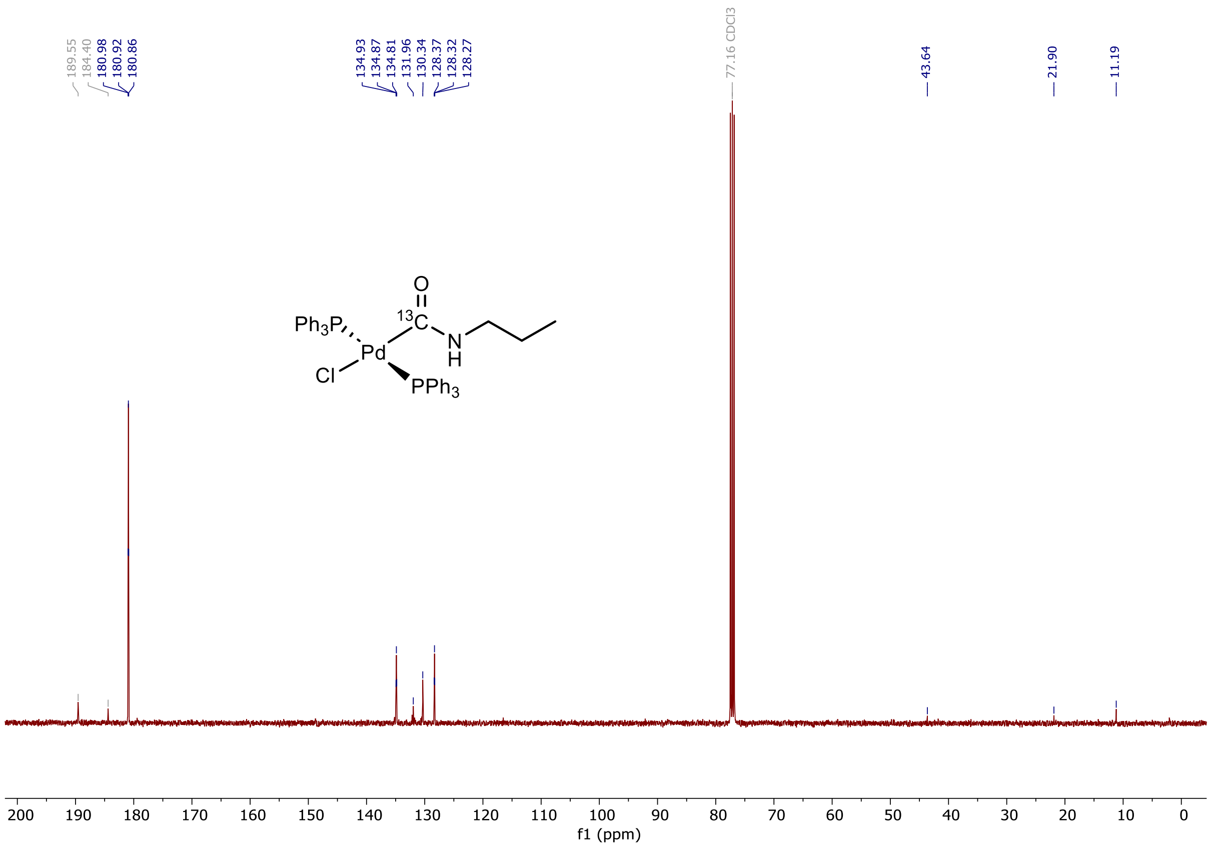

^{31}P-NMR

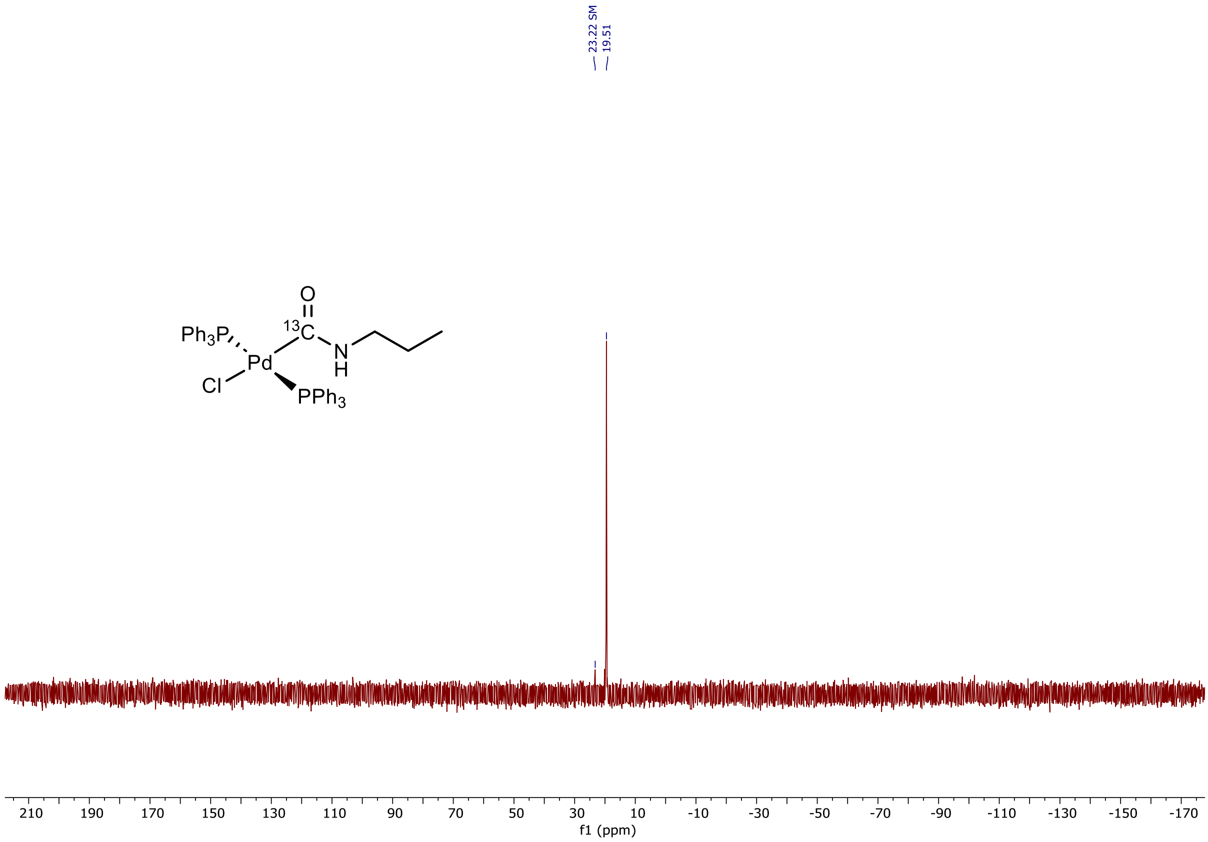

23.22 SM
19.51
Ph3P
13C
O
N
H
Pd
Cl
PPh3
210
190
170
150
130
110
90
70
50
30
10
-10
-30
-50
-70
-90
-110
-130
-150
-170
f1 (ppm)

trans-Chloro(benzylamidecarbonyl)bis(triphenylphosphine) palladium(II) (Pd-2)

^{1}H-NMR

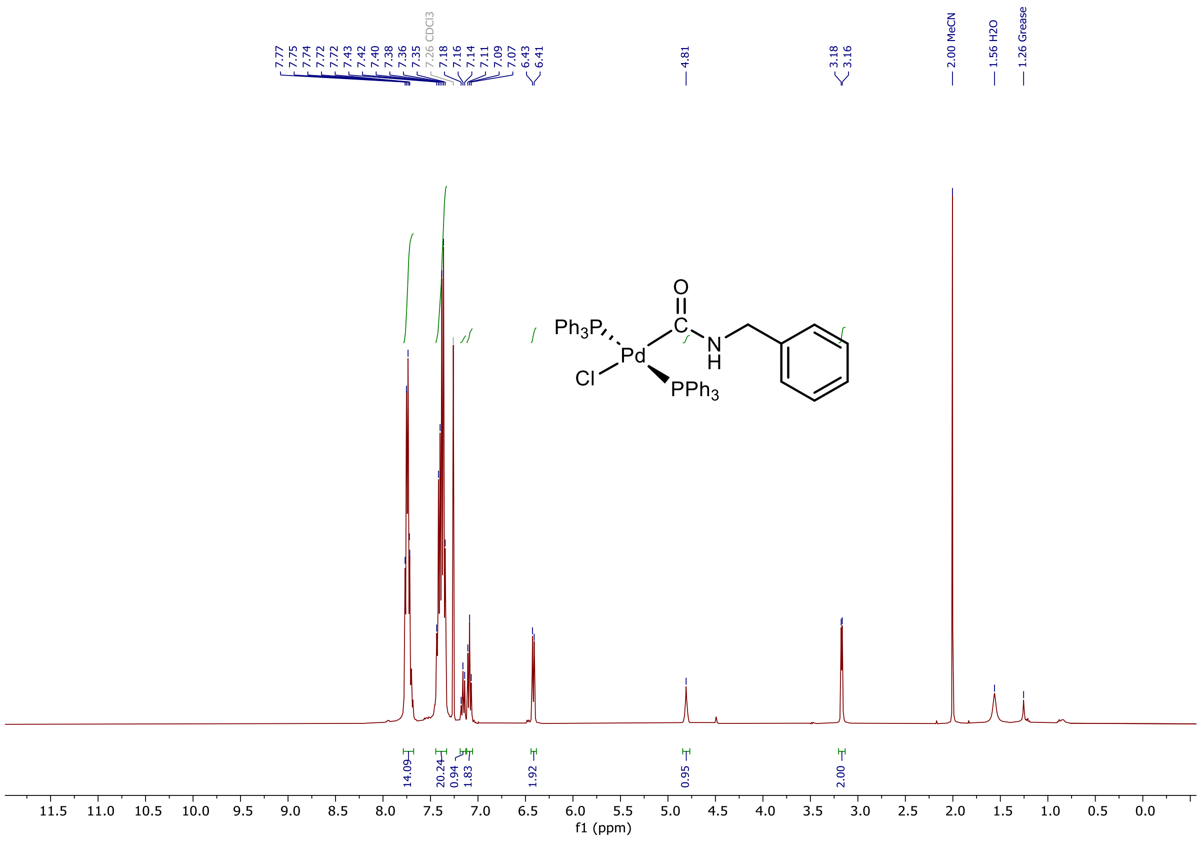

^{13}C-NMR

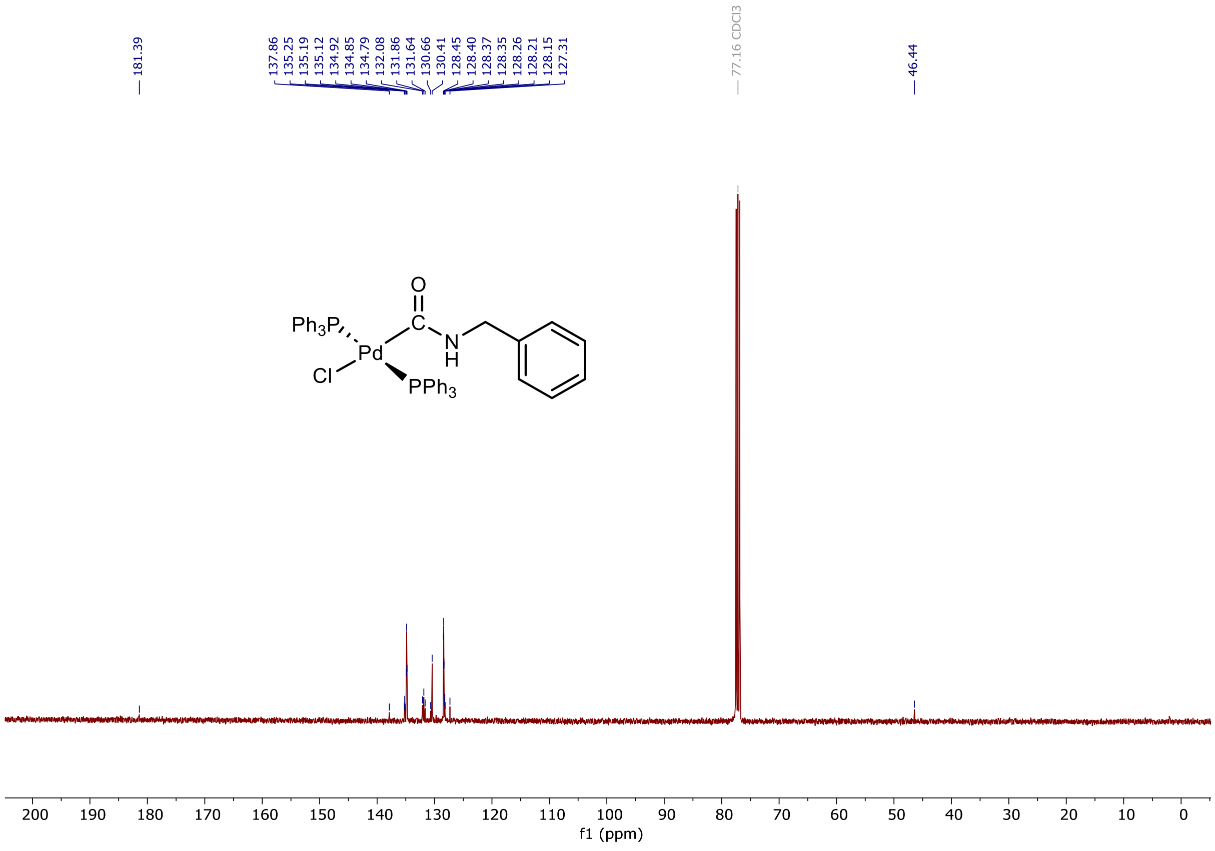

^{31}P-NMR

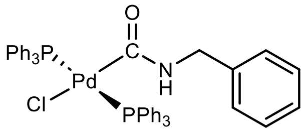
Ph3P
Pd
Cl
PPh3
O
C
N
H

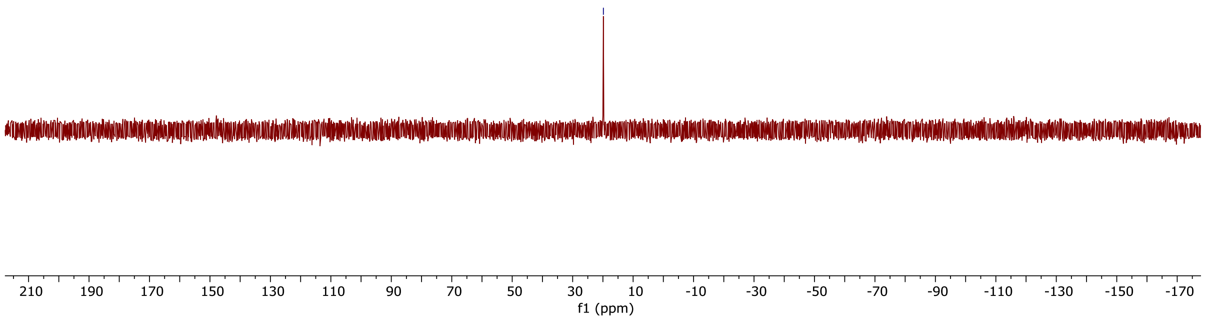
19.84
210
190
170
150
130
110
90
70
50
30
10
-10
-30
-50
-70
-90
-110
-130
-150
-170
f1 (ppm)

***trans*-Chloro(2,4-difluorobenzylaminecarbonyl)bis(triphenylphosphine) palladium(II) (Pd-3)**

^{1}H-NMR

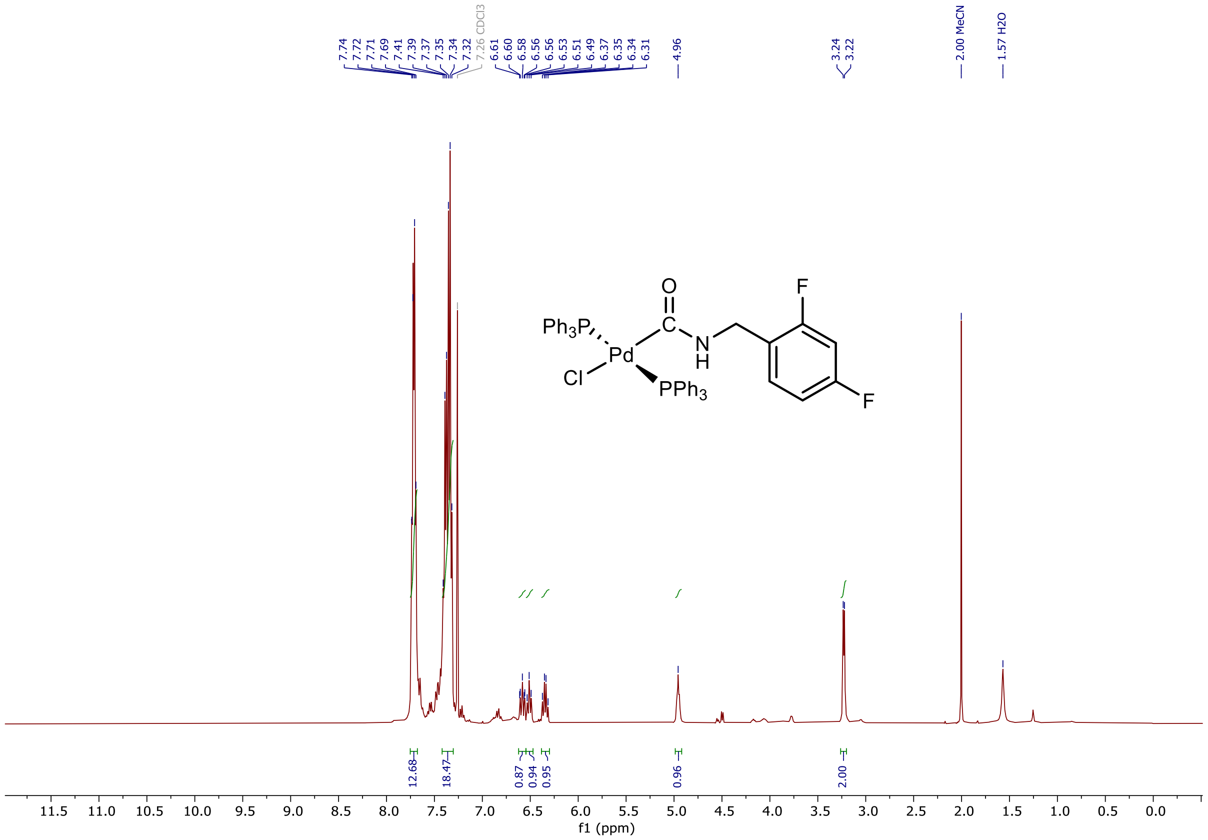

^{13}C-NMR

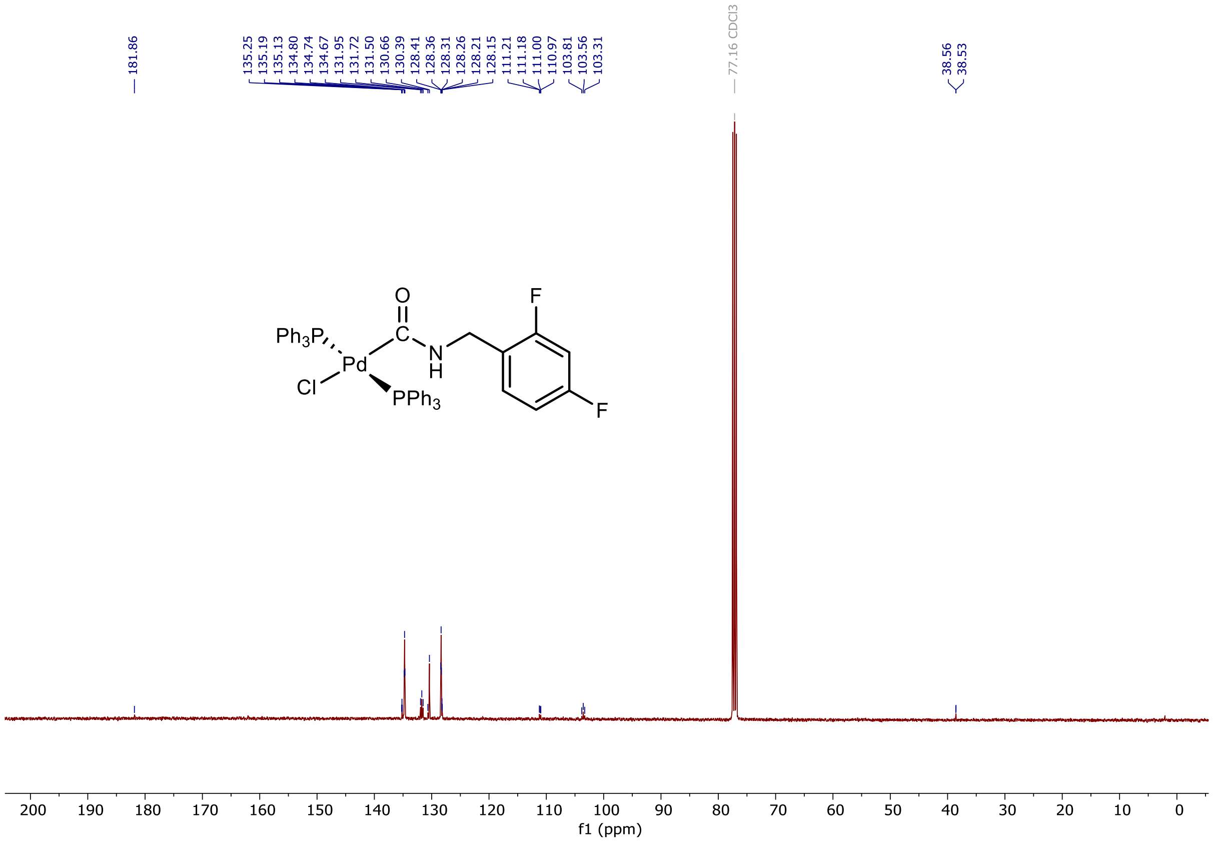

^{31}P-NMR

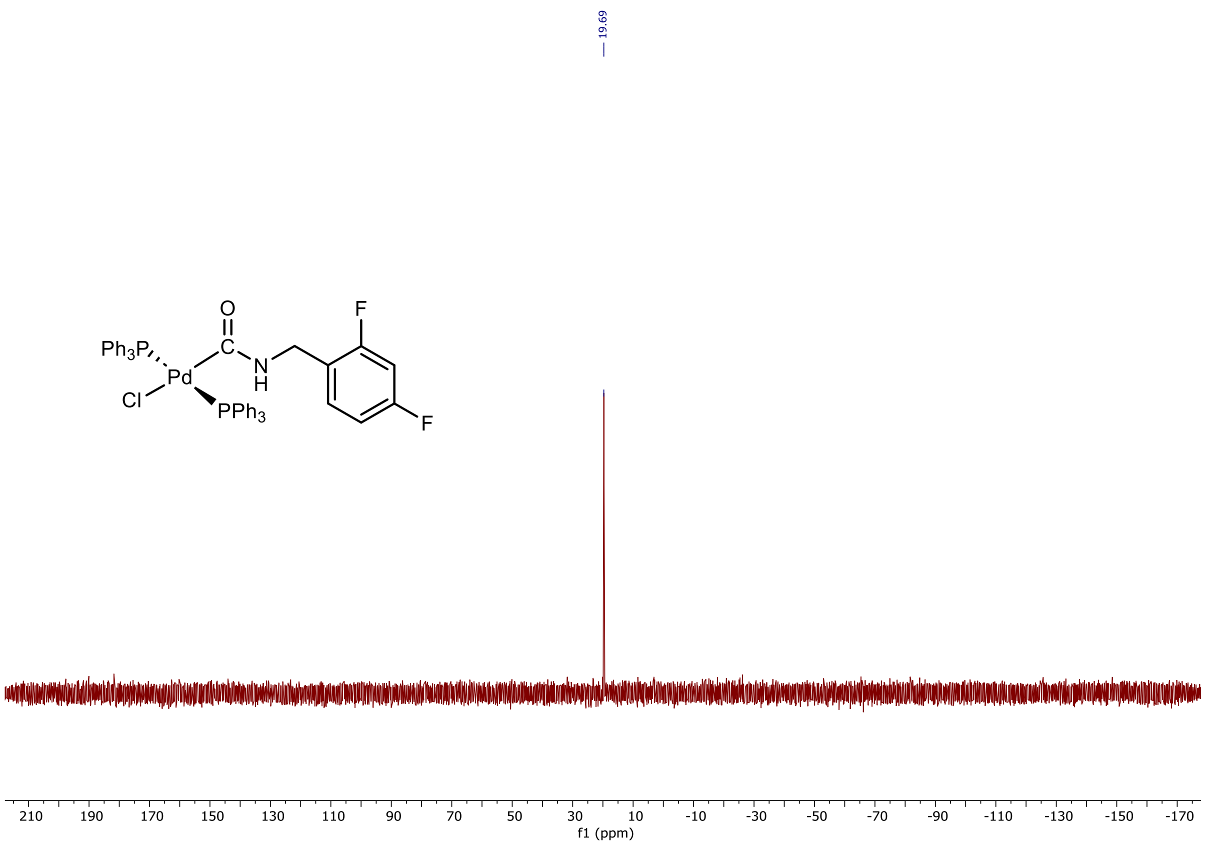

^{19}F-NMR

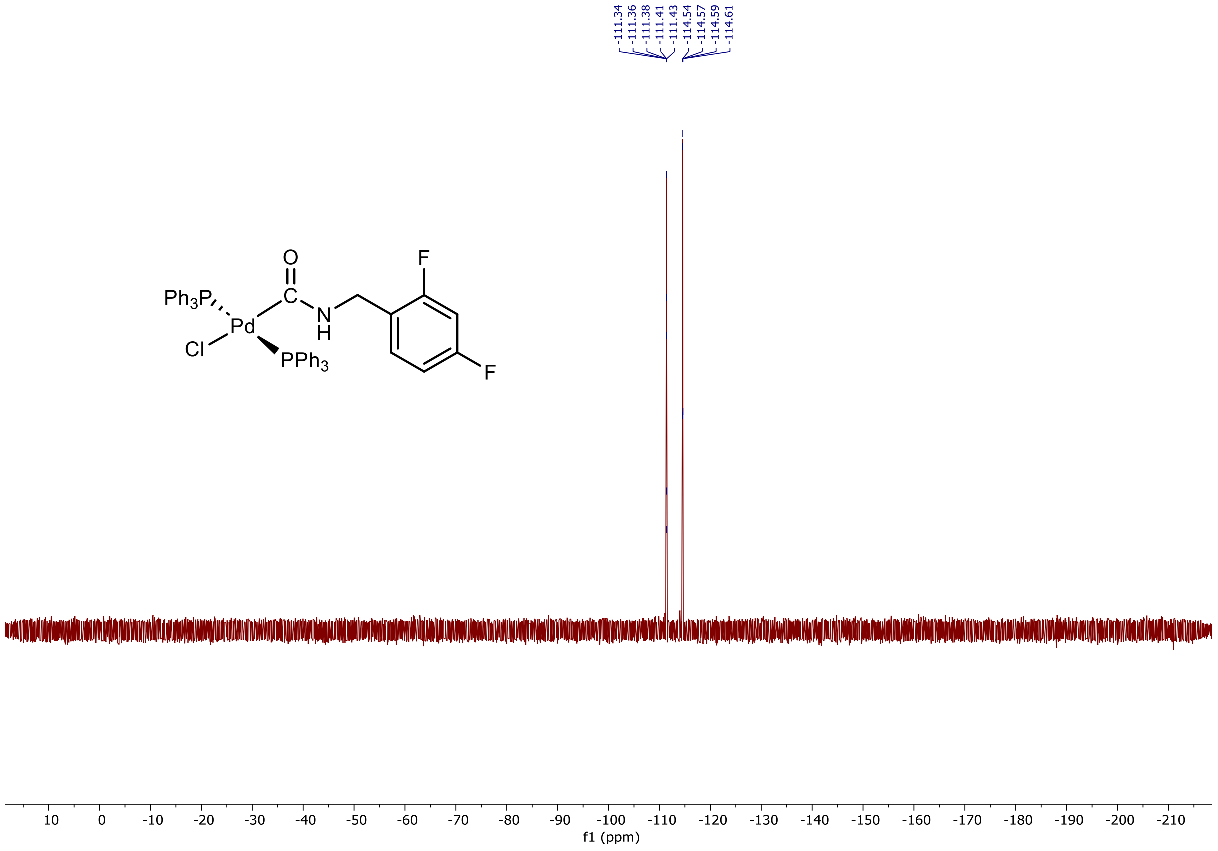

***trans*-Chloro(2,4-difluorobenzylamine-^{13}C-carbonyl)bis(triphenylphosphine) palladium(II) (^{13}C-Pd-3)**

^{1}H-NMR

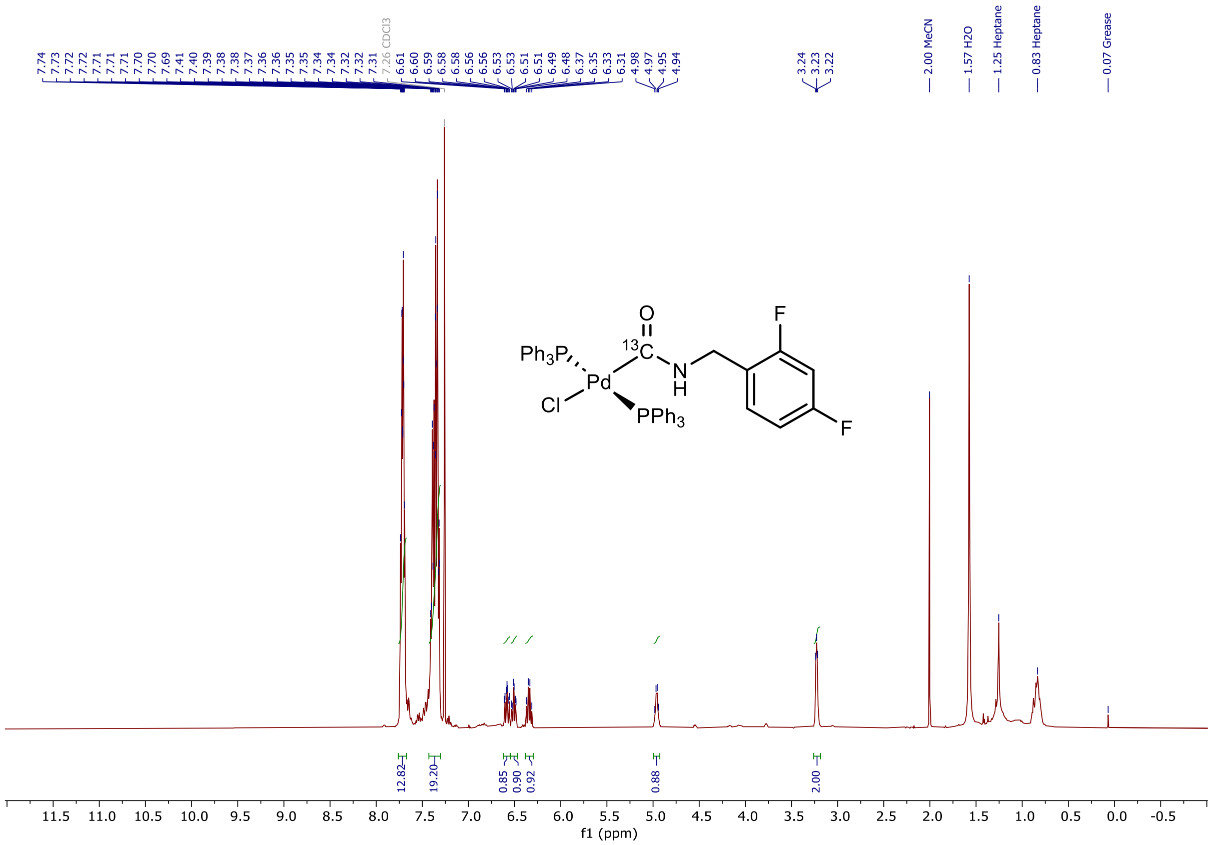

^{13}C-NMR

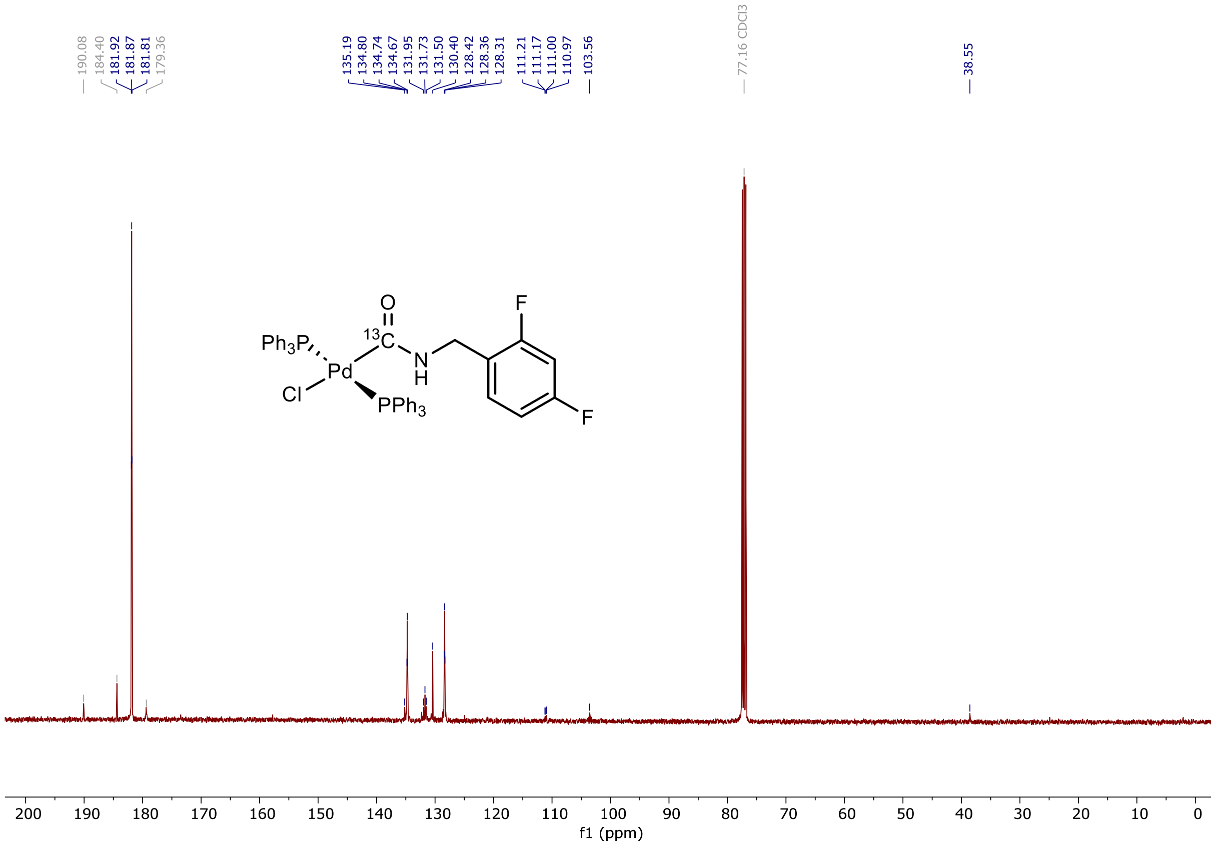

^{31}P-NMR

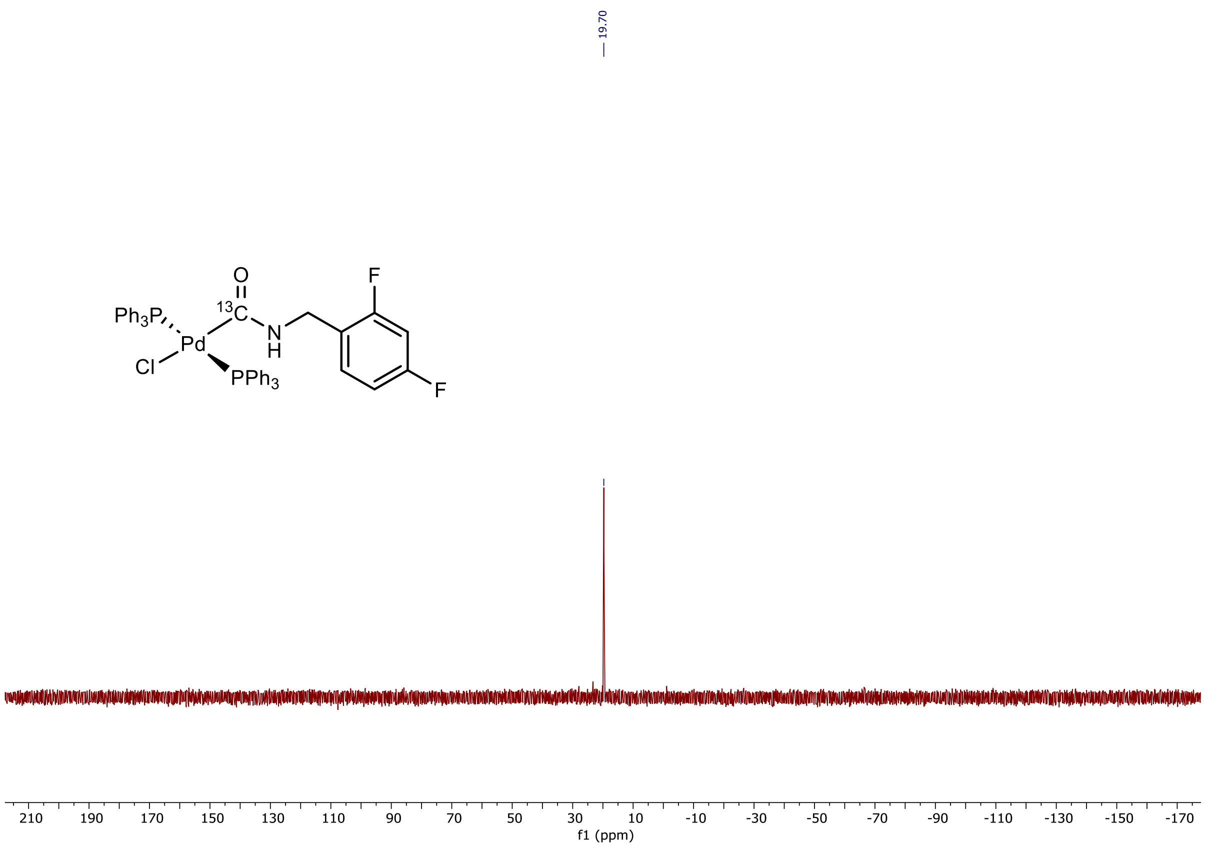

^{19}F-NMR

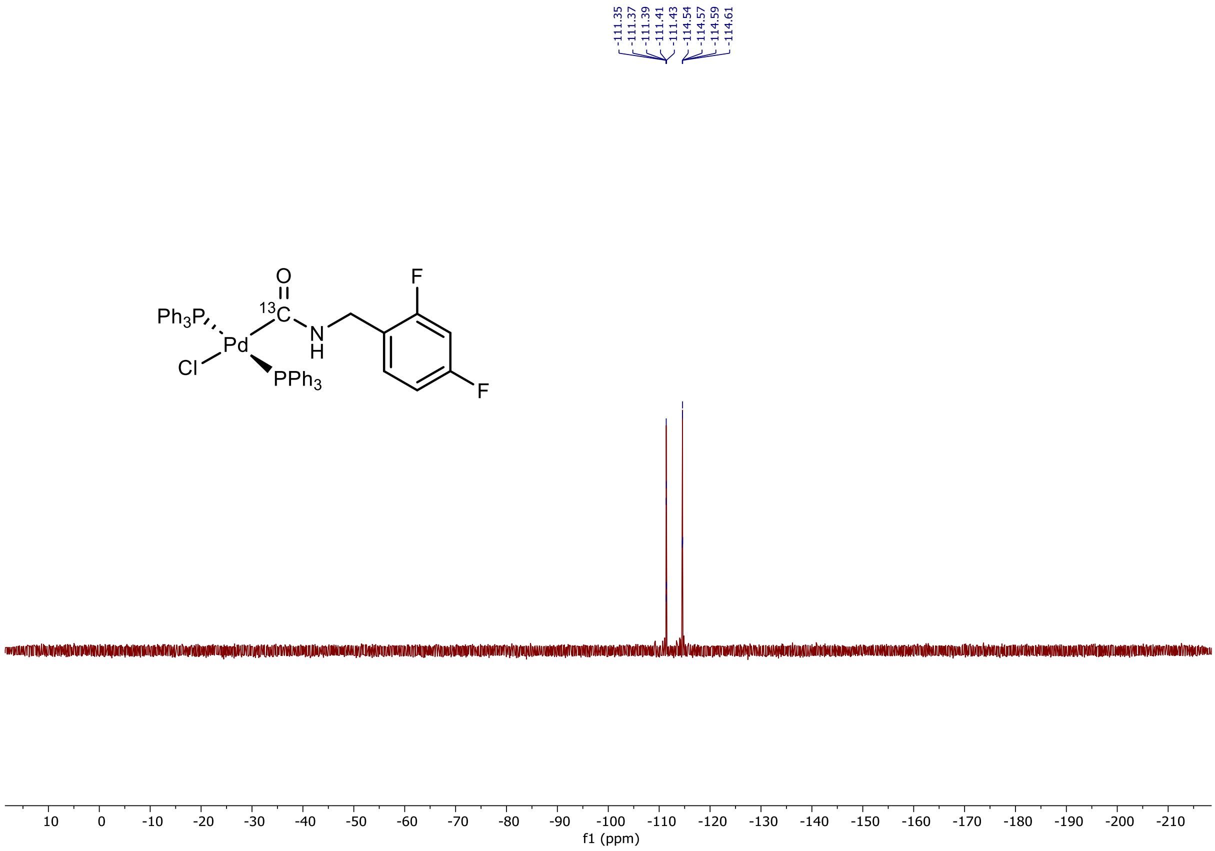

***trans*-Chloro(melthylaminecarbonyl)bis(triphenylphosphine) palladium(II) (Pd-4)**

^{1}H-NMR

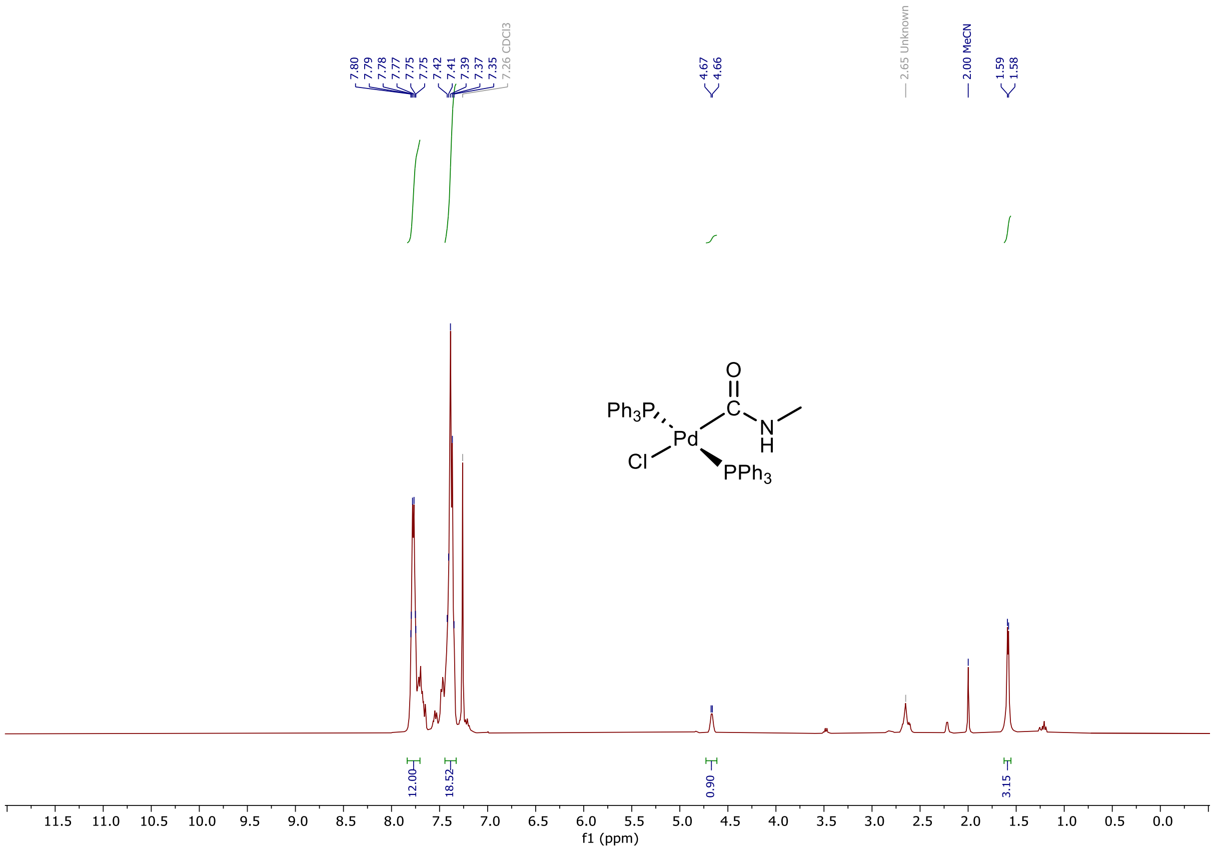

^{13}C-NMR

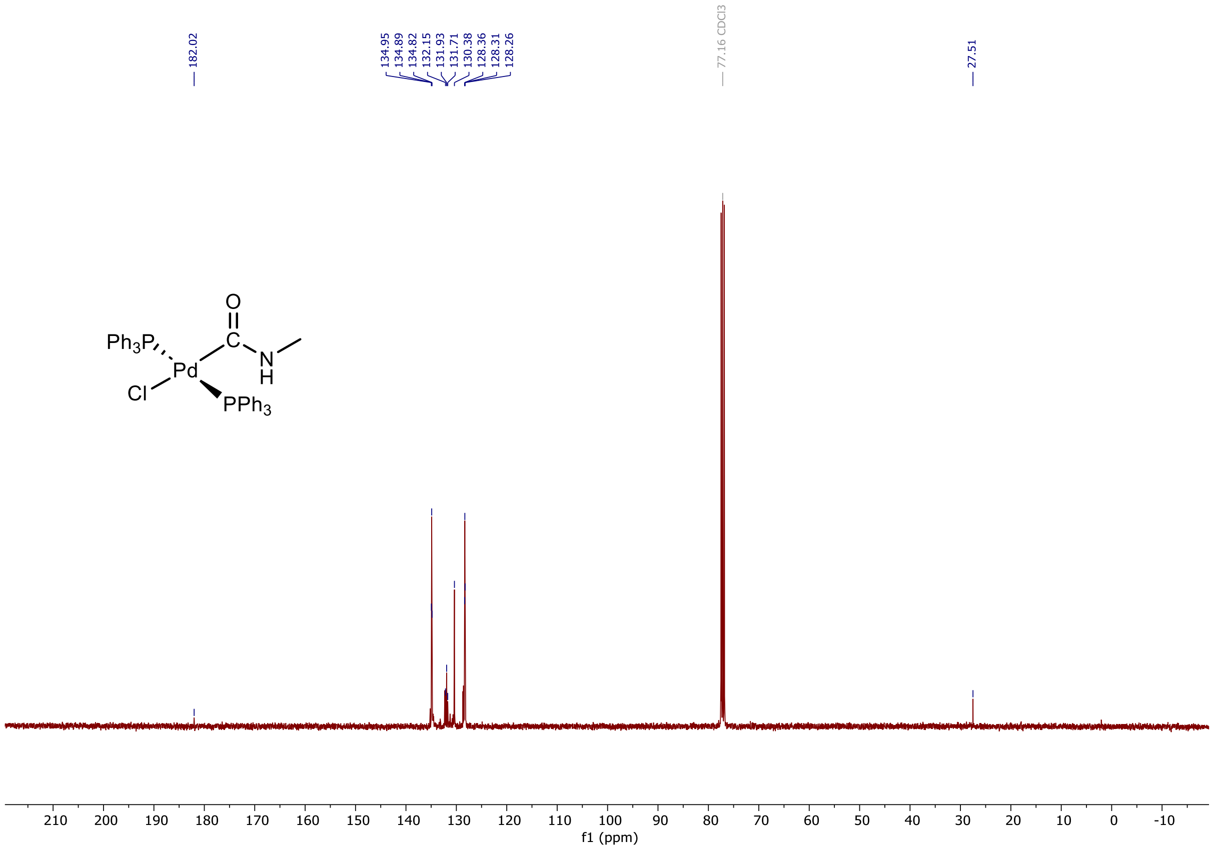

^{31}P-NMR

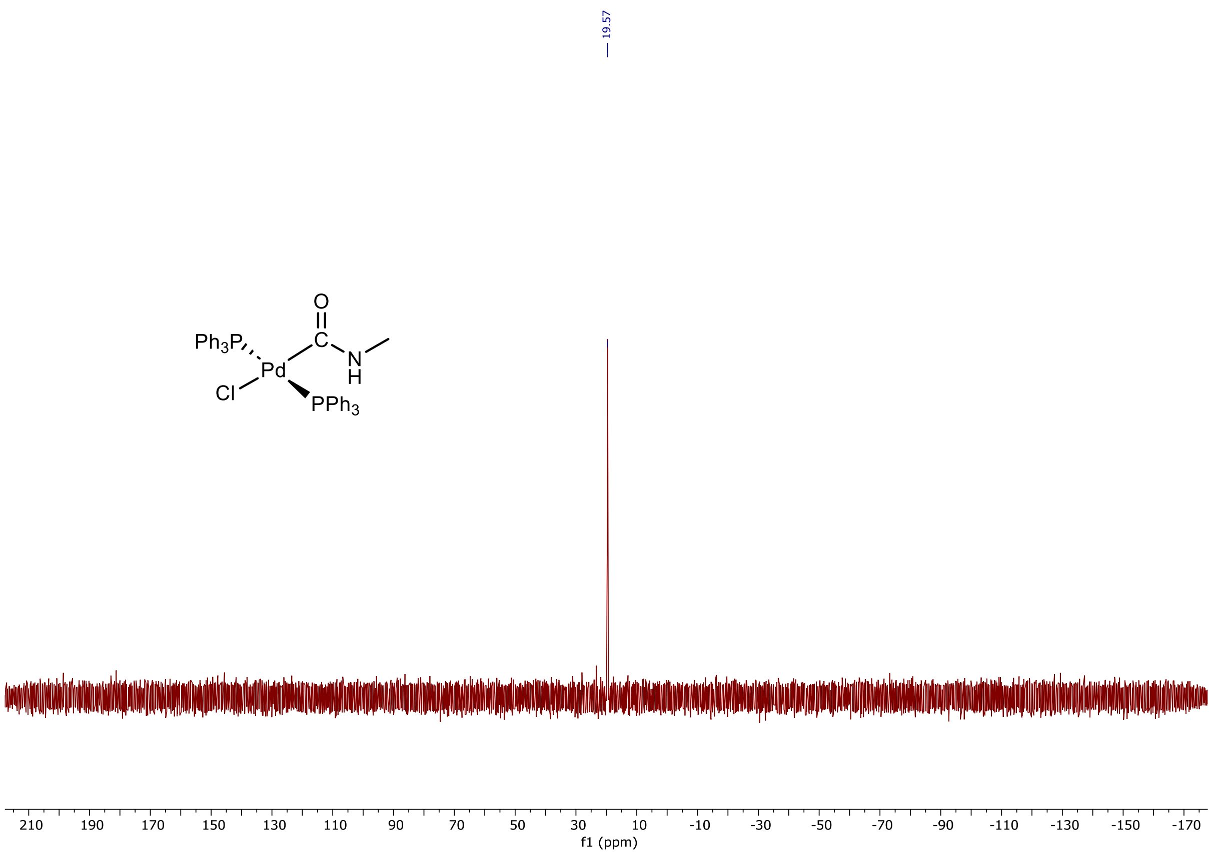
19.57
Ph3P
Pd
Cl
PPh3
O
C
N
H
210
190
170
150
130
110
90
70
50
30
10
-10
-30
-50
-70
-90
-110
-130
-150
-170
f1 (ppm)

trans-Chloro(melthylamine-^{13}C-carbonyl)bis(triphenylphosphine) palladium(II) (^{13}C-Pd-4)

^{1}H-NMR

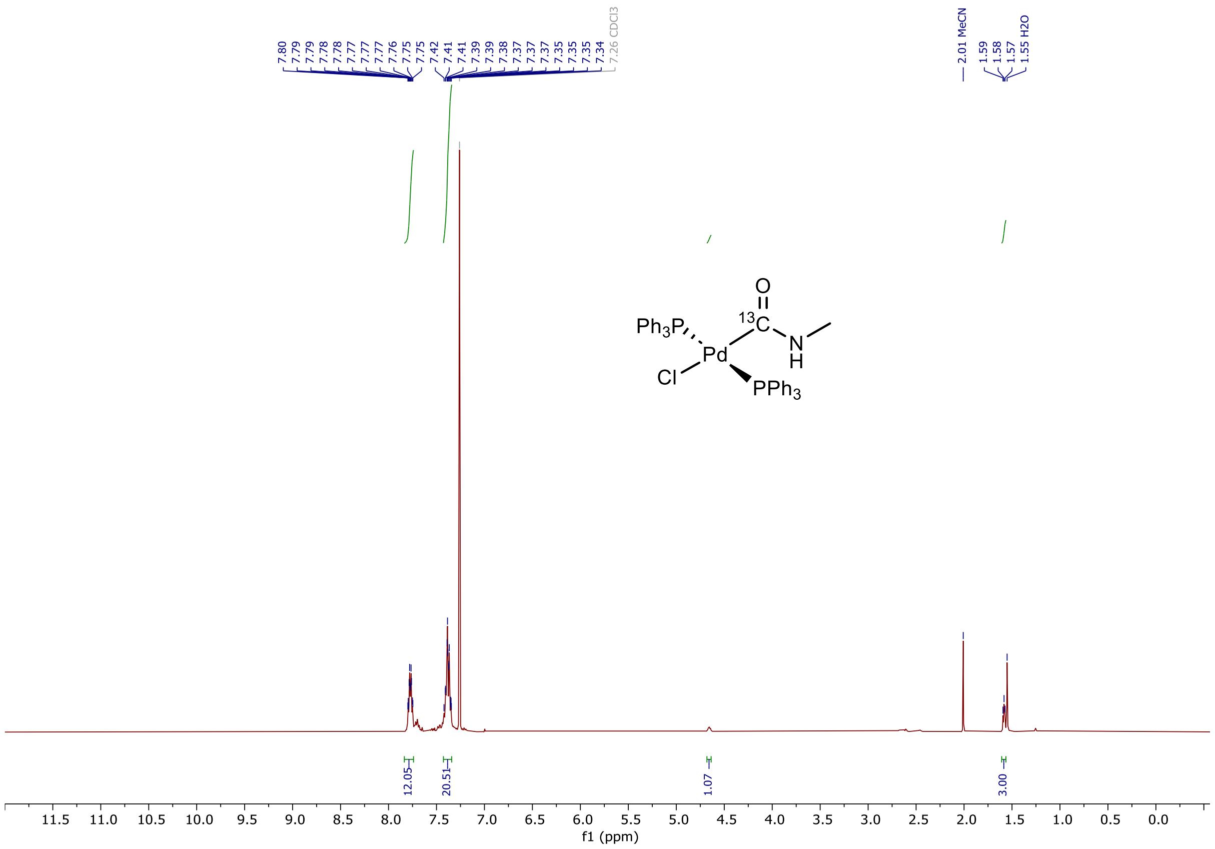

^{13}C-NMR

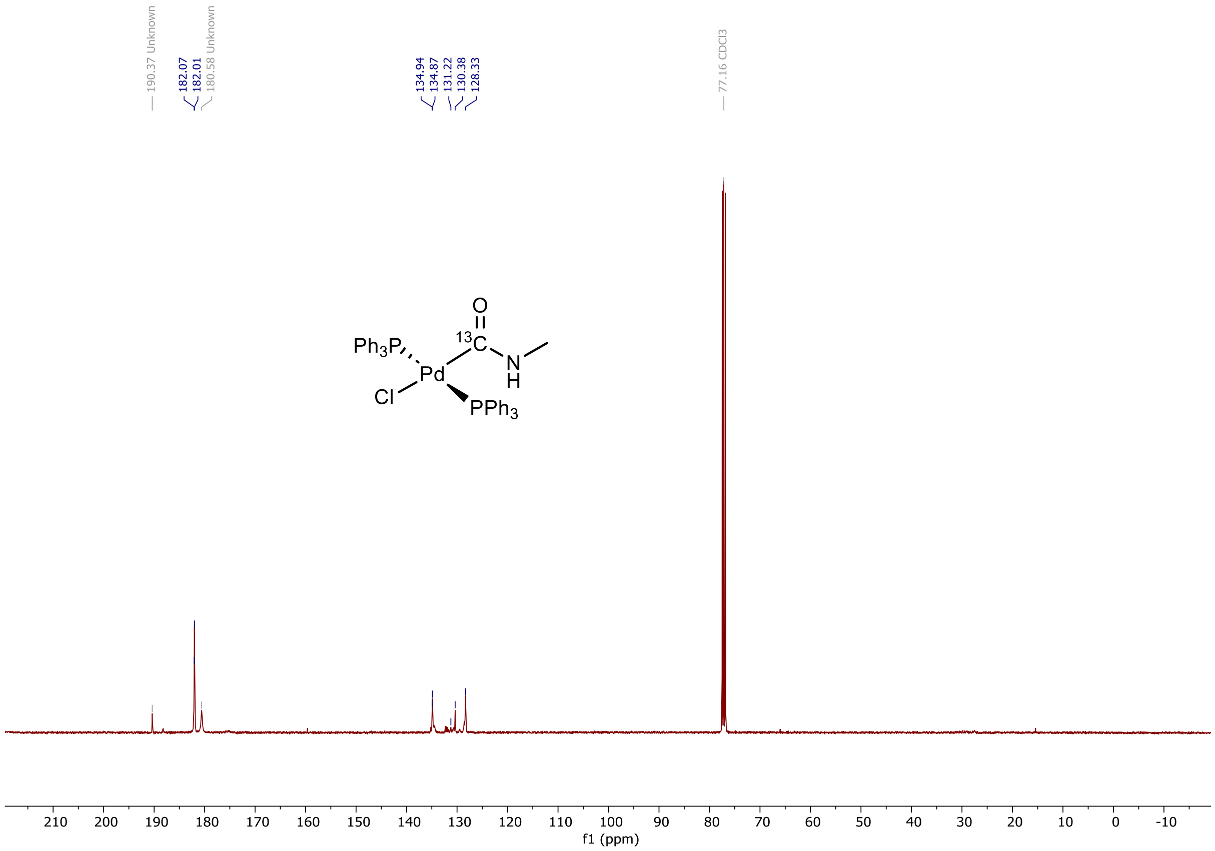

^{31}P-NMR

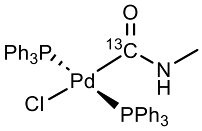

Ph3P
13C
O
N
H
Pd
Cl
PPh3

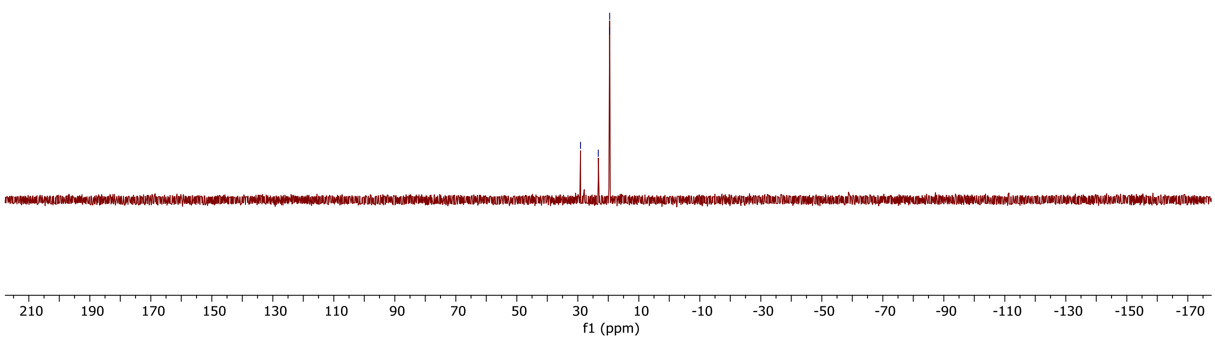

29.11 TPPO
23.27 SM
19.58
19.55
210 190 170 150 130 110 90 70 50 30 10 -10 -30 -50 -70 -90 -110 -130 -150 -170
f1 (ppm)

trans-Chloro(piperidinecarbonyl)bis(triphenylphosphine) palladium(II) (Pd-5)

^{1}H-NMR

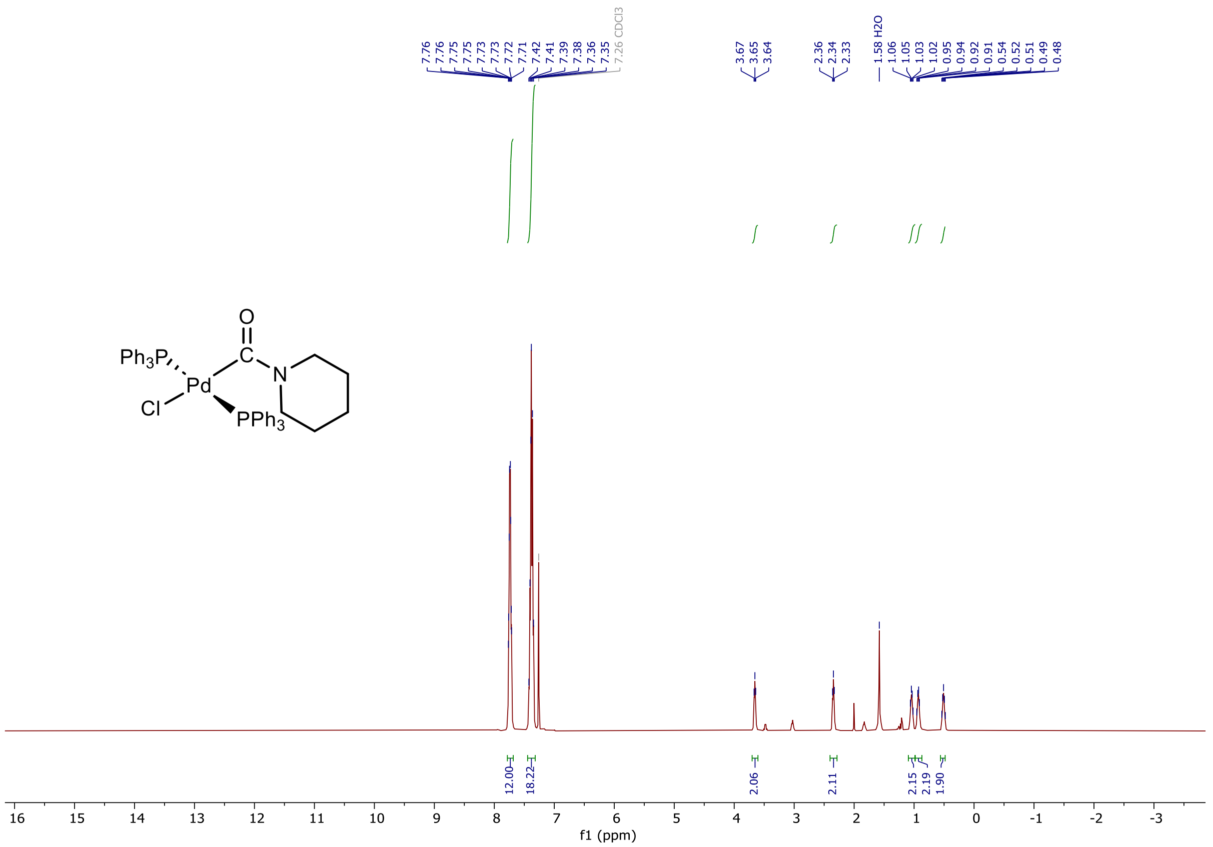

^{13}C-NMR

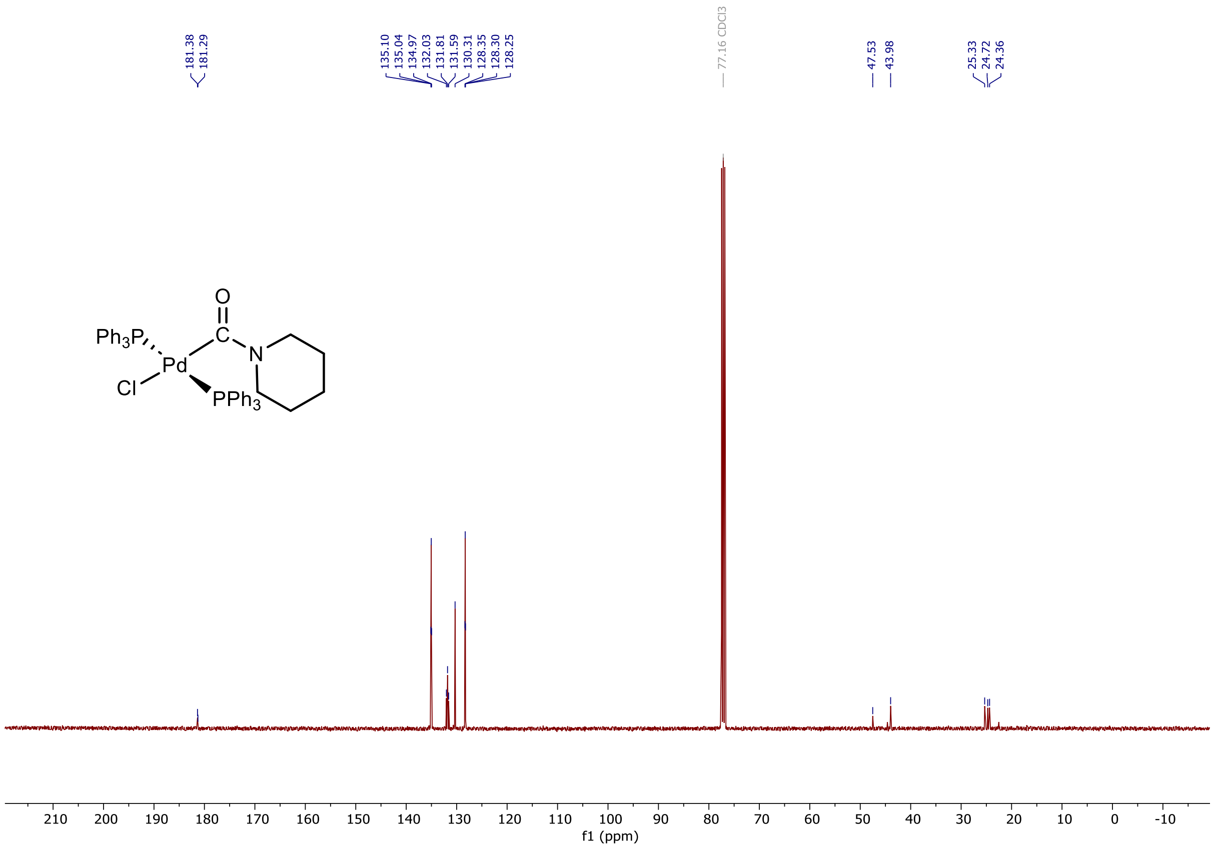

^{31}P-NMR

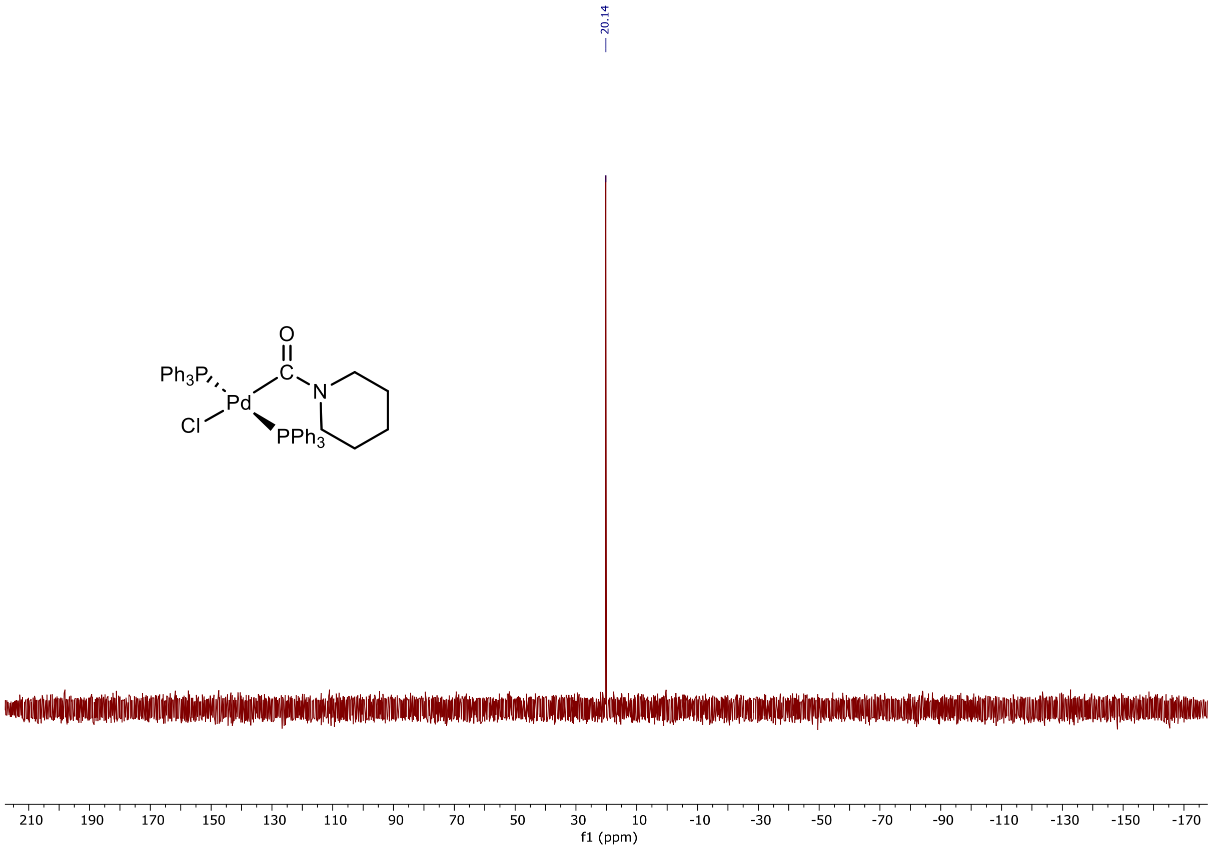

20.14
Ph3P
Pd
Cl
PPh3
O
C
N
210
190
170
150
130
110
90
70
50
30
10
-10
-30
-50
-70
-90
-110
-130
-150
-170
f1 (ppm)

trans-Chloro(piperidine-^{13}C-carbonyl)bis(triphenylphosphine) palladium(II) (^{13}C-Pd-5)

^{1}H-NMR

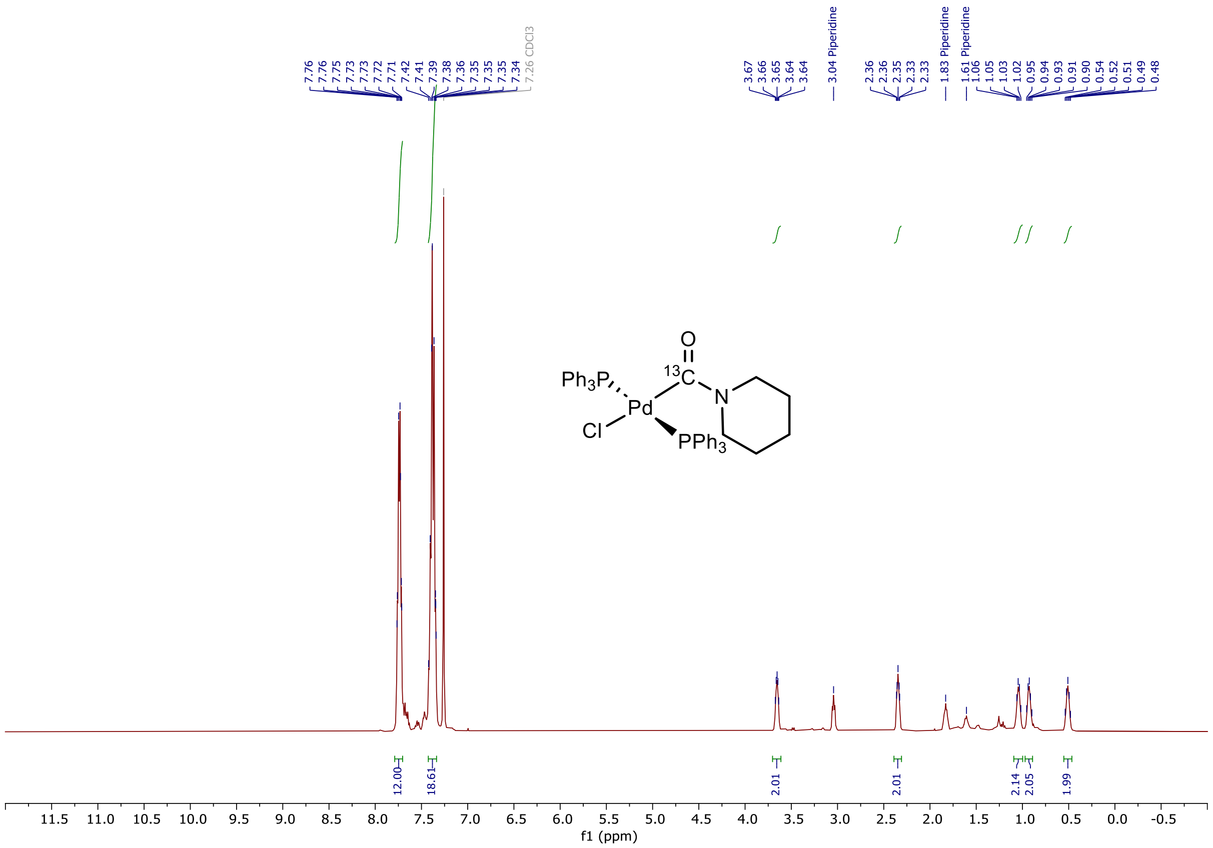

^{13}C-NMR

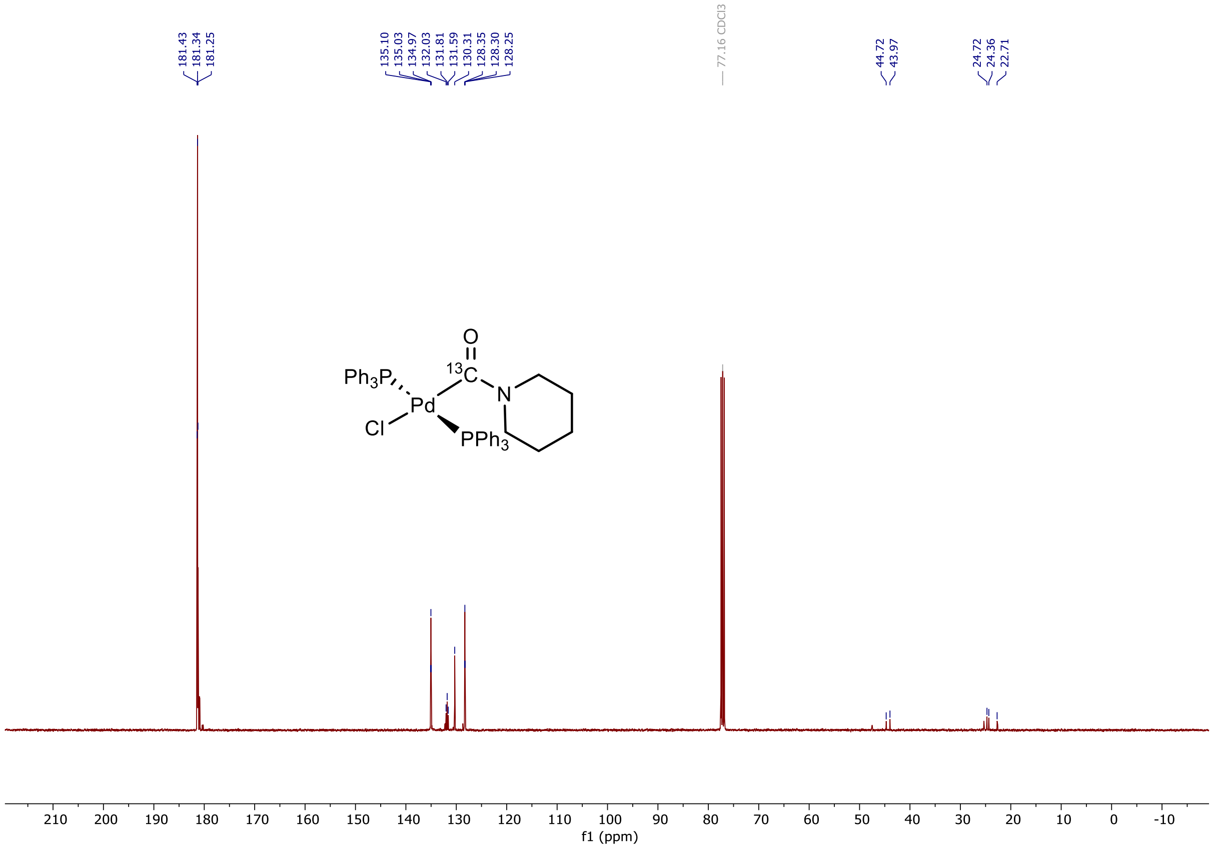

^{31}P-NMR

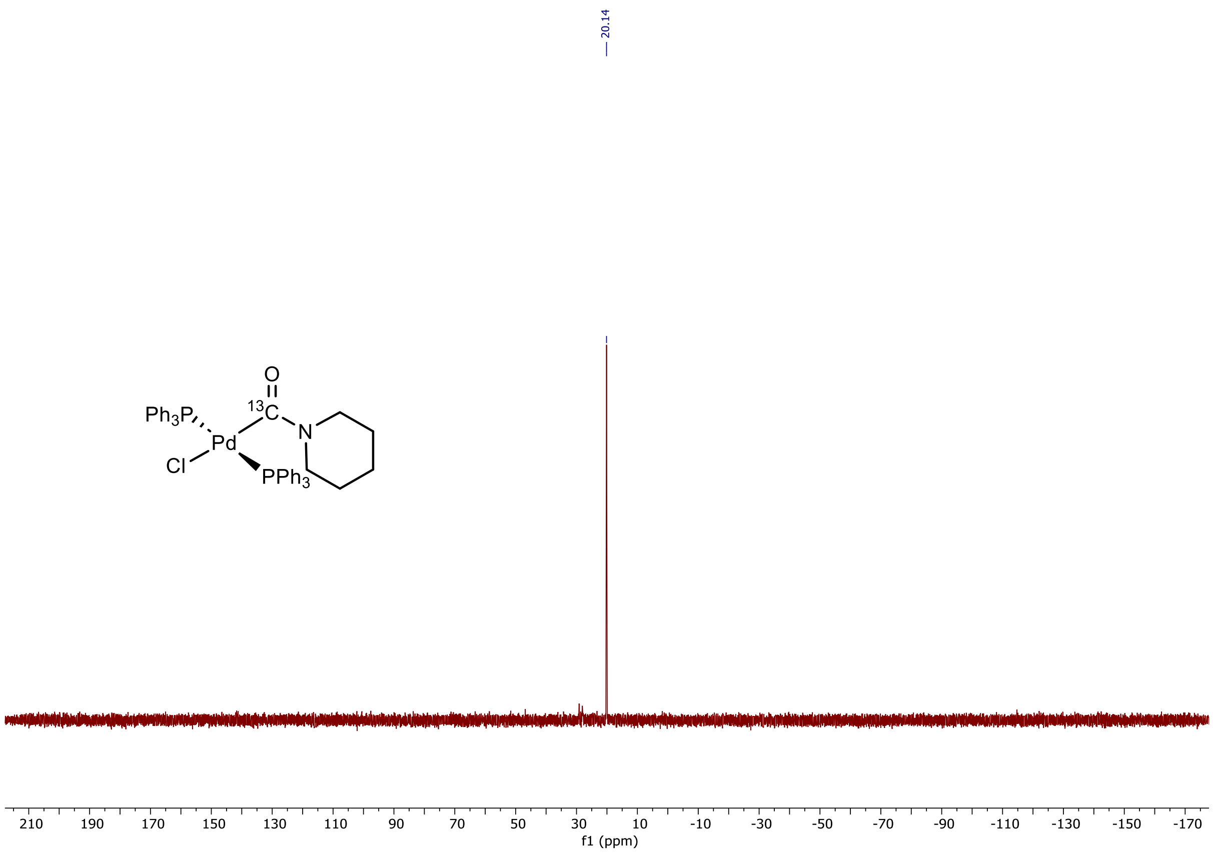

20.14
Ph3P
Pd
Cl
PPh3
13C
O
N
210
190
170
150
130
110
90
70
50
30
10
-10
-30
-50
-70
-90
-110
-130
-150
-170
f1 (ppm)

trans-Chloro(morpholinecarbonyl)bis(triphenylphosphine) palladium(II) (Pd-6)

^{1}H-NMR

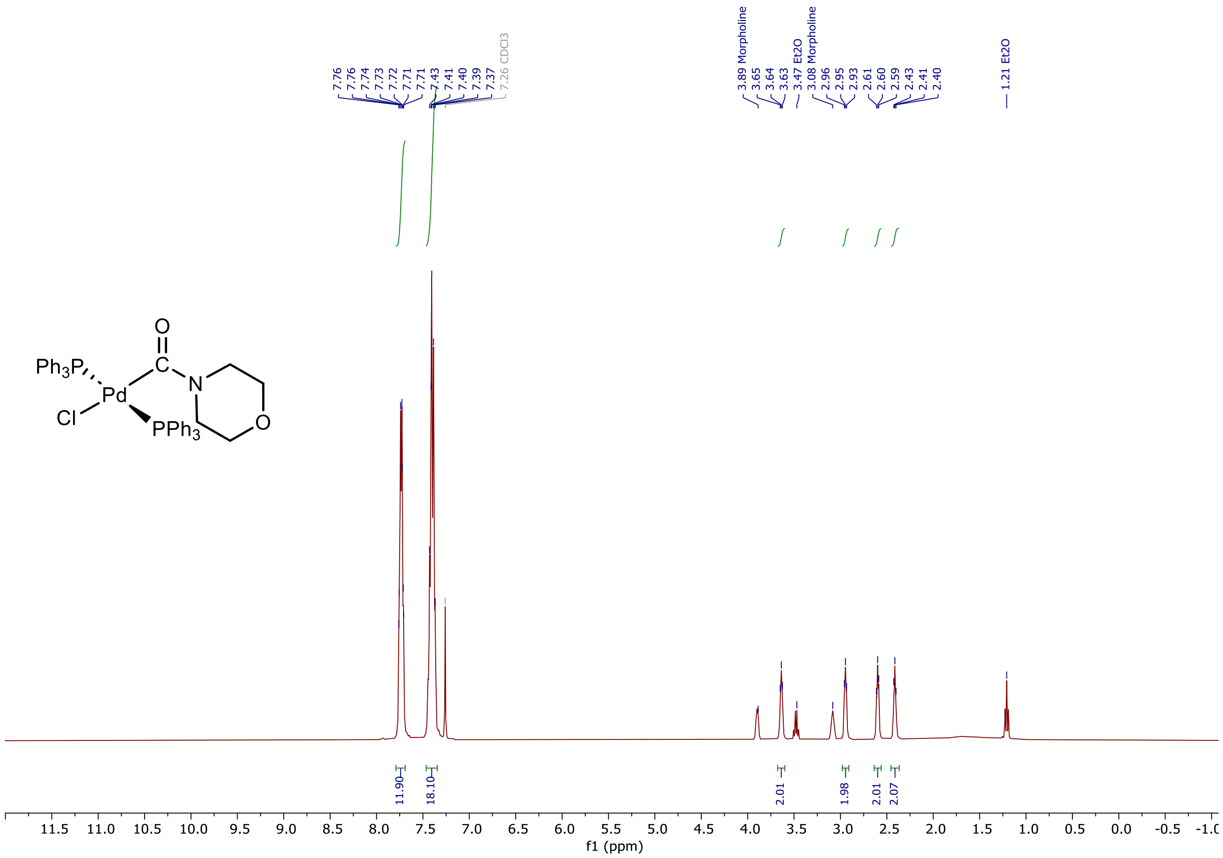

^{13}C-NMR

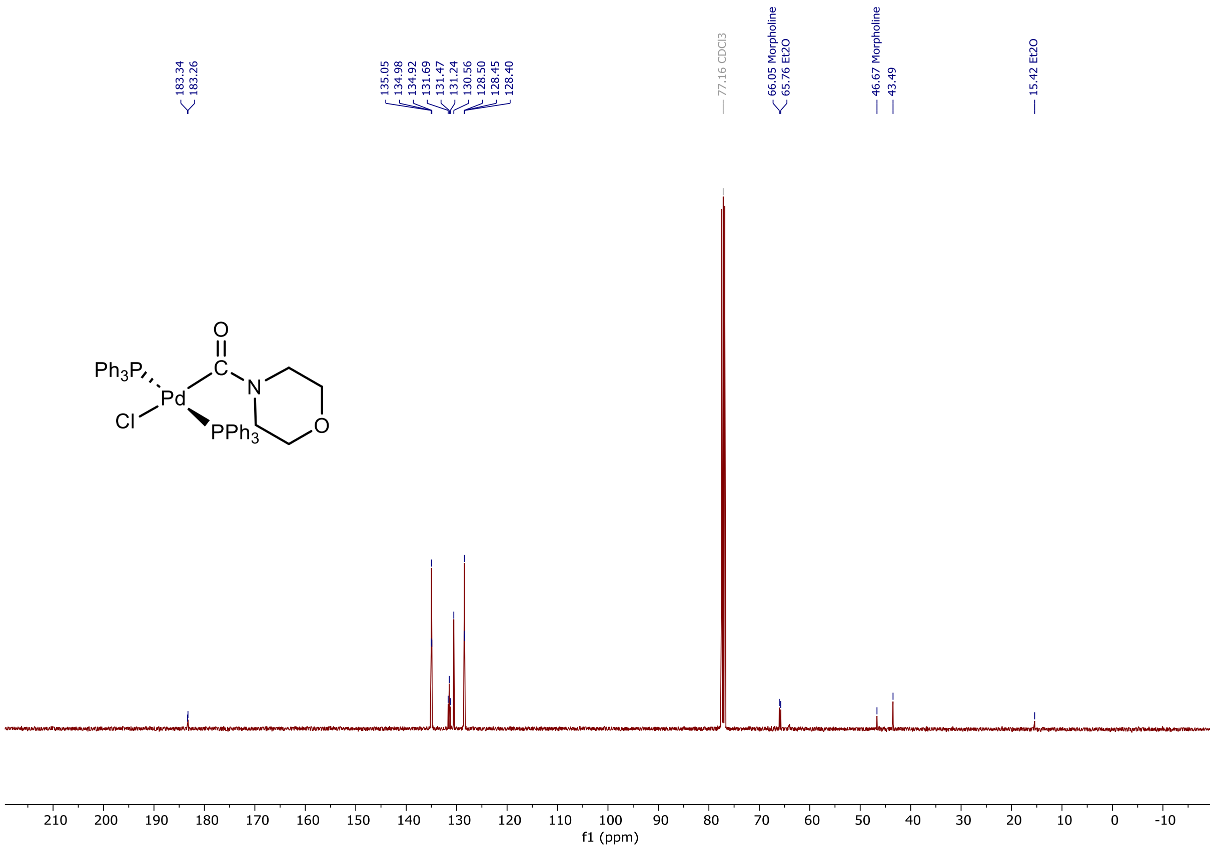

^{31}P-NMR

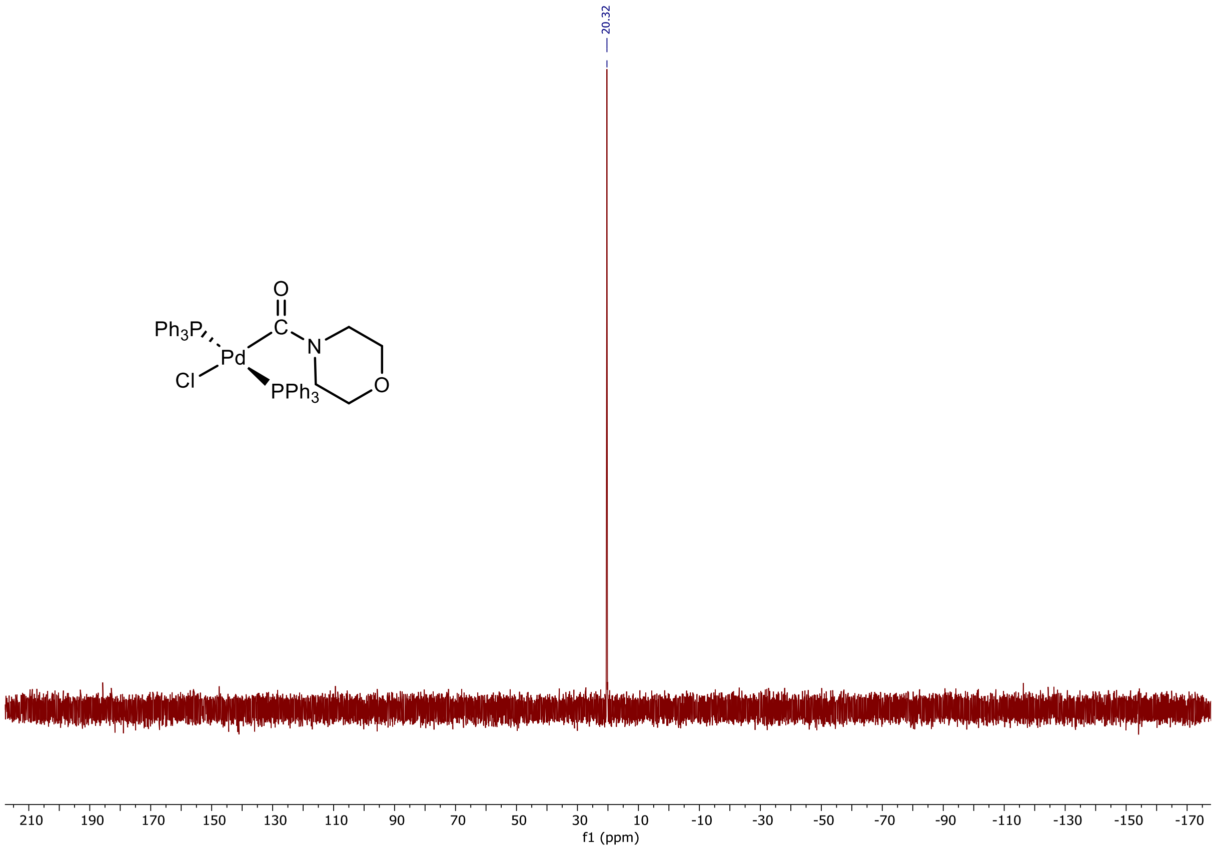

20.32
Ph3P
Pd
Cl
PPh3
O
C
N
O
210
190
170
150
130
110
90
70
50
30
10
-10
-30
-50
-70
-90
-110
-130
-150
-170
f1 (ppm)

***trans*-Chloro(cyclopropyl(4l²-piperazin-1-yl)methanonecarbonyl)bis(triphenylphosphine) palladium(II) (Pd-7)**

^{1}H-NMR

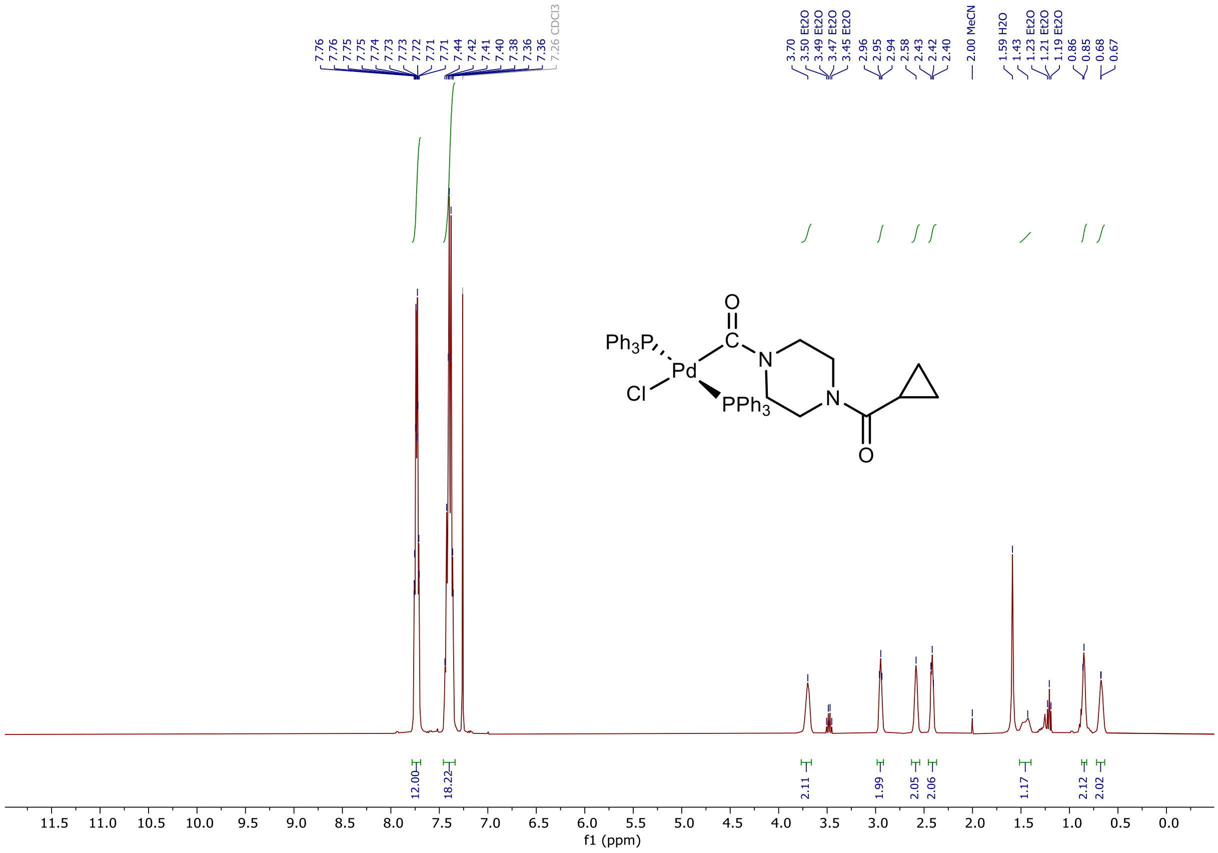

^{13}C-NMR

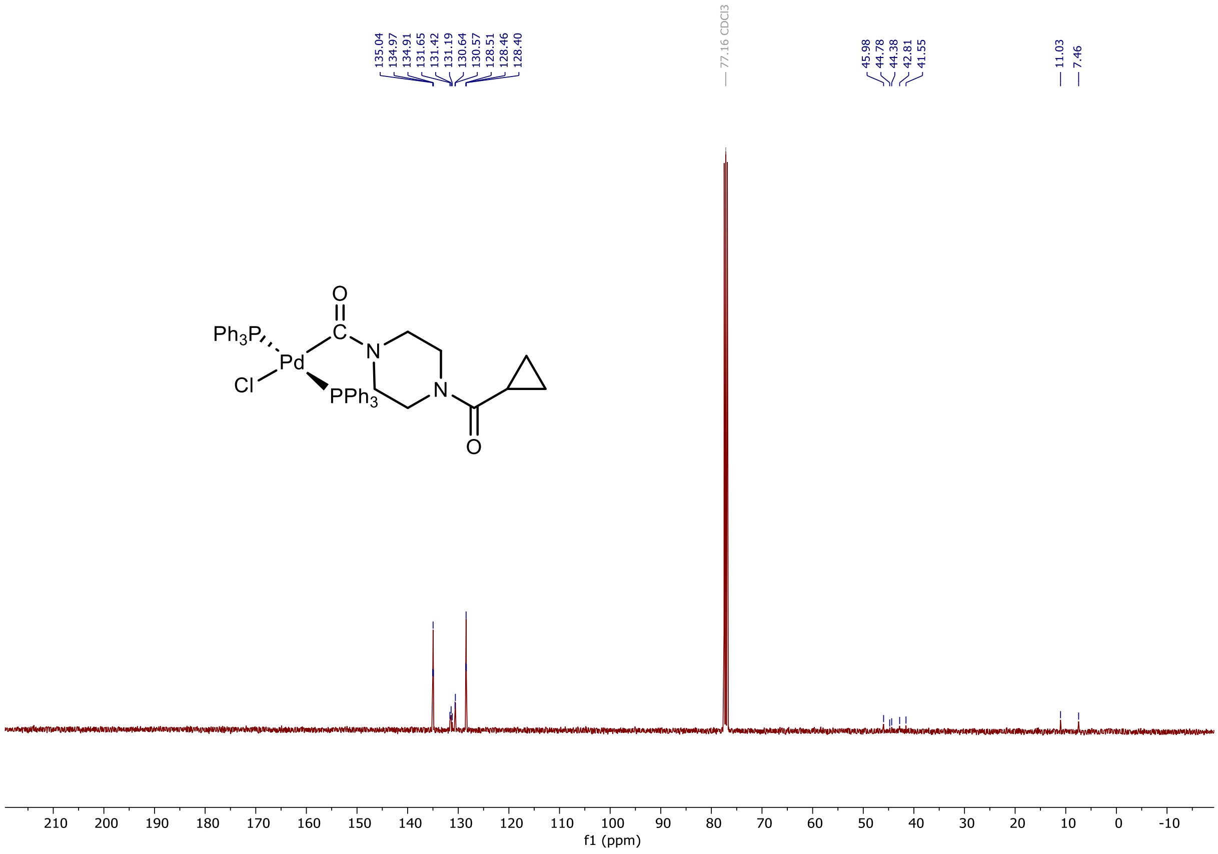

^{31}P-NMR

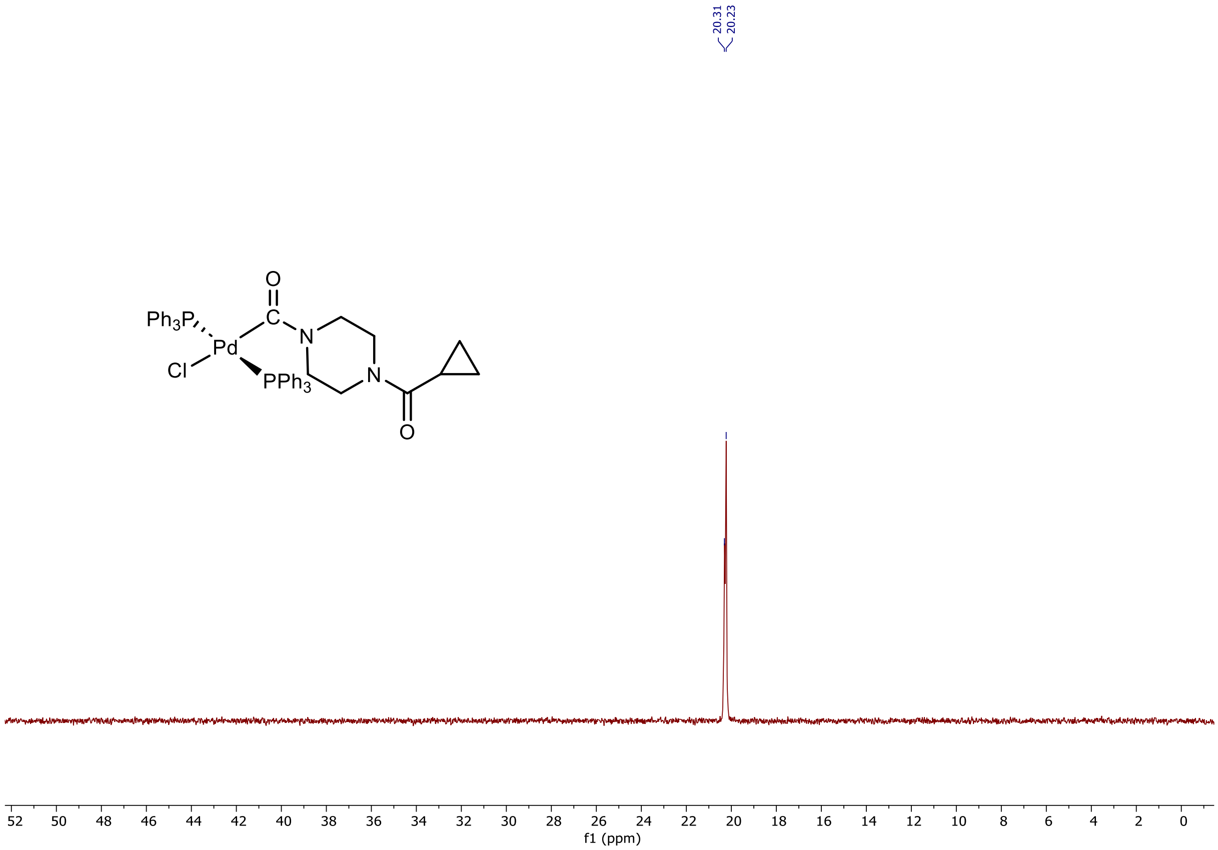
20.31
20.23
Ph3P
Pd
Cl
PPh3
C
O
N
N
O
52 50 48 46 44 42 40 38 36 34 32 30 28 26 24 22 20 18 16 14 12 10 8 6 4 2 0
f1 (ppm)

***trans*-Chloro(cyclopropyl(4l²-piperazin-1-yl)methanone-^{13}C-carbonyl)bis(triphenylphosphine) palladium(II) (^{13}C-Pd-7)**

^{1}H-NMR

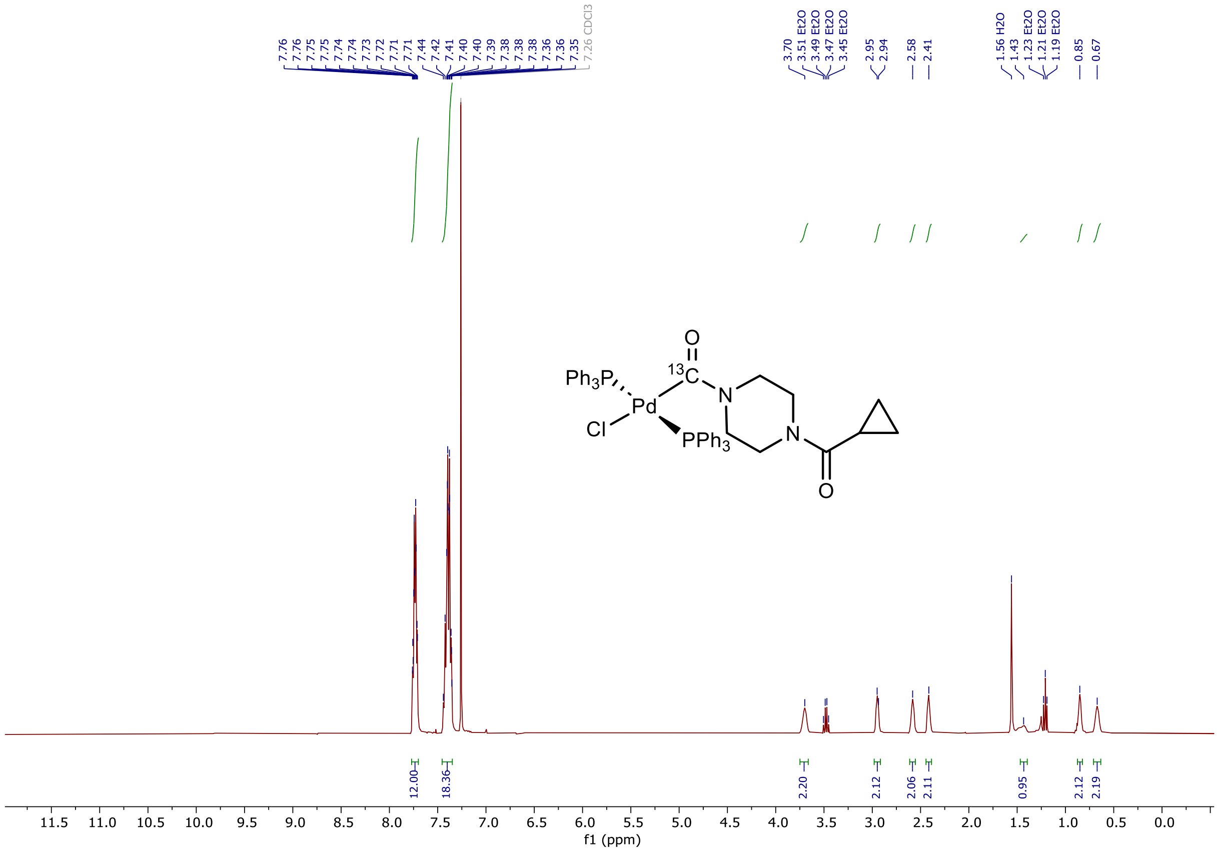

^{13}C-NMR

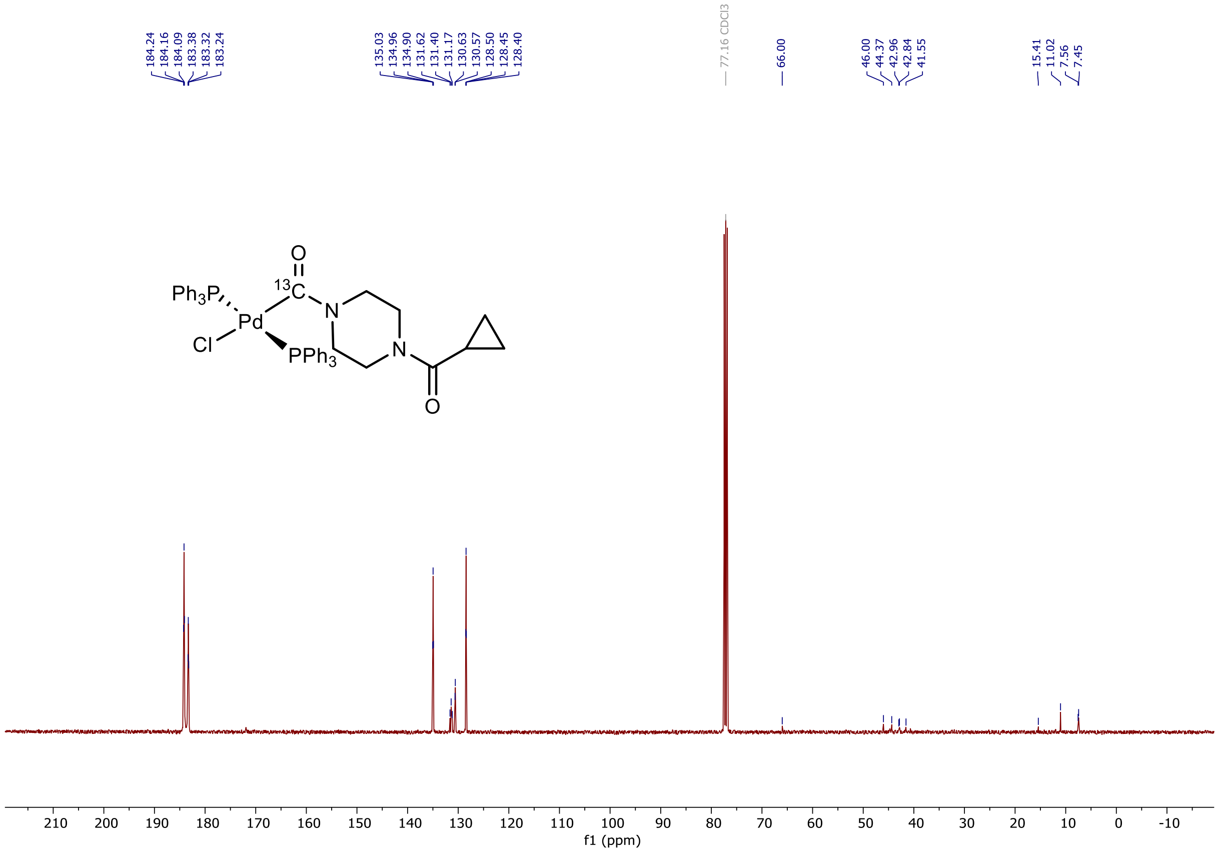

^{31}P-NMR

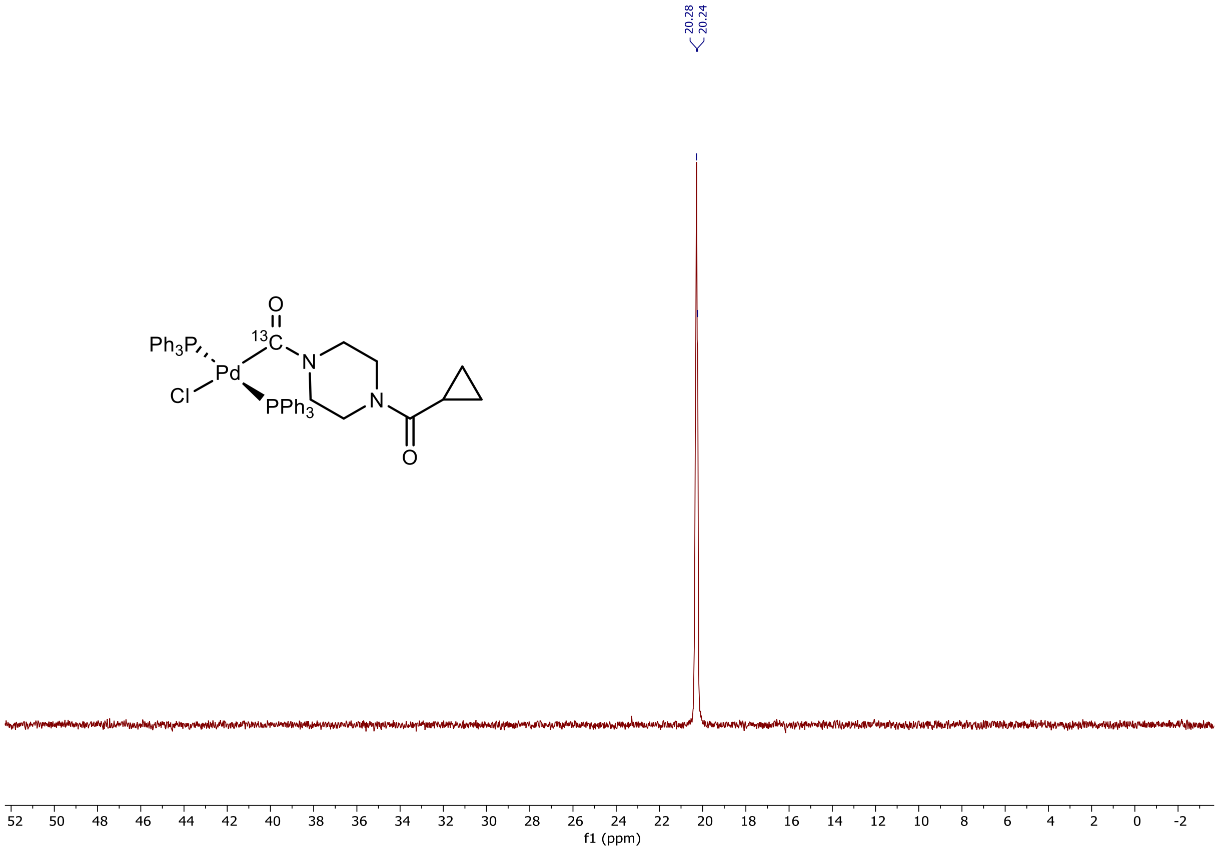

20.28
20.24
Ph3P
Pd
Cl
PPh3
13C
O
N
N
O
52 50 48 46 44 42 40 38 36 34 32 30 28 26 24 22 20 18 16 14 12 10 8 6 4 2 0 -2
f1 (ppm)

***trans*-Chloro(prop-2-yn-1-ylaminecarbonyl)bis(triphenylphosphine) palladium(II) (Pd-8)**

^{1}H-NMR

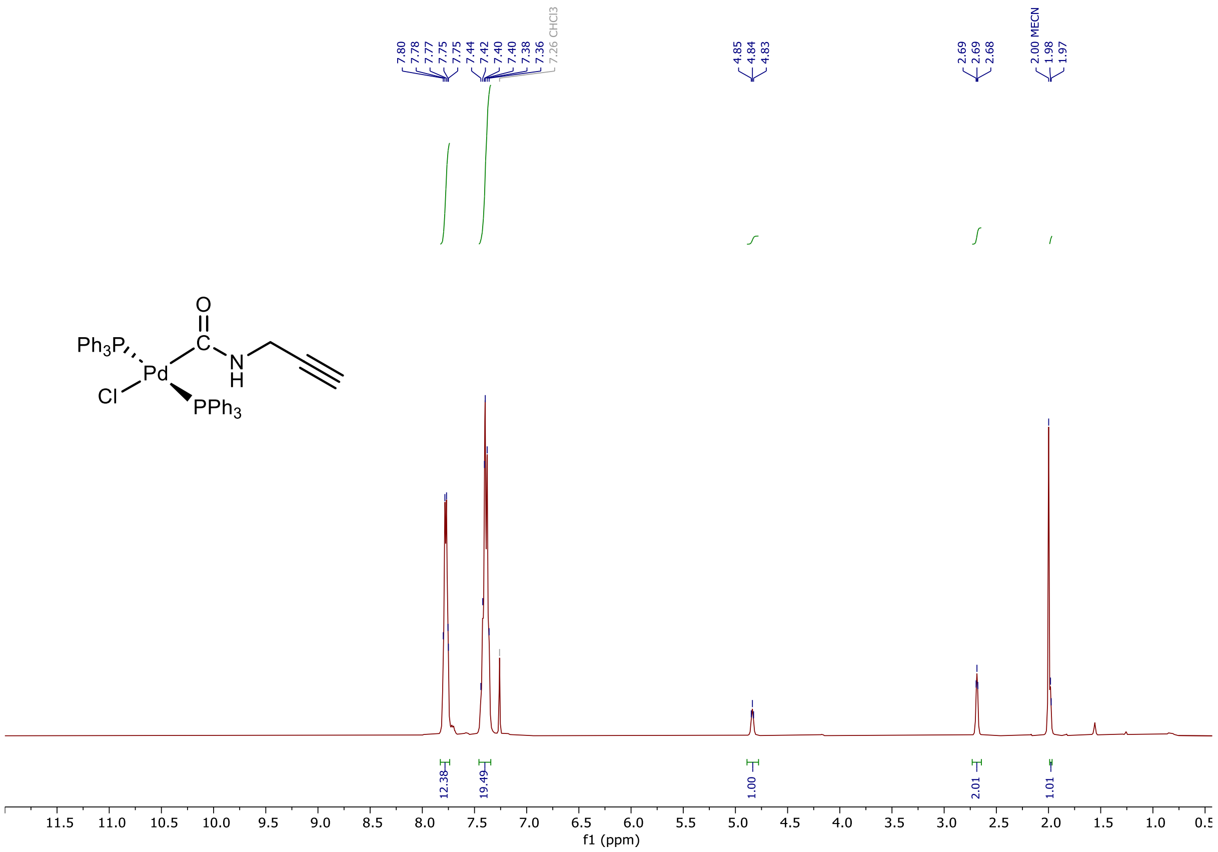

^{13}C-NMR

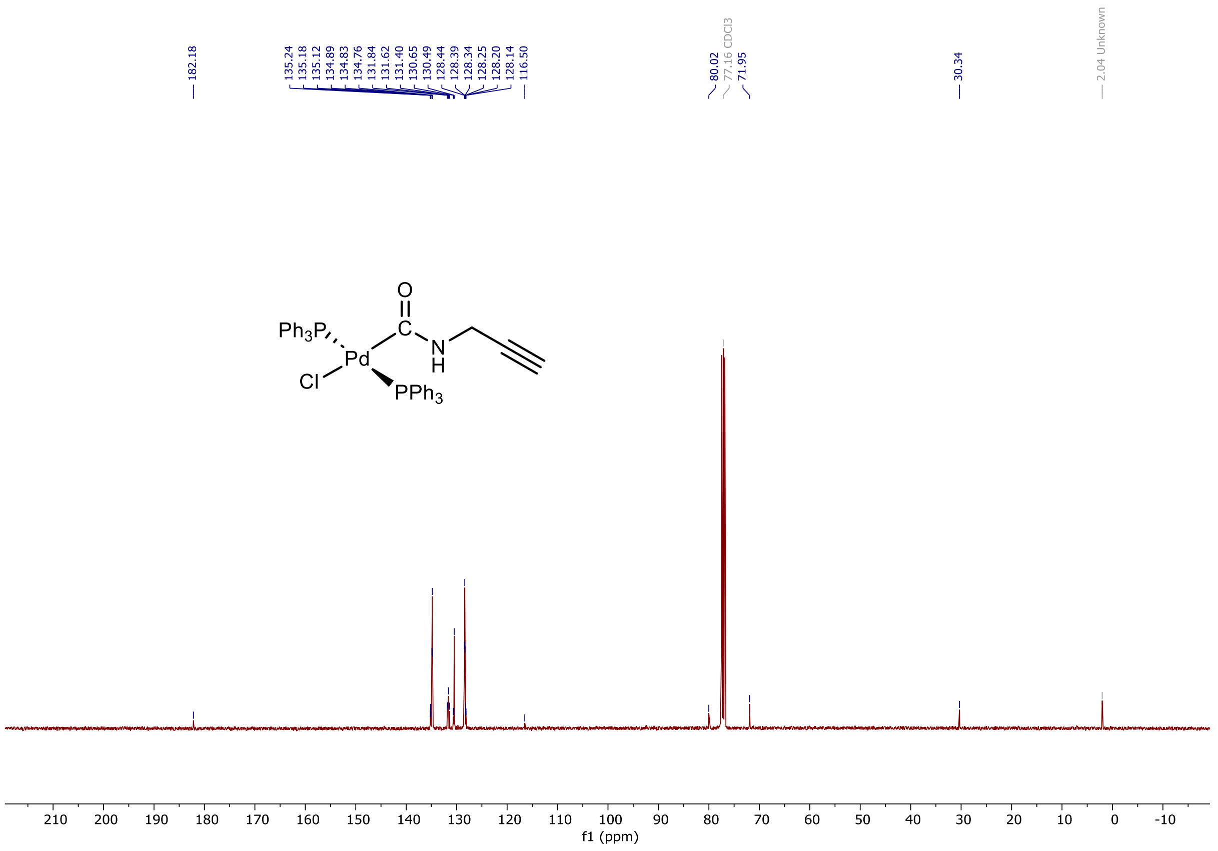

^{31}P-NMR

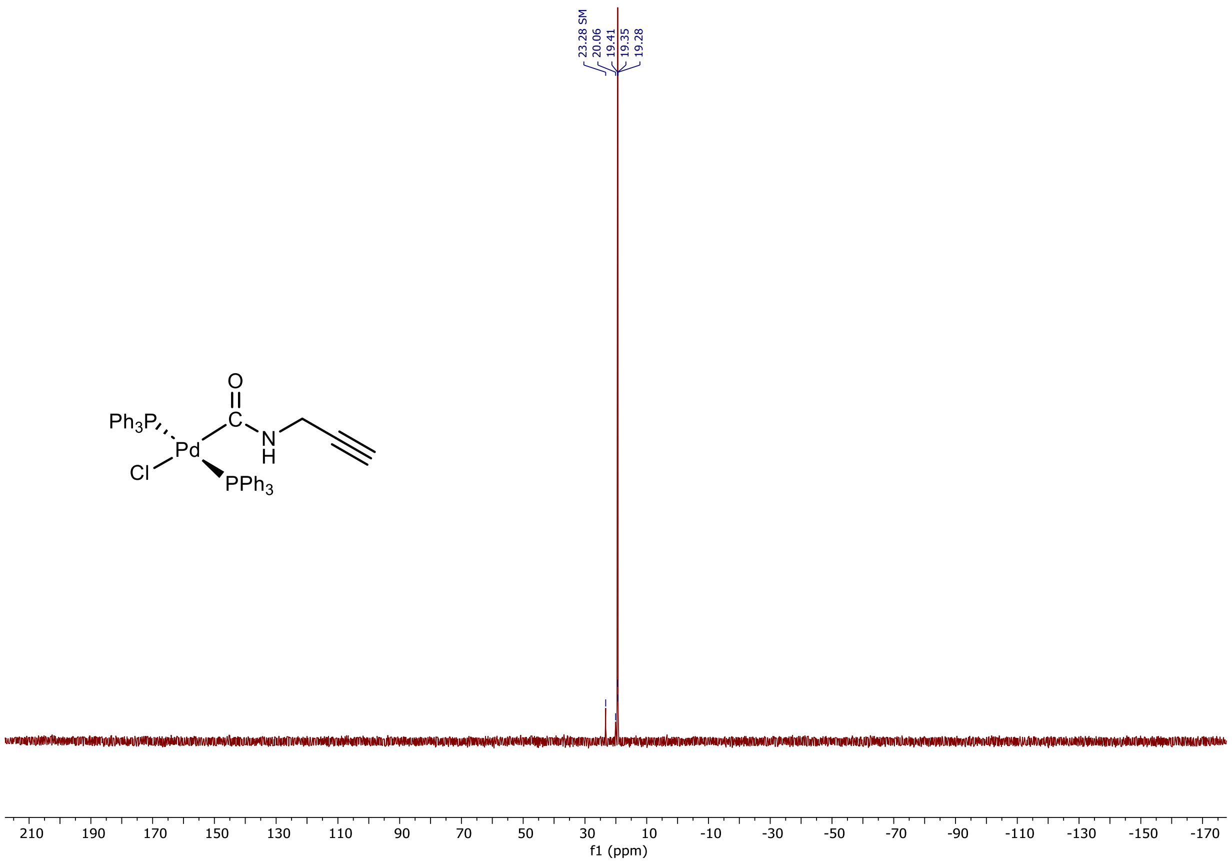

23.28 SM
20.06
19.41
19.35
19.28
Ph3P
Pd
Cl
PPh3
O
C
N
H
210
190
170
150
130
110
90
70
50
30
10
-10
-30
-50
-70
-90
-110
-130
-150
-170
f1 (ppm)

***trans*-Chloro(2-chloro-11-(piperazin-1-yl)dibenzo[*b,f*][1,4]oxazepine)carbonyl)bis(triphenylphosphine) palladium(II) (Pd-10)**

^{1}H-NMR

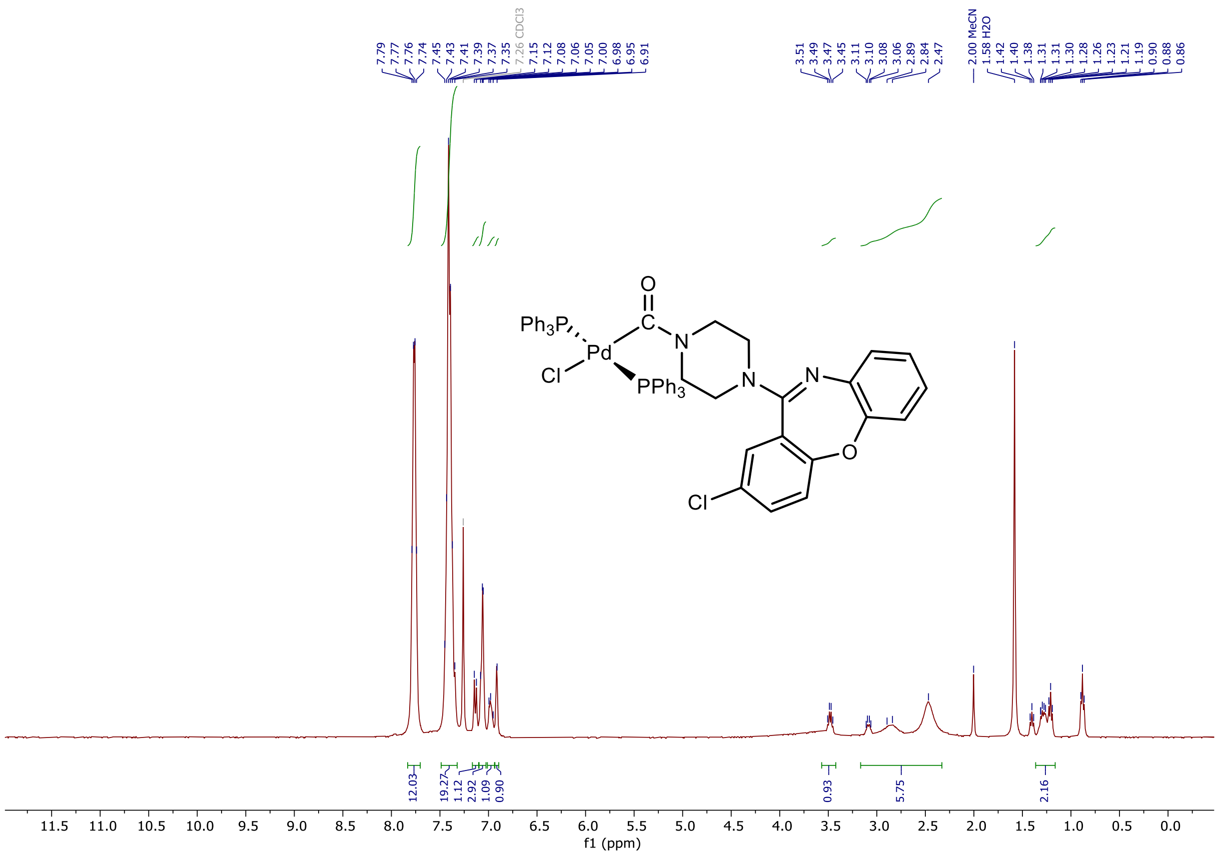

^{13}C-NMR

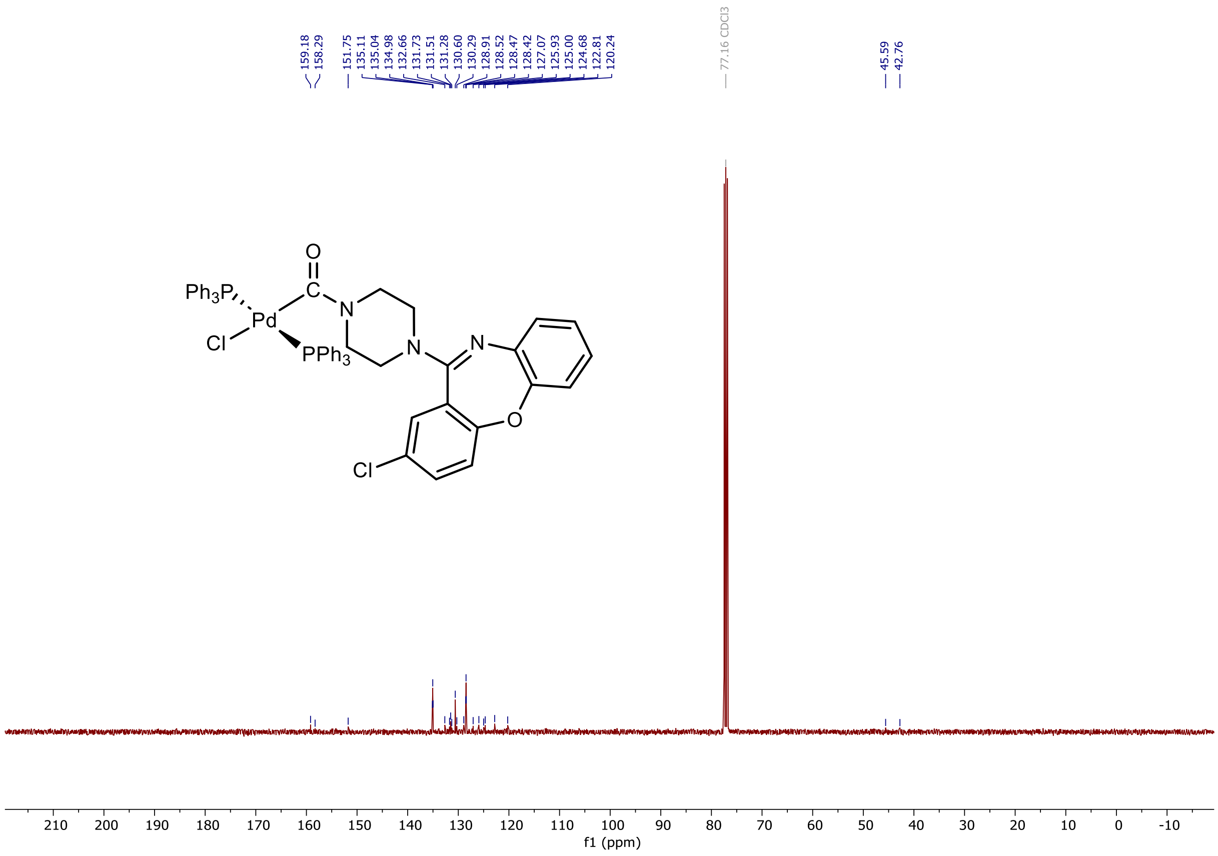

^{31}P-NMR

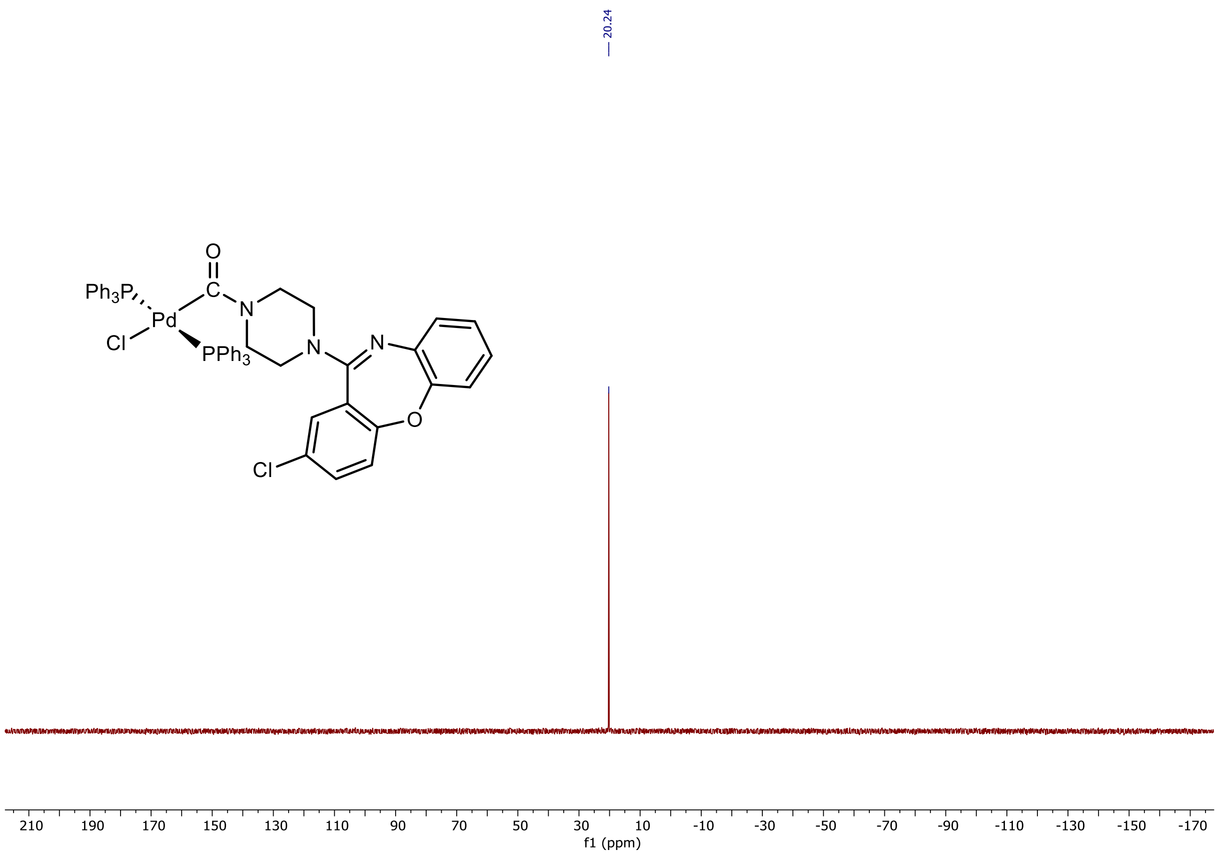

20.24
Ph3P
Pd
Cl
PPh3
O
C
N
N
N
O
Cl
210
190
170
150
130
110
90
70
50
30
10
-10
-30
-50
-70
-90
-110
-130
-150
-170
f1 (ppm)

***trans*-Chloro(2-chloro-11-(piperazin-1-yl)dibenzo[*b,f*][1,4]oxazepine)-^{13}C-carbonyl)bis(tri-phenylphosphine) palladium(II) (^{13}C-Pd-10)**

^{1}H-NMR

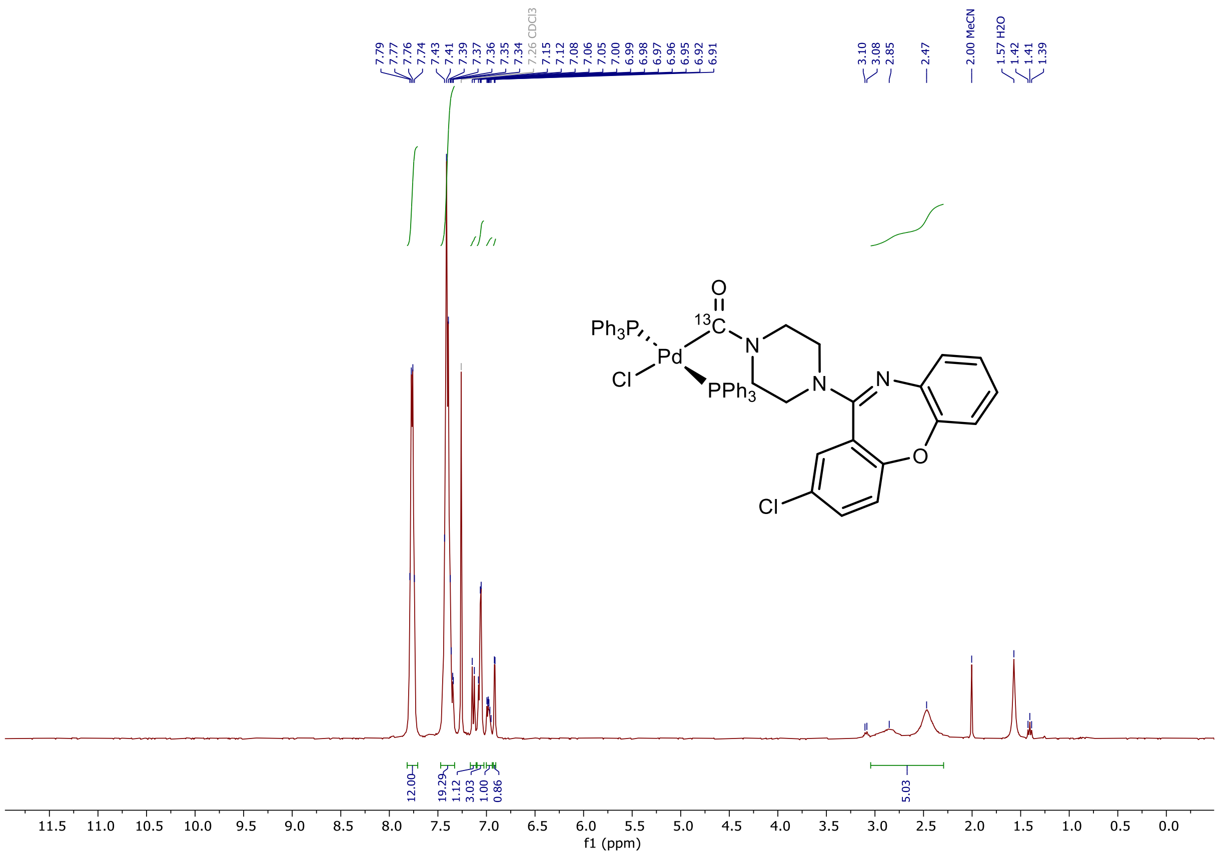

^{13}C-NMR

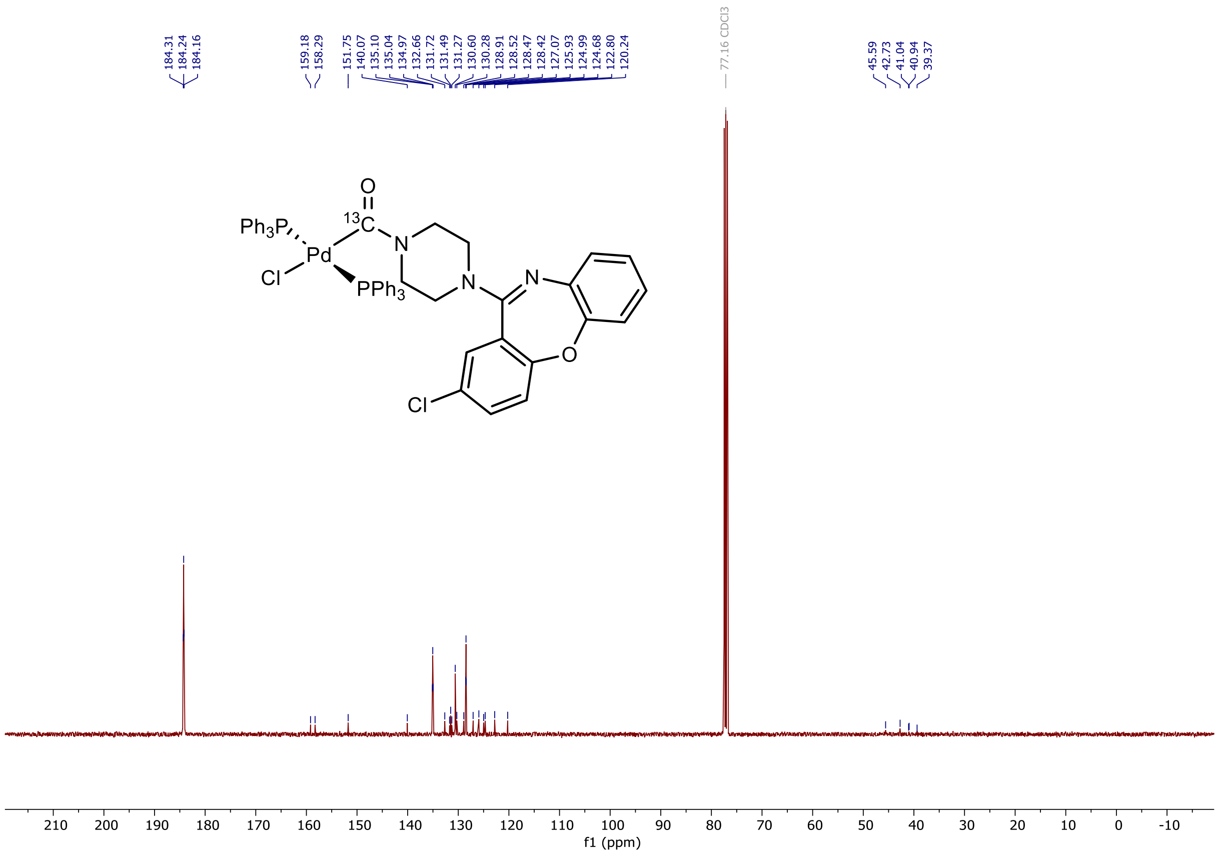

^{31}P-NMR

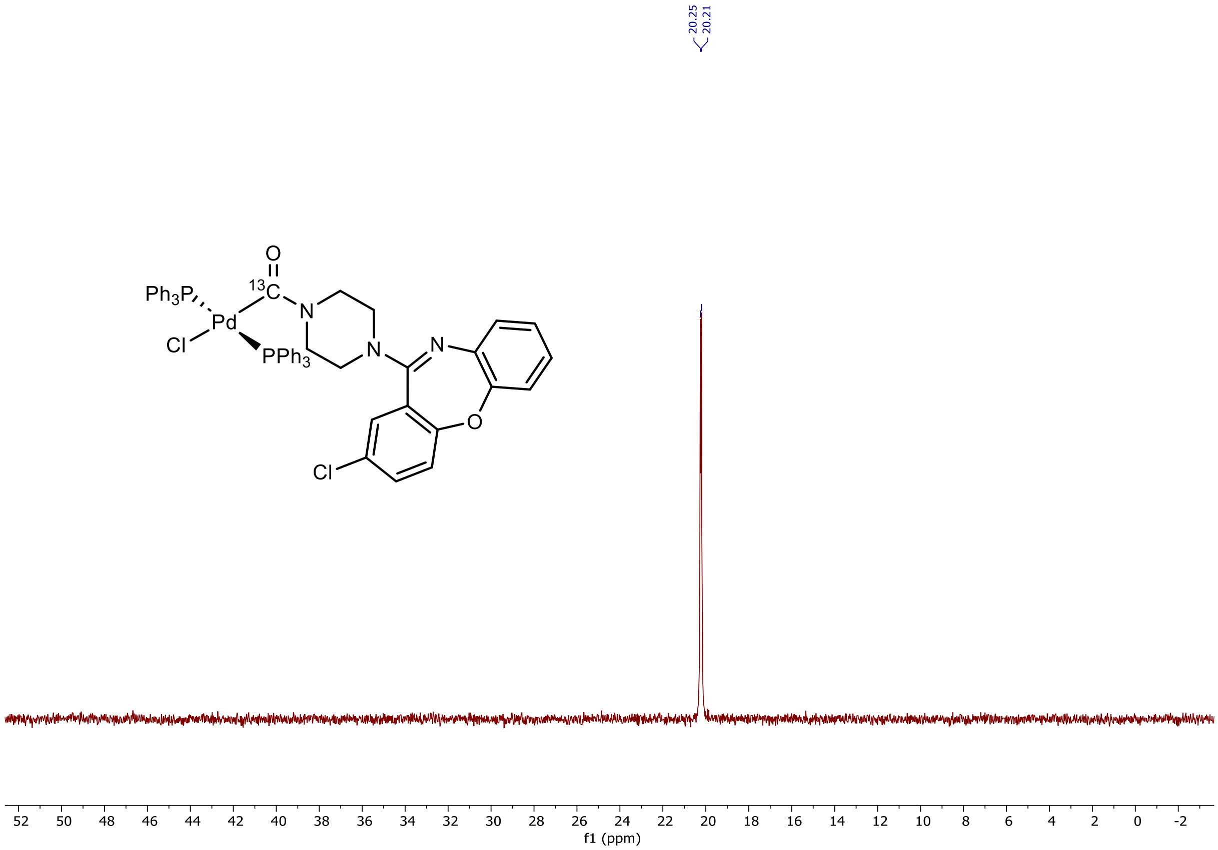
20.25
20.21
Ph3P
Pd
Cl
PPh3
13C
O
N
N
N
O
Cl
52 50 48 46 44 42 40 38 36 34 32 30 28 26 24 22 20 18 16 14 12 10 8 6 4 2 0 -2
f1 (ppm)

***trans*-Chloro(2-chloro-11-(piperazin-1-yl)dibenzo[*b,f*][1,4]oxazepine)-^{14}C-carbonyl)bis(triphenylphosphine) palladium(II) (^{14}C-Pd-10)**

^{1}H-NMR

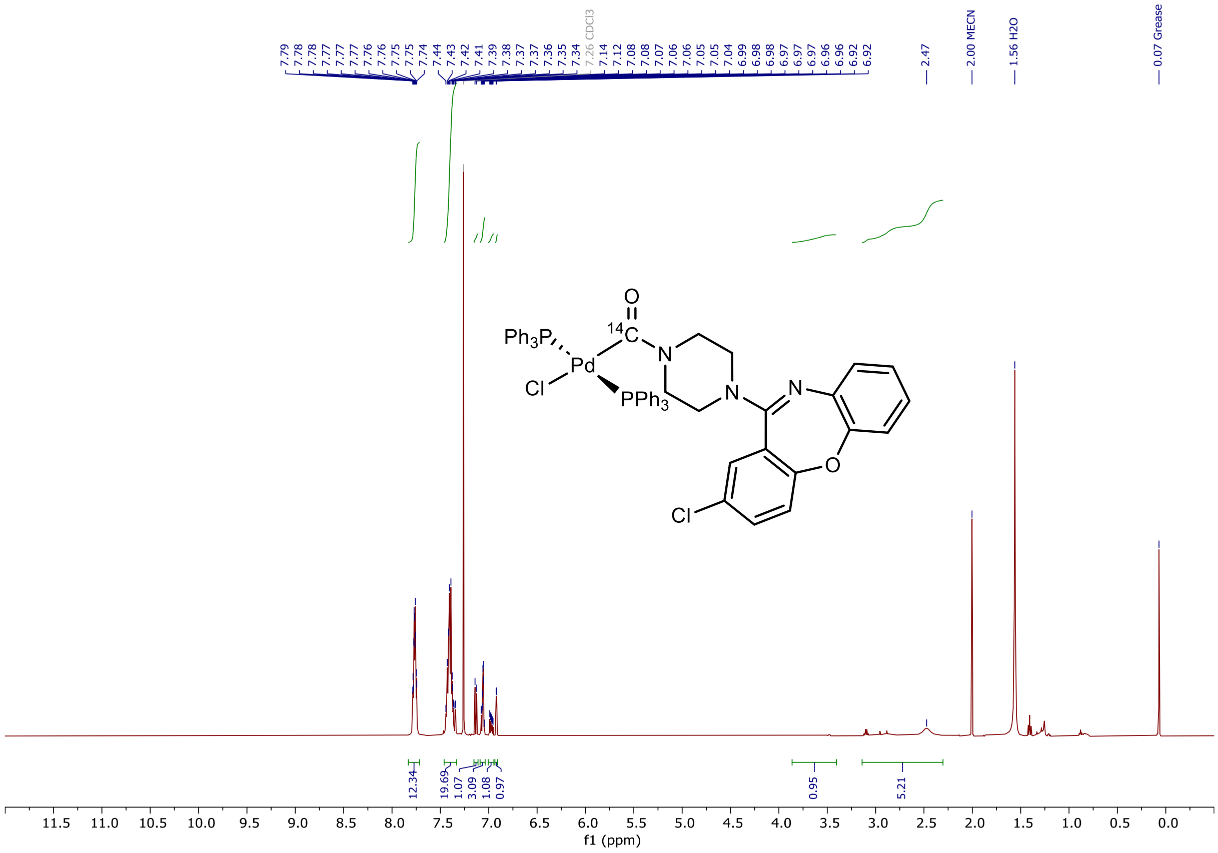

^{13}C-NMR

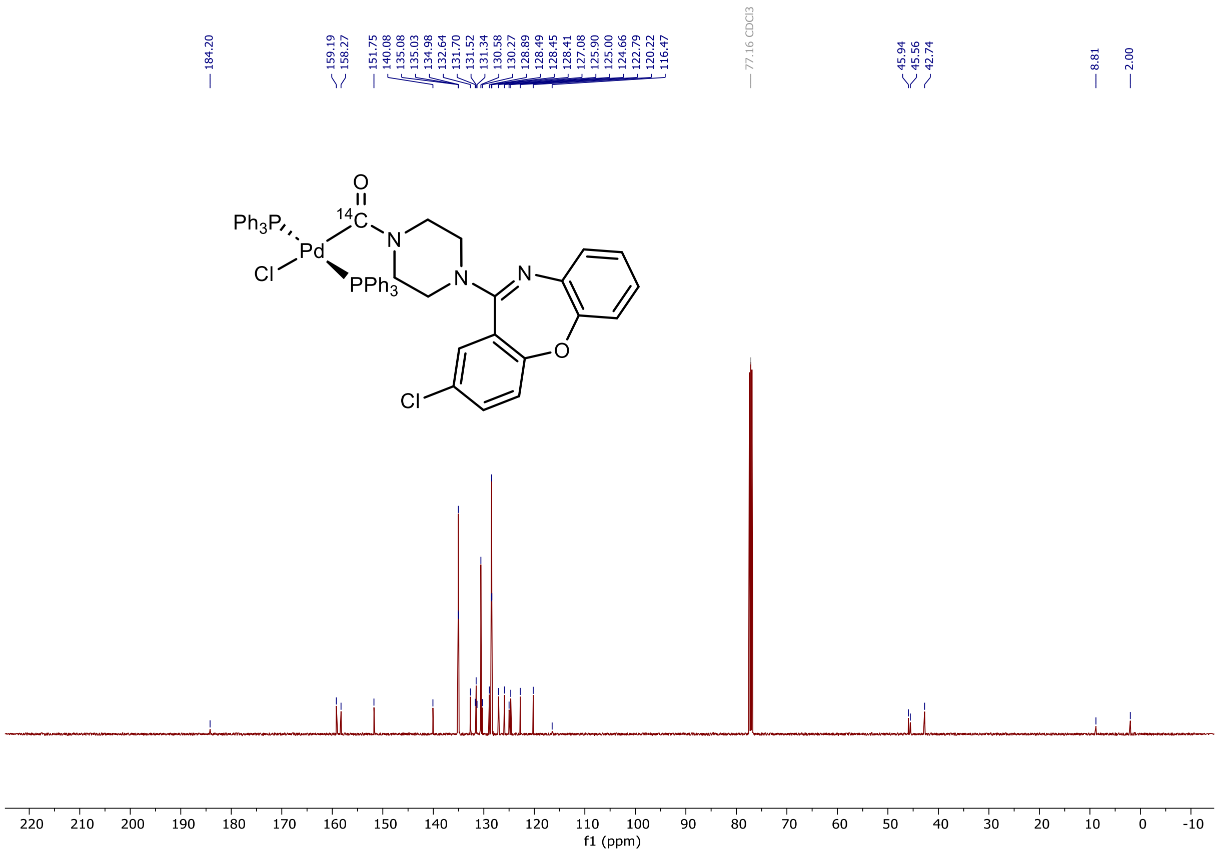

^{31}P-NMR

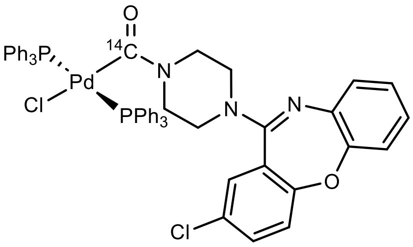

Ph3P
Pd
Cl
PPh3
14C
O
N
N
N
O
Cl

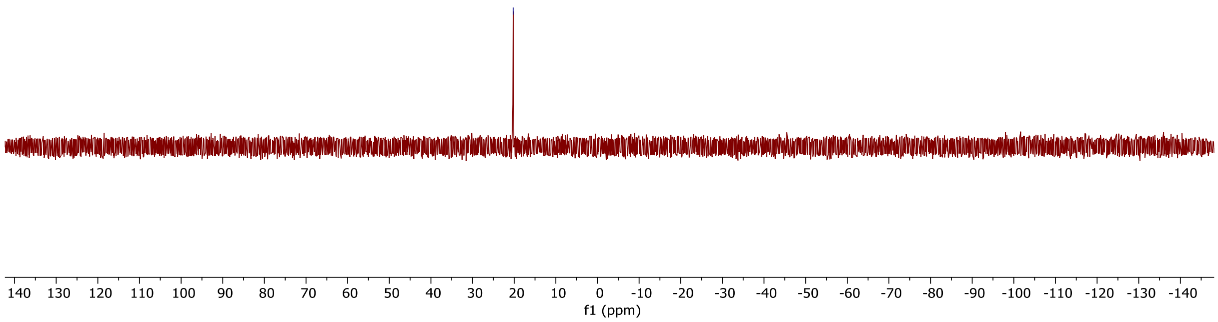

20.25
140 130 120 110 100 90 80 70 60 50 40 30 20 10 0 -10 -20 -30 -40 -50 -60 -70 -80 -90 -100 -110 -120 -130 -140
f1 (ppm)

***trans*-Chloro(1-cyclopropyl-6-fluoro-4-oxo-7-(piperidin-4-yl)-1,4-dihydroquinoline-3-carboxylic acid)carbonyl)bis(triphenylphosphine) palladium(II) (Pd-11)**

^{1}H-NMR

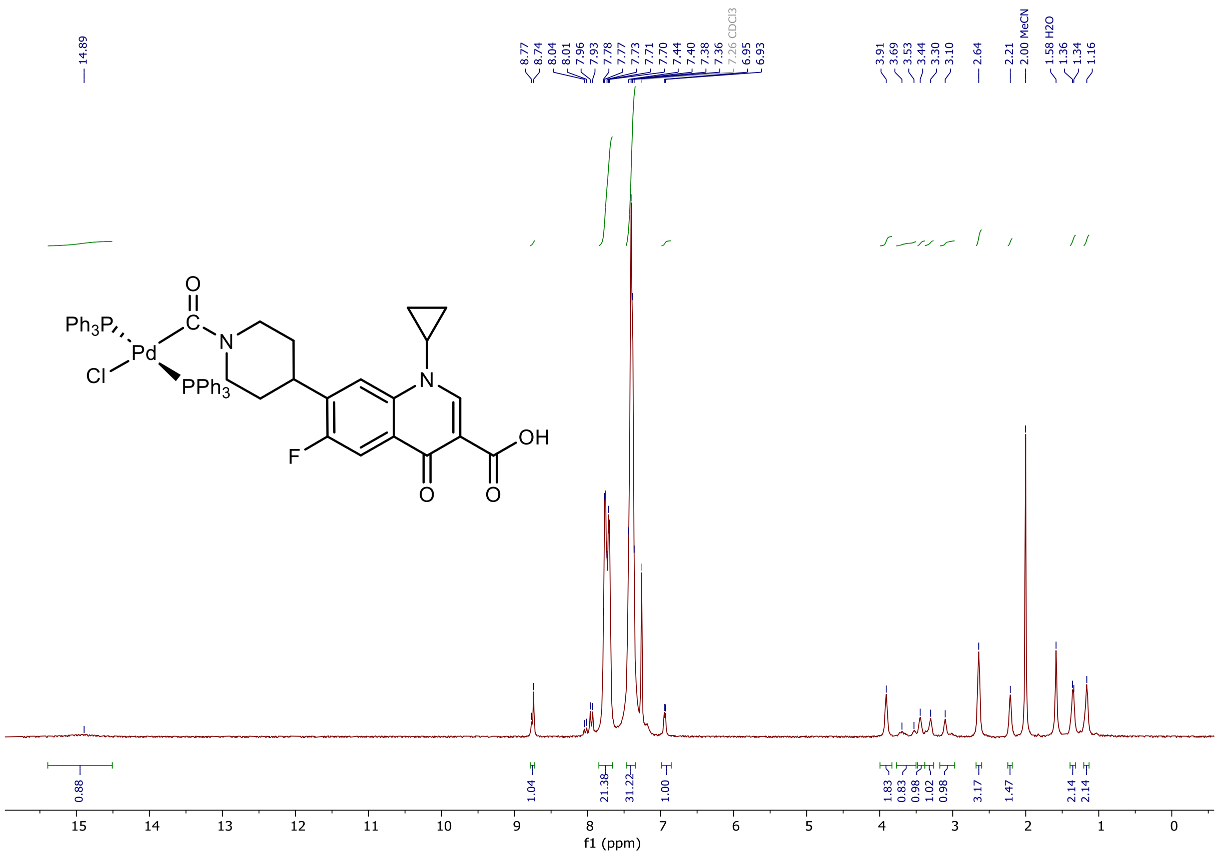

^{13}C-NMR

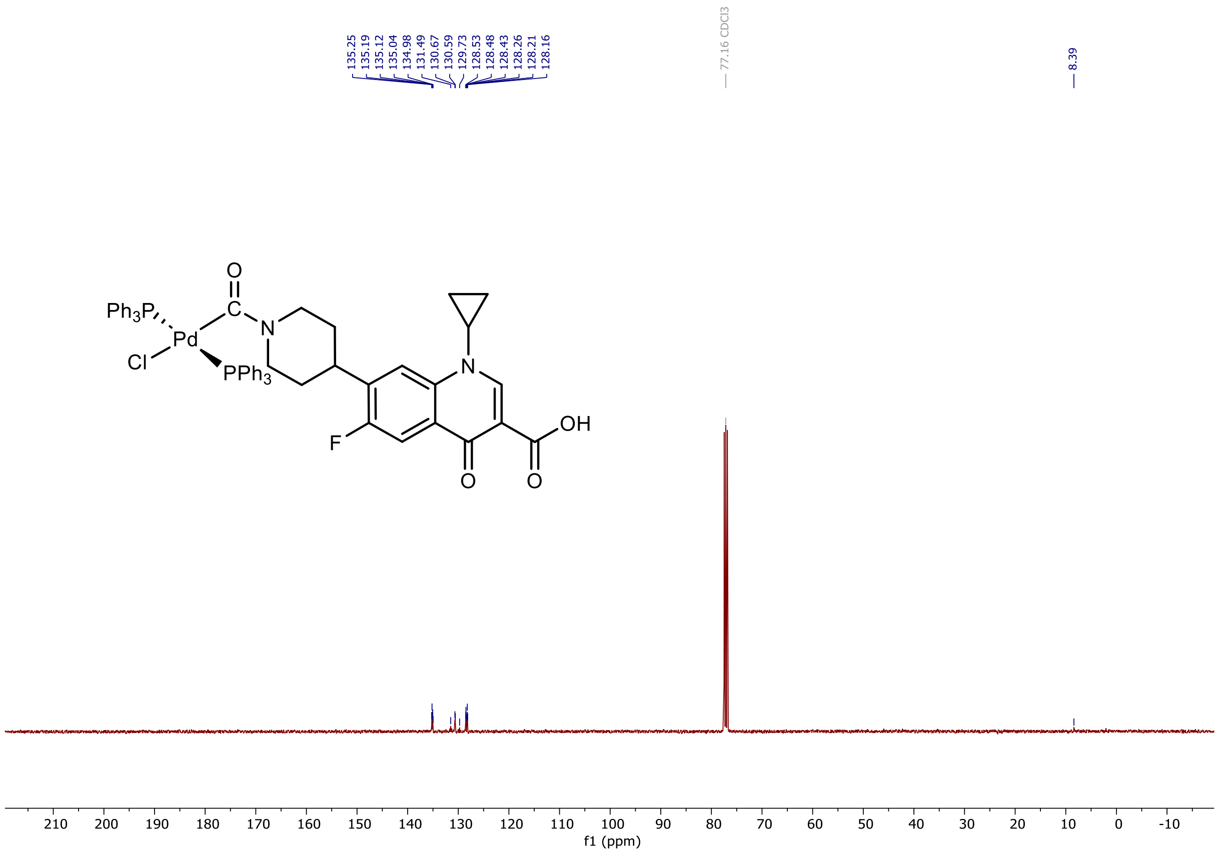

^{31}P-NMR

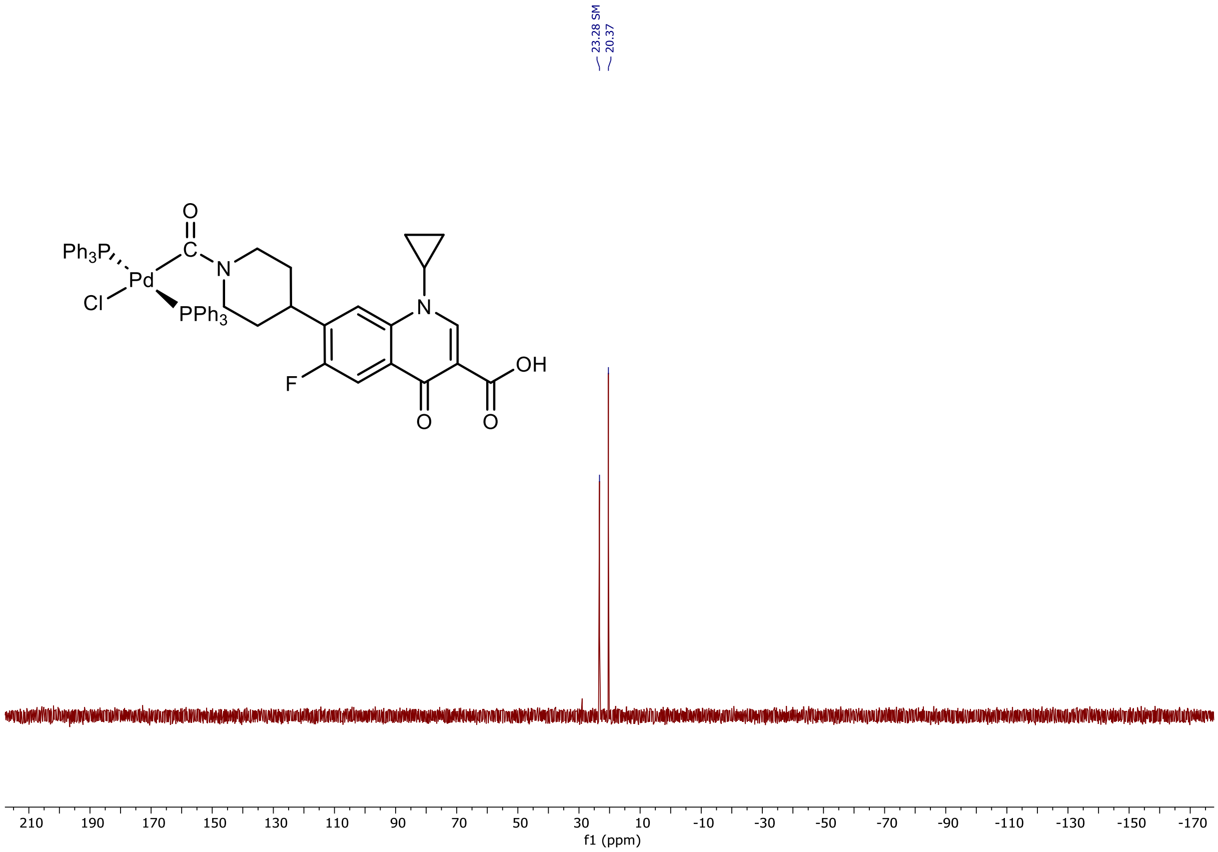

^{19}F-NMR

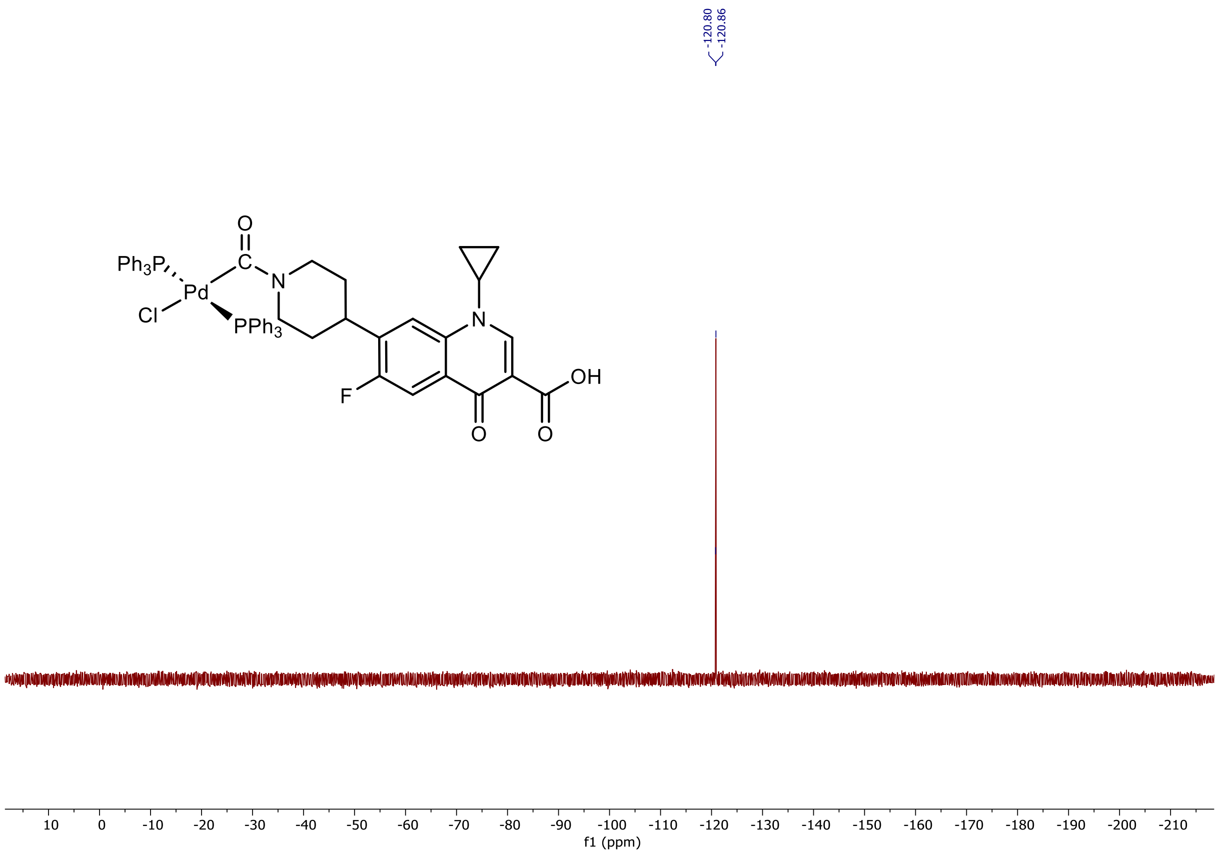

***trans*-Chloro(1-cyclopropyl-6-fluoro-4-oxo-7-(piperidin-4-yl)-1,4-dihydroquinoline-3-carboxylic acid)-^{13}C-carbonyl)bis(triphenylphosphine) palladium(II) (^{13}C-Pd-11)**

^{1}H-NMR

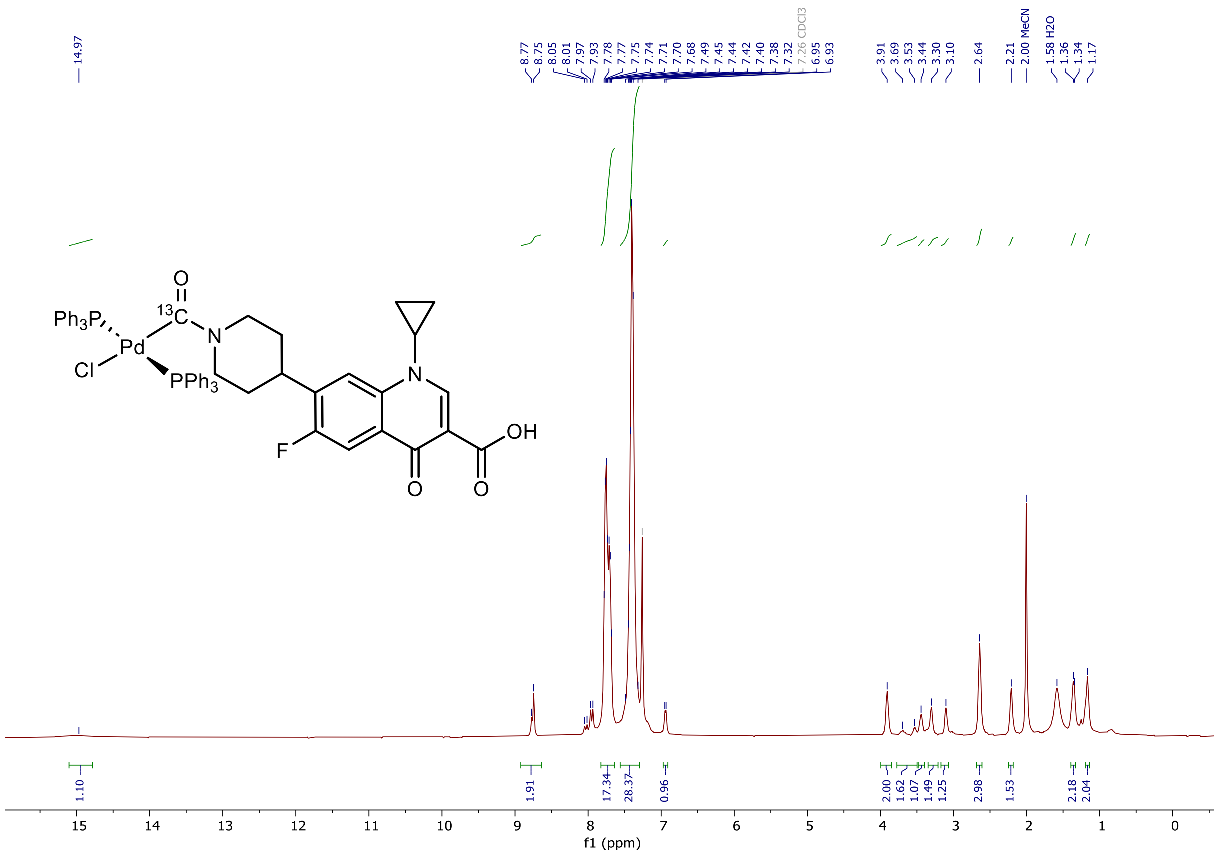

^{13}C-NMR

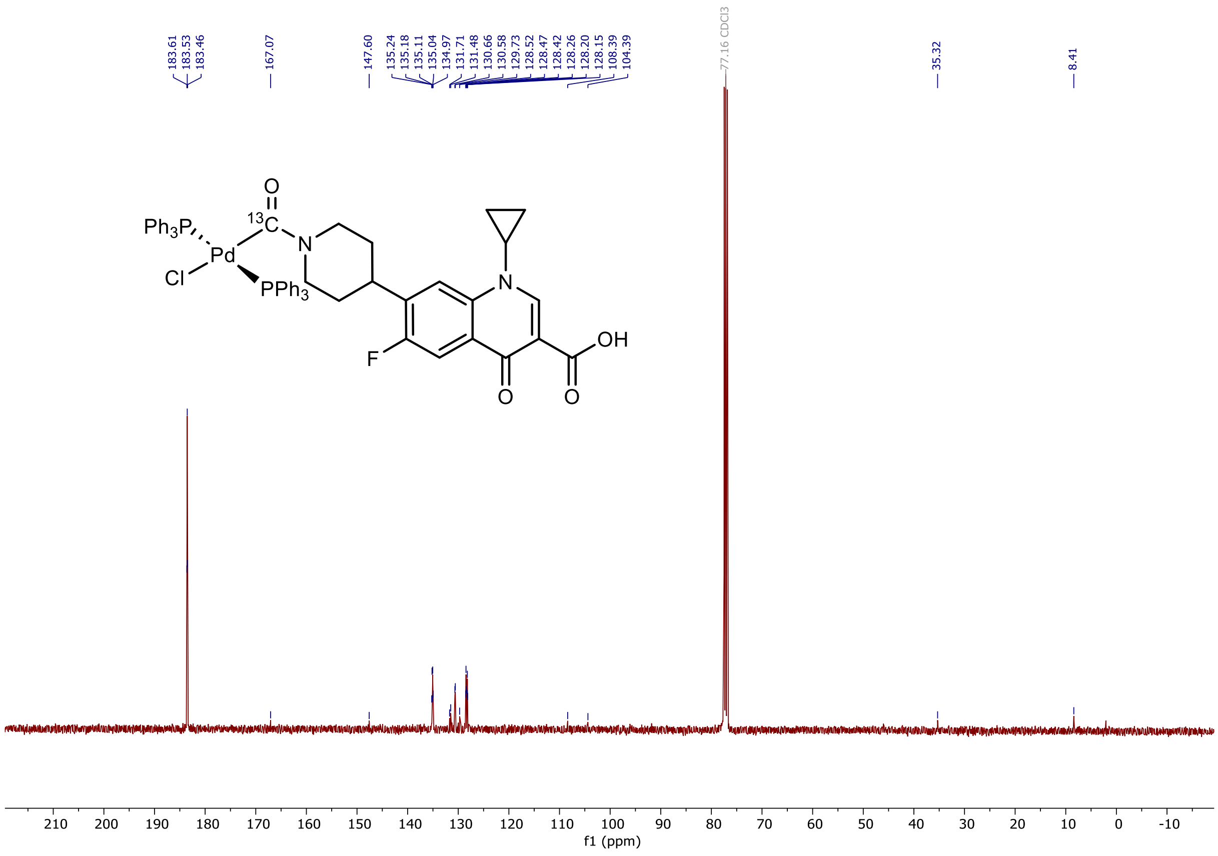

^{31}P-NMR

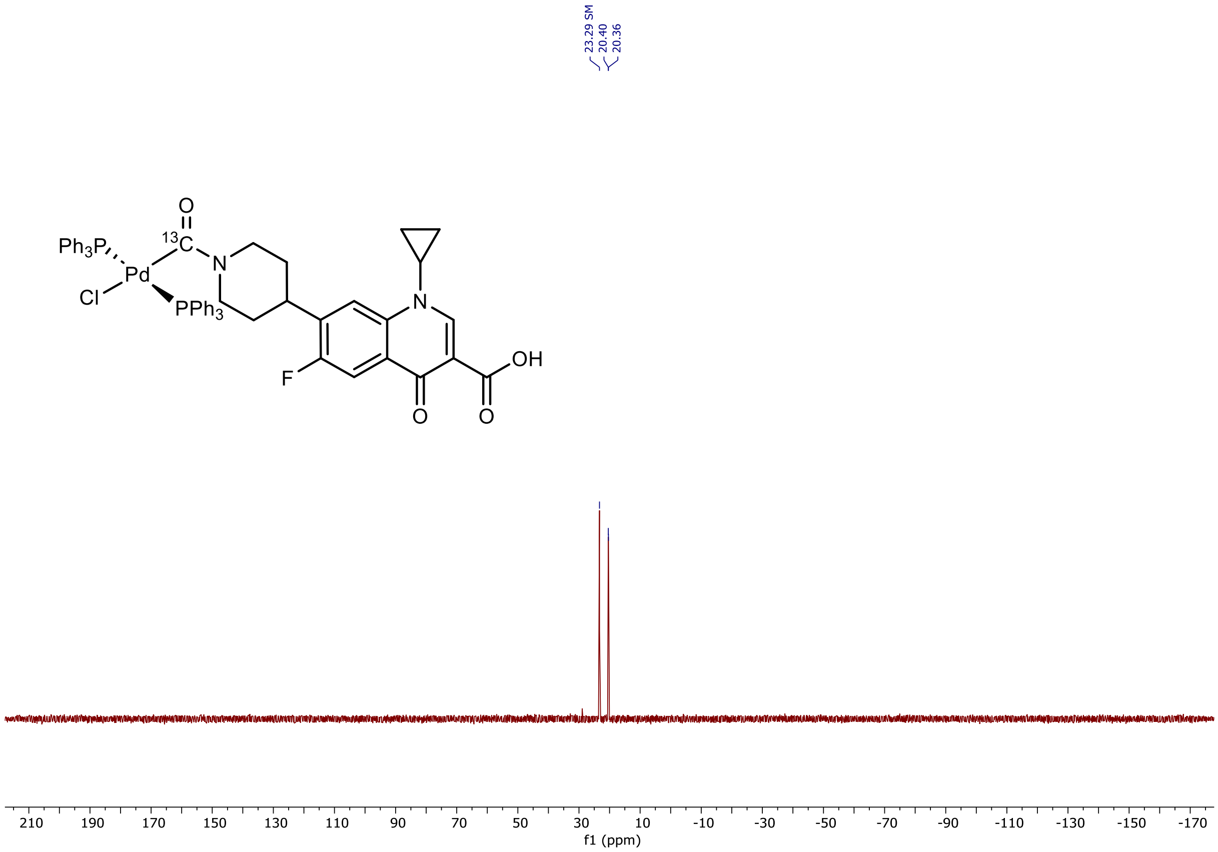

^{19}F-NMR

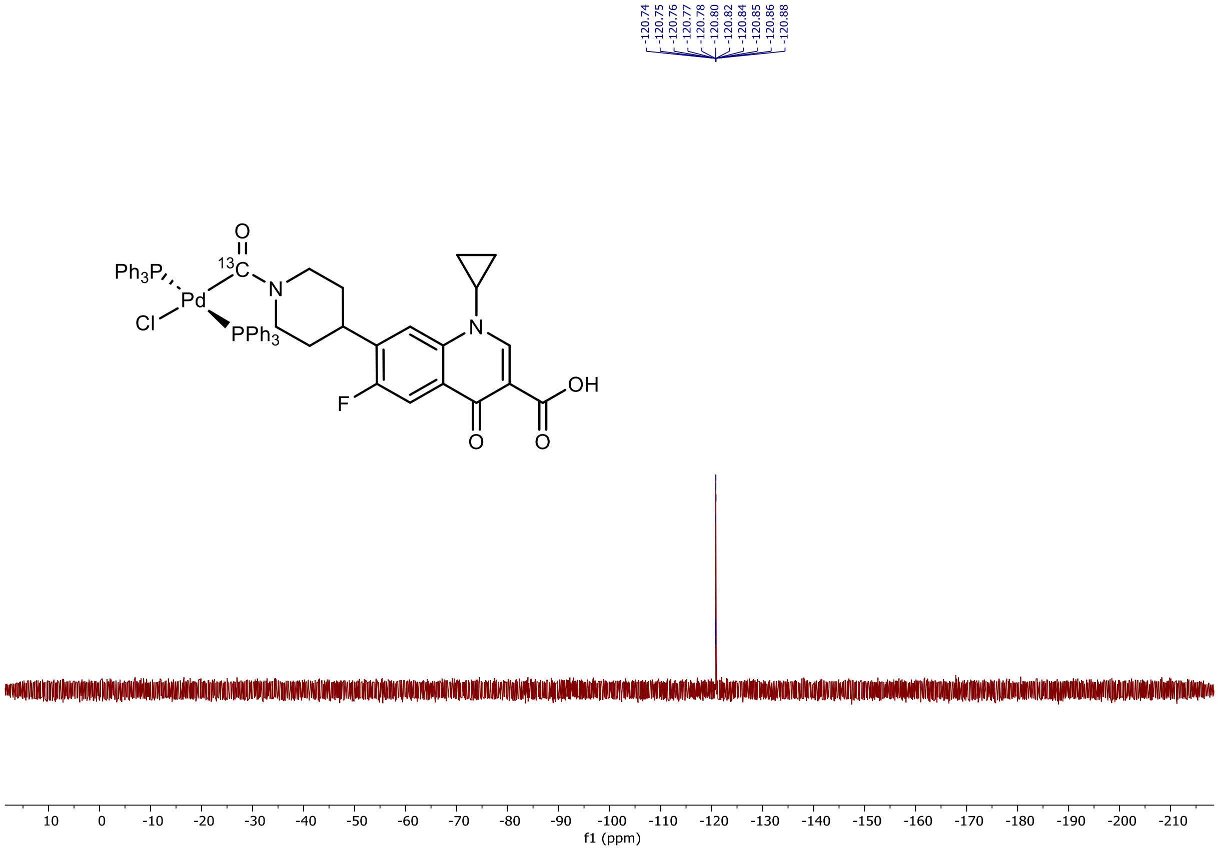

***trans*-Chloro((3S,4R)-3-((benzo[*d*][1,3]dioxol-5-yloxy)methyl)-4-(4-fluorophenyl)piperidine)carbonyl)bis(triphenylphosphine) palladium(II) (Pd-12)**

^{1}H-NMR

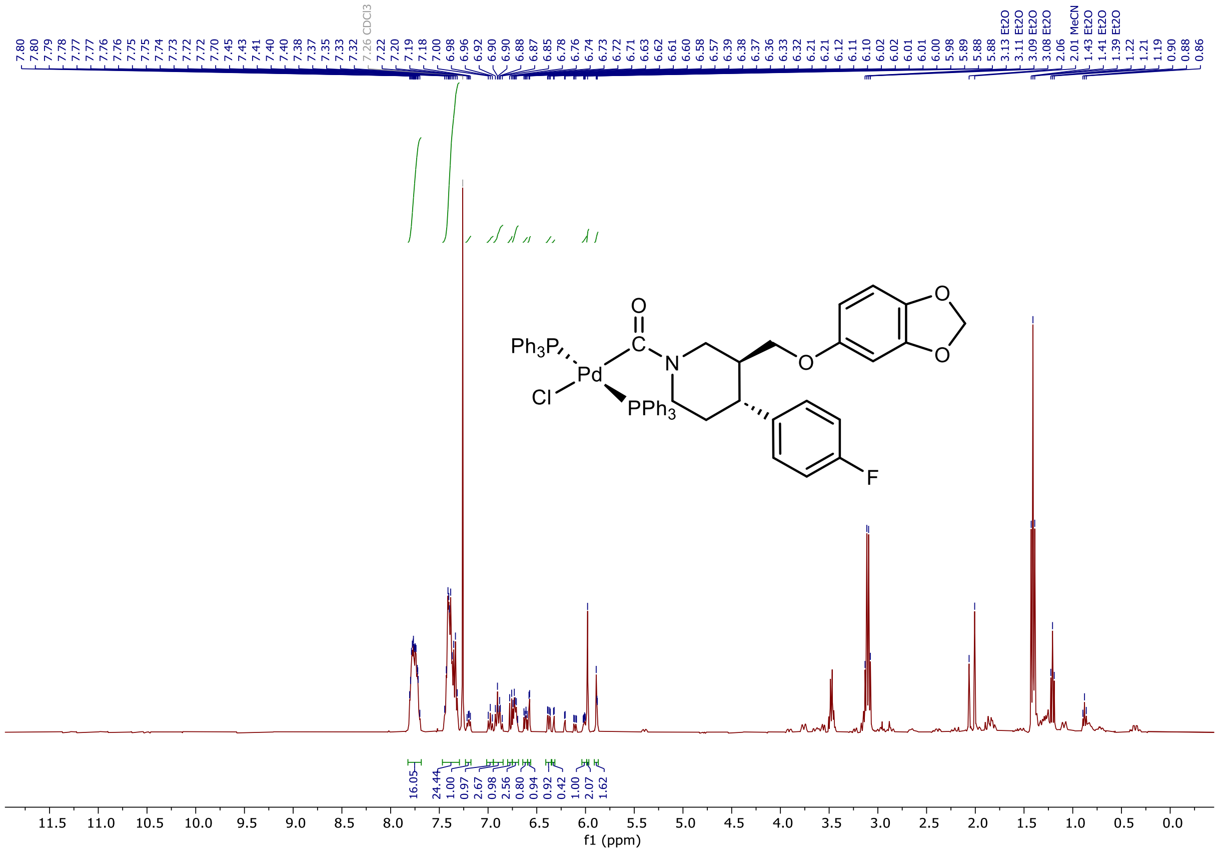

^{13}C-NMR

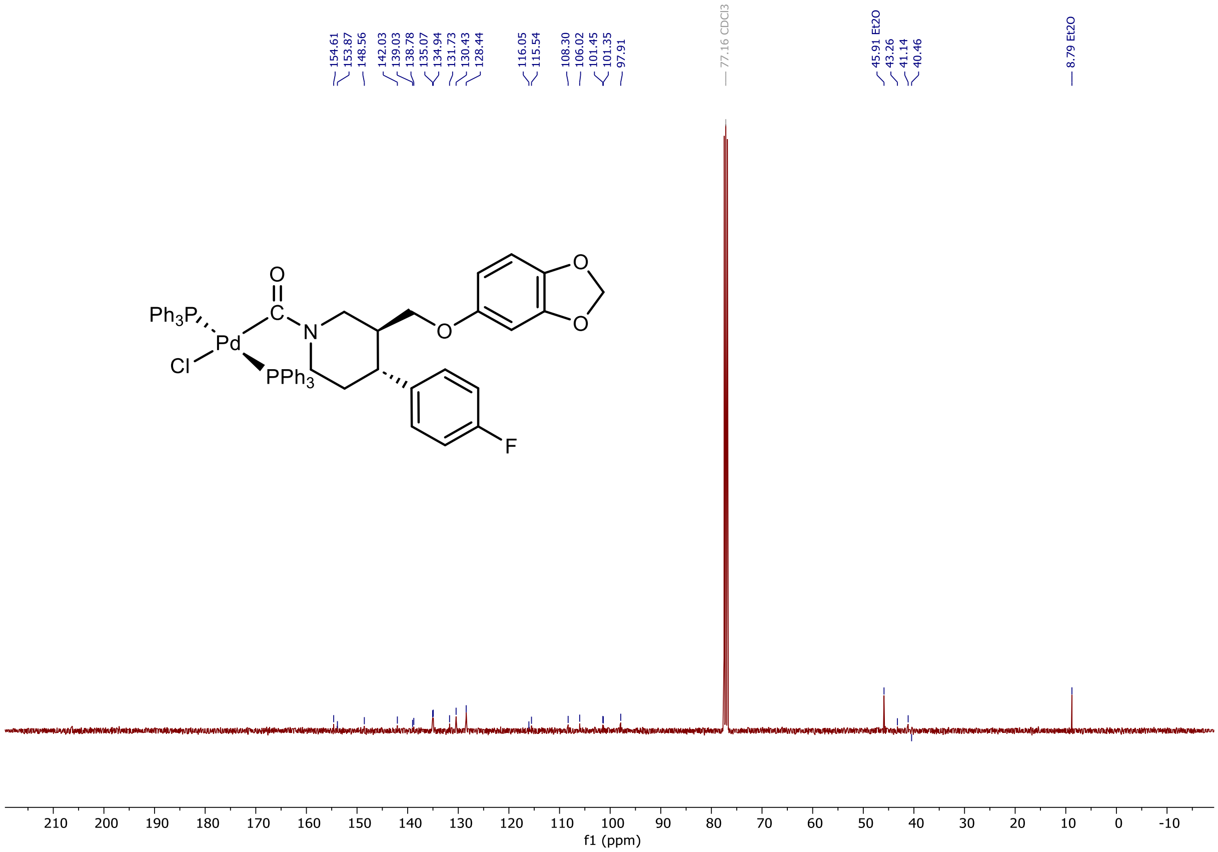

^{31}P-NMR

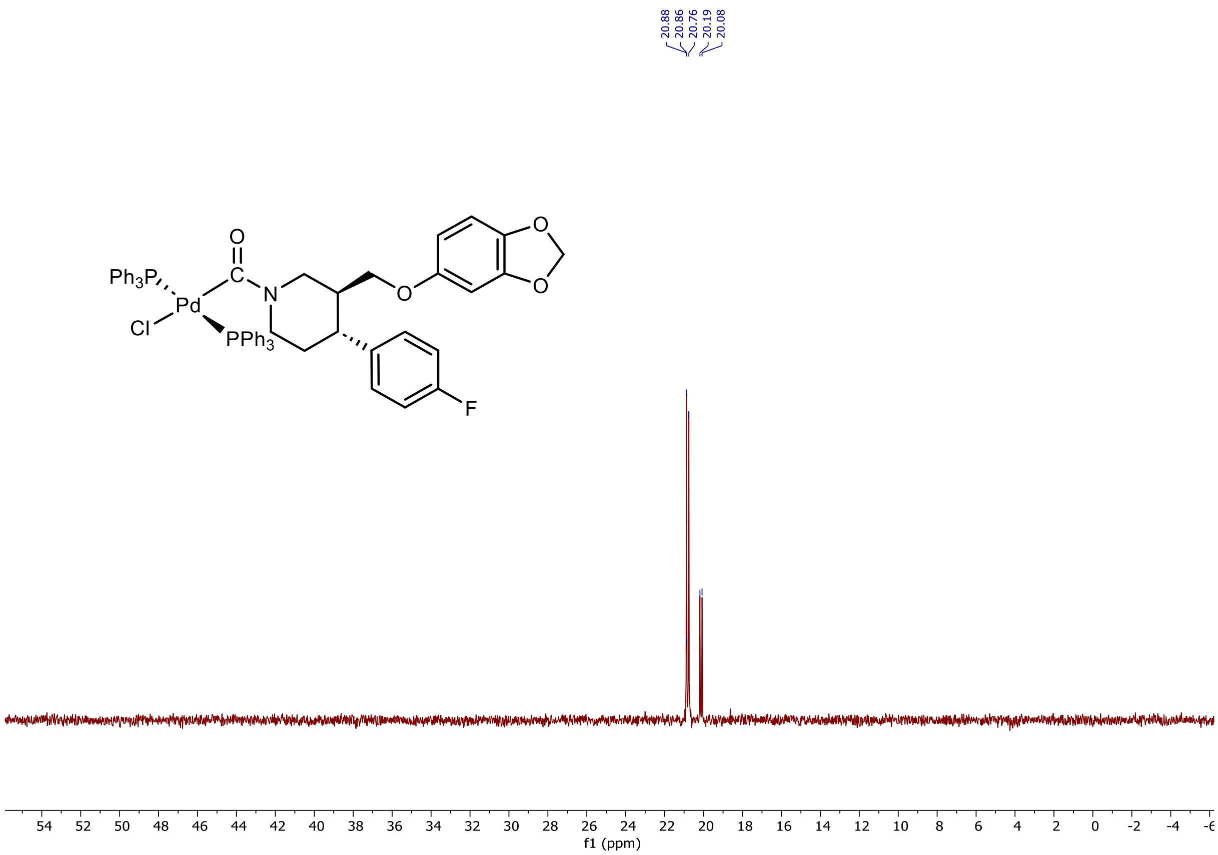

^{19}F-NMR

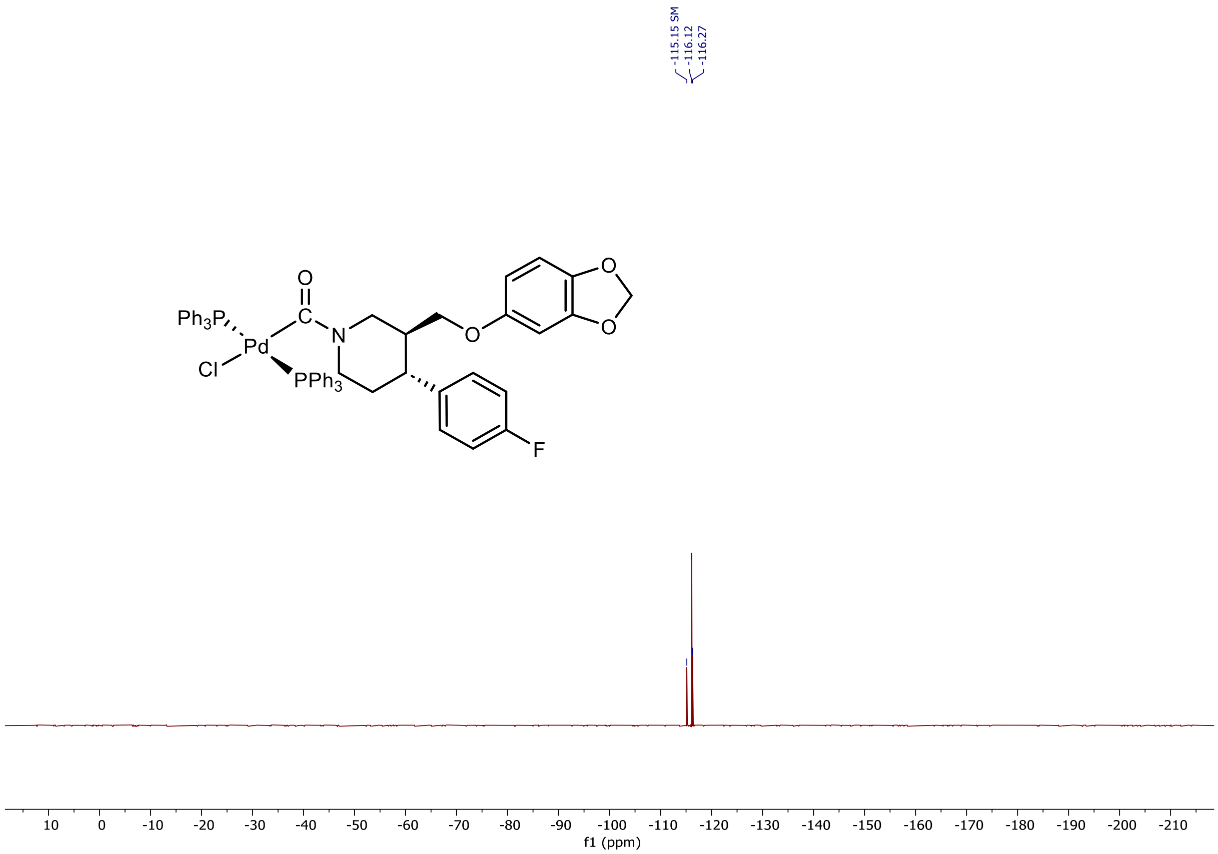

trans-Chloro((3S,4R)-3-((benzo[*d*][1,3]dioxol-5-yloxy)methyl)-4-(4-fluorophenyl)piperidine)-^{13}C-carbonyl)bis(triphenylphosphine) palladium(II) (^{13}C-Pd-12)

^{1}H-NMR

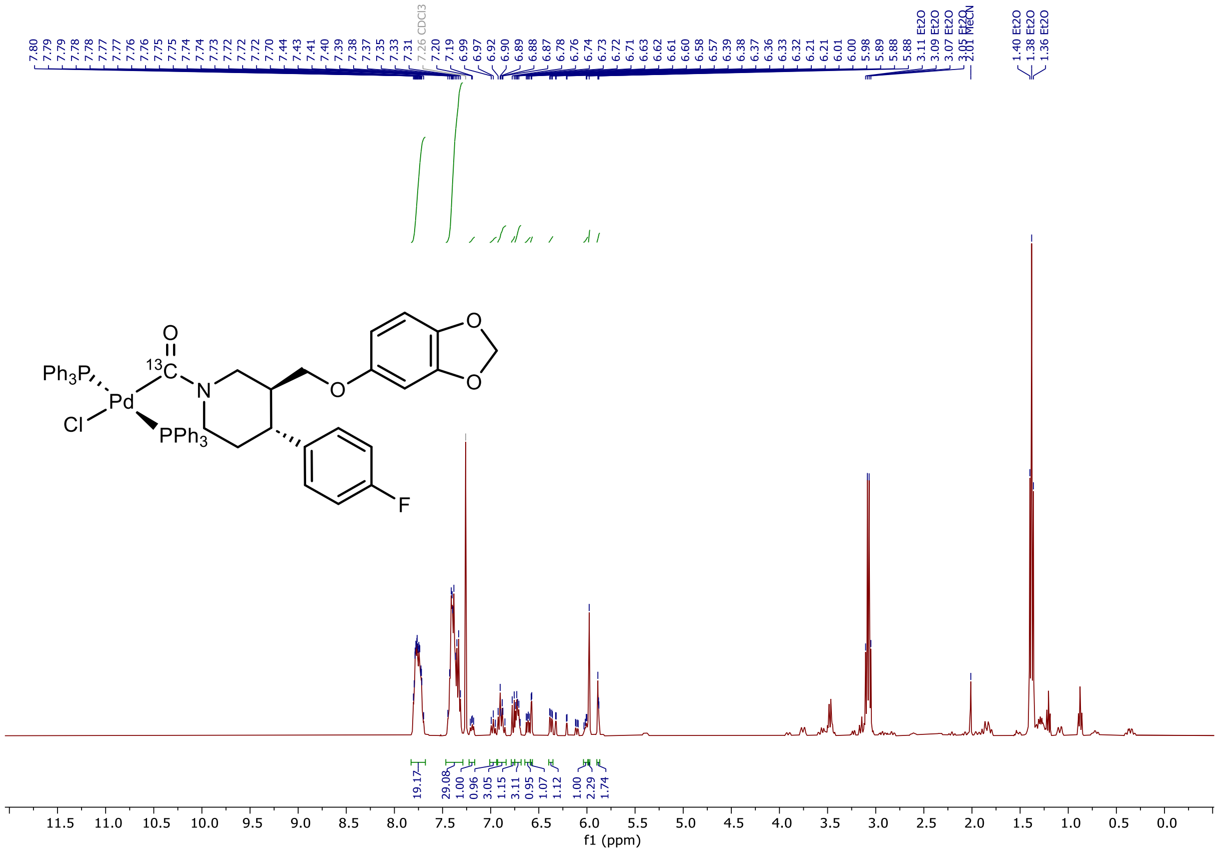

^{13}C-NMR

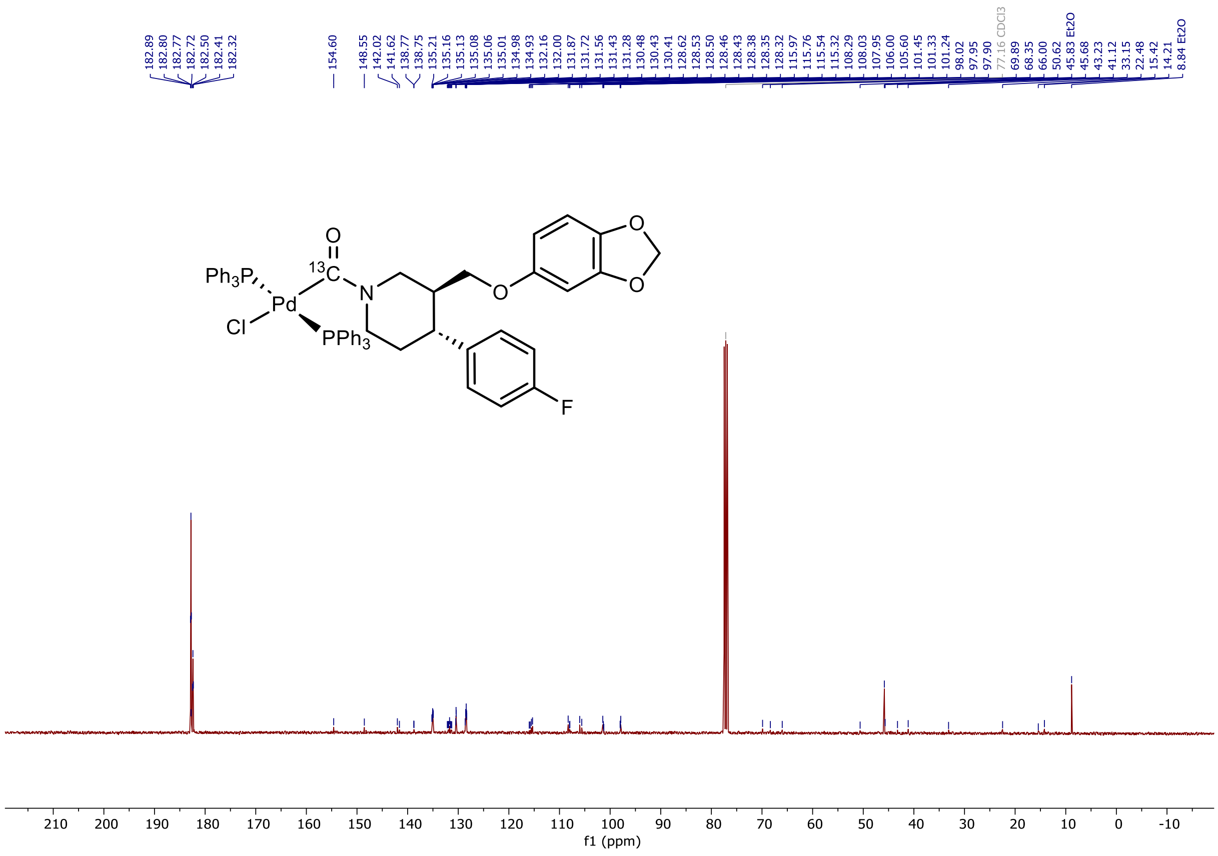

^{31}P-NMR

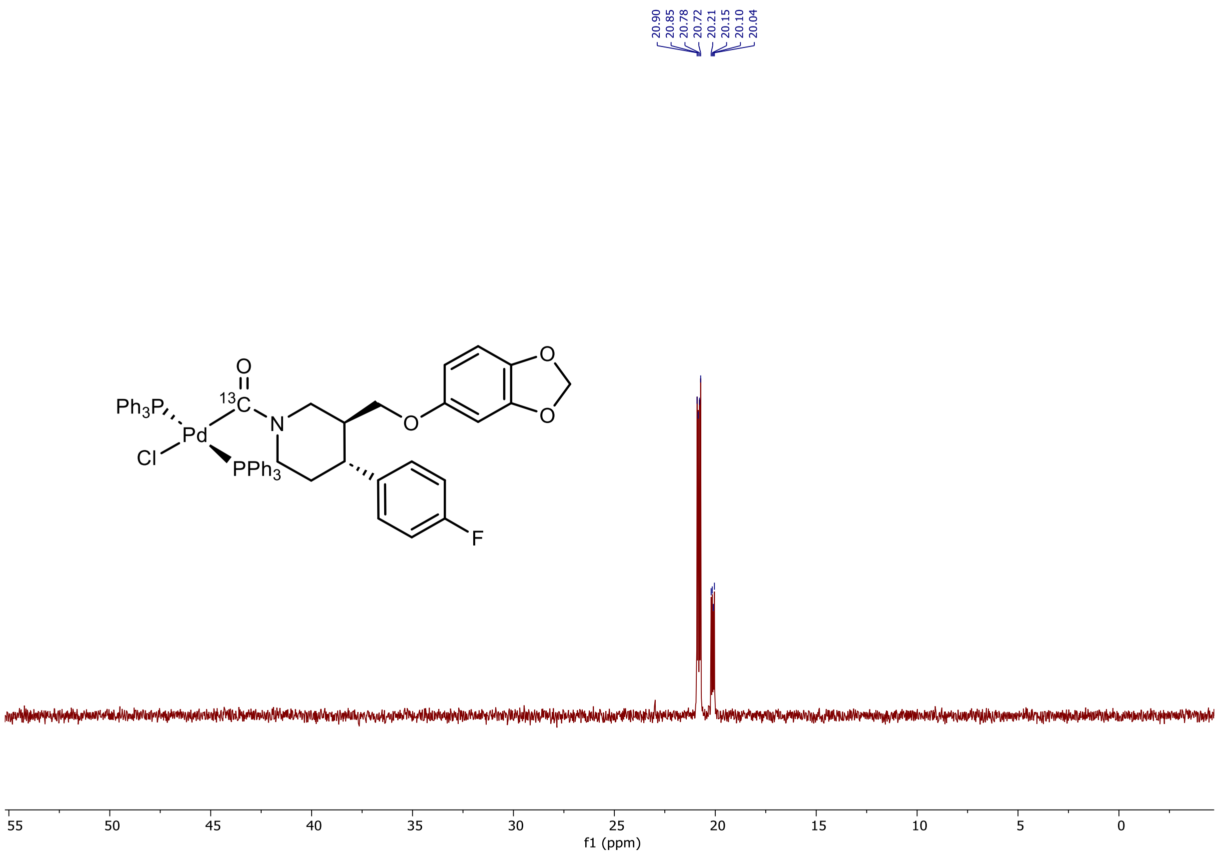

^{19}F-NMR

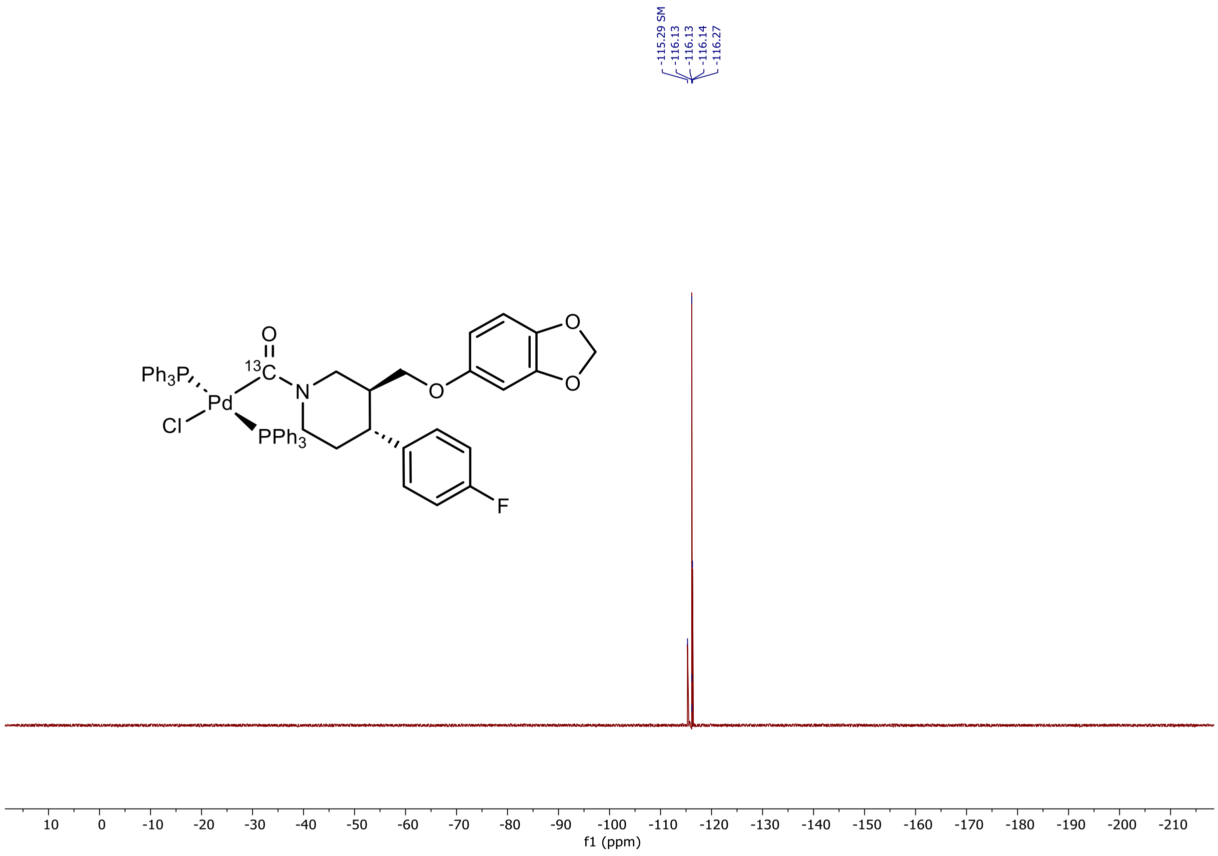

***trans*-Chloro(4-(4-(4-fluorophenyl)-1-(piperidin-4-yl)-1H-imidazol-5-yl)-2-methoxy-pyrimidine)carbonyl)bis(triphenylphosphine) palladium(II) (Pd-13)**

^{1}H-NMR

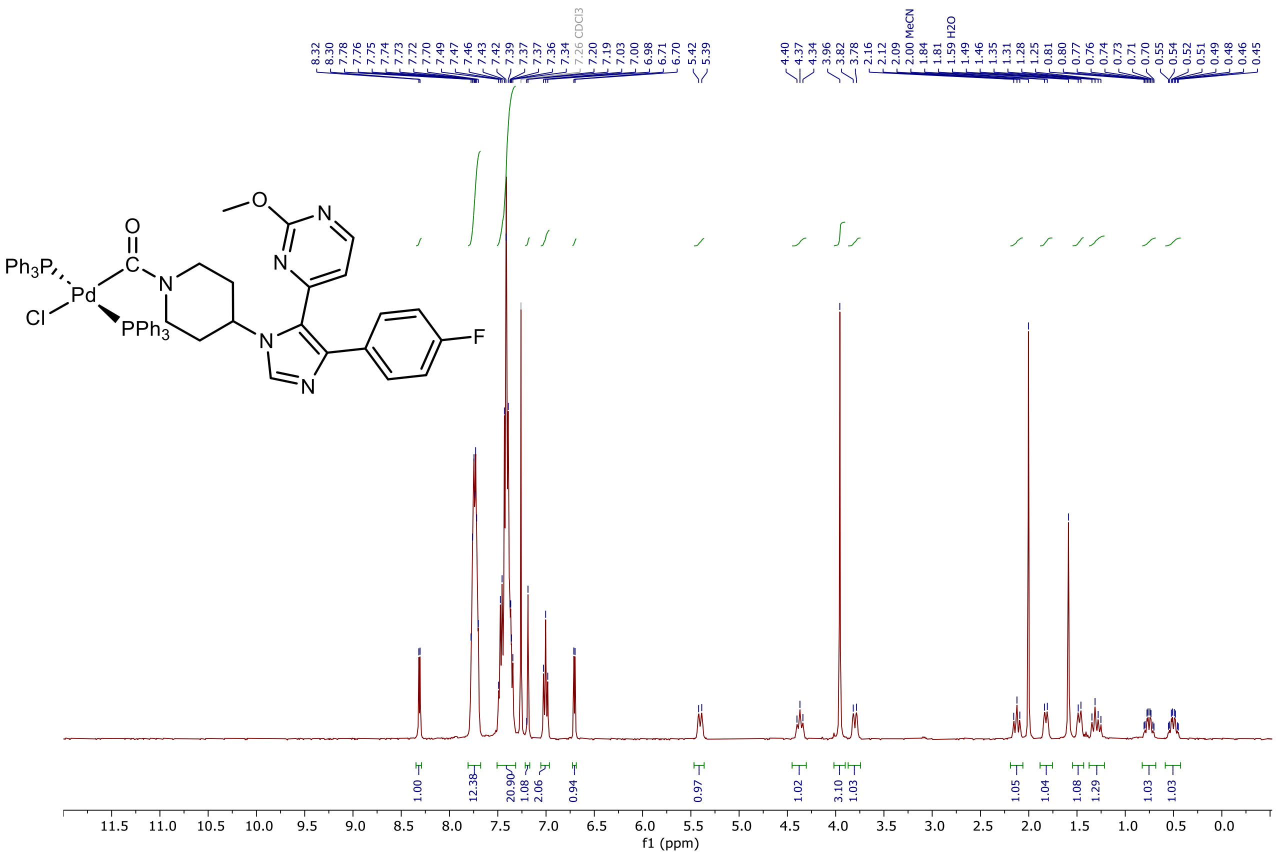

^{13}C-NMR

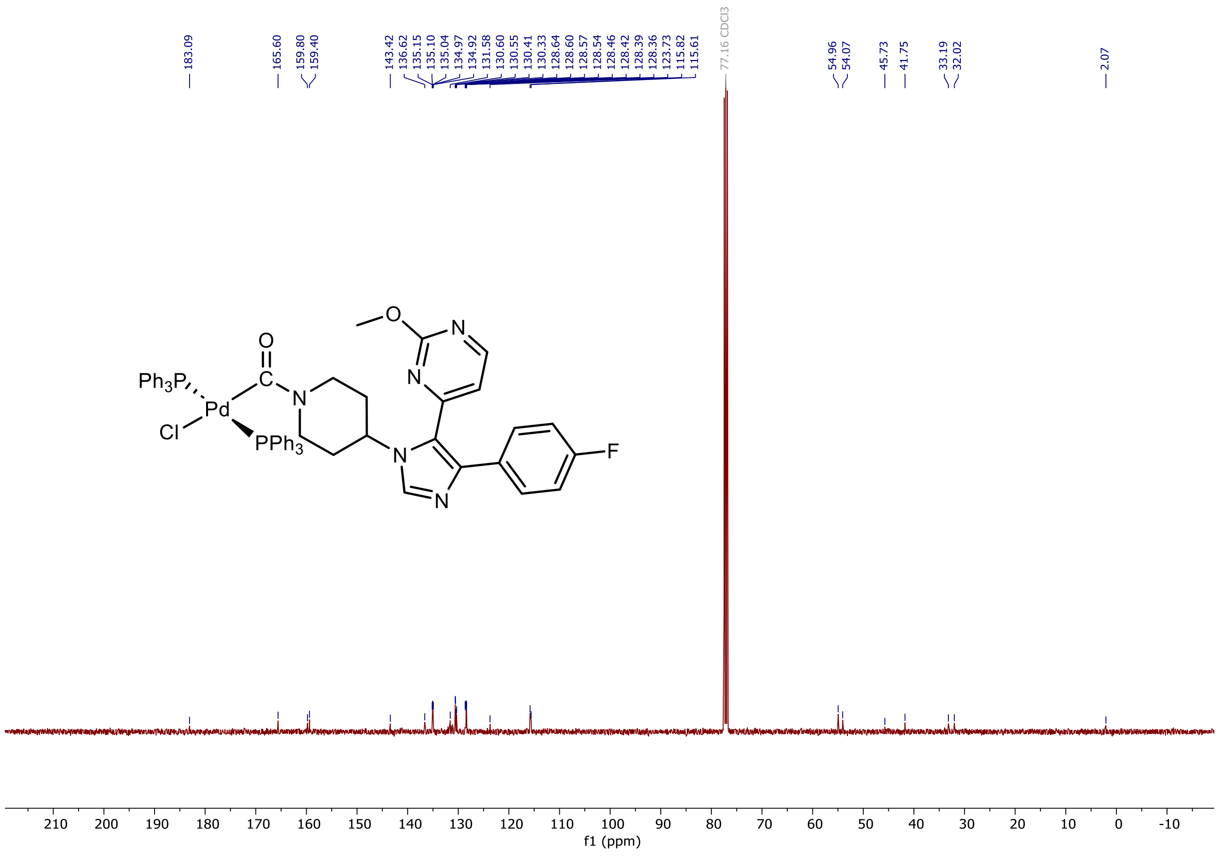

^{31}P-NMR

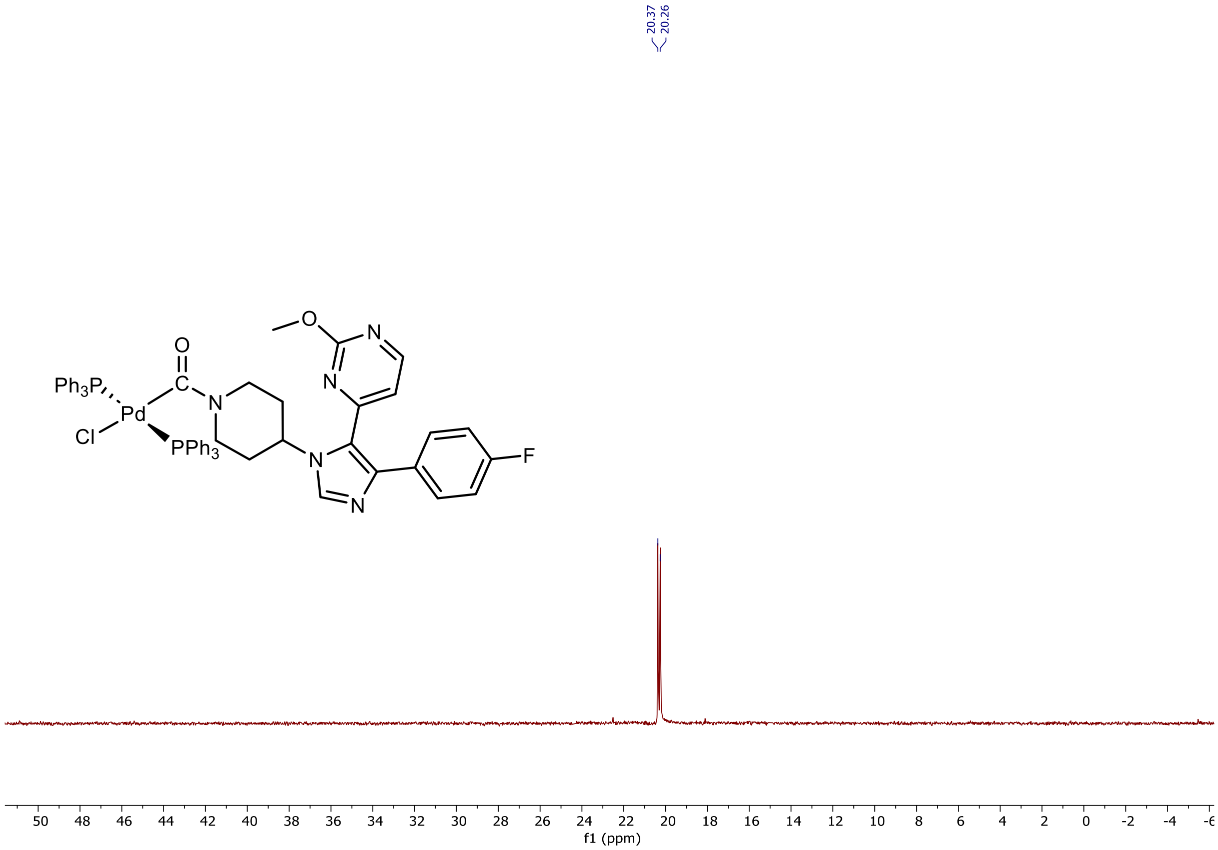

^{19}F-NMR

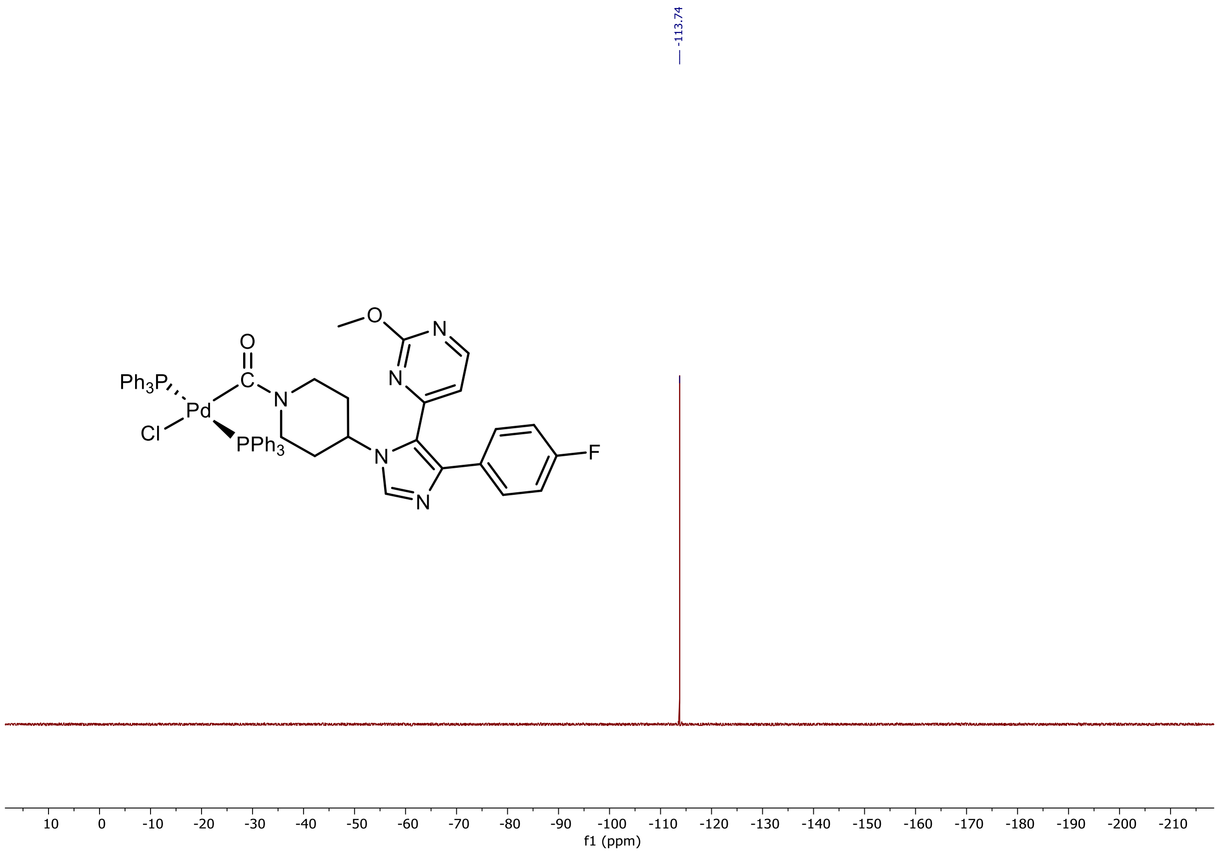

***trans*-Chloro(4-(4-(4-fluorophenyl)-1-(piperidin-4-yl)-1H-imidazol-5-yl)-2-methoxy-pyrimidine)-^{13}C-carbonyl)bis(triphenylphosphine) palladium(II) (^{13}C-Pd-13)**

^{1}H-NMR

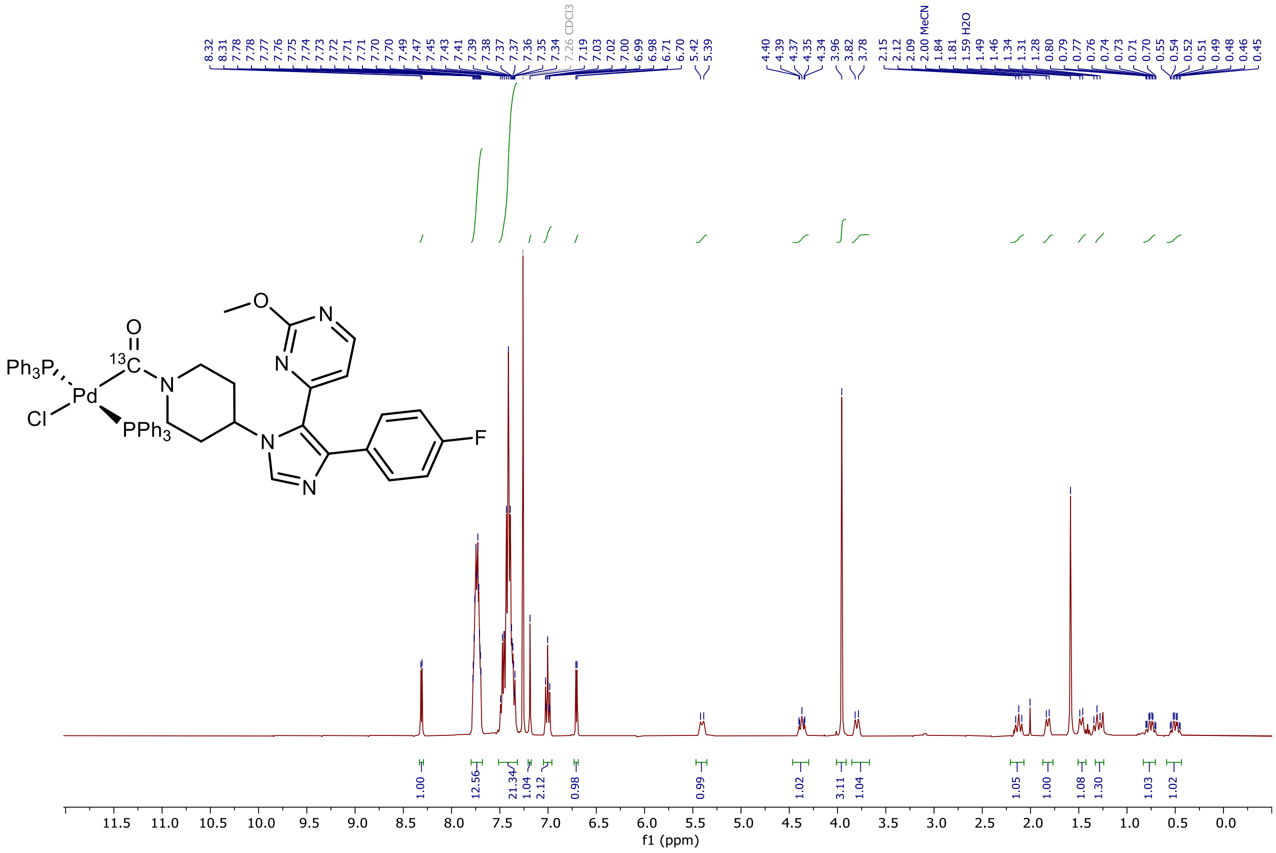

^{13}C-NMR

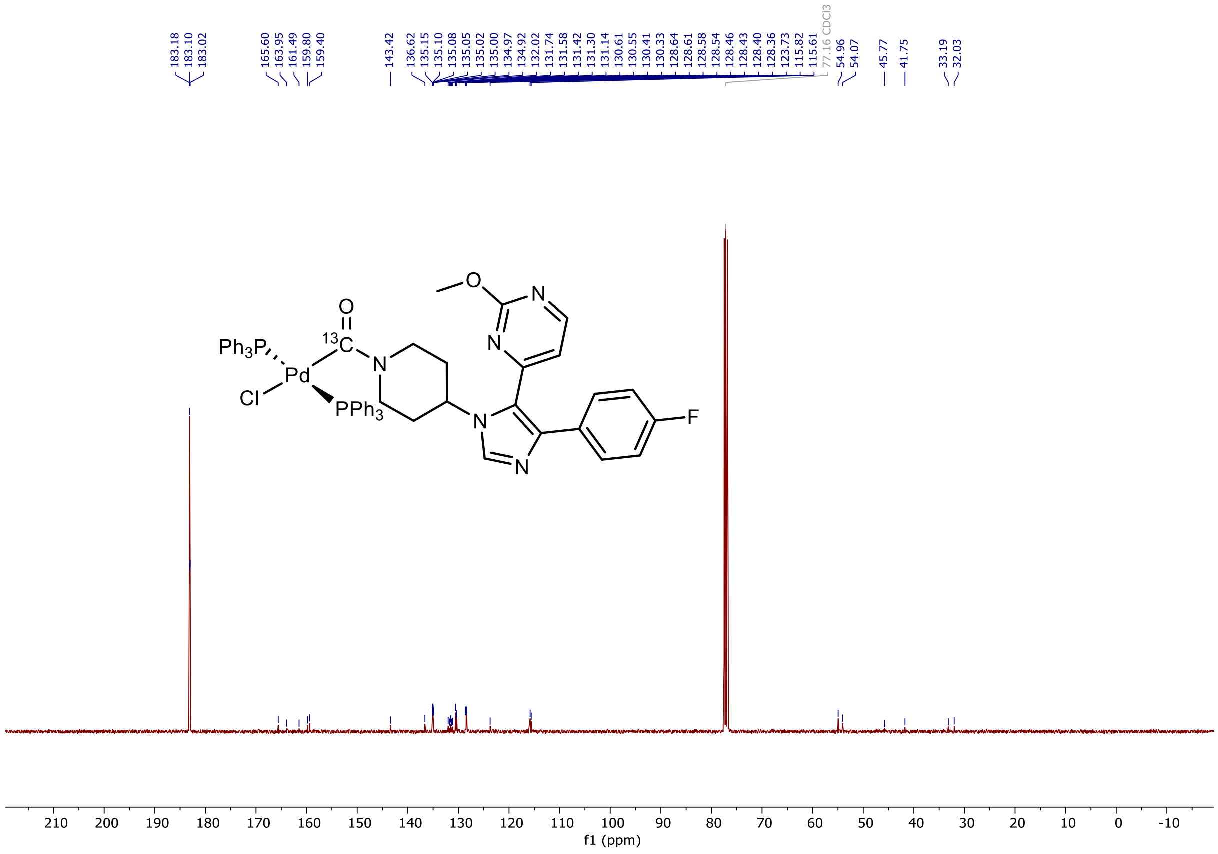

^{31}P-NMR

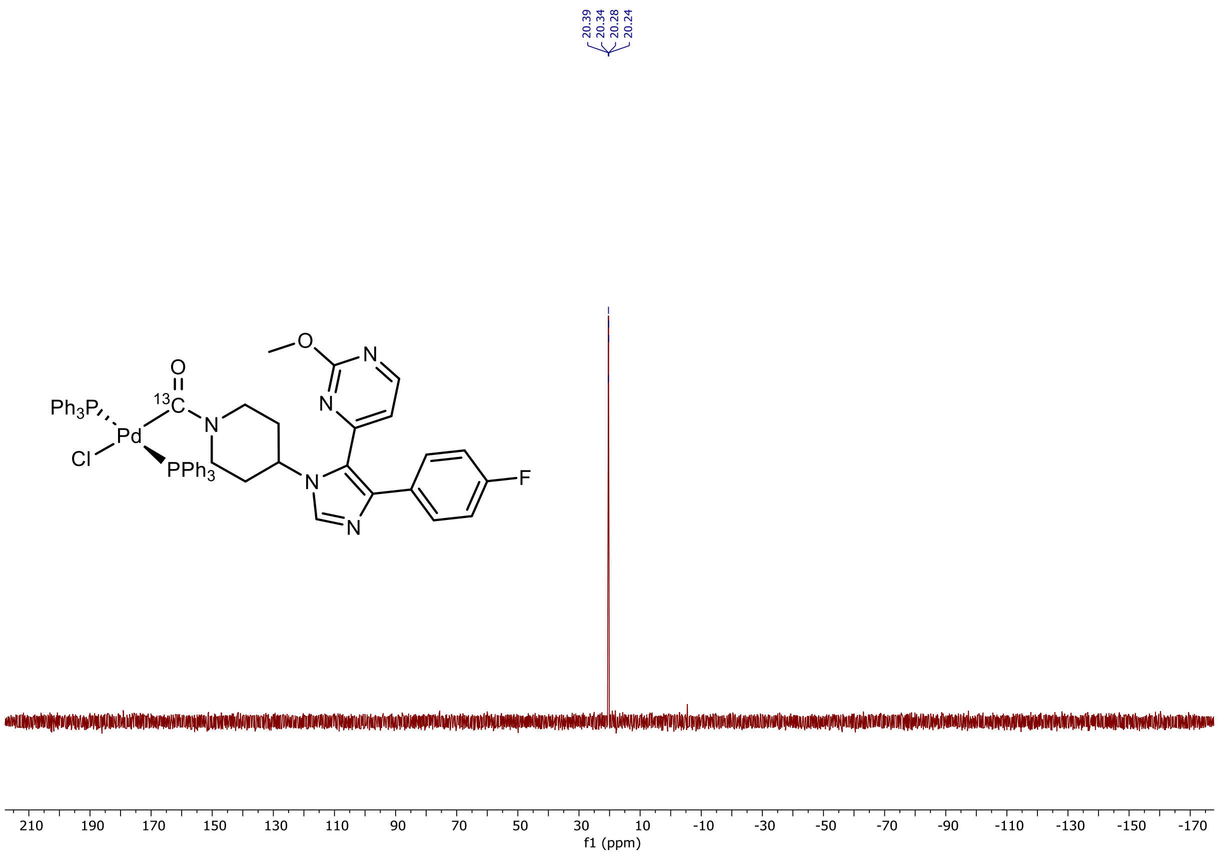

^{19}F-NMR

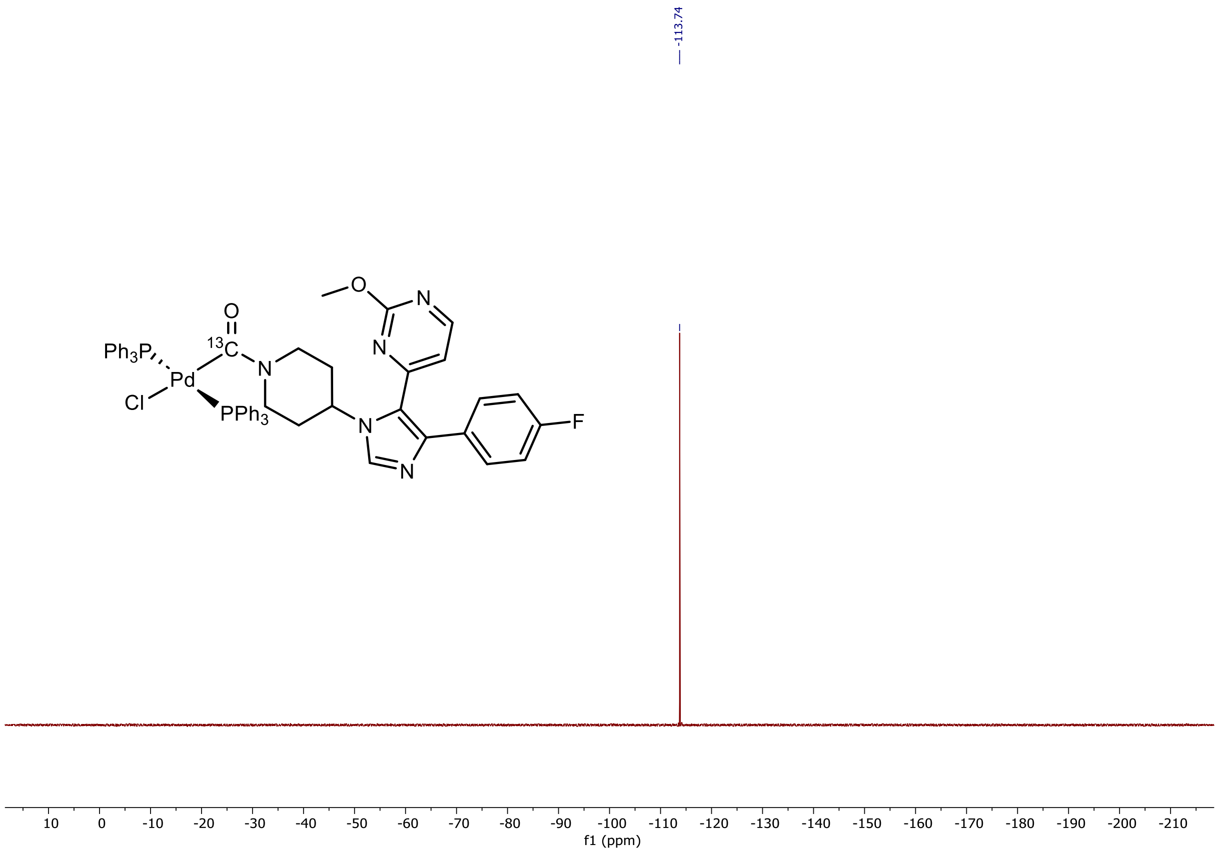

***trans*-Chloro(4-(4-(4-fluorophenyl)-1-(piperidin-4-yl)-1H-imidazol-5-yl)-2-methoxy-pyrimidine)-^{14}C-carbonyl)bis(triphenylphosphine) palladium(II) (^{14}C-Pd-13)**

^{1}H-NMR

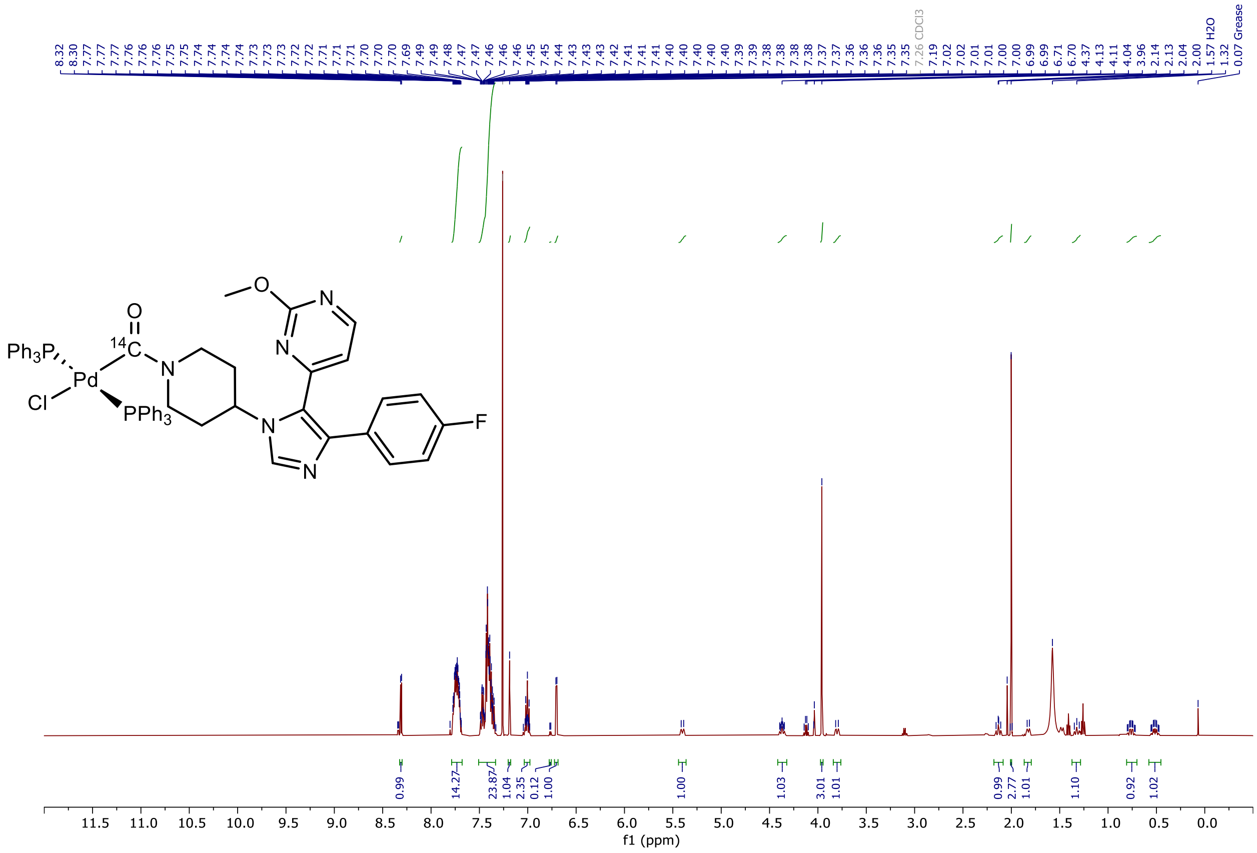

^{13}C-NMR

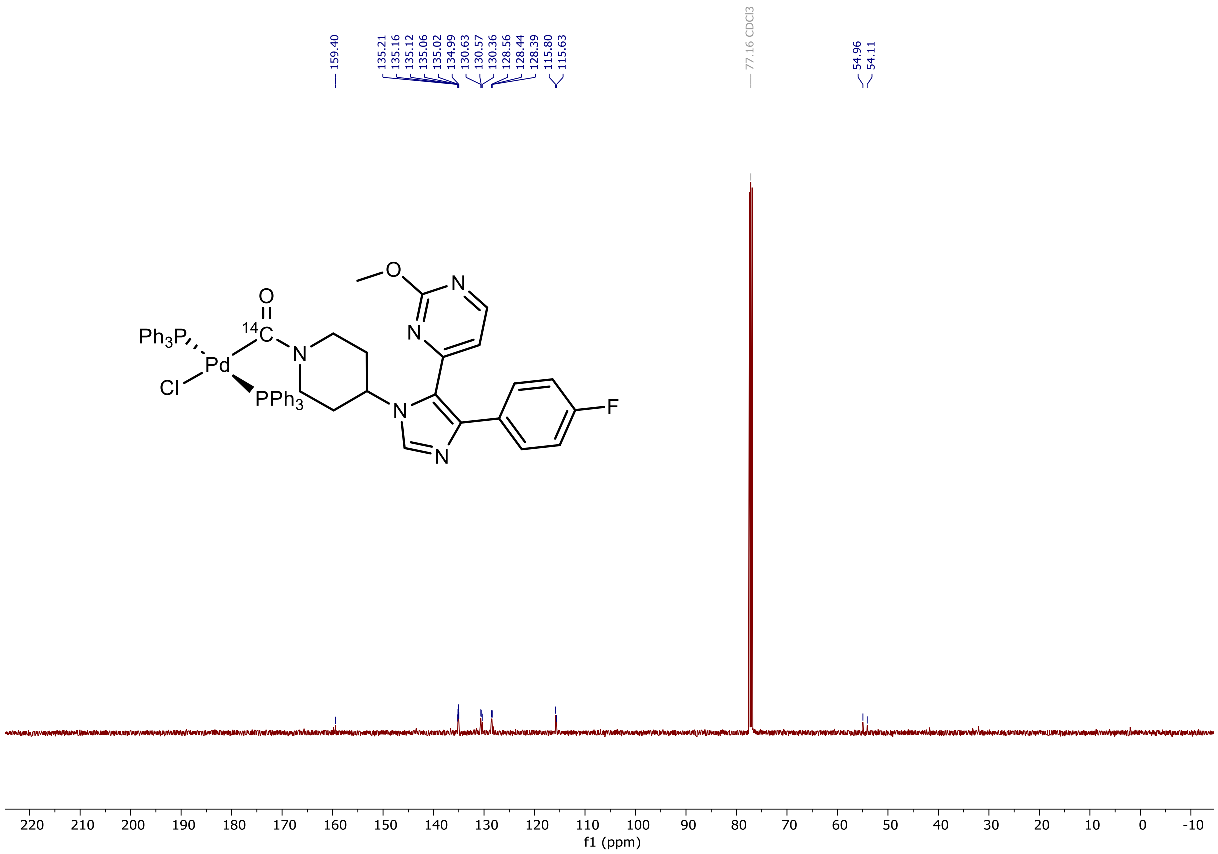

^{31}P-NMR

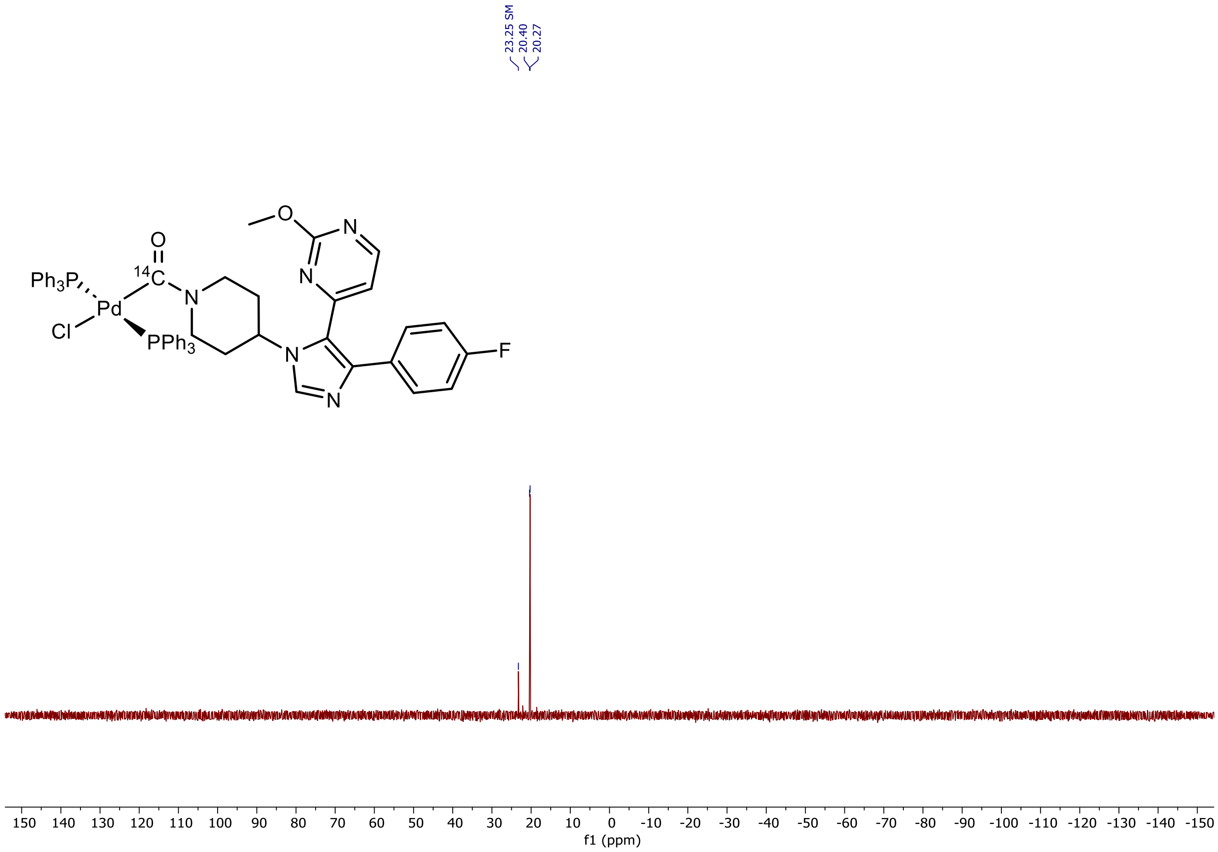

N-Propyl-[1,1'-biphenyl]-4-carboxamide (1a)

^{1}H-NMR

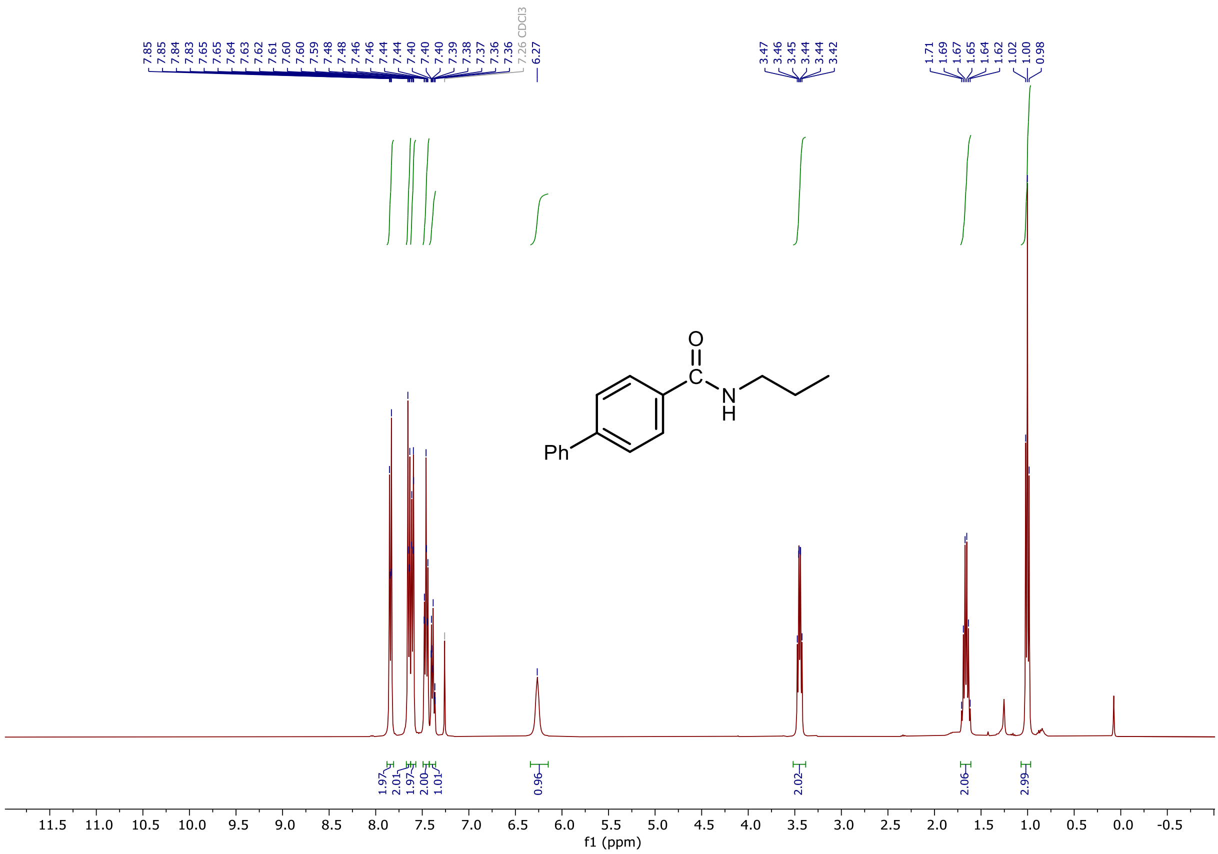

^{13}C-NMR

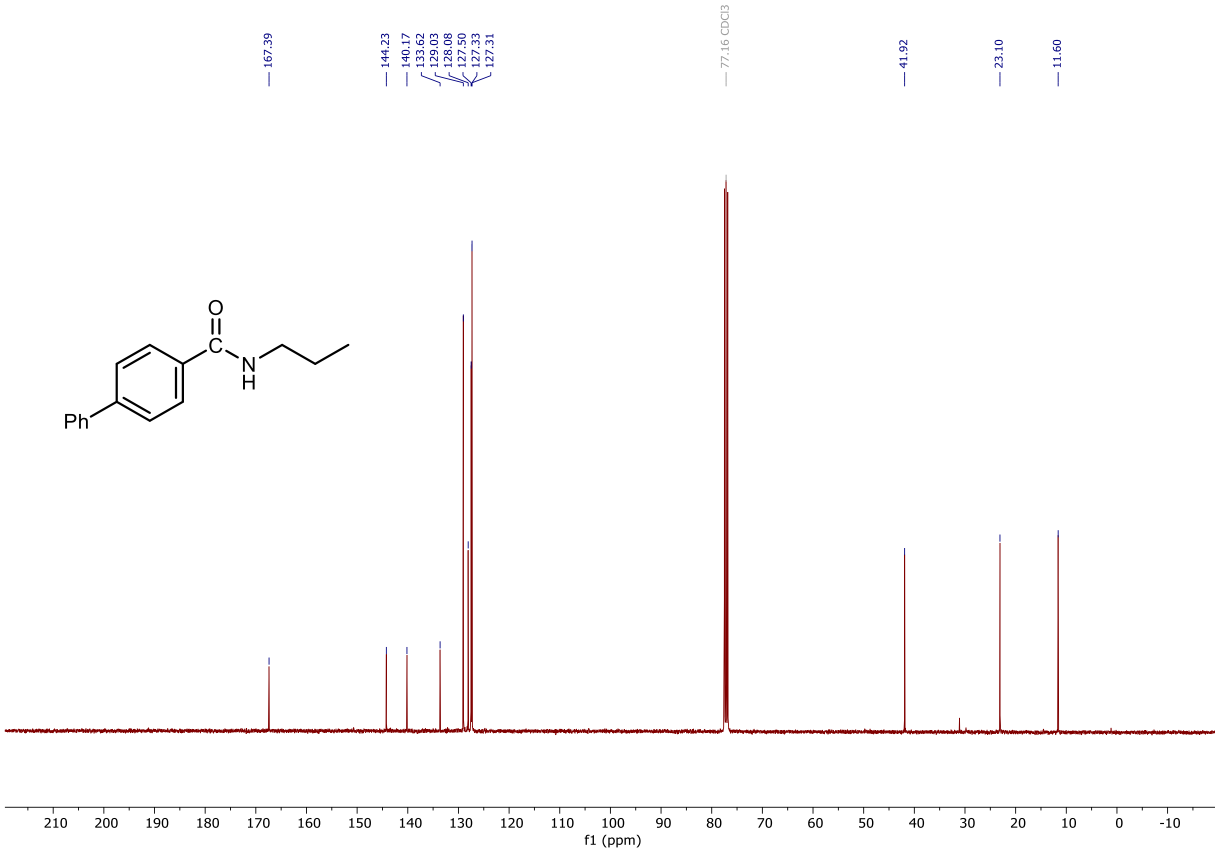

N-Propyl-[1,1'-biphenyl]-4-^{13}C-carboxamide (^{13}C-1a)

^{1}H-NMR

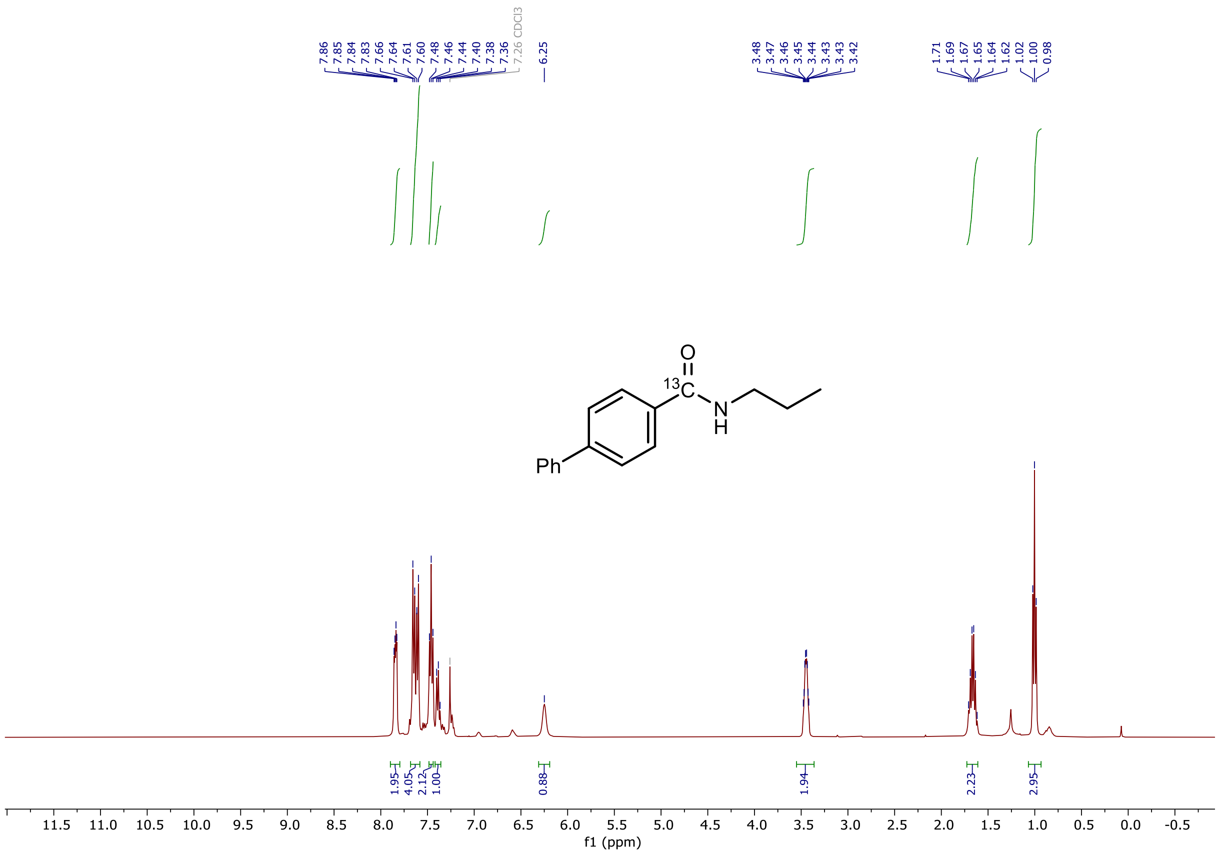

^{13}C-NMR

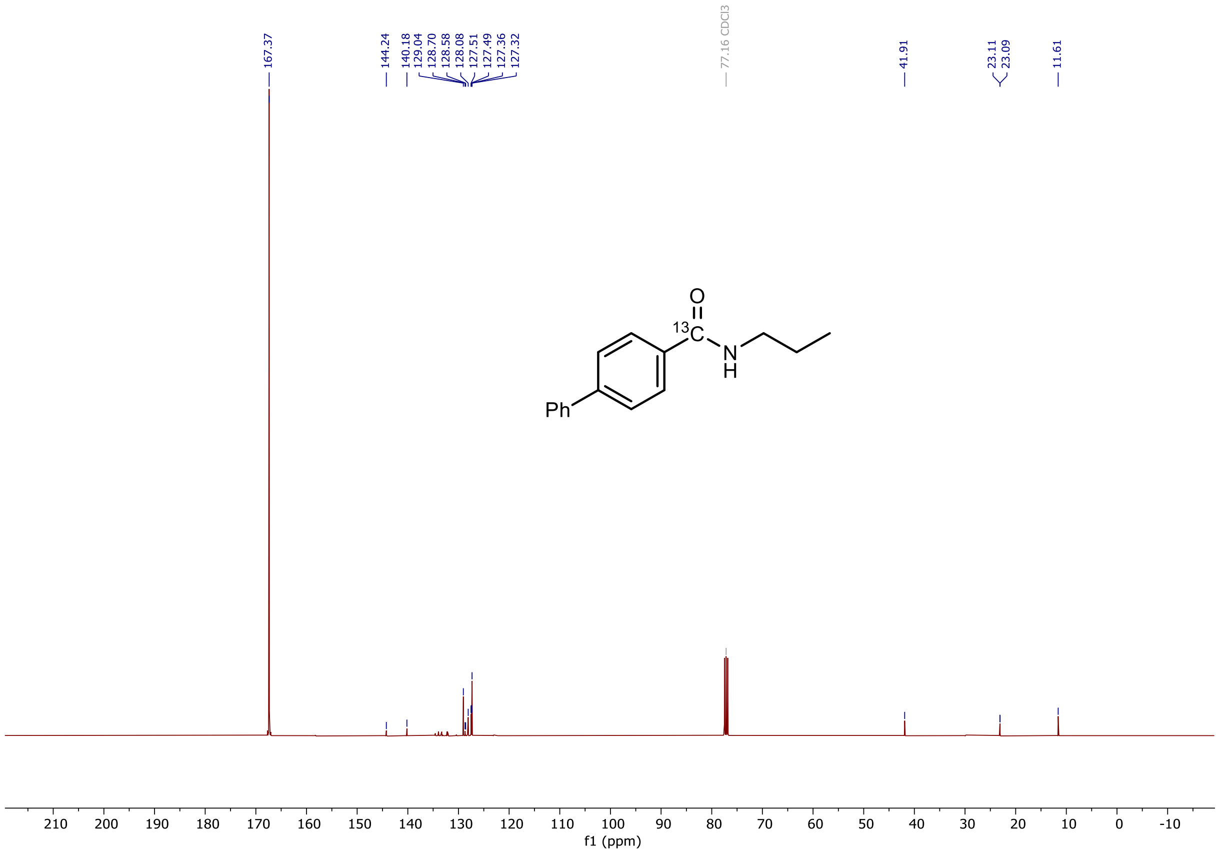

N-Benzyl-[1,1'-biphenyl]-4-carboxamide (2a)

^{1}H-NMR

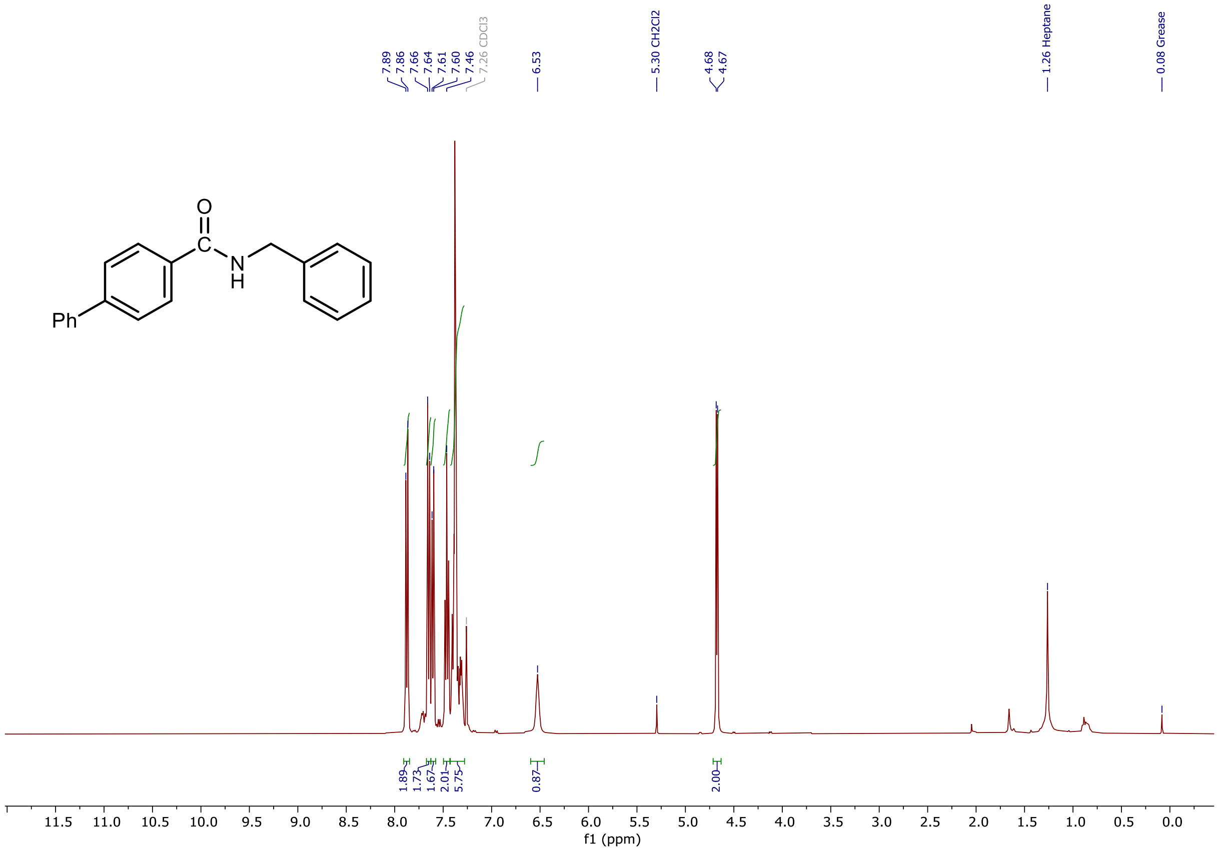

^{13}C-NMR

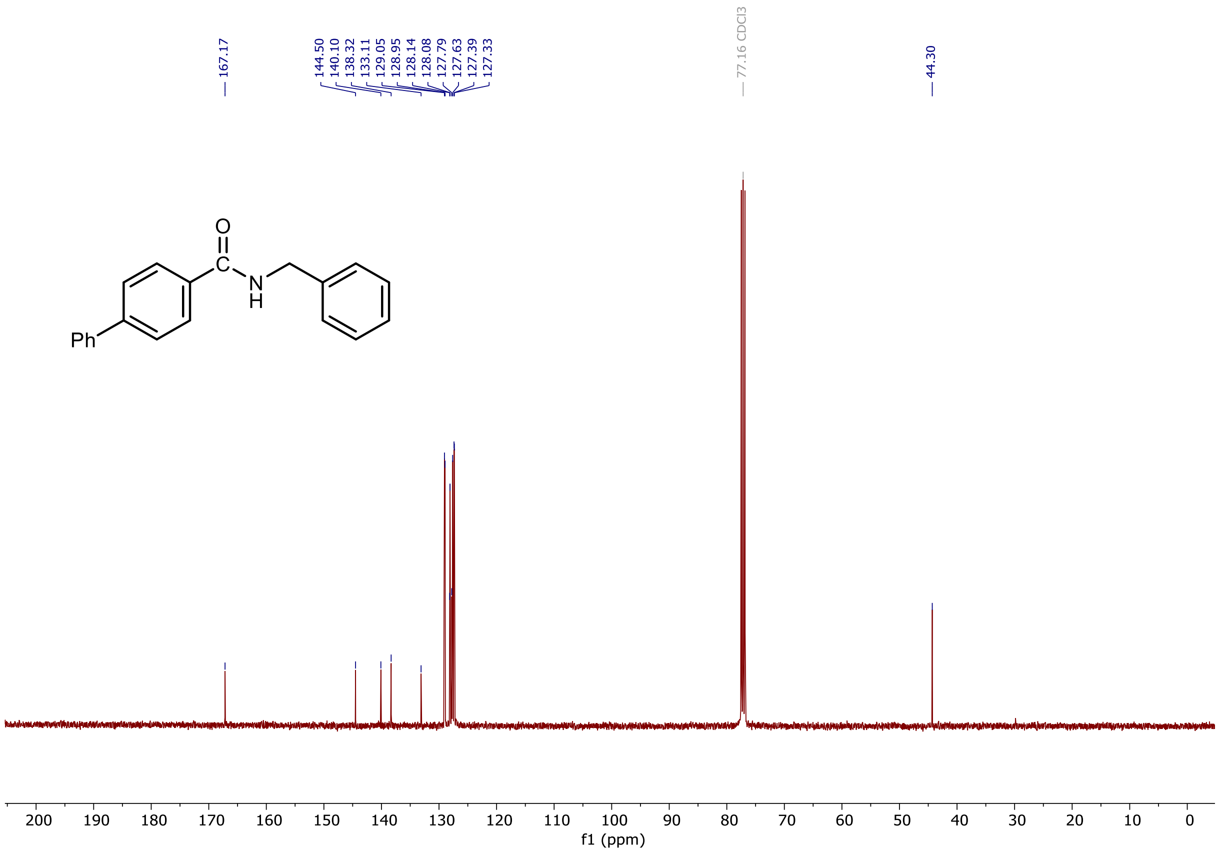

N-(2,4-Difluorobenzyl)-[1,1'-biphenyl]-4-carboxamide (3a)

^{1}H-NMR

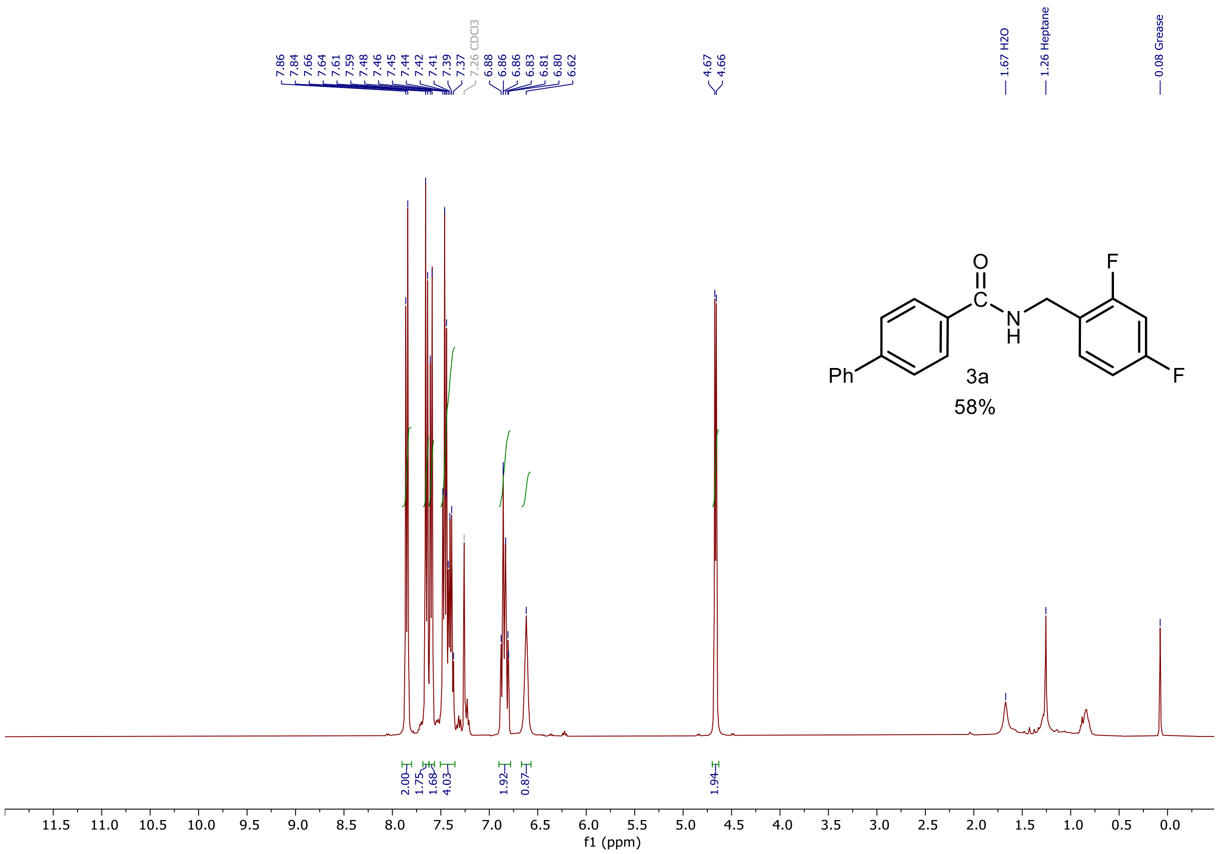

^{13}C-NMR

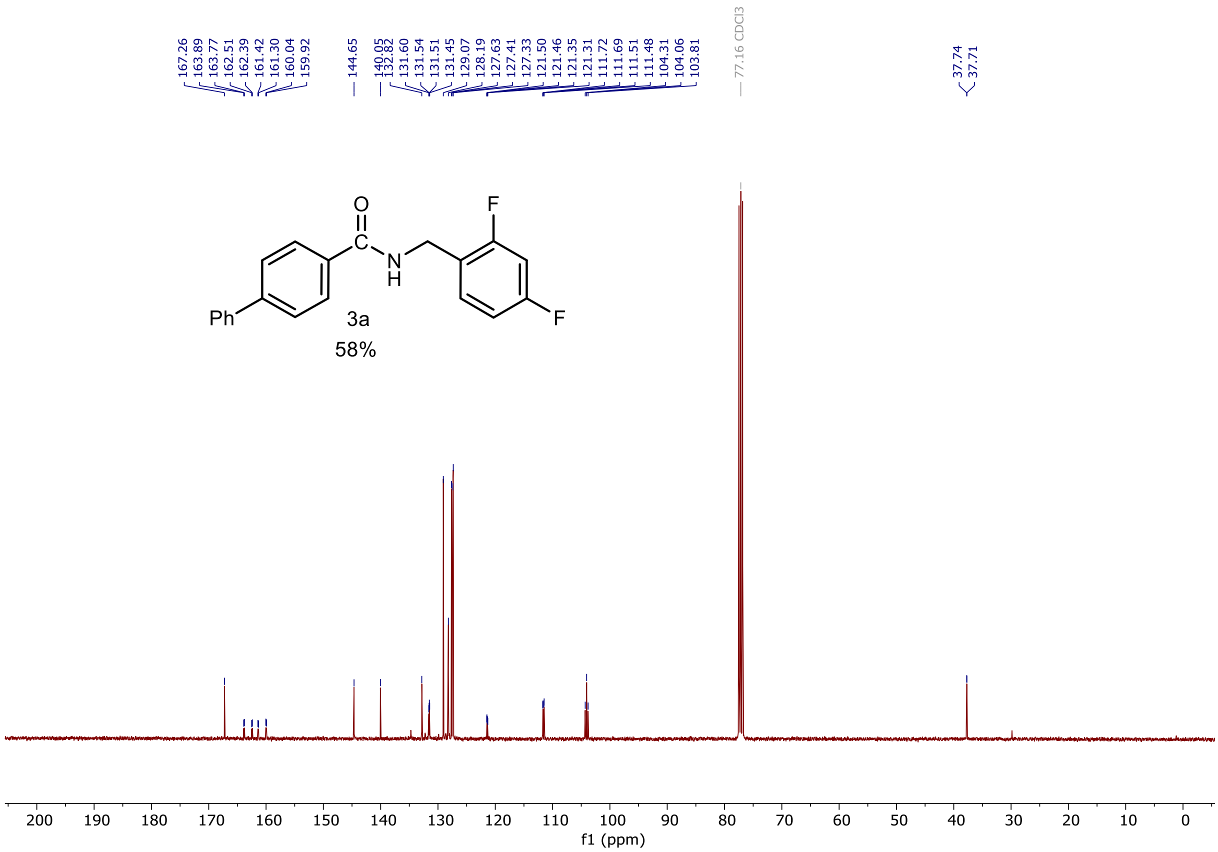

^{19}F-NMR

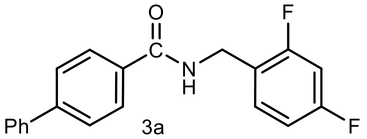

3a
58%

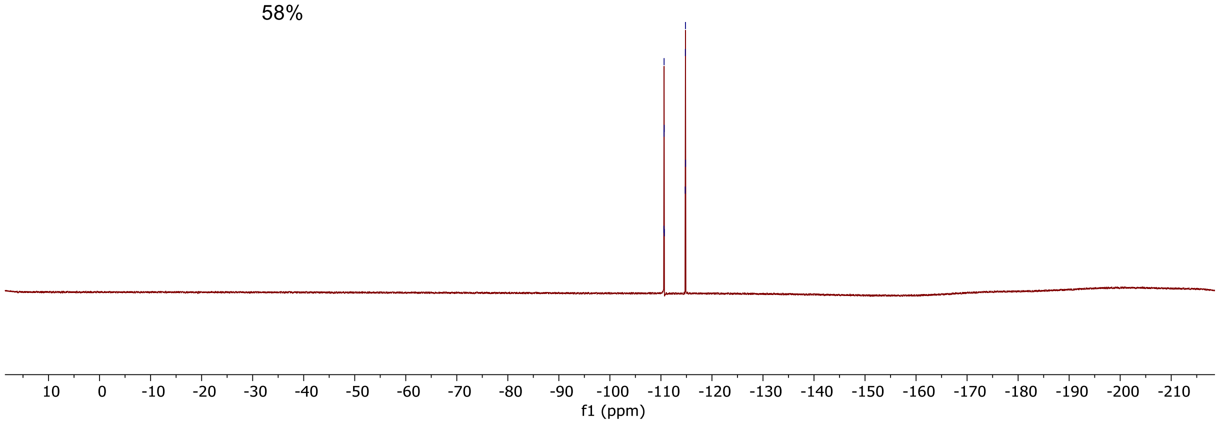
-110.59
-110.61
-110.63
-110.65
-110.67
-114.77
-114.79
-114.81
-114.84
10
0
-10
-20
-30
-40
-50
-60
-70
-80
-90
-100
-110
-120
-130
-140
-150
-160
-170
-180
-190
-200
-210
f1 (ppm)

N-Methyl-[1,1'-biphenyl]-4-carboxamide (4a)

^{1}H-NMR

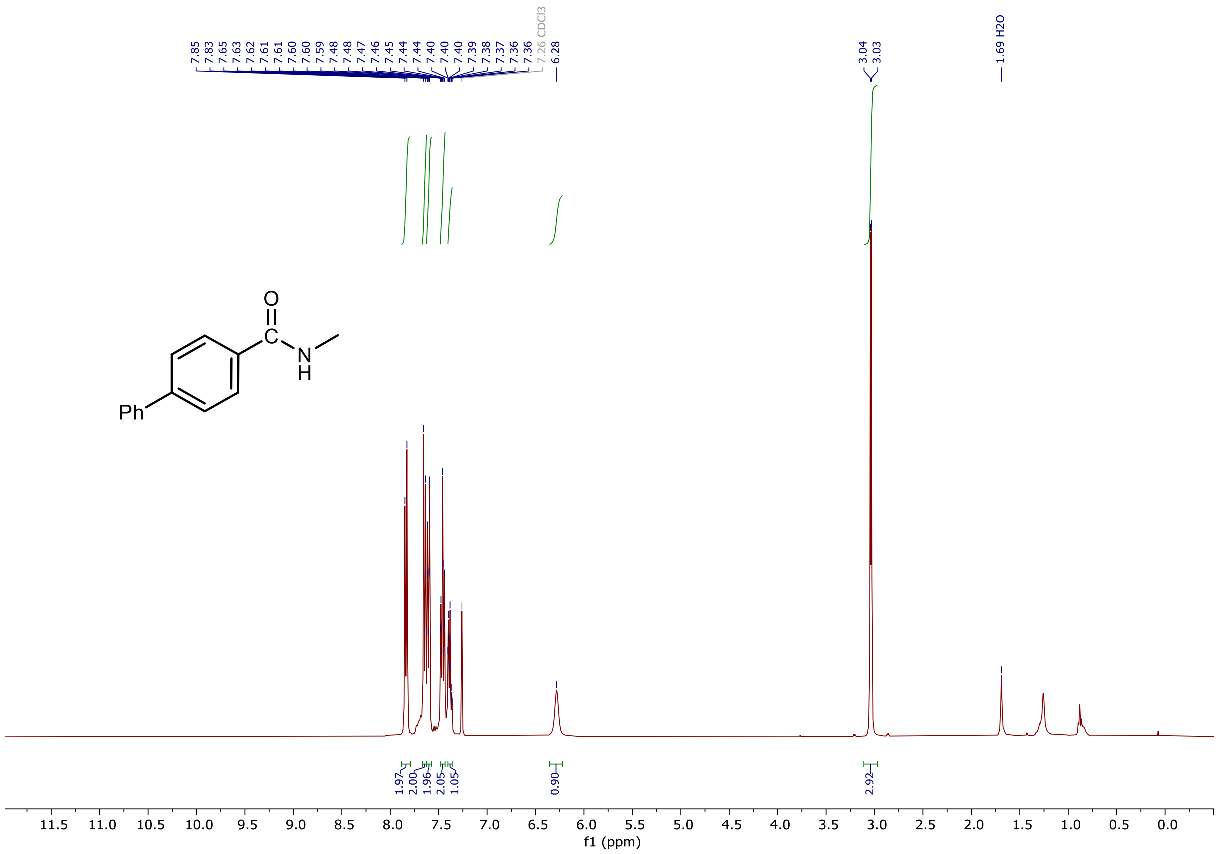

^{13}C-NMR

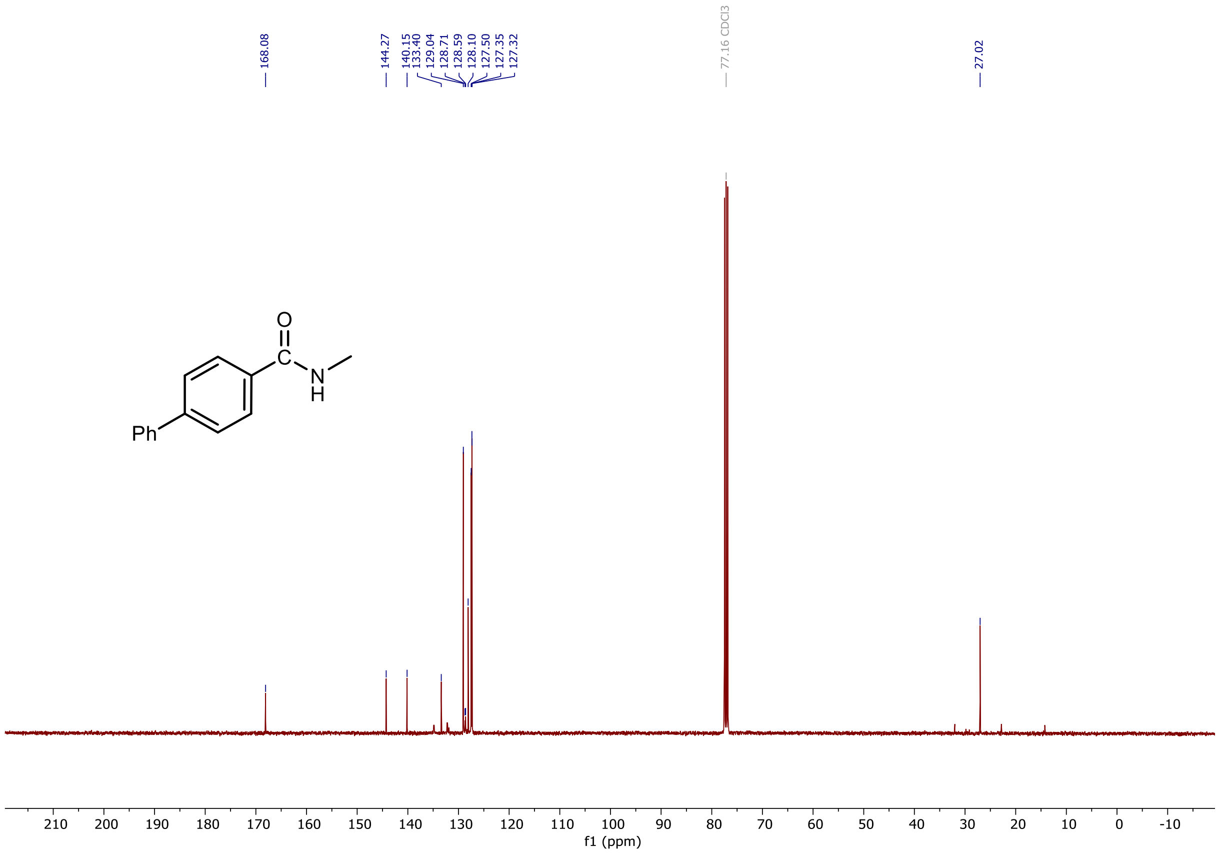

N-Methyl-[1,1'-biphenyl]-4-^{13}C-carboxamide (^{13}C-4a)

^{1}H-NMR

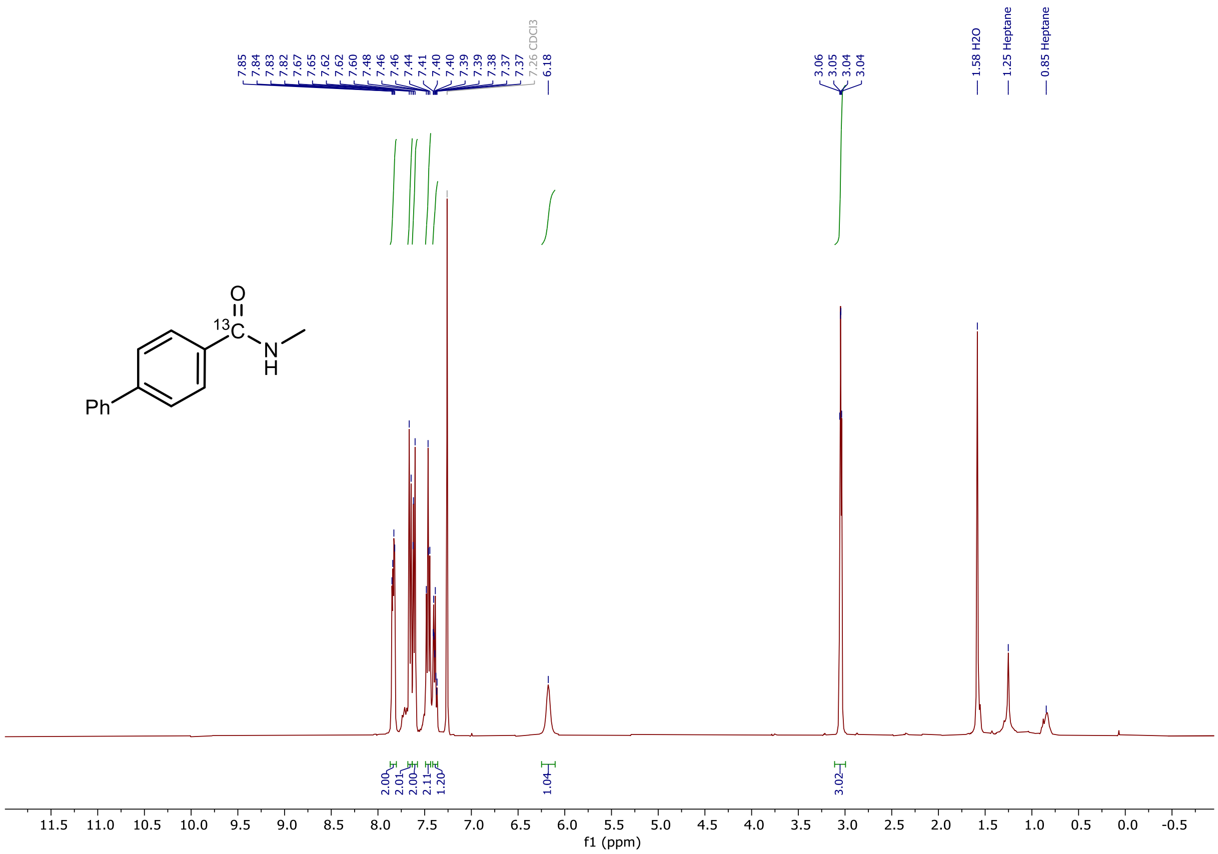

^{13}C-NMR

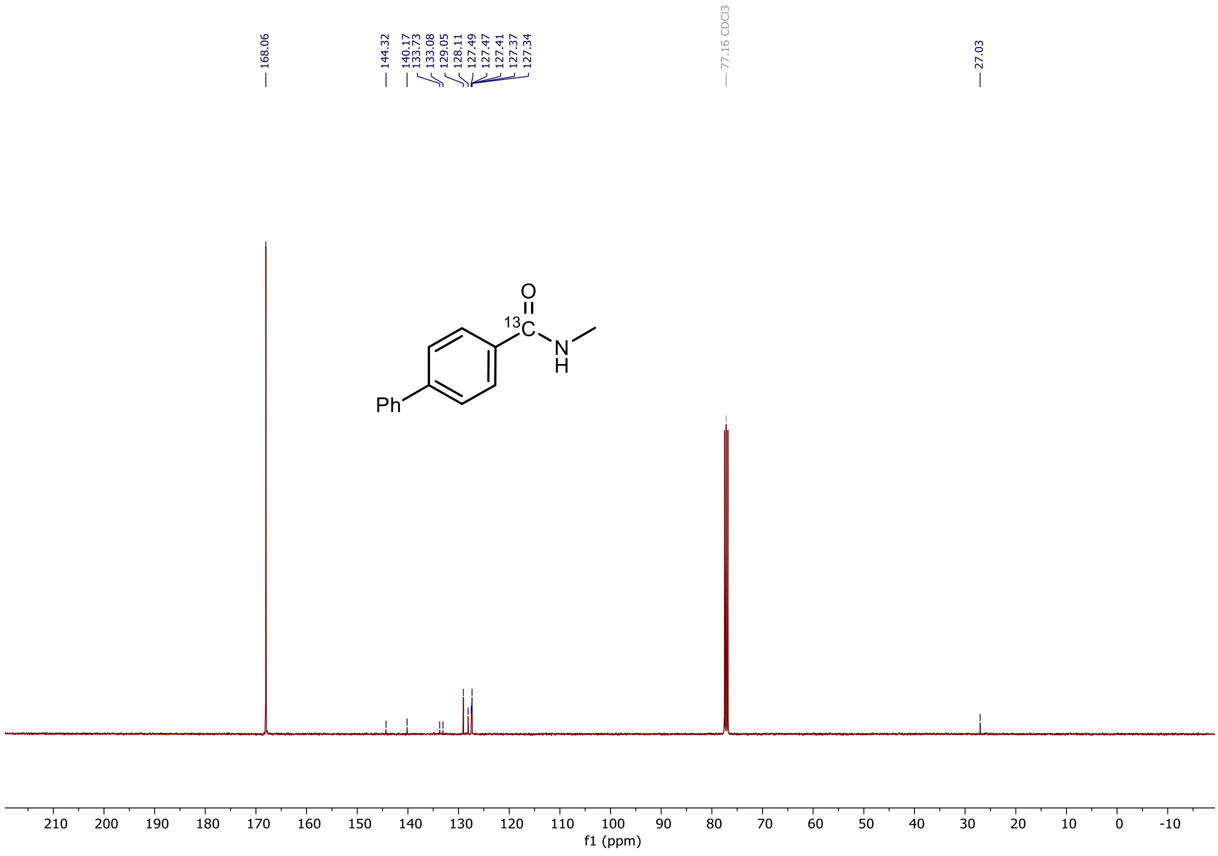

[1,1'-biphenyl]-4-yl(piperidin-1-yl)methanone (5a)

^{1}H-NMR

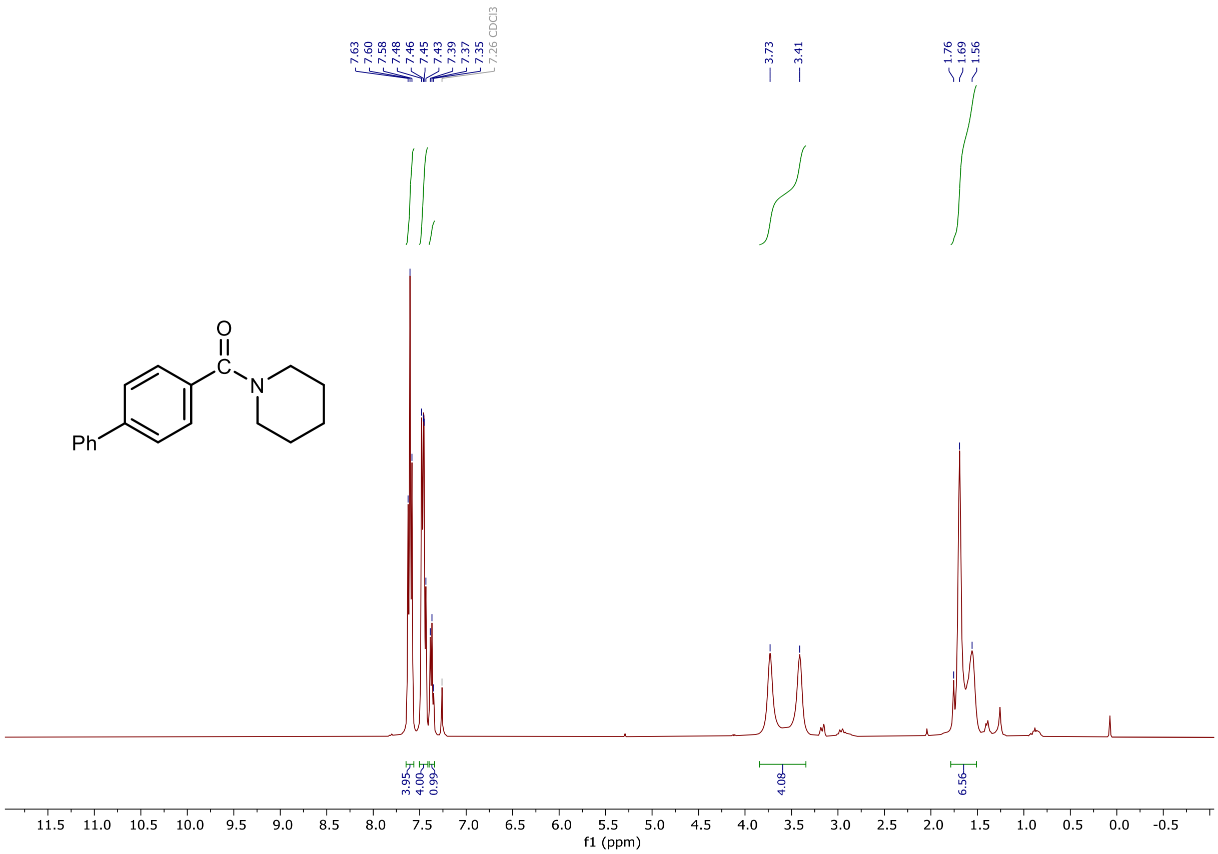

^{13}C-NMR

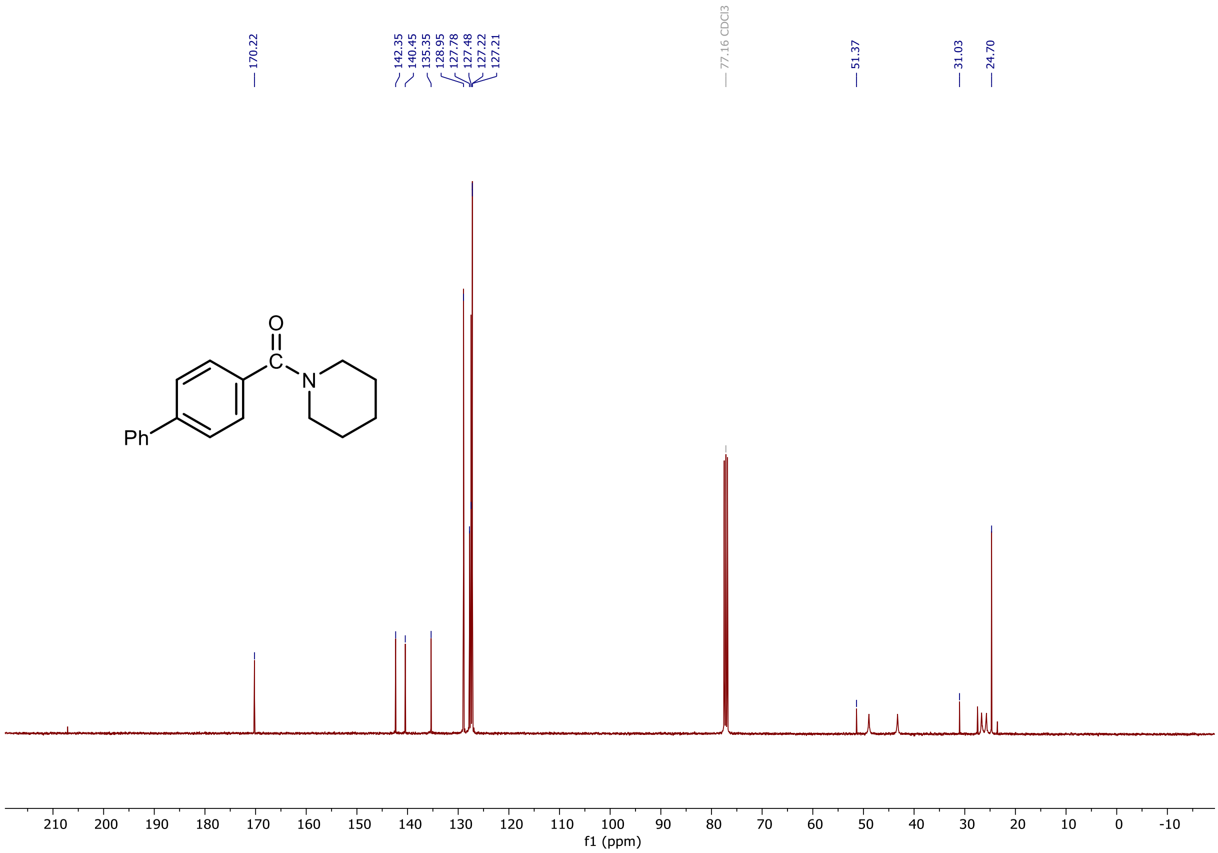

[1,1'-biphenyl]-4-yl(morpholine-1-yl)methanone (6a)

^{1}H-NMR

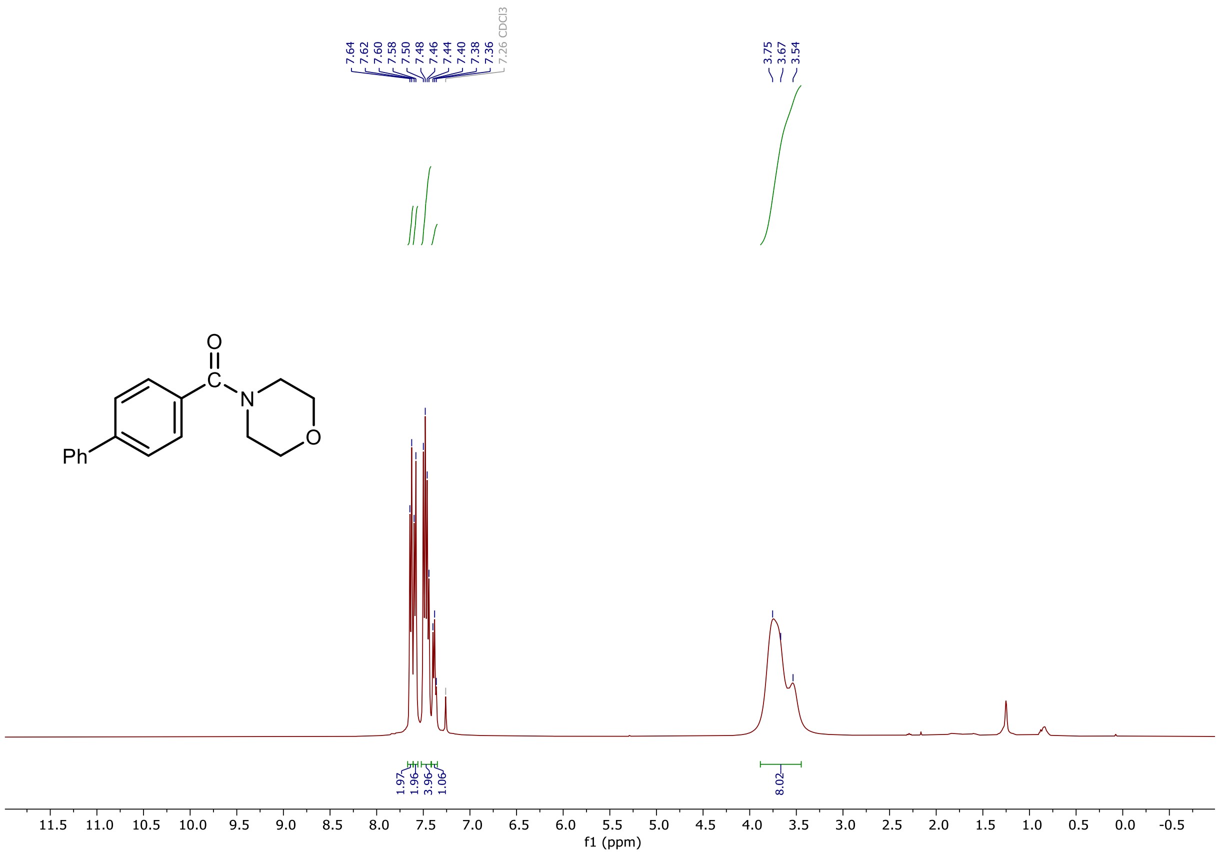

^{13}C-NMR

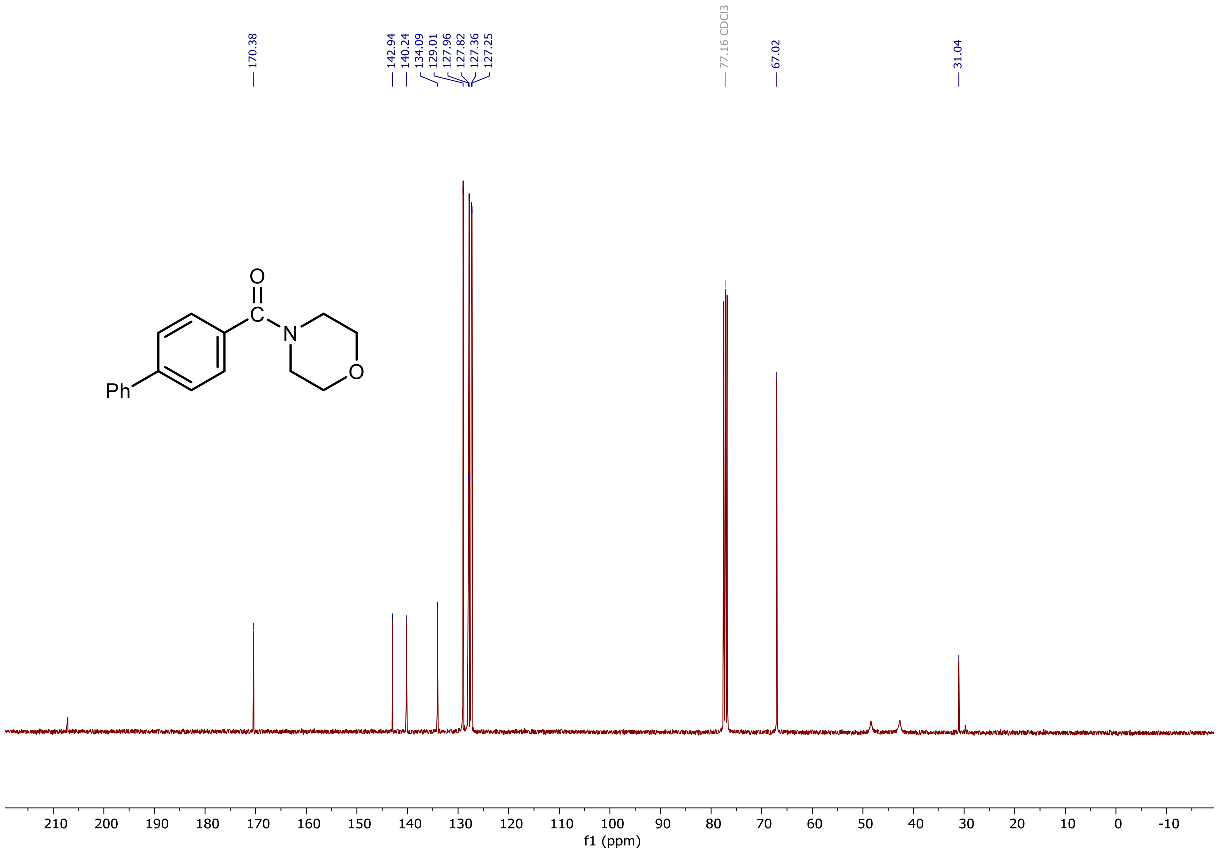

(4-([1,1'-biphenyl]-4-carbonyl)piperazin-1-yl)(cyclopropyl)methanone (7a)

^{1}H-NMR

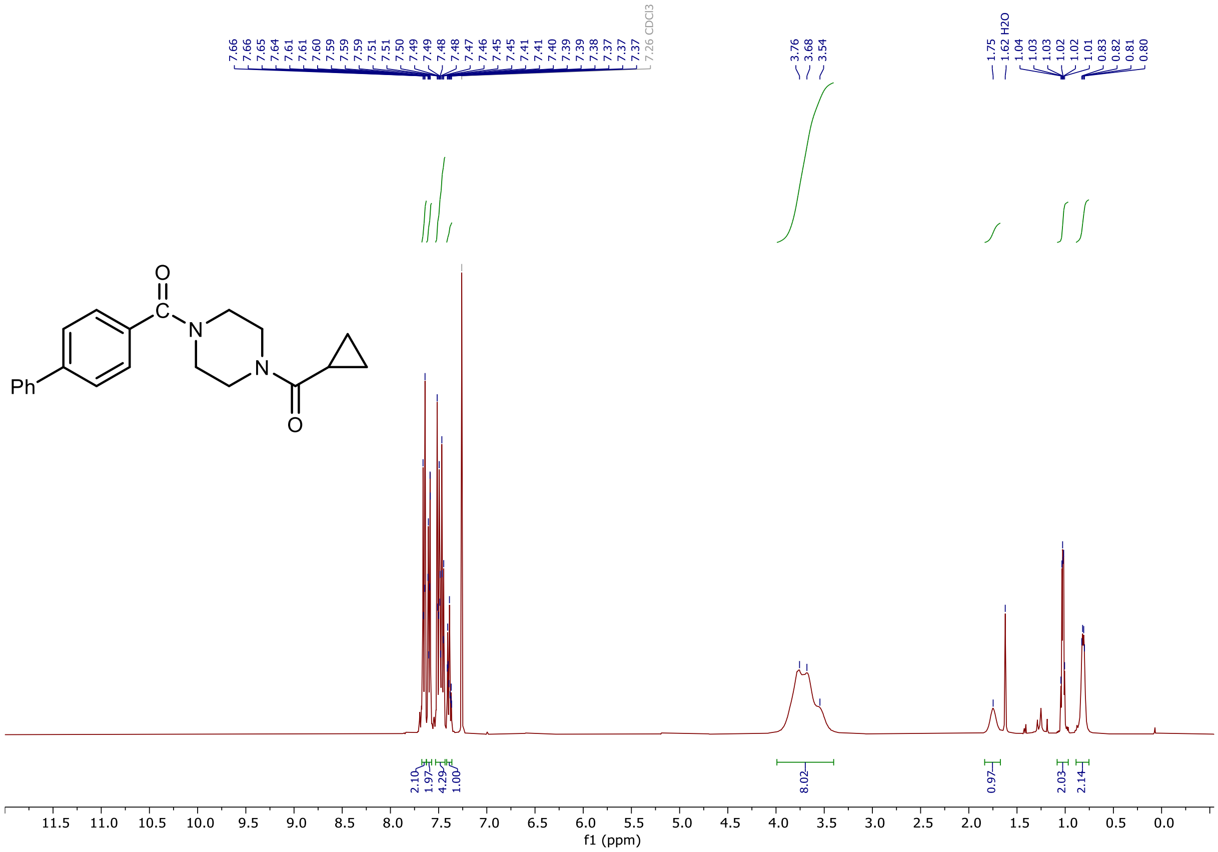

^{13}C-NMR

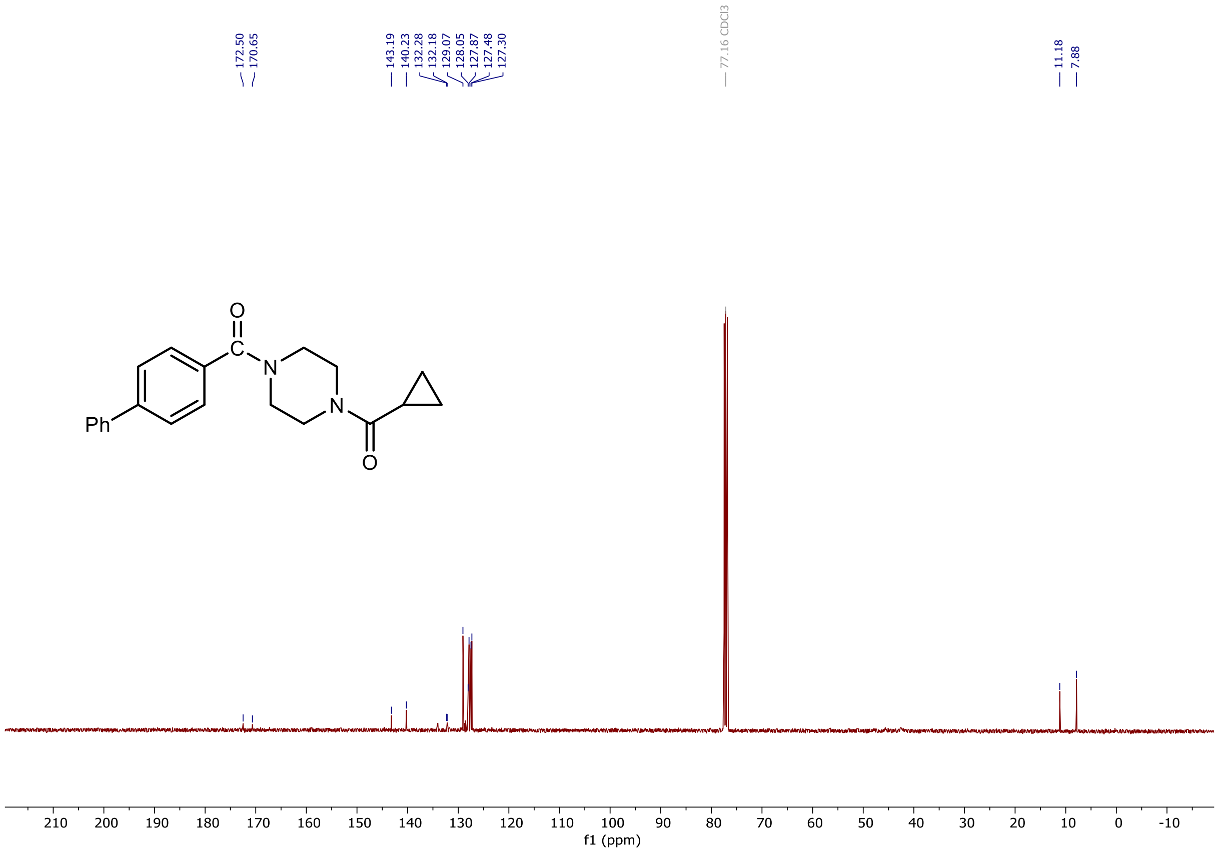

(4-([1,1'-biphenyl]-4-carbonyl)piperazin-1-yl)(cyclopropyl)-^{13}C-methanone (^{13}C-7a)

^{1}H-NMR

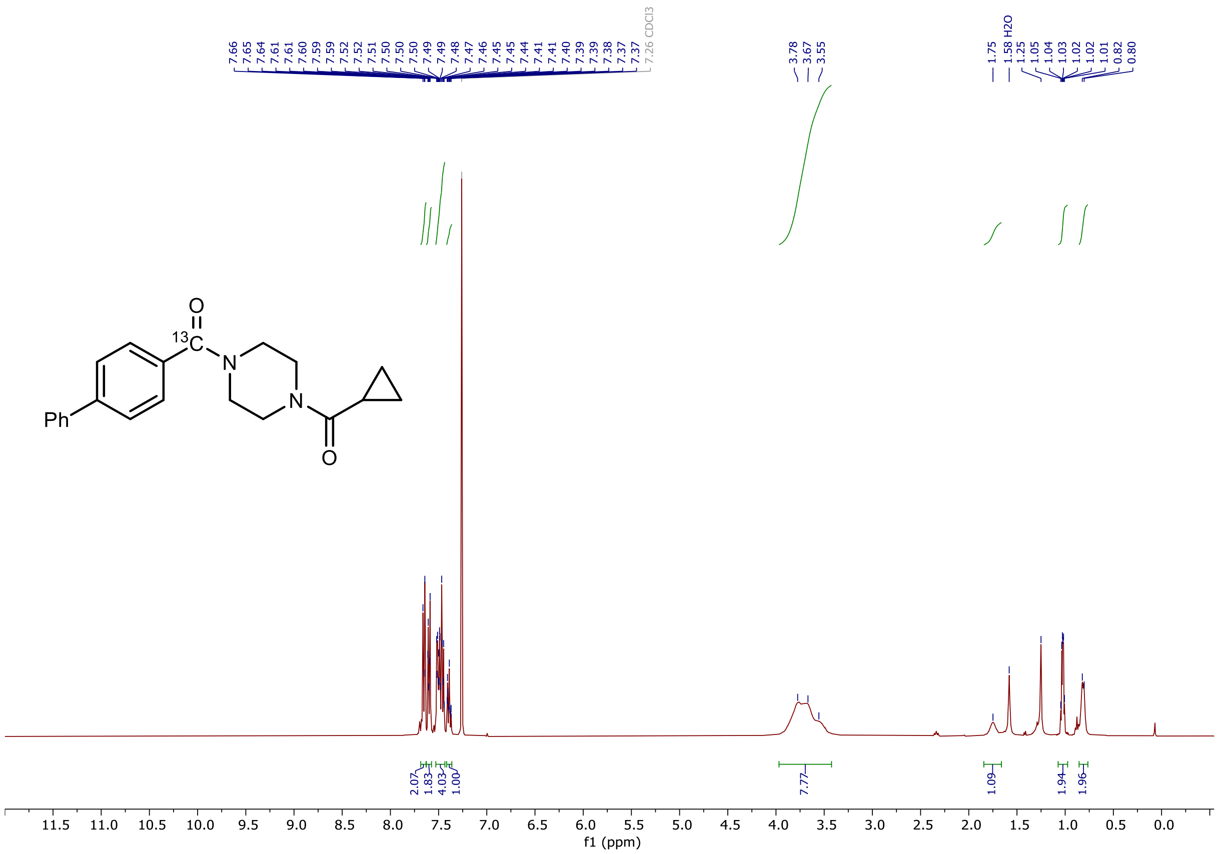

^{13}C-NMR

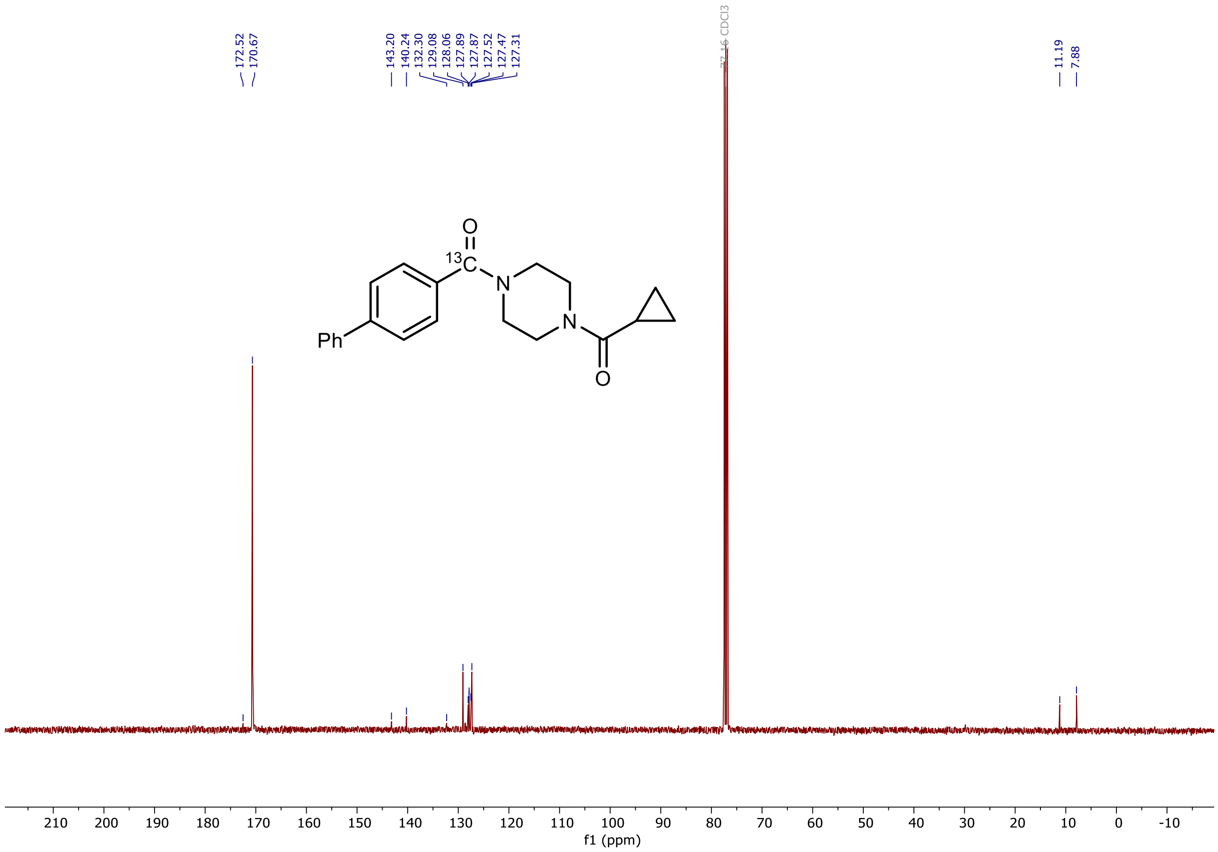

4-(*tert*-Butyl)-*N*-propylbenzamide (1b)

^{1}H-NMR

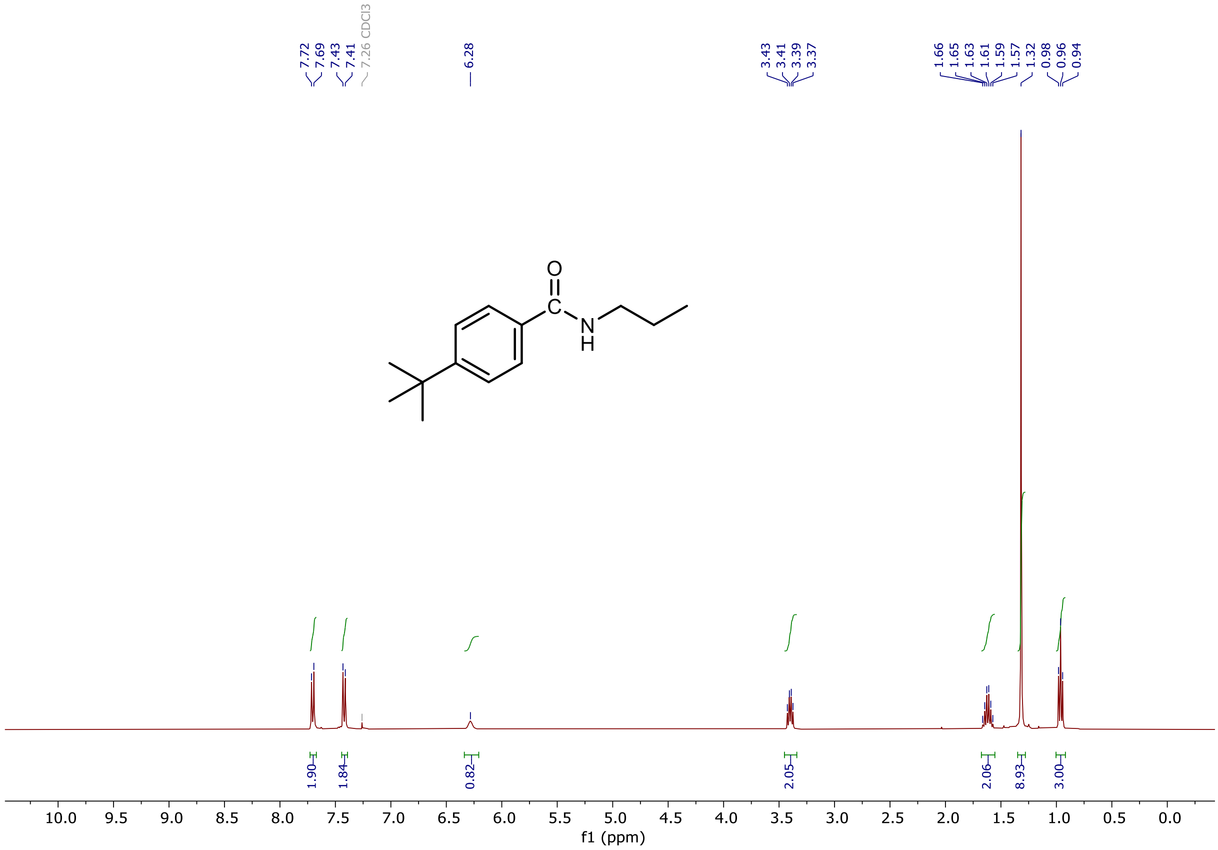

^{13}C-NMR

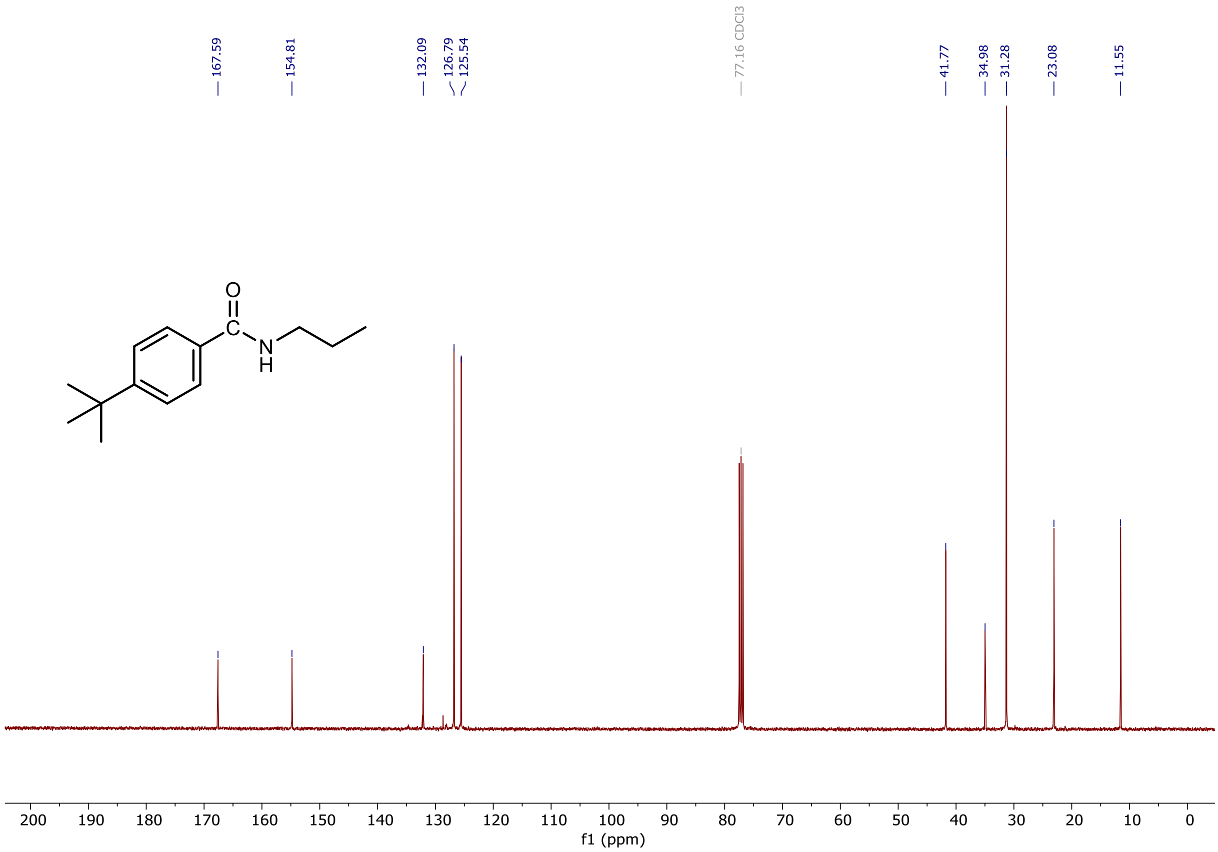

4-(*tert*-Butyl)-*N*-propylbenz-^{13}C-amide (^{13}C-1b)

^{1}H-NMR

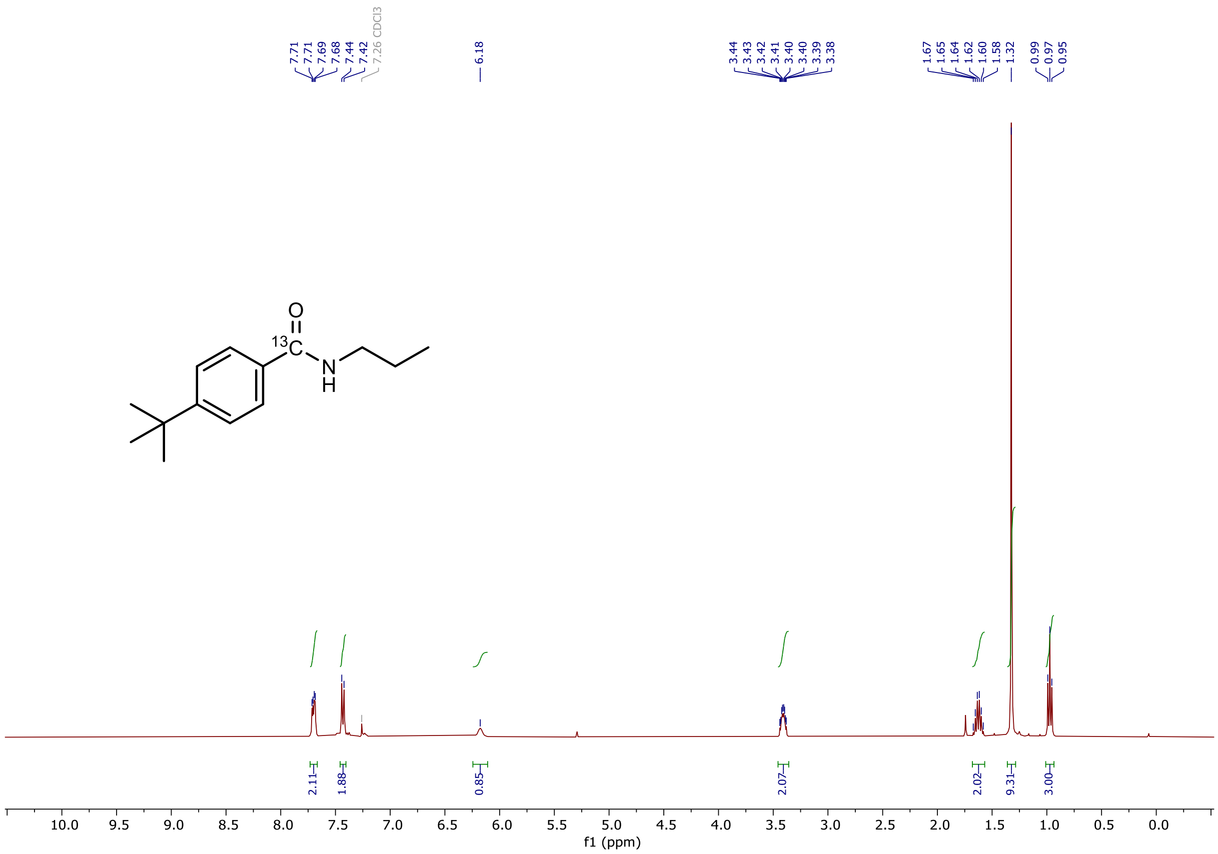

^{13}C-NMR

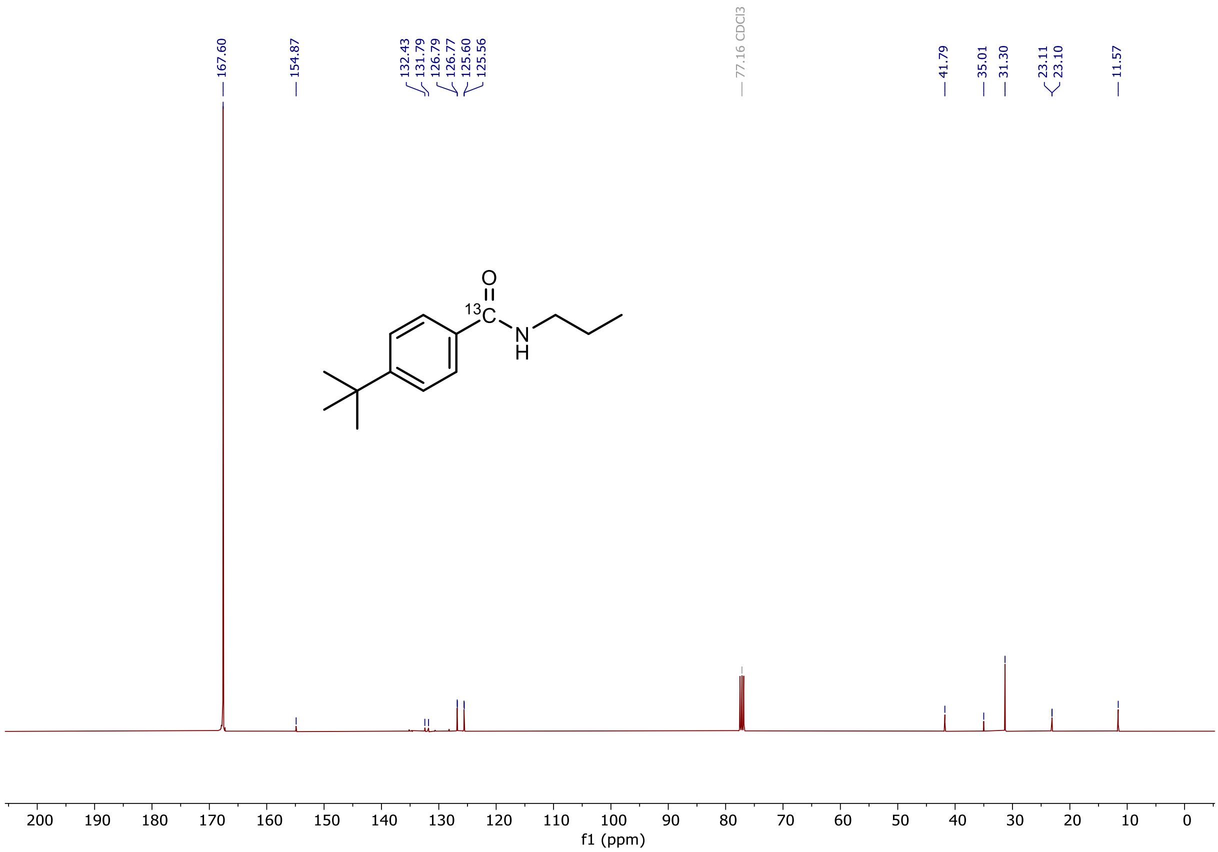

4-Methoxy-*N*-propylbenzamide (1c)

^{1}H-NMR

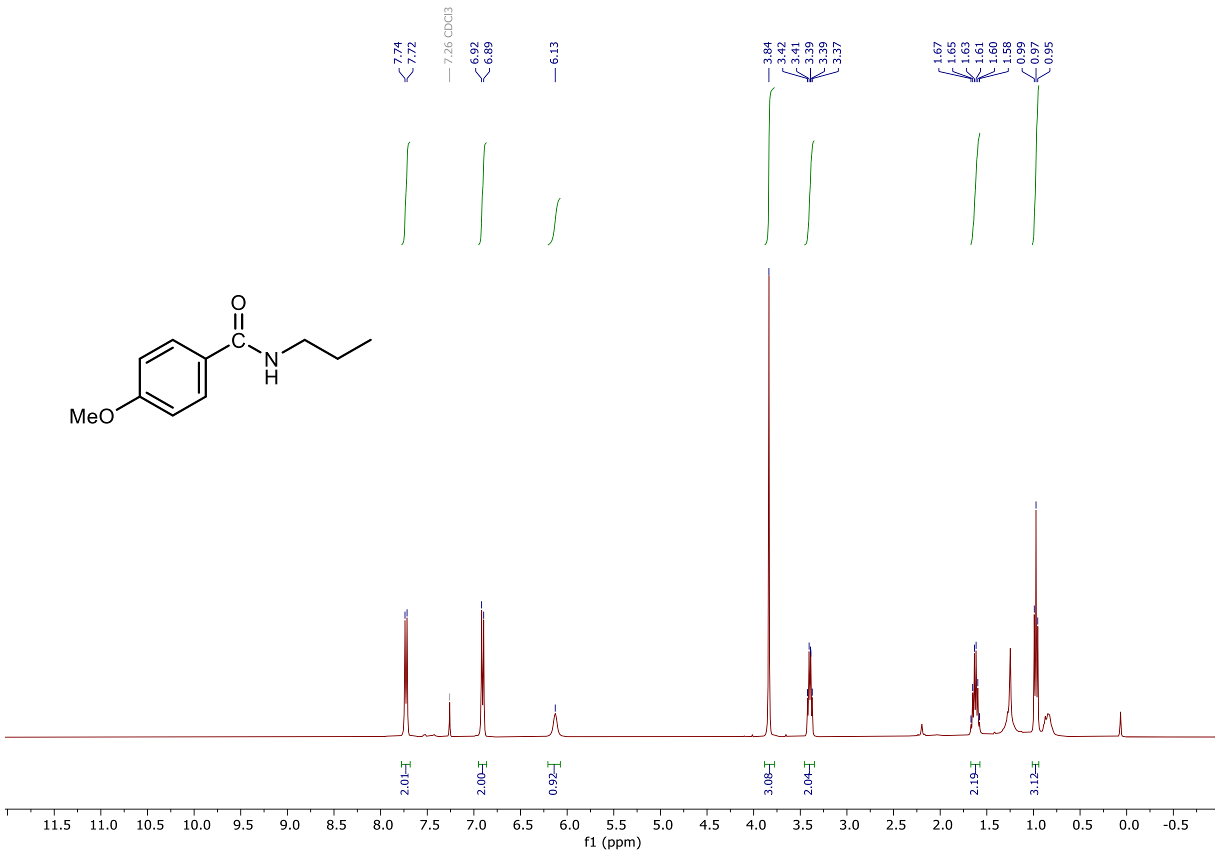

^{13}C-NMR

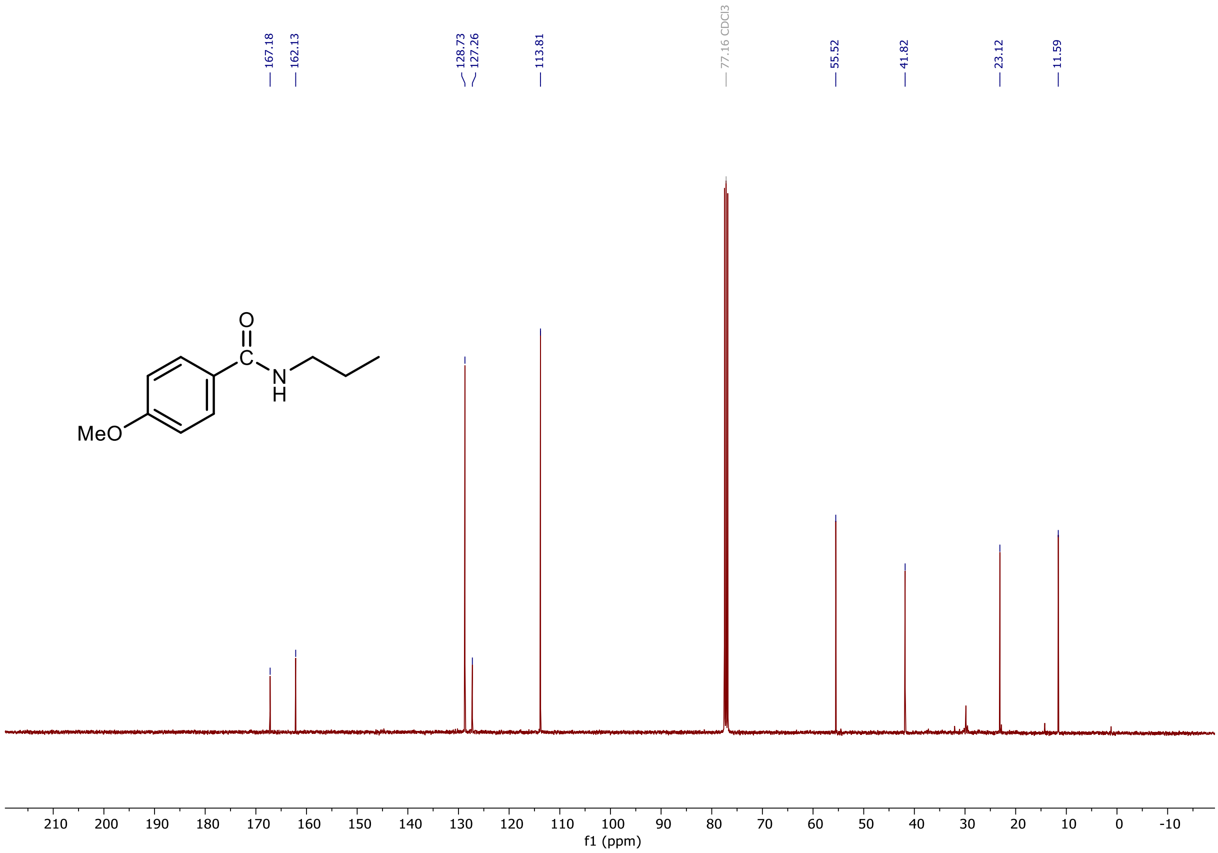

4-(Methylthio)-*N*-propylbenzamide (1d)

^{1}H-NMR

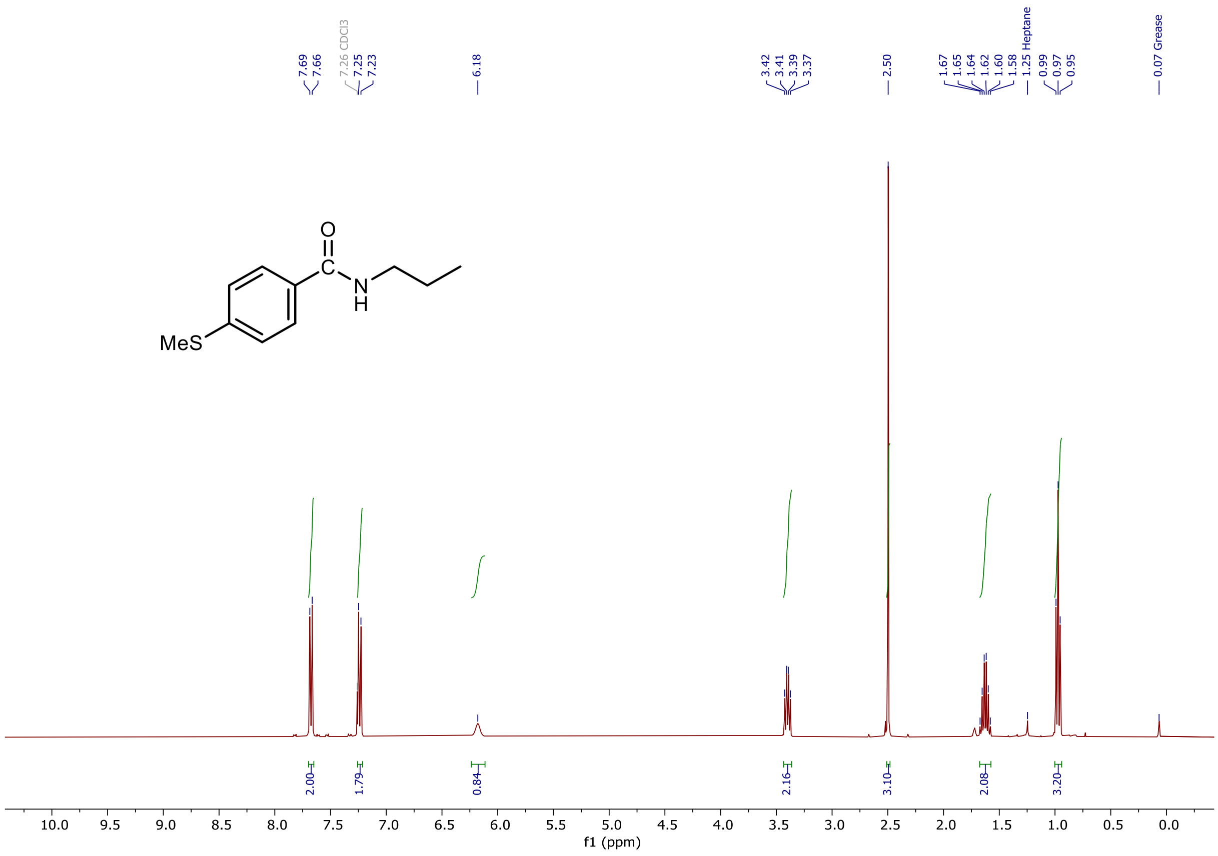

^{13}C-NMR

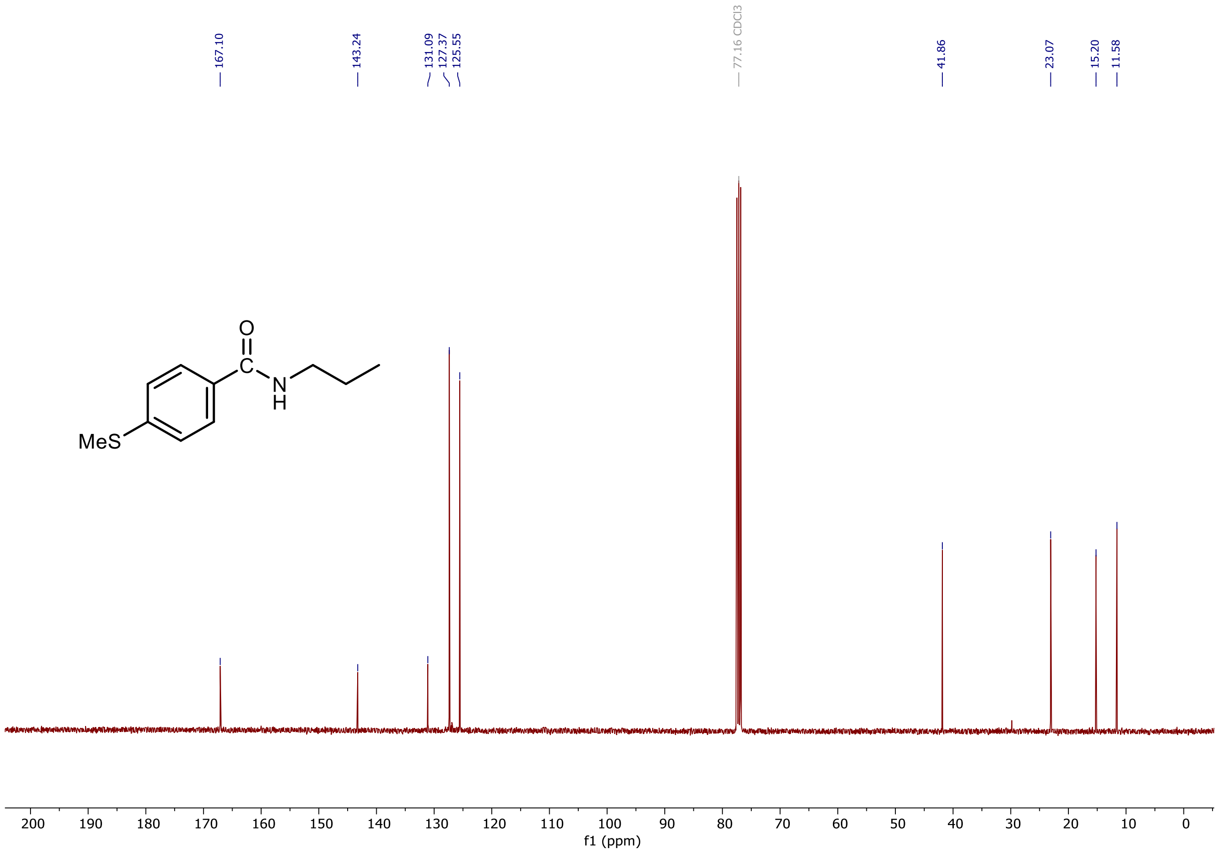

4-Cyano-*N*-propylbenzamide (1e)

^{1}H-NMR

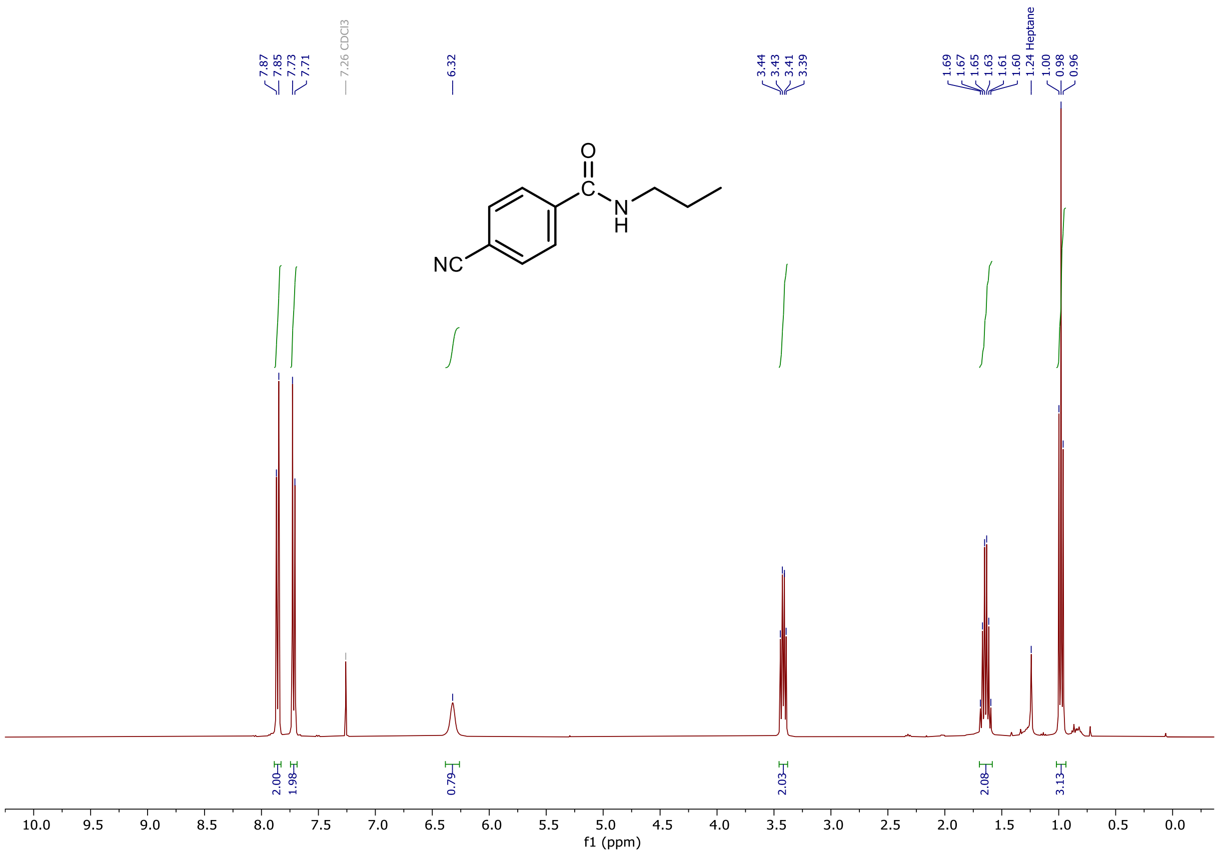

^{13}C-NMR

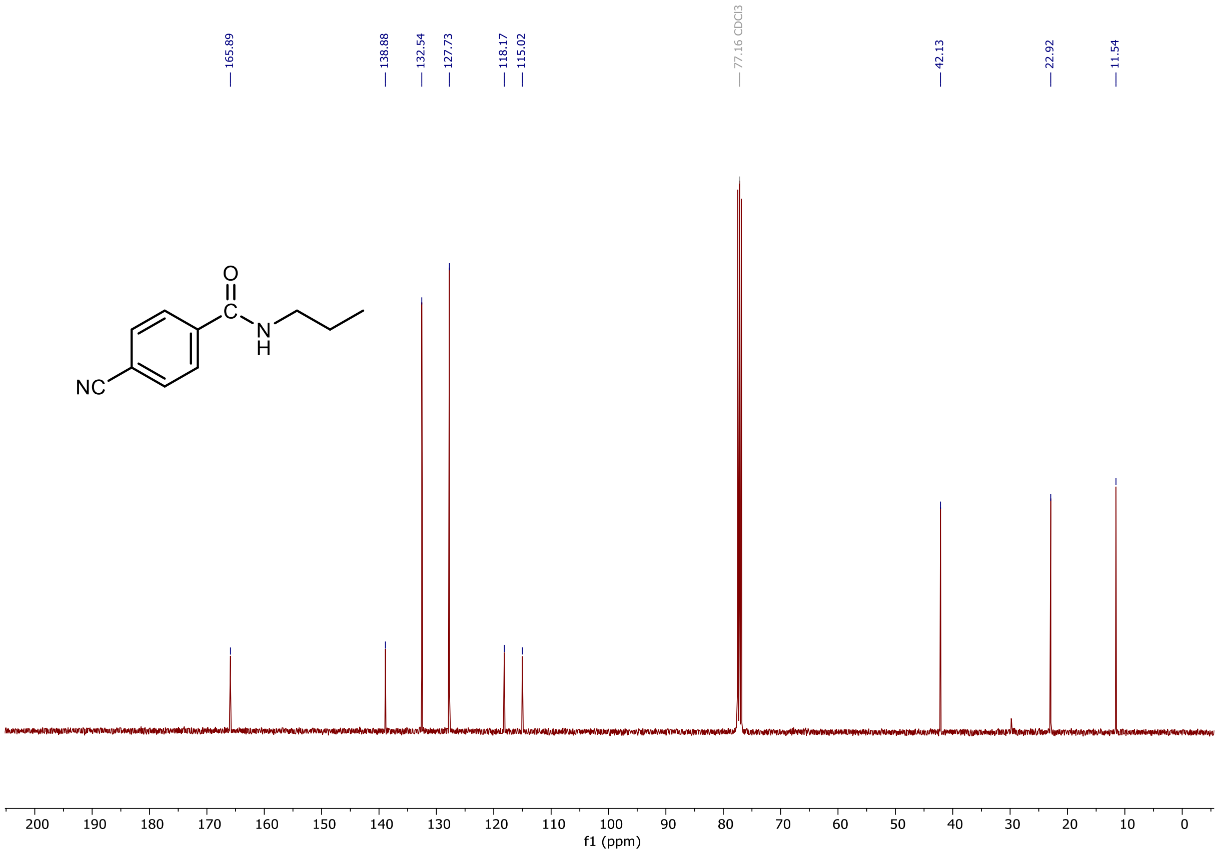

4-Fluoro-*N*-propylbenzamide (1f)

^{1}H-NMR

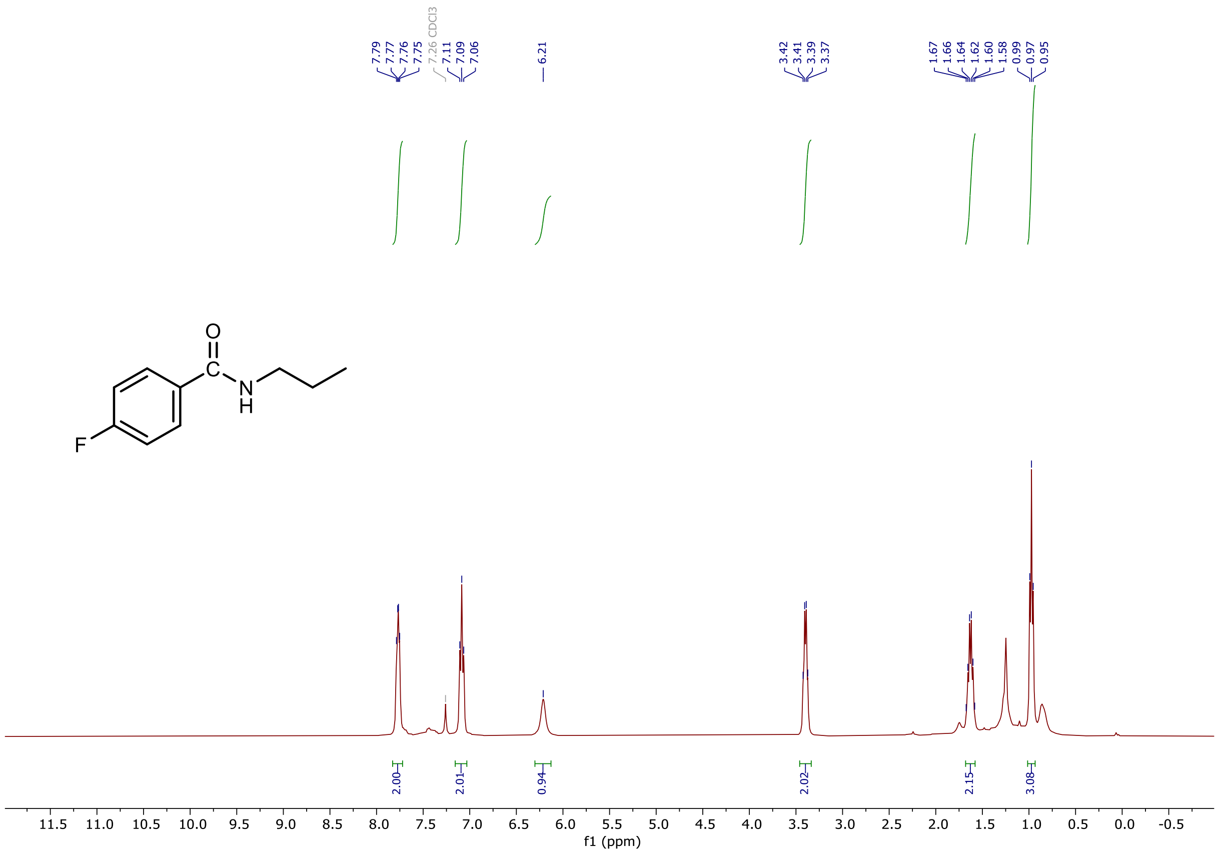

^{13}C-NMR

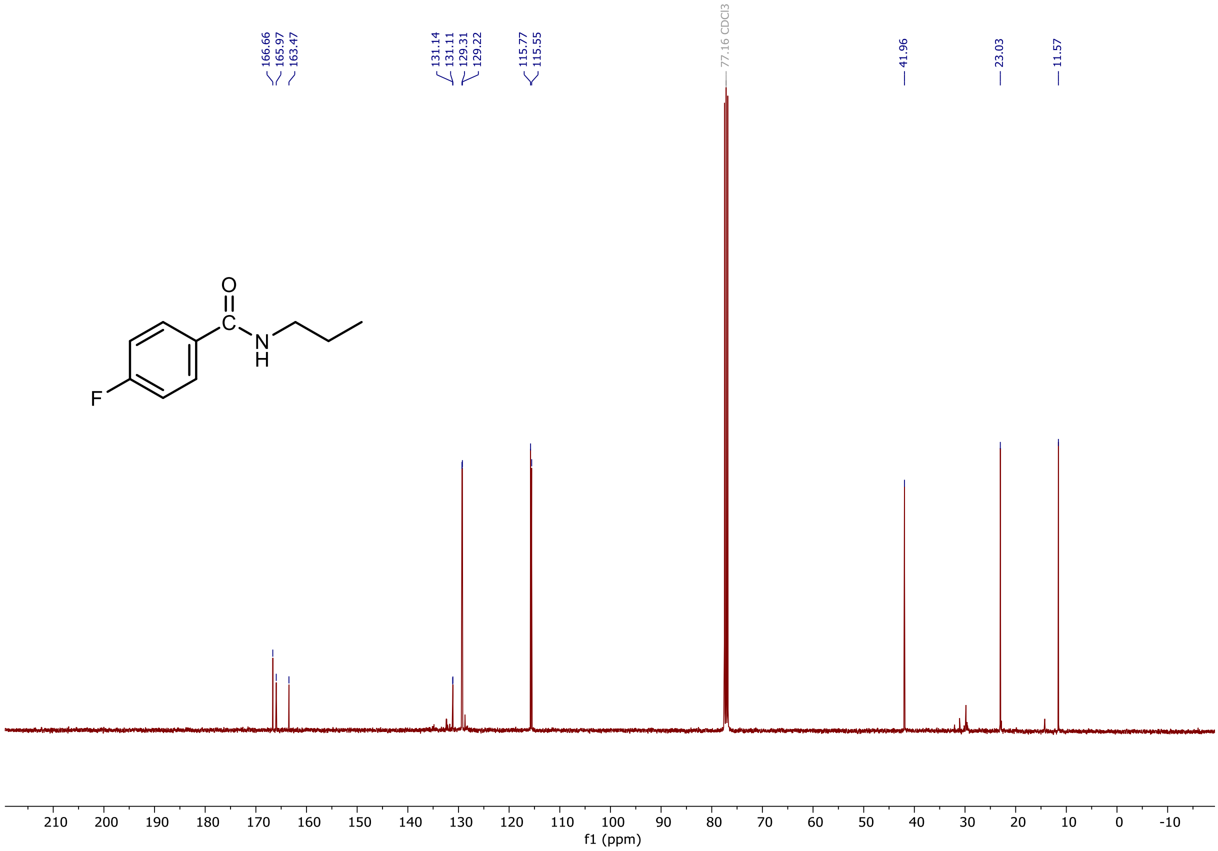

^{19}F-NMR

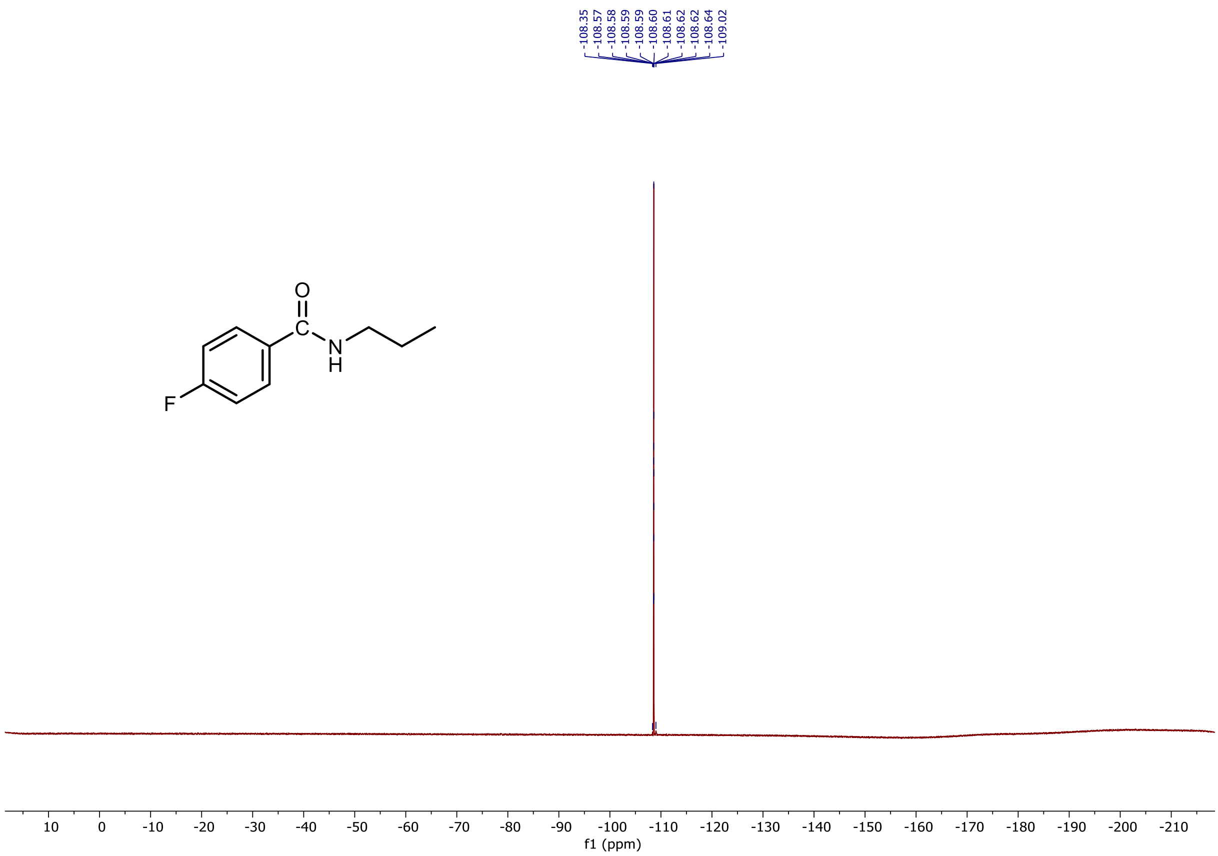
-108.35
-108.57
-108.58
-108.59
-108.59
-108.60
-108.61
-108.62
-108.62
-108.64
-109.02
O
C
N
H
F
10
0
-10
-20
-30
-40
-50
-60
-70
-80
-90
-100
-110
-120
-130
-140
-150
-160
-170
-180
-190
-200
-210
f1 (ppm)

N-Propyl-4-(2,2,2-trifluoroacetyl)benzamide (1g)

^{1}H-NMR

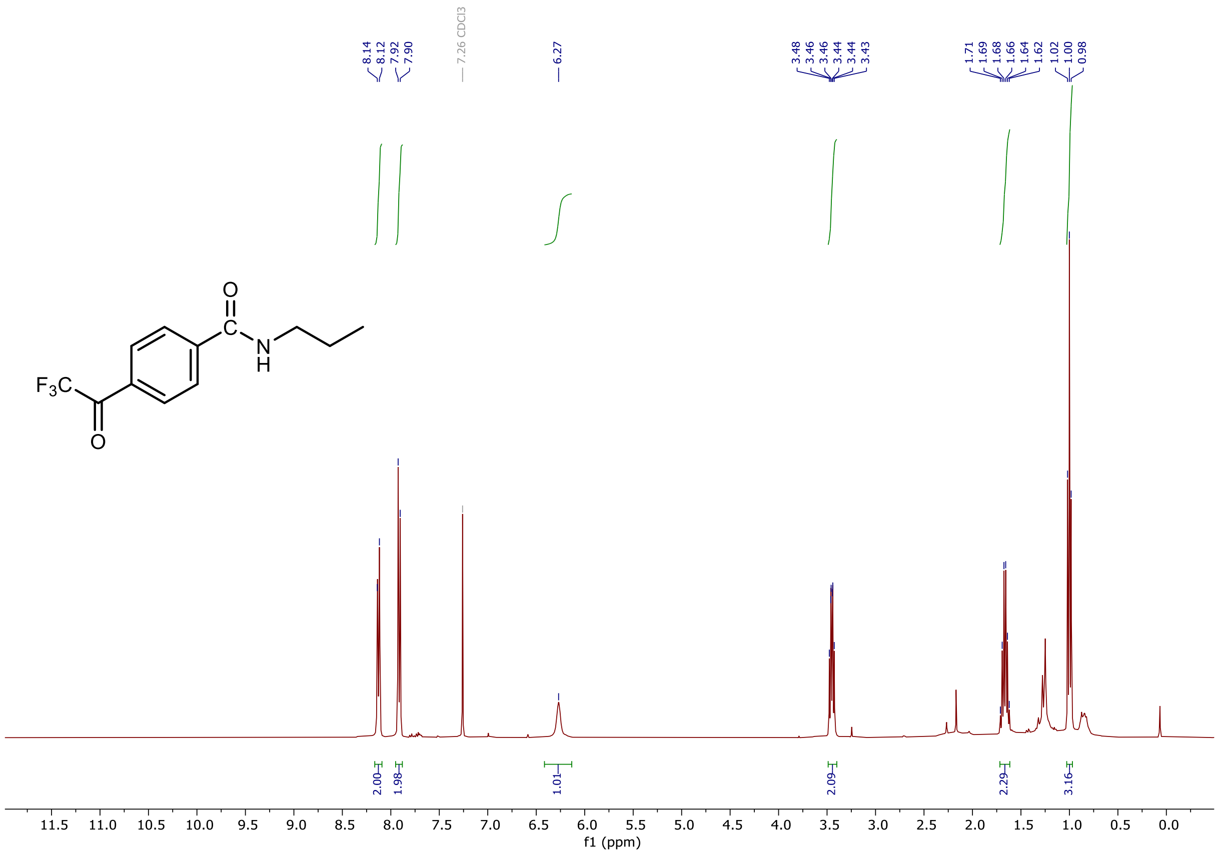

^{13}C-NMR

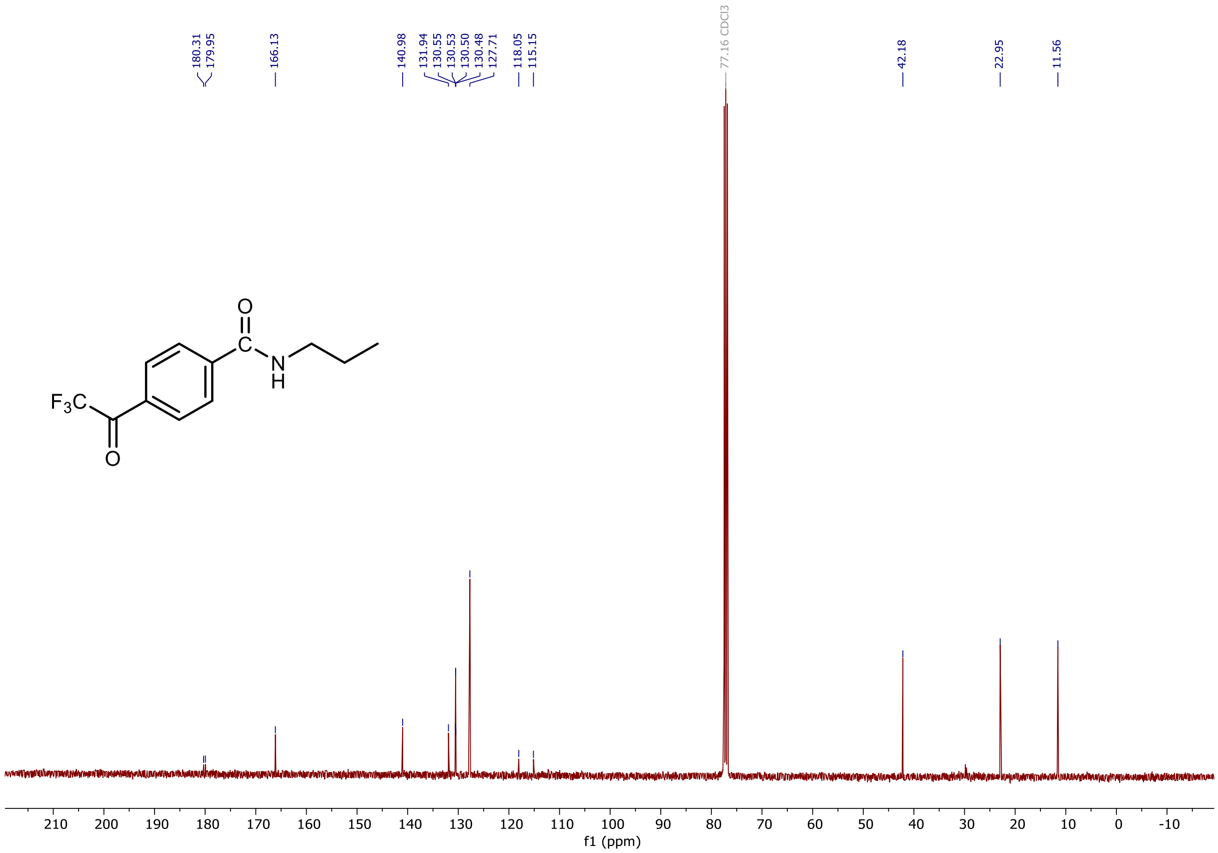

^{19}F-NMR

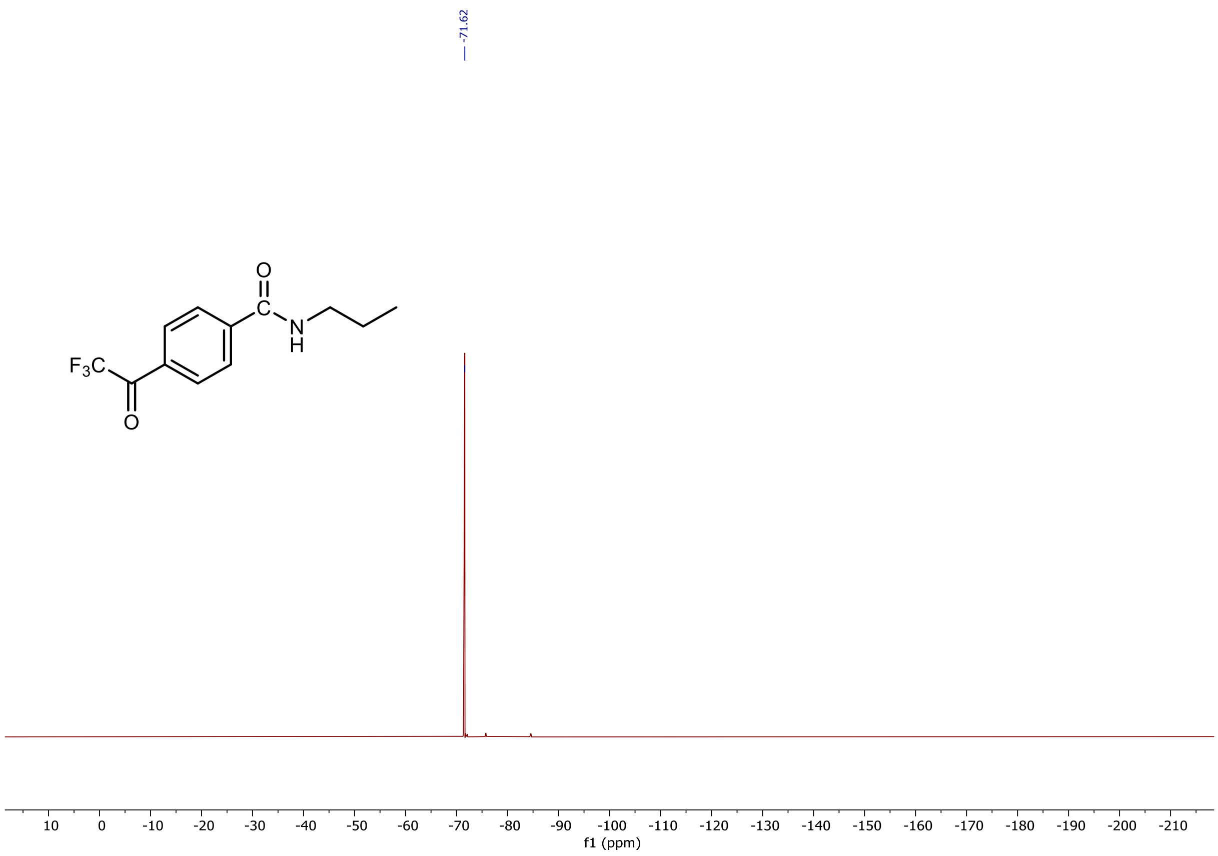
-71.62
F3C
O
C
N
H
f1 (ppm)
10 0 -10 -20 -30 -40 -50 -60 -70 -80 -90 -100 -110 -120 -130 -140 -150 -160 -170 -180 -190 -200 -210

N-Propyl-4-(2,2,2-trifluoro-1-hydroxyethyl)benzamide (1h)

^{1}H-NMR

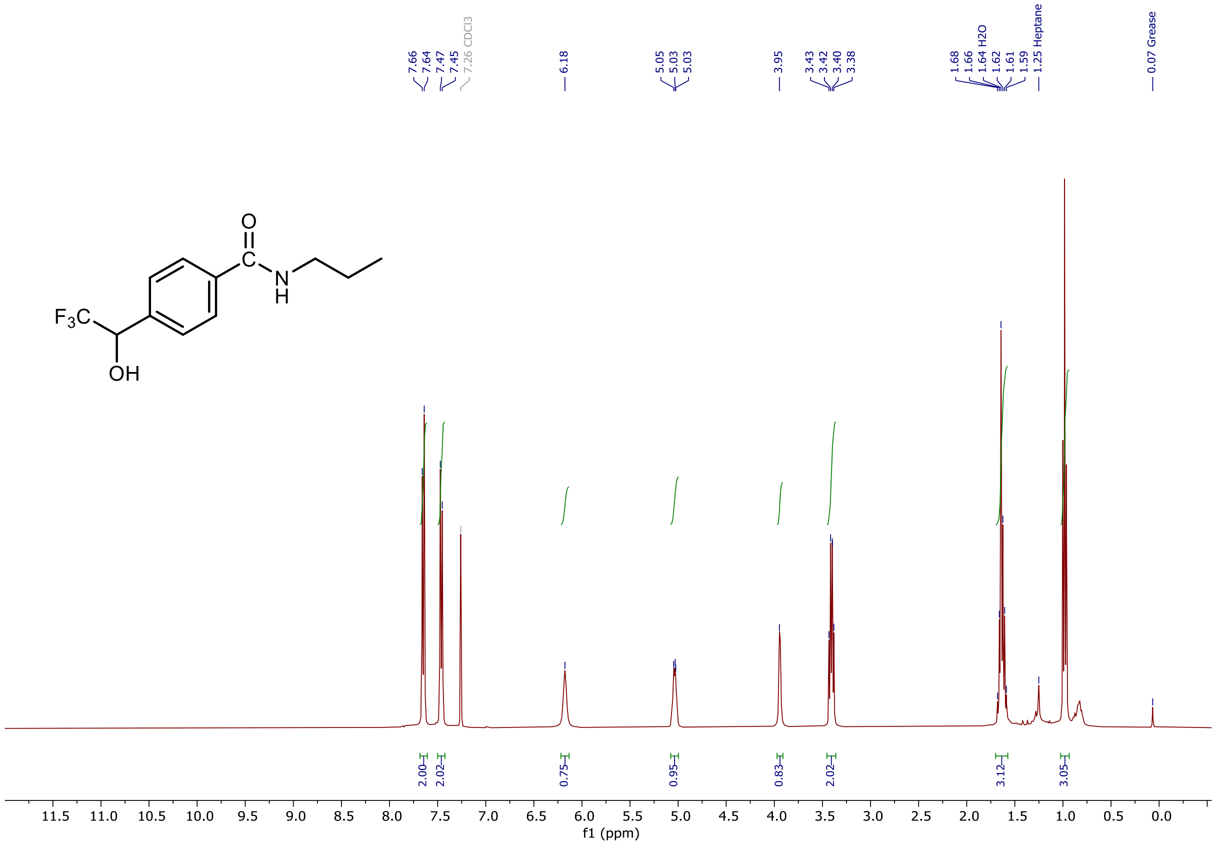

^{13}C-NMR

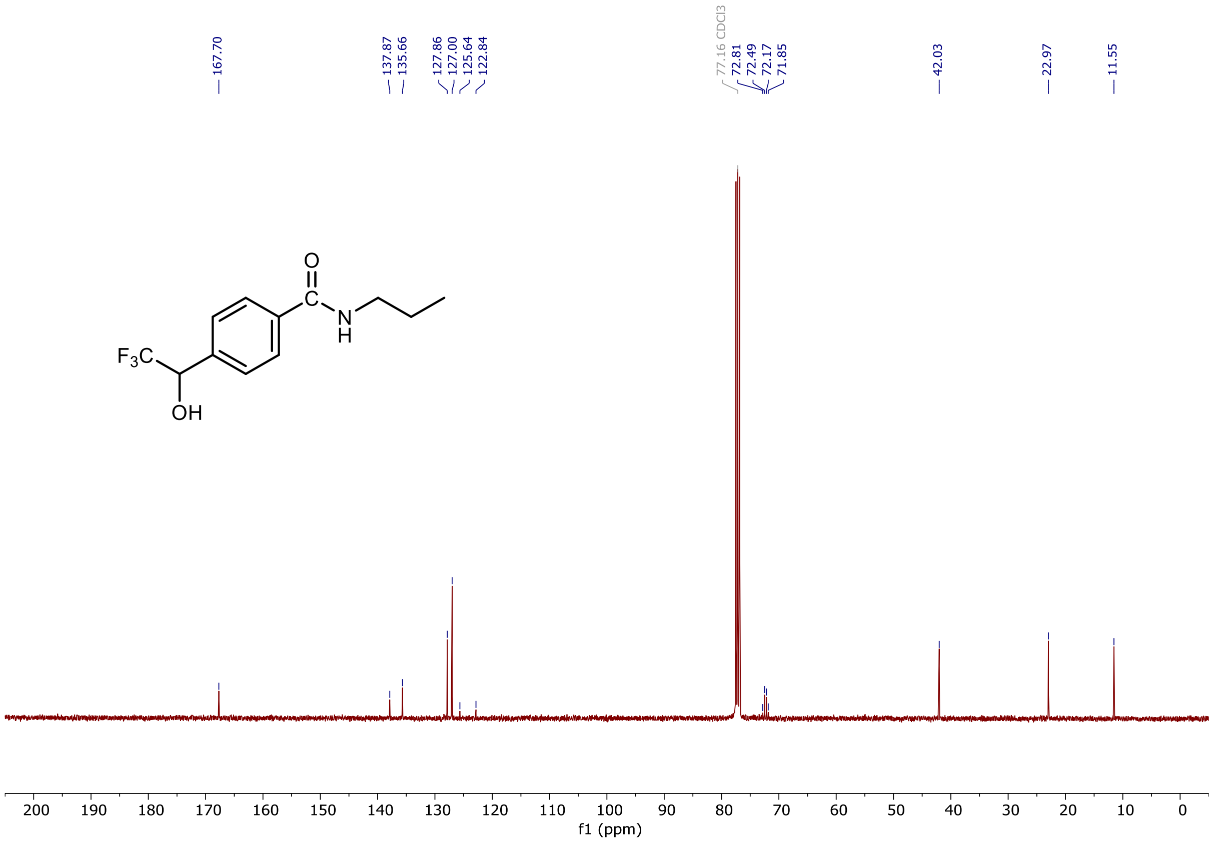

^{19}F-NMR

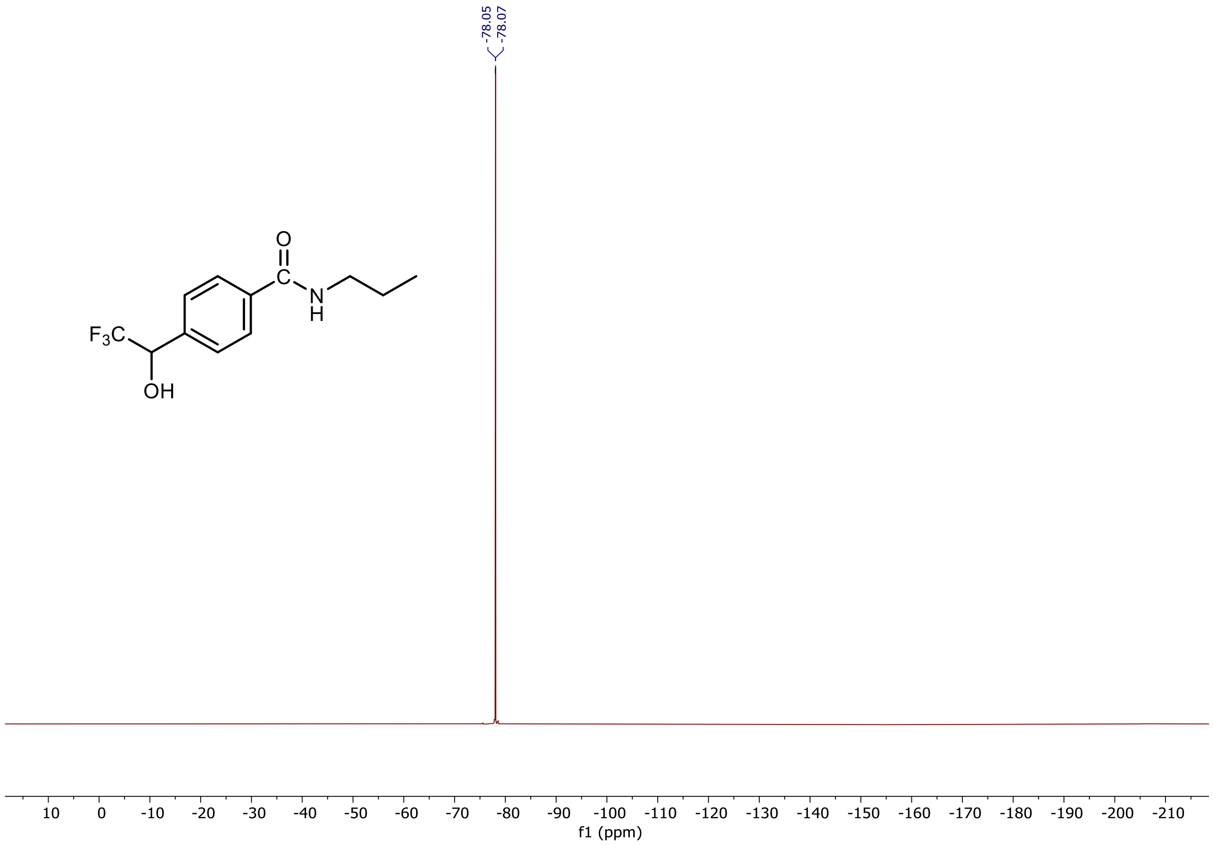
-78.05
-78.07
10
0
-10
-20
-30
-40
-50
-60
-70
-80
-90
-100
-110
-120
-130
-140
-150
-160
-170
-180
-190
-200
-210
f1 (ppm)

N-Propyl-4-(2,2,2-trifluoro-1-hydroxyethyl)benz-^{13}C-amide (^{13}C-1h)

^{1}H-NMR

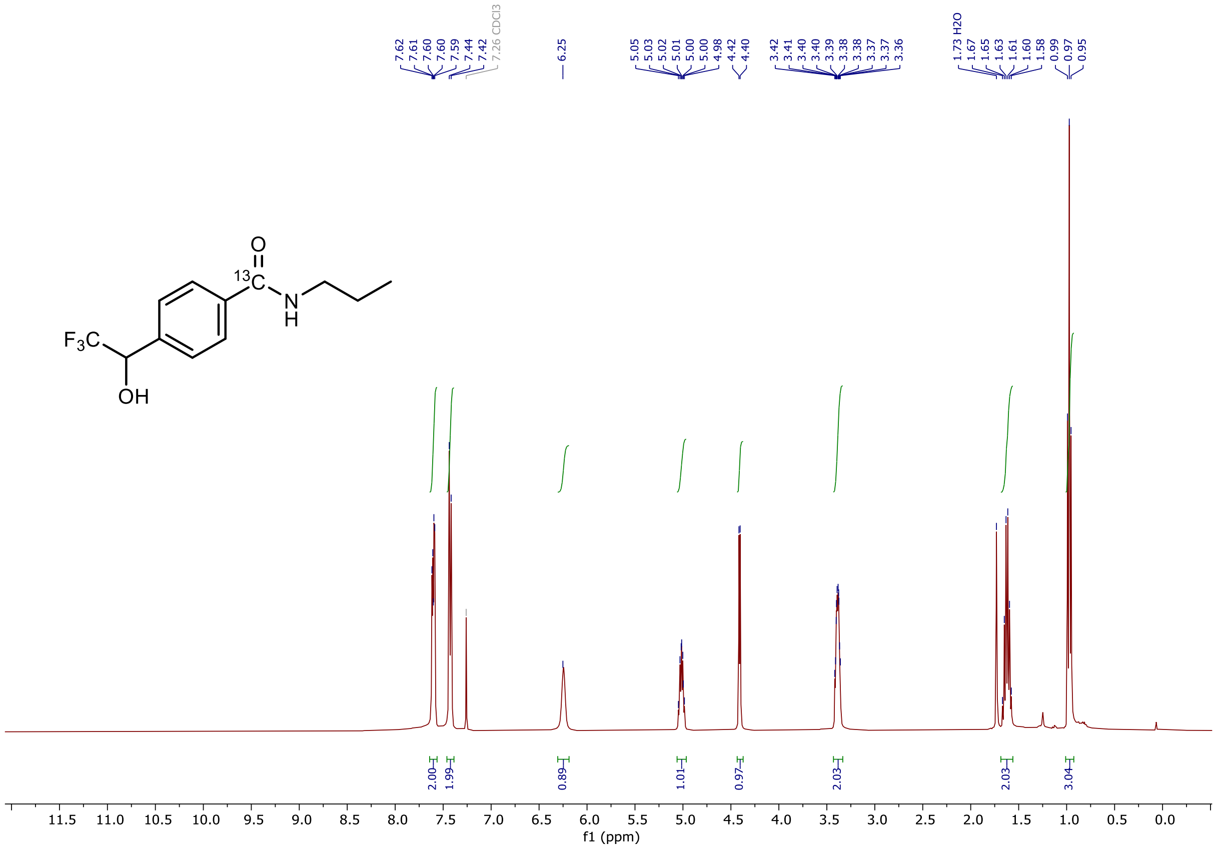

^{13}C-NMR

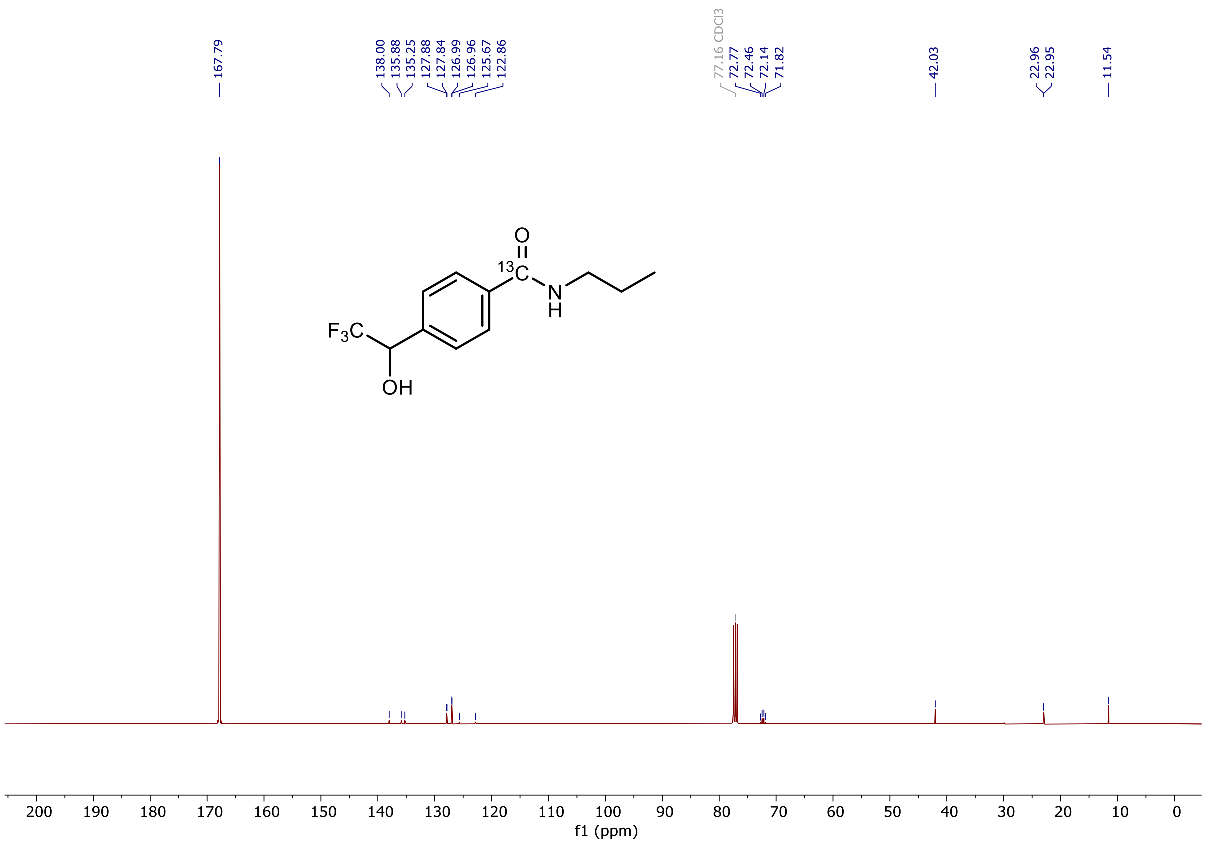

^{19}F-NMR

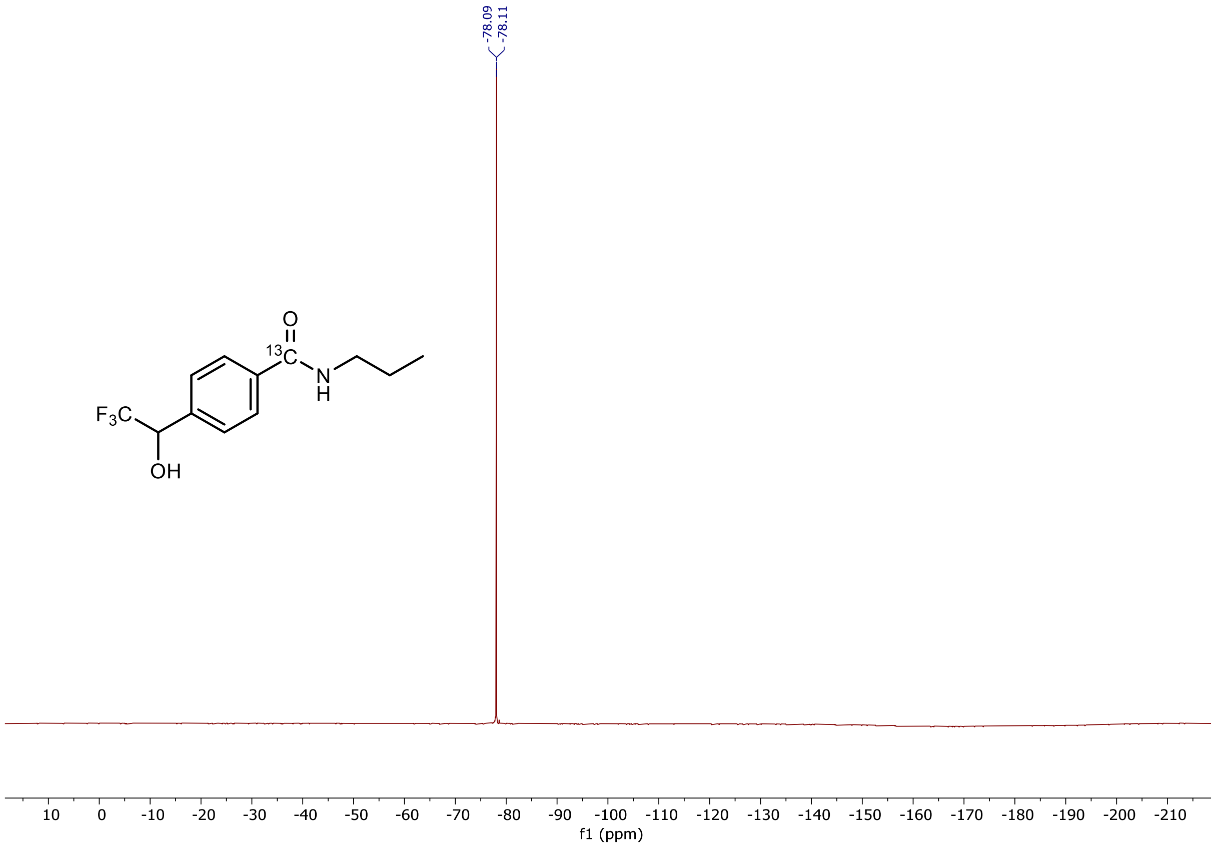

-78.09
-78.11
O
13C
N
H
F3C
OH
10 0 -10 -20 -30 -40 -50 -60 -70 -80 -90 -100 -110 -120 -130 -140 -150 -160 -170 -180 -190 -200 -210
f1 (ppm)

tert-Butyl 2-(propylcarbamoyl)-10H-phenothiazine-10-carboxylate (1i)

^{1}H-NMR

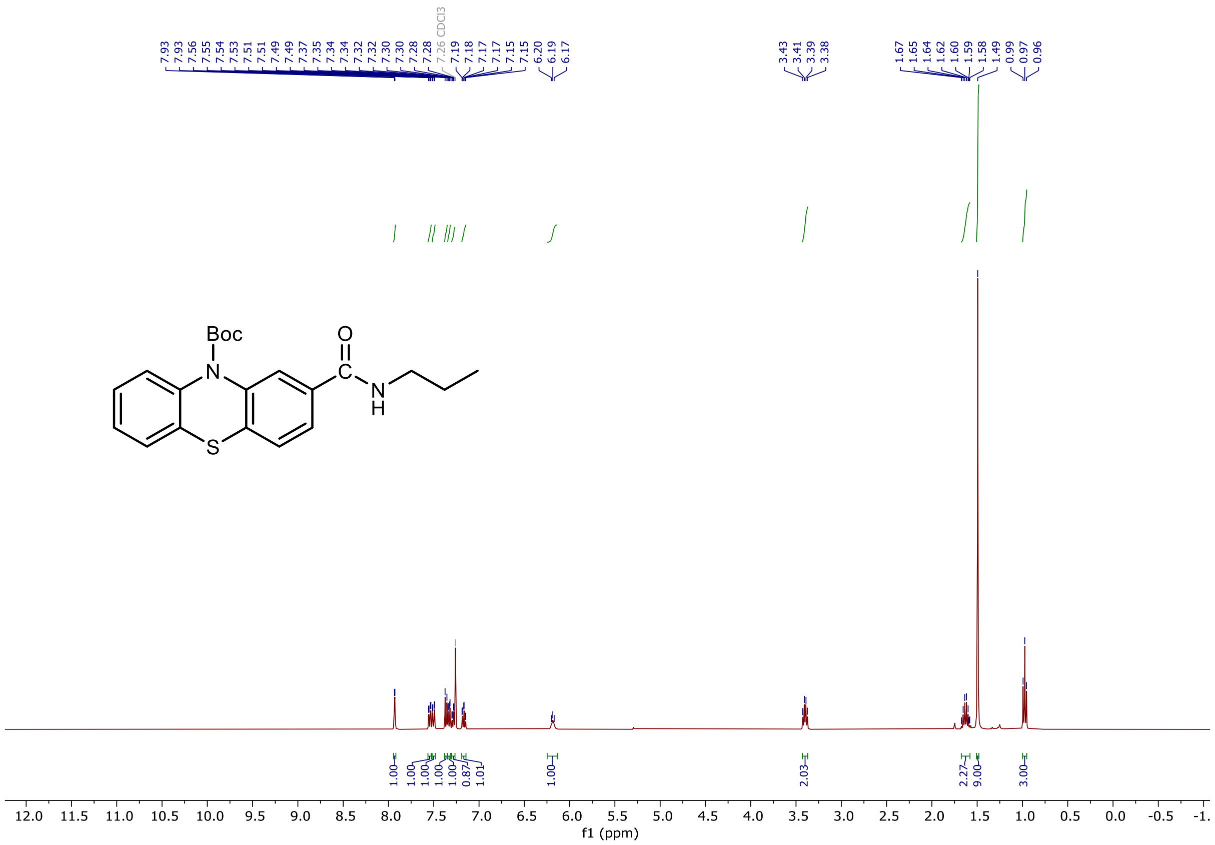

^{13}C-NMR

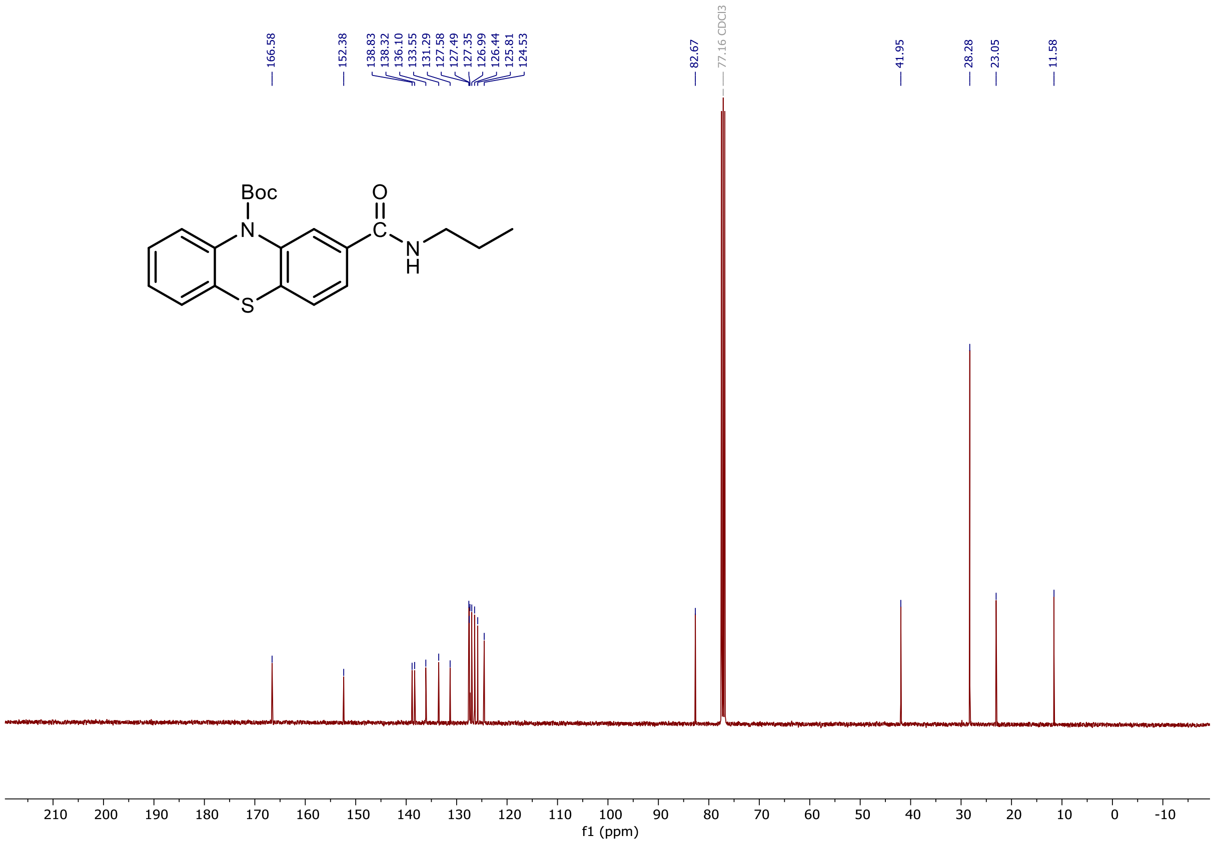

tert-Butyl 2-(propyl-^{13}C-carbamoyl)-10*H*-phenothiazine-10-carboxylate (^{13}C-1i)

^{1}H-NMR

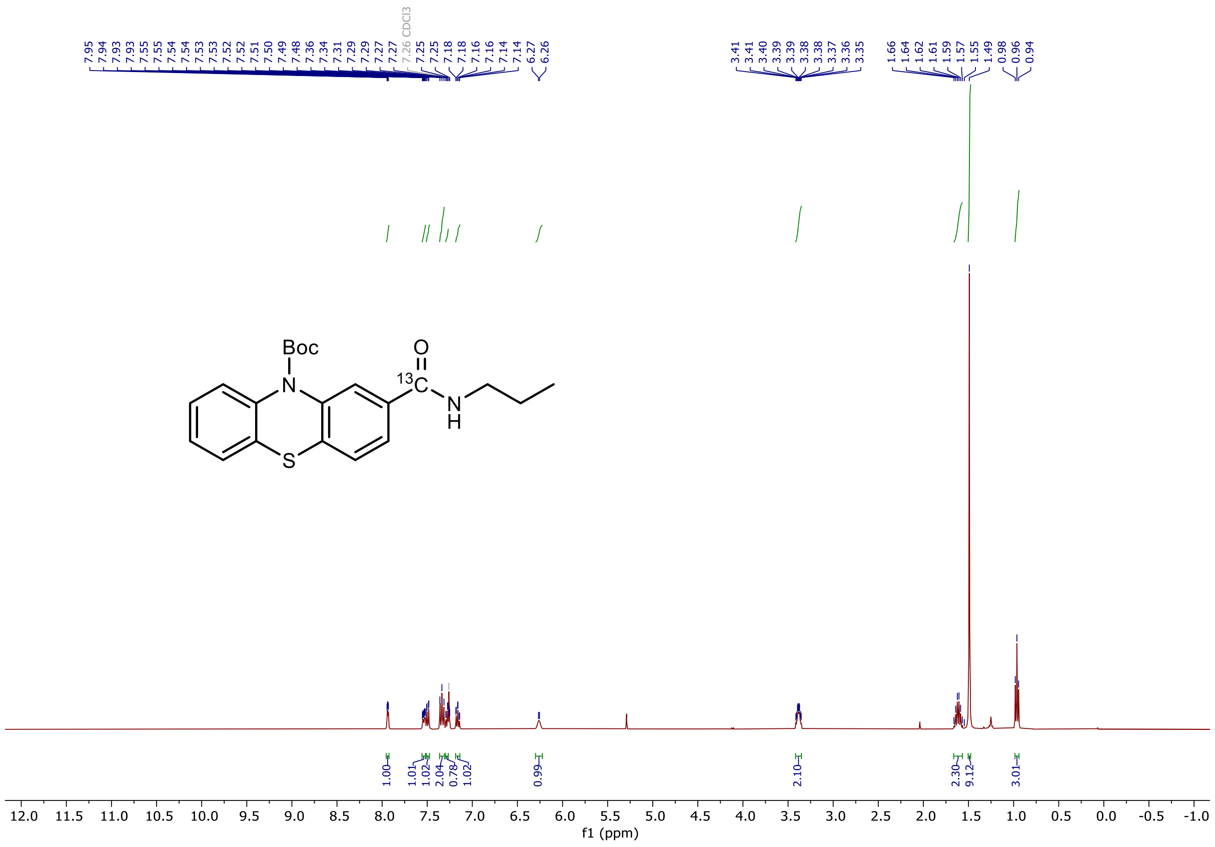

^{13}C-NMR

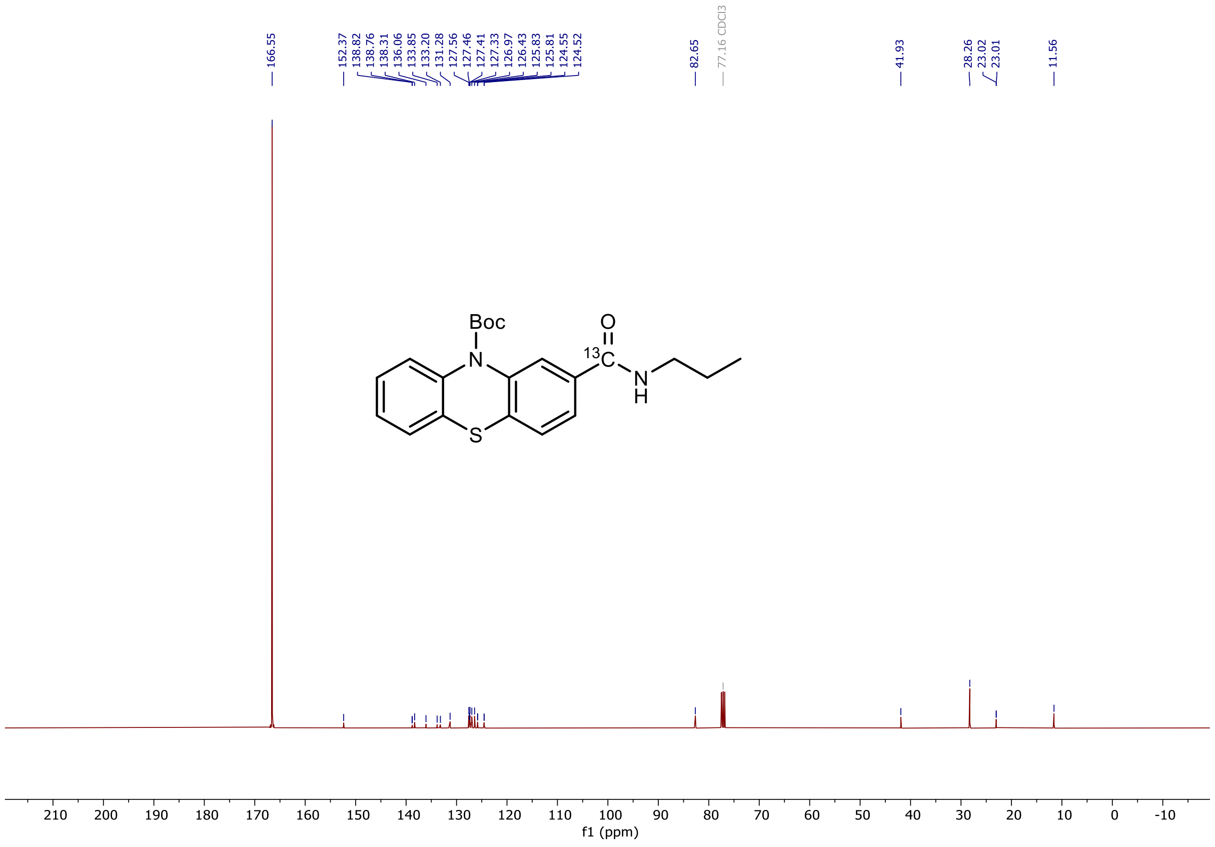

Methyl 2-(((*tert*-butyldimethylsilyl)oxy)(4-(propylcarbamoyl)phenyl)methyl)acrylate (1j)

^{1}H-NMR

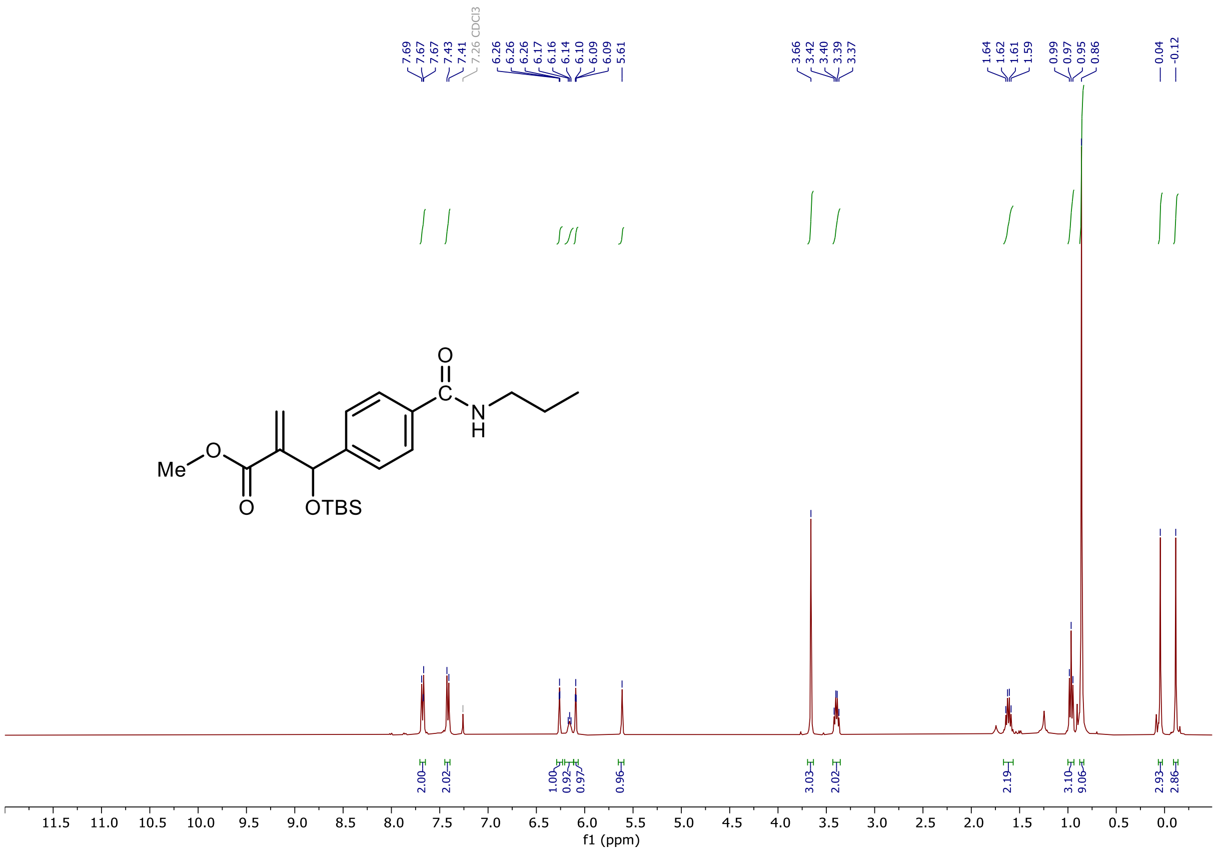

^{13}C-NMR

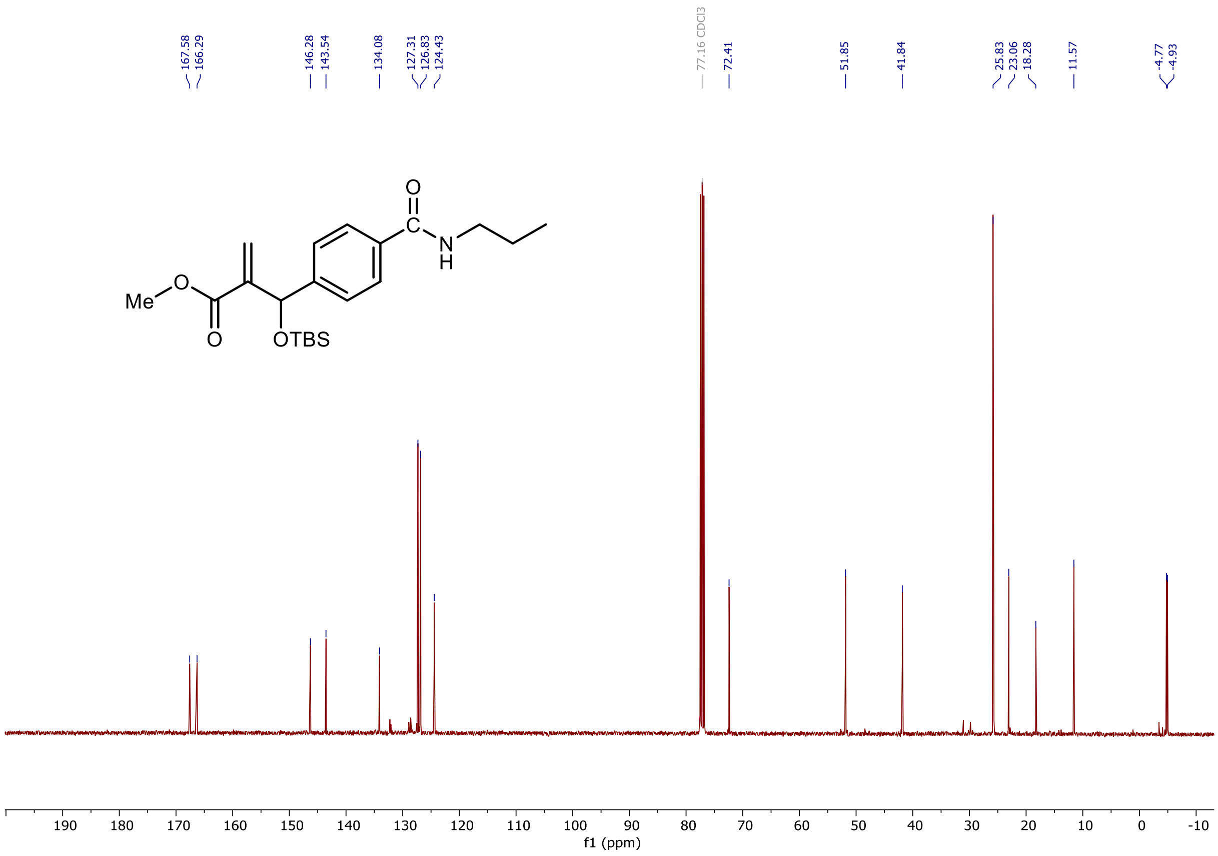

Methyl 2-(((*tert*-butyldimethylsilyl)oxy)(4-(propyl-^{13}C-carbamoyl)phenyl)methyl)acrylate (^{13}C-1j)

^{1}H-NMR

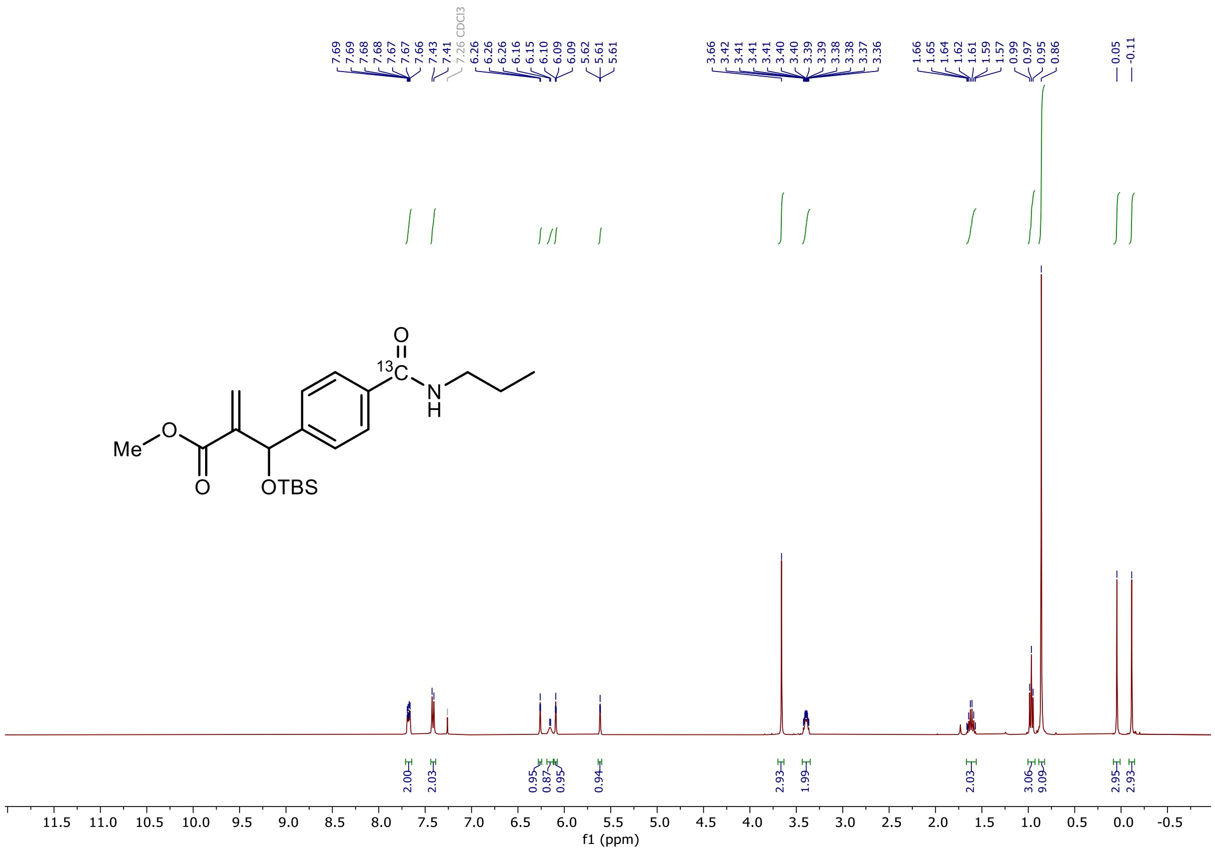

^{13}C-NMR

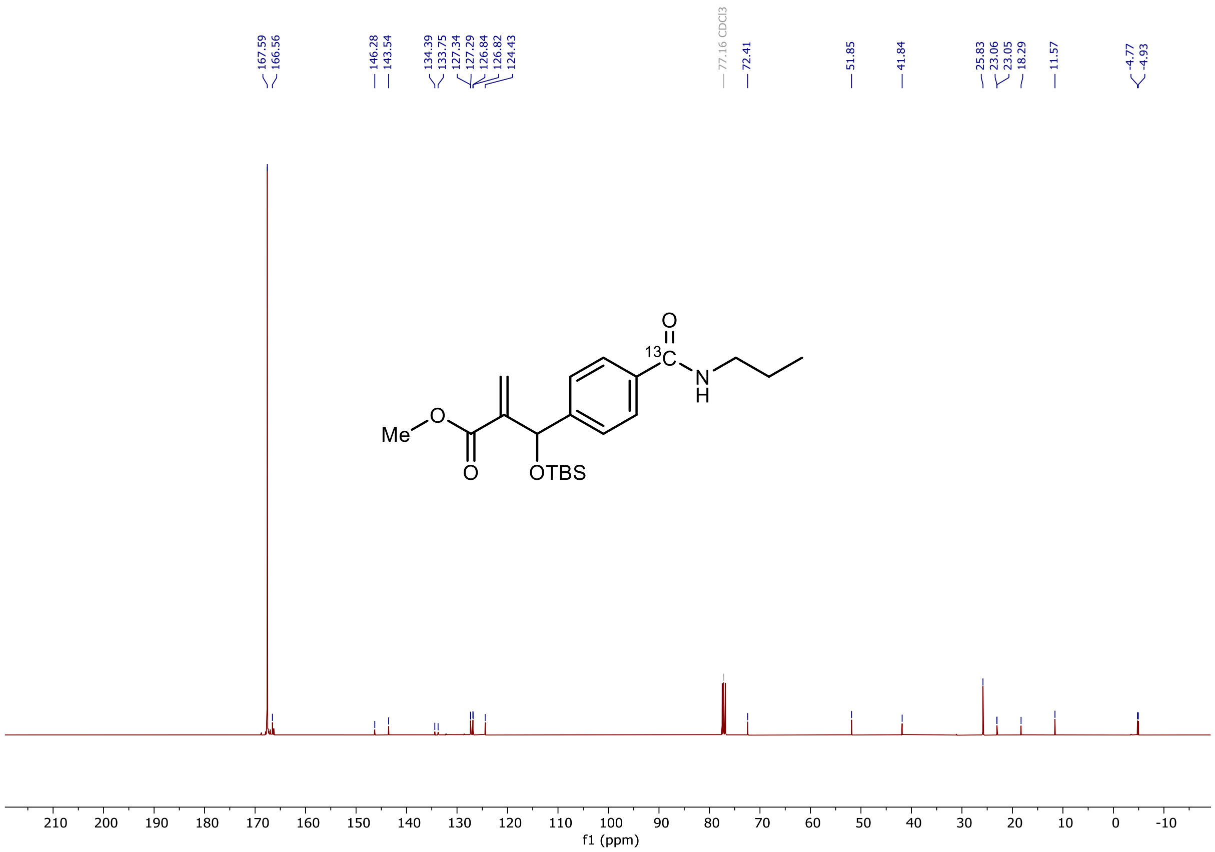

4-(3,3-Dimethylbutanamido)-3,5-difluoro-*N*-propylbenzamide (1k)

^{1}H-NMR

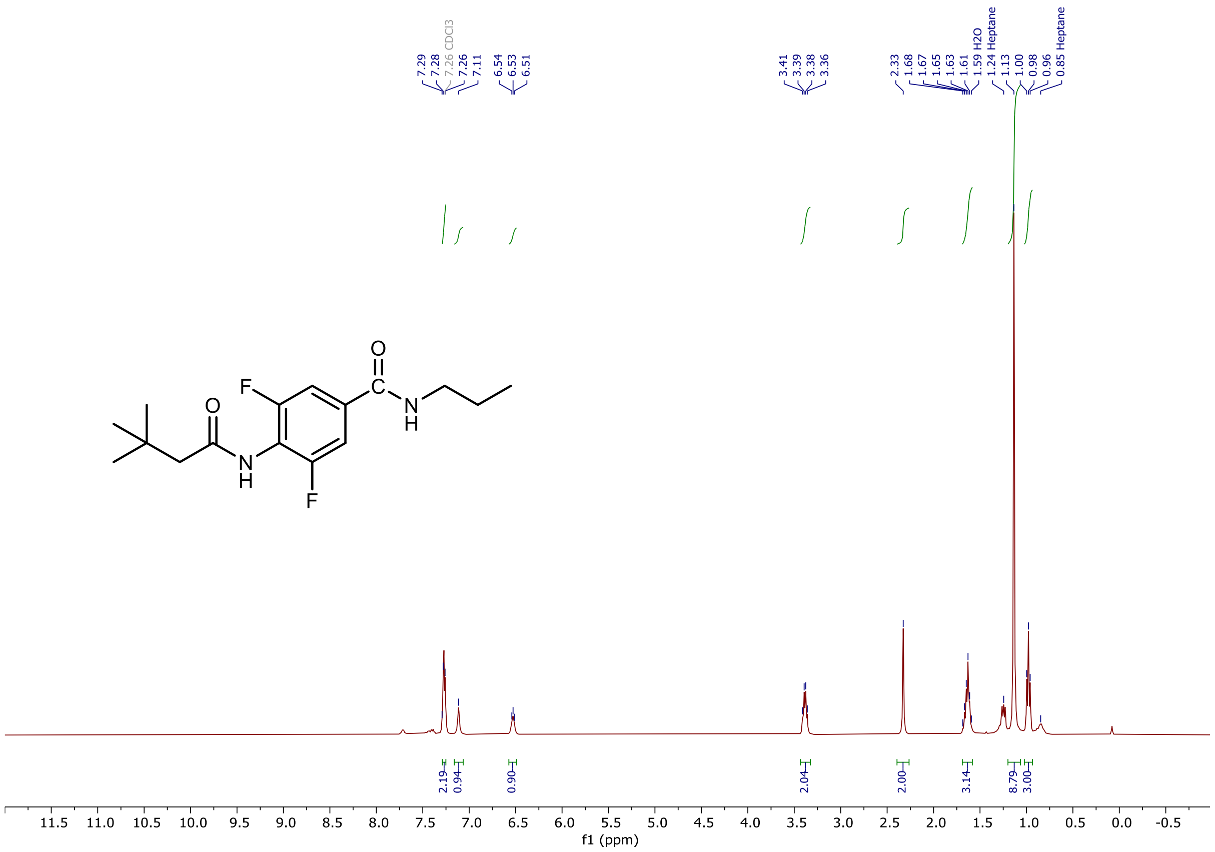

^{13}C-NMR

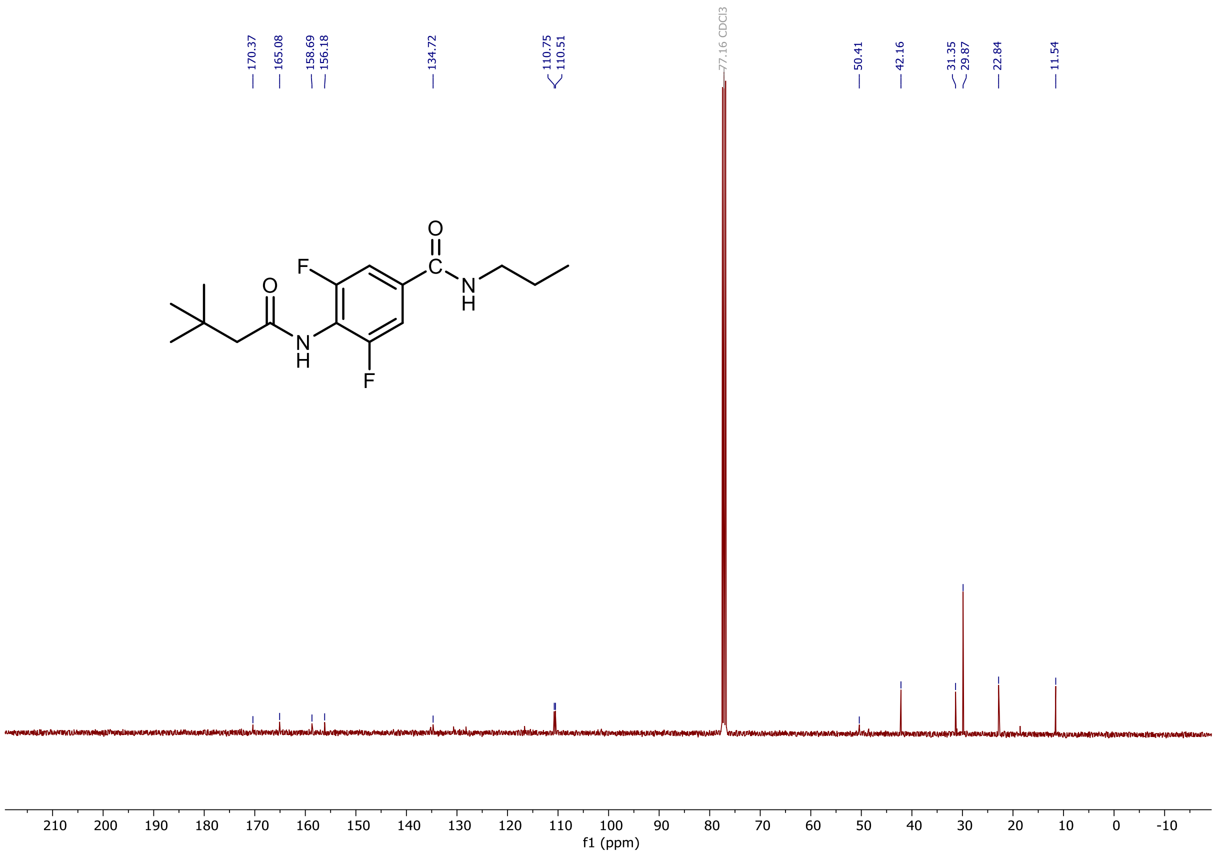

^{19}F-NMR

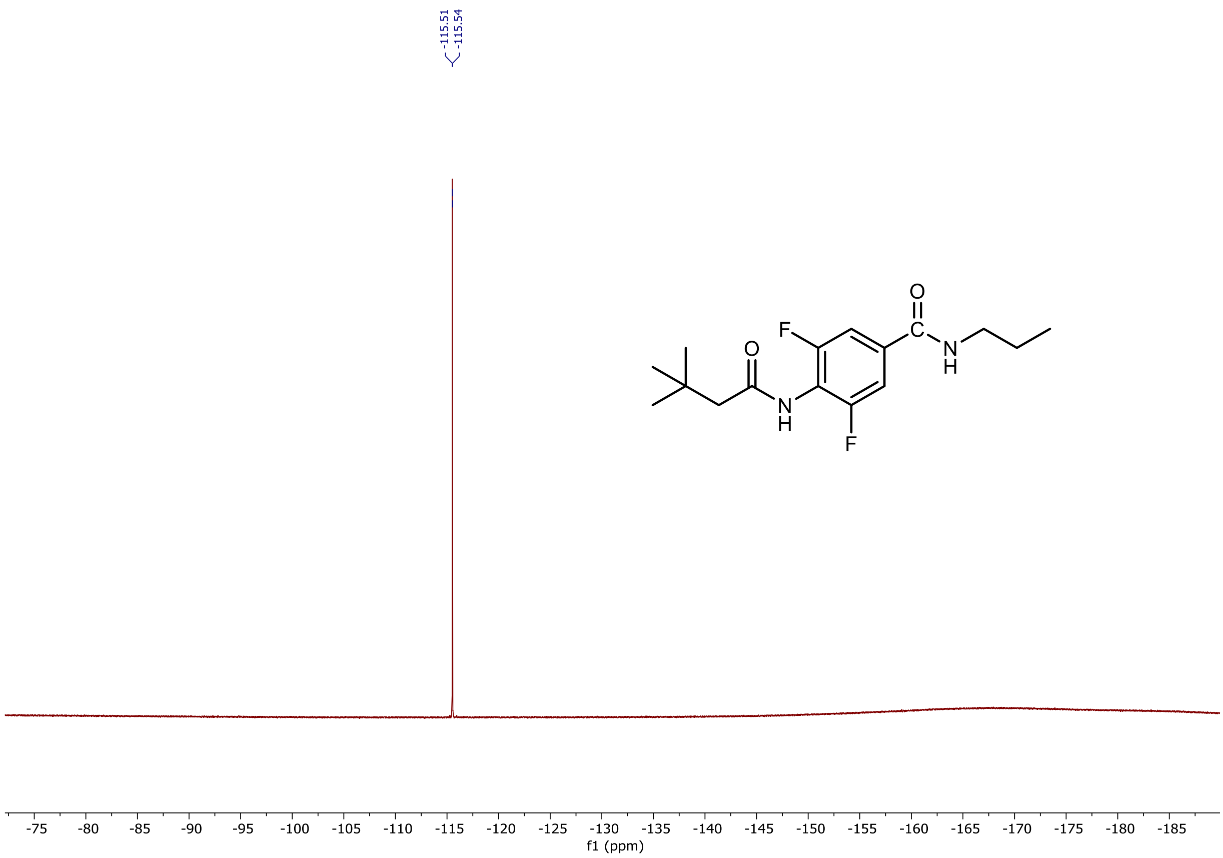

-115.51
-115.54
F
F
O
O
C
N
H
N
H
-75 -80 -85 -90 -95 -100 -105 -110 -115 -120 -125 -130 -135 -140 -145 -150 -155 -160 -165 -170 -175 -180 -185
f1 (ppm)

4-(3,3-Dimethylbutanamido)-3,5-difluoro-*N*-propylbenz-^{13}C-amide (^{13}C-1k)

^{1}H-NMR

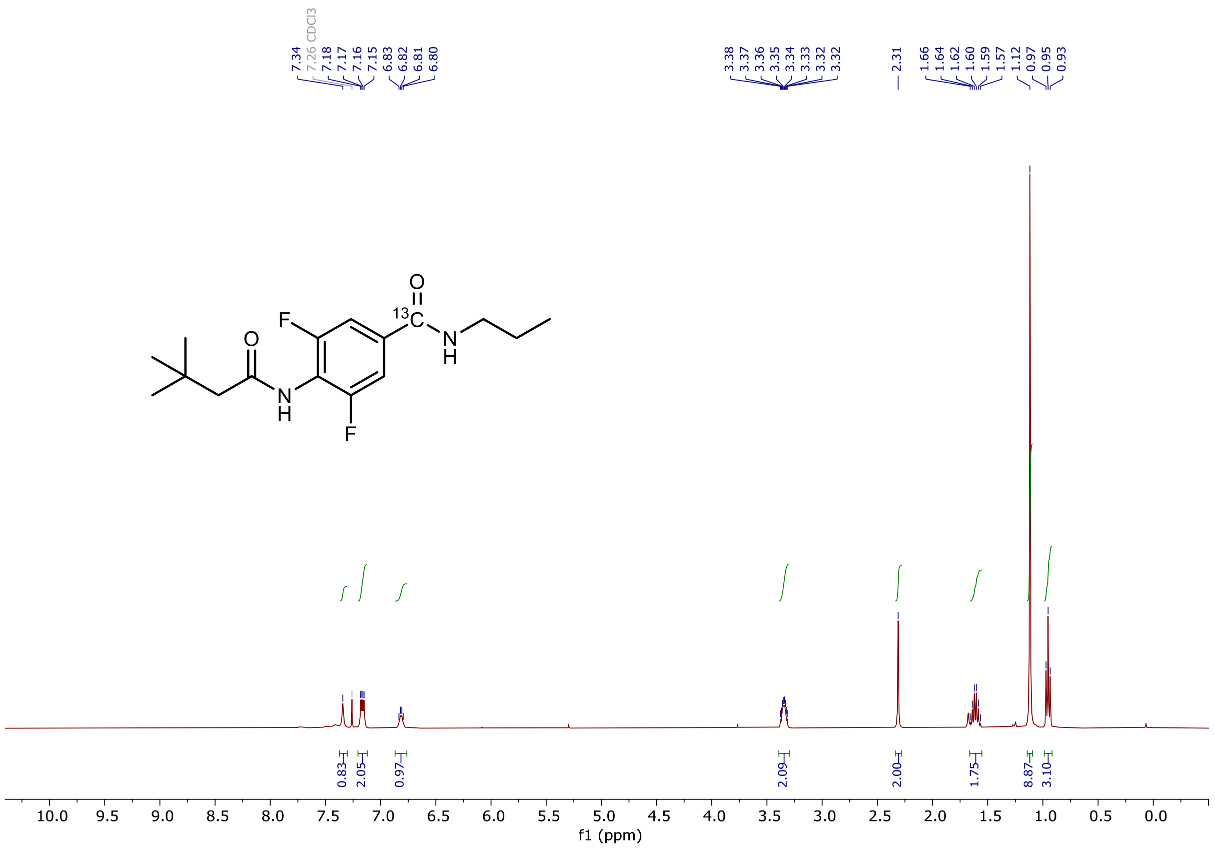

^{13}C-NMR

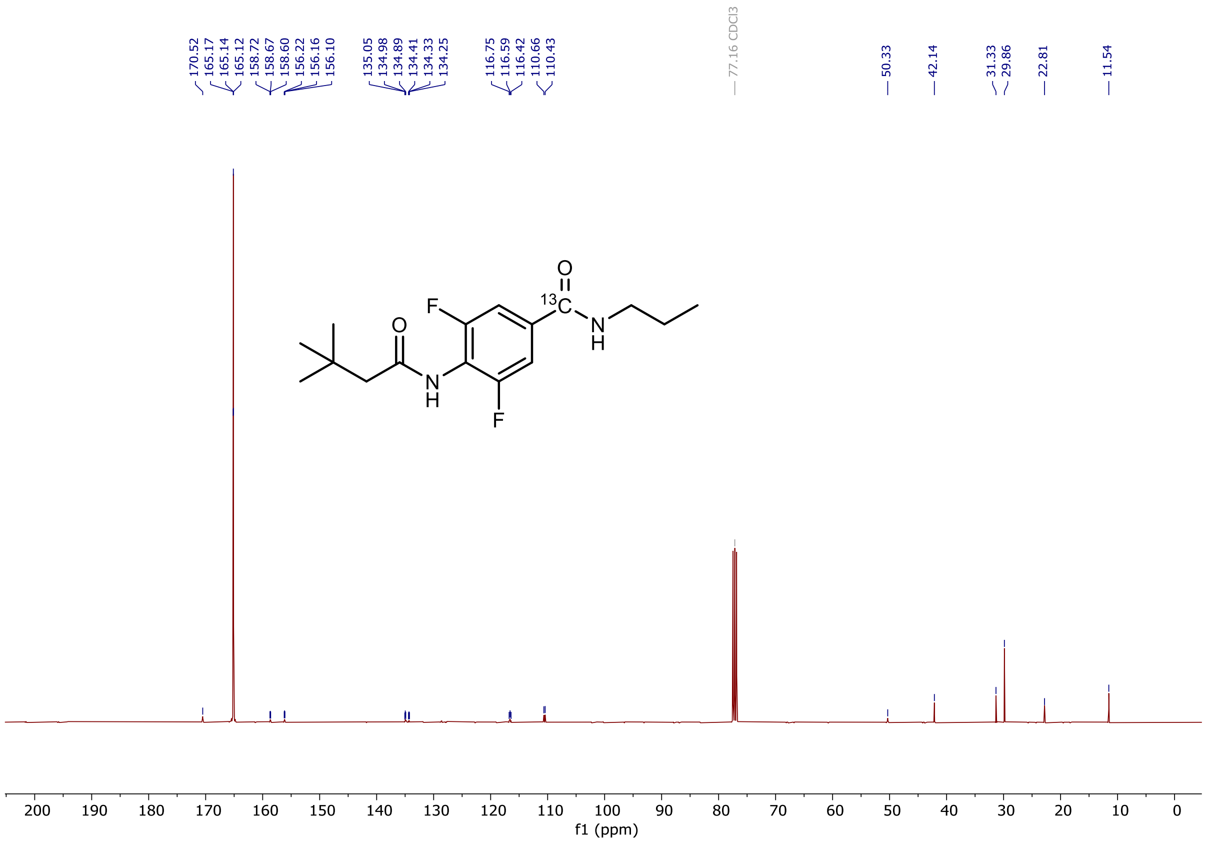

^{19}F-NMR

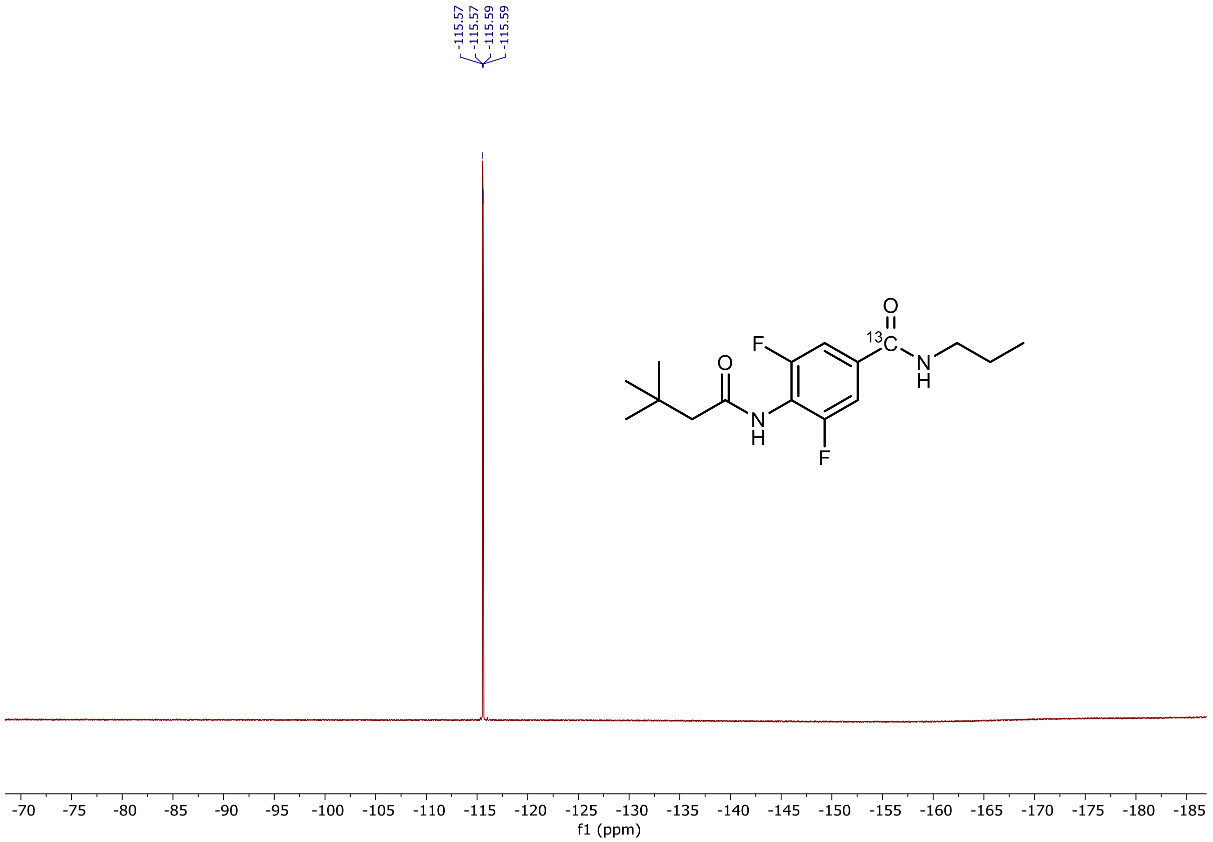

-115.57
-115.57
-115.59
-115.59
-70 -75 -80 -85 -90 -95 -100 -105 -110 -115 -120 -125 -130 -135 -140 -145 -150 -155 -160 -165 -170 -175 -180 -185
f1 (ppm)
F
O
13C
N
H
O
N
H
F

N-Propyldibenzo[*b,d*]furan-4-carboxamide (1l)

^{1}H-NMR

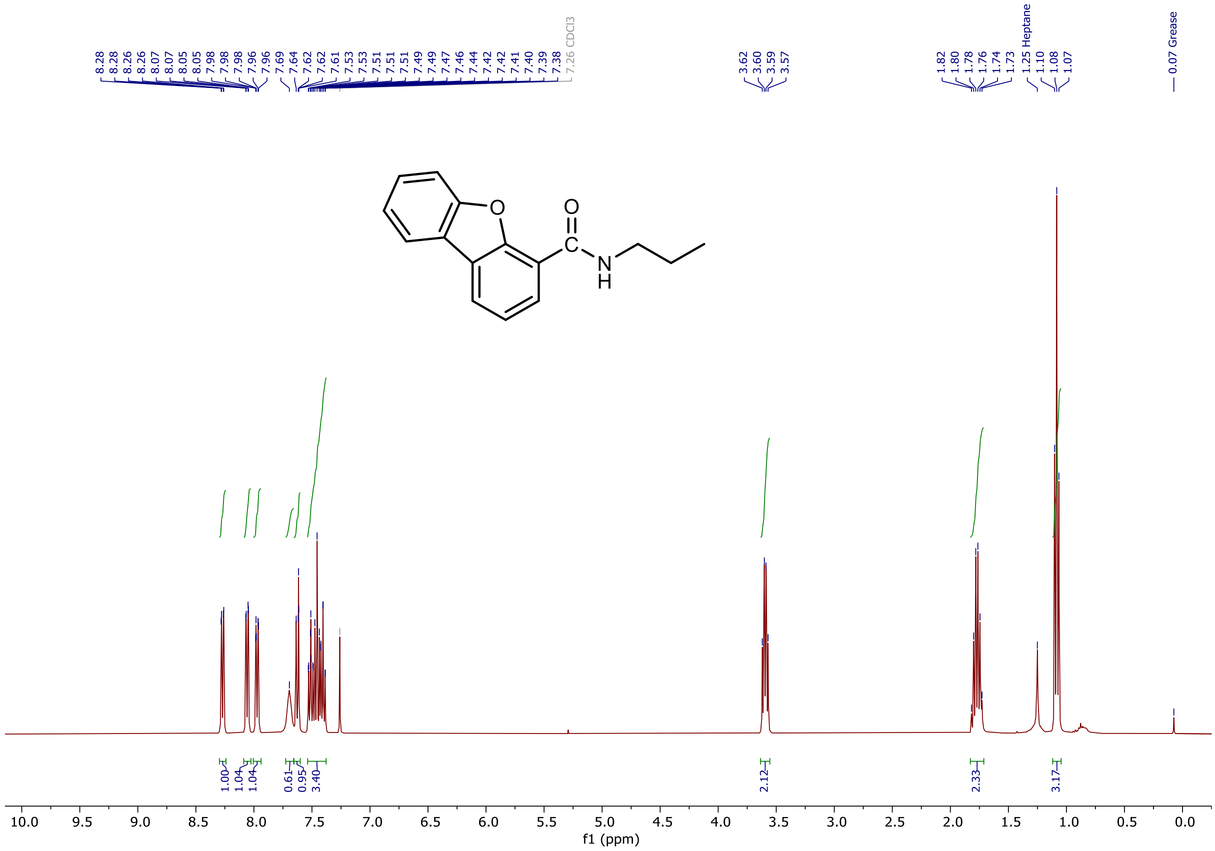

^{13}C-NMR

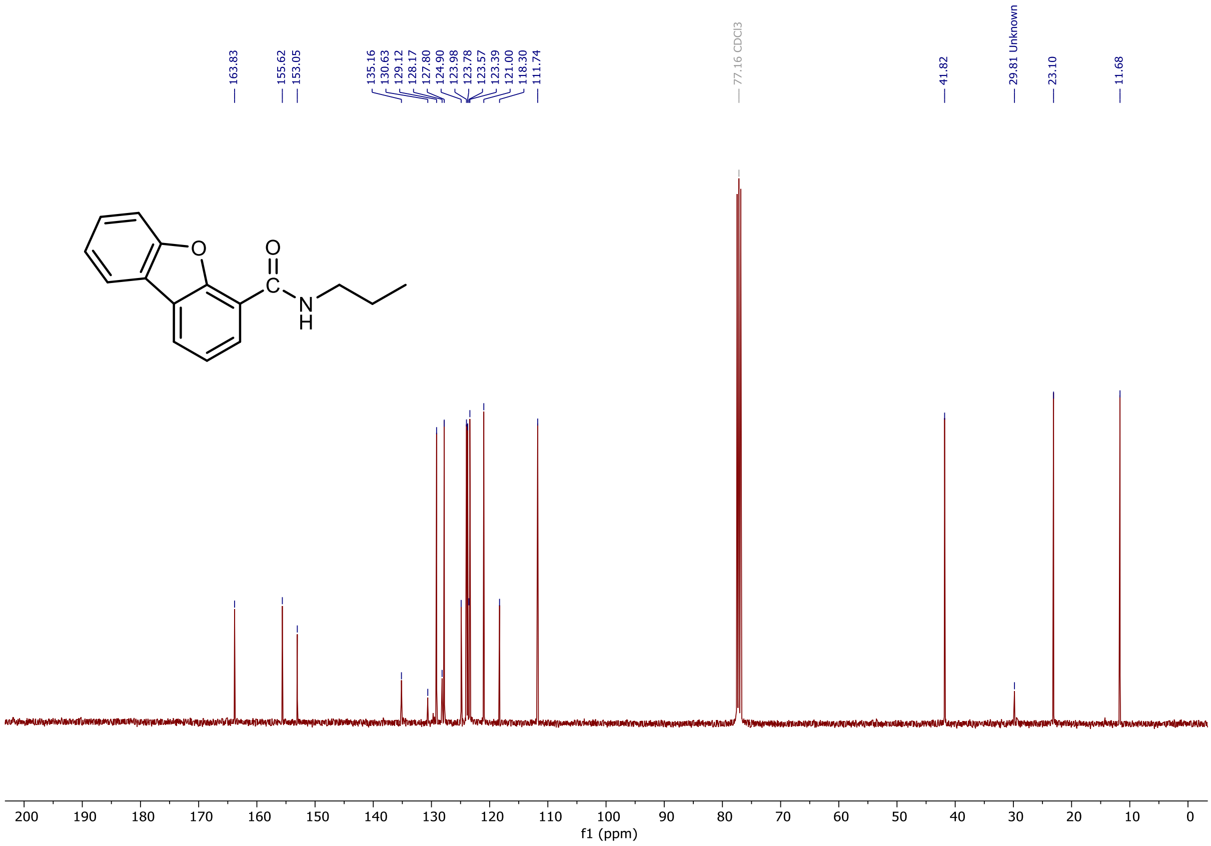

***N*-Propylcinnamamide (1m)**

^{1}H-NMR

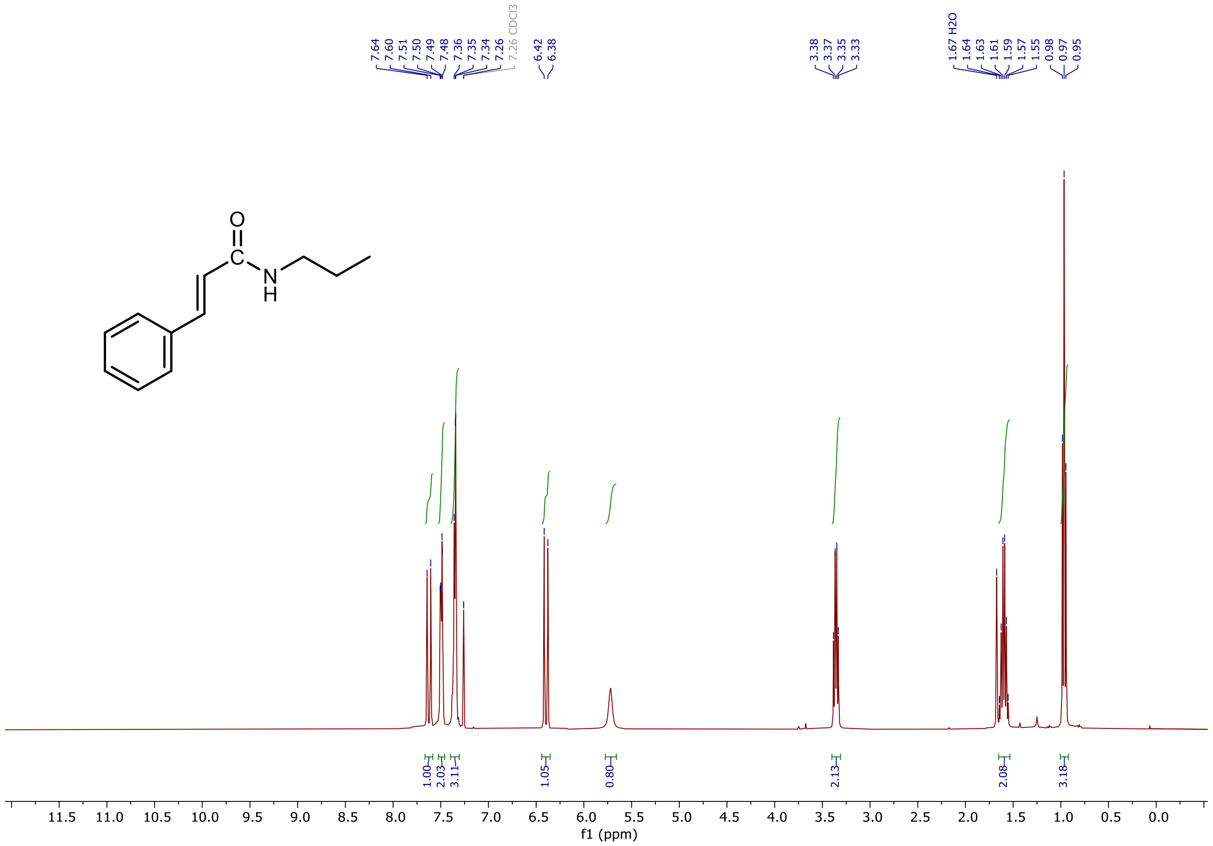

^{13}C-NMR

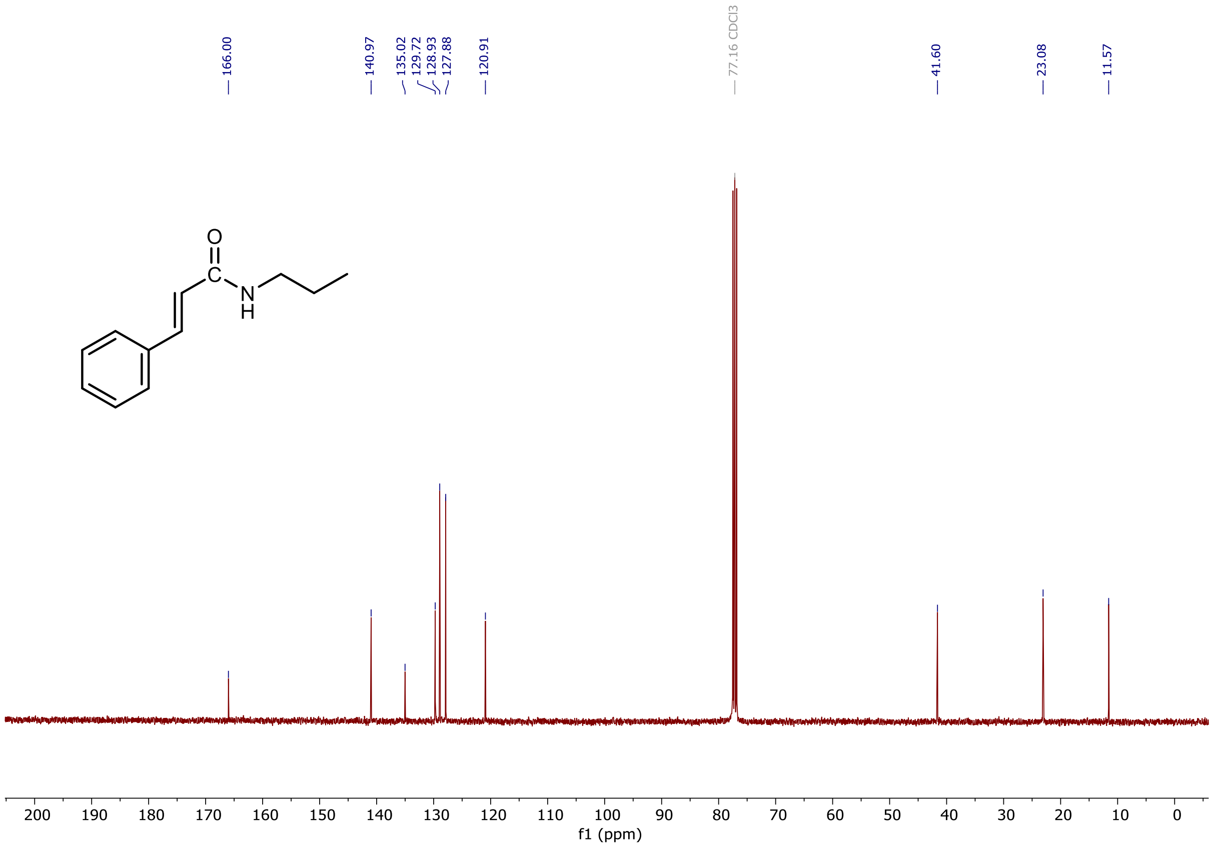

[1,1'-biphenyl]-4-yl(4-(2-chlorodibenzo[*b,f*][1,4]oxazepin-11-yl)piperazin-1-yl)-methanone (^{12}C-10a)

^{1}H-NMR

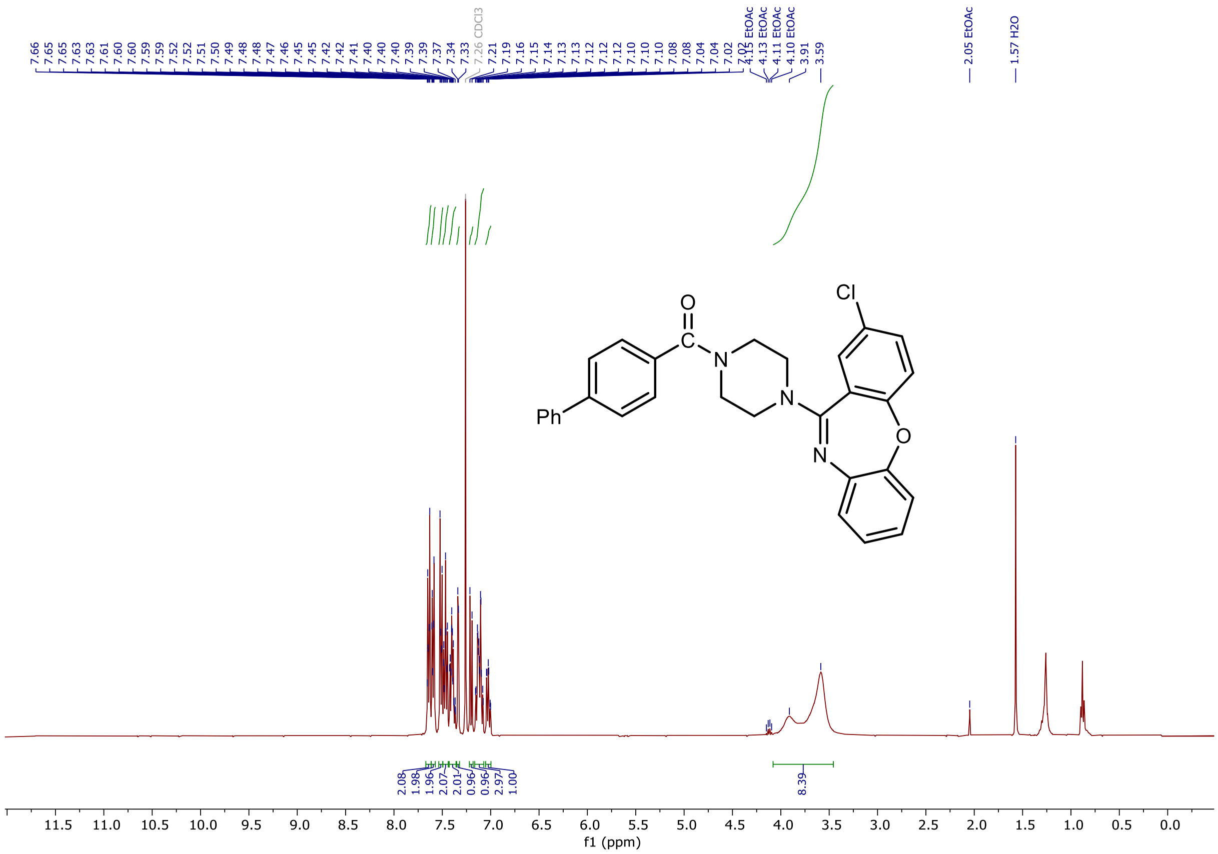

^{13}C-NMR

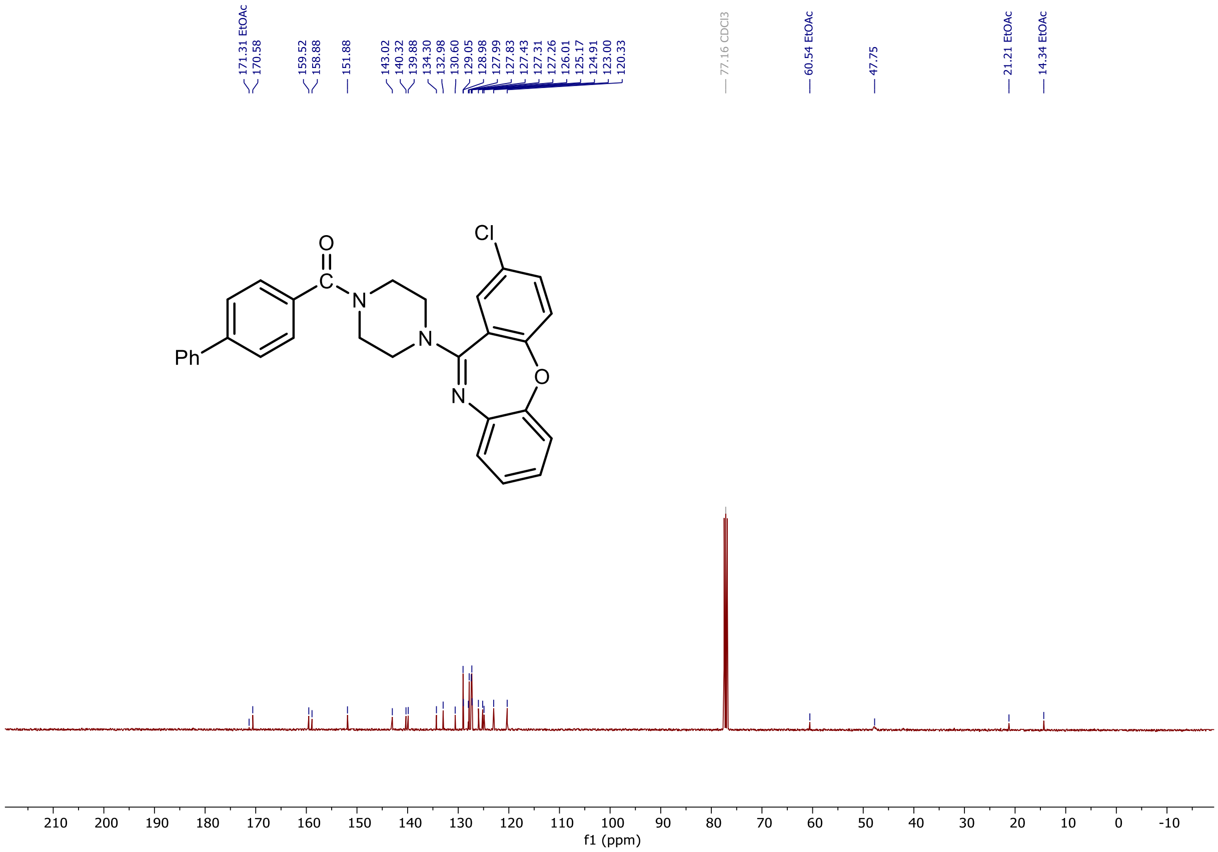

[1,1'-biphenyl]-4-yl(4-(2-chlorodibenzo[*b,f*][1,4]oxazepin-11-yl)piperazin-1-yl)-^{13}C-methanone (^{13}C-10a)

^{1}H-NMR

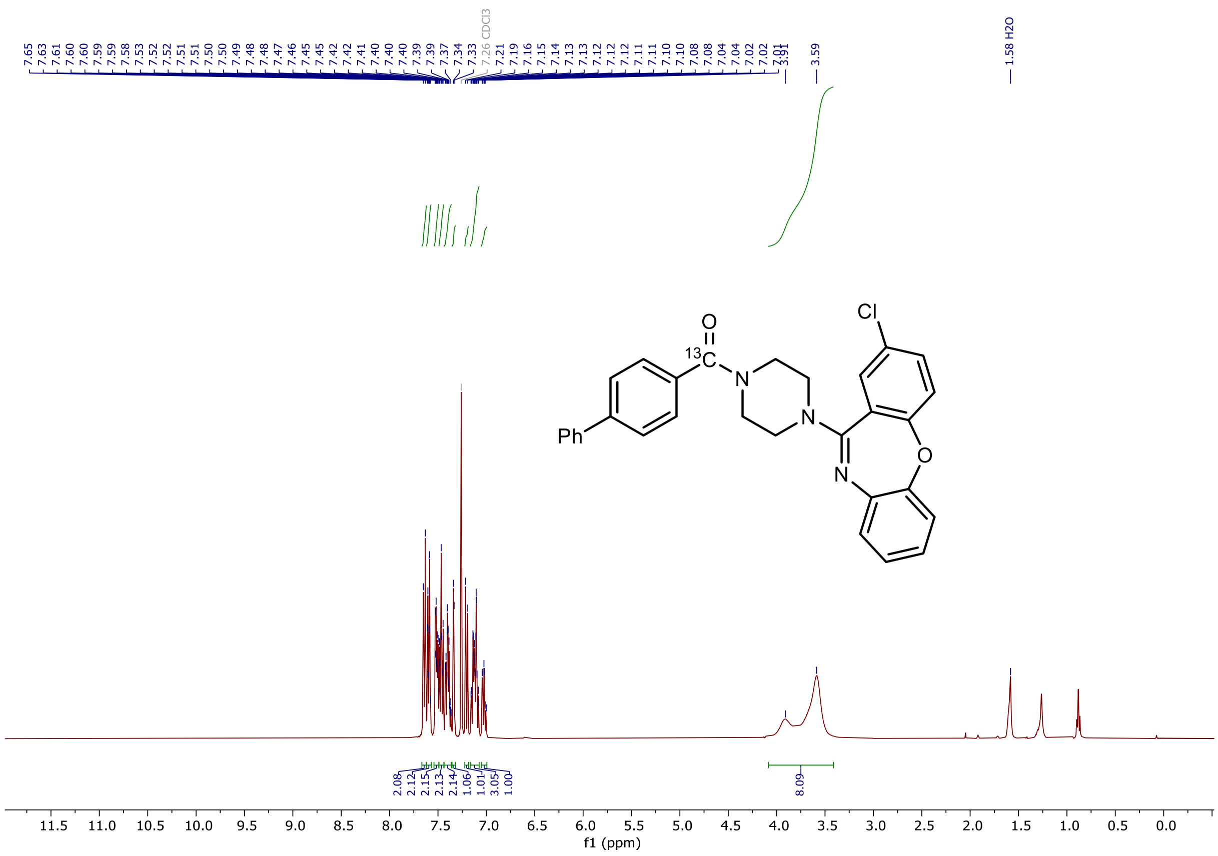

^{13}C-NMR

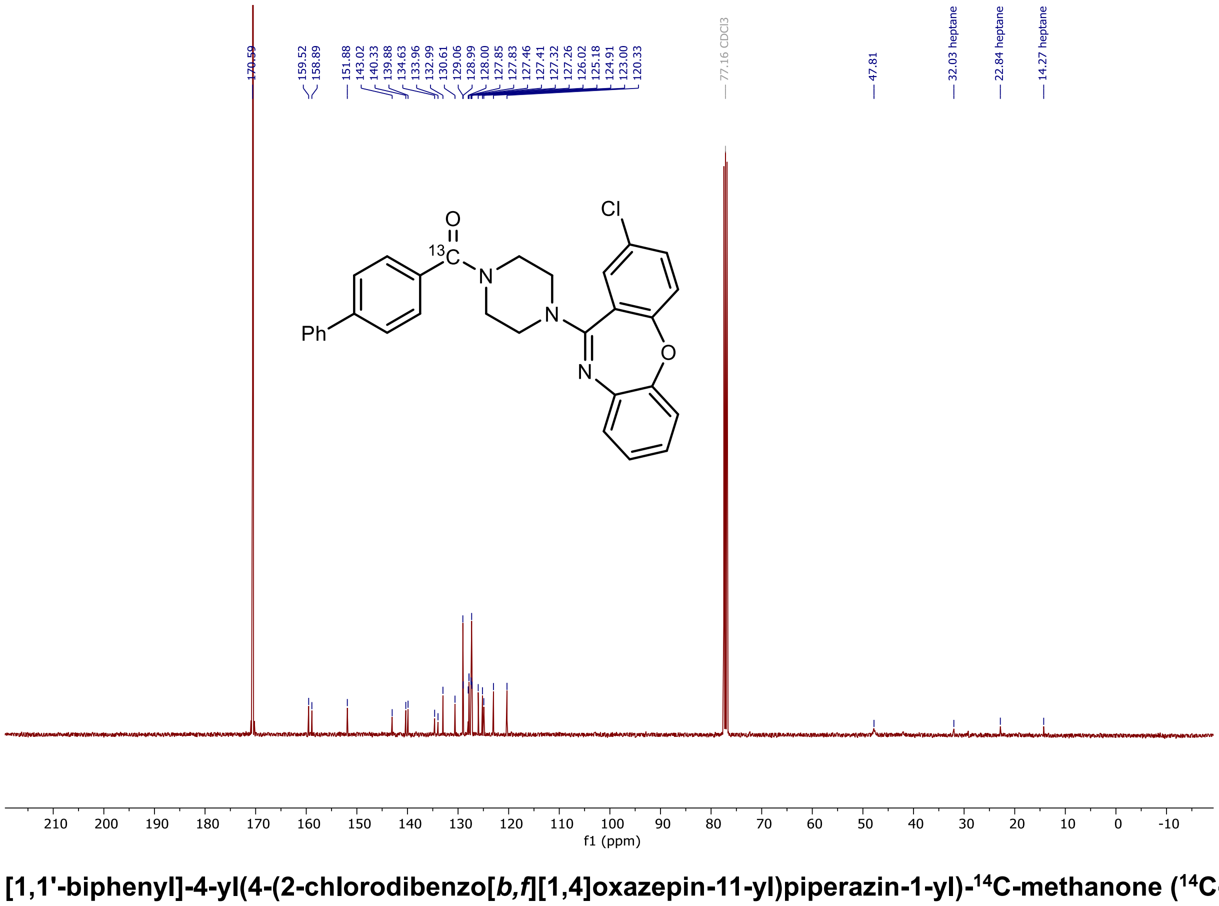

[1,1'-biphenyl]-4-yl(4-(2-chlorodibenzo[*b,f*][1,4]oxazepin-11-yl)piperazin-1-yl)-^{14}C-methanone (^{14}C-10a)

^{1}H-NMR

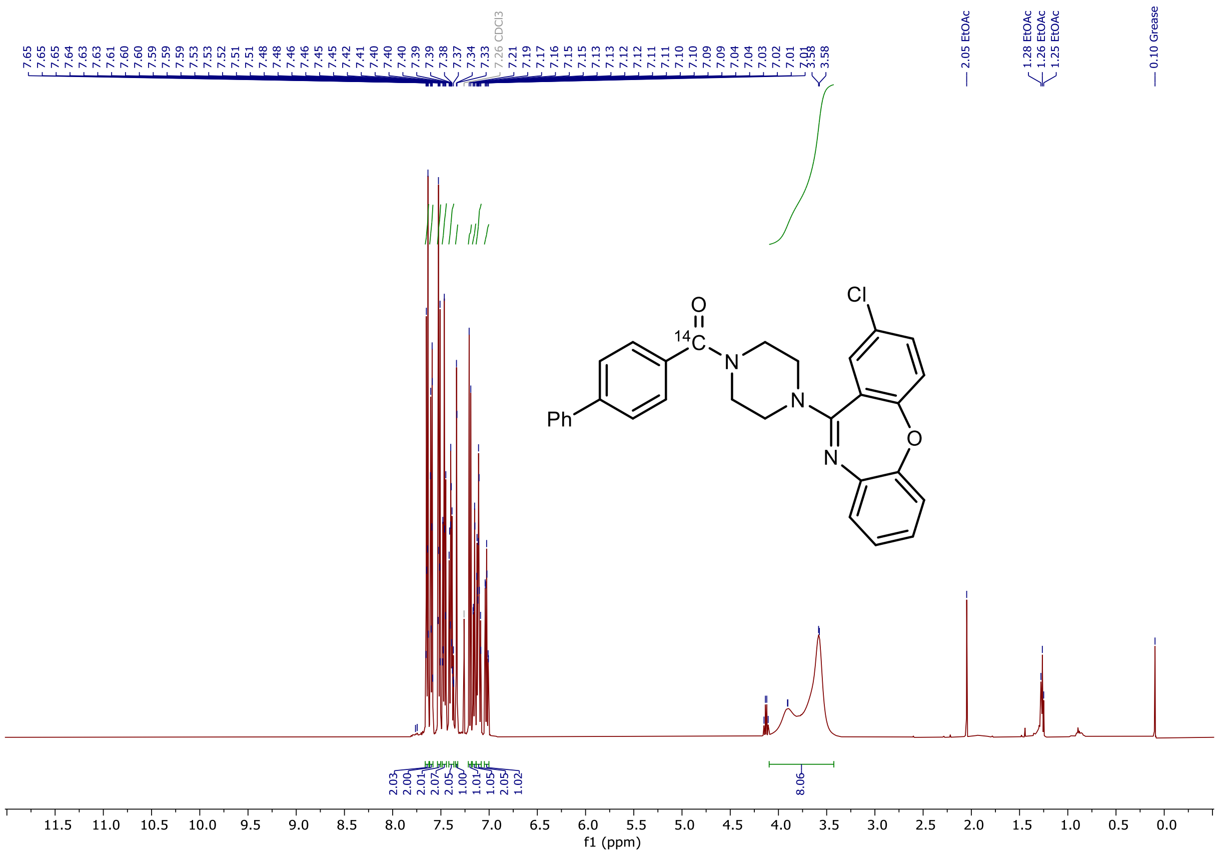
7.65
7.65
7.64
7.63
7.63
7.61
7.60
7.59
7.59
7.59
7.53
7.53
7.52
7.51
7.51
7.48
7.46
7.45
7.45
7.42
7.41
7.40
7.40
7.40
7.39
7.39
7.38
7.37
7.34
7.33
7.26 CDCl3
7.21
7.19
7.17
7.16
7.15
7.15
7.13
7.13
7.12
7.12
7.11
7.11
7.10
7.10
7.09
7.09
7.04
7.04
7.03
7.02
7.01
3.58
3.58
2.05 EtOAc
1.28 EtOAc
1.26 EtOAc
1.25 EtOAc
0.10 Grease
Cl
O
14C
N
N
N
O
Ph
2.03
2.00
2.01
2.07
2.05
1.00
1.01
1.05
2.05
1.02
8.06
f1 (ppm)

^{13}C-NMR

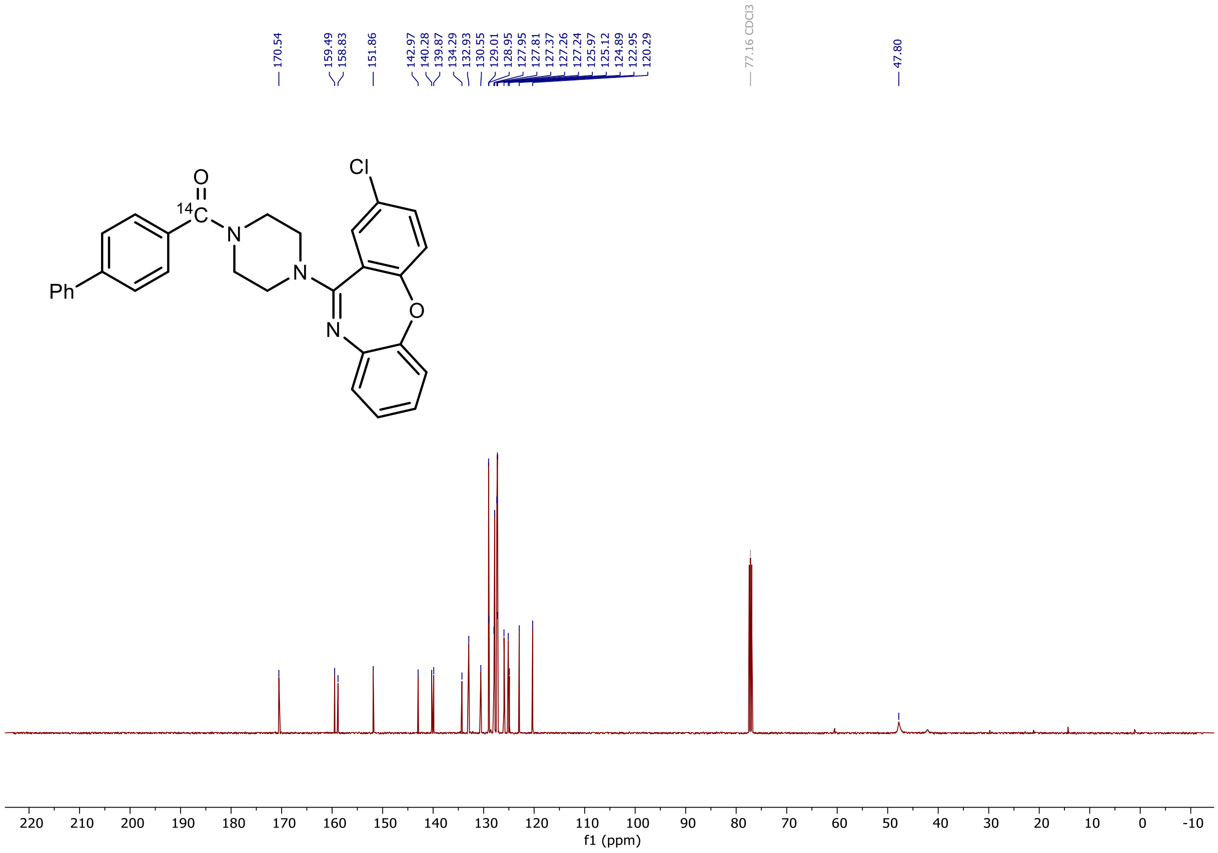
170.54
159.49
158.83
151.86
142.97
140.28
139.87
134.29
132.93
130.55
129.01
128.95
127.95
127.81
127.37
127.26
127.24
125.97
125.12
124.89
122.95
120.29
77.16 CDCl3
47.80
Cl
O
14C
N
N
N
O
Ph
f1 (ppm)

6-(4-(2-chlorodibenzo[*b,f*][1,4]oxazepin-11-yl)piperazine-1-carbonyl)-1-methylindolin-2-one (^{12}C-10p)

^{1}H-NMR

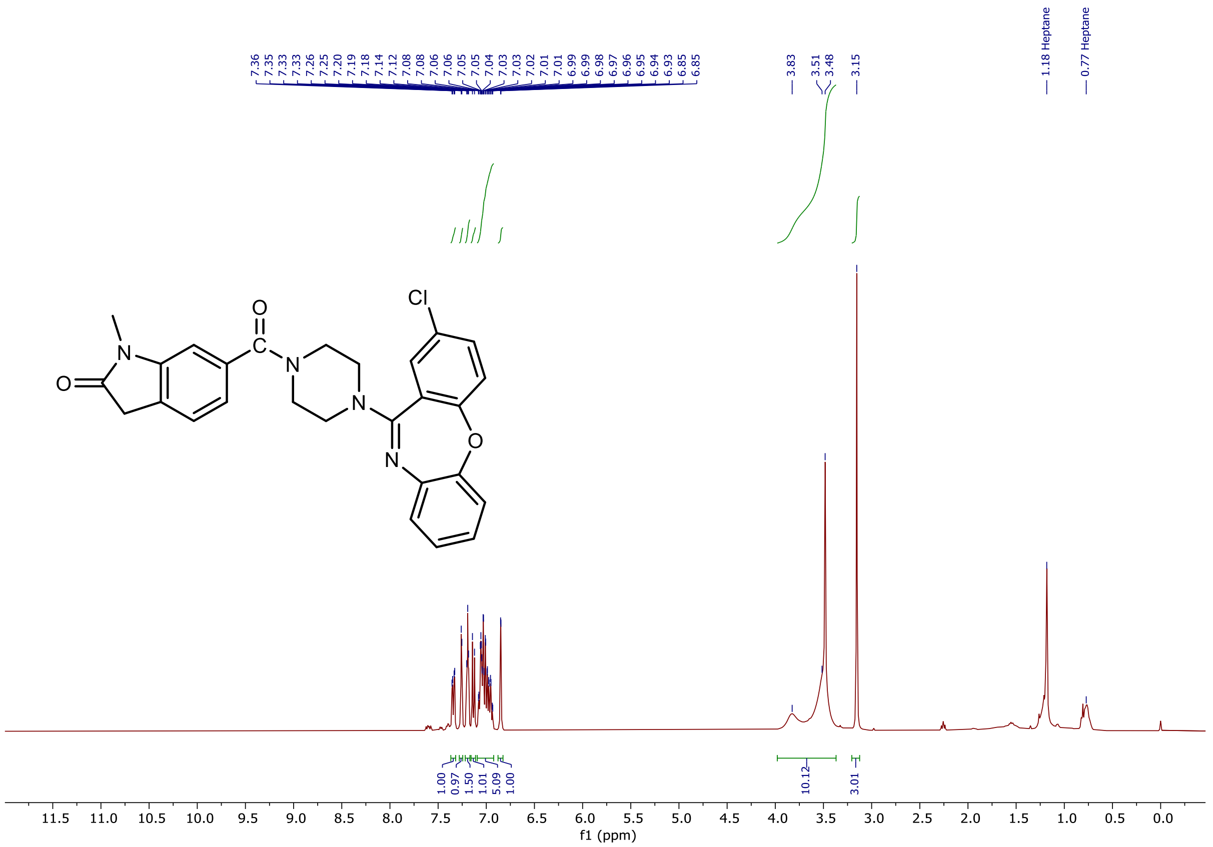

^{13}C-NMR

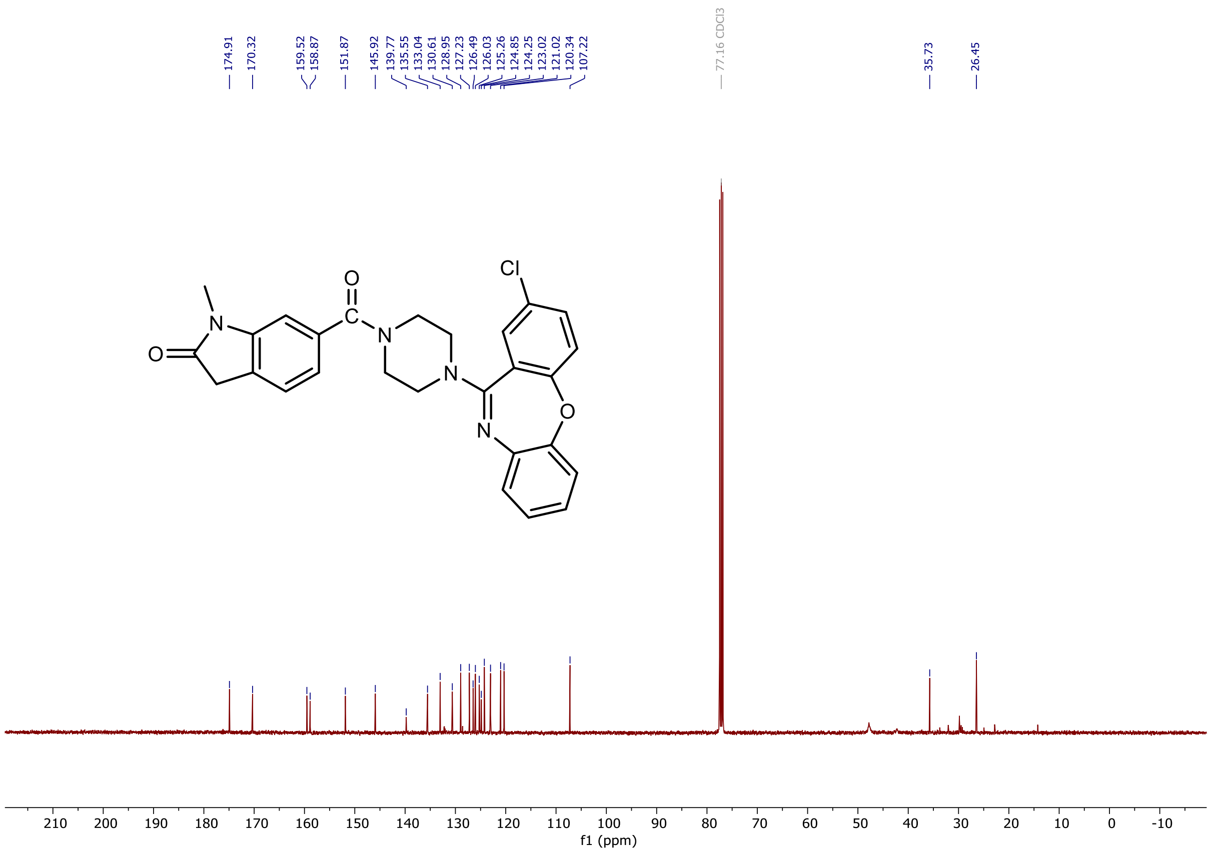

6-(4-(2-chlorodibenzo[*b,f*][1,4]oxazepin-11-yl)piperazine-1-^{13}C-carbonyl)-1-methylindolin-2-one (^{13}C-10p)

^{1}H-NMR

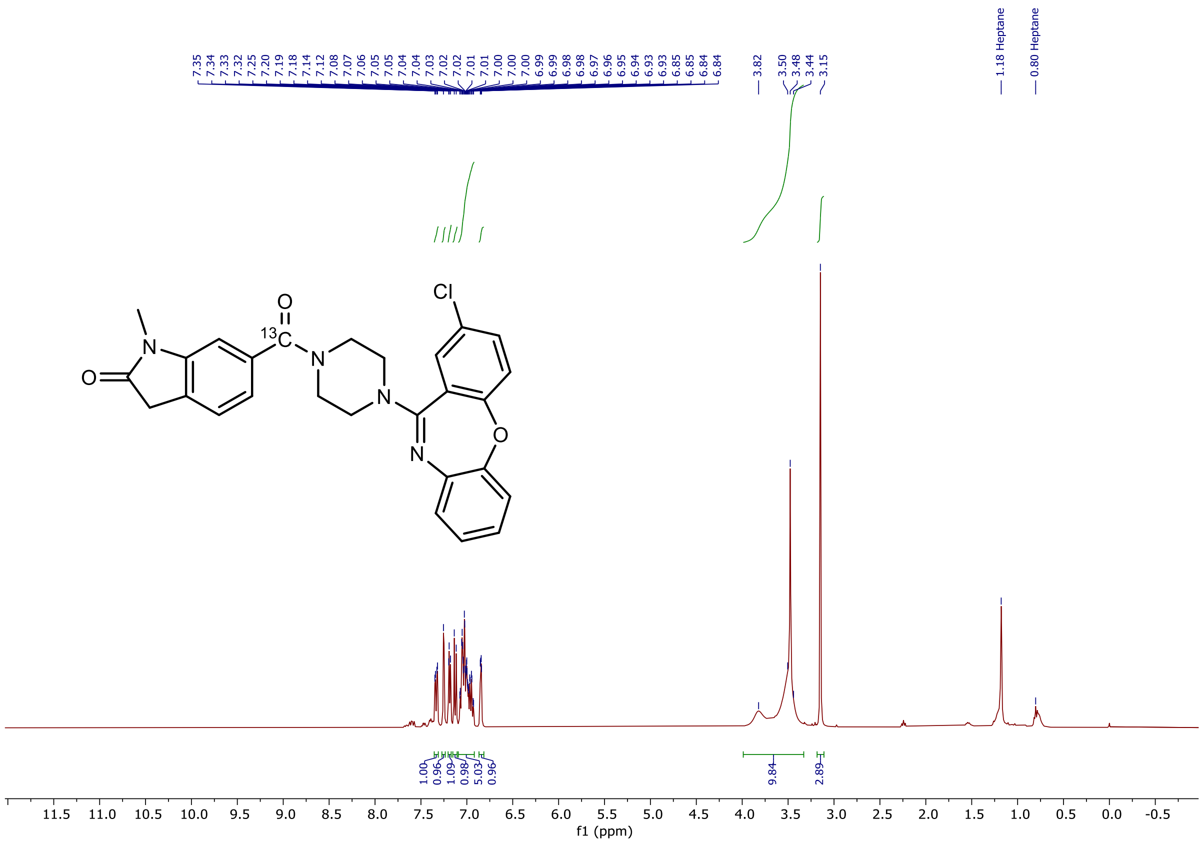

^{13}C-NMR

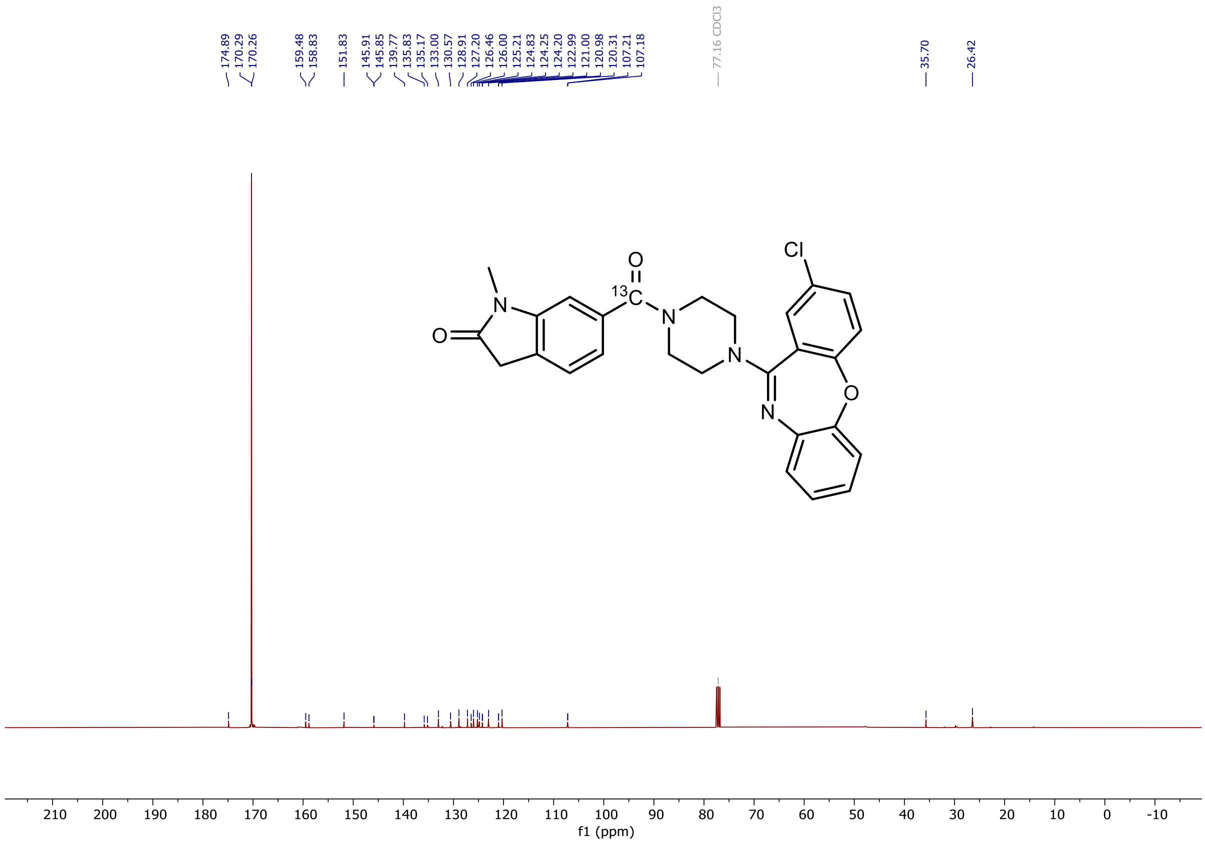

6-(4-(2-chlorodibenzo[*b,f*][1,4]oxazepin-11-yl)piperazine-1-^{14}C-carbonyl)-1-methylindolin-2-one (^{14}C-10p)

^{1}H-NMR

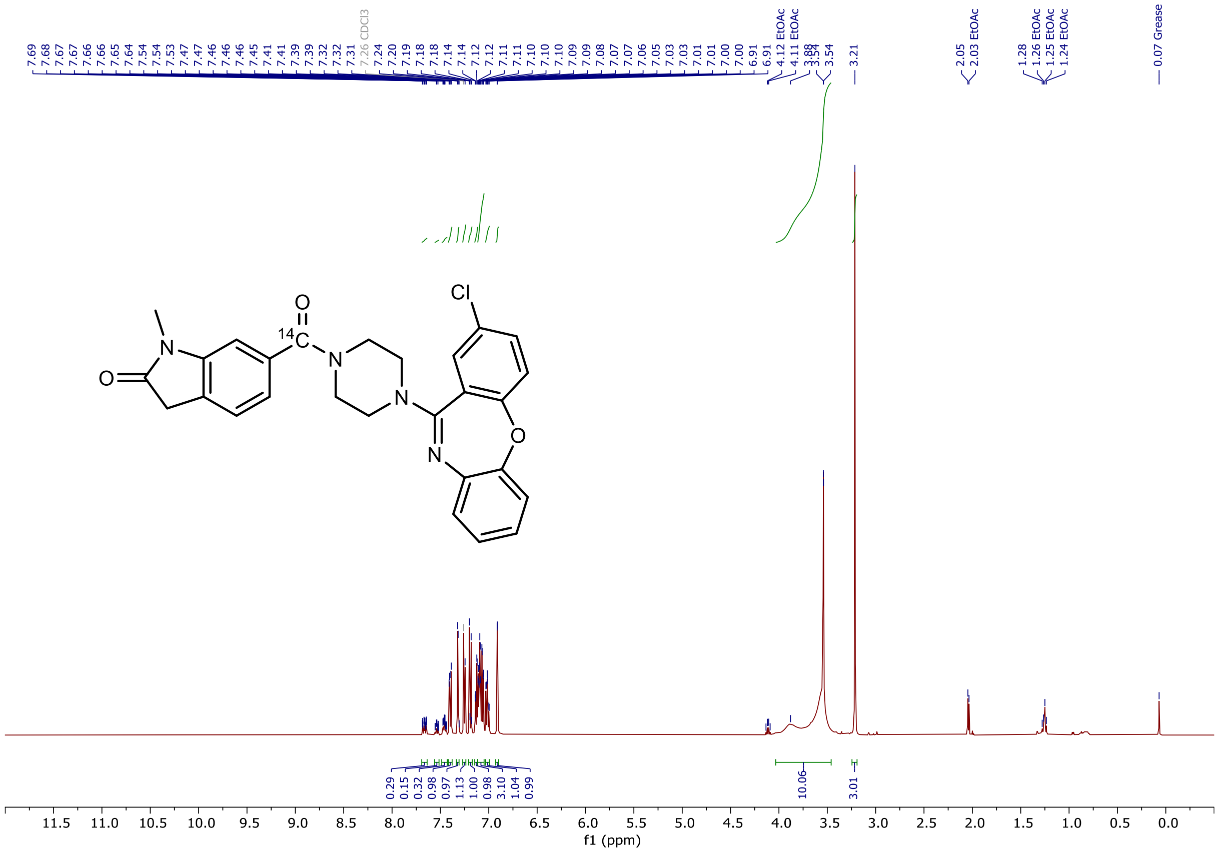

^{13}C-NMR

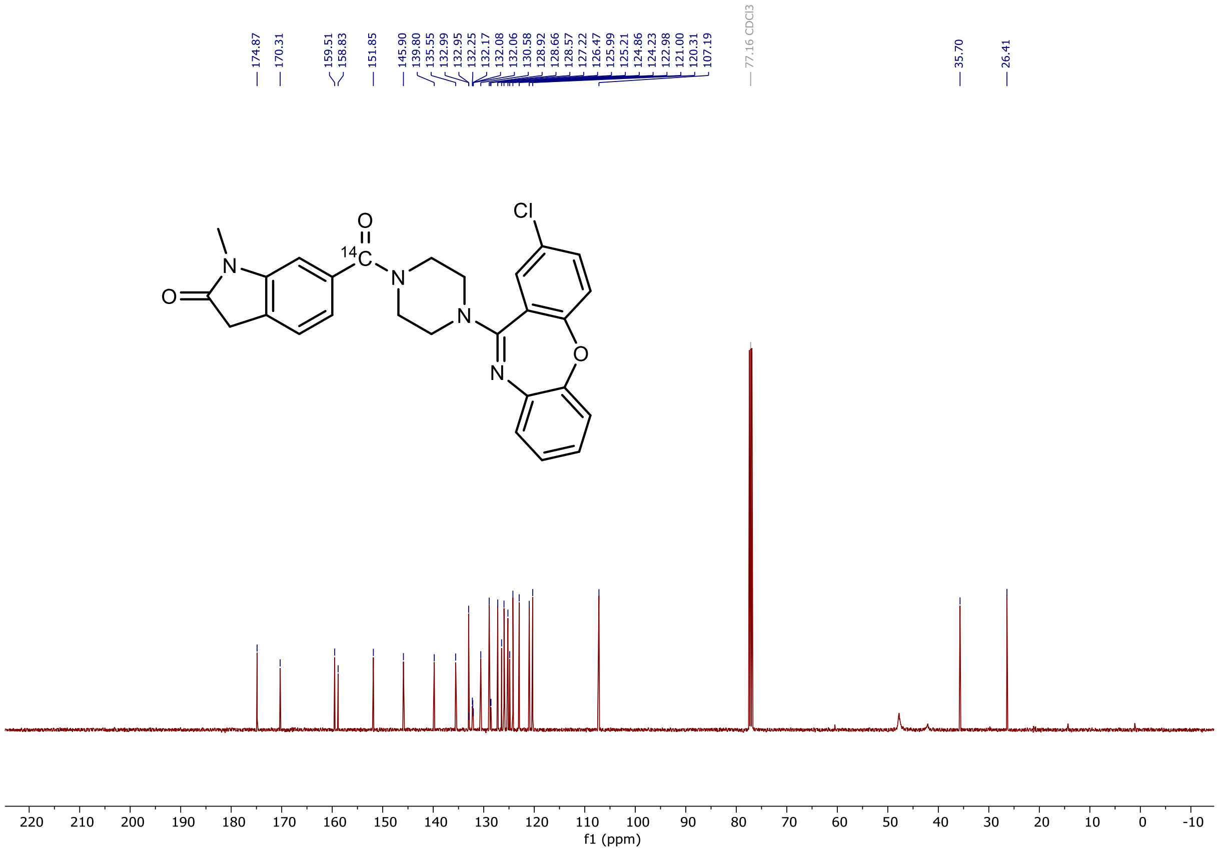

5-(4-(2-chlorodibenzo[b,f][1,4]oxazepin-11-yl)piperazine-1-carbonyl)-2-(2,6-dioxopiperidin-3-yl)isoindoline-1,3-dione (^{12}C-10q)

^{1}H-NMR

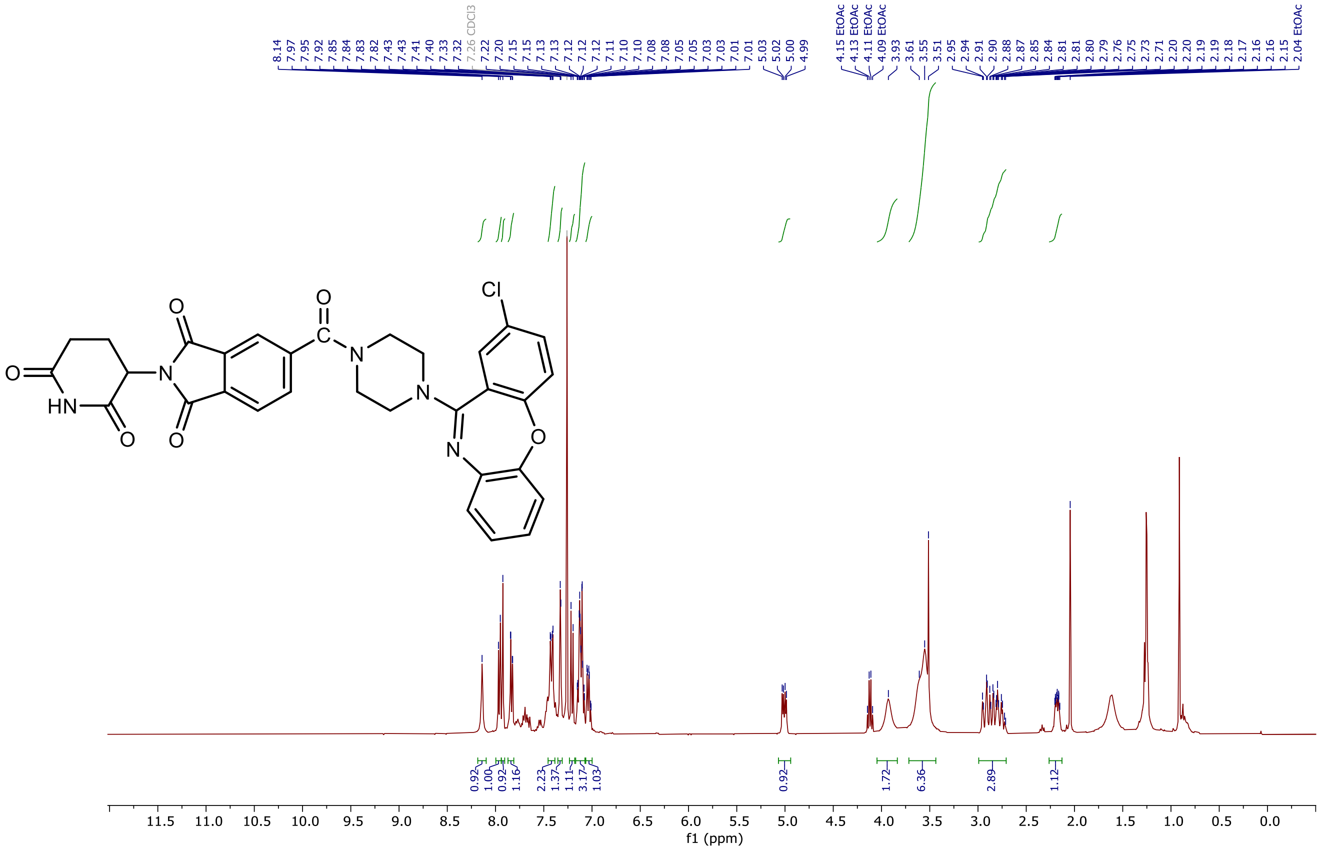

^{13}C-NMR

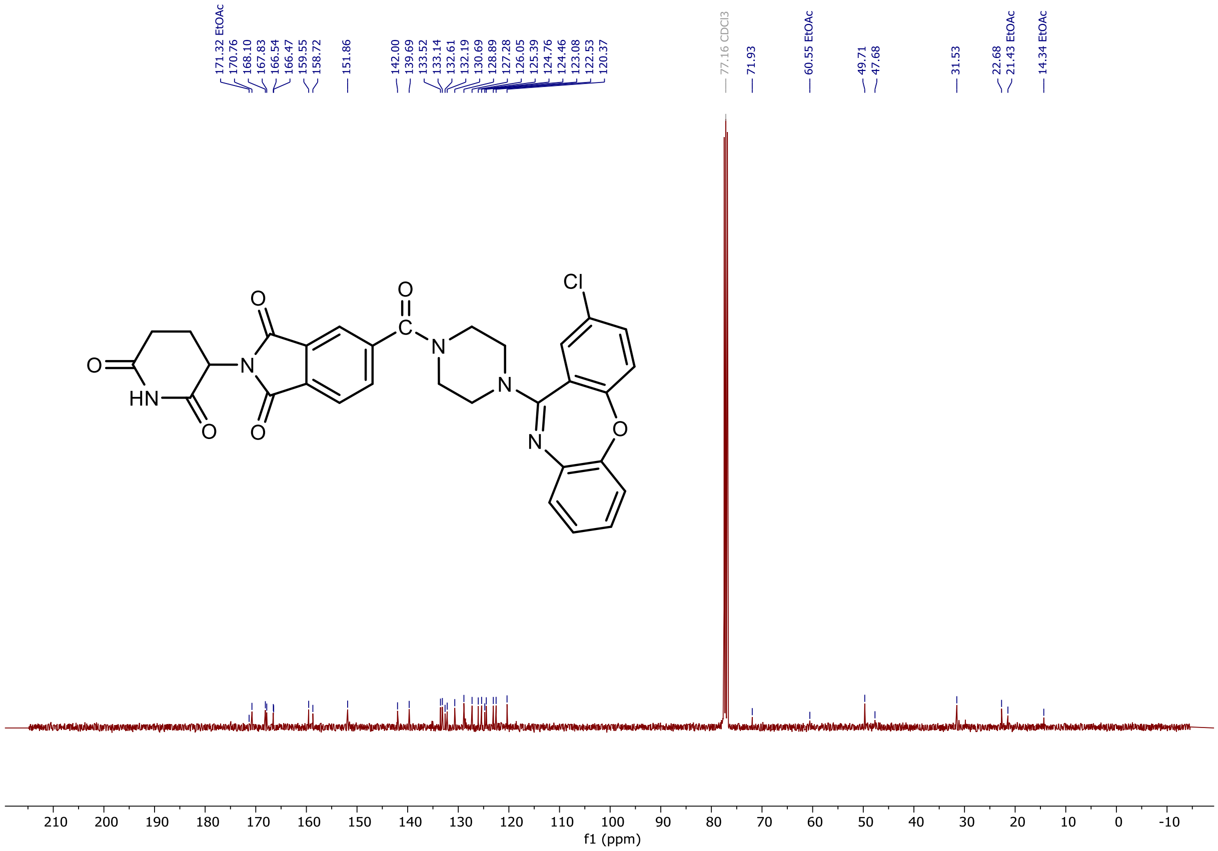

5-(4-(2-chlorodibenzo[b,f][1,4]oxazepin-11-yl)piperazine-1-^{13}C-carbonyl)-2-(2,6-dioxopiperidin-3-yl)isoindoline-1,3-dione (^{13}C-10q)

^{1}H-NMR

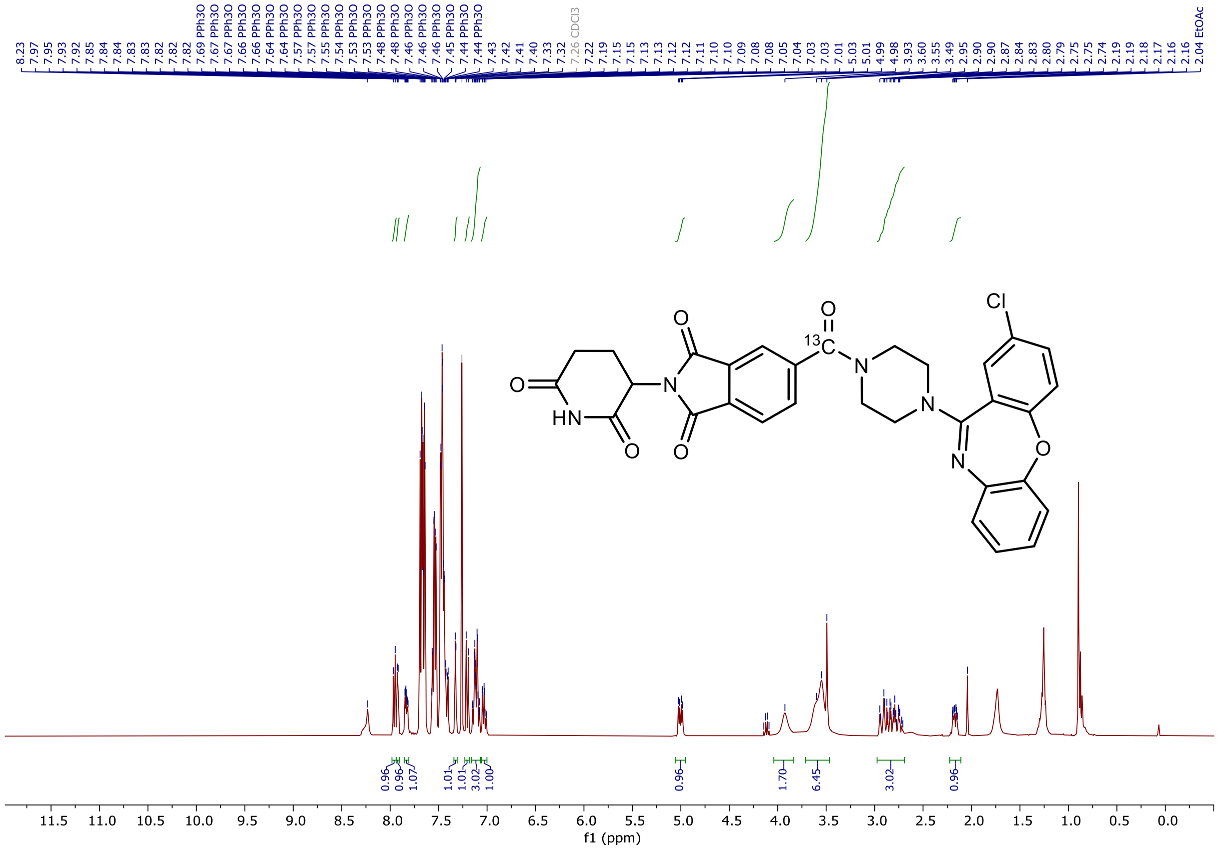

^{13}C-NMR

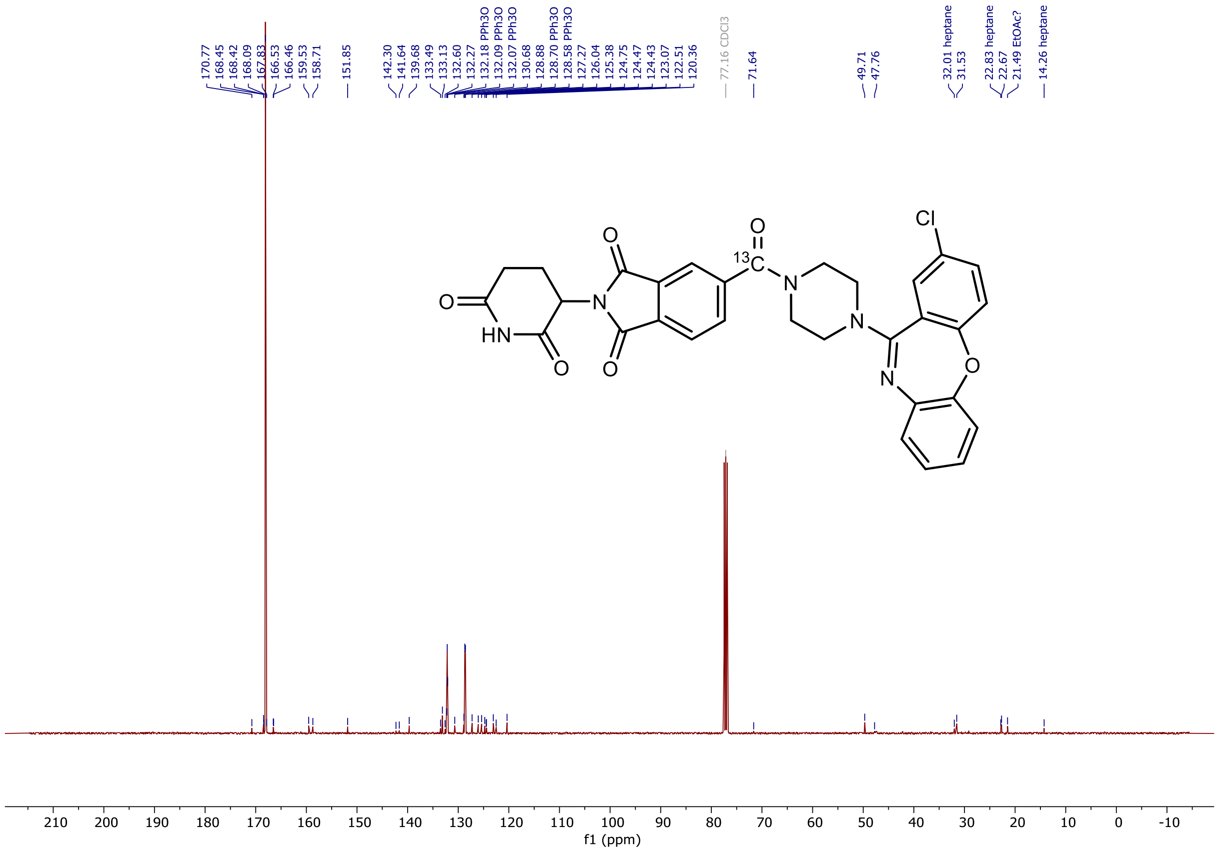

2-(2,6-dioxopiperidin-3-yl)-5-(piperidine-1-carbonyl)isoindoline-1,3-dione (^{12}C-5q)

^{1}H-NMR

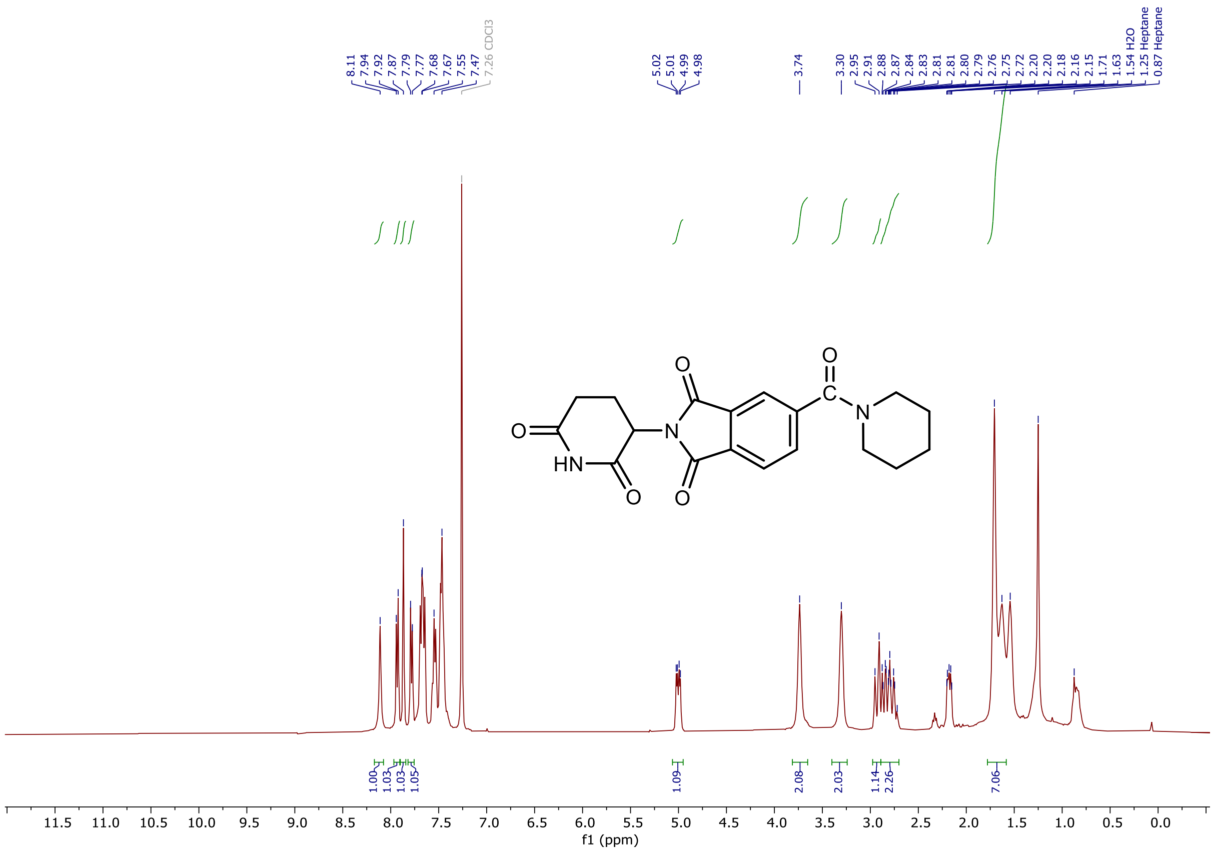

^{13}C-NMR

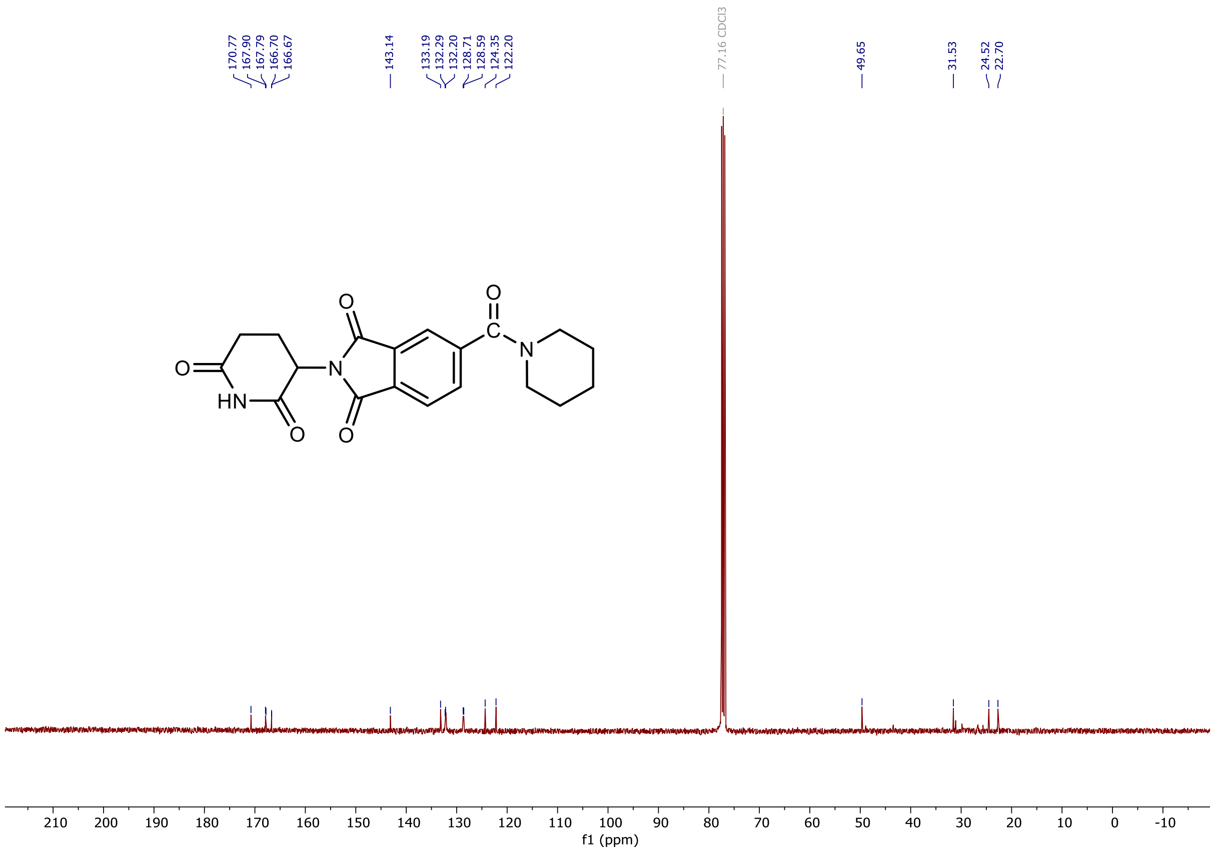

2-(2,6-dioxopiperidin-3-yl)-5-(piperidine-1-^{13}C -carbonyl)isoindoline-1,3-dione (^{13}C-5q)

^{1}H-NMR

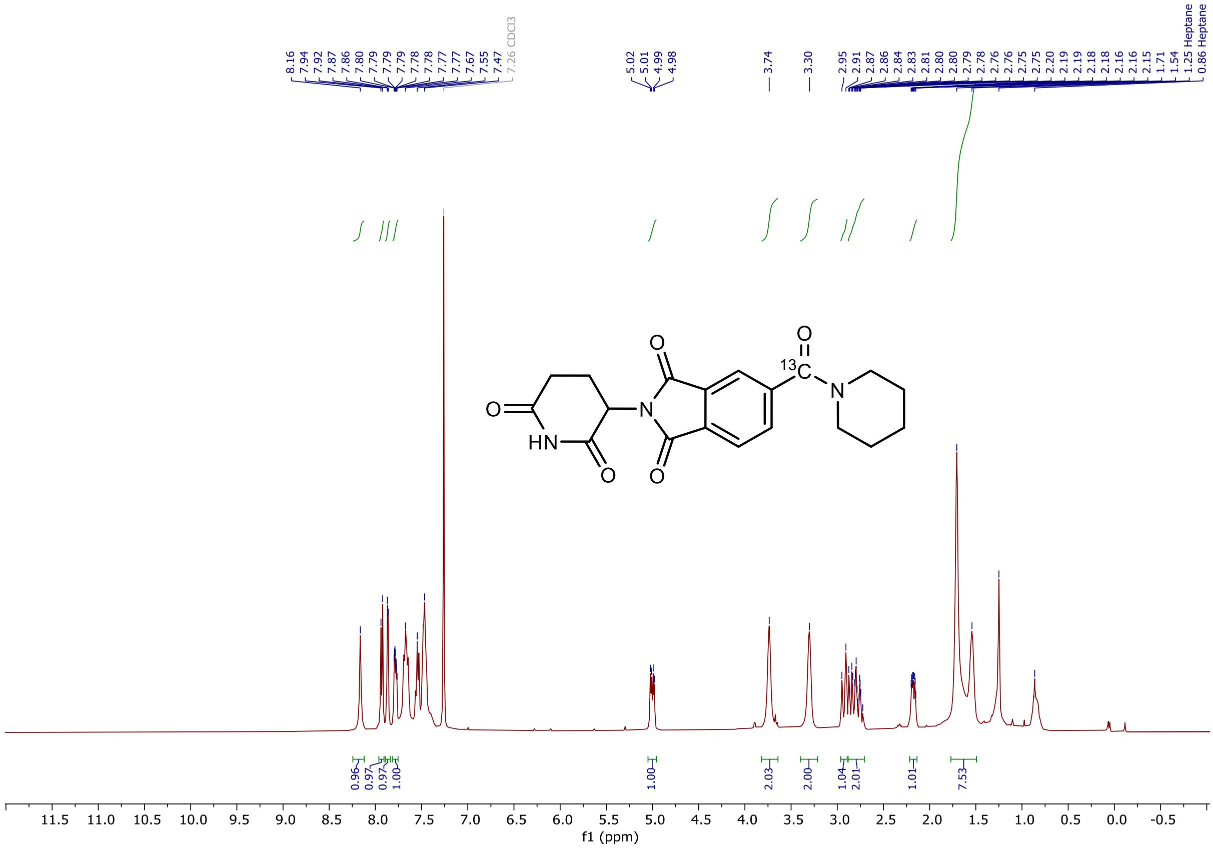

^{13}C-NMR

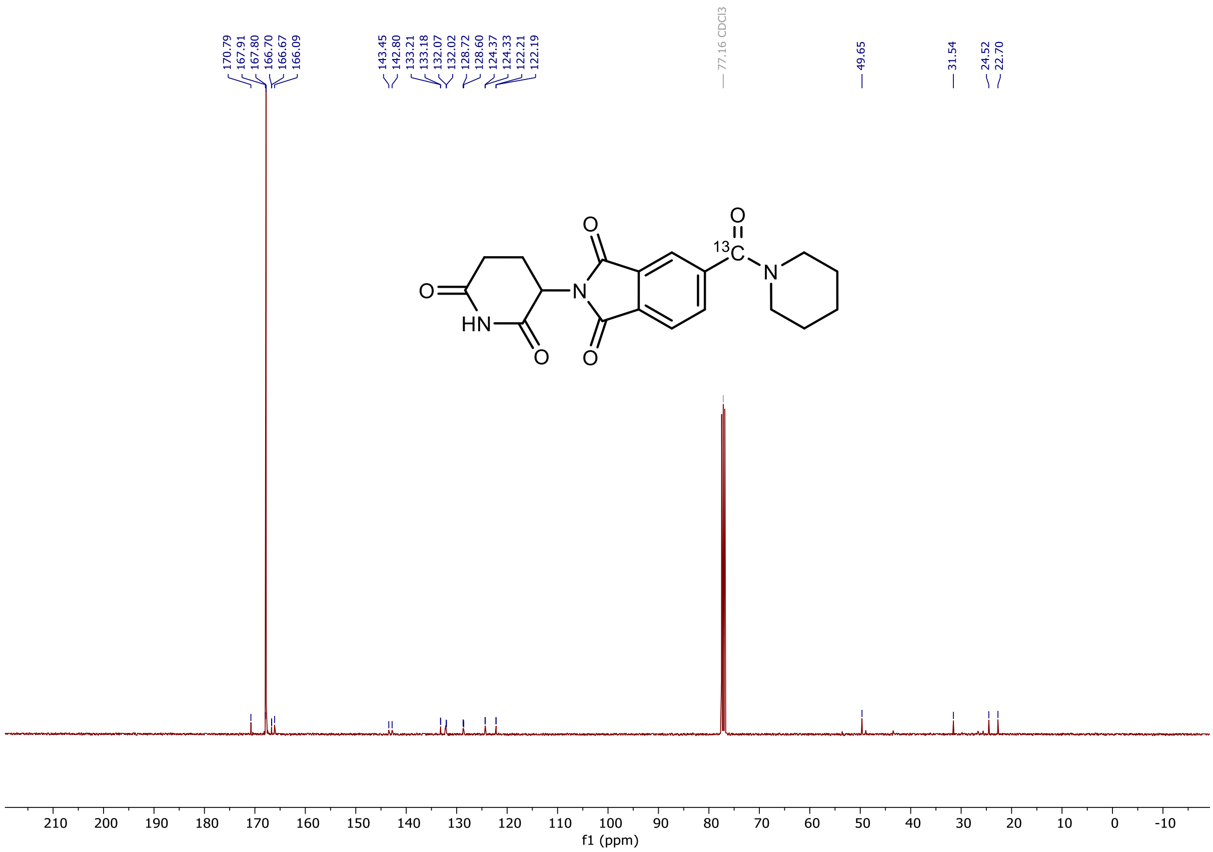

7-(4-([1,1'-biphenyl]-4-carbonyl)piperazin-1-yl)-1-cyclopropyl-6-fluoro-4-oxo-1,4-dihydroquinoline-3-carboxylic acid (^{12}C-11a)

^{1}H-NMR

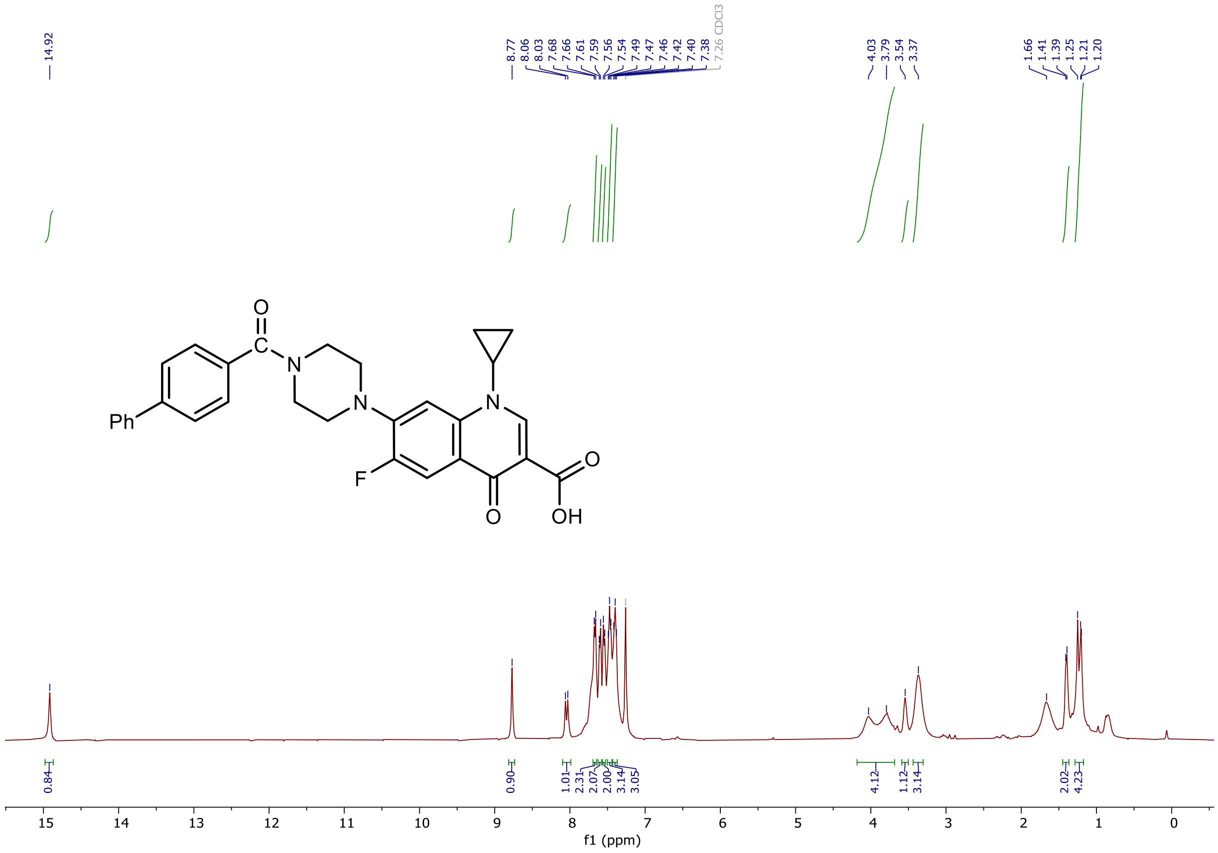

^{13}C-NMR

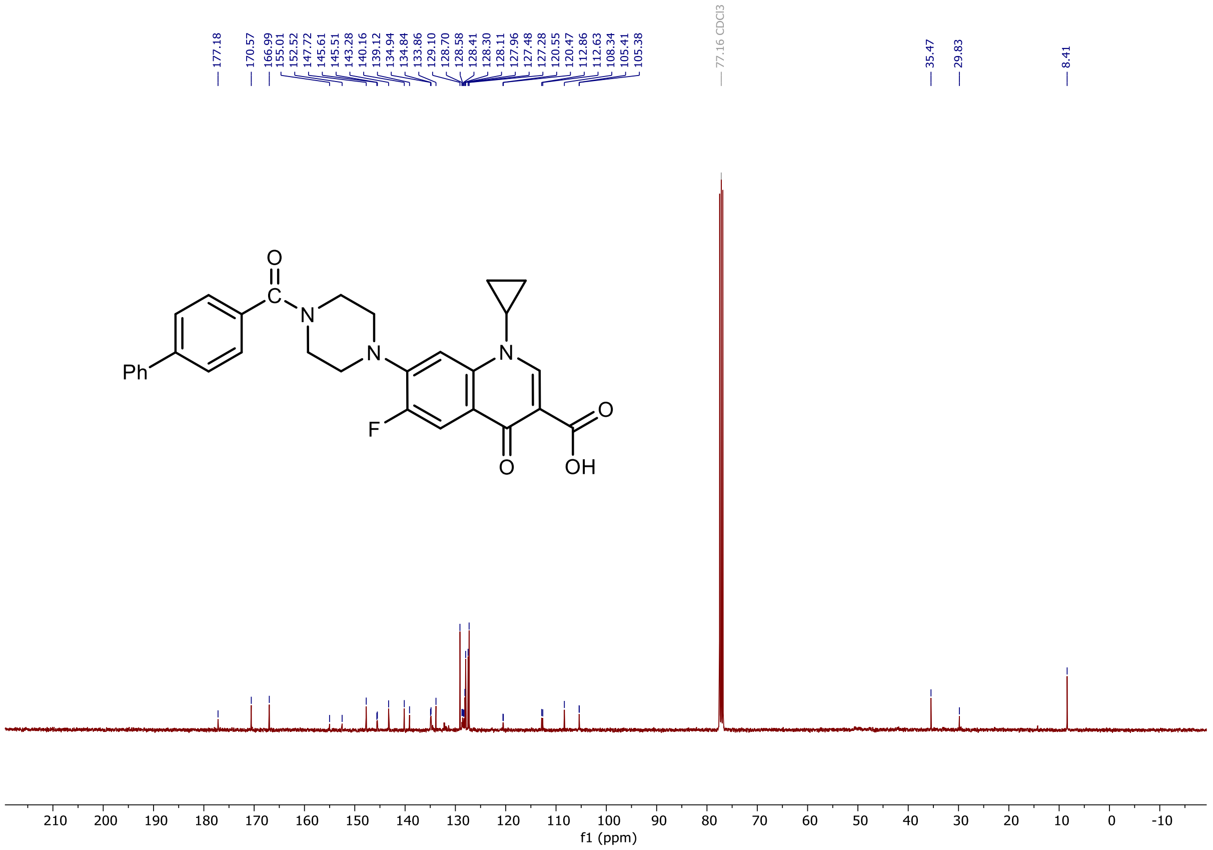

^{19}F-NMR

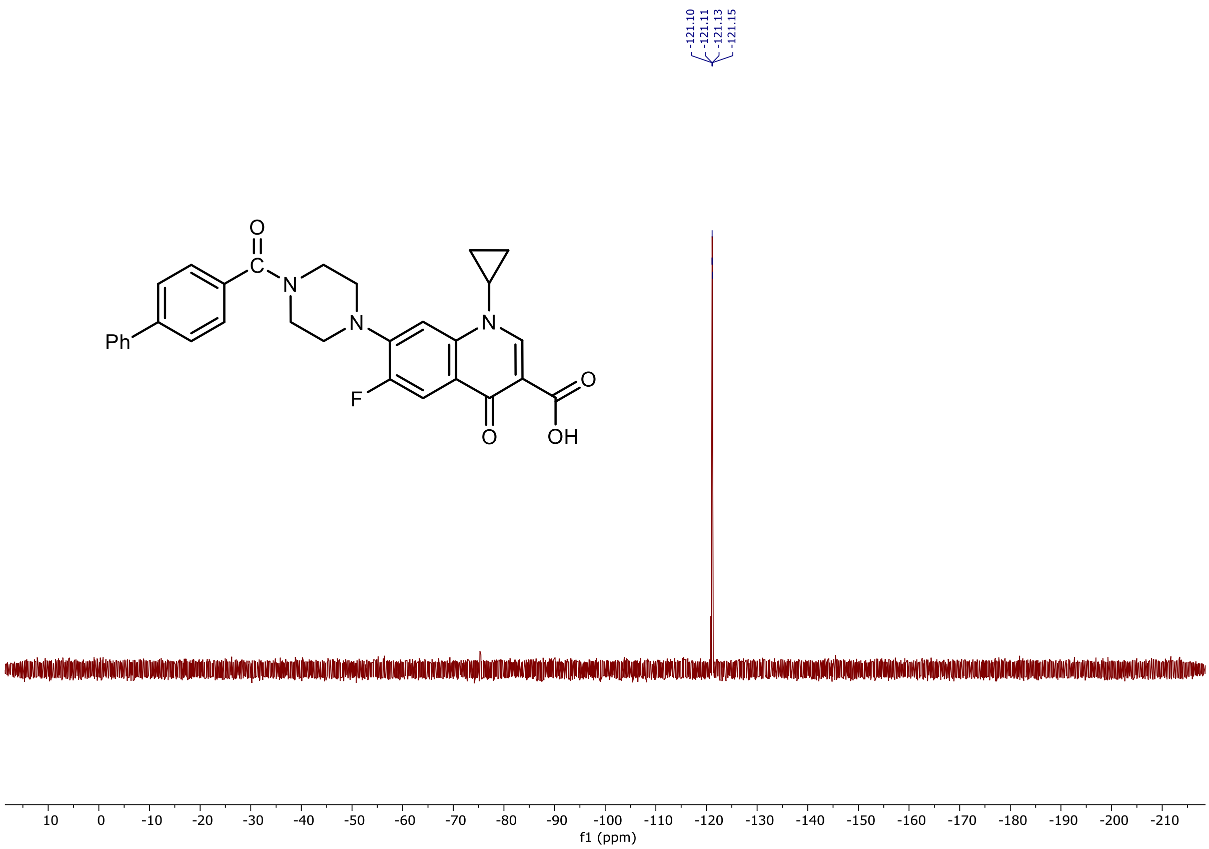

O
C
N
N
N
Ph
F
O
OH
O
-121.10
-121.11
-121.13
-121.15
10 0 -10 -20 -30 -40 -50 -60 -70 -80 -90 -100 -110 -120 -130 -140 -150 -160 -170 -180 -190 -200 -210
f1 (ppm)

7-(4-([1,1'-biphenyl]-4-^{13}C-carbonyl)piperazin-1-yl)-1-cyclopropyl-6-fluoro-4-oxo-1,4-dihydroquinoline-3-carboxylic acid (^{13}C-11a)

^{1}H-NMR

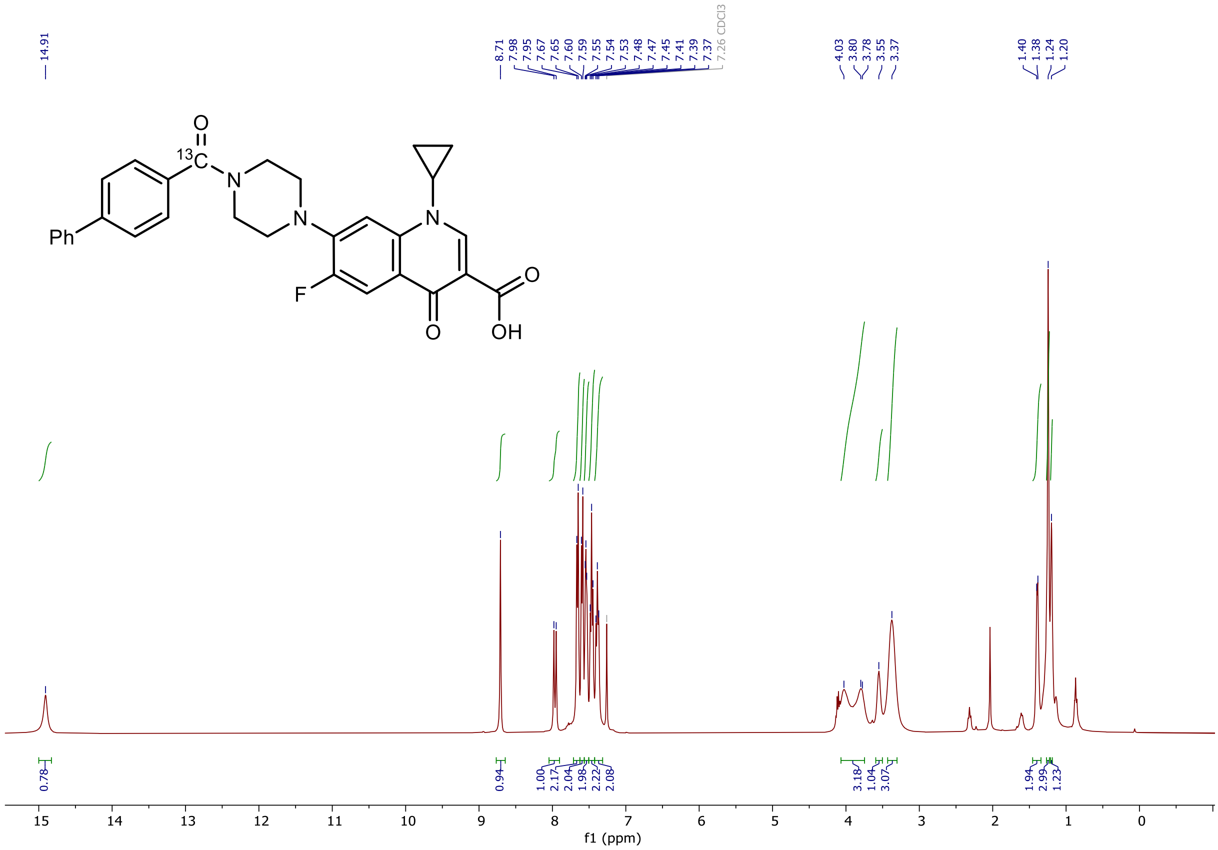

^{13}C-NMR

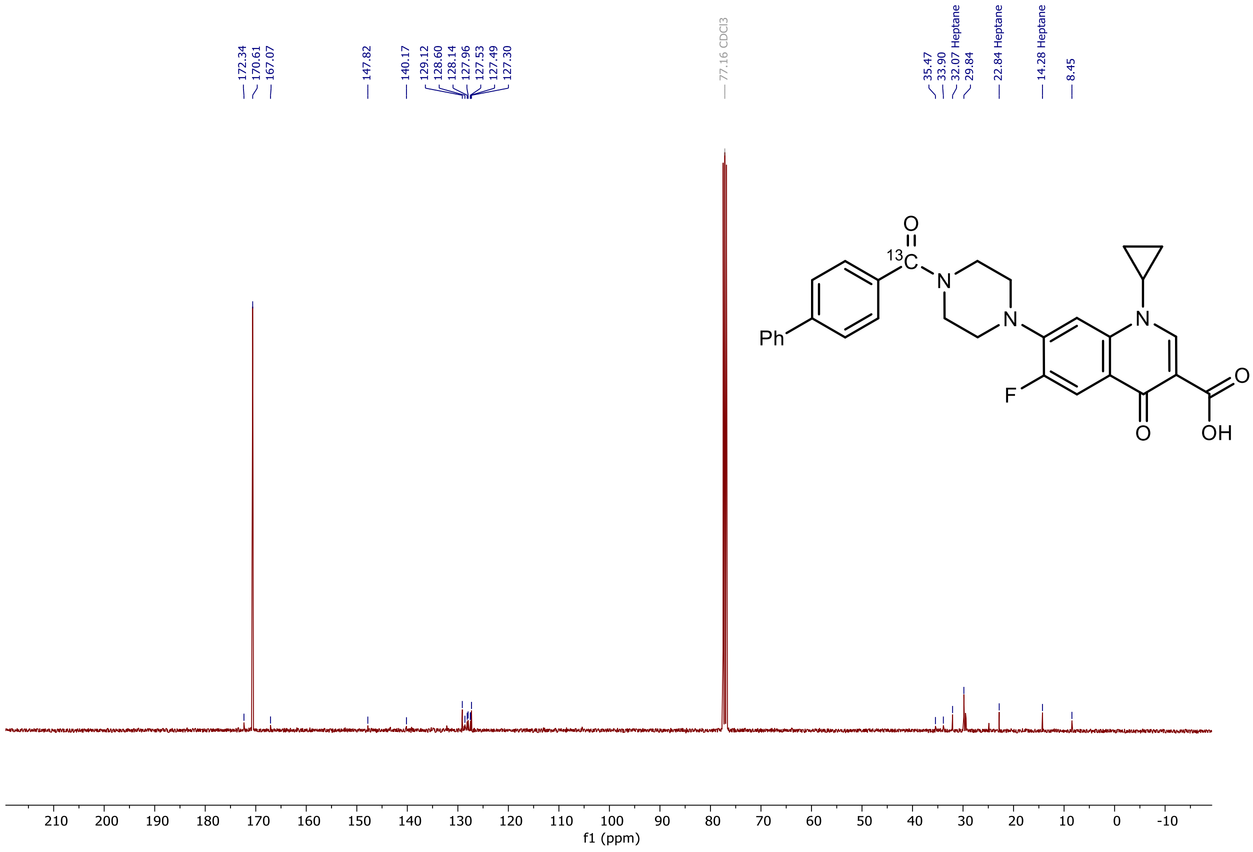

^{19}F-NMR

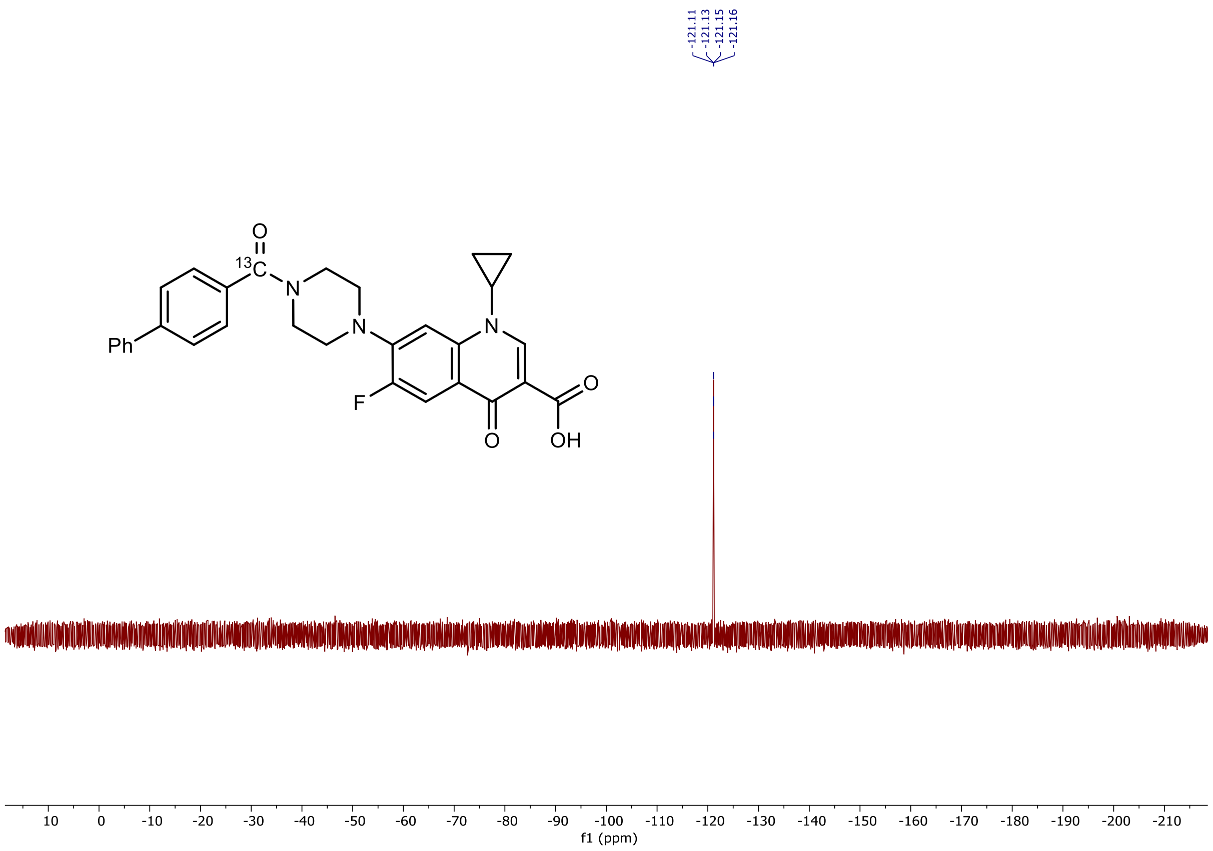

-121.11
-121.13
-121.15
-121.16
Ph
^{13}C
O
N
N
N
F
O
OH
10
0
-10
-20
-30
-40
-50
-60
-70
-80
-90
-100
-110
-120
-130
-140
-150
-160
-170
-180
-190
-200
-210
f1 (ppm)

[1,1'-biphenyl]-4-yl((3*S*,4*R*)-3-((benzo[*d*][1,3]dioxol-5-yloxy)methyl)-4-(4-fluorophenyl)-piperidin-1-yl)methanone (^{12}C-12a)

^{1}H-NMR

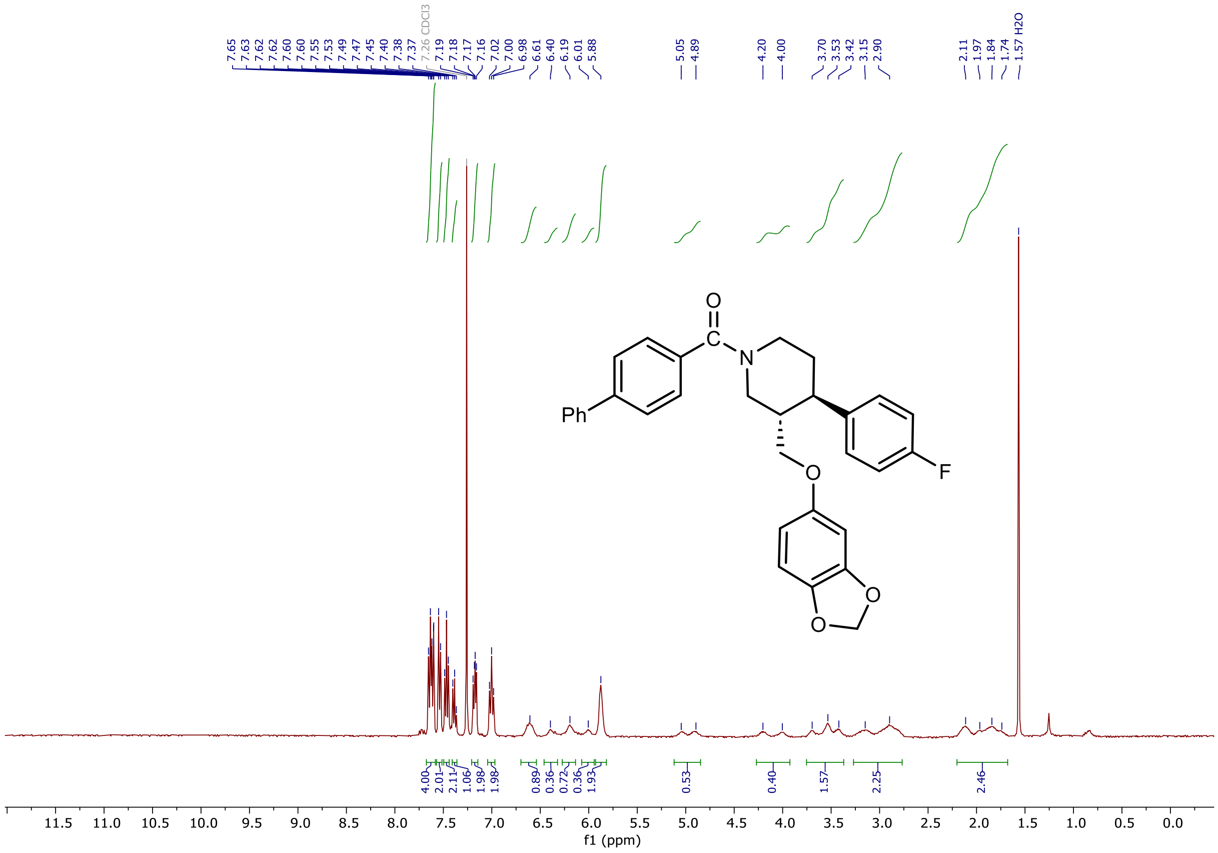

^{13}C-NMR

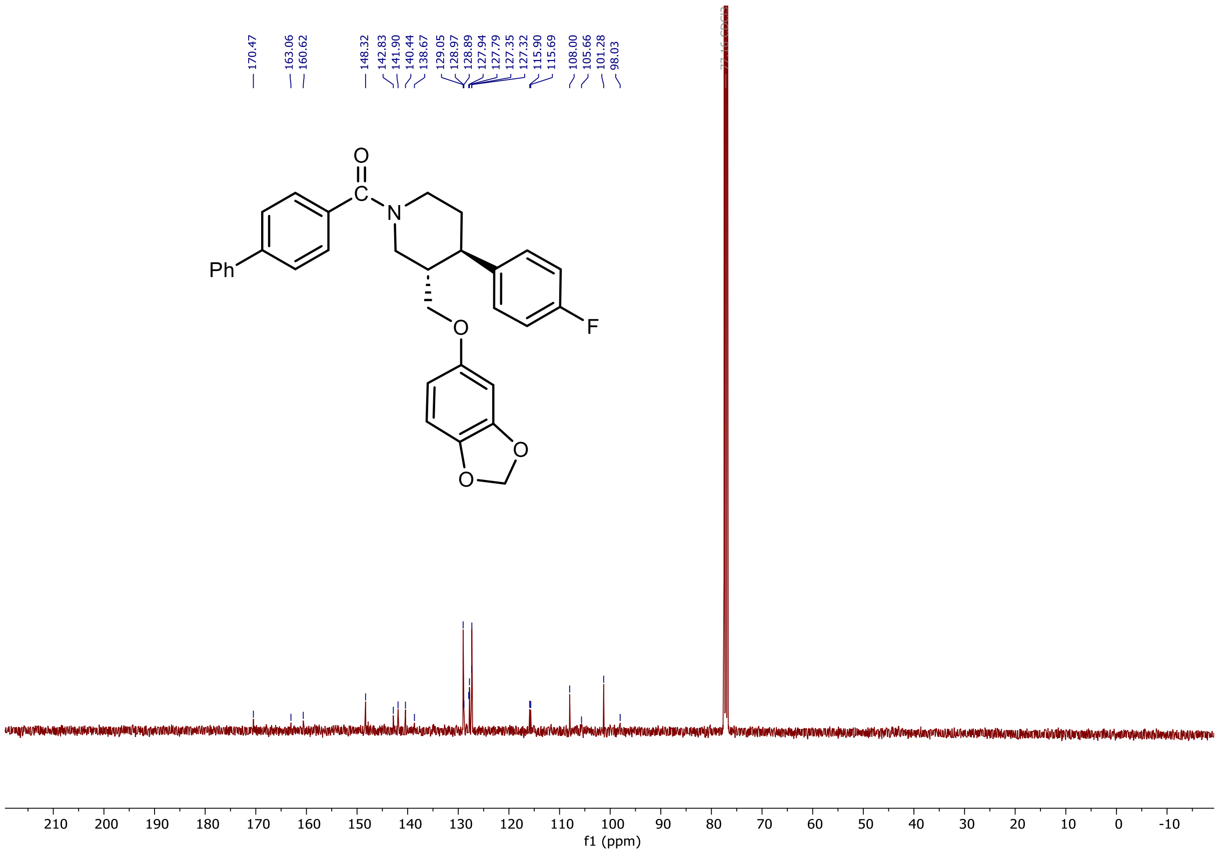

^{19}F-NMR

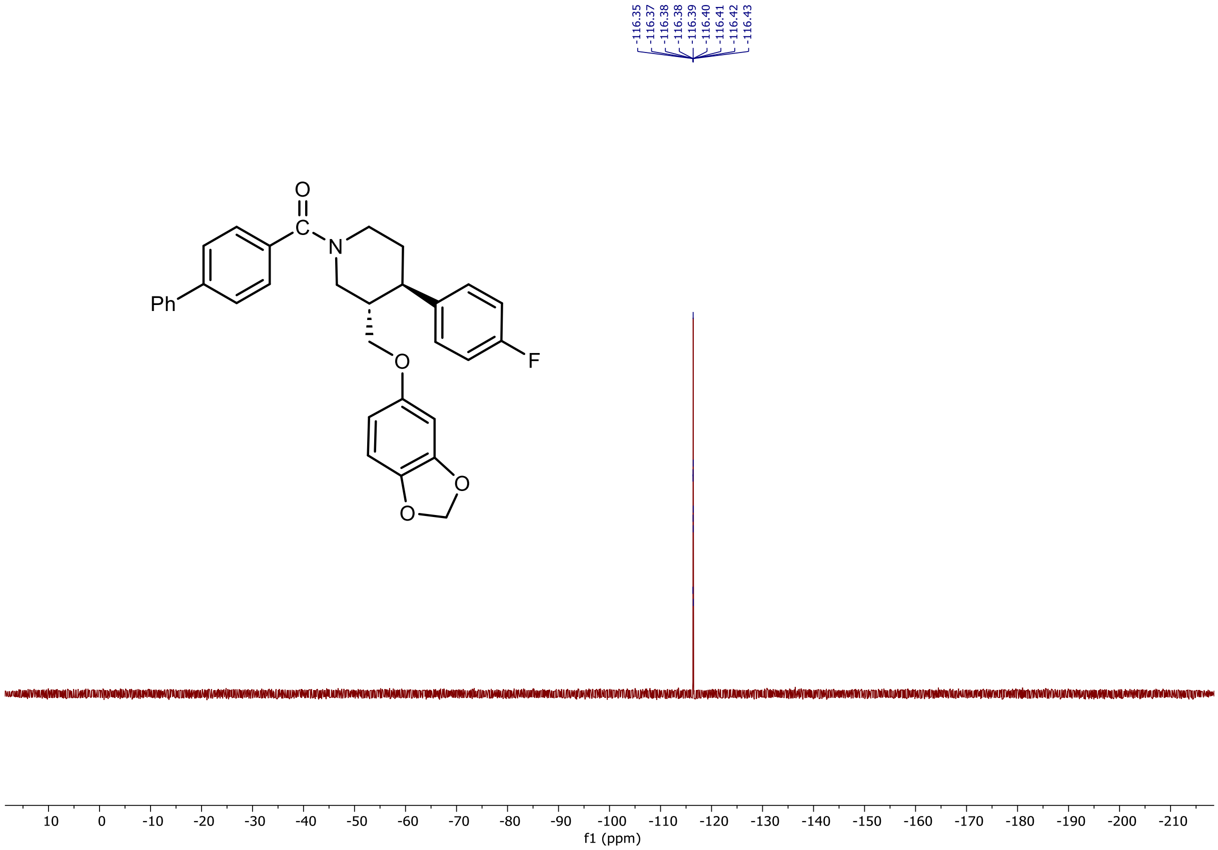

-116.35
-116.37
-116.38
-116.38
-116.39
-116.40
-116.41
-116.42
-116.43
O
C
N
Ph
O
F
O
O
10
0
-10
-20
-30
-40
-50
-60
-70
-80
-90
-100
-110
-120
-130
-140
-150
-160
-170
-180
-190
-200
-210
f1 (ppm)

[1,1'-biphenyl]-4-yl((3*S*,4*R*)-3-((benzo[*d*][1,3]dioxol-5-yloxy)methyl)-4-(4-fluorophenyl)-piperidin-1-yl)-^{13}C-methanone (^{13}C-12a)

^{1}H-NMR

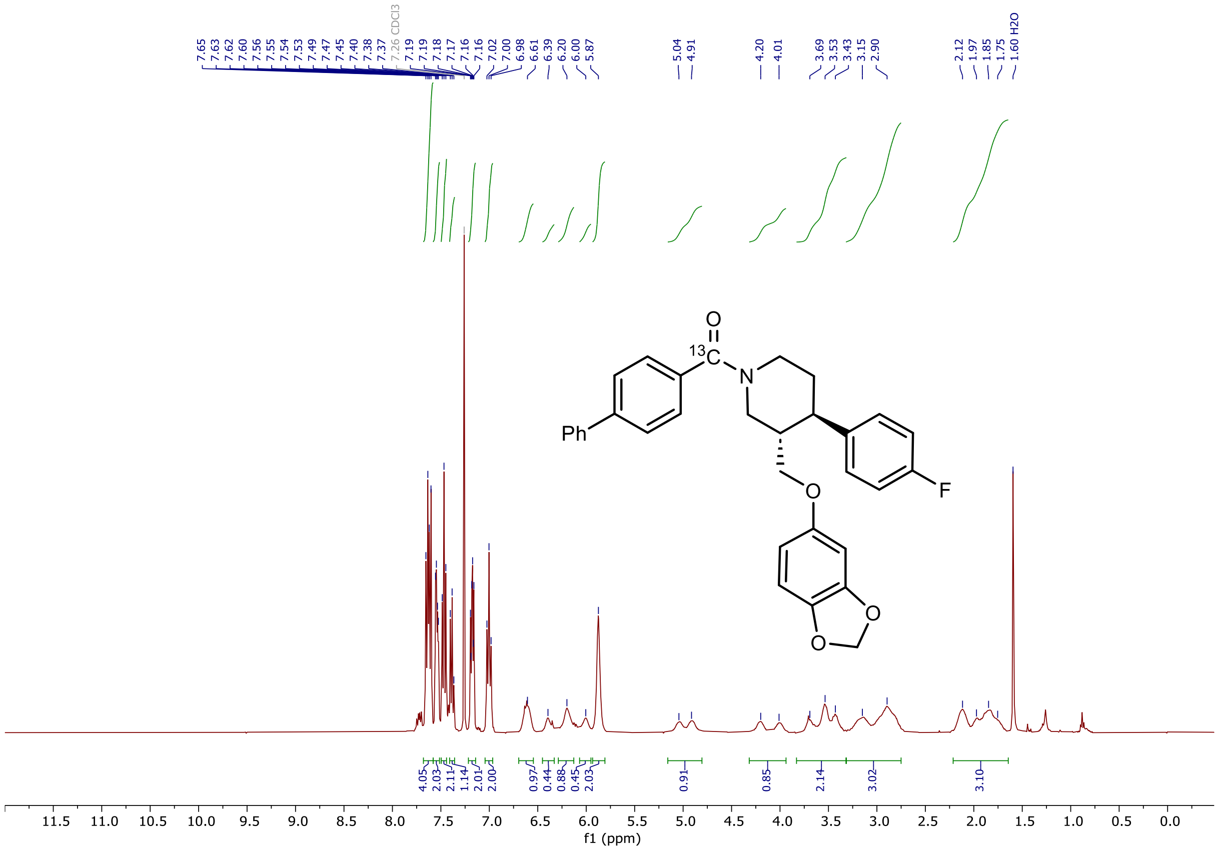

^{13}C-NMR

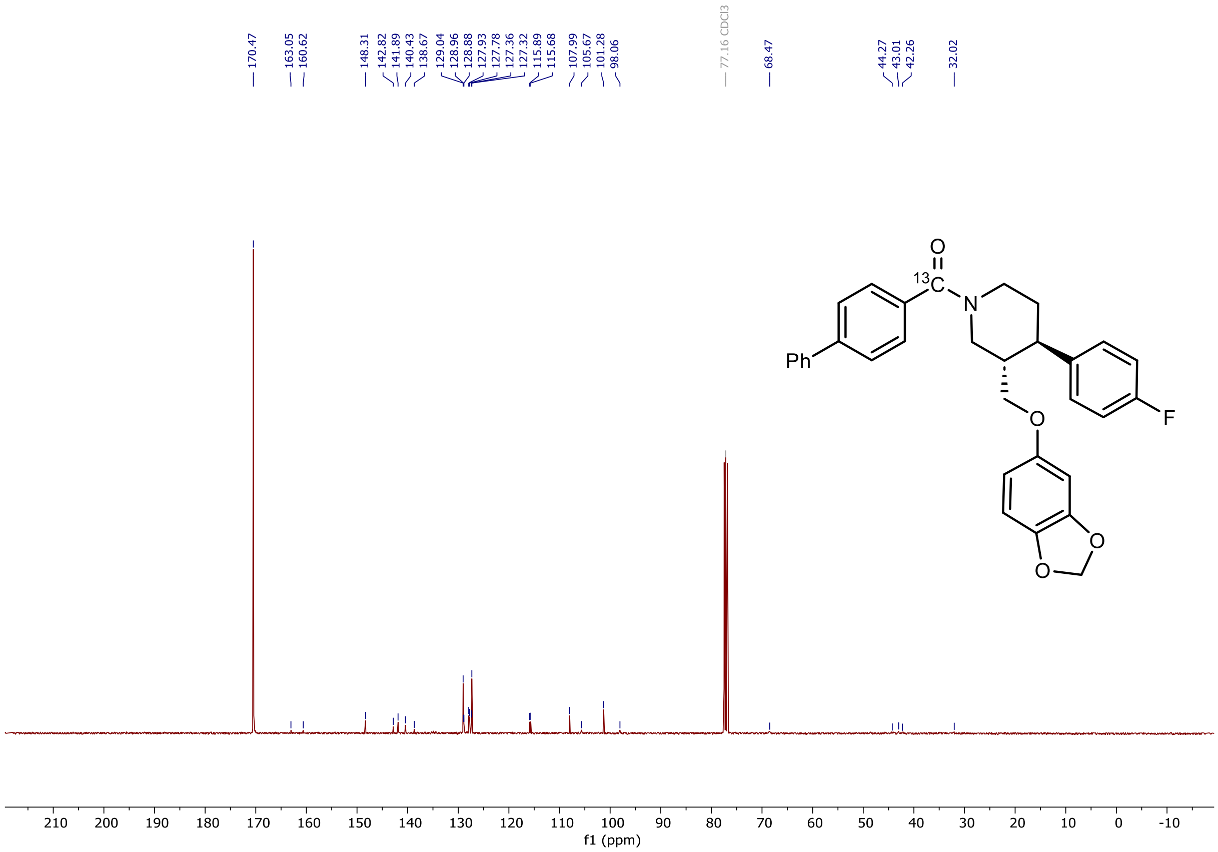

^{19}F-NMR

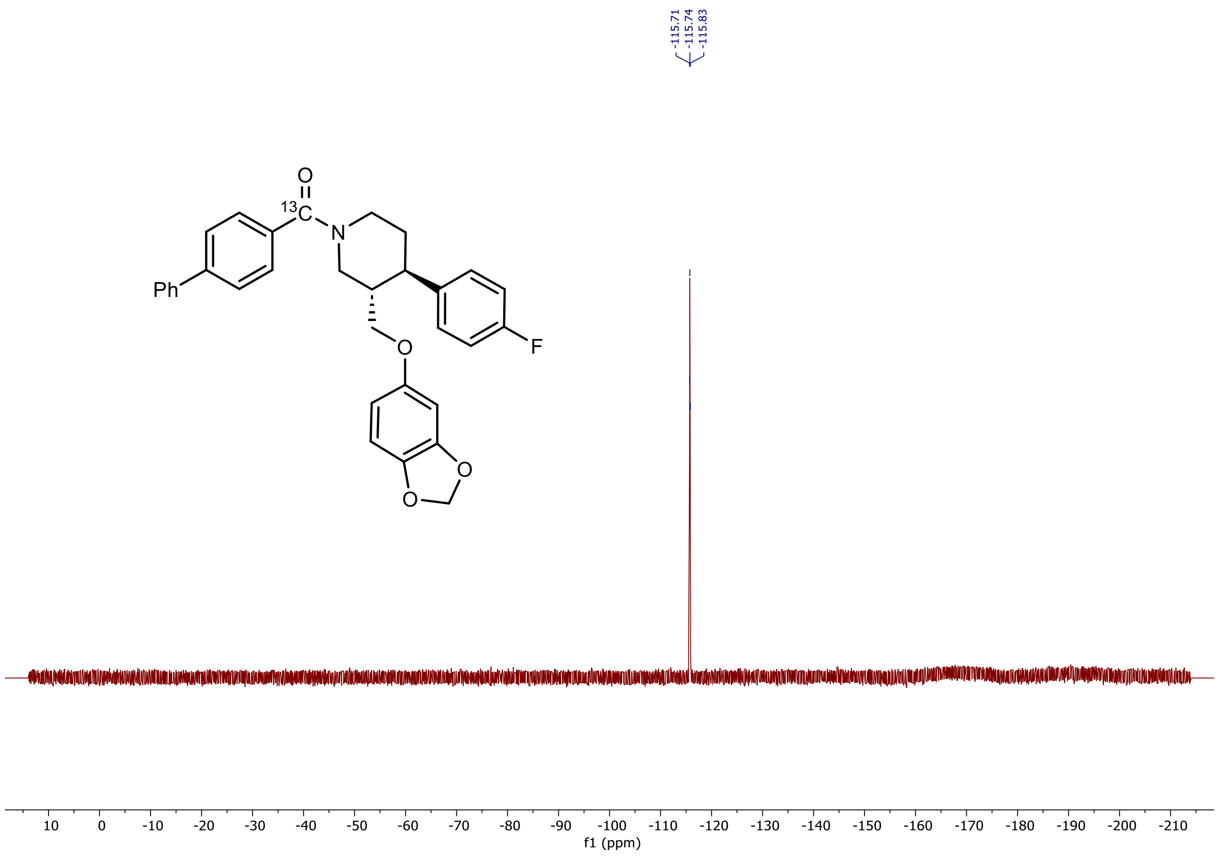

-115.71
-115.74
-115.83
Ph
O
13C
N
F
O
O
O
10
0
-10
-20
-30
-40
-50
-60
-70
-80
-90
-100
-110
-120
-130
-140
-150
-160
-170
-180
-190
-200
-210
f1 (ppm)

((3R,4S)-3-((benzo[d][1,3]dioxol-5-yloxy)methyl)-4-(4-fluorophenyl)piperidin-1-yl)(4-(((2-ethylquinolin-4-yl)oxy)methyl)phenyl)methanone (^{12}C-12r)

^{1}H-NMR

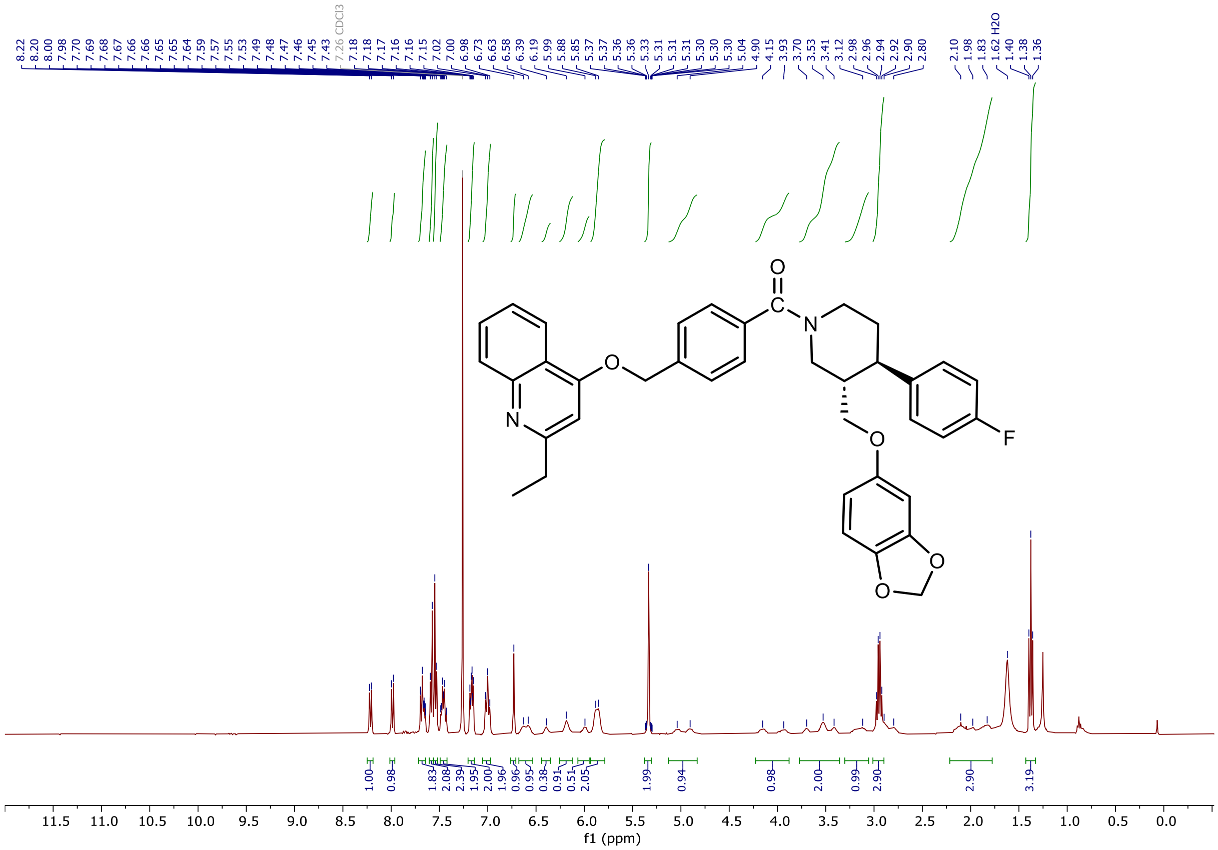

^{13}C-NMR

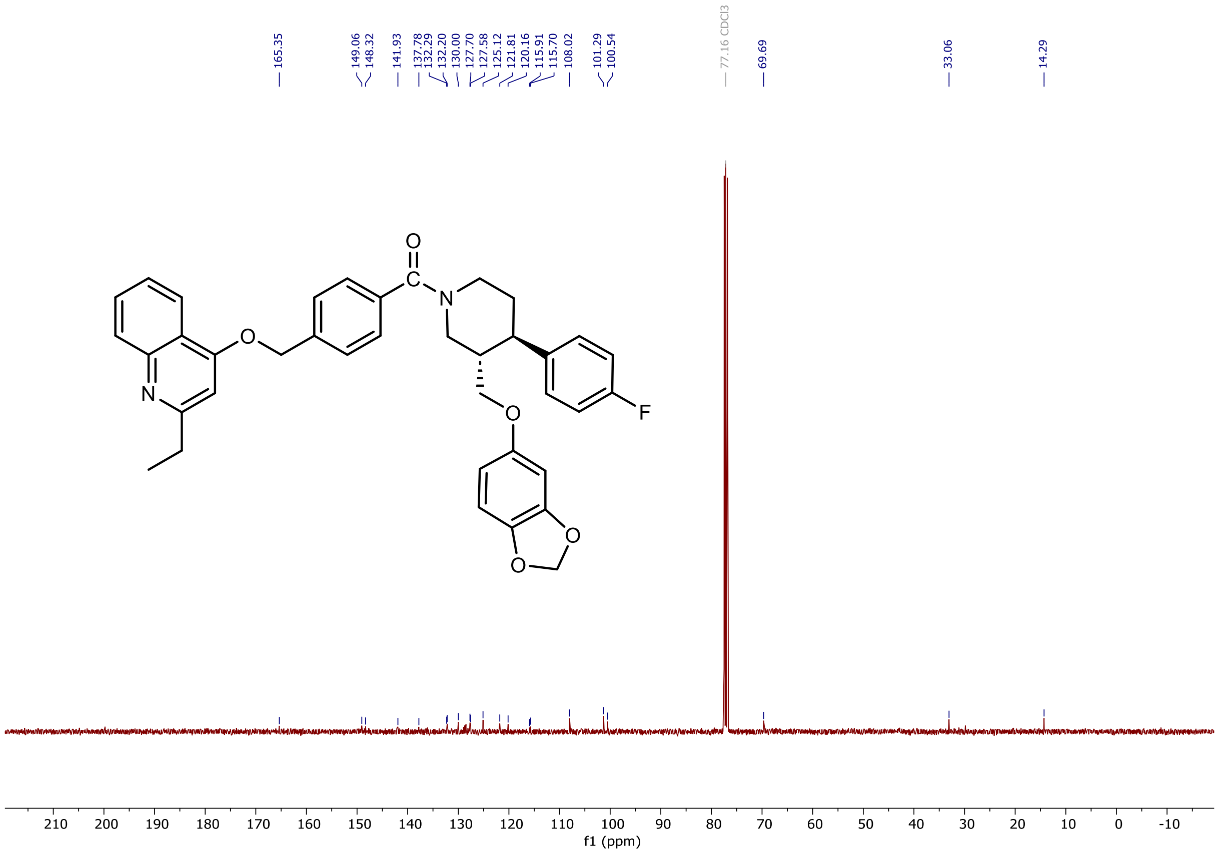

^{19}F-NMR

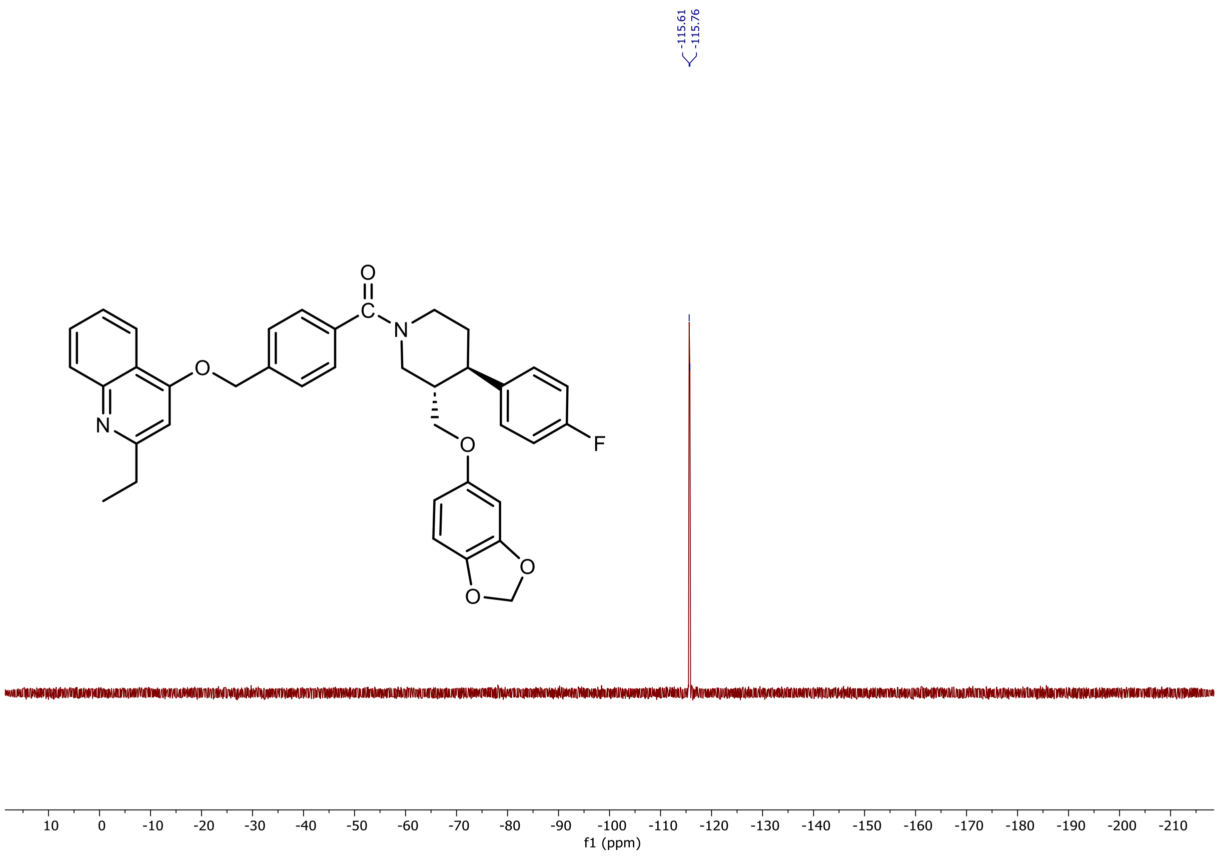

-115.61
-115.76
O
C
N
O
N
F
O
O
O
10
0
-10
-20
-30
-40
-50
-60
-70
-80
-90
-100
-110
-120
-130
-140
-150
-160
-170
-180
-190
-200
-210
f1 (ppm)

((3R,4S)-3-((benzo[d][1,3]dioxol-5-yloxy)methyl)-4-(4-fluorophenyl)piperidin-1-yl)(4-(((2-ethylquinolin-4-yl)oxy)methyl)phenyl)-^{13}C-methanone (^{13}C-12r)

^{1}H-NMR

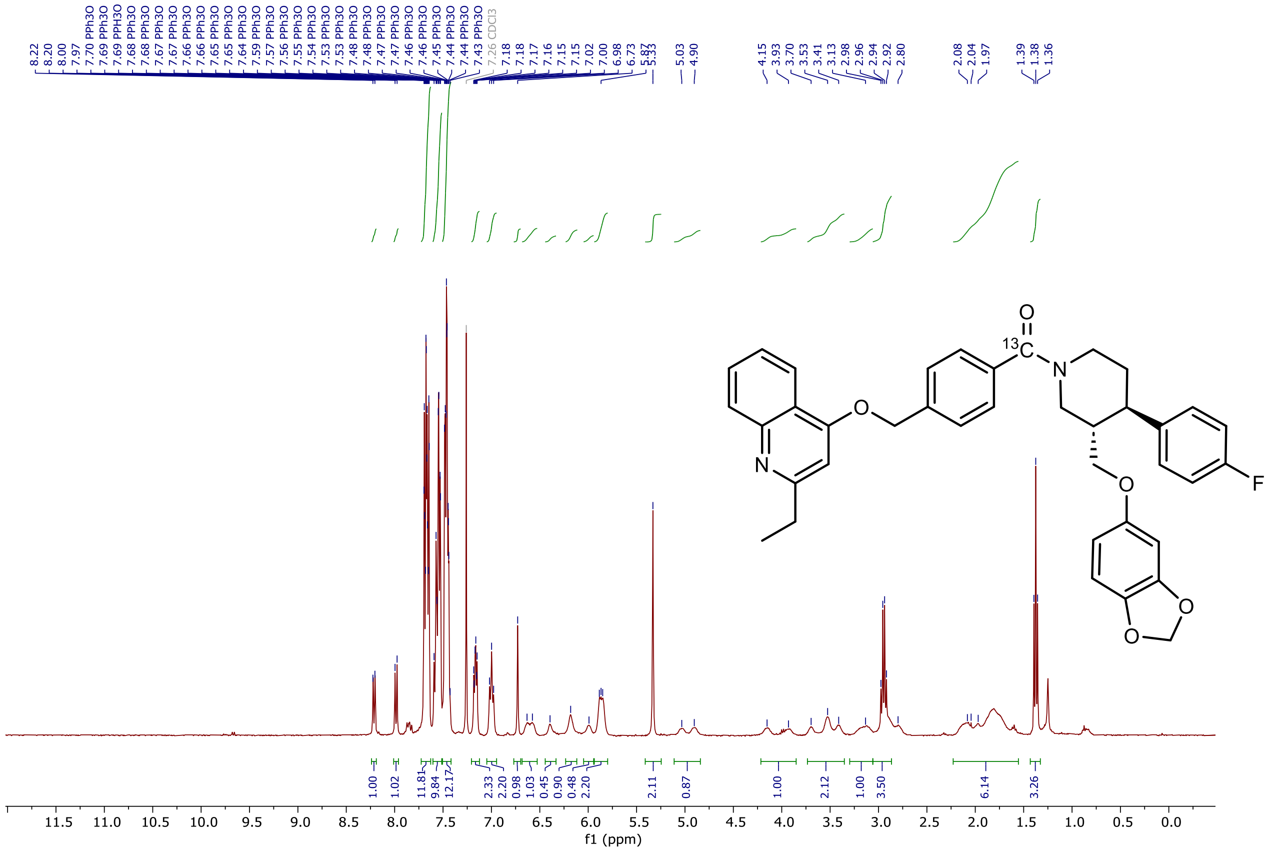

^{13}C-NMR

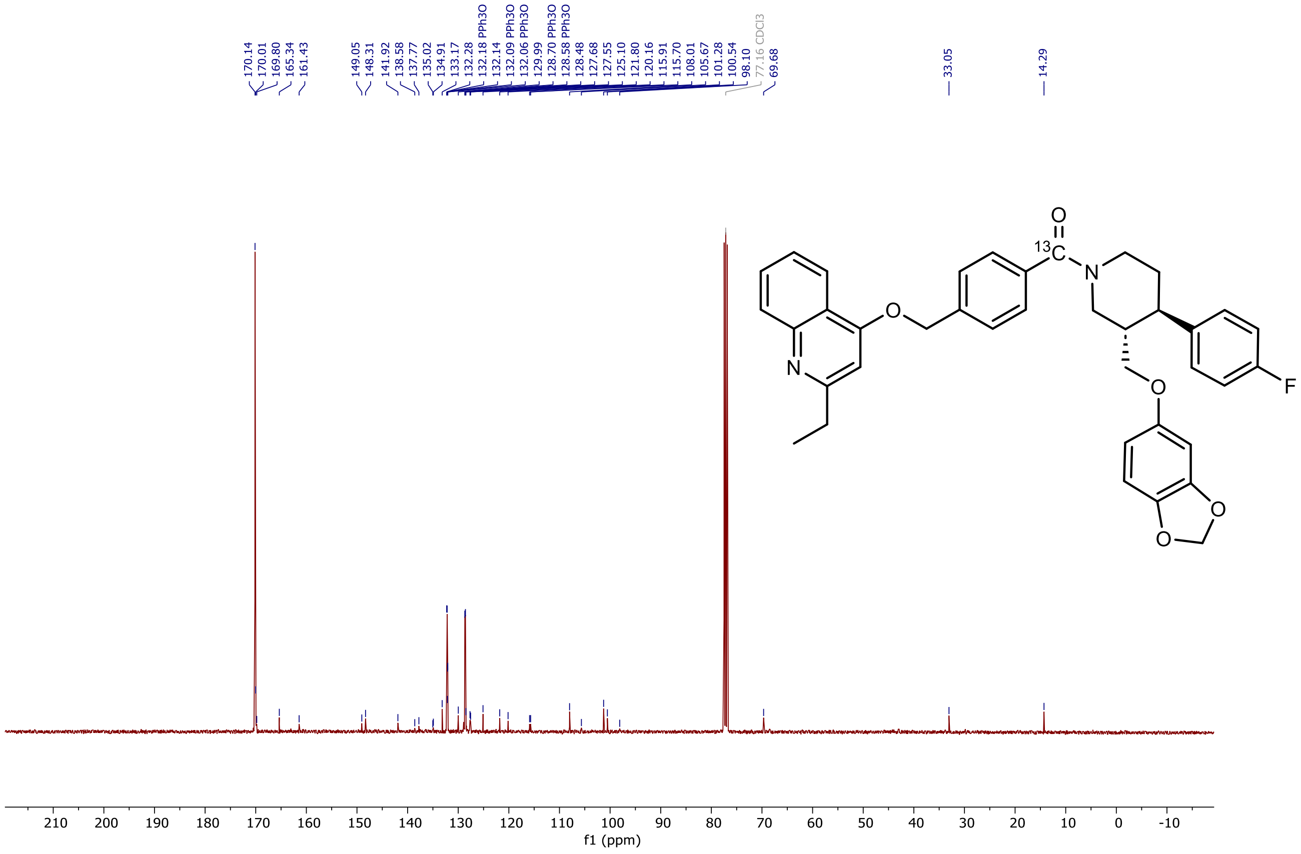

^{19}F-NMR

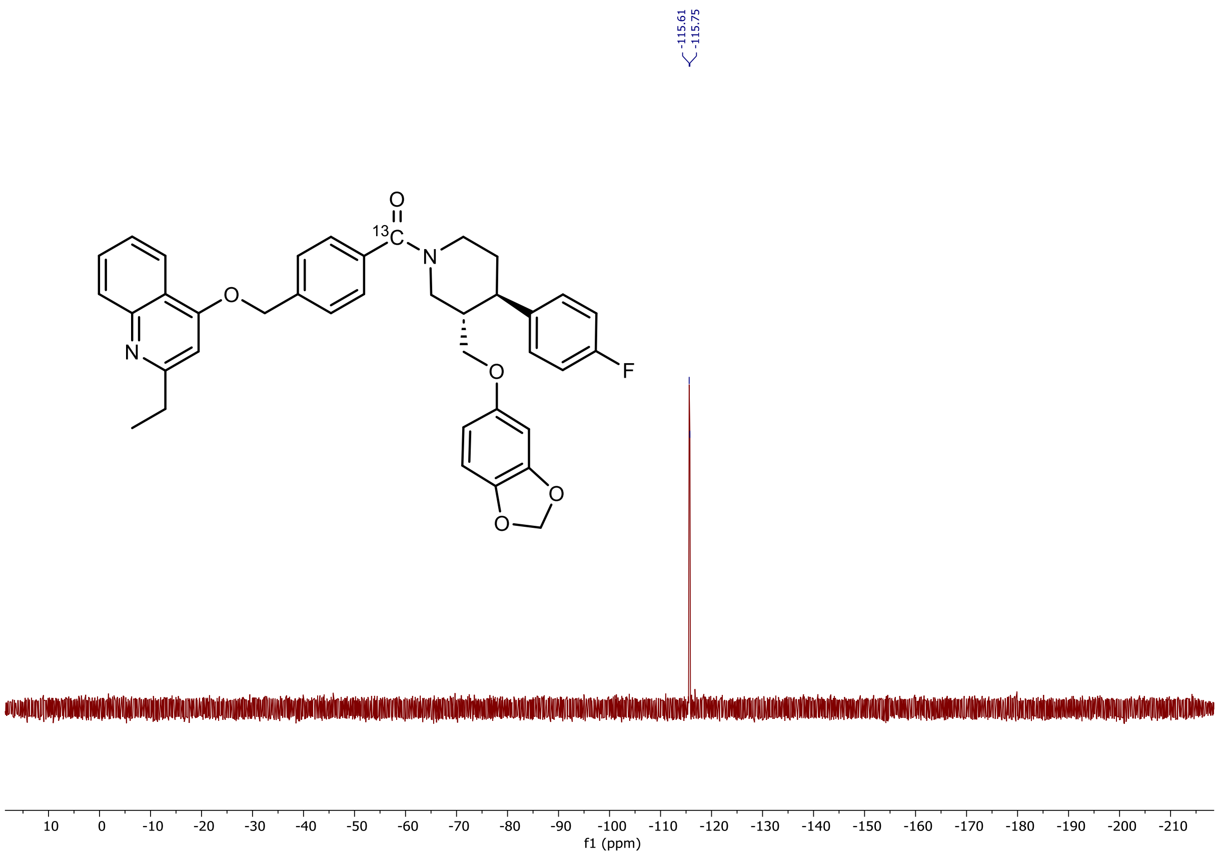

-115.61
-115.75
O
13C
N
O
N
F
O
O
O
10
0
-10
-20
-30
-40
-50
-60
-70
-80
-90
-100
-110
-120
-130
-140
-150
-160
-170
-180
-190
-200
-210
f1 (ppm)

[1,1'-biphenyl]-4-yl(4-(4-(4-fluorophenyl)-5-(2-methoxypyrimidin-4-yl)-1H-imidazol-1-yl)piperidin-1-yl)methanone ([12]C-13a)

^{1}H-NMR

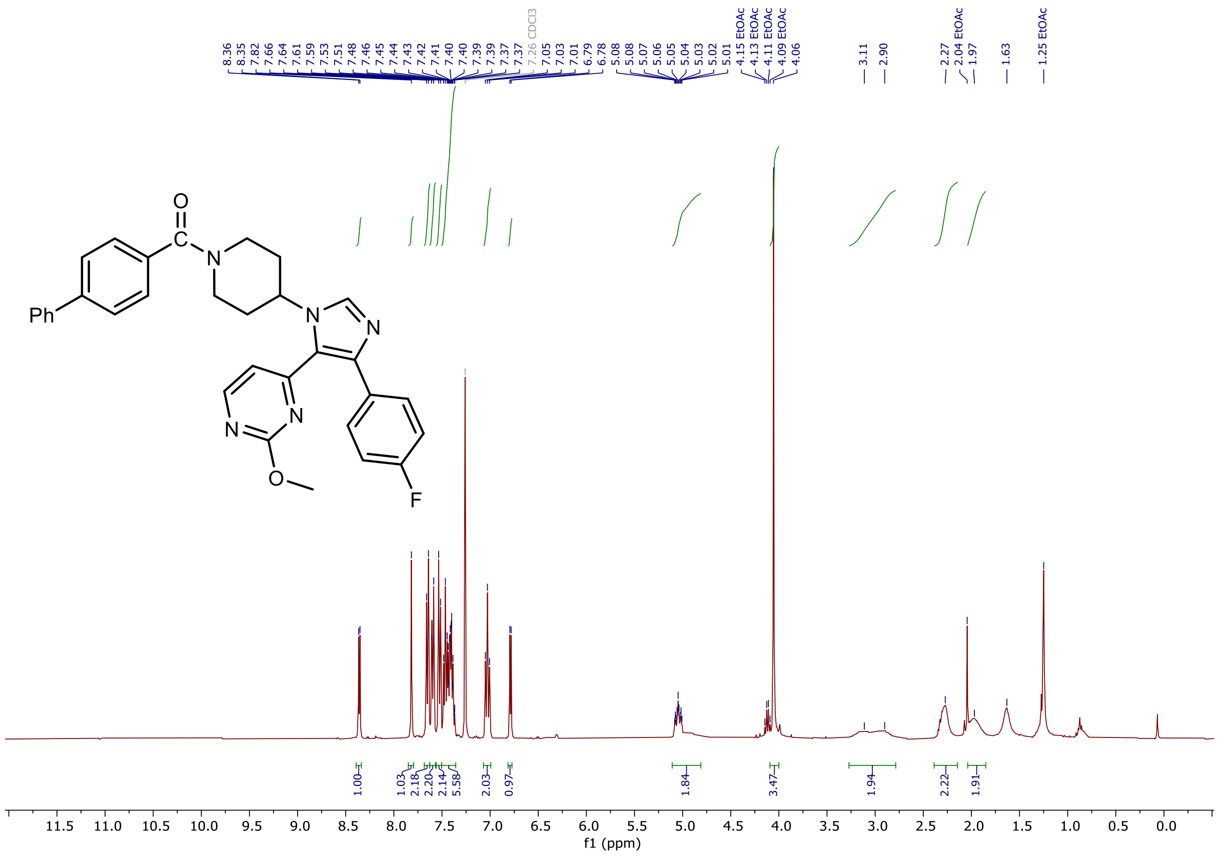

^{13}C-NMR

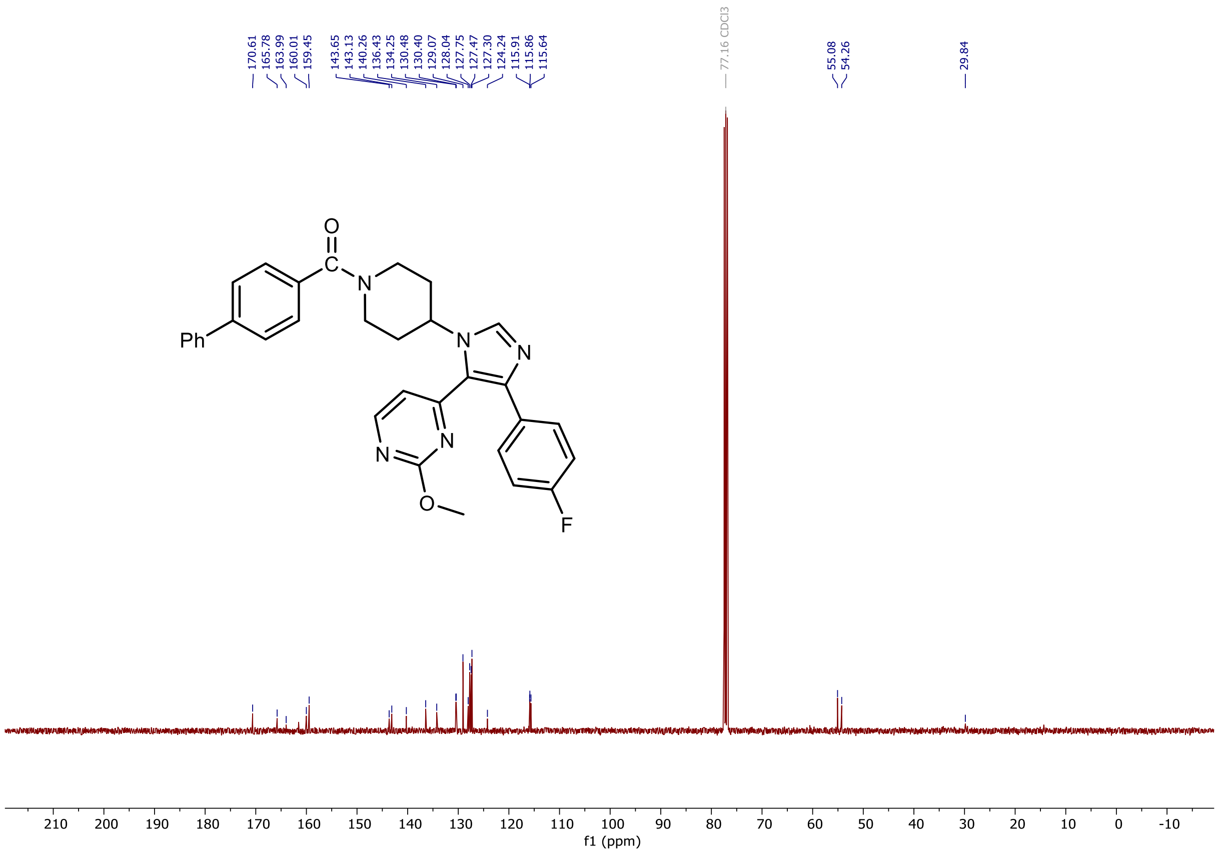

^{19}F-NMR

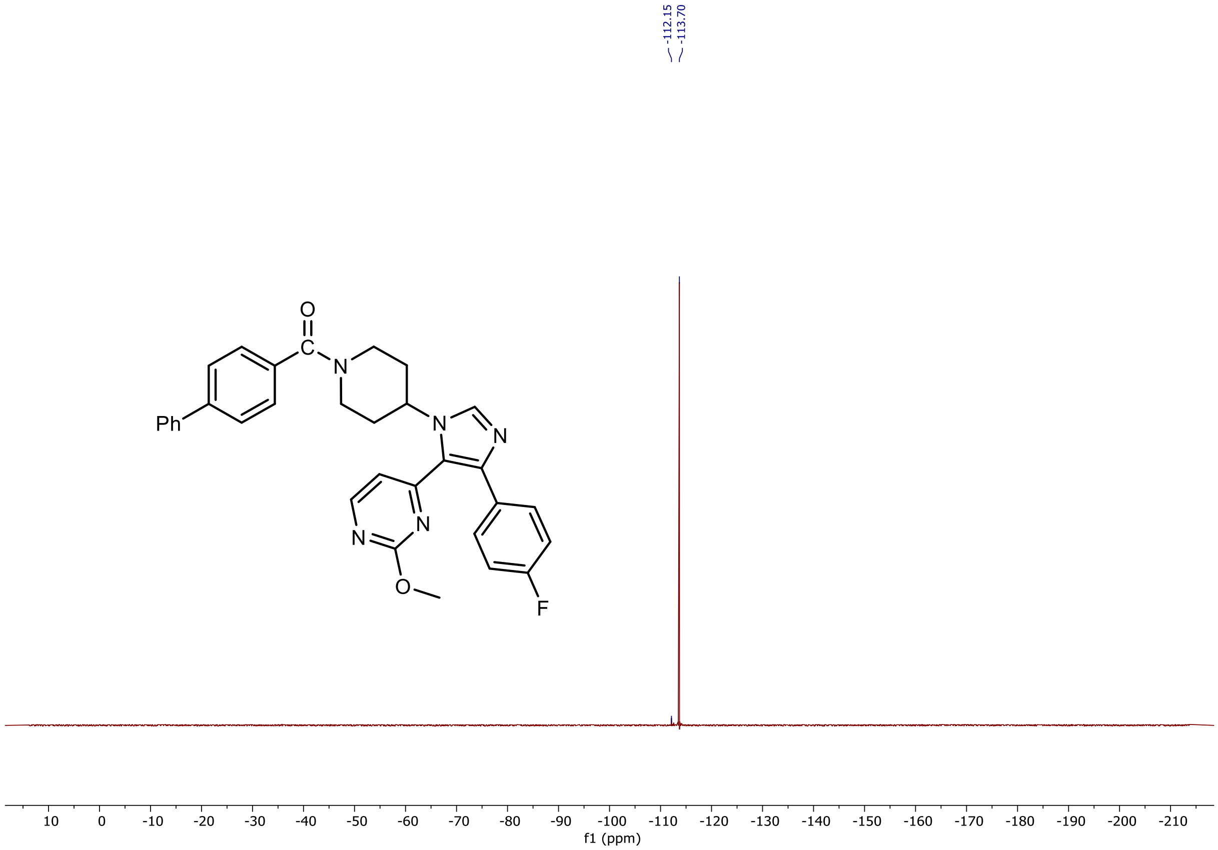

-112.15
-113.70
O
C
N
Ph
N
N
N
N
O
F
10 0 -10 -20 -30 -40 -50 -60 -70 -80 -90 -100 -110 -120 -130 -140 -150 -160 -170 -180 -190 -200 -210
f1 (ppm)

[1,1'-biphenyl]-4-yl(4-(4-(4-fluorophenyl)-5-(2-methoxypyrimidin-4-yl)-1H-imidazol-1-yl)piperidin-1-yl)-^{13}C-methanone (^{13}C-13a)

^{1}H-NMR

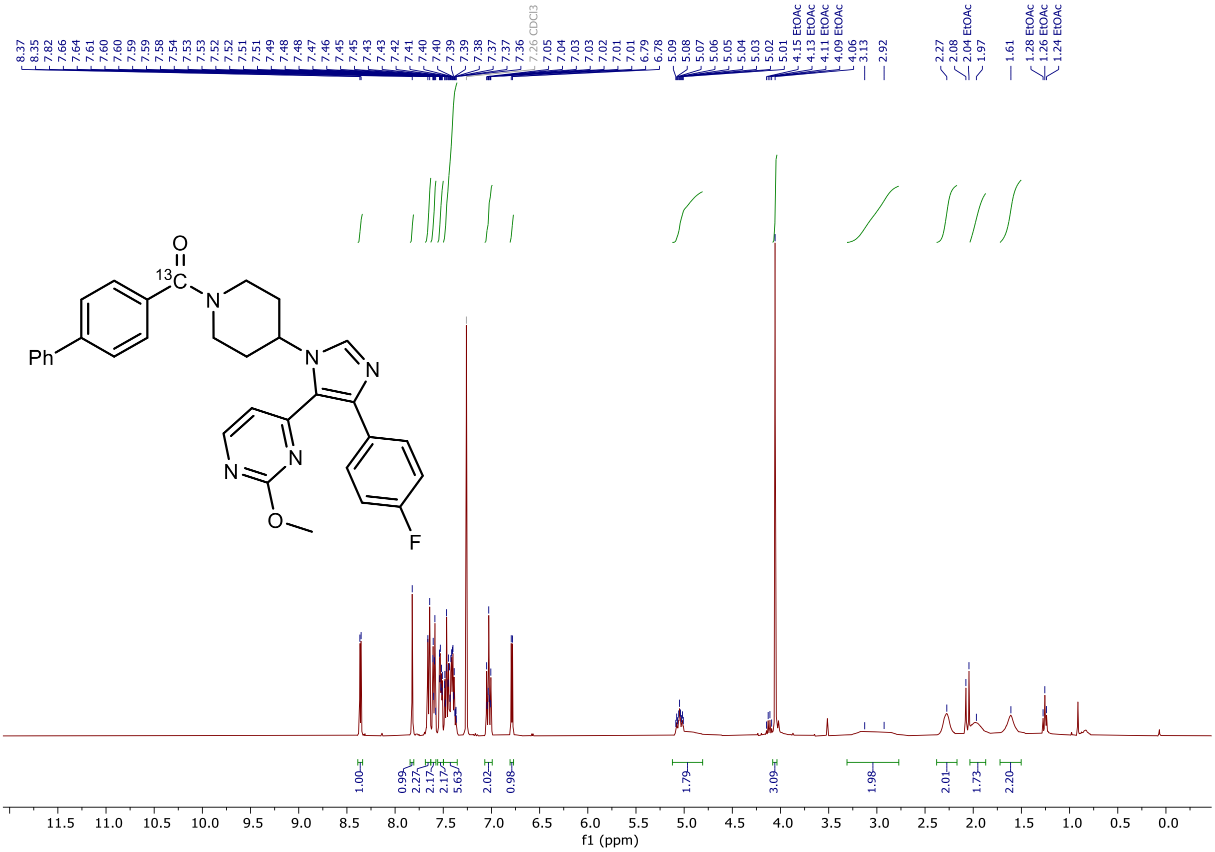

^{13}C-NMR

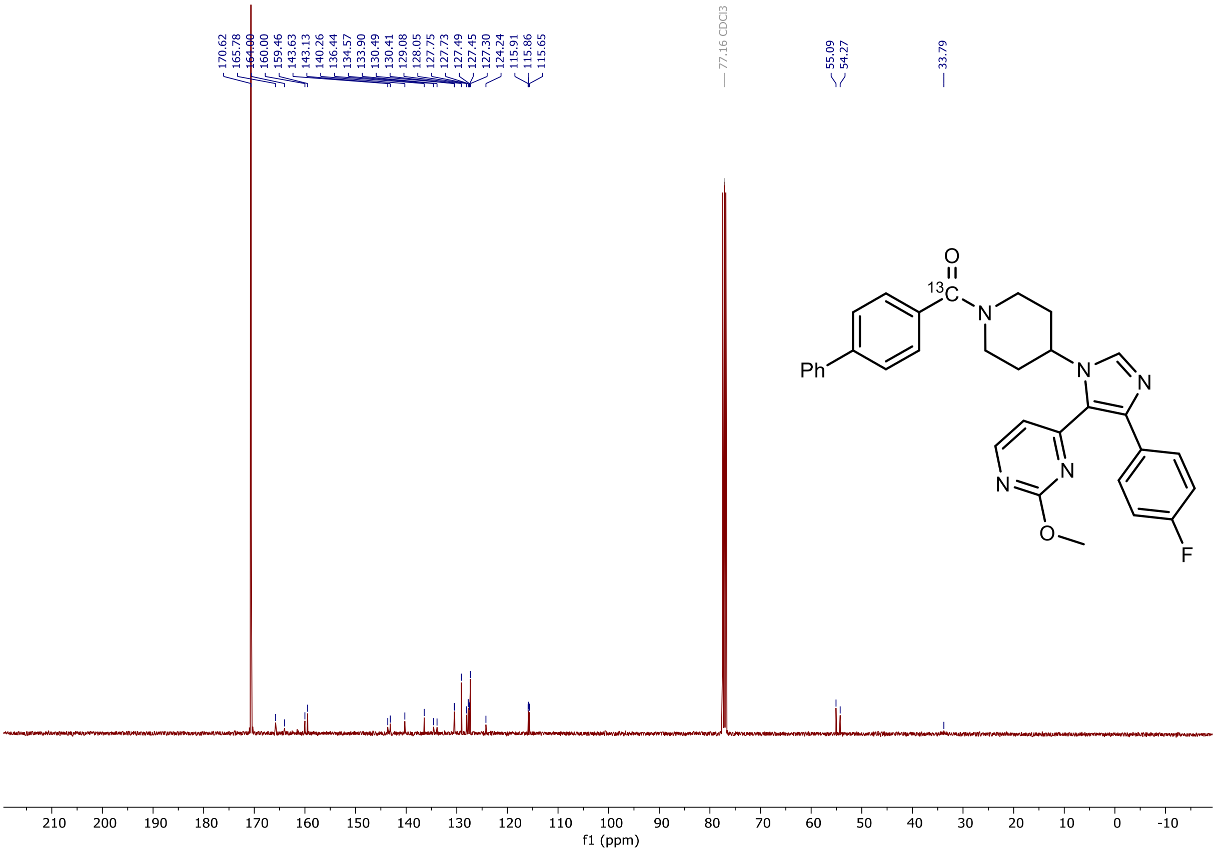

^{19}F-NMR

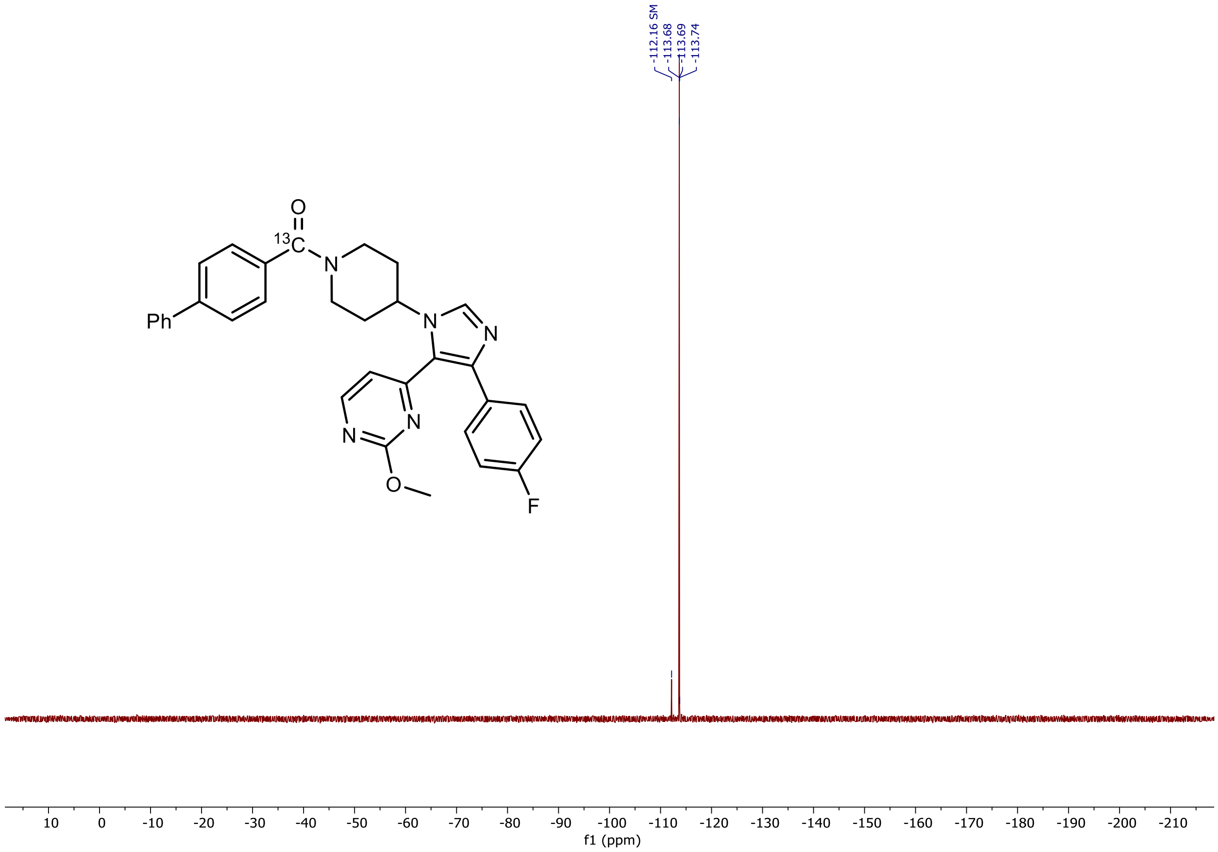
-112.16 SM
-113.68
-113.69
-113.74
10
0
-10
-20
-30
-40
-50
-60
-70
-80
-90
-100
-110
-120
-130
-140
-150
-160
-170
-180
-190
-200
-210
f1 (ppm)

[1,1'-biphenyl]-4-yl(4-(4-(4-fluorophenyl)-5-(2-methoxypyrimidin-4-yl)-1*H*-imidazol-1-yl)piperidin-1-yl)-^{14}C-methanone (^{14}C-13a)

^{1}H-NMR

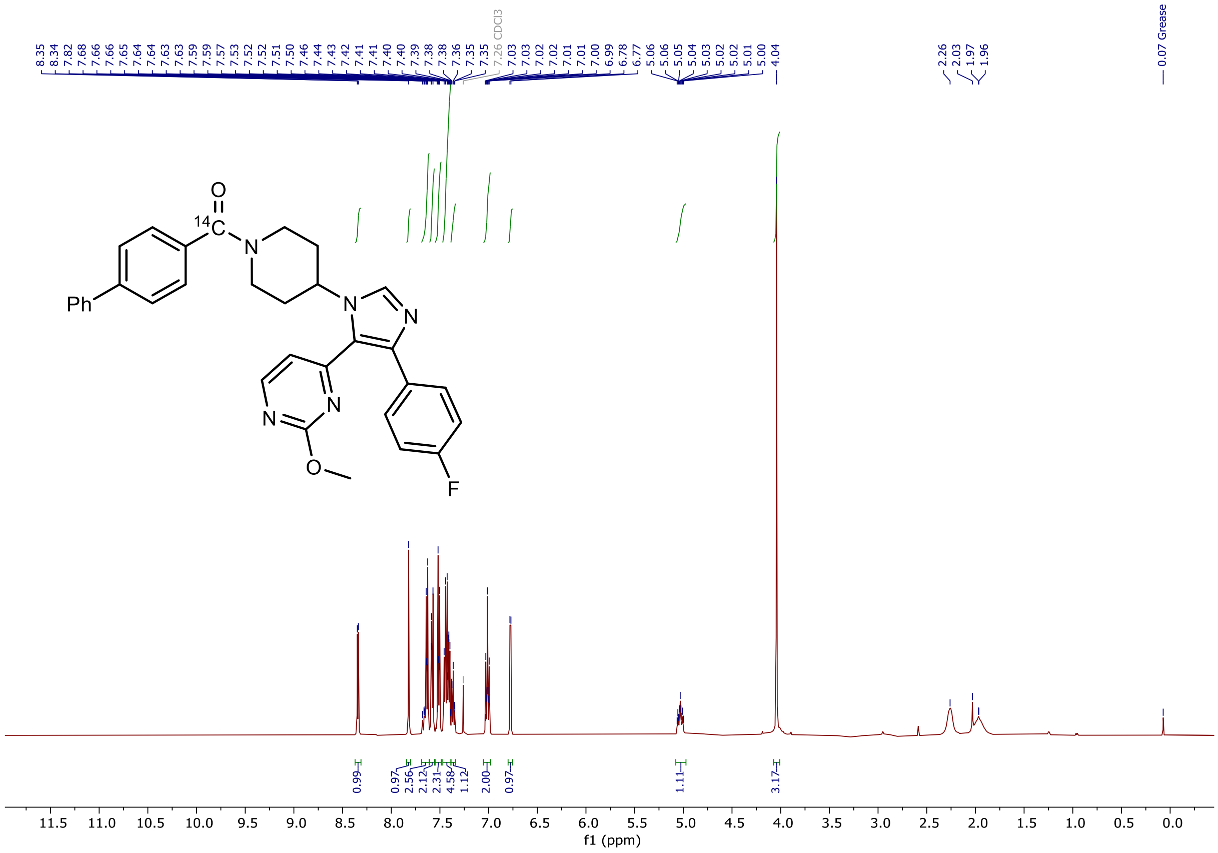

^{13}C-NMR

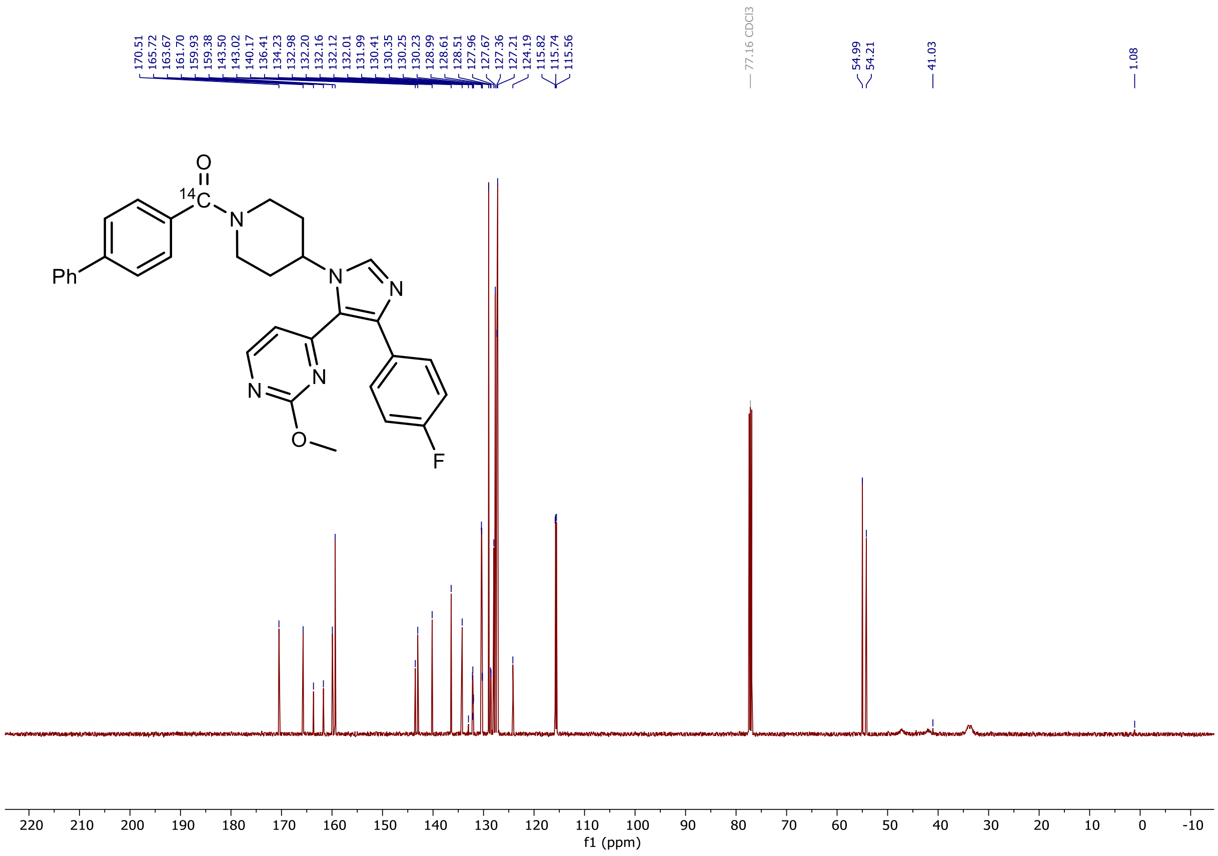

[1,2,4]triazolo[1,5-a]pyridin-6-yl(4-(4-(4-fluorophenyl)-5-(2-methoxypyrimidin-4-yl)-1*H*-imidazol-1-yl)piperidin-1-yl)-methanone (^{12}C-13s)

^{1}H-NMR

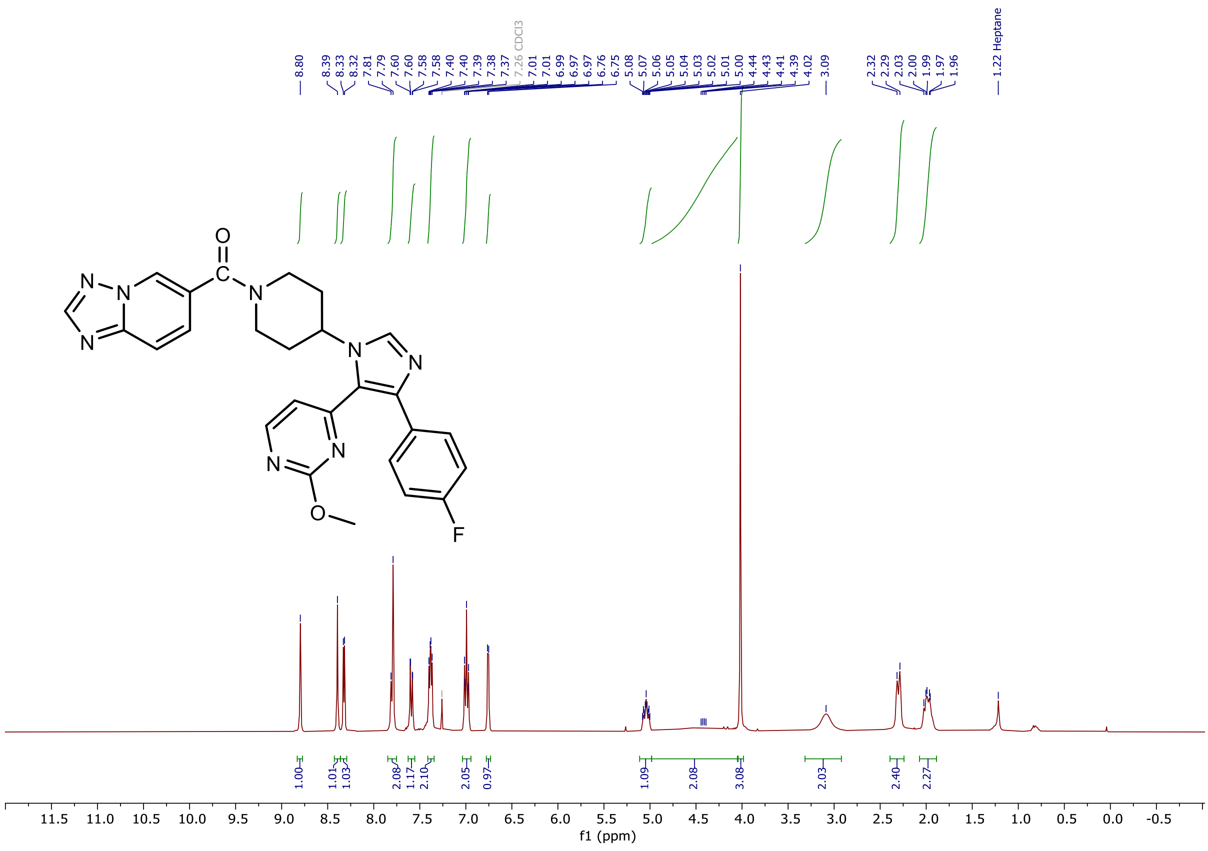

^{13}C-NMR

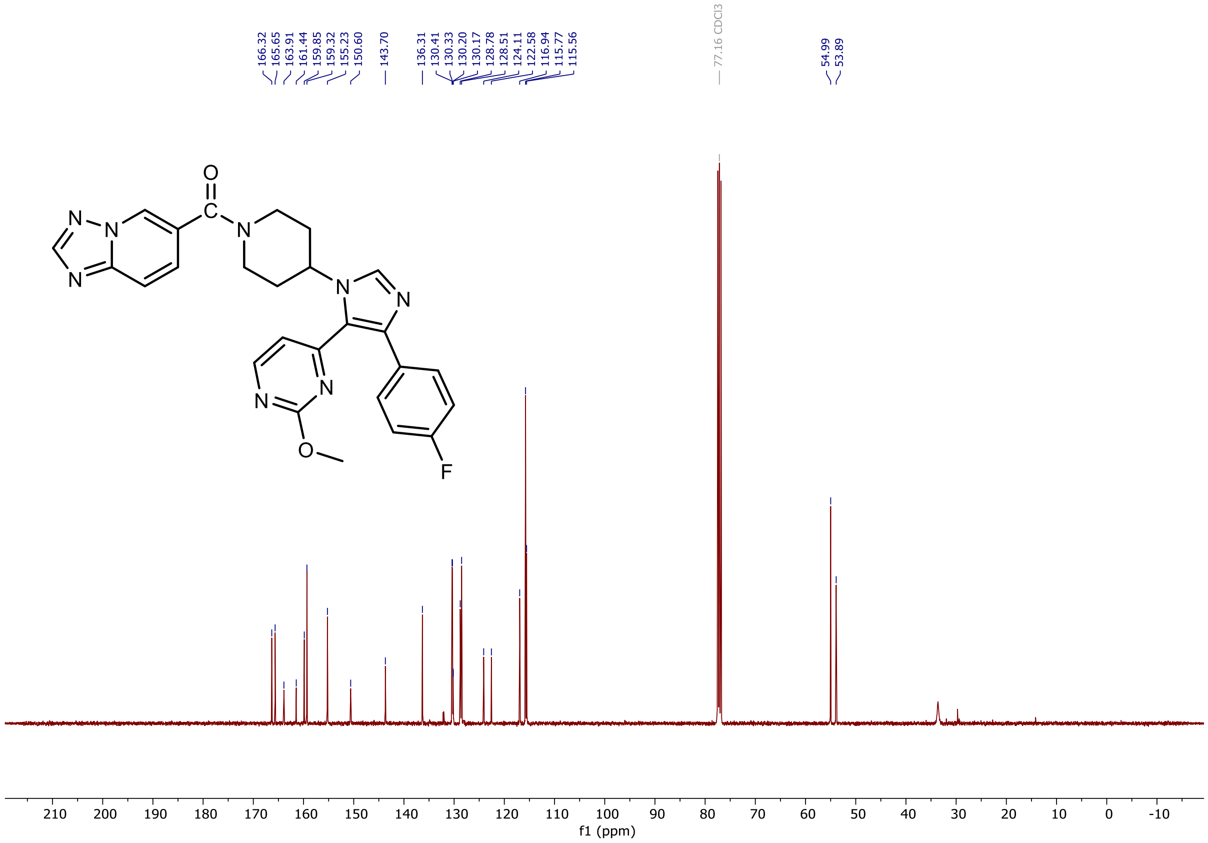

^{19}F-NMR

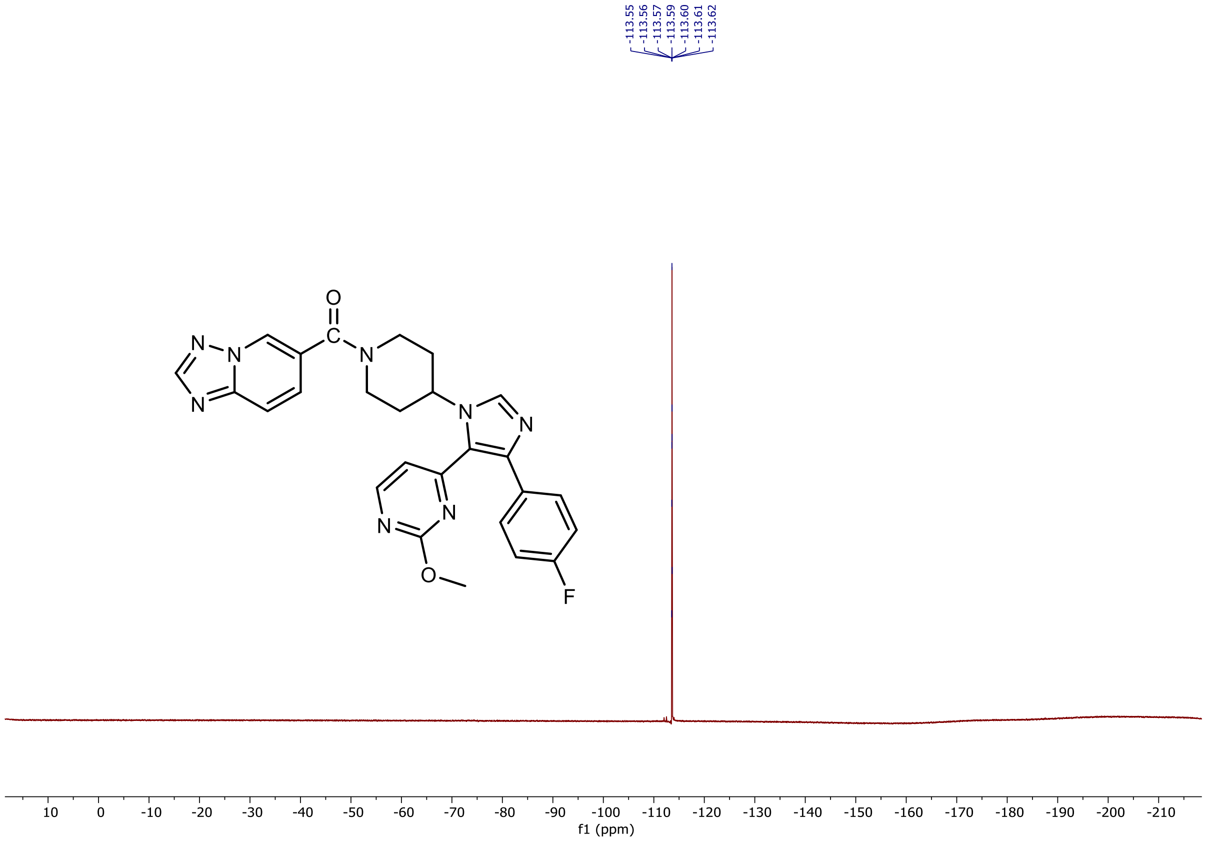

-113.55
-113.56
-113.57
-113.59
-113.60
-113.61
-113.62
10
0
-10
-20
-30
-40
-50
-60
-70
-80
-90
-100
-110
-120
-130
-140
-150
-160
-170
-180
-190
-200
-210
f1 (ppm)

[1,2,4]triazolo[1,5-a]pyridin-6-yl(4-(4-(4-fluorophenyl)-5-(2-methoxypyrimidin-4-yl)-1*H*-imidazol-1-yl)piperidin-1-yl)-^{13}C-methanone (^{13}C-13s)

^{1}H-NMR

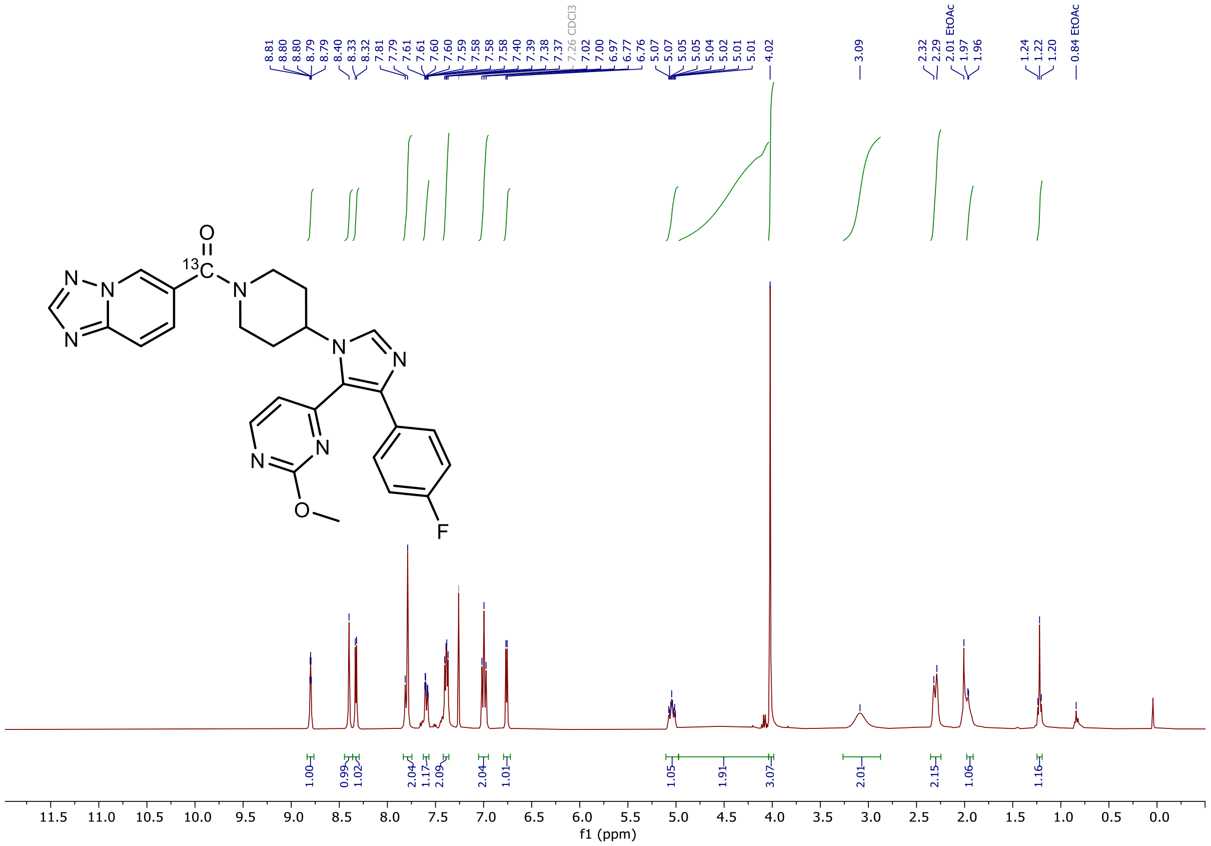

^{13}C-NMR

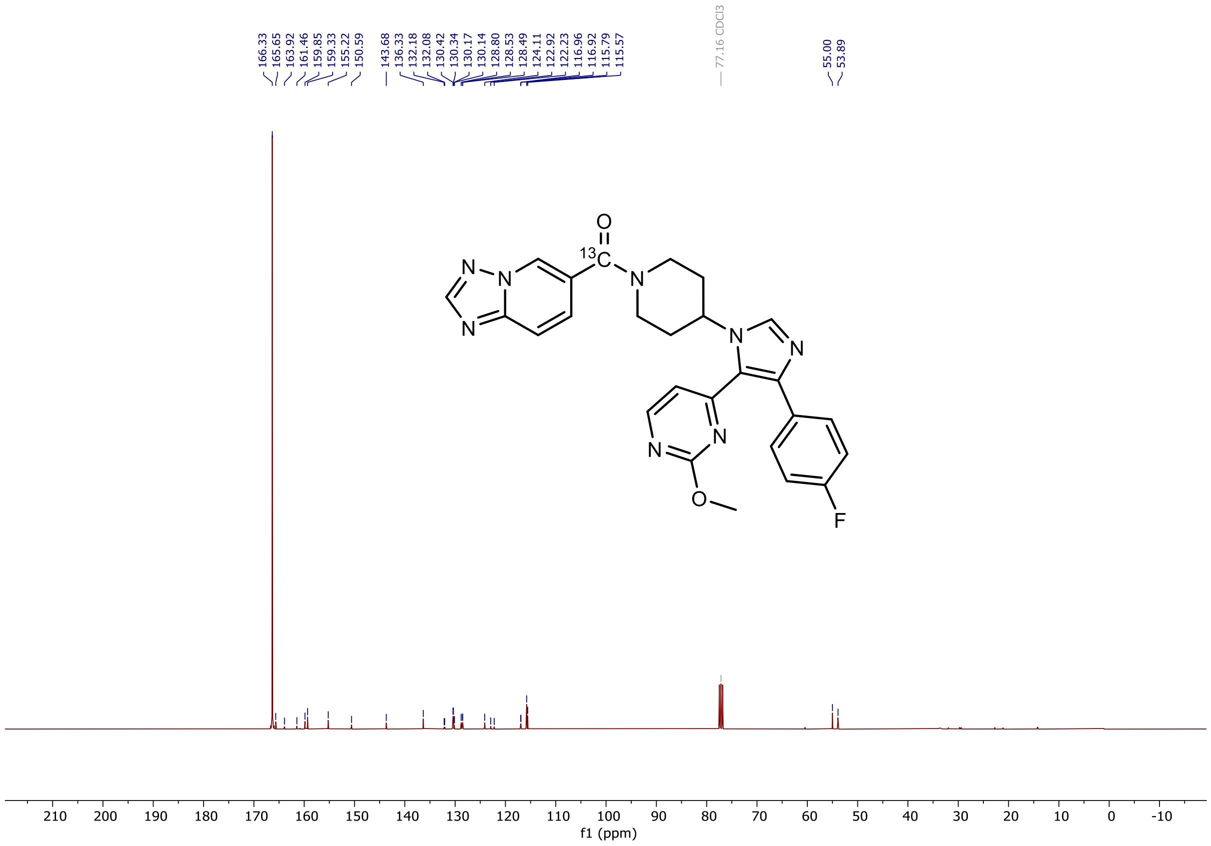

^{19}F-NMR

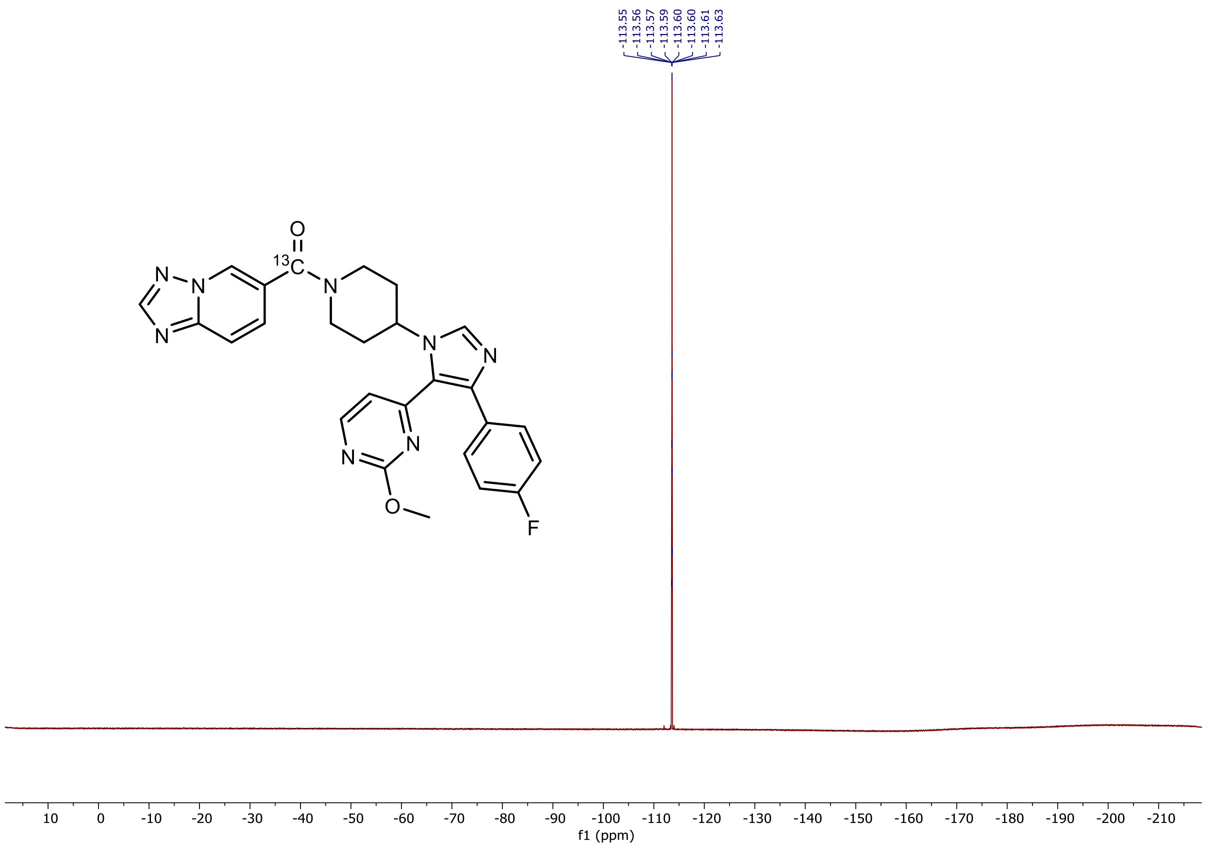
-113.55
-113.56
-113.57
-113.59
-113.60
-113.60
-113.61
-113.63
10 0 -10 -20 -30 -40 -50 -60 -70 -80 -90 -100 -110 -120 -130 -140 -150 -160 -170 -180 -190 -200 -210
f1 (ppm)

[1,2,4]triazolo[1,5-a]pyridin-6-yl(4-(4-(4-fluorophenyl)-5-(2-methoxypyrimidin-4-yl)-1*H*-imidazol-1-yl)piperidin-1-yl)-^{14}C-methanone (^{14}C-13s)

^{1}H-NMR

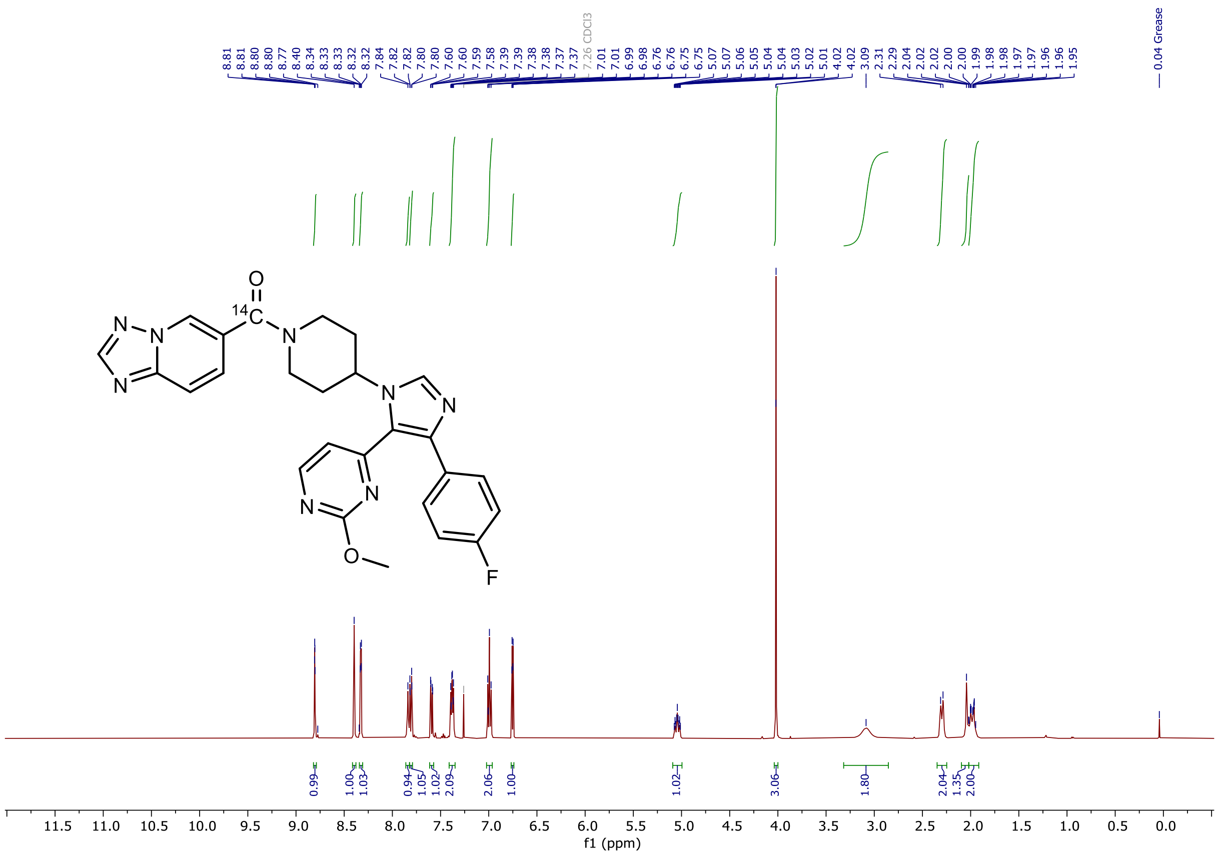

^{13}C-NMR

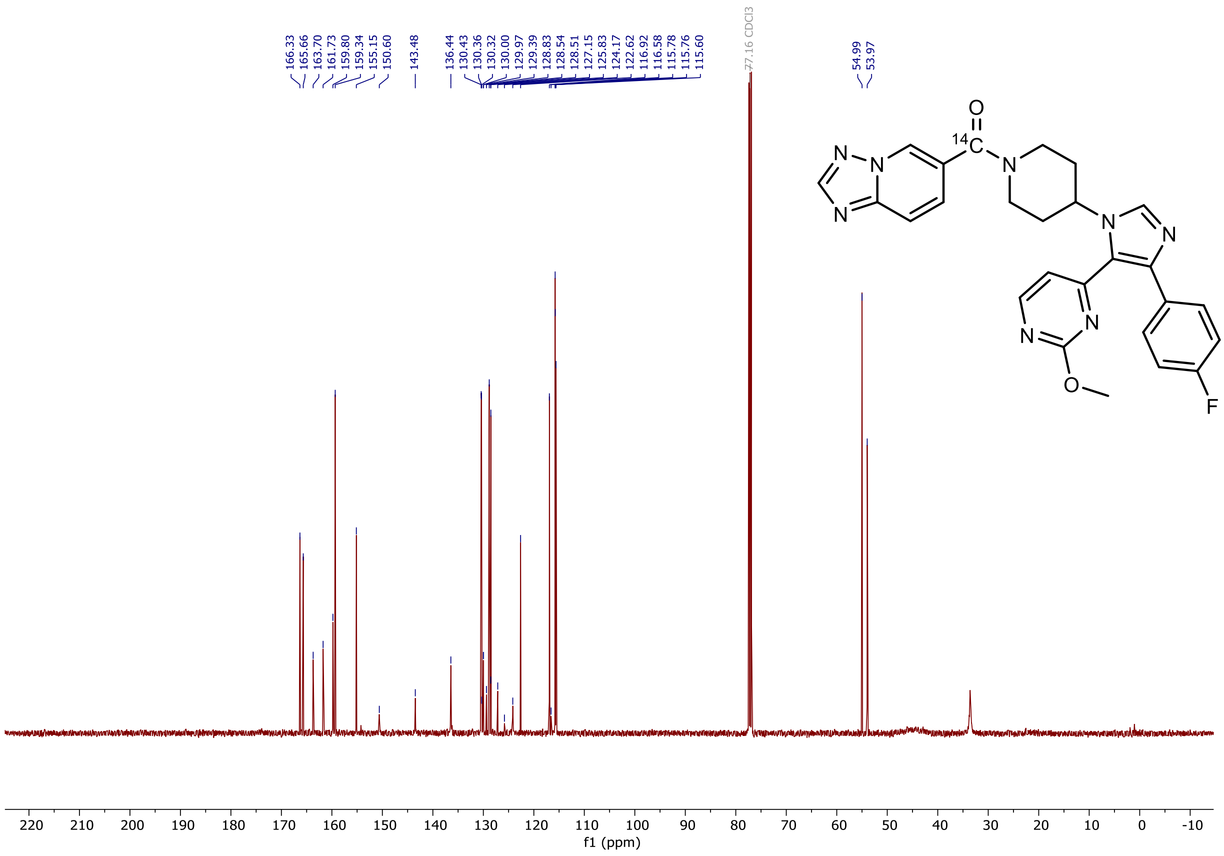

8.1 Additional spectra

***trans*-Chloro(2-morpholinoethan-1-amine-carbonyl)bis(triphenylphosphine) palladium(II) (Pd-9)**

^{1}H-NMR

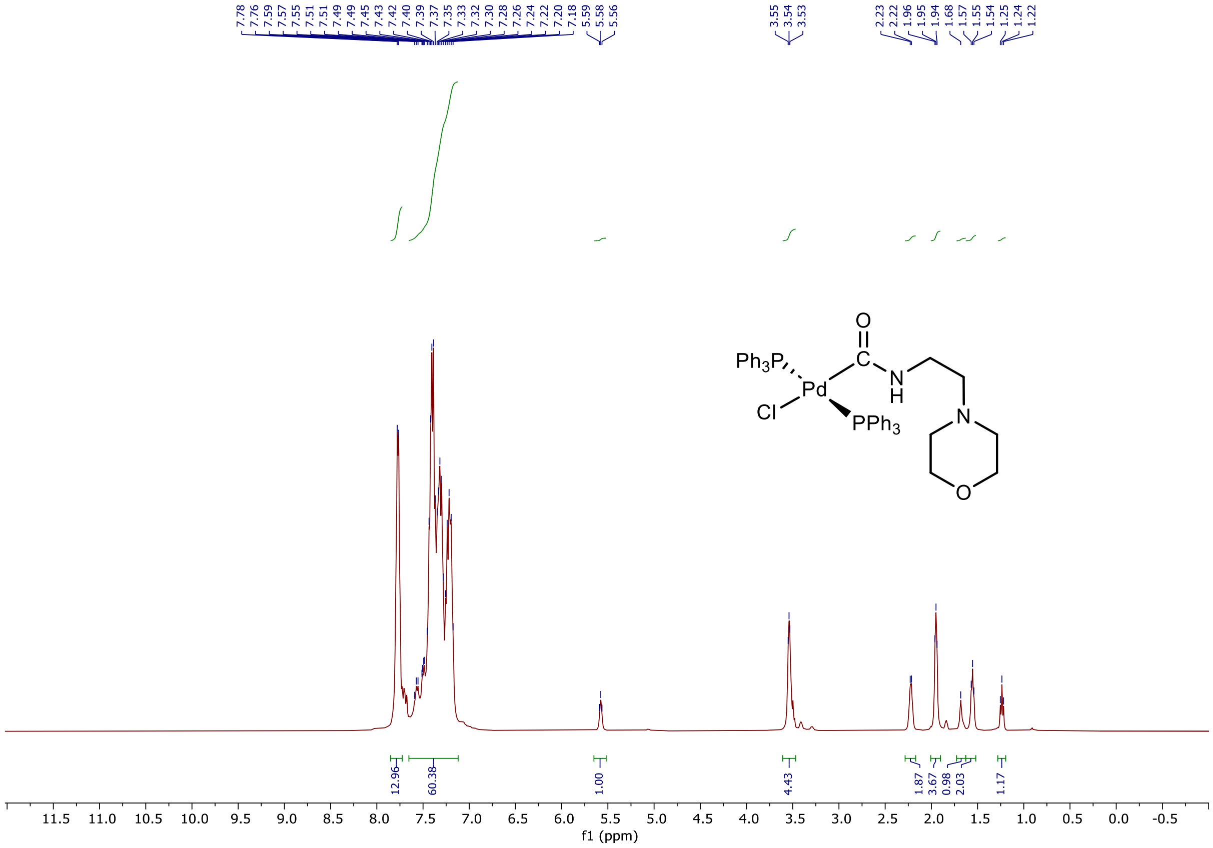

^{13}C-NMR

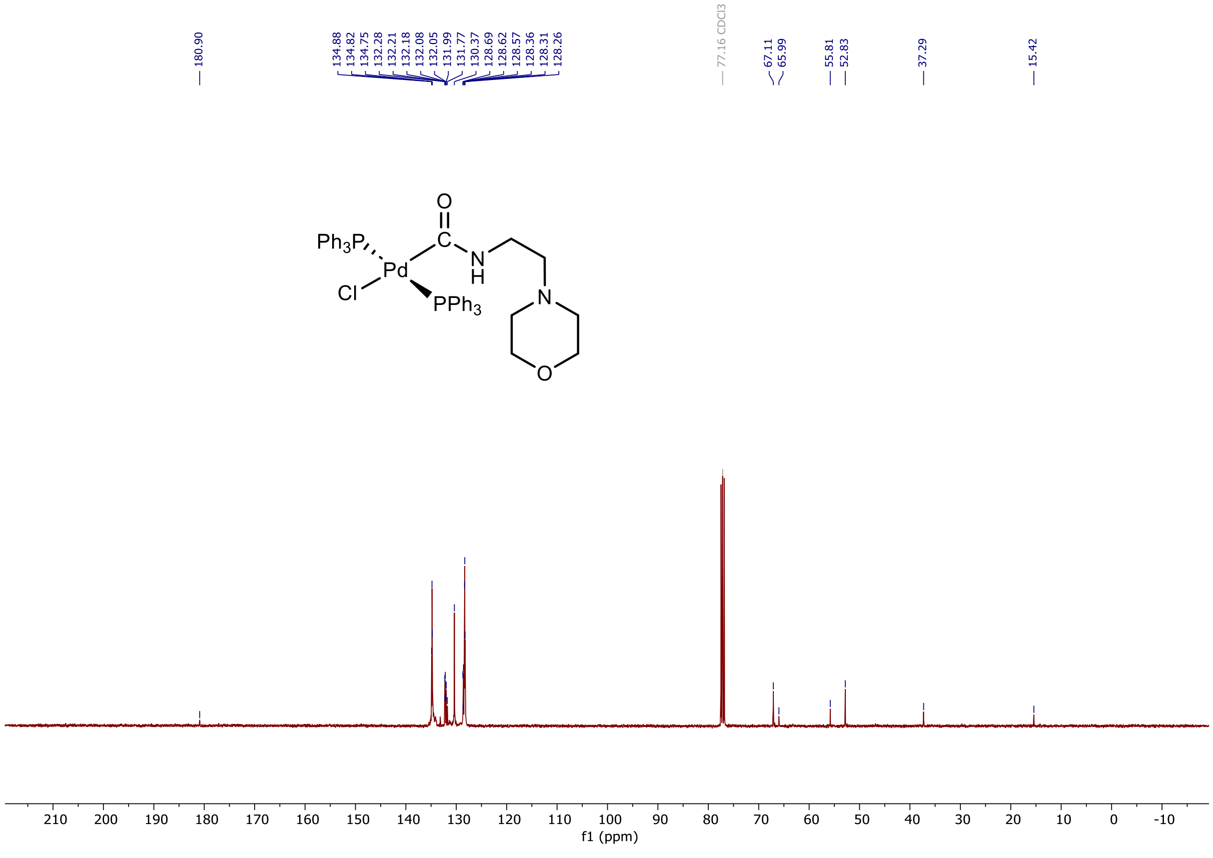

^{31}P-NMR

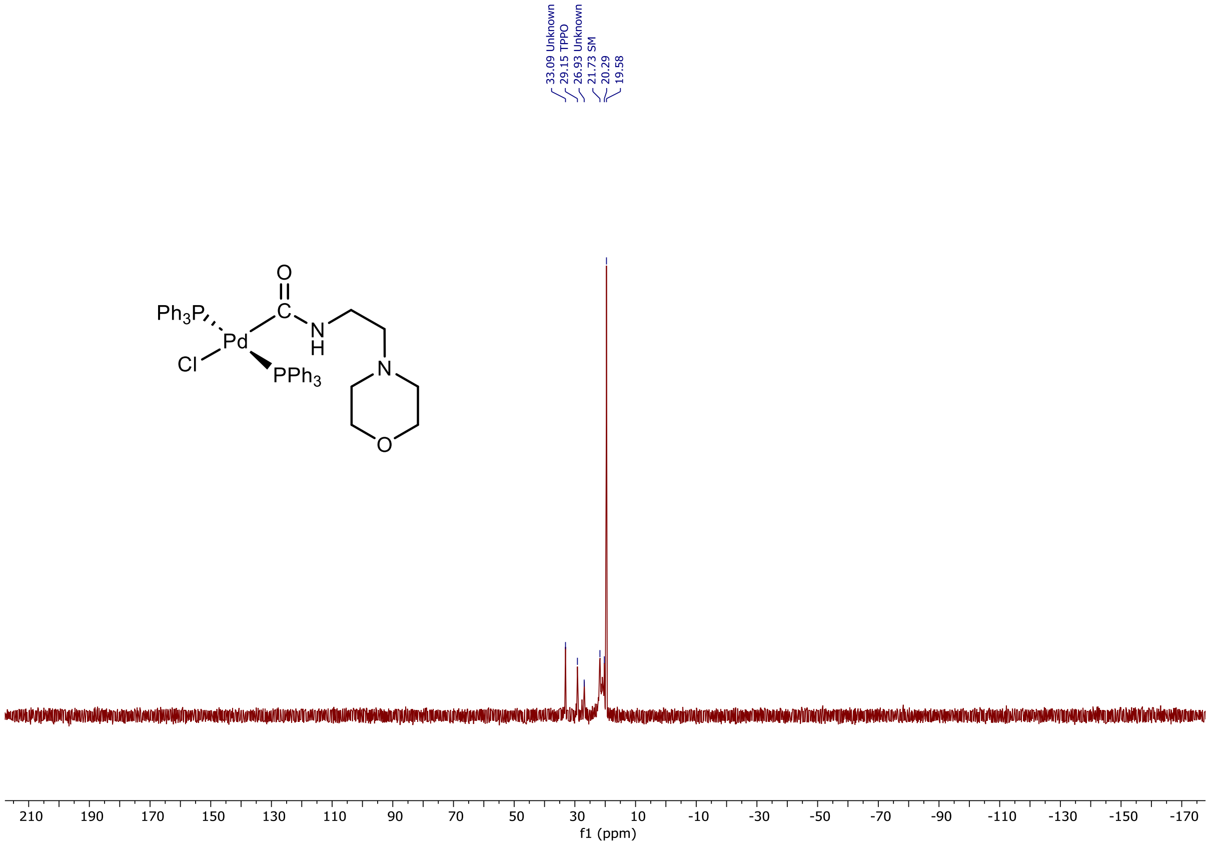
Ph3P
Pd
Cl
PPh3
C
O
N
H
N
O
33.09 Unknown
29.15 TPPO
26.93 Unknown
21.73 SM
20.29
19.58
210
190
170
150
130
110
90
70
50
30
10
-10
-30
-50
-70
-90
-110
-130
-150
-170
f1 (ppm)

trans-Chloro(1-ethynylpiperazine-carbonyl)bis(triphenylphosphine) palladium(II) (Pd-16)

^{1}H-NMR

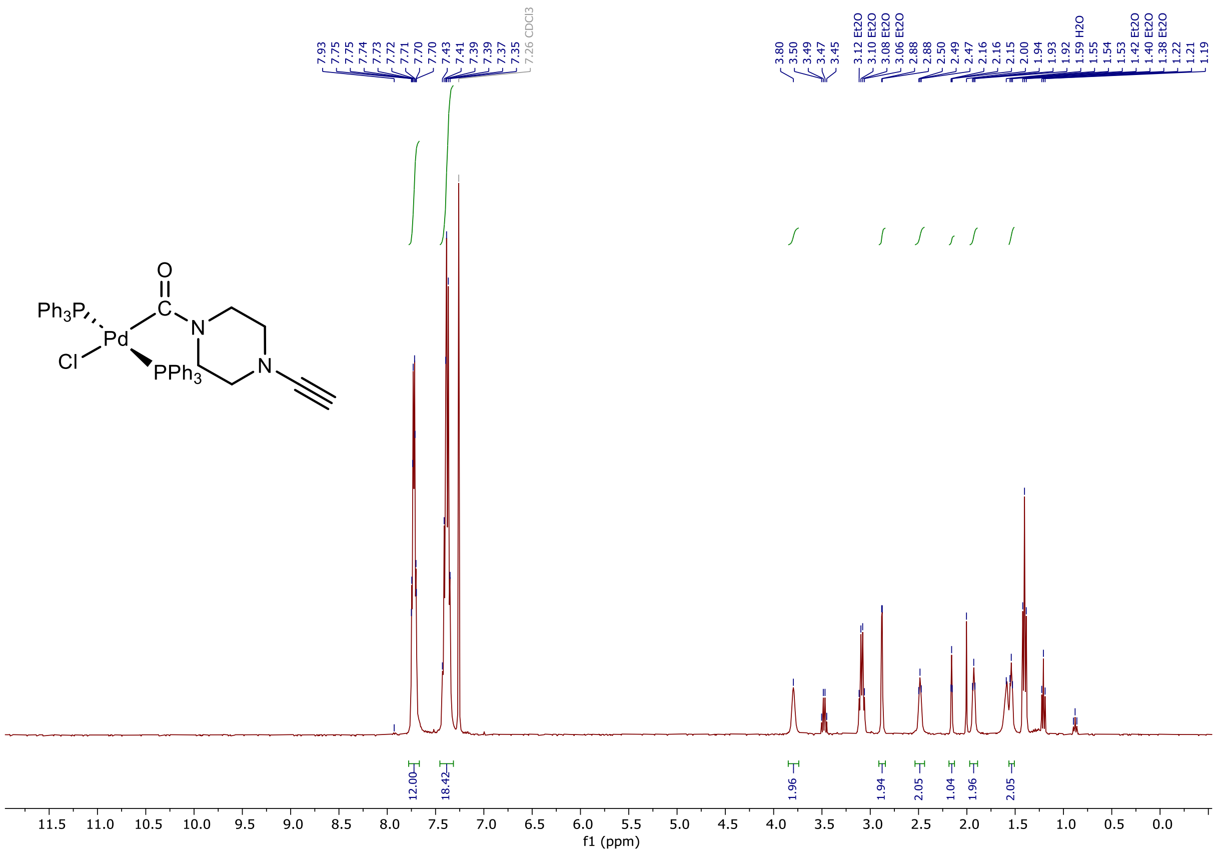

^{31}P-NMR

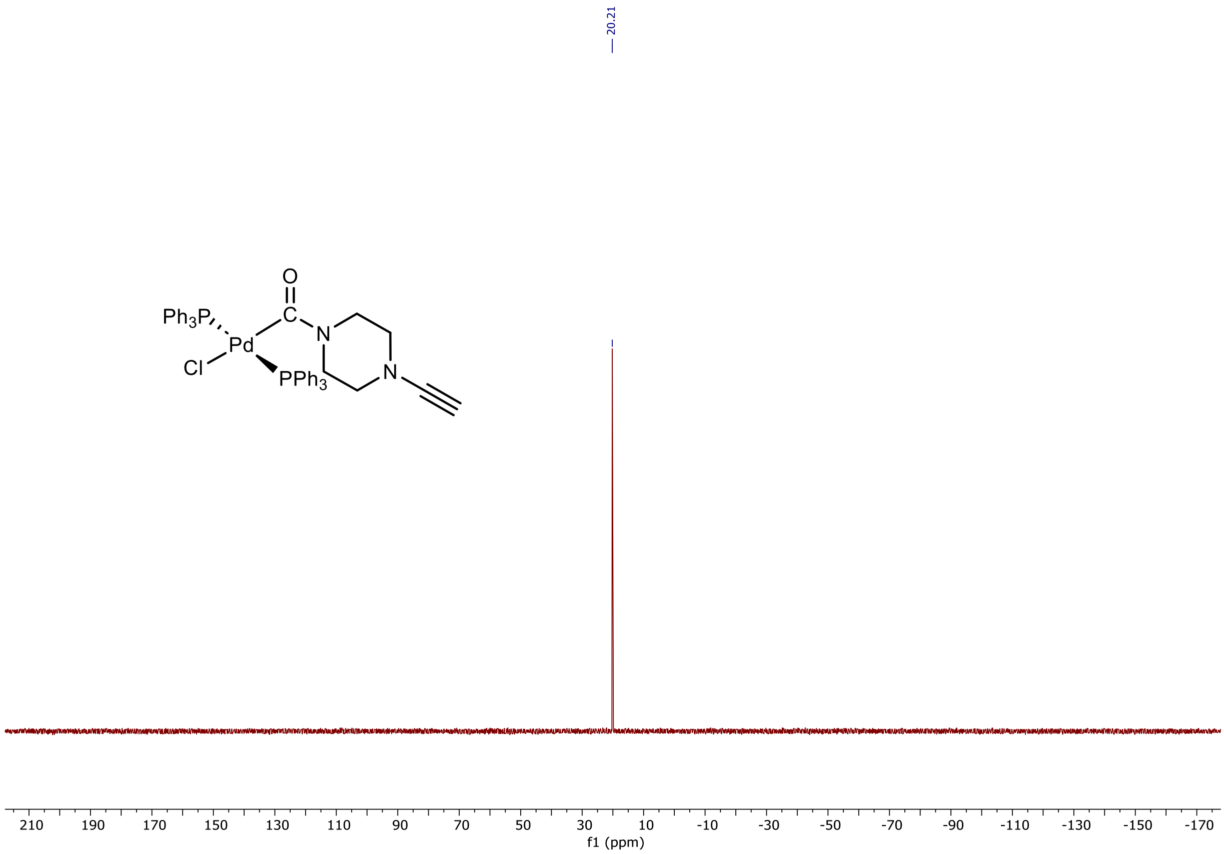

***trans*-Chloro(8-fluoro-5-(4-((methylamino)methyl)phenyl)-2,3,4,6-tetrahydro-1*H*-azepino[5,4,3-*cd*]indol-1-one -carbonyl)bis(triphenylphosphine) palladium(II) (Pd-17)**

^{1}H-NMR

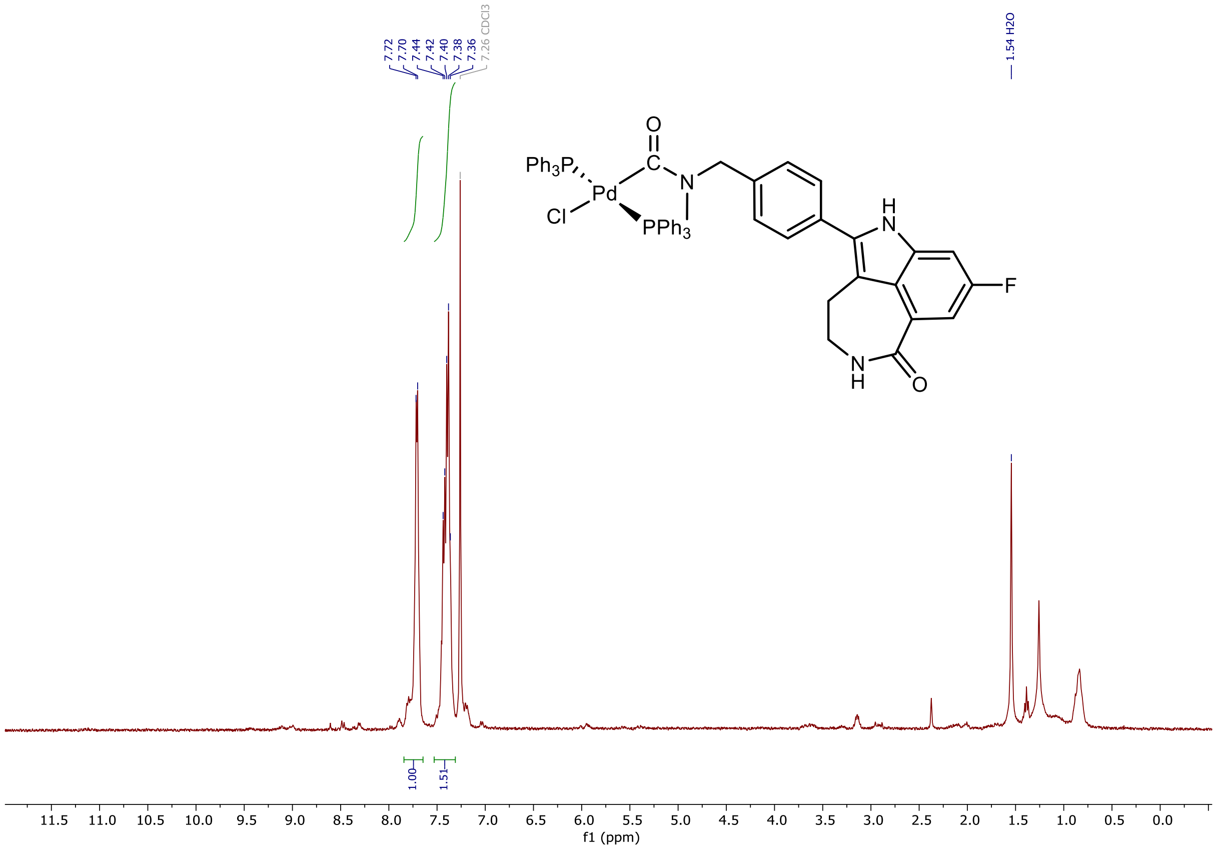

^{31}P-NMR

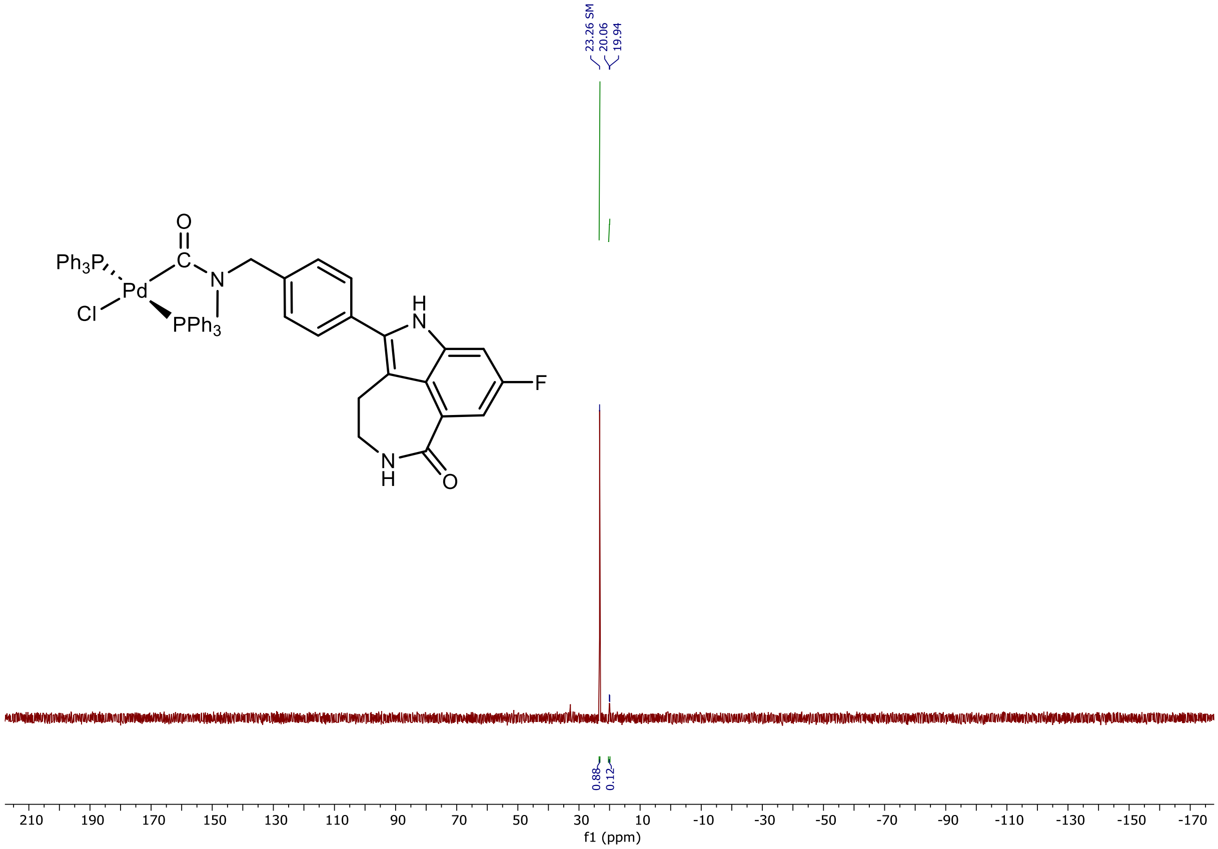

trans-Chloro(1-ethynylpiperazine-carbonyl)bis(triphenylphosphine) palladium(II) (Pd-18)

^{1}H-NMR

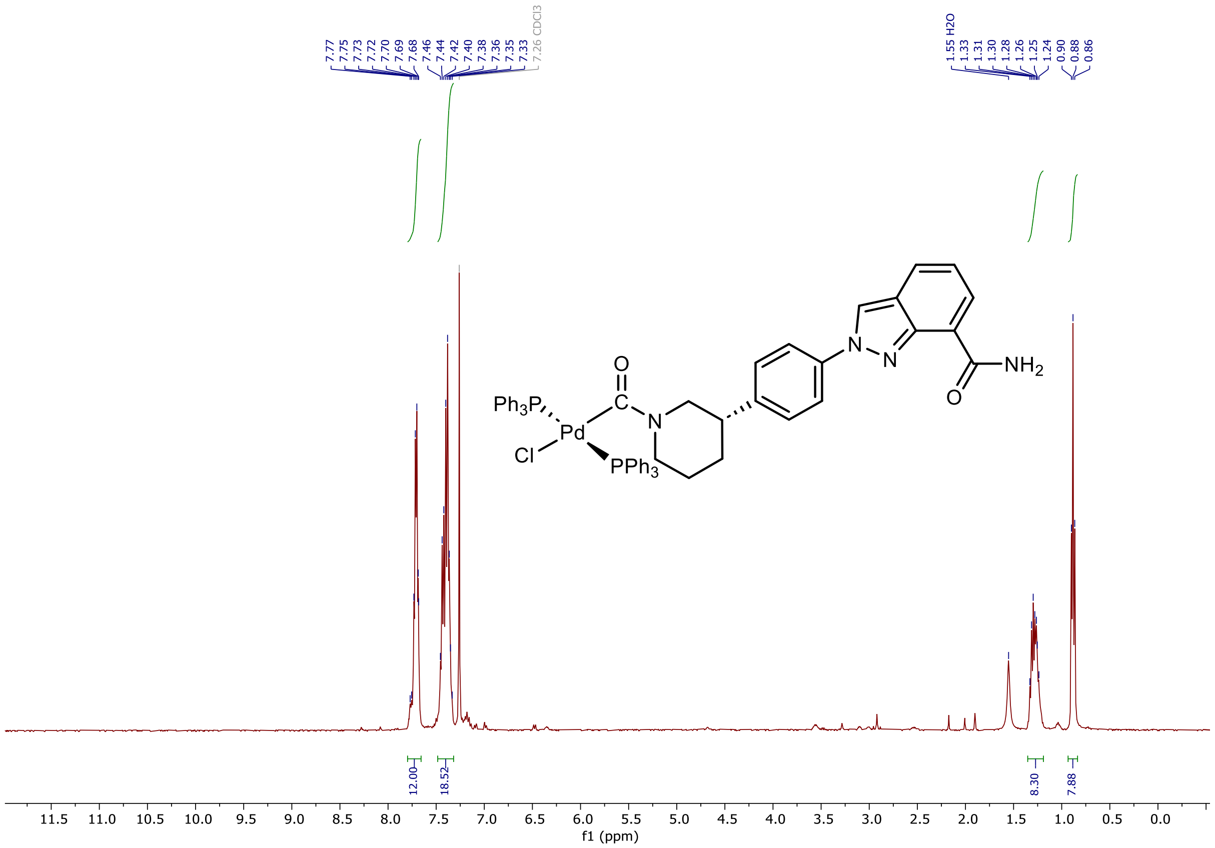

^{31}P-NMR

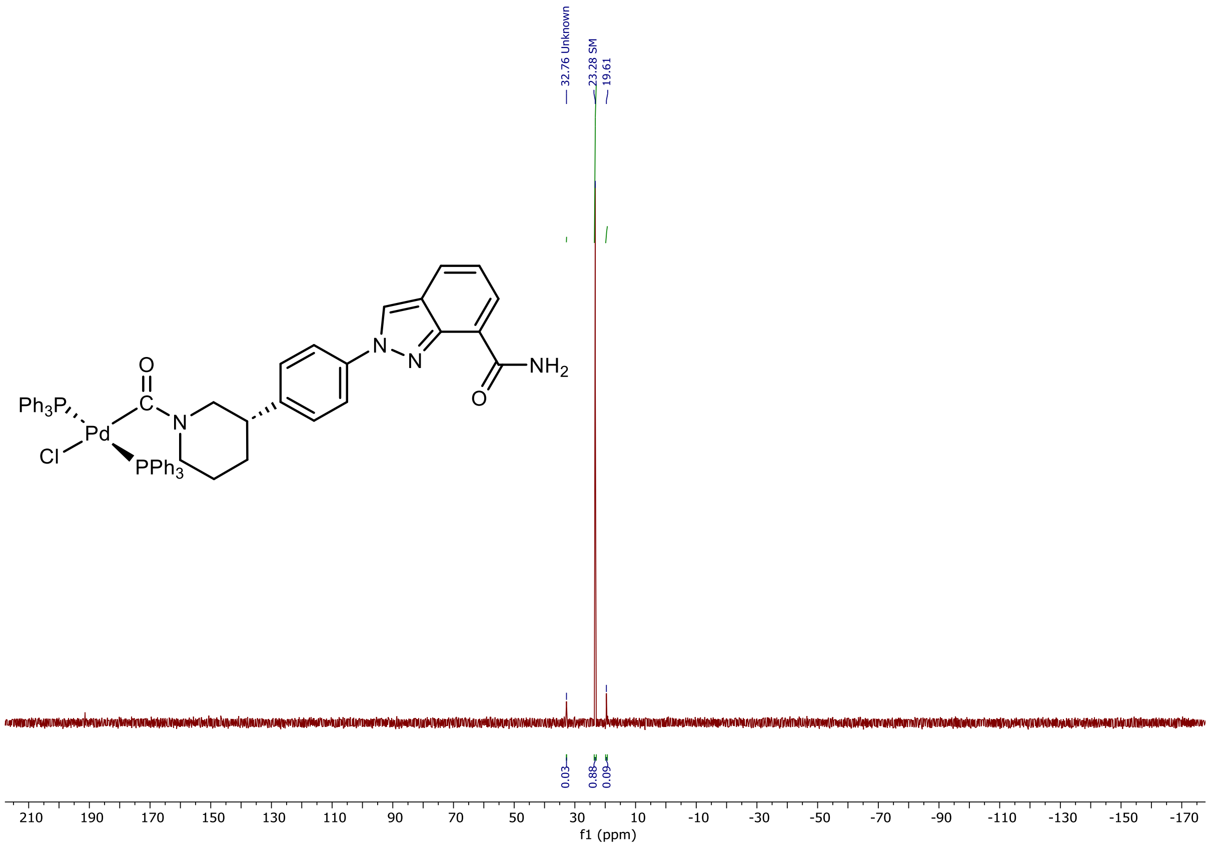

trans-Chloro(4-aniline-carbonyl)bis(triphenylphosphine) palladium(II) (Pd-19)

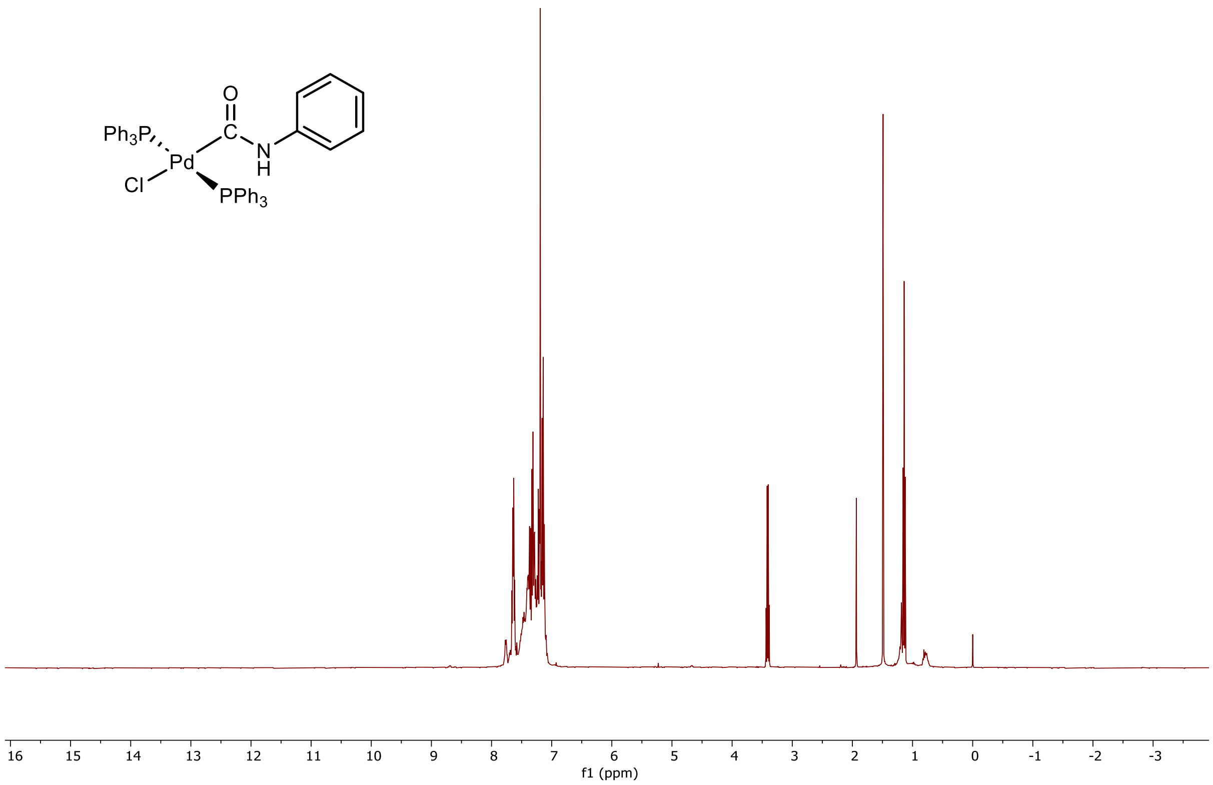

^{31}P-NMR

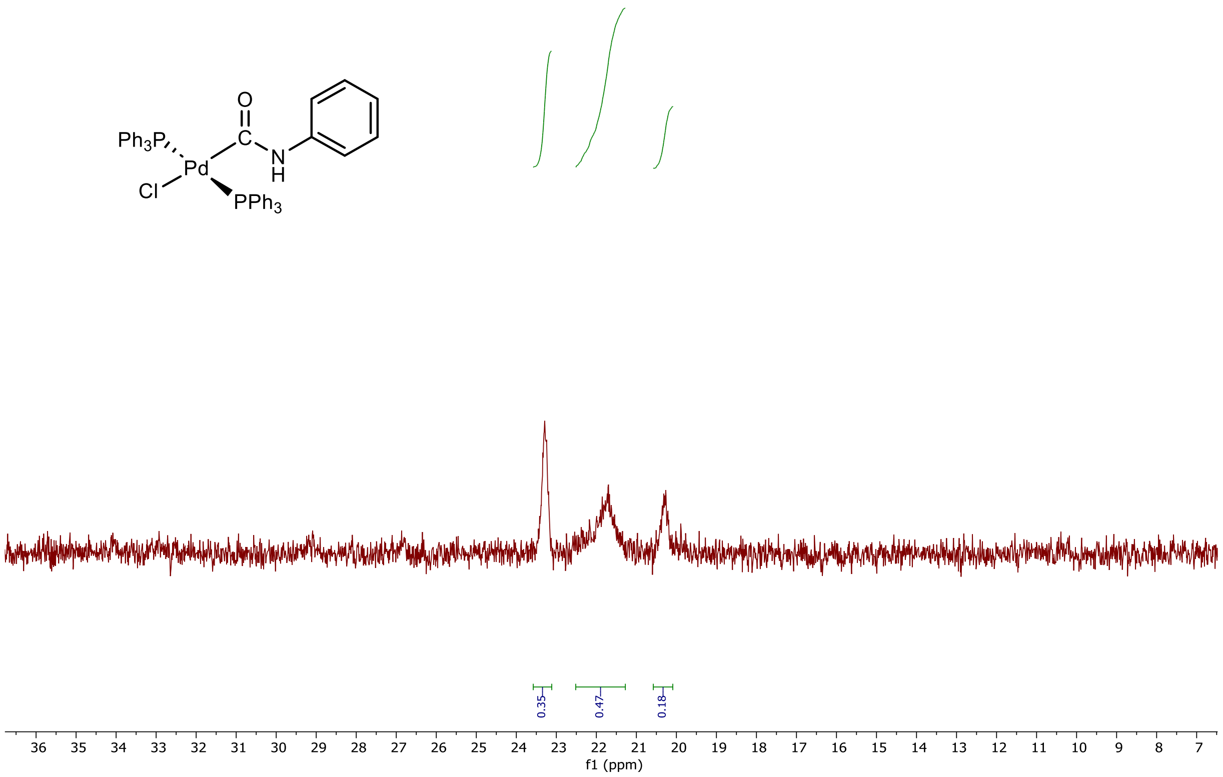

***trans*-Chloro(4-(*para*-toluidine)-carbonyl)bis(triphenylphosphine) palladium(II) (Pd-20)**

^{1}H-NMR

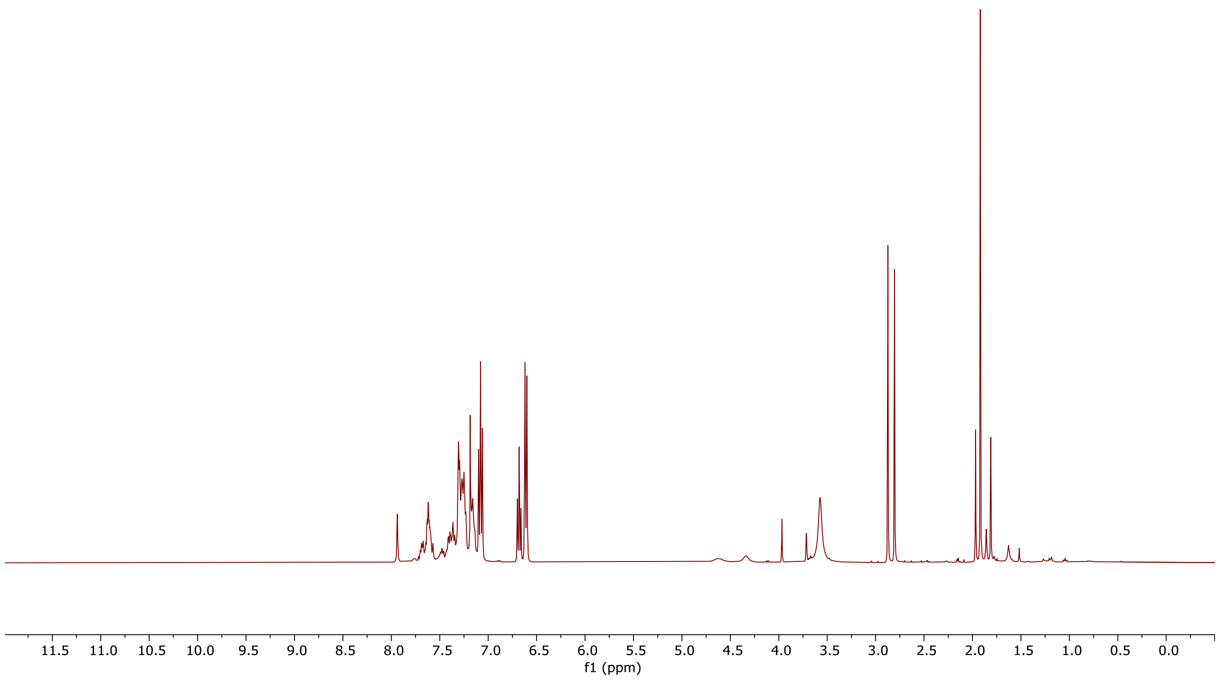

^{31}P-NMR

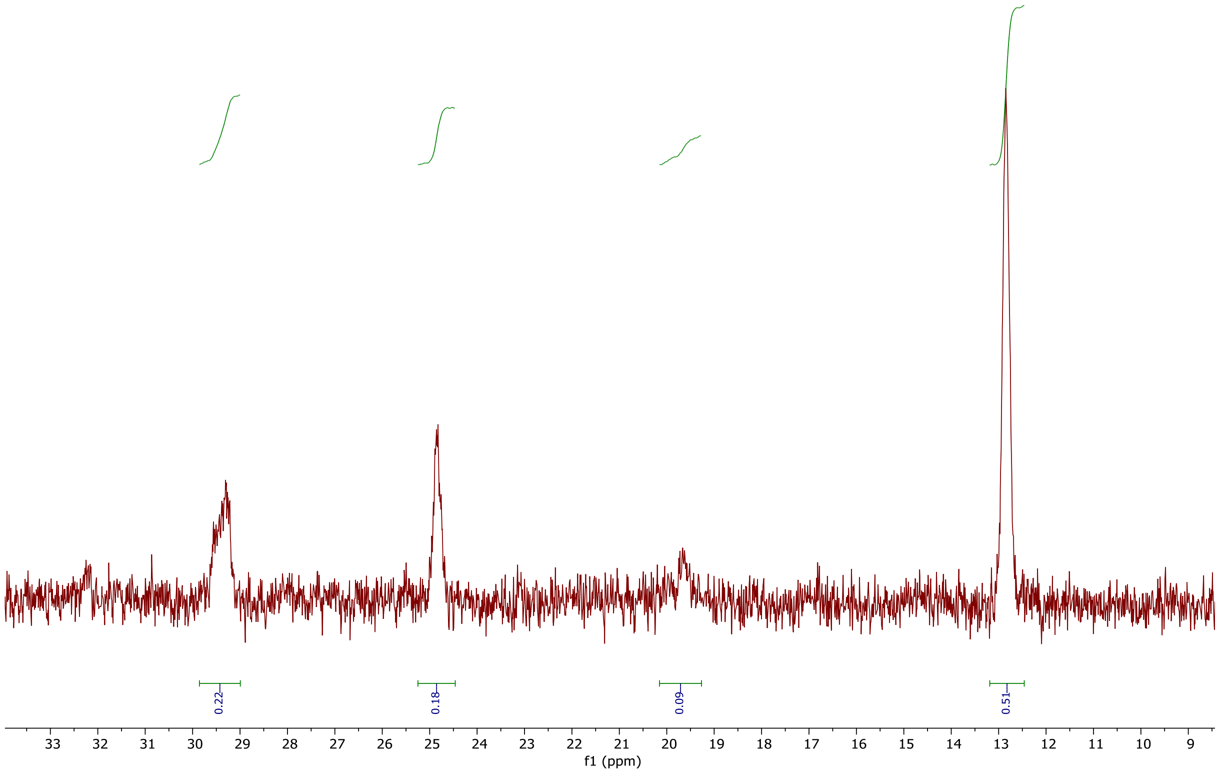

Supplement: Supplementary file 1 — The authors have cited additional references within the Supporting Information section [25, 29, 49, 50, 51, 52, 53, 54, 55, 56, 57]. Supporting File: anie72717‐sup‐0001‐SuppMat.pdf. [file ANIE-65-e1188892-s001.pdf]
